# Isothiourea-Catalyzed Enantioselective Functionalisation of Glycine Schiff Base Aryl Esters via 1,6- and 1,4-Additions

**DOI:** 10.1002/ceur.202300015

**Published:** 2023-05-03

**Authors:** Lotte Stockhammer, Rebecca Craik, Uwe Monkowius, David B. Cordes, Andrew D. Smith, Mario Waser

**Affiliations:** a Institute of Organic Chemistry, Johannes Kepler University Linz, Altenbergerstrasse 69, 4040 Linz (Austria); b EaStCHEM, School of Chemistry, University of St Andrews, KY16 9ST St Andrews, Fife, (UK); c School of Education, Chemistry, Johannes Kepler University Linz, Altenbergerstrasse 69, 4040 Linz (Austria)

**Keywords:** 1,6- and 1,4-addition, α-amino acid derivatives, enantioselective, glycine Schiff base, isothiourea

## Abstract

The enantioselective α-functionalisation of glycine Schiff base aryl esters through isothiourea catalysis is successfully demonstrated for 1,6-additions to para-quinone methides (21 examples, up to 95:5 dr and 96:4 er) and 1,4-additions to methylene substituted dicarbonyl or disulfonyl Michael acceptors (17 examples, up to 98:2 er). This nucleophilic organocatalysis approach gives access to a range of α-functionalised α-amino acid derivatives and further transformations of the activated aryl ester group provide a straightforward entry to advanced amino acid-based esters, amides or thioesters.

## Introduction

**Scheme 1:**
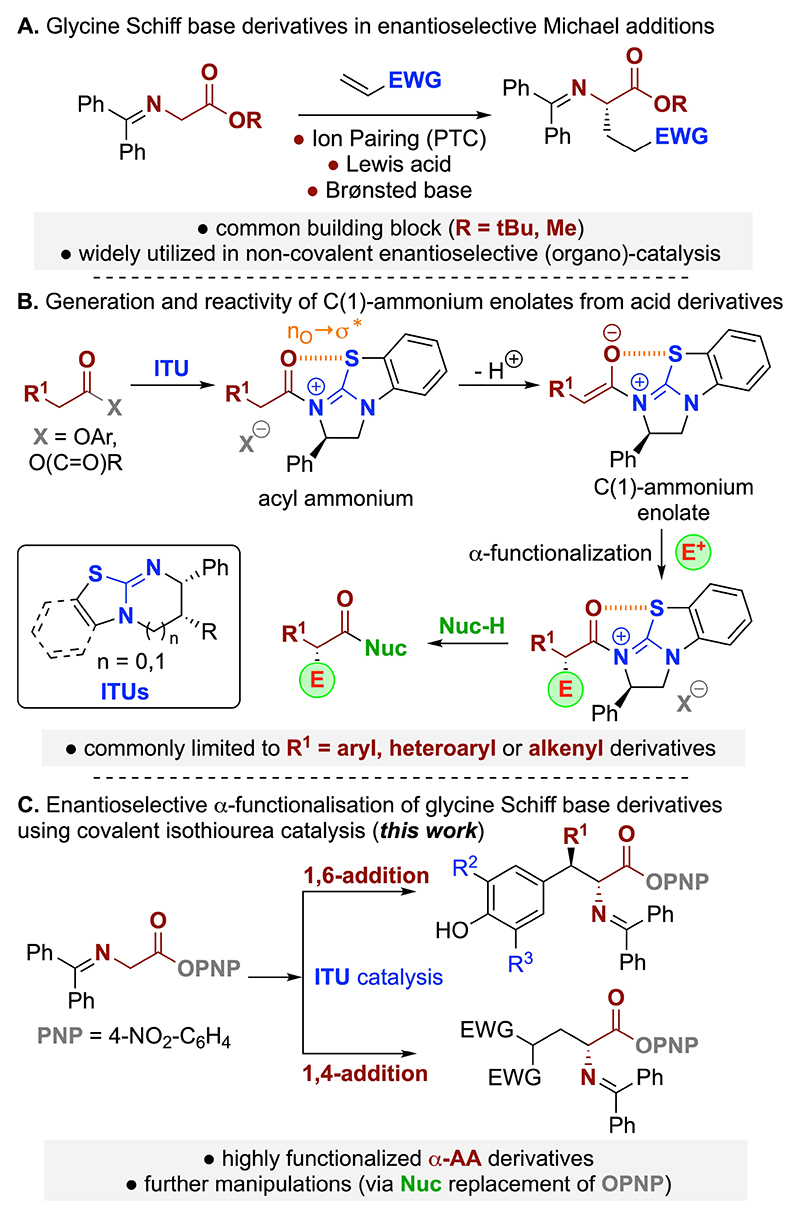
(a) Established strategies for glycine Schiff base Michael addition reactions. (b) The generation of C(1)-ammonium enolates and their established synthetic utilization. (c) The merger of these two concepts to carry out the α-functionalization of glycine Schiff bases under covalent ITU catalysis.

The development of new catalytic enantioselective methods to access enantioenriched α-amino acid derivatives (α-AA) is of huge significance and interest to the synthetic community.^[1–9]^ A plethora of effective synthetic methods has been developed and widely reviewed,^[2–9]^ allowing for the synthesis of non-natural α-amino acid derivatives that incorporate novel structural motifs not found within the chiral pool.^[2]^ One of the most common and direct strategies in this area is to selectively α-functionalise glycine derivatives via formal generation of a nucleophilic glycine anion equivalent.^[4,5]^ Of the many methods developed, the enantioselective α-functionalisation of glycine derived Schiff base derivatives may be the most popular.^[5]^ Since the pioneering work of O’Donnell^[10,11]^ who utilized them as substrates for enantioselective ammonium salt phase transfer catalysis (PTC),^[12]^ they have since been used and developed in a range of enantioselective (organo)-catalytic processes such as α-C_sp_^3^-alkylations, Michael reactions, or Mannich type approaches (considering the most valuable C−C-bond forming strategies only).^[5,13]^ Michael type additions are of fundamental importance to access structurally diverse chiral target molecules^[14]^ and the use of glycine Schiff bases for asymmetric Michael type reactions has been successfully explored using non-covalent (organo)-catalytic strategies through exploiting phase transfer ion pairing catalysis, Brønsted base catalysis and Lewis acid approaches (Scheme 1A).^[5,15]^ Usually, these *non-covalent* activation strategies allow for high levels of stereo-selectivity especially when a sterically demanding t-butyl ester of the glycine Schiff base is used as a starting material (Me-esters have been used too but usually with lower selectivities only). However, due to the stability of this ester further direct manipulations such as transamination or transesterification require a stepwise approach, consisting of ester hydrolysis followed by activation of the free carboxylic acid towards nucleophilic attack.

To the best of our knowledge the enantioselective functionalisation of glycine Schiff base derivatives through *covalent* catalytic activation strategies such as asymmetric Lewis base catalysis has not yet been developed. Within this area, the generation and reactivity of C(1)-ammonium enolates using tertiary amine Lewis bases as catalysts has been widely exploited.^[16]^ Originally exploiting isolable or in-situ generated ketenes from acid chlorides as starting materials,^[17]^ in recent years their in-situ generation from either carboxylic acids or electron-deficient aryl ester derivatives (i. e. p-nitrophenol (PNP) or pentafluorophenol (PFP)) has been extensively investigated.^[18]^ In particular, the use of easily accessible (chiral) isothioureas (ITUs) as Lewis base catalysts in enantioselective transformations has developed widely in this context.^[19]^ The unique potential of this activation strategy lies in the fact that the covalently bound catalyst forms a well-defined chiral C(1)-ammonium enolate (Scheme 1B) which is capable of reacting in a highly face-selective manner with a range of either C- or heteroatom-electrophiles.^[20]^ After the asymmetric α-functionalization the catalyst is liberated by nucleophilic displacement either in an *intermolecular* manner (i. e. from an aryl ester starting material the generated aryloxide will promote turnover to give an activated aryl ester suited for further displacements)^[21]^ or via an *intramolecular* cyclization (when carrying out Michael type additions where the in situ formed enolate acts as the catalyst releasing nucleophile). A frequently encountered limitation in this area is the restricted structural variation allowed within the α-substituted carboxylic acid or ester derivative that can be employed in *intermolecular* reactions. Specifically, most processes require an α-aryl-, α-heteroaryl,^[22]^ or α-alkenyl-substituted derivative^[23]^ to achieve reactivity, with limited exceptions within the literature (Scheme 1B).^[24]^ In this context, and as part of ongoing studies concerning the exploitation of isothioureas in C(1)-ammonium enolate generation, in this manuscript we demonstrate that glycine Schiff base-derived activated aryl esters are viable precursors for enantioselective 1,6- and 1,4-conjugate additions using isothioureas as Lewis base organocatalysts (Scheme 1C). To the best of our knowledge this represents the first example for a *covalent* asymmetric organocatalytic activation of glycine Schiff bases, providing a conceptually unprecedented approach to access α-amino acid derivatives. In addition, considering the reactivity of the activated aryl ester, further direct manipulations by transamination and transesterification are also demonstrated.

## Results and Discussion

### Synthesis of the activated glycine Schiff base 1

**Scheme 2:**
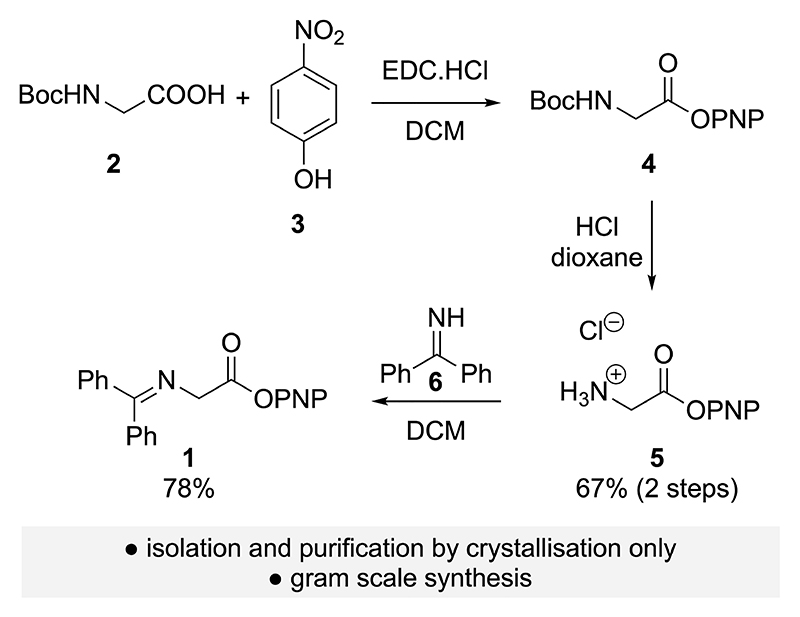
Synthesis of the glycine Schiff base PNP ester **1**.

The syntheses of glycine Schiff base alkyl esters (such as the t-butyl ester, Scheme 1A) are well established.^[5,25]^ However, due to the more reactive nature of the targeted electron-deficient aryl ester variants a careful optimization of the synthesis procedure was necessary.^[26]^ As outlined in Scheme 2, synthesis of the PNP ester **1** was possible by starting from N-Boc-protected glycine (**2**) via an esterification – Boc-deprotection – imine formation sequence. Because of the pronounced sensitivity of the activated ester towards hydrolysis column chromatographic purification was omitted, with isolation and purification by crystallization and trituration developed, allowing a scalable and robust process to access **1** on gram scale. The same strategy was also tested to access the analogous PFP ester, but unfortunately isolation in satisfactory purity by crystallization or chromatography was not possible due to its sensitivity to hydrolysis.

### Asymmetric 1,6-conjugate addition reactions of 1

**Figure 1:**
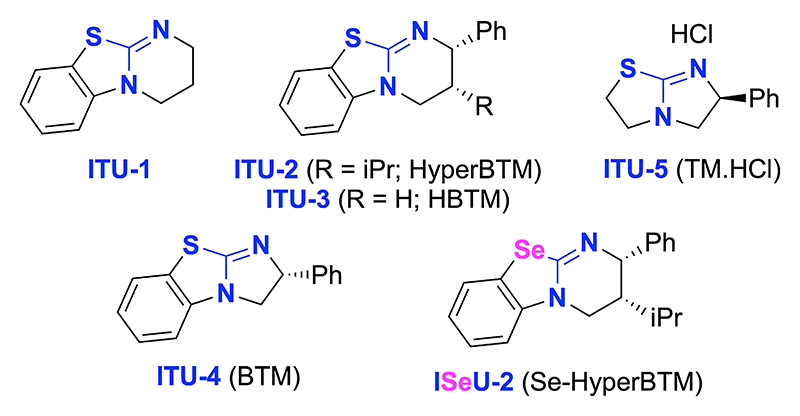
Isothio- and isoselenourea catalysts tested herein.

The asymmetric 1,6-addition of glycine Schiff base alkyl esters to para-quinone methides (p-QMs, **7**)^[27,28]^ has previously been demonstrated utilizing chiral ammonium salt phase transfer catalysts and Cu-catalysts.^[29]^ Considering the structural diversity of β-functionalized α-AA derivatives that should be accessible by reacting aryl ester Schiff base **1** with p-QMs **7**,^[30]^ and the potential of the products to undergo further transesterifications or transaminations, the addition of **1** to p-QM **7a** utilizing established Lewis base catalysts (Figure 1) was investigated.^[31]^

Table 1 gives an overview of a detailed screening and optimization of the addition of **1** to **7a**. First experiments showed that the uncatalyzed racemic background reaction is facile (entry 1) and that the addition of achiral **ITU-1** does not lead to enhanced yield (entry 2). First trials with the established chiral catalysts HyperBTM (**ITU-2**, entry 3) and HBTM (**ITU-3**, entry 5) as well as the HyperBTM isoselenourea analogue **ISeU-2** (entry 4) gave only moderate levels of enantioselectivity. Interestingly the product er increased significantly when using the bicyclo[3.3.0]octene-based ITUs BTM (**ITU-4**) and tetramisole hydrochloride (TM·HCl, **ITU-5**). Both of these catalysts allowed for promising initial enantioselectivities higher than 80:20 (entries 6 and 7) for the major diastereoisomer. Further optimization of the reaction conditions used **ITU-5** as a catalyst (entries 7–16). After testing different solvents and bases, as well as variation of catalyst loading and temperature, the use of low temperature conditions allowed satisfactory isolated yields (77%), good diastereoselectivity (85:15), and high enantioselectivity (93:7) for the major diastereoisomer (entry 16). It is noteworthy that the minor diastereomer was obtained in essentially racemic form. Product **8a**, as well as the other addition products of ester **1** described herein, do not undergo further alkylation when treated with an excess of acceptor and ITU, nor do they epimerize when stirred with an achiral ITU. These results indicate that addition of the ITU is much more hindered for α-alkylated products like compound **8a** as compared to the prochiral substrate **1**.

**Scheme 3:**
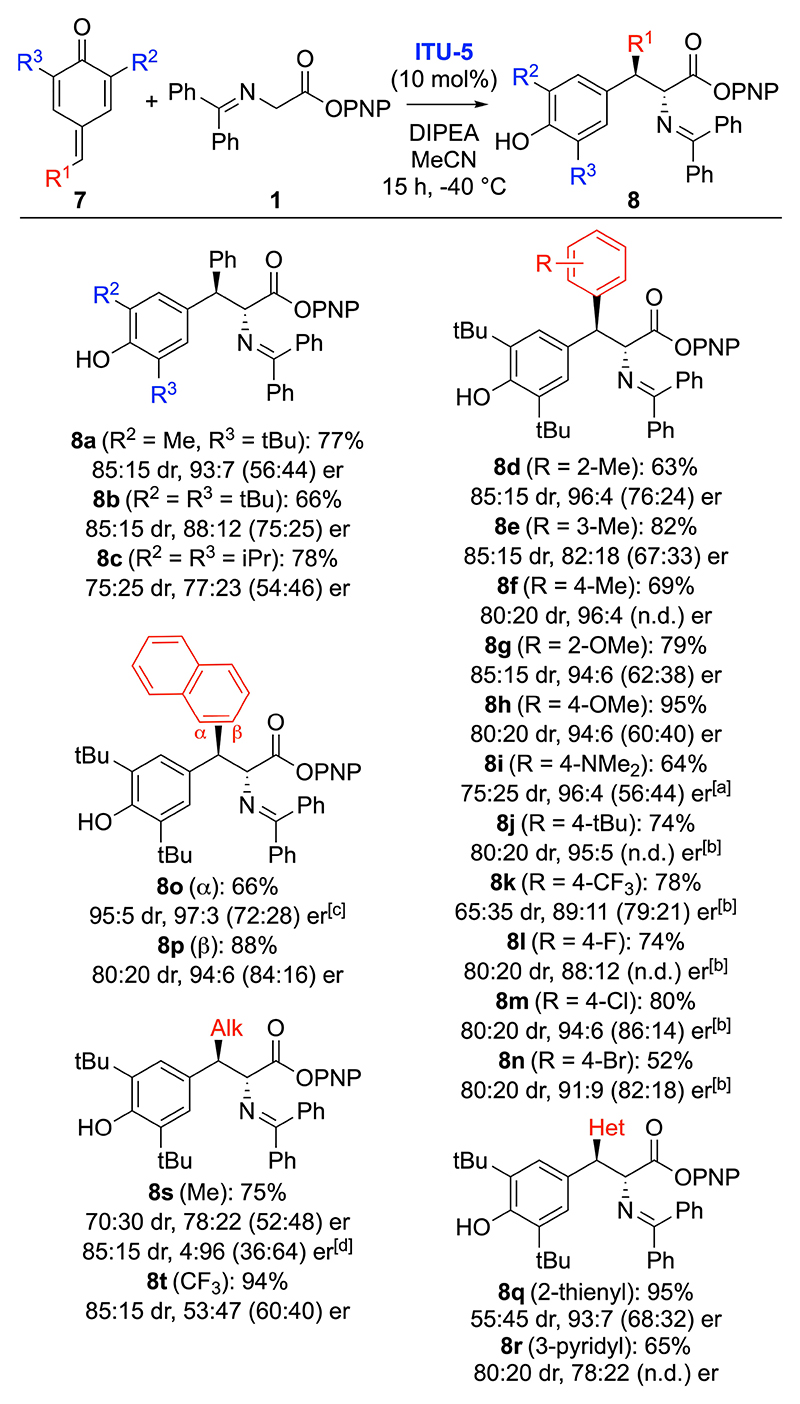
Asymmetric application scope of the 1,6-addition of **1** to p-QMs **7**. [a] Due to solubility issues this reaction was carried out in CH_2_Cl_2_ for 64 h. [b] er was determined by analysing the corresponding morpholine amides (compare with Scheme 4, compound **9 b**). [c] 1 mmol scale. [d] Using **ITU-4**.

Having identified suited conditions for the asymmetric addition of **1** to p-QM **7a** (Table 1, entry 16) the scope for this transformation was investigated (Scheme 3). A variety of different arylidene-based p-QMs **7** were reasonably well tolerated (giving products **8 a**–**r**) although the nature of the substituents had a notable effect on diastereo- as well as enantioselectivity. For example, the α-naphthyl-containing product **8o** was accessible with very high selectivity (95:5 dr, 97:3 er on 1 mmol scale) while the pyridyl-based product **8r** was obtained with lower levels of stereoselectivity (80:20 dr, 78:22 er).

In addition, the use of two alkylidene-based p-QMs gave products **8s** and **8t**. Interestingly, while the use of the p-CF_3_-containing p-QM^[32]^ (giving **8t**) gave poor enantioselectivity, the methyl-substituted product **8s** was obtained in high selectivity when using BTM (**ITU-4**) instead of **ITU-5** (it should be noted that **ITU-4** was also tested for several arylidene-based QMs but in those cases **ITU-5** was found to be superior).

**Scheme 4:**
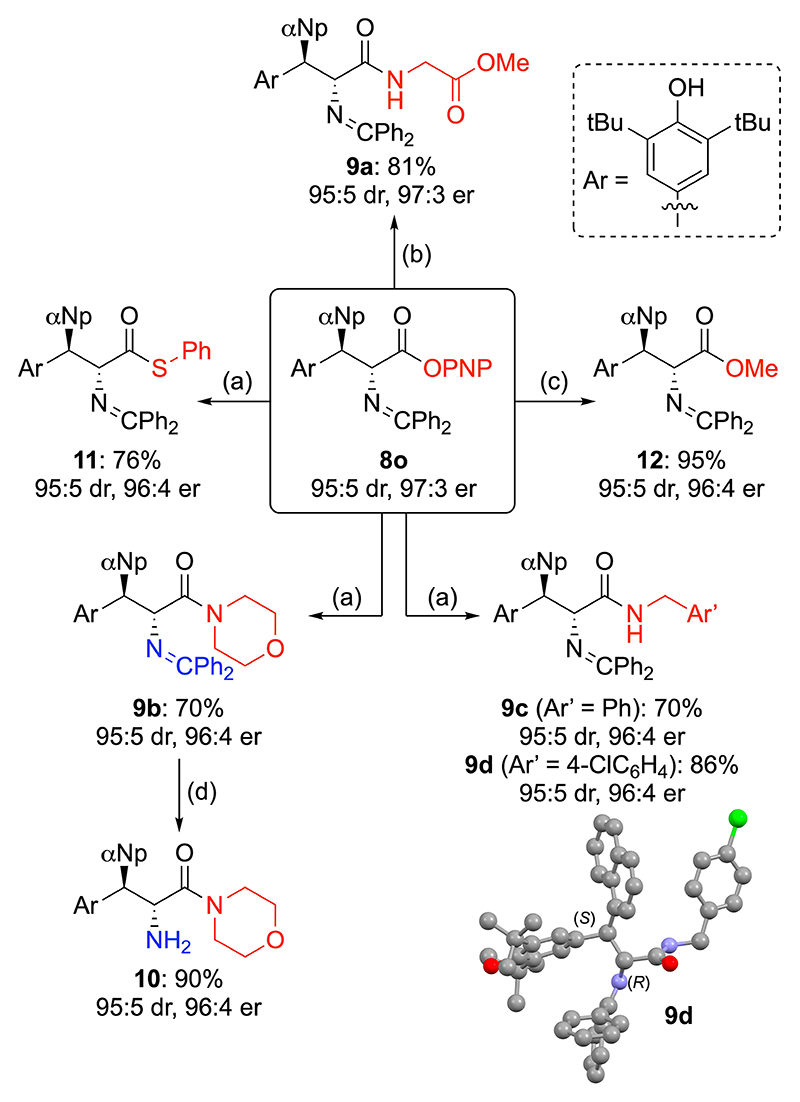
Further functionalizations of the PNP ester **8o**. Conditions: a) nucleophile (benzylamines, morpholine, thiophenol), DMAP, MeCN, r. t.; b) methyl glycinate hydrochloride, DIPEA, DMAP (20 mol%), MeCN, r. t.; c) DMAP, MeOH, r. t.; d) HCl (1 M), THF, 0 °C.

**Scheme 5:**
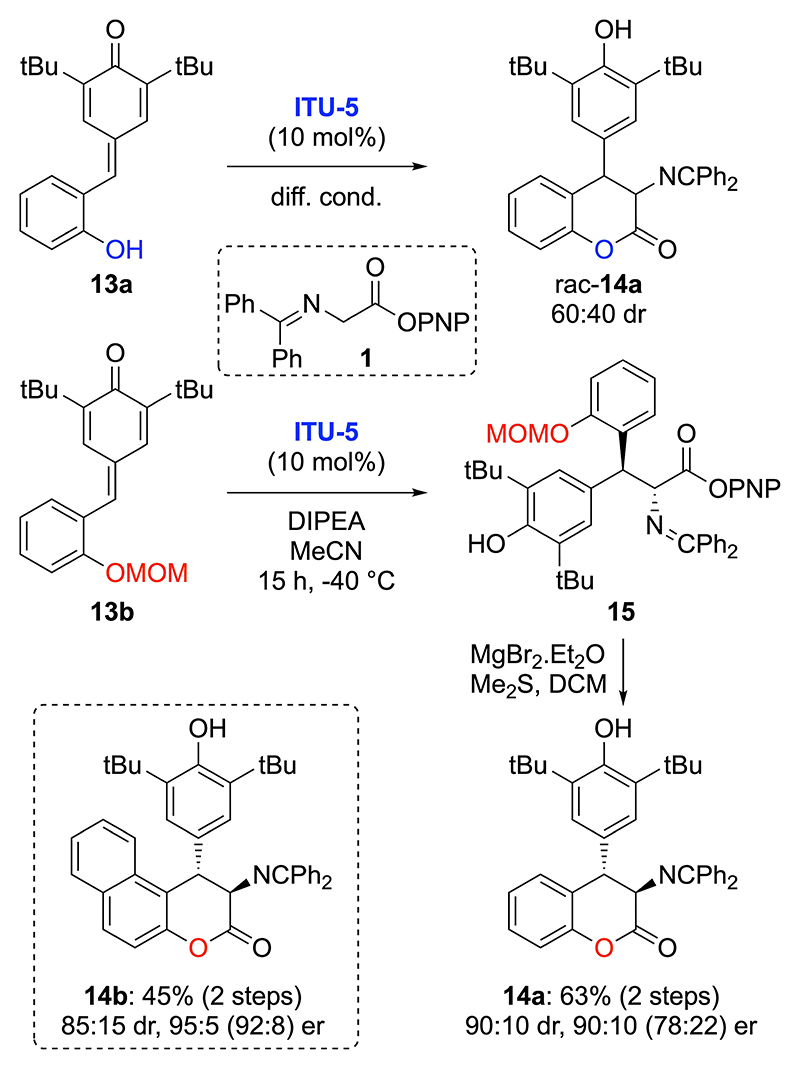
Use of o-OH-containing p-QMs **13**.

The high reactivity of electron-deficient aryl esters was expected to allow efficient follow up transformations. The suitability of the α-Np-based product **8o** to undergo further trans(thio)esterifications and transaminations was investigated (Scheme 4) demonstrating that **8o** reacts readily with primary and secondary amines to access dipeptide **9a** and the amides **9b**–**d** in good chemical yields without any erosion of er or dr The relative and absolute configuration of secondary amide **9d** was determined by single crystal X-ray diffraction analysis,^[33]^ with all other products **8**–**12** assigned by analogy (in addition to comparison with literature data).^[29]^ Alongside these amide-formations, synthesis of the thioester **11** as well as the methylester **12** was also achieved.

o-OH-containing p-QMs like compound **13a** are versatile precursors to access (chiral) bicyclic target molecules upon reaction with suited (pro)-nucleophiles.^[34]^ The ITU-catalysed addition of glycine Schiff base **1** to compound **13a** (Scheme 5). led to direct formation of the targeted product **14a** but in a racemic manner (and modest diastereoselectivity) despite extensive testing of catalytic conditions. The most rational explanation for this outcome is that addition of the phenol group to the activated ester or acyl ammonium intermediate proceeds first, followed by 1,6-addition to the p-QM motif in an intramolecular manner without the ITU-catalyst being covalently bound.^[35]^ A variety of O-protected p-QMs **13** was next tested, with the MOM-protected derivative **13b** giving addition product **15**. Further treatment of **15** with MgBr_2_ and Me_2_S^[36]^ allowed for MOM-deprotection and concomitant cyclization giving product **14a** in good overall yield (63% over two steps) and good diastereo- and enantioselectivity (90:10 dr, 90:10 er). By utilizing the analogous naphthyl-based starting material, the corresponding product **14b** could be accessed with significantly higher levels of stereoselectivity.

### Asymmetric 1,4-conjugate addition reactions of 1

**Scheme 6:**
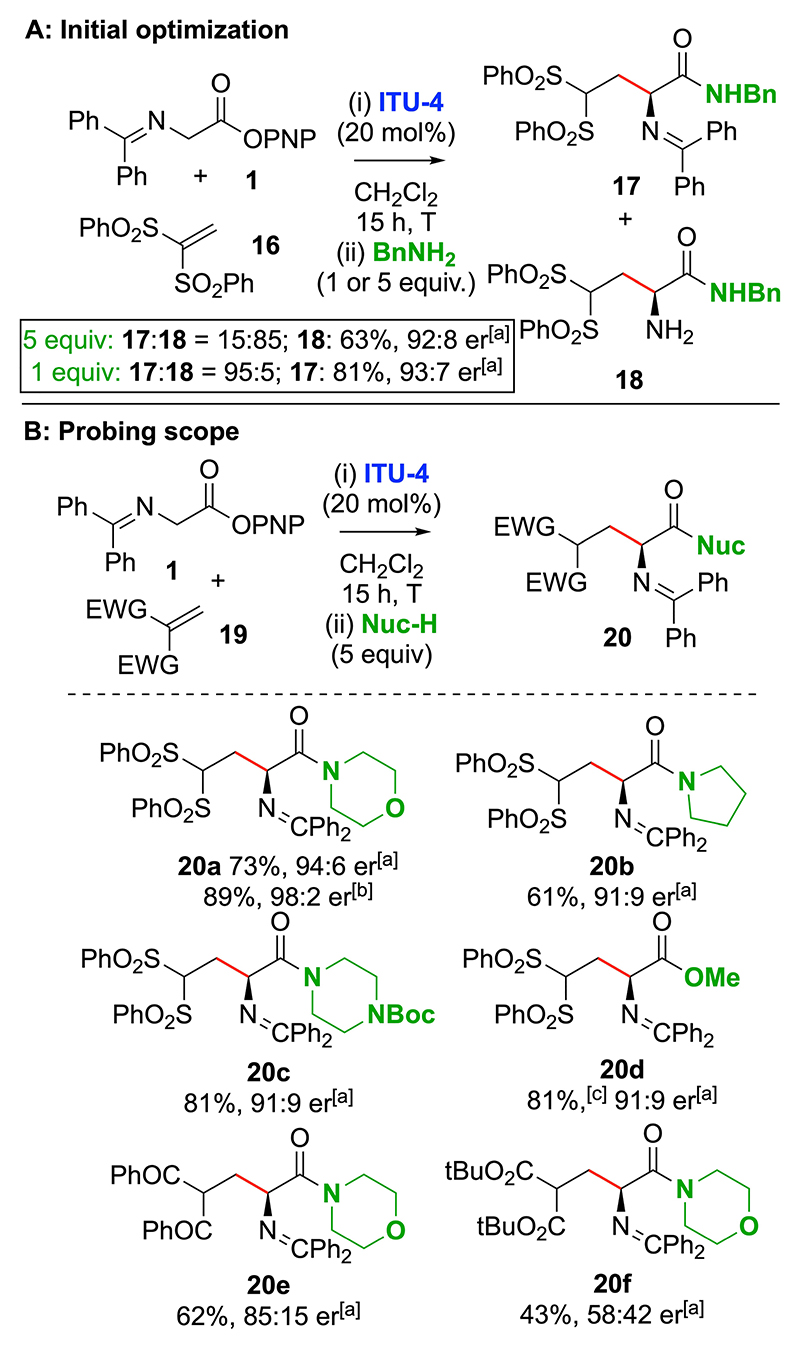
Initial optimisation of reaction with bis-sulfone electrophile **16**. [a] er determined by HPLC analysis on a chiral stationary phase. [b] **16** added dropwise by syringe-pump over 6 h. [c] For step (ii) MeOH and DMAP (20 mol%) were used.

Subsequent work considered the ITU-catalysed addition of glycine Schiff base **1** to α,β-unsaturated bis-sulfone Michael acceptor **16** (Scheme 6A). In this series, while isolation of the corresponding PNP ester product proved facile, assessment of its enantiopurity by HPLC analysis was unreliable, and consequently addition of benzylamine to yield a stable derivative **17** was investigated. Screening of a range of ITUs showed that **ITU-4** ((*R*)-BTM) gave the highest product er, while using an excess (5 equiv.) of benzylamine led to a 15:85 mixture of amine **18** (92:8 er) to imine **17** as a result of transamination and amide formation. However, using 1 equiv. of benzylamine led to selective amide formation, giving **17** in 81% yield and 93:7 er. Further investigation showed that this competitive transamination event did not occur upon addition of secondary amines, with morpholine, pyrrolidine and N-Boc-piperazine giving the corresponding amides **20a**–**20c**, while methanol gave the ester **20d** in good yield and er (61–81% yield, up to 94:6 er, Scheme 6B). In the case of **20a**, further optimisation showed that dropwise addition of the bis-sulfone Michael acceptor **16** allowed the catalyst loading to be reduced to 5 mol%, giving **20a** in 89% yield and 98:2 er. Unfortunately, extension of these protocols to alternative di-ketone or diester Michael acceptors gave **20e** and **20f** with reduced product er, particularly for diester product **20f** (58:42 er), necessitating re-optimisation to find a generally applicable protocol.

**Scheme 7:**
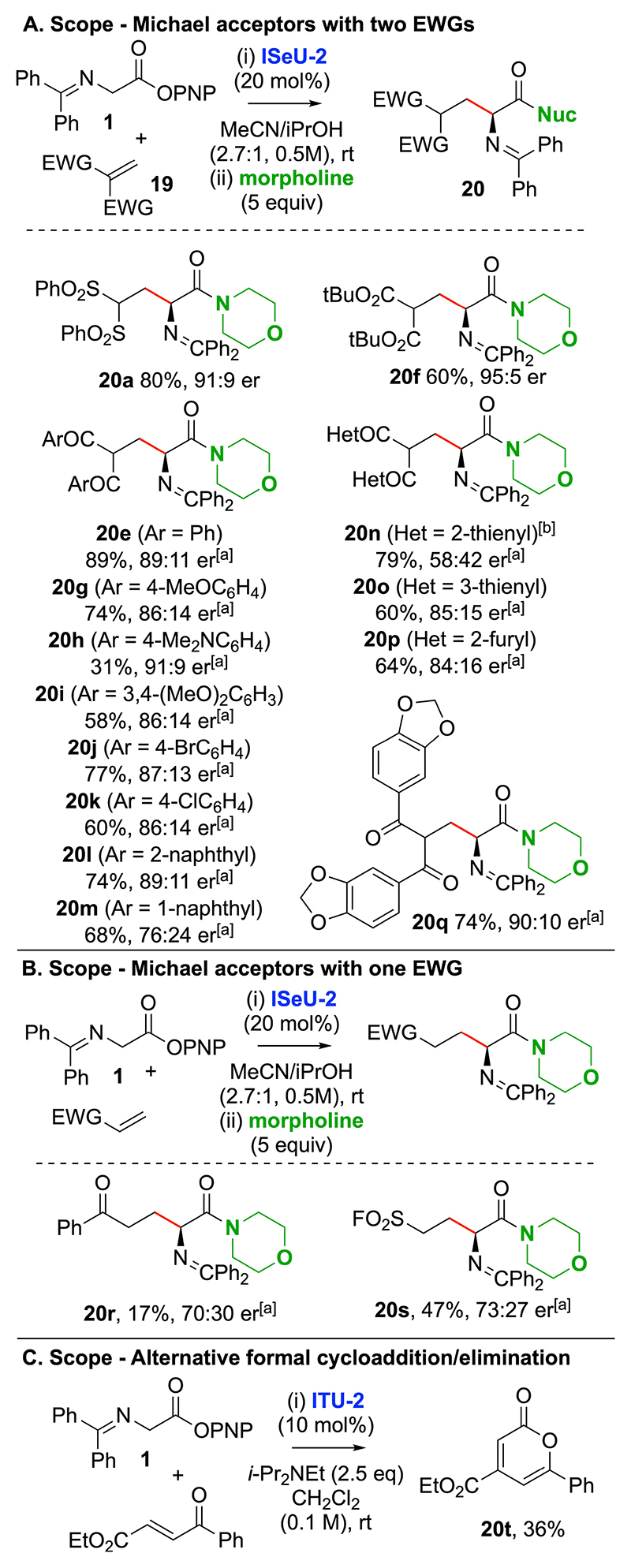
Scope and limitations using (2*S*,3*R*)-HyperSe (**ISeU-2**) in MeCN/iPrOH. [a] er determined by HPLC analysis on a chiral stationary phase. [b] Reaction carried out in EtOAc; in MeCN/iPrOH the corresponding isopropyl ester was isolated in 68% yield and 66:34 er.

Further investigation re-optimised the reaction process to find a more general protocol applicable to bis-sulfone, di-ketone and di-ester Michael acceptors (Scheme 7A). Screening of a range of ITU catalyst and solvent combinations (see Supporting Information for full optimisation) showed that the use of the recently developed isoselenourea **ISeU-2** (20 mol%) in an MeCN/iPrOH solvent mixture provided the most generally applicable protocol, giving bis-sulfone derivative **20a** in 80% yield and 91:9 er, di-ester derivative **20f** in 60% yield and 95:5 er, and diketone derivative **20e** in 89% yield and 89:11 er. The generality of this process was explored through extension to a range of di-aryl ketone Michael acceptors, with electron donating (4-OMeC_6_H_4_; 4-NMe_2_C_6_H_4_; 3,4-(OMe)_2_C_6_H_3_), halogen (4-BrC_6_H_4_; 4-ClC_6_H_4_), 1- and 2-naphthyl, as well as heterocyclic variants (2-thienyl, 3-thienyl; 2-furyl) explored, giving products **20g**–**20q** in up to 74% yield and 90:10 er. Interestingly the 2-thienyl derivative gave preferentially the corresponding isopropyl ester (68% yield, 66:34 er) under the standard conditions, with EtOAc used as the reaction solvent to generate **20n**. Unfortunately, extension to the use of alternative Michael acceptors containing a single electron withdrawing substituent (COPh or SO_2_F) led to consistently low product yields and enantioselectivity (Scheme 7B). Furthermore, the use of ethyl (*E*)-4-oxo-4-phenylbut-2-enoate led to the formation of **20t**, presumably arising from formal [4 + 2]-cycloaddition and elimination, in unoptimized 36% yield (Scheme 7C).

**Scheme 8:**
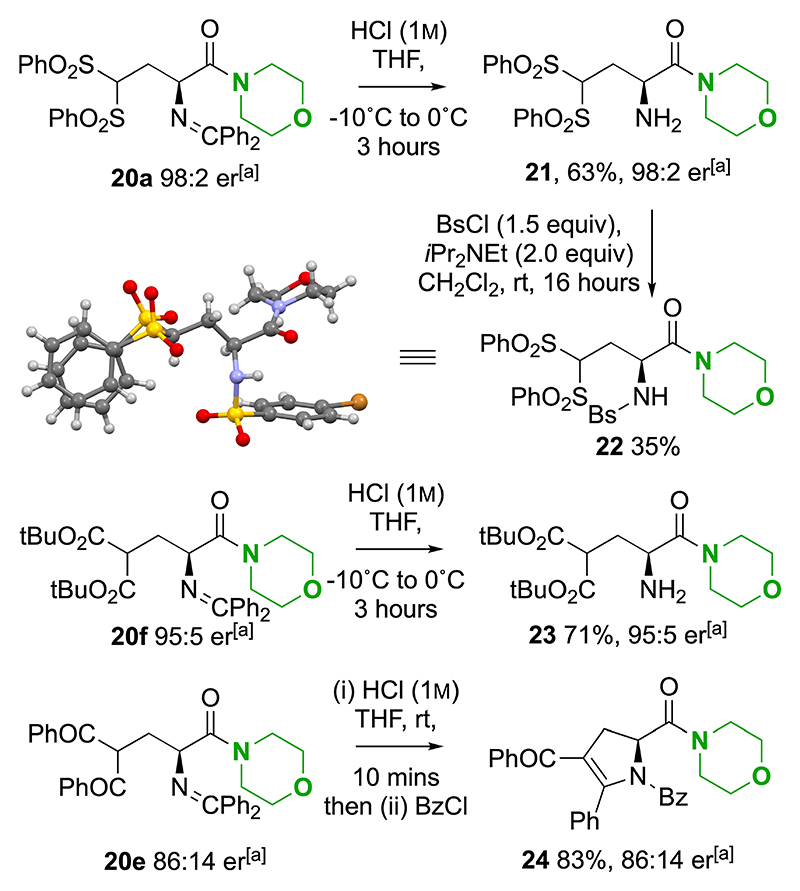
Further product derivatization. [a] er determined by HPLC analysis on a chiral stationary phase; dr determined by ^1^H NMR spectroscopic analysis. Bs=4-bromobenzenesulfonyl.

Having demonstrated the scope of this transformation, further derivatisation focused upon hydrolysis of the Schiff base imine functionality to yield the free amine by treatment with 1 M HCl in THF. This was readily applied to the bis-sulfone **20a** and diester **20f** derivatives (Scheme 8), giving the desired amines **21** and **23** respectively in good yields and without erosion of enantiopurity. The absolute configuration within **21** was established unambiguously by derivatisation to N-4-bromobenzenesulfonyl (NBs) derivative **22** and subsequent single crystal X-ray diffraction analysis.^[33]^ When applied to the corresponding diphenyl-ketone derivative **20e** (86:14 er), imine hydrolysis, followed by intramolecular cyclisation onto the dicarbonyl gave a mixture of tautomeric species, that followed by treatment with benzoyl chloride gave N−Bz protected pyrrolidine **24** in 86:14 er and excellent yield.

## Conclusions

In conclusion, this manuscript demonstrates the enantioselective α-functionalisation of glycine Schiff base aryl esters through chiral isothiourea catalysis. Successful applications in the 1,6-additions to para-quinone methides (21 examples, up to 95:5 dr and 96:4 er) and 1,4-additions to methylene substituted dicarbonyl or disulfonyl Michael acceptors (17 examples, up to 98:2 er) demonstrate the generality of this Lewis base activation protocol. Overall, this approach gives access to a range of α-functionalised α-amino acid derivatives, with further transformations of the activated aryl ester group and the imine protecting group providing straightforward access to a range of advanced amino acid-based esters, amides or thioesters as well as pyrrolidine derivatives.^[37]^

### Experimental Section

General experimental details, further optimizations, analytical details and characterization, as well as copies of NMR spectra and HPLC chromatograms can be found in the online Supporting Information.

#### General procedure for the 1,6-addition of 1 to p-QMs

Schiff base **1** (1 equiv.) and TM.HCl (**ITU-5**; 10 mol%) were dissolved in freshly degassed MeCN (0.07 mol L^−1^) at −40 °C. Then, DIPEA (1 equiv.) was added and the mixture was stirred for 5 min. The respective quinone methide (1 equiv.) was added in one portion and the mixture was further stirred for 15 h. After this, the circulation chiller was turned off, and the mixture was allowed to reach rt over 1 h. It was filtered over Na_2_SO_4_ and washed with CH_2_Cl_2_ (3×). The filtrate was concentrated on the rotary evaporator and the crude products were purified by column chromatography on deactivated silica (heptanes followed by heptanes/EtOAc 10/1 and 5/1). Products were obtained as a mixture of diastereomers in indicated yields and enantiopurities.

#### General procedure for the 1,4-addition of 1 to different Michael acceptors

The Schiff base **1** (1.0 equiv.), the Michael acceptor (1.0 equiv.) and the ITU or ISeU (20 mol%) were added to a flame dried vial. IPA (0.5 M) and MeCN (1.3 M) were then added, and the reaction was stirred at rt and monitored by ^1^H NMR. When all the Michael acceptor was consumed, the nucleophile (Nuc-H) was added, and the reaction was stirred for 10 min at rt. The reaction mixture was diluted with EtOAc (40 volumes) and NaOH (1 M) solution (40 volumes). The aq. layer was extracted with EtOAc (× 2 vol). The organic layer was then washed with NaOH (1 M) (× 2 vol), brine (× 1 vol), dried over MgSO_4_, filtered, and concentrated *in vacuo*. The crude residue was purified by column chromatography using the solvent system stated in the Supporting Information. Products were obtained as a mixture of diastereomers in indicated yields and enantiopurities.

## Supplementary Material

SI

## Figures and Tables

**Table 1:** Optimization of the 1,6-addition of glycine Schiff base **1** to p-QM **7a**.^a^

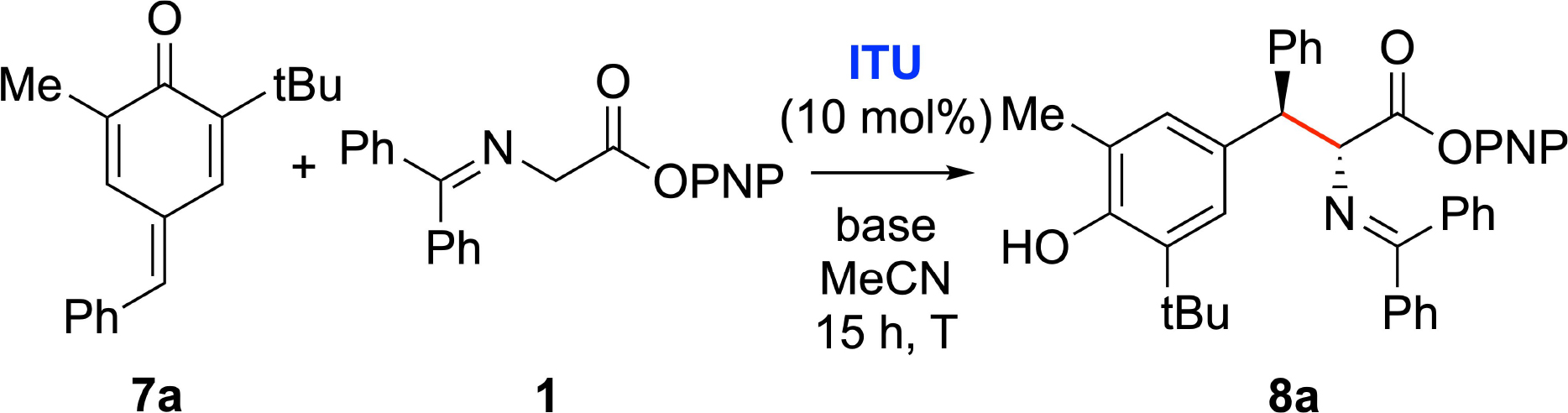						
Entry	ITU	Base	*T* [°C]	Yield[%]^b^	dr^c^	er^d,e^
1	–	Et_3_N	25	70	75:25	–
2	**ITU-1**	Et_3_N	25	53	75:25	–
3	**ITU-2**	Et_3_N	25	85	70:30	42:58 (45:55)
4	**ISeU-2**	Et_3_N	25	73	60:40	26:74 (44:56)
5	**ITU-3**	Et_3_N	25	79	75:25	38:62 (40:60)
6	**ITU-4**	Et_3_N	25	66	65:35	18:82 (69:31)
7	**ITU-5**	Et_3_N	25	66	75:25	84:16 (40:60)
8	**ITU-5**	DIPEA	25	67	75:25	90:10 (45:55)
9	**ITU-5**	DABCO	25	58	70:30	90:10 (40:60)
10	**ITU-5**	K_2_CO_3_	25	73	70:30	87:13 (40:60)
11^f^	**ITU-5**	DIPEA	25	41	60:40	89:11 (59:41)
12^g^	**ITU-5**	DIPEA	25	78	65:35	89:11 (38:62)
13	**ITU-5**(5%)	DIPEA	25	71	75:25	80:20 (46:54)
14	**ITU-5** **(20%)**	DIPEA	25	60	70:30	90:10 (43:57)
15	**ITU-5**	DIPEA	− 20	72	80:20	93:7 (52:48)
16	**ITU-5**	DIPEA	− 40	77	85:15	93:7 (56:44)

a All reactions were run using 0.05-0.1 mmol **1, 7a** (1 equiv.) and base (1 equiv.) in acetonitrile under the given conditions unless otherwise stated.

b Isolated yields of the mixture of diastereomers.

c Determined by 1^H^ NMR of the crude product.

d Determined by HPLC using a chiral stationary phase (values in brackets refer to the er of the minor diastereoisomer).

e For the major stereoisomer the depicted (*R*,*R*)-configuration was assigned in analogy to the results obtained by single crystal X-ray analysis of derivative **9d** (see below) and comparison of the analytical details of products **8** (Scheme 3) and derivatives thereof (Scheme 4) with reported data.^[29]^

f CH_2_Cl_2_ as the solvent.

g Acetone as the solvent.

## Data Availability

The data that support the findings of this study are openly available in University of St Andrews Research Portal. at https://doi.org/10.17630/631a7545-ad96-427c-aa3f-a18664e93537, reference number 37.
