# Supplementary material for: Isothiourea-Catalyzed Enantioselective Functionalisation of Glycine Schiff Base Aryl Esters via 1,6- and 1,4-Additions
Source: ChemistryEurope. Author manuscript; Available in PMC 2024 Jun 14. (PMC7616101; doi:10.1002/ceur.202300015)
Supplement: SI [file EMS196535-supplement-SI.pdf]

ChemSystemsChem

Supporting Information

# Isothiourea-Catalyzed Enantioselective Functionalisation of Glycine Schiff Base Aryl Esters via 1,6- and 1,4-Additions

Lotte Stockhammer[+], Rebecca Craik[+], Uwe Monkowius, David B. Cordes, Andrew D. Smith,* and Mario Waser*

## Contents


**1 General Information Linz** ........ 4

**2 General Information St. Andrews** ........ 5

**3 Preparation of starting materials** ........ 7

3.1 Synthesis of activated glycine Schiff base ester 1 ........ 7

3.2 General procedure A: Claisen condensation with ester ........ 9

3.3 General procedure B: Claisen condensation with acyl chloride ........ 9

3.4 General procedure C: synthesis of diketone Michael acceptors ........ 10

3.5 General procedure D: Synthesis of aryl p-quinone methides ........ 10

3.6 General procedure E: Synthesis of MOM protected QMs ........ 11

3.7 Synthesis of diketones ........ 12

3.8 Synthesis of Michael acceptors ........ 18

3.9 Synthesis of quinone methides ........ 27

**4 1,6-Addition of Glycine Schiff base aryl ester to p-quinone methides** ........ 35

4.1 General information on all reactions between Schiff bases and quinone methides ........ 35

4.2 General procedure F for the alkylation of Schiff base ester with *p*-QMs ........ 35

4.3 Derivatisation to the corresponding morpholine amide for HPLC analysis ........ 35

4.4 Products of asymmetric alkylation of glycine Schiff base with *p*-QMs ........ 36

4.5 Synthesis of Chroman-2-one derivatives 14 ........ 55

4.6 Further transformations ........ 57

4.7 Relative and Absolute Configuration of p-QM-based 1,6-addition products ........ 63

**5 1,4-Conjugate additions of Glycine Schiff base aryl ester to Michael acceptors** ........ 65

5.1 General procedure G: Michael addition reactions of Glycine Schiff base esters ........ 65

5.2 General procedure H: Michael addition reactions of Glycine Schiff base esters using slow addition ........ 65

5.3 Products of asymmetric 1,4-additions of Glycine Schiff base aryl ester to Michael acceptors .. 66

5.4 Product derivatisations ........ 87

5.5 Optimisation tables for Michael addition reactions ........ 91

5.5.1. Optimisation of reaction with electrophile 16 ........ 91

5.5.2. Optimisation of reaction with electrophile **S39** ........ 94

5.6 Identification of side-products observed during the optimisation ... 95

5.7 Absolute configuration ... 97

**References ... 98**

**6 Appendix: Analytical data** ... 101

# 1 General Information Linz

NMR spectra were recorded on a Bruker Avance III 300 MHz spectrometer with a broad band observe probe and a sample changer for 16 samples which is property to the Austro Czech NMR Research Center “RERI uasb”. All NMR spectra were referenced on the solvent residual peak ($CDCl_3$: δ 7.26 ppm for $^1$H NMR and δ 77.16 ppm for $^{13}$C NMR). NMR data are reported as follows: chemical shift (δ ppm), multiplicity (s = singlet, d = doublet, t = triplet, q = quartet, m = multiplet, br = broad), coupling constants (Hz) and integrals.

High resolution mass spectra were obtained using an Agilent QTOF 6520 with ESI source. Optical rotations were measured on a Schmidt+Haensch Unipol L 100 polarimeter ($[\alpha]_D$ values are listed in deg·cm$^3$·g$^{-1}$·dm$^{-1}$; concentration *c* is given in g/100 mL).

Preparative column chromatography was carried out using Davisil LC 60A 70–200 MICRON silica gel. Thin layer chromatography was performed on Macherey-Nagel pre-coated TLC plates (silica gel, 60 $F_{254}$, 0.20 mm, ALUGRAM® Xtra SIL). TLC plates were visualized under 254 nm UV lamp.

Enantiomeric ratios (*e.r.*) were determined by HPLC analysis using a Dionex Summit HPLC system with a CHIRAL ART Amylose-SA (4.6 mm × 250 mm, 5 µm), CHIRAL ART Cellulose-SB (4.6 mm × 250 mm, 5 µm) and CHIRALPAK Amylose AD-H (4.6 mm × 250 mm, 5 µm) chiral stationary phase. Preparative HPLC was carried out using a Thermo Scientific Dionex Ultimate 3000 system with variable wavelength detection and a Grace Alltima Silica 10µm 250x10 mm column.

Dry solvents were taken from an mBRAUN SPS solvent purifier. All reactions were run under an Argon atmosphere unless otherwise stated. All chemicals were purchased from commercial suppliers and used without further purification unless otherwise stated.

## 2 General Information St. Andrews

All reagents and solvents were obtained from commercial suppliers and were used as received without further purification unless otherwise stated. Purification was carried out according to standard laboratory methods. (*S*)-Tetramisole·HCl **ITU-1** was purchased from Sigma-Aldrich, (*R*)-benzotetramisole (BTM) **ITU-4**, (2*S*,3*R*)-HyperBTM **ITU-2**, (4b*S*,12a*R*)-Fused-BTM **ITU-S6**, (*S*)-$^{i}$PrBTM·HCl **ITU-S7** and (2*S*,3*R*)-HyperSe **ISeU-2** were prepared in house [1–4].

**Purification of solvents**

Anhydrous solvents ($Et_2O$, $CH_2Cl_2$, THF and toluene) were obtained after passing through an alumina column (Mbraun SPS-800). Anhydrous MeCN, DMSO and MeOH were purchased from Acros Organics and used without further purification. Petrol corresponds to petroleum ether 40–60 °C and is defined as PE.

**Purification of reagents**

Benzylamine was distilled under vacuum using pre-dried glassware and then stored with 4 Å activated molecular sieves in a Schlenk tube under nitrogen.

**Experimental details**

Reactions involving moisture sensitive reagents were carried out in flame-dried glassware under an inert atmosphere ($N_2$) using standard vacuum line techniques. Room temperature (rt) refers to 20–25 °C. Temperatures of 0 °C and –78 °C were obtained using ice/water and $CO_2$(s)/acetone baths, respectively. Temperatures of 0 °C to –78 °C for overnight reactions were obtained using an immersion cooler (HAAKE EK 90) with acetone as bath medium. Reaction involving heating were performed using DrySyn blocks and a contact thermocouple. *In vacuo* refers to the use of either a Büchi Rotavapor R-200 with a Büchi V-491 heating bath and Büchi V-800 vacuum controller, a Büchi Rotavapor R-210 with a Büchi V-491 heating bath and Büchi V-850 vacuum controller, a Heidolph Laborota 4001 with vacuum controller, an IKA RV10 rotary evaporator with a IKA HB10 heating bath and ILMVAC vacuum controller, or an IKA RV10 rotary evaporator with a IKA HB10 heating bath and Vacuubrand CVC3000 vacuum controller. Rotary evaporator condensers are fitted to Julabo FL601 Recirculating Coolers filled with ethylene glycol and set to –6 °C.

**Purification of products**

Analytical thin layer chromatography was performed on pre-coated aluminium plates (Kieselgel 60 F254 silica) and visualisation was achieved using ultraviolet light (254 nm) and/or staining with aqueous $KMnO_4$ solution followed by heating. Manual column chromatography was performed in glass columns fitted with porosity 3 sintered discs over Geduran® Si 60 (40–60 µm) or Kieselgel 60 (0.040–0.063 mm) silica in the solvent system stated. Automated chromatography was performed on a Biotage Isolera Four running Biotage OS578 with a UV/Vis detector using the method stated and cartridges filled with Kieselgel 60 silica.

**Analysis of products**

**Melting points (mp)** were recorded on an Electrothermal 9100 melting point apparatus, (dec) refers to decomposition.

**Optical rotations** $[\alpha]_D^{20}$ were measured on a Perkin Elmer Precisely/Model-341 polarimeter operating at the sodium D line with a 100 mm path cell at 20 °C.

**HPLC analyses** were obtained on either a Shimadzu HPLC consisting of a DGU-20A5 degassing unit, LC-20AT liquid chromatography pump, SIL-20AHT autosampler, CMB-20A communications bus module, SPD-M20A diode array detector and a CTO-20A column oven or a Shimadzu HPLC consisting of a DGU-20A5R degassing unit, LC-20AD liquid chromatography pump, SIL-20AHT autosampler, SPD-20A UV/Vis detector and a CTO-20A column oven. Separation was achieved using either DAICEL CHIRALCEL OD-H and OJ-H columns or DAICEL CHIRALPAK, IA, IB, IC and ID columns using the method stated. HPLC traces of enantiomerically enriched compounds were compared with authentic racemic spectra prepared beforehand.

**Infrared spectra ($\nu_{max}$)** were recorded on a Shimadzu IRAffinity-1 Fourier transform IR spectrophotometer fitted with a Specac Quest ATR accessory (diamond puck). Spectra were recorded of either thin films or solids, with characteristic absorption wavenumbers ($\nu_{max}$) reported in $cm^{-1}$.

**$^{1}$H, $^{13}$C{$^{1}$H}, and $^{19}$F{$^{1}$H} NMR spectra** were acquired on either a Bruker AV400 with a BBFO probe ($^{1}$H 400 MHz; $^{13}$C{$^{1}$H} 101 MHz; $^{19}$F{$^{1}$H} 377 MHz), a Bruker AVII 400 with a BBFO probe ($^{1}$H 400 MHz; $^{13}$C{$^{1}$H} 101 MHz; $^{19}$F{$^{1}$H} 376 MHz), a Bruker AVIII-HD 500 with a SmartProbe BBFO+ probe ($^{1}$H 500 MHz, $^{13}$C{$^{1}$H} 126 MHz, $^{19}$F{$^{1}$H} 470 MHz), or a Bruker AVIII 500 with a CryoProbe Prodigy BBO probe ($^{1}$H 500 MHz, $^{13}$C{$^{1}$H} 126 MHz, $^{19}$F{$^{1}$H} 470 MHz), in the deuterated solvent stated. All chemical shifts are quoted in parts per million (ppm) relative to the residual solvent peak. All coupling constants, *J*, are quoted in Hz. Multiplicities are indicated as s (singlet), d (doublet), t (triplet), q (quartet), m (multiplet), and multiples thereof. The abbreviation Ar denotes aromatic, and app denotes apparent. NMR peak assignments were confirmed using 2D $^{1}$H correlated spectroscopy (COSY), 2D $^{1}$H nuclear Overhauser effect spectroscopy (NOESY), 2D $^{1}$H−$^{13}$C heteronuclear multiple-bond correlation spectroscopy (HMBC), 2D $^{1}$H−$^{13}$C heteronuclear single quantum coherence (HSQC) and 2D $^{1}$H total correlation spectroscopy (TOCSY) where necessary.

**Mass spectrometry (HRMS)**

data were acquired by either electrospray ionisation (ESI), chemical ionisation (CI), electron impact (EI), atmospheric solids analysis probe (ASAP), atmospheric pressure chemical ionization (APCI) or nanospray ionisation (NSI) by the University of St Andrews Mass Spectrometry Facility or the University of Edinburgh Mass Spectrometry Facility

# 3 Preparation of starting materials

## 3.1 Synthesis of activated glycine Schiff base ester 1

### Preparation of benzophenone imine (6)

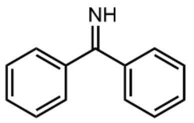

**6**

According to literature, [5] a 250 mL three necked round bottom flask equipped with a dropping funnel was charged with a magnetic stirring bar and magnesium tunings (1.6 g, 66 mmol). The flask was flame dried, after which the magnesium tunings were layered with 10 mL anhydrous $Et_2O$. A solution of bromobenzene (6.8 mL, 64 mmol) in 20 mL $Et_2O$ was put into a dropping funnel and roughly one third was added to the flask. If the Grignard reaction did not start itself at this stage, a crystal of iodine was added. The remaining bromobenzene solution was added at such a rate that a gentle reflux was maintained. After complete addition, the mixture was further refluxed for 30 min. A solution of benzonitrile (4.9 mL, 48 mmol) in 20 mL $Et_2O$ was added to the dropping funnel and added at such a rate that a gentle reflux was maintained (roughly 30 min). As a lot of solid emerged, 10 more mL of anhydrous $Et_2O$ were added, and the mixture was refluxed for further 30 min and then stirred at r.t. overnight. After cooling to r.t., 13 mL MeOH were added slowly via dropping funnel (addition time around 30 min) and the mixture was left to stir for another 2.5 h. The solids were filtered off, washed with some $Et_2O$ and the low boiling components were evaporated on the rotary evaporator. An orange oil was obtained which was further purified by Kugelrohr distillation (150 °C, 10 mbar) to yield benzophenone imine as a yellowish liquid in a yield of 6.1 g (70%). NMR data was in accordance with literature [6].

**$^1$H-NMR** (300 MHz, $CDCl_3$, 298 K): $\delta$ / ppm = 9.73 (s, 1 H, -N**H**), 7.71 (br. s, 2 H, -Ar-**H**), 7.51-7.40 (m, 8 H, Ar-**H**).

### Preparation of N-Boc-Gly-OH (2)

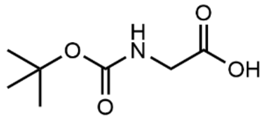

**2**

According to literature [7], NaOH pellets (4.0 g, 100 mmol) were added to 90 mL of water and 60 mL of *t*-BuOH. The mixture was stirred until the pellets were fully dissolved. Then, glycine (6.0 g, 80 mmol) was added portion wise over 10 min. After this, $Boc_2O$ (17.4 g, 80 mmol) was added in 7 equal portions every 5 min. The mixture was stirred overnight and then adjusted to pH = 3 with $KHSO_4$. It was extracted with EtOAc trice, the combined organic layers were dried over $Na_2SO_4$, and the solvents were evaporated. N-Boc-Gly-OH was obtained as a white solid in a yield of 12.8 g (91%) upon drying under high vacuum. NMR data was in accordance with literature [7].

**$^1$H-NMR** (300 MHz, $CDCl_3$, 298 K): $\delta$ / ppm = 10.95 (br. s, 1 H, -COO**H**), 6.77 (br. s, 1 H, -N**H**), 3.95-3.88 (m, 2 H, -C**H$_2$**), 1.43 (s, 9 H, -C**H$_3$**).

**4-Nitrophenyl (tert-butoxycarbonyl)glycinate (4)**

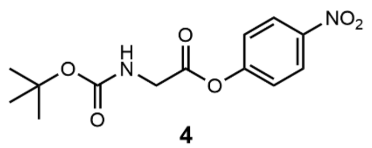

**4**

In analogy to a known procedure [8], N-Boc-Gly-OH (1 eq) and EDC · HCl (1.6 eq) were dissolved in anhydrous DCM (0.5 mol $L^{-1}$) and stirred at r.t. until everything was dissolved. Then, p-$NO_2$ phenol (1 eq) was added, and the mixture was stirred for 20 h at r.t. Water was added, the phases separated, and the aqueous phase extracted with DCM three times. The combined organic layers were dried over $Na_2SO_4$, filtered, and concentrated on the rotary evaporator. The obtained Boc-protected ester was used in the next step without further purification.

**$^1$H-NMR** (300 MHz, $CDCl_3$, 298 K): $\delta$ / ppm = 8.27 (d, $J$ = 9.2 Hz, 2 H, Ar-**H**), 7.31 (d, $J$ = 9.2 Hz, 2 H, Ar-**H**), 5.10 (br. s, 1 H, -N**H**), 4.20 (d, $J$ = 5.8 Hz, 2 H, -C**H₂**), 1.47 (s, 9 H, -C**H₃**).

**4-Nitrophenyl glycinate hydrochloride (5)**

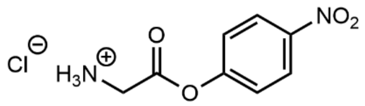

**5**

With slight adaptions to a known procedure [9], a 10-fold excess of hydrogen chloride in dioxane (4 mol $L^{-1}$ solution) was added to the crude product of esterification. The mixture was stirred for 1.5 h at r.t. after which the emerging solid was filtered off and washed with little dioxane. The hydrochloride salt was obtained as a white solid in a yield of 67% after both steps.

**$^1$H-NMR** (300 MHz, MeOD d4, 298 K): $\delta$ / ppm = 8.36 (d, $J$ = 9.2 Hz, 2 H, Ar-**H**), 7.49 (d, $J$ = 9.2 Hz, Ar-**H**), 4.22 (s, 2 H, -C**H₂**).

**$^{13}$C-NMR** (75 MHz, MeOD d4, 298 K): $\delta$ / ppm = 167.0 (1 C, -**C**=O), 155.9 (1 C, **$C_{Ar}$**), 147.4 (1 C, **$C_{Ar}$**), 126.4 (2 C, **$C_{Ar}$**), 123.7 (2 C, **$C_{Ar}$**), 41.4 (1 C, -**C**$H_2$).

**HRMS (ESI-TOF):** m/z calculated for $[M]^+$: 197.0557, found 197.0555.

**Melting point:** 150.4 – 153.6 °C

## 4-Nitrophenyl 2-((diphenylmethylene)amino)acetate (1)

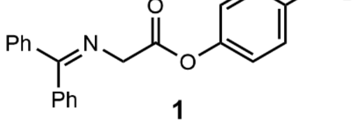

**1**

In analogy to a known procedure [10], hydrochloride salt **5** (1 eq) was dissolved in anhydrous DCM (0.2 mol $L^{-1}$) and benzophenone imine (1 eq) was added in one portion. The mixture was stirred for 16 h at r.t. The suspension was filtered over a pad of $Na_2SO_4$ and flushed with some additional DCM. The filtrate was concentrated on the rotary evaporator to yield an oily residue. This was triturated with some $Et_2O$ (add until the compound precipitated) and put into a freezer at -28 °C for 2 h. The solid was collected by suction filtration, washed with little pre-cooled $Et_2O$, and dried under high vacuum to yield glycine Schiff base ester **1** as an off-white to slightly yellow solid in a yield of 78%.

**$^{1}$H-NMR** (300 MHz, $CDCl_3$, 298 K): *δ* / ppm = 8.27 (d, *J* = 9.2 Hz, 2 H, Ar-**H**), 7.52-7.50 (m, 2 H, Ar-**H**), 7.44-7.34 (m, 3 H, Ar-**H**), 7.31 (d, *J* = 9.2 Hz, 2 H, Ar-**H**), 7.26-7.21 (m, 2 H, Ar-**H**), 4.48 (s, 2 H, -C**H**$_2$).

**$^{13}$C-NMR** (75 MHz, $CDCl_3$, 298 K): *δ* / ppm = 173.2 (1 C, -**C**=O), 168.4 (1 C, -**C**=N), 155.4 (1 C, **C**$_{Ar}$), 145.5 (1 C, **C**$_{Ar}$), 139.0 (1 C, **C**$_{Ar}$), 135.9 (1 C, **C**$_{Ar}$), 131.0 (1 C, **C**$_{Ar}$), 129.3 (1 C, **C**$_{Ar}$), 219.1 (2 C, **C**$_{Ar}$), 129.0 (2 C, **C**$_{Ar}$), 128.4 (2 C, **C**$_{Ar}$), 127.7 (2 C, **C**$_{Ar}$), 125.4 (2 C, **C**$_{Ar}$), 122.5 (2 C, **C**$_{Ar}$), 55.6 (1 C, -**C**H$_2$).

**HRMS (ESI-TOF):** m/z calculated for $[M+H]^+$: 361.1183, found 361.1182.

**Melting point:** 100.6 – 103.6 °C

## 3.2 General procedure A: Claisen condensation with ester

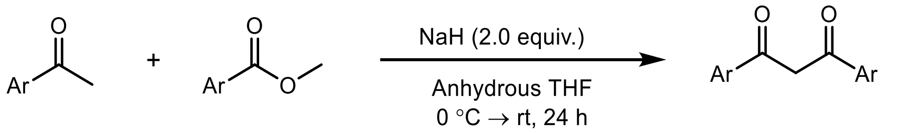


Following a modified procedure by Abe and co-workers [11], NaH 60% suspension in mineral oil (2.0 equiv.) was suspended in anhydrous THF (4.2 M) under $N_2$. The reaction was cooled to 0 °C and stirred for 30 min. The required ester (1.0 equiv.) was dissolved in anhydrous THF (2 M) and added dropwise to the reaction mixture. The required ketone (1.0 equiv.) was dissolved in anhydrous THF (2 M) and added dropwise to the reaction mixture. The reaction was then stirred for 3 h at 0 °C. The reaction was allowed to warm to rt and stirred at rt for 16 h. The reaction mixture was poured over a mixture of ice and aq. HCl 1 M (1:1, 5 vol). The mixture was extracted with EtOAc (x3 vol). The combined organic layers were dried ($MgSO_4$), filtered, and concentrated *in vacuo*. The crude residue was purified using the method stated.

## 3.3 General procedure B: Claisen condensation with acyl chloride

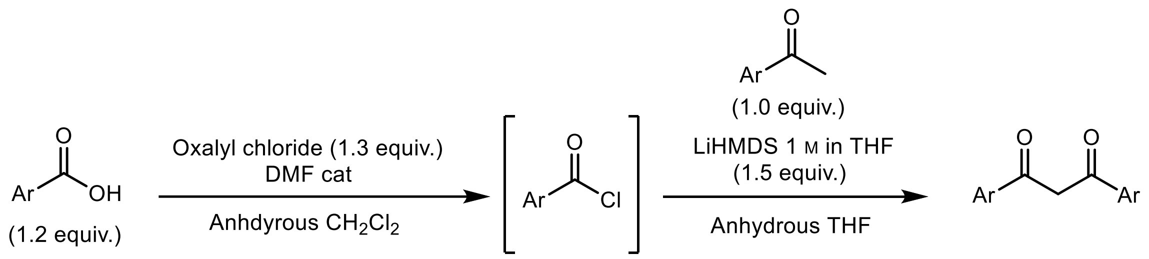


Oxalyl chloride (1.3 equiv.) and DMF (cat) were added to a solution of the required carboxylic acid (1.2 equiv.) in anhydrous $CH_2Cl_2$ (0.3 M) under $N_2$. The reaction was stirred at rt for 1 h and was then concentrated *in vacuo* to yield the crude acyl chloride. Separately, the required ketone (1.0 equiv.) was dissolved in anhydrous THF (0.5 M) and the solution was cooled to –78 °C. LiHMDS 1 M in THF (1.5 equiv.) was added slowly to the solution of ketone. The reaction was then stirred for 1 h at the same temperature. The crude residue of acyl chloride was dissolved in anhydrous THF (1 M) and added dropwise to the reaction mixture. The reaction was warmed to rt and left to stir for 16 h. The reaction

was quenched with a solution of aq. citric acid 10% (×1 vol). It was then extracted with EtOAc (×2 vol) and the combined organic layers were washed with brine (×1 vol), dried ($MgSO_4$), filtered, and concentrated *in vacuo*. The crude residue was purified via column chromatography using the solvent system stated.

## 3.4 General procedure C: synthesis of diketone Michael acceptors

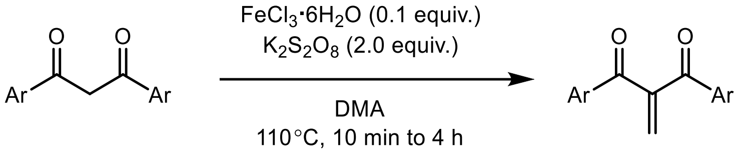


Following a procedure by Li and coworkers [12], the required diketone (1.0 equiv.), potassium persulfate (2.0 equiv.), iron (III) chloride hexahydrate (0.1 equiv.) and DMA (0.25 M), were added to a round bottomed flask. The reaction was stirred at 110 °C and monitored by $^1H$ NMR. After all the diketone was consumed, the resulting mixture was cooled to rt and diluted with $Et_2O$ (1 vol). The mixture was washed with brine (×3 vol), dried ($MgSO_4$), filtered, and concentrated *in vacuo*. The crude residue was purified via column chromatography using the solvent system stated.

## 3.5 General procedure D: Synthesis of aryl p-quinone methides

Aryl quinone methides **7a-7t** were prepared according to an established procedure [13].

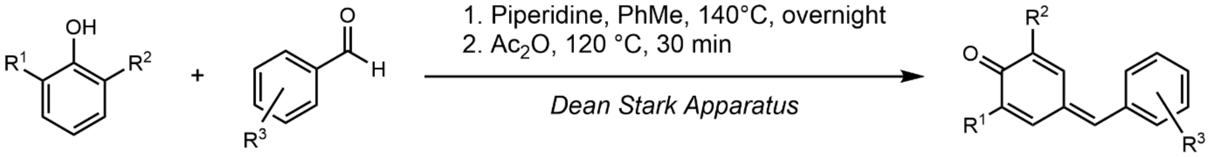


In a *Dean Stark Apparatus*, the respective phenol (1.1 eq.) and benzaldehyde (1 eq.) were dissolved in toluene (0.3 mol $L^{-1}$) and heated to 140 °C. Then, a solution of piperidine in toluene (2 eq piperidine, c = 1.0 mol $L^{-1}$) was added dropwise over 30 min and refluxing was continued overnight. After this, the solution was cooled to 120 °C and $Ac_2O$ (2 eq.) was added dropwise and it was further stirred for 30 min. The solution was poured on ice water, the phases were separated and the aqueous layer was extracted with EtOAc trice. The combined organic layers were washed with brine, dried over $Na_2SO_4$, and filtered. The solvents were evaporated under reduced pressure and the crude products purified by column chromatography on silica using a gradient from heptanes to heptanes/EtOAc 5/1.

## 3.6 General procedure E: Synthesis of MOM protected QMs

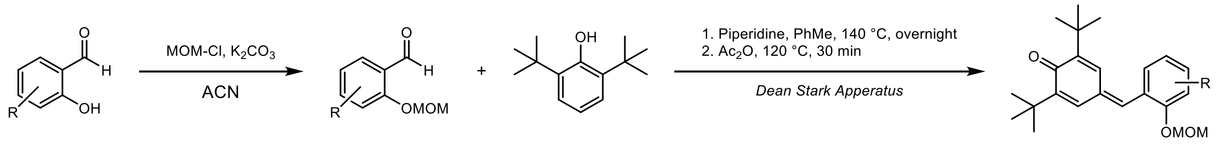


With slight adaptions to literature [13,14], the salicylaldehyde derivative (10 mmol, 1 eq) and $K_2CO_3$ (20 mmol, 2 eq) were suspended in ACN (0.25 mol $L^{-1}$). MOM-Cl (12 mmol, 1.2 eq) was added in one portion and the mixture was stirred at rt overnight. The mixture was diluted with 20 mL DCM and 20 mL 0.5 M NaOH were added. The mixture was stirred for further 30 min to make sure that any excess MOM-Cl is destroyed. After this, the phases were separated and the aqueous layer was extracted with DCM trice. The combined organic phases were dried over $Na_2SO_4$, filtered, and concentrated. The crude products were purified by column chromatography on silica (heptanes/EtOAc 10/1) to give the MOM protected salicylaldehydes.

The protected salicylaldehyde derivatives (1 eq) and 2,6-di(*tert*-butyl)phenol (1.1 eq) were dissolved in toluene (0.3 mol $L^{-1}$) in a *Dean Stark apperatus* and heated to 140 °C. Then, a solution of piperidine in toluene (2 eq piperidine, c = 1.0 mol $L^{-1}$) was added dropwise over 30 min and refluxing was continued overnight. After this, the solution was cooled to 120 °C and $Ac_2O$ (2 eq.) was added dropwise and it was further stirred for 30 min. The solution was poured on ice water, the phases were separated and the aqueous layer was extracted with EtOAc trice. The combined organic layers were washed with brine, dried over $Na_2SO_4$, and filtered. The solvents were evaporated under reduced pressure and the crude products purified by column chromatography on silica using a gradient from heptanes to heptanes/EtOAc 5/1.

### 3.7 Synthesis of diketones

**1,3-Bis(4-methoxyphenyl)propane-1,3-dione (S25)**

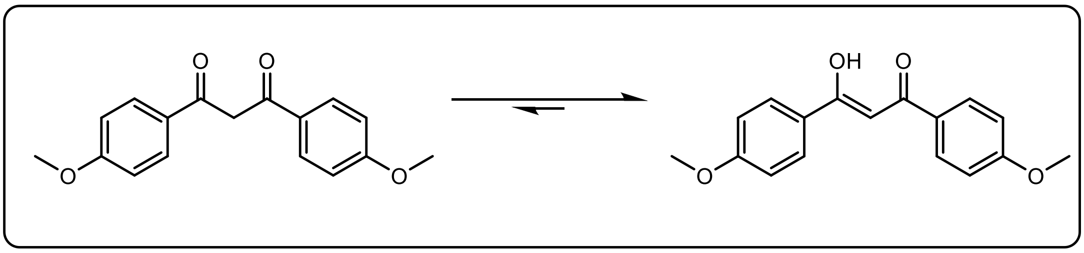


Following a modified procedure by Zou and co-workers [15], NaH 60% suspension in mineral oil (905 mg, 24 mmol, 2.0 equiv.) was dissolved in anhydrous THF (2.2 mL, 11 M) in a two-necked round bottomed flask under $N_2$. The reaction was cooled to 0 °C and a solution of 4-methylbenzoate (2.0 g, 12 mmol, 1.0 equiv.) in anhydrous THF (2.2 mL, 5 M) and added dropwise to the reaction mixture. A solution of 4'-methoxyacetophenone (2.7 g, 18 mmol, 1.5 equiv.) in anhydrous THF (2.2 mL, 8 M) was then added dropwise to the reaction mixture. The reaction was warmed to rt and stirred for 1 h at rt. It was then heated at 30 °C and stirred for 1 h. The reaction mixture was poured over a mixture of ice/water (1/1, 50 mL) and phosphoric acid (1.2 mL). The precipitate was filtered and washed with cold water (50 mL). The solid was recrystallised in acetone/ethanol to yield the title compound as the enol (2.6 g, 76%) as a yellow solid with data in accordance with the literature [16]

**$^1$H-NMR** (400 MHz, $CDCl_3$) $\delta_H$: 3.88 (6H, s, OC**H**$_3$ ×2), 6.73 (1H, s, C=C**H**), 6.98 (4H, d, *J* 9.6, C**H** Ar), 7.96 (4H, d, *J* 8.3, C**H** Ar ×4)

**Melting point:** 104-106 °C (acetone/ethanol) {Lit [16]} 118-120 °C (PE/EtOAc)

**1,3-Bis(4-(dimethylamino)phenyl)propane-1,3-dione (S26)**

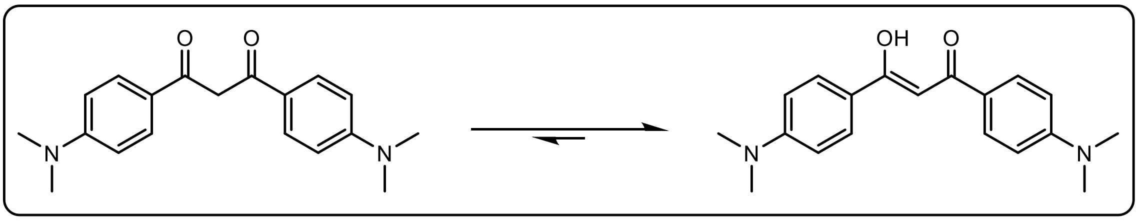


Following general procedure B, oxalyl chloride (1.3 mL, 16.0 mmol), DMF (drop), 4-(dimethylamino)benzoic acid (2.4 g, 14.8 mmol) in anhydrous $CH_2Cl_2$ (50 mL) at rt for 1 h gave the crude acyl chloride. 1-(4-(Dimethylamino)phenyl)ethan-1-one (2 g, 12.3 mmol) in anhydrous THF (25 mL), LiHMDS 1 M in THF (18.5 mL, 18.5 mmol) and the acyl chloride in anhydrous THF (15 mL) at rt for 16 h, gave an impure solid. The crude material was suspended in $Et_2O$ and the precipitated was filtered and washed with further $Et_2O$ to remove any remaining starting material. The solid was then purified via column chromatography (30:70, Hexane:$Et_2O$, $R_f$ = 0.5) to yield the title compound as a yellow solid (1.6 g, 42%).

*Note*: By $^1$H/$^{13}$C NMR in $CDCl_3$, the product was present as a mixture of tautomers (5:1, enol:diketone).

**$^1$H-NMR** (400 MHz, $CDCl_3$) $\delta_H$: 3.09 (12H, s, N(C**H**$_3$)$_2$ ×2), 6.70 (1H, s, C=C**H**), 6.73 (4H, d, *J* 9.2, C**H** Ar ×4), 7.92 (4H, d, *J* 8.3, C**H** Ar ×4)

**$^{13}$C-NMR** (126 MHz, $CDCl_3$) $\delta_C$: 40.1 (N(**C**H$_3$)$_2$ ×2), 90.0 (C=**C**H), 111.2 (**C**H Ar ×4), 123.4 (**C** Ar ×2), 128.8 (**C**H Ar ×4), 152.9 (**C** Ar ×2), 184.0 (**C**=O)

**HRMS** (ESI$^+$) $C_{19}H_{22}N_2O_2$ [M+H]$^+$ found 311.1751 requires 311.1754 (+1.0 ppm)

**Melting point:** 177-182 °C ($Et_2O$)

**1,3-Bis(3,4-dimethoxyphenyl)propane-1,3-dione (S27)**

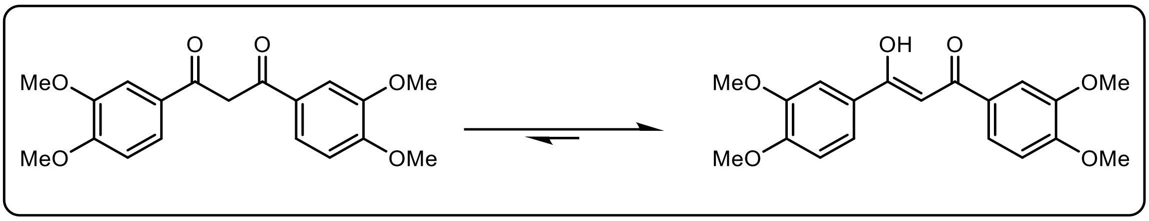


Following general procedure A, NaH 60% suspension in mineral oil (538 mg, 22.4 mmol) in anhydrous THF (6 mL), methyl 3,4-dimethoxybenzoate (2 g, 10.2 mmol) in anhydrous THF (6 mL) and 1-(3,4-dimethoxyphenyl)ethan-1-one (1.8 g, 10.2 mmol) in anhydrous THF (6 mL) at rt for 16 h gave an oily residue. $Et_2O$ was added and the solid that precipitated was filtered and washed with more $Et_2O$ to yield the title compound as a yellow solid (1.3 g, 34%).

*Note*: By $^1$H/$^{13}$C NMR in $CDCl_3$, the product was present as a mixture of tautomers (5:1, enol:diketone).

**$^1$H-NMR** (400 MHz, $CDCl_3$) $\delta_H$: 3.98 (6H, s, C(4)OC**H**$_3$ ×2), 4.0 (6H, s, C(3)OC**H**$_3$ ×2), 6.76 (1H, s, C=C**H**), 6.96 (2H, d, *J* 8.5, C**H** Ar ×2), 7.57 (2H, d, *J* 2.0, C**H** Ar ×2), 7.62 (2H, dd, *J* 8.5, 1.8, C**H** Ar ×2)

**$^{13}$C-NMR** (126 MHz, $CDCl_3$) $\delta_C$: 56.1 (O**C**H$_3$ ×4), 91.8 (C=**C**H), 109.8 (**C**H Ar ×2), 110.6 (**C**H Ar ×2), 121.0 (**C**H Ar ×2), 128.5 (**C**=CH), 149.2 (**C**OCH$_3$ ×2), 152.8 (**C**OCH$_3$ ×2), 184.6 (**C**=O)

**HRMS** (ESI$^+$) $C_{19}H_{20}O_6$ [M+H]$^+$ found 345.1330 requires 345.1333 (–0.9 ppm)

**Melting point:** 118-121 °C ($Et_2O$)

**1,3-Bis(4-bromophenyl)propane-1,3-dione (S28)**

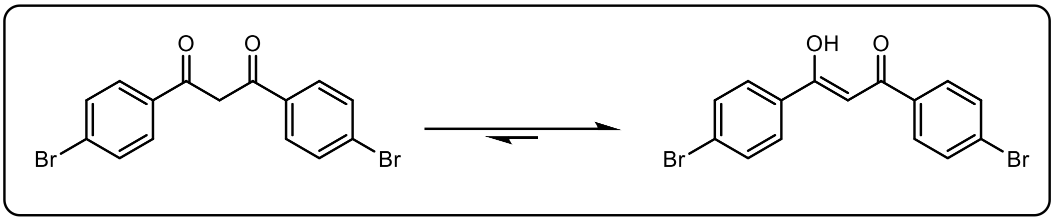


Following general procedure A, NaH 60% suspension in mineral oil (492 mg, 20.5 mmol) in anhydrous THF (5 mL), methyl 4-bromobenzoate (2.0 g, 9.3 mmol) in anhydrous THF (5 mL), 4′-bromoacetophenone (1.9 g, 9.3 mmol) in anhydrous THF (5 mL) at rt for 16 h, gave the title compound as an orange solid and as the enol (2.9 g, 81%) used without any further purification and with data in accordance with the literature [17].

**$^1$H-NMR** (400 MHz, $CDCl_3$) $\delta_H$: 6.79 (1H, s, C=C**H**), 7.66 (4H, d, *J* 9.4, C**H** Ar x4), 7.87 (4H, d, *J* 8.6, C**H** Ar x4).

**Melting point:** 164-168 °C (EtOAc) {Lit [17] 198-200 °C (EtOH)};

**1,3-Bis(4-chlorophenyl)propane-1,3-dione (S29)**

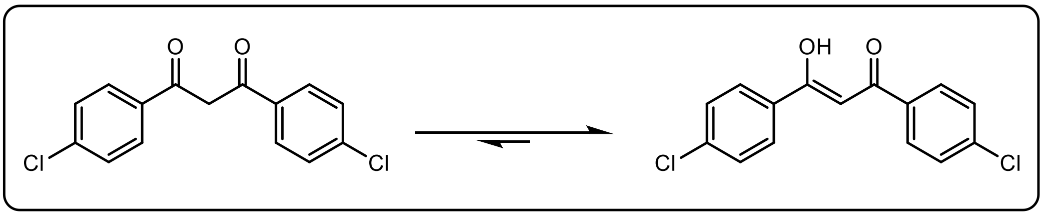


Following general procedure A, NaH 60% suspension in mineral oil (562 mg, 23.4 mmol) in anhydrous THF (6.5 mL), methyl 4-chlorobenzoate (2 g, 11.7 mmol) in anhydrous THF (6.5 mL), 4′-chloroacetophenone (1.8 g, 11.7 mmol) in anhydrous THF (6.5 mL) at rt for 16 h gave after purification via column chromatography (30:70, EtOAc:Hexane, $R_f$ = 0.7) the title compound as a yellow solid and as the enol (2.4 g, 71%) with data in accordance with the literature [17].

**$^1$H-NMR NMR** (400 MHz, $CDCl_3$) $\delta_H$: 6.79 (1H, s, C=C**H**), 7.49 (4H, d, *J* 9.2, C**H** Ar ×4), 7.94 (4H, d, *J* 9.2, C**H** Ar ×4).

**Melting point:** 128-129°C (EtOAc/Hexane) {Lit [17]130-131°C (EtOH)};

**1,3-Di(naphthalen-2-yl)propane-1,3-dione (S30)**

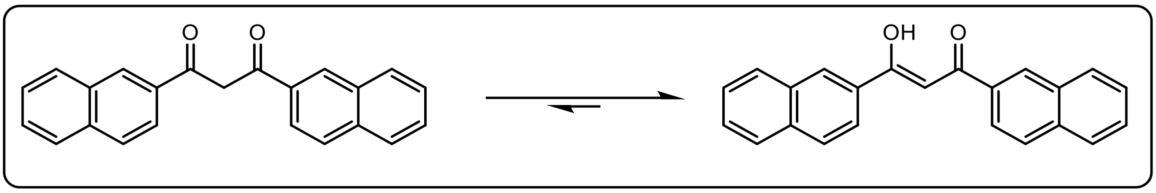


Following general procedure A, NaH 60% suspension in mineral oil (807 mg, 21.4 mmol) in anhydrous THF (5 mL), methyl 2-naphthoate (2 g, 10.7 mmol) in anhydrous THF (5 mL) and 1-(naphthalen-2-yl)ethan-1-one (1.8 g, 10.7 mmol) in anhydrous THF (5 mL) at rt for 16 h, gave a solid that was washed with $Et_2O$ and filtered to give the title compound as the enol (1.3 g, 37%) as a yellow solid with data in accordance with the literature [18].

**$^1$H-NMR** (400 MHz, $CDCl_3$) $\delta_H$: 7.15 (1H, s, C=C**H**), 7.55-7.63 (4H, m, C**H** Ar ×4), 7.91 (2H, d, *J* 8.0, C**H** Ar ×2), 7.96 (2H, d, *J* 8.5, C**H** Ar ×2), 8.01 (2H, d, *J* 8.0, C**H** Ar ×2), 8.08 (2H, dd, *J* 8.5, 1.9, C**H** Ar ×2), 8.59 (2H, s, C**H** Ar ×2)

**$^{13}$C-NMR** (126 MHz, $CDCl_3$) $\delta_C$: 93.8 (C=**C**H), 123.3 (**C**H Ar ×2), 126.8 (**C**H Ar ×2), 127.8 (**C**H Ar ×2), 128.2 (**C**H Ar ×2), 128.4 (**C**H Ar ×2), 128.5 (**C**H Ar ×2), 129.4 (**C**H Ar ×2), 132.8 (**C** Ar ×2), 132.9 (**C** Ar ×2), 135.4 (**C** Ar ×2), 185.6 (**C**=O and HO**C**=CH)

**HRMS** ($ESI^+$) $C_{23}H_{16}O_2$ $[M+H]^+$ found 325.1223 requires 325.1223 (±0.0 ppm), $[M+Na]^+$ found 347.1033 requires 347.1048 (–2.7 ppm)

**Melting point:** 155-158 °C ($Et_2O$) {Lit [19] 171-172 °C}

**1,3-Di(naphthalen-1-yl)propane-1,3-dione (S31)**

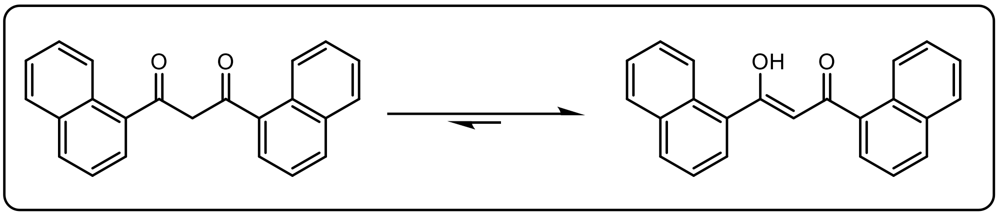


Following general procedure B, oxalyl chloride (1 mL, 12.6 mmol), DMF (cat), 1-naphtoic acid (2 g, 11.6 mmol) in anhydrous $CH_2Cl_2$ (39 mL) at rt for 1 h gave the crude acyl chloride. 1-(Naphthalen-1-yl)ethan-1-one (1.7 g, 9.7 mmol) and LiHMDS 1 M in THF (14.6 mL, 14.6 mmol) in anhydrous THF (19 mL), and the acyl chloride in anhydrous THF (12 mL) at rt for 16 h, gave after purification by column chromatography (30:70, hexane:$Et_2O$, $R_f$ = 0.5), the product mixed with some starting material. The crude residue was suspended in $Et_2O$, the solid was filtered and washed with $Et_2O$ to yield the title compound as a yellow solid and as the enol (988 mg, 32%) with data in accordance with the literature [20].

$^1$**H-NMR** (400 MHz, $CDCl_3$) $\delta_H$: 6.63 (1H, s, C=C**H**), 7.53-7.61 (4H, m, C**H** Ar ×4), 7.62-7.67 (2H, m, C**H** Ar ×2), 7.87 (2H, dd, *J* 7.4, 1.2, C**H** Ar ×2), 7.94 (2H, d, *J* 8.0, C**H** Ar ×2), 8.02 (2H, d, *J* 8.6, C**H** Ar ×2), 8.65 (2H, d, *J* 8.2, C**H** Ar ×2)

$^{13}$**C-NMR** (126 MHz, $CDCl_3$) $\delta_C$: 103.1 (C=**C**H), 124.8 (**C**H Ar ×2), 125.7 (**C**H Ar ×2), 126.5 (**C**H Ar ×2), 127.3 (**C**H Ar ×2), 127.4 (**C**H Ar ×2), 128.6 (**C**H Ar ×2), 130.2 (**C** Ar ×2), 131.9 (**C**H Ar ×2), 133.9 (**C** Ar ×2), 134.5 (**C** Ar ×2), 189.2 (**C**=O)

**HRMS** (ESI$^+$) $C_{23}H_{16}O_2$ $[M+H]^+$ found 325.1226 requires 325.1223 (+0.9 ppm)

**Melting point:** 100-105 °C ($Et_2O$)

**1,3-Di(thiophen-2-yl)propane-1,3-dione (S32)**

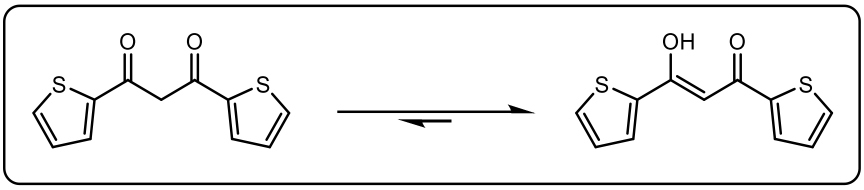

Following general procedure A, NaH 60% suspension in mineral oil (677 mg, 28.2 mmol) in anhydrous THF (8 mL), methyl thiophene-2-carboxylate (1.6 mL, 14.1 mmol) in anhydrous THF (8 mL) and 1-(thiophen-2-yl)ethan-1-one (1.5 mL, 14.1 mmol) in anhydrous THF (8 mL) a at rt for 16 h gave after purification by column chromatography (30:70, $Et_2O$:hexane, $R_f$ = 0.4) the title compound as a yellow solid (2.3 g, 69%) with data in accordance with the literature [21].

Note: By $^1$H/$^{13}$C NMR in $CDCl_3$, the product was present as a mixture of tautomers (5:1, enol:diketone).

$^1$**H-NMR** (400 MHz, $CDCl_3$) $\delta_H$: 6.56 (1H, s, C=C**H**), 7.19 (2H, dd, *J* 5.0, 4.1, C**H** Ar ×2), 7.63 (2H, dd, *J* 4.8, 1.0, C**H** Ar ×2), 7.80 (2H, dd, *J* 3.9, 1.2, C**H** Ar ×2);

$^{13}$**C-NMR** (126 MHz, $CDCl_3$) $\delta_C$: 92.7 (C=**C**H), 128.3 (**C**H Ar ×2), 130.0 (**C**H Ar ×2), 132.0 (**C**H Ar ×2), 140.6 (**C** Ar ×2), 178.8 (**C**=O)

**HRMS** (ESI$^+$) $C_{11}H_8O_2S_2$ $[M+Na]^+$ found 258.9855 requires 258.9863 (–3.1 ppm)

**Melting point:** 80-84 °C ($Et_2O$/hexane) {Lit [22] 98 °C ($Et_2O$)}

**1,3-Di(thiophen-3-yl)propane-1,3-dione (S33)**

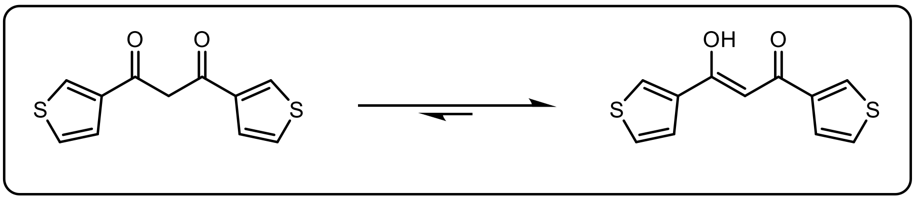

Following general procedure A, NaH 60% suspension in mineral oil (560 mg, 23.3 mmol) in anhydrous THF (6 mL), methyl thiophene-3-carboxylate (1.3 g, 10.6 mmol) in anhydrous THF (6 mL) and 1-(thiophen-3-yl)ethan-1-one (1.5 g, 10.6 mmol) in anhydrous THF (6 mL) at rt for 16 h, gave after purification by column chromatography (30:70, $Et_2O$:PE, $R_f$ = 0.5), the title compound as a yellow solid and as the enol (1.3 g, 52%).

**$^1$H-NMR** (400 MHz, $CDCl_3$) $\delta_H$: 6.51 (1H, s, C=C**H**), 7.41 (2H, dd, *J* 4.8, 3.0, C**H** Ar ×2), 7.56 (2H, dd, *J* 5.1, 1.3, C**H** Ar ×2), 8.11 (2H, dd, *J* 3.0, 1.3, C**H** Ar ×2)

**$^{13}$C-NMR** (126 MHz, $CDCl_3$) $\delta_C$: 94.4 (C=**C**H), 125.9 (**C**H Ar ×2), 126.6 (**C**H Ar ×2), 129.7 (**C**H Ar ×2), 139.3 (**C** Ar), 180.5 (**C**=O)

**HRMS** ($ESI^+$) $C_{11}H_8O_2S_2$ $[M+H]^+$ found 237.0036 requires 237.0038 (–0.8 ppm)

**Melting point:** 101-103 °C ($Et_2O$/PE)

**1,3-Di(furan-2-yl)propane-1,3-dione (S34)**

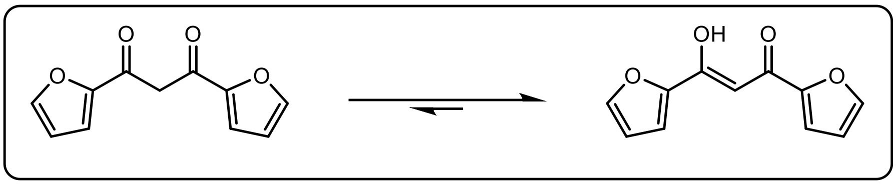


Following general procedure B, 2-furyl methyl-ketone (1.1 mL, 11.2 mmol) in anhydrous THF (22 mL), LiHMDS 1 M in THF (16.8 mL, 16.8 mmol) and 2-furoyl chloride (1.3 mL, 13.4 mmol) in anhydrous THF (13 mL) at rt for 16 h, gave after purification by column chromatography (30:70, $Et_2O$:Hexane, $R_f$ = 0.4), the title compound (1.3 g, 57%) as a light-yellow solid with data in accordance with the literature.[1]

<u>*Note*</u>: By $^1$H/$^{13}$C NMR in $CDCl_3$, the product was present as a mixture of tautomers (8:1, enol:diketone).

**$^1$H-NMR** (500 MHz, $CD_2Cl_2$) $\delta_H$: 6.60 (2H, dd, *J* 1.7, *J* 3.5, C**H** Ar ×2), 6.67 (1H, s, C=C**H**), 7.22 (2H, dd, *J* 0.9, *J* 3.6, C**H** Ar ×2), 7.63 (2H, dd, *J* 0.8, *J* 1.7, C**H** Ar ×2)

**$^{13}$C-NMR** (126 MHz, $CDCl_3$) $\delta_C$: 92.1 (C=**C**H), 112.6 (**C**H Ar ×2), 115.5 (**C**H Ar ×2), 146.1 (**C**H Ar ×2), 150.3 (**C** Ar ×2), 174.7 (**C**=O)

**HRMS** (ESI+) $C_{11}H_8O_4$ $[M+H]^+$ found 205.0496 requires 205.0495 (+0.8 ppm), $[M+Na]^+$ found 227.0314 requires 227.0320 (–2.6 ppm)

**Melting point:** 64-65 °C ($Et_2O$/hexane)

**1,3-Bis(benzo[d][1,3]dioxol-5-yl)propane-1,3-dione (S35)**

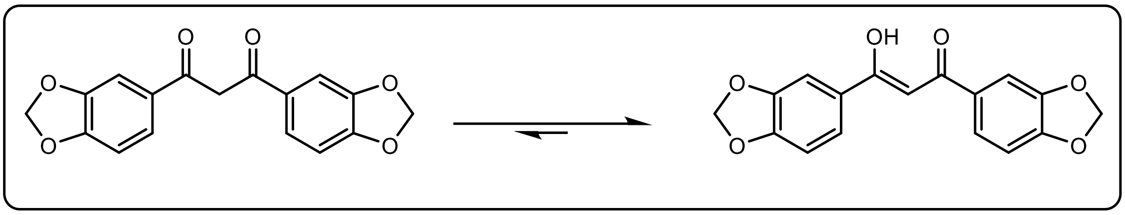

Following general procedure B, oxalyl chloride (1.1 mL, 13 mmol), DMF (cat), piperonylic acid (2 g, 12 mmol), anhydrous $CH_2Cl_2$ (40 mL) at rt for 1 h gave the crude acyl chloride. 1-(benzo[d][1,3]dioxol-5-yl)ethan-1-one (1.6 g, 10 mmol) and LiHMDS 1 M in THF (15 mL, 15 mmol) in anhydrous THF (20 mL) and the crude acyl chloride in anhydrous THF (12 mL) at rt for 16 h, gave after purification by column chromatography (98:2, $CH_2Cl_2$:$Et_2O$, $R_f$ = 0.7), the title compound as a yellow solid and as the enol (853 mg, 28%).

**$^1$H-NMR** (400 MHz, $CDCl_3$) $\delta_H$: 6.08 (4H, s, OC**H**$_2$O ×2), 6.65 (C=C**H**), 6.91 (2H, d, *J* 8.4, C**H** Ar ×2), 7.46 (2H, d, *J* 1.9, C**H** Ar ×2), 7.59 (2H, dd, *J* 8.1, 1.6, C**H** Ar ×2)

**$^{13}$C-NMR NMR** (126 MHz, $CDCl_3$) $\delta_C$: 91.9 (C=**C**H), 101.8 (O**C**H$_2$O), 107.2 (**C**H Ar ×2), 108.2 (**C**H Ar ×2), 122.7 (**C**H Ar ×2), 130.0 (**C** Ar ×2), 148.2 (**C** Ar ×2), 151.3 (**C** Ar ×2), 184.3 (**C**=O)

**HRMS** (ESI$^+$) $C_{17}H_{12}O_6$ $[M+H]^+$ found 313.0703 requires 313.0707 (–1.3 ppm)

**Melting point:** 168-174 °C ($CH_2Cl_2$/$Et_2O$)

## 3.8 Synthesis of Michael acceptors

**Bis(phenylsulfonyl)methane (S36)**

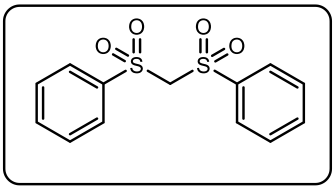

Following a procedure by Sulzer-Mosse and co-workers [23], 35% $H_2O_2$ solution in water (5.5 mL, 64.5 mmol, 5.0 equiv.), was added to a solution of bis(phenylthiol)methane (3.0 g, 12.9 mmol, 1.0 equiv.) in acetic acid (43 mL, 0.3 M) and acetic anhydride (12 mL, 1.1 M) at 0 °C. The reaction was stirred for 24 h at rt (if starting material or partially oxidised product was observed after 24 h more peroxide can be added). Water (90 mL) was added slowly to the reaction mixture. The precipitate was filtered and washed with water (80 mL). The solid was then recrystallised in toluene to yield the title compound (2.7 g, 70%) as a white solid with data in accordance with the literature [24].

**$^1$H-NMR** (400 MHz, $CDCl_3$) $\delta_H$: 4.75 (2H, s, C**H**$_2$($SO_2$)$_2$), 7.60-7.66 (4H, m, C**H** Ph ×4), 7.72-7.78 (2H, m, C**H** Ph ×2), 7.97-8.02 (4H, m, C**H** Ph ×4).

**Melting point:** 119-120 °C (toluene) {Lit [24] 121-122 °C (Hexane/EtOAc)}

**(Ethene-1,1-diyldisulfonyl)dibenzene (16)**

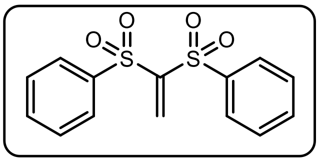

Following a procedure by Alexakis and co-workers [25], bis(phenylsulfonyl)methane **S36** (2.0 g, 6.8 mmol, 1.0 equiv.), was added slowly to a solution of formaldehyde 40% in water (1.5 mL, 20.0 mmol, 3.0 equiv.) and piperidine (3.4 mL, 34 mmol, 5.0 equiv.) in MeOH (22 mL, 0.3 M) at 0 °C. After 70 minutes at 0 °C, a mixture of ice and water (48 mL) was added, and it was stirred for 10 min. The white precipitate was filtered and washed with additional water (50 mL). The white solid was redissolved in $CH_2Cl_2$ (22 mL, 0.3 M) and aq. HCl 1 M in water (34 mL, 34 mol, 5.0 equiv.) was added. The biphasic mixture was stirred vigorously for 3 h at rt. The layers were separated, and the aq. layer was extracted with $CH_2Cl_2$ (40 mL ×2). The combined organic layers were dried ($MgSO_4$), filtered and concentrated *in vacuo*. The crude material was recrystallised from $CH_2Cl_2$/hexane to yield the title compound (1.31 g, 62%) as a white solid with data in accordance with the literature [26].

**$^1$H-NMR** (400 MHz, $CDCl_3$) $\delta_H$: 7.22 (2H, s, C**H**$_2$=C($SO_2$Ph)$_2$), 7.56 (4H, t, *J* 7.7, C**H** Ph ×4 7.68 (2H, app t, *J* 7.4, C**H** Ph ×2), 7.97 (4H, d, *J* 8.3, C**H** Ph ×4)

**Melting point:** 100-102 °C (hexane/$CH_2Cl_2$) {Lit [24] 126-127 °C (ligroin/benzene)}

**2-Methylene-1,3-diphenylpropane-1,3-dione (S37)**

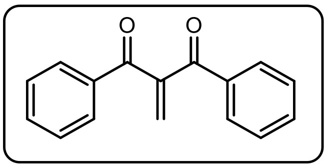

Following general procedure C, 1,3-diphenyl-1,3-propanedione (500 mg, 2.2 mmol), potassium persulfate (1.2 g, 4.4 mmol), iron (III) chloride hexahydrate (60 mg, 0.2 mmol) and DMA (9 mL) at 110 °C for 4 h gave after purification by column chromatography (30:70, EtOAc:Hexane, $R_f$ = 0.5) the title compound as a light-yellow oil (232 mg, 45%).

**$^1$H-NMR** (500 MHz, $CDCl_3$) $\delta_H$: 6.36 (2H, s, C=C**H**$_2$), 7.48 (4H, app t, *J* 8.3, C**H** Ph ×4), 7.57-7.61 (2H, m, C**H** Ph ×2), 7.89-7.92 (C**H** Ph ×4)

**$^{13}$C-NMR NMR** (126 MHz, $CDCl_3$) $\delta_C$: 128.7 (**C**H Ph ×4), 129.6 (**C**H Ph ×4), 131.4 (C=**C**H$_2$), 133.5 (**C**H Ph ×2), 136.2 (**C**(1) Ph ×2), 147.9 (**C**=CH$_2$), 194.2 (**C**=O ×2)

**HRMS** (ESI+) $C_{16}H_{12}O_2$ $[M+H]^+$ found 237.0908 requires 237.0910 (–0.8 ppm), $[M+Na]^+$ found 259.0727 requires 259.0735 (–3.1 ppm)

**IR $\nu_{max}$** (film): 3063 (C-H alkene), 1674 (C=O ketone), 1651 (C=O ketone), 1597 (C=C alkene conjugated), 1485, 1329, 1283, 1163, 980

**Diisopropylammonium 2,2,2-trifluoroacetate (S38)**

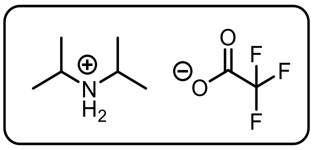

Following a procedure by Connell and co-workers [27], TFA (7.5 mL, 100 mmol, 1.0 equiv.) was added dropwise to a solution of diisopropylamine (14 mL, 100 mmol, 1.0 equiv.) in $Et_2O$ (100 mL, 1.0 M) at 0 °C. The reaction mixture was stirred for 5 min at 0 °C and the solid was filtered and washed with $Et_2O$ to yield the title compound (17.5 g, 81%) with data in accordance with the literature [27].

**$^1$H-NMR** 1.21 (12H, d, J 6.6, CH(C**H**$_3$)$_2$ ×2), 3.35 (2H, m, C**H**(CH$_3$)$_2$ ×2), 8.36 (2H, br s, N**H**$_2$)

**Melting point:** 108-110 °C ($Et_2O$) {Lit [28] 122-123 °C ($Et_2O$)}

**Di-tert-butyl 2-methylenemalonate (S39)**

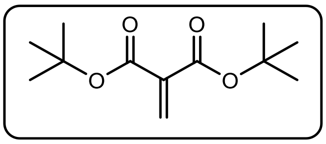

Following a procedure by Connell and co-workers [27], TFA (70 µL, 0.9 mmol, 0.1 equiv.) and diisopropylammonium 2,2,2-trifluoroacetate **S38** (2 g, 9.2 mmol, 1.0 equiv.) were added to a mixture of di-tert-butyl malonate (2.0 g, 9.2 mmol, 1.0 equiv.), paraformaldehyde (550 mg, 18.4 mmol, 2.0 equiv.) and 4 Å activated molecular sieves in anhydrous THF (9.2 mL, 1 M). The reaction mixture was stirred under $N_2$ at reflux for 2 h. The mixture was cooled down to rt and more paraformaldehyde (550 mg, 18.4 mmol, 2.0 equiv.) was added. The reaction was stirred at 80 °C for 16 h under $N_2$. The reaction was then cooled to rt and the solvent removed *in vacuo*. The crude residue was then dissolved in $Et_2O$ (20 mL) and washed with aq. HCl 1 M in water (30 mL ×1), aq. NaOH 1 M (30 mL ×1) and brine (30 mL ×1). The organic layer was dried ($MgSO_4$), filtered, and concentrated *in vacuo*. The crude residue was purified by column chromatography (10:90, EtOAc:hexane, $R_f$ = 0.5) to yield the title compound (706 mg, 34%) as a colourless liquid with data in accordance with the literature [29].

**$^1$H-NMR** (400 MHz, $CDCl_3$) $\delta_H$: 1.50 (18H, s, C(C**H**$_3$)$_3$ ×2), 6.24 (2H, s, C=C**H**$_2$)

**$^{13}$C-NMR NMR** (126 MHz, $CDCl_3$) $\delta_C$: 28.0 (C(**C**$H_3$)$_3$ ×2), 81.9 (**C**($CH_3$)$_3$ ×2), 130.8 (C=**C**$H_2$), 138.4 (**C**=$CH_2$), 163.8 (**C**=O ×2)

**HRMS** (ESI+) $C_{12}H_{20}O_4$ $[M+Na]^+$ found 251.1248 requires 251.1259 (–4.4 ppm)

**IR $\nu_{max}$** (liquid) 2978 (C-H alkene), 1717 (C=O ester), 1368, 1250, 1123, 851

**1,3-Bis(4-methoxyphenyl)-2-methylenepropane-1,3-dione (S40)**

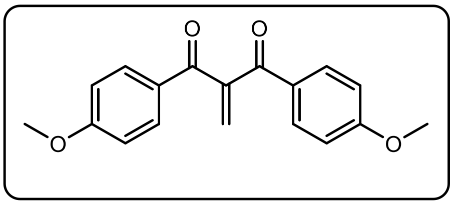

Following general procedure C, 1,3-bis(4-methoxyphenyl)propane-1,3-dione **S25** (500 mg, 1.8 mmol), potassium persulfate (973 mg, 3.6 mmol), iron (III) chloride hexahydrate (48.6 mg, 0.18 mmol) and DMA (7 mL) at 110 °C for 4 h gave after purification by column chromatography (50:50, EtOAc:Hexane, $R_f$ = 0.5) the title compound (296 mg, 56%) as a light yellow solid.

**$^1$H-NMR** (500 MHz, $CDCl_3$) $\delta_H$: 3.85 (6H, s, OC**H**$_3$ ×2), 6.22 (2H, s, C=C**H**$_2$), 6.92 (4H, d, *J* 9.1, C**H** Ar ×4), 7.89 (4H, d, *J* 7.9, C**H** Ar ×4)

**$^{13}$C-NMR** (126 MHz, $CDCl_3$) $\delta_C$: 55.5 (O**C**$H_3$ ×2), 113.9 (**C**H Ar ×4), 129.2 (**C** Ar ×2), 129.4 (C=**C**$H_2$), 132.1 (**C**H Ar ×4), 148.7 (**C**=$CH_2$), 163.9 (**C** Ar ×2), 192.8 (**C**=O ×2)

**HRMS** (ESI$^+$) $C_{18}H_{16}O_4$ $[M+H]^+$ found 297.1114 requires 297.1121 (–2.4 ppm)

**Melting point:** 82-85 °C (EtOAc/hexane)

**1,3-Bis(4-(dimethylamino)phenyl)-2-methylenepropane-1,3-dione (S41)**

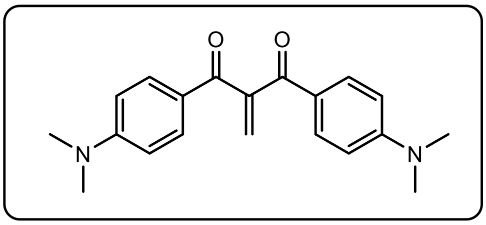

Following general procedure C, 1,3-bis(4-(dimethylamino)phenyl)propane-1,3-dione **S26** (550 mg, 1.8 mmol), potassium persulfate (973 mg, 3.0 mmol), iron (III) chloride hexahydrate (48.7 mg, 0.15 mmol) and DMA (7 mL) at 110 °C for 30 min, gave after purification by column chromatography (30:70, $Et_2O$:$CH_2Cl_2$, $R_f$ = 0.7), the title compound (70 mg, 14%) as a yellow solid.

**$^1$H-NMR** (400 MHz, $CDCl_3$) $\delta_H$: 3.07 (12H, s, N(C**H**$_3$)$_2$ ×2), 6.12 (2H, s, C=C**H**$_2$), 6.65 (4H, d, *J* 9.6, C**H** Ar ×4), 7.88 (4H, d, *J* 8.8, C**H** Ar ×4)

**$^{13}$C-NMR** (126 MHz, $CDCl_3$) $\delta_C$: 40.1 (N(**C**$H_3$)$_2$ ×2), 110.8 (**C**H Ar ×4), 124.0 (**C** Ar ×2), 127.2 (C=**C**$H_2$), 132.2 (**C**H Ar ×4), 150.1 (**C**=$CH_2$), 153.6 (**C** Ar ×2), 192.4 (**C**=O ×2)

**HRMS** (ESI$^+$) $C_{20}H_{22}N_2O_2$ [M+Na]$^+$ found 345.1571 requires 345.1579 (–2.3 ppm)

**Melting point:** 148-154 °C ($Et_2O/CH_2Cl_2$)

**1,3-Bis(3,4-dimethoxyphenyl)-2-methylenepropane-1,3-dione (S42)**

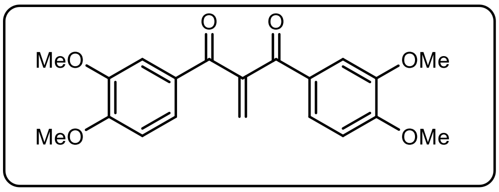


Following general procedure C, 1,3-bis(3,4-dimethoxyphenyl)propane-1,3-dione **S27** (756 mg, 2.2 mmol), potassium persulfate (1.2 g, 4.4 mmol), iron (III) chloride hexahydrate (59.5 mg, 0.22 mmol) and DMA (9 mL) at 110 °C for 30 min, gave after purification by column chromatography (30:70, $Et_2O$:$CH_2Cl_2$, $R_f$ = 0.6). the title compound (543 mg, 69%). as a sticky green oil.

**$^1$H-NMR** (400 MHz, $CDCl_3$) $\delta_H$: 3.92 (6H, s, COC**H**$_3$ ×2), 3.95 (6H, s, COC**H**$_3$ ×2), 6.26 (2H, C=**C**$H_2$), 6.88 (2H, d, *J* 8.5, C**H** Ar ×2), 7.50-7.52 (2H, m, C**H** Ar ×2), 7.54 (2H, dd, *J* 1.9, *J* 8.4, C**H** Ar ×2)

**$^{13}$C-NMR** (126 MHz, $CDCl_3$) $\delta_C$: 56.0 (O**C**$H_3$ ×2), 56.1 (O**C**$H_3$ ×2), 110.0 (**C**H Ar ×2), 111.2 (**C**H Ar ×2), 125.0 (**C**H Ar ×2), 129.3 (C=**C**$H_2$), 129.3 (**C** Ar ×2), 148.6 (**C**=$CH_2$), 149.2 (**C** Ar ×2), 153.7 (**C** Ar ×2), 192.7 (**C**=O)

**HRMS** (ESI$^+$) $C_{20}H_{20}O_6$ [M+H]$^+$ found 357.1330 requires 357.1333 (–0.8 ppm)

**IR $v_{max}$** (film) 2936, 1643 (C=O), 1582 (C=C alkene), 1510, 1418, 1261 (C-O), 1128, 1018

**1,3-Bis(4-bromophenyl)-2-methylenepropane-1,3-dione (S43)**

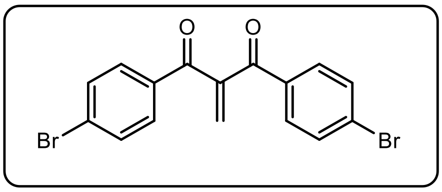


Following general procedure C, 1,3-bis(4-bromophenyl)propane-1,3-dione **S28** (764 mg, 2.2 mmol), potassium persulfate (1.2 g, 4.4 mmol), iron (III) chloride hexahydrate (59.5 mg, 0.22 mmol) and DMA (9 mL) at 110 °C for 10 min, gave after purification via silica plug (30:70, EtOAc:Hexane, $R_f$ = 0.8), the title compound (445.7 mg, 51%) as an orange solid.

**$^1$H-NMR** (400 MHz, $CDCl_3$) $\delta_H$: 6.36 (2H, s, C=C**H**$_2$), 7.63 (4H, d, *J* 8.6, C**H** Ar ×4), 7.75 (4H, d, *J* 8.6, C**H** Ar ×4)

**$^{13}$C-NMR** (126 MHz, $CDCl_3$) $\delta_C$: 129.0 (**C** Ar ×2), 131.0 (**C**H Ar ×4), 131.6 (C=**C**$H_2$), 132.1 (**C**H Ar ×4), 134.8 (**C** Ar ×2), 147.2 (**C**=$CH_2$), 192.9 (**C**=O ×2)

**HRMS** (ESI+) $C_{16}H_{10}Br_2O_2$ $[M+H]^+$ found 392.9128 requires 392.9120 (–2.0 ppm)

**Melting point:** 102-104 °C (EtOAc/hexane)

**1,3-Bis(4-chlorophenyl)-2-methylenepropane-1,3-dione (S44)**

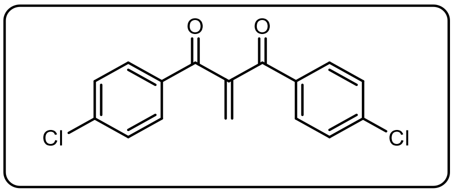


Following general procedure C, 1,3-bis(4-chlorophenyl)propane-1,3-dione **S29** (526 mg, 2.2 mmol), potassium persulfate (1.2 g, 4.4 mmol), iron (III) chloride hexahydrate (59.5 mg, 0.22 mmol) and DMA (9 mL) at 110 °C for 15 min, gave after purification via silica plug (30:70, EtOAc:Hexane, $R_f$ = 0.9), the title compound (303 mg, 45%) as a yellow solid.

**$^1$H-NMR** (400 MHz, $CDCl_3$) $\delta_H$: 6.34 (2H, s, C=C**H**$_2$), 7.46 (4H, d, *J* 7.8, C**H** Ar ×42), 7.83 (4H, d, *J* 7.8, C**H** Ar ×4)

**$^{13}$C-NMR** (126 MHz, $CDCl_3$) $\delta_C$: 129.1 (**C**H Ar ×4), 130.9 (**C**H Ar ×4), 131.3 (C=**C**$H_2$), 134.5 (**C** Ar ×2), 140.2 (**C** Ar ×2), 147.4 (**C**=$CH_2$), 192.6 (**C**=O ×2)

**HRMS** (ESI+) $C_{16}H_{10}Cl_2O_2$ $[M+H]^+$ found 305.0133 requires 305.0131 (–0.8 ppm)

**Melting point:** 77-80 °C (EtOAc/hexane)

**2-Methylene-1,3-di(naphthalen-2-yl)propane-1,3-dione (S45)**

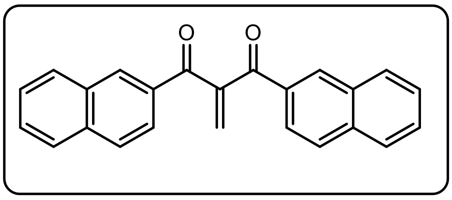


Following general procedure C, 1,3-di(naphthalen-2-yl)propane-1,3-dione **S30** (300 mg, 0.9 mmol), potassium persulfate (485 mg, 3.6 mmol), iron (III) chloride hexahydrate (24.3 mg, 0.18 mmol) and DMA (3.6 mL) at 110 °C for 4 h gave after purification by column chromatography (30:70, EtOAc : hexane, $R_f$ = 0.7) the title compound as a light white solid (116.1 mg, 38%).

**$^1$H-NMR** (500 MHz, $CDCl_3$) $\delta_H$: 6.47 (2H, s, C=C**H**$_2$), 7.55-7.60 (2H, m, C**H** Ar ×2), 7.61-7.66 (2H, m, C**H** Ar ×2), 7.90 (2H, d, *J* 7.5, C**H** Ar ×2), 7.92 (2H, d, *J* 8.9, C**H** Ar ×2), 7.96 (2H, d, *J* 8.3, C(**H** Ar ×2), 8.02 (2H, dd, *J* 8.3, 2.1, C**H** Ar ×2), 8.48 (2H, s, C**H** Ar ×2)

**$^{13}$C-NMR** (126 MHz, $CDCl_3$) $\delta_C$: 124.7 (**C**H Ar ×2), 127.0 (**C**H Ar ×2), 127.9 (**C**H Ar ×2), 128.8 (**C**H Ar ×2), 128.8 (**C**H Ar ×2), 129.7 (**C**H Ar ×2), 130.9 (C=**C**$H_2$), 131.9 (**C**H Ar ×2), 132.4 (**C** Ar ×2), 133.7 (**C** Ar ×2), 135.8 (**C** Ar ×2), 148.4 (**C**=$CH_2$), 194.2 (**C**=O ×2)

**HRMS** ($ESI^+$) $C_{24}H_{16}O_2$ $[M+H]^+$ found 337.1227 requires 337.1223 (+1.2 ppm), $[M+Na]^+$ found 359.1048 requires 359.1048 (0 ppm)

**Melting point:** 103-105 °C (EtOAc/hexane)

**2-Methylene-1,3-di(naphthalen-1-yl)propane-1,3-dione (S46)**

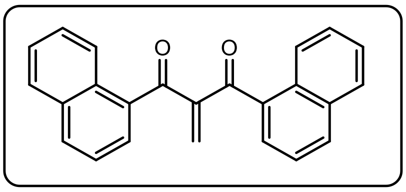

Following general procedure C, 1,3-di(naphthalen-1-yl)propane-1,3-dione **S31** (480 mg, 1.5 mmol), potassium persulfate (811 mg, 3.0 mmol), iron (III) chloride hexahydrate (40.5 mg, 0.15 mmol) and DMA (6 mL) at 110 °C for 15 min, gave after purification by column chromatography (30:70, EtOAc:Hexane, $R_f$ = 0.4) the title compound (153 mg, 30%) as a sticky off-white solid (153 mg, 30%).

**$^1$H-NMR** (400 MHz, $CDCl_3$) $\delta_H$: 6.46 (1H, s, C=C**H**$_2$), 7.42-7.47 (2H, m, C**H** Ar ×2), 7.50-7.60 (4H, m, C**H** Ar ×4), 7.80-7.87 (4H, m, C**H** Ar ×4), 7.92 (2H, d, *J* 8.8, C**H** Ar ×2), 8.45 (2H, d, *J* 8.7, C**H** Ar ×2)

**$^{13}$C-NMR** (126 MHz, $CDCl_3$) $\delta_C$: 124.1 (**C**H Ar ×2), 125.5 (**C**H Ar ×2), 126.6 (**C**H Ar ×2), 127.9 (**C**H Ar ×2), 128.3 (**C**H Ar ×2), 129.2 (**C**H Ar ×2), 130.5 (**C** Ar ×2), 132.7 (C=**C**$H_2$), 132.9 (**C**H Ar ×2), 133.8 (**C** Ar ×2), 134.7 (**C** Ar ×2), 151.5 (**C**=$CH_2$), 195.9 (**C**=O)

**HRMS** ($ESI^+$) $C_{24}H_{16}O_2$ $[M+Na]^+$ found 359.1039 requires 359.1048 (–2.5 ppm)

**IR $\nu_{max}$** (film): 3049 (C-H stretching alkene), 1655 (C=O), 1508, 1310, 1234, 949 (C=C bending), 781

**2-Methylene-1,3-di(thiophen-2-yl)propane-1,3-dione (S47)**

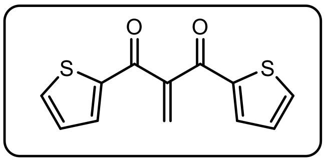

Following general procedure C, 1,3-di(thiophen-2-yl)propane-1,3-dione **S32** (520 mg, 2.2 mmol), potassium persulfate (1.2 g, 4.4 mmol), iron (III) chloride hexahydrate (59.5 mg, 0.22 mmol) and DMA (9 mL) at 110 °C for 4 h, gave after purification by column chromatography (50:50, EtOAc:Hexane, $R_f$ = 0.7), the title compound (319.2 mg, 58 %) as a yellow solid.

**$^{1}$H-NMR** (500 MHz, $CDCl_3$) $\delta_H$: 6.38 (2H, s, C=C**H**$_2$), 7.15 (2H, dd, *J* 3.9, *J* 4.7, C**H** Ar ×2), 7.70 (2H, dd, *J* 1.0, *J* 3.8, C**H** Ar ×2), 7.74 (2H, dd, *J* 1.0, *J* 5.1, C**H** Ar ×2)

**$^{13}$C-NMR** (126 MHz, $CDCl_3$) $\delta_C$: 128.4 (**C**H Ar ×2), 129.4 (C=**C**H$_2$), 134.8 (**C**H Ar ×2), 135.5 (**C**H Ar ×2), 143.0 (**C** Ar ×2), 147.7 (**C**=CH$_2$), 185.1 (**C**=O ×2)

**HRMS** (ESI$^+$) $C_{12}H_8O_2S_2$ $[M+Na]^+$ found 270.9858 requires 270.9863 (–1.8 ppm)

**Melting point:** 94-98 °C (EtOAc/hexane)

**2-Methylene-1,3-di(thiophen-3-yl)propane-1,3-dione (S48)**

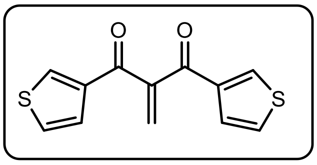


Following general procedure C, 1,3-di(thiophen-3-yl)propane-1,3-dione **S33** (600 mg, 2.5 mmol), potassium persulfate (1.4 g, 5.0 mmol), iron (III) chloride hexahydrate (67.6 mg, 0.25 mmol) and DMA (10 mL) at 110 °C for 1 h, gave after purification by column chromatography ($Et_2O$:$CH_2Cl_2$, 30:70, $R_f$ = 0.8), the title compound (377 mg, 61%) as an orange viscous oil.

**$^{1}$H-NMR** (400 MHz, $CDCl_3$) $\delta_H$: 6.31 (1H, s, C=C**H**$_2$), 7.36 (2H, dd, *J* 3.0, *J* 5.2, C**H** Ar ×2), 7.57 (2H, dd, *J* 1.1, *J* 5.5, C**H** Ar ×2), 8.05 (2H, dd, *J* 1.3, *J* 2.8, C**H** Ar ×2)

**$^{13}$C-NMR** (126 MHz, $CDCl_3$) $\delta_C$: 126.8 (**C**H Ar ×2), 127.7 (**C**H Ar ×2), 129.0 (**C**=CH$_2$), 140.9 (**C** Ar ×2), 149.5 (**C**=CH$_2$), 187.0 (**C**=O ×2)

**HRMS** (ESI$^+$) $C_{12}H_8O_2S_2$ $[M+Na]^+$ found 278.9854 requires 270.9863 (– 3.3 ppm)

**IR $\nu_{max}$** (film) 1659 (C=O), 1634 (C=O), 1506, 1412, 1306, 1246, 1146, 872, 766

**1,3-Di(furan-2-yl)-2-methylenepropane-1,3-dione (S49)**

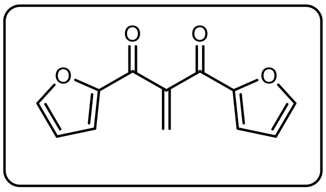


Following general procedure C, 1,3-di(furan-2-yl)propane-1,3-dione **S34** (600 mg, 2.9 mmol), potassium persulfate (1.6 g, 5.8 mmol), iron (III) chloride hexahydrate (78.4 mg, 0.29 mmol) and DMA (12 mL) at 110 °C for 15 min, gave after purification by column chromatography (50:50, EtOAc:Hexane, $R_f$ = 0.4), the title compound (303 mg, 48%) as a yellow solid.

$^{1}$**H-NMR** (400 MHz, $CDCl_3$) $\delta_H$: 6.48 (2H, s, C=C**H**$_2$), 6.56 (2H, dd, *J* 3.8, 1.7, C**H** Ar ×2), 7.26 (2H, dd, *J* 3.6, 0.7, C**H** Ar ×2), 7.62 (2H, dd, *J* 1.6, 0.8, C**H** Ar ×2)

$^{13}$**C-NMR** (126 MHz, $CDCl_3$) $\delta_C$: 112.6 (**C**H Ar ×2), 120.2 (**C**H Ar ×2), 131.2 (C=**C**H$_2$), 146.4 (**C**=CH$_2$), 147.6 (**C**H Ar ×2), 151.7 (**C** Ar ×2), 179.7 (**C**=O ×2)

**HRMS** $C_{12}H_8O_4$ $[M+H]^+$ found 217.0489 requires 217.0495 (–2.8 ppm)

**Melting point:** 78-82 °C (EtOAc/hexane)

**1,3-Bis(benzo[d][1,3]dioxol-5-yl)-2-methylenepropane-1,3-dione (S50)**

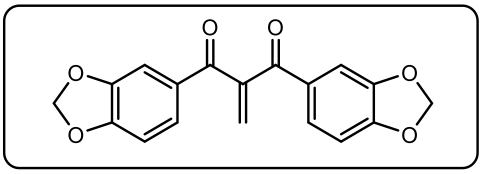

Following general procedure C, 1,3-bis(benzo[d][1,3]dioxol-5-yl)propane-1,3-dione **S35** (687 mg, 2.2 mmol), potassium persulfate (1.2 g, 4.4 mmol), iron (III) chloride hexahydrate (59.5 mg, 0.22 mmol) and DMA (9 mL) at 110 °C for 1 h, gave after purification by column chromatography (30:70, $Et_2O:CH_2Cl_2$, $R_f$ = 0.8), the title compound (365.3 mg, 51%) as a yellow sticky solid.

$^{1}$**H-NMR** (400 MHz, $CDCl_3$) $\delta_H$: 6.07 (4H, s, OC**H**$_2$O ×2), 6.23 (2H, s, C=C**H**$_2$), 6.85 (2H, d, *J* 8.1, C**H** Ar ×2), 7.40 (2H, d, *J* 1.6, C**H** Ar ×2), 7.49 (2H, dd, *J* 1.7, *J* 8.2, C**H** Ar ×2)

$^{13}$**C-NMR** (126 MHz, $CDCl_3$) $\delta_C$: 102.0 (O**C**H$_2$O), 108.0 (**C**H Ar ×2), 109.0 (**C**H Ar ×2), 126.7 (**C**H Ar ×2), 129.5 (**C**=CH$_2$), 130.9 (**C** Ar ×2), 148.3 (**C**=CH$_2$ and **C**OCH$_2$), 152.3 (**C**OCH$_2$), 192.2 (**C**=O)

**HRMS** (ESI$^+$) $C_{18}H_{12}O_6$ $[M+H]^+$ found 325.0704 requires 325.0707 (–0.9 ppm)

**Melting point:** 98-104 °C ($CH_2Cl_2$)

**1-Phenylprop-2-en-1-one (S51)**

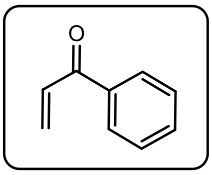

Following general procedure C, acetophenone (486 µL, 4.2 mmol), potassium persulfate (2.3 g, 8.4 mmol), iron (III) chloride hexahydrate (113.5 mg, 0.42 mmol) and DMA (17 mL) at 110 °C for 4 h, gave after purification by column chromatography (10:90, EtOAc:Hexane, $R_f$ = 0.3), the title compound (201 mg, 36%) as a yellow liquid.

**$^{1}$H-NMR** (400 MHz, $CDCl_3$) $\delta_H$: 5.93 (1H, dd, *J* 10.5, 1.7, CH=C**H$^a$**H), 6.44 (1H, dd, *J* 17.4, 1.6 CH=CH**H$^b$**), 7.16 (1H, dd, *J* 16.9, 10.6, C**H**=$CH_2$), 7.45-7.50 (2H, m, C**H** Ph x2), 7.55-7.59 (1H, m, C**H** Ph x1), 7.92-7.97 (2H, m, C**H** Ph x2)

**$^{13}$C-NMR** (126 MHz, $CDCl_3$) $\delta_C$: 128.7 (**C**H Ph x2), 128.7 (**C**H Ph x2), 130.3 (CH=**C**$H_2$), 132.4 (**C**H=$CH_2$), 133.0 (**C**(4)H Ph), 137.3 (**C**(1) Ph), 191.1 (**C**=O)

**HRMS** (ESI$^+$) $C_9H_8O$ $[M+H]^+$ found 133.0644 requires 133.0648 (–3.0 ppm)

**IR v$_{max}$** (liquid) 1670 (C=O), 1607 (C=C stretching alkene), 1595, 1447, 1402, 1231

## 3.9 Synthesis of quinone methides

**(*E*)-4-benzylidene-2-(tert-butyl)-6-methylcyclohexa-2,5-dien-1-one (7a)**

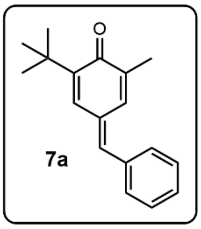

Quinone methide **7a** was synthesized according to general procedure D. Yield and spectral data were in accordance with literature [30].

**1H-NMR** (300 MHz, CDCl3, 298 K): δ / ppm = 7.49-7.38 (m, 6 H, Ar-**H** + -C**H**), 7.21 (s, 1 H, -C**H**), 7.07 (d, J = 2.6 Hz, 1 H, -C**H**), 2.04 (s, 3 H, -C**H$_3$**), 1.34 (s, 9 H, -C**H$_3$**).

**4-benzylidene-2,6-di-tert-butylcyclohexa-2,5-dien-1-one (7b)**

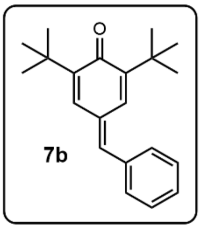

Quinone methide **7b** was synthesized according to general procedure D. Yield and spectral data were in accordance with literature [30].

**$^{1}$H-NMR** (300 MHz, $CDCl_3$, 298 K): *δ* / ppm = 7.52 (d, *J* = 2.4 Hz, 1 H, -C**H**), 7.46-7.38 (m, 5 H, Ar-**H**), 7.18 (s, 1 H, -C**H**), 7.01 (d, *J* = 2.4 Hz, 1 H, -C**H**), 1.33 (s, 9 H, -C**H$_3$**), 1.29 (s, 9 H, -C**H$_3$**).

**4-benzylidene-2,6-diisopropylcyclohexa-2,5-dien-1-one (7c)**

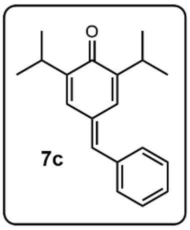

Quinone methide **7c** was synthesized according to general procedure D. Yield and spectral data were in accordance with literature [31].

**$^{1}$H-NMR** (300 MHz, CDCl3, 298 K): δ / ppm = 7.48-7.38 (m, 6 H, Ar-**H** + -C**H**), 7.23 (s, 1 H, -C**H**), 6.98 (d, *J* = 2.4 Hz, 1 H, -C**H**), 3.19 (hept., *J* = 6.9 Hz, 2 H, -C**H**), 1.17 (d, *J* = 6.9 Hz, 6 H, -C**H$_3$**), 1.13 (d, *J* = 6.9 Hz, 6 H, -C**H$_3$**).

**2,6-di-tert-butyl-4-(2-methylbenzylidene)cyclohexa-2,5-dien-1-one (7d)**

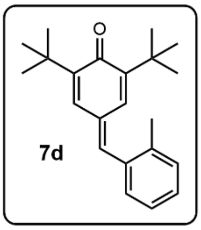


Quinone methide **7d** was synthesized according to general procedure D. Yield and spectral data were in accordance with literature [32].

**$^{1}$H-NMR** (300 MHz, $CDCl_3$, 298 K): $\delta$ / ppm = 7.34 (d, $J$ = 2.3 Hz, 1 H, -C**H**), 7.29-7.27 (m, 5 H, Ar-**H**), 7.06 (d, $J$ = 2.3 Hz, 1 H, -C**H**), 2.38 (s, 3 H, -C**H$_3$**), 1.34 (s, 9 H, -C**H$_3$**), 1.26 (s, 9 H, -C**H$_3$**).

**2,6-di-tert-butyl-4-(3-methylbenzylidene)cyclohexa-2,5-dien-1-one (7e)**

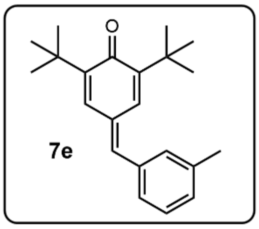


Quinone methide **7e** was synthesized according to general procedure D. Yield and spectral data were in accordance with literature [32].

**$^{1}$H-NMR** (300 MHz, $CDCl_3$, 298 K): $\delta$ / ppm = 7.54 (d, $J$ = 2.3 Hz, 1 H, -C**H**), 7.34-7.16 (m, 5 H, Ar-**H** + -C**H**), 7.01 (d, $J$ = 2.3 Hz, 1 H, -C**H**), 2.41 (s, 3 H, -C**H$_3$**), 1.33 (s, 9 H, -C**H$_3$**), 1.30 (s, 9 H, -C**H$_3$**).

**2,6-di-tert-butyl-4-(4-methylbenzylidene)cyclohexa-2,5-dien-1-one (7f)**

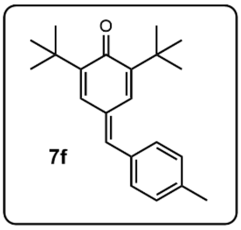


Quinone methide **7f** was synthesized according to general procedure D. Yield and spectral data were in accordance with literature [30].

**$^{1}$H-NMR** (300 MHz, $CDCl_3$, 298 K): $\delta$ / ppm = 7.55 (d, $J$ = 2.4 Hz, 1 H, -C**H**), 7.37 (d, $J$ = 8.1 Hz, 2 H, Ar-**H**), 7.26 (d, $J$ = 8.1 Hz, 2 H, Ar-**H**), 7.16 (s, 1 H, -C**H**), 7.01 (d, $J$ = 2.4 Hz, 1 H, -C**H**), 2.41 (s, 3 H, -C**H$_3$**), 1.33 (s, 9 H, -C**H$_3$**), 1.30 (s, 9 H, -C**H$_3$**).

**2,6-di-tert-butyl-4-(2-methoxybenzylidene)cyclohexa-2,5-dien-1-one (7g)**

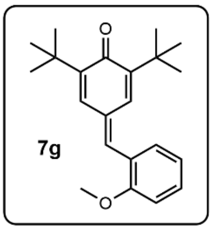


Quinone methide **7g** was synthesized according to general procedure D. Yield and spectral data were in accordance with literature [32].

**$^{1}$H-NMR** (300 MHz, CDCl3, 298 K): δ / ppm = 7.47 (d, J = 2.3 Hz, 1 H, -C**H**), 7.36-7.42 (m, 3 H, Ar-**H** + -C**H**), 7.08 (d $J$ = 2.3 Hz, 1 H, -C**H**), 7.04 (t, $J$ = 3.7 Hz, 1 H, Ar-**H**), 6.96 (d, $J$ = 8.5 Hz, 1 H, Ar-**H**), 3.90 (s, 3 H, -OC**H$_3$**), 1.34 (s, 9 H, -C**H$_3$**), 1.29 (s, 9 H, -C**H$_3$**).

**2,6-di-tert-butyl-4-(4-methoxybenzylidene)cyclohexa-2,5-dien-1-one (7h)**

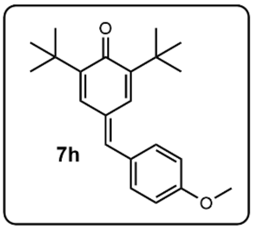


Quinone methide **7h** was synthesized according to general procedure D. Yield and spectral data were in accordance with literature [30].

**$^{1}$H-NMR** (300 MHz, CDCl3, 298 K): δ / ppm = 7.56 (d, *J* = 2.3 Hz, 1 H, -C**H**), 7.44 (d, *J* = 8.6 Hz, 2 H, Ar-**H**), 7.13 (s, 1 H, -C**H**), 7.00 (d, *J* = 2.3 Hz, 1 H, -C**H**), 6.98 (d, *J* =8.6 Hz, 2 H, Ar-**H**), 3.87 (s, 3 H, -OC**H**$_3$), 1.33 (s, 9 H, -C**H**$_3$), 1.32 (s, 9 H, -C**H**$_3$).

**2,6-di-tert-butyl-4-(4-(dimethylamino)benzylidene)cyclohexa-2,5-dien-1-one (7i)**

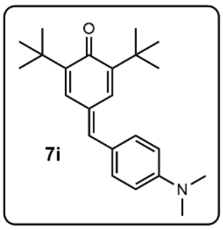


Quinone methide **7i** was synthesized according to general procedure D. Yield and spectral data were in accordance with literature [33].

**$^{1}$H-NMR** (300 MHz, CDCl3, 298 K): δ / ppm = 7.67 (d, *J* = 2.4 Hz, 1 H, -C**H**), 7.45 (d, *J* = 8.9 Hz, 2 H, Ar-**H**), 7.10 (s, 1 H, -C**H**), 7.00 (d, *J* = 2.4 Hz, 1 H, -C**H**), 6.75 (d, *J* = 8.9 Hz, 2 H, Ar-**H**), 3.07 (s, 6 H, -NC**H**$_3$), 1.34 (s, 9 H, -C**H**$_3$), 1.33 (s, 9 H, -C**H**$_3$).

**2,6-di-tert-butyl-4-(4-(tert-butyl)benzylidene)cyclohexa-2,5-dien-1-one (7j)**

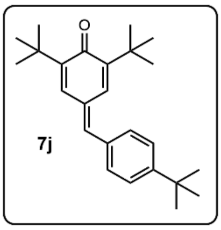


Quinone methide **7j** was synthesized according to general procedure D. Yield and spectral data were in accordance with literature [32].

**$^{1}$H-NMR** (300 MHz, CDCl3, 298 K): δ / ppm = 7.75 (d, *J* = 2.2 Hz, 1 H, -C**H**), 7.59 (d, *J* = 8.5 Hz, 2 H, Ar-**H**), 7.54 (d, *J* = 8.5 Hz, 2 H, Ar-**H**), 7.26 (s, 1 H, -C**H**), 7.14 (d, *J* = 2.2 Hz, 1 H, -C**H**), 1.49 (s, 9 H, -C**H**$_3$), 1.47 (s, 9 H, -C**H**$_3$), 1.47 (s, 9 H, -C**H**$_3$).

**2,6-di-tert-butyl-4-(4-(trifluoromethyl)benzylidene)cyclohexa-2,5-dien-1-one (7k)**

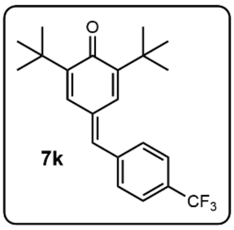


Quinone methide **7k** was synthesized according to general procedure D. Yield and spectral data were in accordance with literature [30].

**$^{1}$H-NMR** (300 MHz, CDCl3, 298 K): δ / ppm = 7.71 (d, *J* = 8.1 Hz, 2 H, Ar-**H**), 7.55 (D, *J* = 8.1 Hz, 2 H, Ar-**H**), 7.42 (d, *J* = 2.3 Hz, 1 H, -C**H**), 7.16 (s, 1 H, -C**H**), 7.02 (d, *J* = 2.3 Hz, 1 H, -C**H**), 1.33 (s, 9 H, -C**H**$_3$), 1.29 (s, 9 H, -C**H**$_3$).

**2,6-di-tert-butyl-4-(4-fluorobenzylidene)cyclohexa-2,5-dien-1-one (7l)**

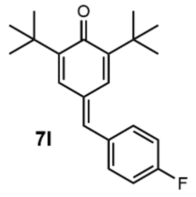

7l

Quinone methide **7l** was synthesized according to general procedure D. Yield and spectral data were in accordance with literature [30].

**$^{1}$H-NMR** (300 MHz, CDCl3, 298 K): δ / ppm = 7.46-7.41 (m, 3 H, Ar-**H** + -C**H**), 7.18-7.12 (m, 3 H, Ar-**H** + -C**H**), 7.00 (d, *J* = 2.4 Hz, 1 H, -C**H**), 1.33 (s, 9 H, -C**H$_3$**), 1.29 (s, 9 H, -C**H$_3$**).

**2,6-di-tert-butyl-4-(4-chlorobenzylidene)cyclohexa-2,5-dien-1-one (7m)**

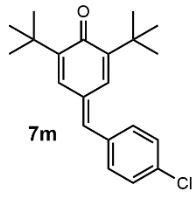

7m

Quinone methide **7m** was synthesized according to general procedure D. Yield and spectral data were in accordance with literature [30].

**$^{1}$H-NMR** (300 MHz, CDCl3, 298 K): δ / ppm = 7.41-7.46 (m, 3 H, Ar-**H** + -C**H**), 7.38 (d, *J* = 8.7 Hz, 2 H, Ar-**H**), 7.11 (s, 1 H, -C**H**), 7.00 (d, *J* = 2.4 Hz, 1 H, -C**H**), 1.33 (s, 9 H, -C**H$_3$**), 1.30 (s, 9 H, -C**H$_3$**).

**4-(4-bromobenzylidene)-2,6-di-tert-butylcyclohexa-2,5-dien-1-one (7n)**

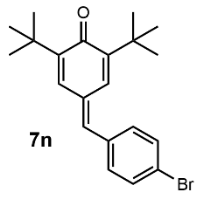

7n

Quinone methide **7n** was synthesized according to general procedure D. Yield and spectral data were in accordance with literature [31].

**$^{1}$H-NMR** (300 MHz, CDCl3, 298 K): δ / ppm = 7.60 (d, *J* = 8.4 Hz, 2 H, Ar-**H**), 7.46 (d, *J* = 2.3 Hz, 1 H, -C**H$_3$**), 7.33 (d, *J* = 8.4 Hz, 2 H, Ar-**H**), 7.10 (s, 1 H, -C**H**), 7.02 (d, *J* = 2.3 Hz, 1 H, -C**H**), 1.36 (s, 9 H, -C**H$_3$**), 1.33 (s, 9 H, -C**H$_3$**).

**2,6-di-tert-butyl-4-(naphthalen-1-ylmethylene)cyclohexa-2,5-dien-1-one (7o)**

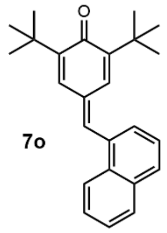

7o

Quinone methide **7o** was synthesized according to general procedure D. Yield and spectral data were in accordance with literature [30].

**$^{1}$H-NMR** (300 MHz, $CDCl_3$, 298 K): *δ* / ppm = 8.04-8.00 (m, 1 H, Ar-**H**), 7.91-7.89 (m, 2 H, Ar-**H**), 7.77 (s, 1 H, -C**H**), 7.57-7.46 (m, 4 H, Ar-**H**), 7.38 (d, *J* = 2.2 Hz, 1 H, -C**H**), 7.19 (d, *J* = 2.2 Hz, 1 H, Ar-**H**), 1.37 (s, 9 H, -C**H$_3$**), 1.24 (s, 9 H, -C**H$_3$**).

**2,6-di-tert-butyl-4-(naphthalen-2-ylmethylene)cyclohexa-2,5-dien-1-one (7p)**

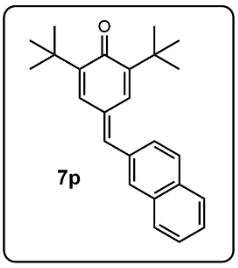


Quinone methide **7p** was synthesized according to general procedure D. Yield and spectral data were in accordance with literature [32].

**$^1$H-NMR** (300 MHz, $CDCl_3$, 298 K): *δ* / ppm = 7.94-7.86 (m, 4 H, Ar-**H**), 7.65-7.53 (m, 4 H, Ar-**H** + -C**H**), 7.34 (br., 1 H, -C**H**), 7.08 (d, *J* = 2.2 Hz, 1 H, -C**H**), 1.36 (s, 9 H, -C**H₃**), 1.32 (s, 9 H, -C**H₃**).

**2,6-di-tert-butyl-4-(thiophen-2-ylmethylene)cyclohexa-2,5-dien-1-one (7q)**

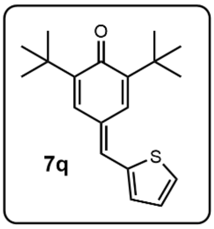


Quinone methide **7q** was synthesized according to general procedure D. Yield and spectral data were in accordance with literature [31].

**$^1$H-NMR** (300 MHz, CDCl3, 298 K): δ / ppm = 7.87 (d, *J* = 2.5 Hz, 1 H, -C**H**), 7.57 (d, *J* = 5.1 Hz, 1 H, Ar-**H**), 7.33 (d, *J* = 3.7 Hz, 1 H, Ar-**H**), 7.19 (s, 1 H, -C**H**), 7.13 (dd, $J_1$ = 3.7 Hz, $J_2$ = 5.1 Hz, 1 H, Ar-**H**), 6.96 (d, *J* = 2.5 Hz, 1 H, -C**H**), 1.37 (s, 9 H, -C**H₃**), 1.32 (9 H, -C**H₃**).

**2,6-di-tert-butyl-4-(pyridin-3-ylmethylene)cyclohexa-2,5-dien-1-one (7r)**

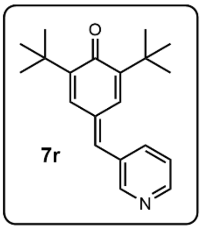


Quinone methide **7r** was synthesized according to general procedure D. Yield and spectral data were in accordance with literature [33].

**$^1$H-NMR** (300 MHz, CDCl3, 298 K): δ / ppm = 8.71 (d, *J* = 1.3 Hz, 1 H, Ar-**H**), 8.61 (dd, $J_1$ = 1.3 Hz, $J_2$ = 4.7 Hz, 1 H, Ar-**H**), 7.76 (d, *J* = 7.9 Hz, 1 H, Ar-**H**), 7.41-7.37 (m, 2 H, Ar-**H** + -C**H**), 7.10 (s, 1 H, -C**H**), 7.02 (d, *J* = 2.4 Hz, 1 H, -C**H**), 1.33 (s, 9 H, -C**H₃**), 1.29 (s, 9 H, -C**H₃**).

**2-(Methoxymethoxy)benzaldehyde (13ba)**

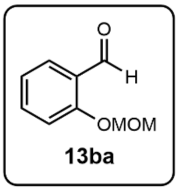


MOM protected aldehyde **13ba** was synthesized according to general procedure E described above. Yield and spectral data were in accordance with literature [34].

**$^1$H-NMR** (300 MHz, $CDCl_3$, 298 K): *δ* / ppm = 10.51 (d, *J* = 0.7 Hz, 1 H, -C**H**O), 7.84 (dd, $J_1$ = 1.8 Hz, $J_2$ = 7.6 Hz, 1 H, Ar-**H**), 7.56-7.50 (m, 1 H, Ar-**H**), 7.22 (d, *J* = 8.4 Hz, 1 H, Ar-**H**), 7.08 (t, *J* = 7.6 Hz, 1 H, Ar-**H**), 5.30 (s, 2 H, -OC**H₂**), 3.52 (s, 3 H, -OC**H₃**).

### 2-(Methoxymethoxy)-1-naphthaldehyde (13ca)

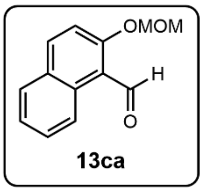


MOM protected aldehyde **13ca** was synthesized according to general procedure E described above. Yield and spectral data were in accordance with literature [35].

**$^1$H-NMR** (300 MHz, $CDCl_3$, 298 K): *δ* / ppm = 10.94 (s, 1 H, -C**H**O), 9.28-9.25 (m, 1 H, Ar-**H**), 8.03 (d, *J* = 9.2 Hz, 1 H, Ar-**H**), 7.79-7.76 (m, 1 H, Ar-**H**), 7.65-7.60 (m, 1 H, Ar-**H**), 7.47-7.41 (m, 2 H, Ar-**H**), 5.40 (s, 2 H, -OC$\mathbf{H_2}$), 3.56 (s, 3 H, -OC$\mathbf{H_3}$).

### 2,6-di-tert-butyl-4-(2-(methoxymethoxy)benzylidene)cyclohexa-2,5-dien-1-one (13b)

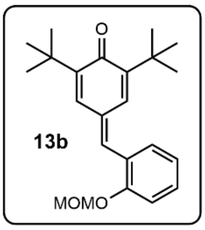


Following general procedure E, MOM protected quinone methide **13b** was obtained as a sticky yellow oil that solidified after prolonged storage in a yield of 69%.

**$^1$H-NMR** (300 MHz, $CDCl_3$, 298 K): *δ* / ppm = 7.46 (d, *J* = 2.3 Hz, 1 H, -C**H**), 7.40-7.34 (m, 3 H, Ar-**H** + -C**H**), 7.21 (d, *J* = 8.5 Hz, 1 H, Ar-**H**), 7.11-7.06 (m, 2 H, Ar-**H** + -C**H**), 5.26 (s, 2 H, -OC$\mathbf{H_2}$), 3.51 (s, 3 H, -OC$\mathbf{H_3}$), 1.34 (s, 9 H, -C$\mathbf{H_3}$), 1.29 (s, 9 H, -C$\mathbf{H_3}$).

**$^{13}$C-NMR** (75 MHz, $CDCl_3$, 298 K) *δ* / ppm = 186.8 (1 C, -**C**=O), 156.3 (1 C, $\mathbf{C_{Ar}}$), 149.2 (1 C, $\mathbf{C_q}$), 147.6 (1 C, $\mathbf{C_q}$), 138.7 (1 C, $\mathbf{C_{Ar}}$), 138.7 (1 C, -**C**H), 135.4 (1 C, -**C**H), 132.0 (1 C, $\mathbf{C_{Ar}}$), 131.8 (1 C, $\mathbf{C_q}$),130.9 (1 C, $\mathbf{C_{Ar}}$), 128.4 (1 C, $\mathbf{C_{Ar}}$), 125.8 (1 C, -**C**H), 121.8 (1 C, -$\mathbf{C_{Ar}}$), 114.7 (1 C, -$\mathbf{C_{Ar}}$), 94.8 (1 C, -O**C**$H_2$O), 56.5 (1 C, -O**C**$H_3$), 35.6 (1 C, $\mathbf{C_q}$), 35.2 (1 C, $\mathbf{C_q}$), 29.7 (3 C, -**C**$H_3$), 29.7 (3 C, -**C**$H_3$).

**Melting point:** 71.8 – 73.1 °C

**HRMS:** *m/z:* $[M+H]^+$ calcd for $C_{23}H_{30}O_3^+$ 355.2268 found 355.2273

### 2,6-di-tert-butyl-4-((2-(methoxymethoxy)naphthalen-1-yl)methylene)cyclohexa-2,5-dien-1-one (13c)

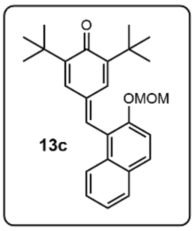


Following general procedure E, MOM protected quinone methide **13c** was obtained as a yellow solid in a yield of 50%.

**$^1$H-NMR** (300 MHz, CDCl3, 298 K): *δ* / ppm = 7.88 (d, *J* = 9.1 Hz, 1 H, Ar-**H**), 7.85-7.81 (m, 1 H, Ar-**H**), 7.78-7.75 (m, 1 H, Ar-**H**), 7.51 (d, *J* = 9.1 Hz, 1 H, Ar-**H**), 7.49-7.38 (m, 3 H, Ar-**H** + -C**H**), 7.20 (d, *J* = 2.4 Hz, 1 H, -C**H**), 6.88 (d, *J* = 2.4 Hz, 1 H, -C**H**), 5.27 (s, 2 H, -OC$\mathbf{H_2}$), 3.50 (s, 3 H, -OC$\mathbf{H_3}$), 1.38 (s, 9 H, -C$\mathbf{H_3}$), 1.11 (s, 9 H, -C$\mathbf{H_3}$).

**$^{13}$C-NMR** (75 MHz, $CDCl_3$, 298 K) *δ* / ppm = 187.0 (1 C, -**C**=O), 153.1 (1 C, $\mathbf{C_{Ar}}$), 148.3 (1 C, $\mathbf{C_q}$), 147.6 (1 C, $\mathbf{C_q}$), 137.2 (1 C, -**C**H), 134.7 (1 C, -**C**H), 134.3 (1 C, $\mathbf{C_q}$), 132.7 (1 C, $\mathbf{C_{Ar}}$), 131.1 (1 C, $\mathbf{C_{Ar}}$), 129.9 (1 C, $\mathbf{C_{Ar}}$), 129.7 (1 C, $\mathbf{C_{Ar}}$), 128.4 (1 C, $\mathbf{C_{Ar}}$), 127.0 (1 C, $\mathbf{C_{Ar}}$), 125.2 (1 C, $\mathbf{C_{Ar}}$),124.6 (1 C, -**C**H), 119.7 (1 C, $\mathbf{C_{Ar}}$), 116.2 (1 C, $\mathbf{C_{Ar}}$), 85.5 (1 C, -O**C**$H_2$O), 56.5 (1 C, -O**C**$H_3$), 35.3 (1 C, $\mathbf{C_q}$), 35.2 (1 C, $\mathbf{C_q}$), 29.7 (3 C, -**C**$H_3$), 29.6 (3 C, -**C**$H_3$).

**Melting point:** 113.4 – 115.6 °C

**HRMS:** *m/z:* $[M+H]^+$ calcd for $C_{27}H_{33}O_3^+$ 405.2424 found 405.2427

**Synthesis of methyl quinone methide (7s)**

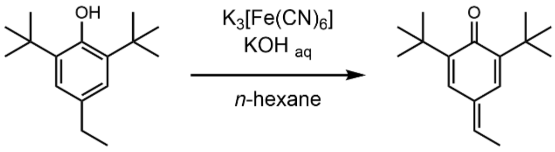


**7sa** **7s**

According to literature [36], A solution of $K_3[Fe(CN)_6]$ (5.62 g, 17.1 mmol, 4 eq) and KOH (1.00 g, 17.9 mmol, 4.2 eq) in 50 mL deionized water was added to a stirred solution of 2,6-di *t*-butyl-4-ethyl phenol (1.00 g, 4.3 mmol, 1 eq) in 120 mL *n*-hexane. The biphasic mixture was stirred at r.t. for 1.5 h, after which the layers were separated and the aqueous phase was extracted with heptanes. The combined organic phases were dried over $Na_2SO_4$, filtered, and concentrated. The crude was purified by column chromatography on silica (heptanes/EtOAc 20/1) to yield quinone methide **7s** as a yellow solid in a yield of 80%.

**$^1$H-NMR** (300 MHz, $CDCl_3$, 298 K): *δ* / ppm = 7.30 (d, *J* = 2.6 Hz, 1 H, -C**H**), 6.84 (d, *J* = 2.6 Hz, 1 H, -C**H**), 6.40 (q, *J* = 7.7 Hz, 1 H, -C**H**), 2.12 (d, *J* = 7.7 Hz, 3 H, -C**H$_3$**), 1.32 (s, 9 H, -C**H$_3$**), 1.28 (s, 9 H, -C**H$_3$**).

**CF3 quinone methide (7t)**

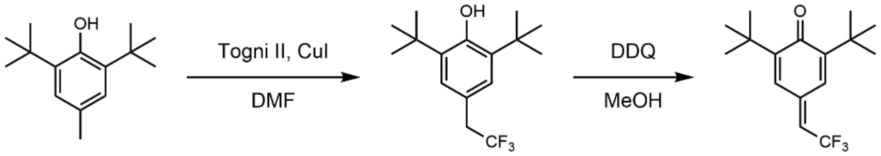


**7taa** **7ta** **7t**

According to a known procedure [37], CuI (68 mg, 0.36 mmol, 0.1 eq) and Togni II reagent (1.73 g, 5.4 mmol, 1.5 eq)were dissolved in 18 mL DMF. Then, 2,6-di *t*-butyl-4-methyl phenol was added and the mixture was stirred at 40 °C for 1 h. It was diluted with EtOAc and a saturated solution of $NaHCO_3$ was added. The phases were separated, and the organic layer was dried with $Na_2SO_4$, filtered, and concentrated .The crude was purified by column chromatography on silica (heptanes/EtOAc 20/1) to give the trifluoro methylated phenol **7ta** as a yellow residue in a yield of 1.33 g (85%).

**$^1$H-NMR** (300 MHz, $CDCl_3$, 298 K): *δ* / ppm = 7.07 (s, 2 H, **Ar**-H), 5.23 (s, 1 H, -O**H**), 3.27 (q, $J_{H\text{-}F}$ = 11.0 Hz, 2 H, -C**H**), 1.45 (s, 18 H, -C**H$_3$**).

**$^{19}$F-NMR** (282 MHz, $CDCl_3$, 298 K) *δ* / ppm = -74.9 (3 F, -C**F$_3$**).

The product obtained in the first step (0.58 g, 2.0 mmol, 1 eq) was dissolved in 40 mL MeOH and DDQ (1.02 g, 4.5 mmol, 2.25 eq) was added. The mixture was stirred at rt for 1 h and then concentrated on the rotary evaporator. The crude was submitted to column chromatography on silica (heptanes/DCM 10/1) to give quinone methide **7t** as a yellow solid (0.46 g, 80%).

**$^{1}$H-NMR** (300 MHz, $CDCl_3$, 298 K): $\delta$ / ppm = 7.33 (br. 1 H, Ar-**H**), 6.78 (d, $J$ = 2.30 Hz, 1 H, Ar-**H**), 6.03 (q, $J_{H\text{-}F}$ = 9.0 Hz, 1 H, -C**H**), 1.29 (s, 9 H, -C$\mathbf{H_3}$), 1.28 (s, 9 H, -C$\mathbf{H_3}$).

**$^{19}$F-NMR** (282 MHz, $CDCl_3$, 298 K) $\delta$ / ppm = -55.43 (3 F, -C$\mathbf{F_3}$).

# 4 1,6-Addition of Glycine Schiff base aryl ester to p-quinone methides

## 4.1 General information on all reactions between Schiff bases and quinone methides

All asymmetric α-functionalization reactions were run under an atmosphere of argon in freshly degassed (three freeze pump thaw cycles) acetonitrile to prevent imine hydrolysis by dissolved oxygen. Silica for column chromatography was deactivated with $Et_3N$ prior to use in all cases where the imine motif was present. Therefore, 5 mL of $Et_3N$ was added to a slurry of 150 g silica in petrol ether. The solvent was evaporated and the deactivated silica was further dried under high vacuum.

Racemic samples for HPLC analysis were obtained by performing the reaction without the addition of an ITU catalyst or, in case of inferior conversion, using Okamoto's catalyst . This usually led to inferior diastereoselectivity.

Diastereomeric ratio was calculated from crude $^1$H- and $^{19}$F-NMR spectra and further proofed with HPLC chromatograms.

## 4.2 General procedure F for the alkylation of Schiff base ester with *p*-QMs

At -40 °C (using a circulation chiller), Schiff base **1** (18.0 mg, 0.05 mmol, 1 eq) and TM·HCl (1.2 mg, 10 mol%) were dissolved in freshly degassed ACN (0.07 mol $L^{-1}$). Then, DIPEA (8.7 µL, 0.05 mmol, 1 eq) was added and the mixture was stirred for 5 min. The respective quinone methide (0.05 mmol, 1 eq) was added in one portion and the mixture was further stirred for 15 h. After this, the circulation chiller was turned off, and the mixture was allowed to reach rt over 1 h. It was filtered over $Na_2SO_4$ and washed with 2 mL DCM trice. The filtrate was concentrated on the rotary evaporator and the crude products were purified by column chromatography on deactivated silica (heptanes followed by heptanes/EtOAc 10/1 and 5/1). Products were obtained as a mixture of diastereomers.

For the reaction with *p*QM **8o**, the procedure was also performed on a 1.0 mmol scale using the same stoichiometry.

## 4.3 Derivatisation to the corresponding morpholine amide for HPLC analysis

The purified alkylated esters were treated with a 10-fold excess of morpholine and 20 mol% DMAP in ACN and stirred at r.t. for 3 h. After this, the crudes were directly submitted to column chromatography on deactivated silica (heptanes/EtOAc 10/1 followed by 5/1 and 2/1) to obtain the corresponding morpholine amides.

## 4.4 Products of asymmetric alkylation of glycine Schiff base with *p*-QMs

**(2*R*, 3*R*)-4-nitrophenyl 3-(3-(tert-butyl)-4-hydroxy-5-methylphenyl)-2-((diphenylmethylene)amino)-3-phenylpropanoate (8a)**

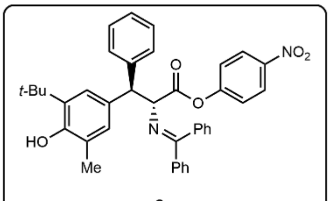


**8a**

Following general procedure F, the product was obtained as a white residue in a yield of 21.4 mg (77%) as a mixture of diastereomers (*dr* 85:15, $er_{major}$ 93:7, $er_{minor}$ 56:44).

**HPLC** (YMC-SB, n-hexane/IPA = 10/1, flow = 0.5 mL min$^{-1}$, $T_{Column}$ = 10 °C, *l* = 250 nm): $t_R$(major diastereomer): 26.76 min (major), 32.62 min (minor); $t_R$(minor diastereomer): 24.92 min (major), 39.37 min (minor).

$r_f$ (heptanes/EtOAc = 5/1) = 0.45

$[\alpha]_D^{24}$ (c 1, $CHCl_3$) = 109

**$^1$H-NMR** (300 MHz, $CDCl_3$, 298 K) *δ* / ppm = 8.12 (d, *J* = 9.2 Hz, 2 H, Ar-**H**), 7.59-7.56 (m, 2 H, Ar-**H**), 7.42-7.29 (m, 10 H, Ar-**H**), 7.20 (d, *J* = 1.9 Hz, 2 H, Ar-**H**), 6.77 (d, *J* = 2.1 Hz, 1 H, Ar-**H**), 6.73 (d, *J* = 9.2 Hz, 2 H, Ar-**H**), 6.70-6.63 (m, 2 H, Ar-**H**), 4.95 (d, *J* = 9.4 Hz, 1 H, -C**H**), 4.90 (d, *J* = 9.4 Hz, 1 H, -C**H**), 4.66 (s, 1 H, -O**H**), 2.12 (s, 3 H, -C**H₃**), 1.30 (s, 9 H, -C**H₃**). *Characteristic signals for minor diastereomer:* 7.05 (d, *J* = 1.9 Hz, 2 H, Ar-**H**), 6.88 (d, *J* = 2.1 Hz, 1 H, Ar-**H**), 4.94 (d, *J* = 8.4 Hz, 1 H, -C**H**), 4.79 (d, *J* = 8.4 Hz, 1 H, -C**H**), 4.69 (s, 1 H, -O**H**), 2.14 (s, 3 H, -$C\mathbf{H_3}$), 1.31 (s, 9 H, -$C\mathbf{H_3}$).

**$^{13}$C-NMR** (75 MHz, $CDCl_3$, 298 K) *δ* / ppm = 172.2 (1 C, -**C**OOR), (1 C, -**C**=N), 169.3 (1 C, -**C**=N), (1 C, -**C**OOR), 155.6 (1 C, $\mathbf{C_{Ar}}$), 151.7 (1 C, $\mathbf{C_{Ar}}$), 145.5 (1 C, $\mathbf{C_{Ar}}$), 141.1 (1 C, $\mathbf{C_{Ar}}$), 139.4 (1 C, $\mathbf{C_{Ar}}$), 135.8 (1 C, $\mathbf{C_{Ar}}$), 135.3 (1 C, $\mathbf{C_{Ar}}$), 131.5 (1 C, $\mathbf{C_{Ar}}$), 130.7 (1 C, $\mathbf{C_{Ar}}$), 129.1 (2 C, $\mathbf{C_{Ar}}$), 129.0 (1 C, $\mathbf{C_{Ar}}$), 128.9 (1 C, $\mathbf{C_{Ar}}$), 128.8 (2 C, $\mathbf{C_{Ar}}$), 128.7 (2 C, $\mathbf{C_{Ar}}$), 128.3 (2 C, $\mathbf{C_{Ar}}$), 128.2 (2 C, $\mathbf{C_{Ar}}$), 128.1 (2 C, $\mathbf{C_{Ar}}$), 127.1 (1 C, $\mathbf{C_{Ar}}$), 126.7 (1 C, $\mathbf{C_{Ar}}$), 125.2 (2 C, $\mathbf{C_{Ar}}$), 122.9 (1 C, $\mathbf{C_{Ar}}$), 122.5 (2 C, $\mathbf{C_{Ar}}$), 71.3 (1 C, -**C**H), 55.2 (1 C, -**C**H), 34.6 (1 C, $\mathbf{C_q}$), 29.8 (3 C,-$\mathbf{C}H_3$), 16.1 (1 C, -$\mathbf{C}H_3$). *Characteristic signals for minor diastereomer:* 172.2 (1 C, -**C**OOR), 169.4 (1 C, -**C**=N), 71.1 (1 C, -**C**H), 55.3 (1 C, -**C**H), 34.6 (1 C, $\mathbf{C_q}$), 29.8 (3 C,-$\mathbf{C}H_3$), 15.8 (1 C, -$\mathbf{C}H_3$).

**HRMS** (ESI-TOF): *m/z:* $[M+H]^+$ calcd for $C_{39}H_{37}N_2O_5^+$ 613.2697 found 613.2710.

**(2*R*, 3*R*)-4-nitrophenyl 3-(3,5-di-tert-butyl-4-hydroxyphenyl)-2-((diphenylmethylene)amino)-3-phenylpropanoate (8b)**

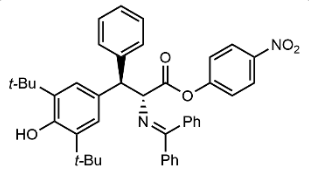


**8b**

Following general procedure F, the product was obtained as a white residue in a yield of 21.6 mg (66%) as a mixture of diastereomers (*dr* 85:15, $er_{major}$ 88:12, $er_{minor}$ 25:75).

**HPLC** (YMC-SA, n-hexane/IPA = 20/1, flow = 0.5 mL $min^{-1}$, $T_{Column}$ = 10 °C, *l* = 240 nm): $t_R$(major diastereomer): 12.54 min (minor), 15.40 min (major); $t_R$ (minor diastereomer): 13.49 min (minor), 13.98 min (major).

***r_f*** (heptanes/EtOAc = 5/1) = 0.47

$[\alpha]_D^{24}$ (c 1, $CHCl_3$) = 93

**$^{1}$H-NMR** (300 MHz, $CDCl_3$, 298 K) *δ* / ppm = 8.11 (d, *J* = 8.9 Hz, 2 H, Ar-**H**), 7.63-7.61 (m, 2 H, Ar-**H**), 7.40-7.20 (m, 10 H, Ar-**H**), 7.14 (s, 2 H, Ar-**H**), 6.68 (d, *J* = 8.9 Hz, 2 H, Ar-**H**), 6.54 (br. s, 2 H, Ar-**H**), 5.08 (s, 1 H, -O**H**), 4.89 (d, *J* = 9.1 Hz, 1 H, -C**H**), 4.84 (d, *J* = 9.1 Hz, 1 H, Ar-**H**), 1.31 (s, 18 H, -C**H₃**). Characteristic *signals for minor diastereomer:* 8.10 (d, *J* = 8.9 Hz, 2 H, Ar-**H**), 7.06 (s, 2 H, Ar-**H**), 6.60 (d, *J* = 8.9 Hz, 2 H, Ar-**H**), 5.10 (s, 1 H, -O**H**), 4.92 (d, *J* = 7.6 Hz, 1 H, -C**H**), 4.78 (d, *J* = 7.6 Hz, 1 H, -C**H**).

**$^{13}$C-NMR** (75 MHz, $CDCl_3$, 298 K) *δ* / ppm = 171.99 (1 C, -**C**OOR), 169.3 (1 C, -**C**=N), 155.6 (1 C, $\mathbf{C_{Ar}}$), 152.8 (1 C, $\mathbf{C_{Ar}}$), 145.5 (1 C, $\mathbf{C_{Ar}}$), 140.9 (1 C, $\mathbf{C_{Ar}}$), 139.3 (1 C, $\mathbf{C_{Ar}}$), 135.9 (1 C, $\mathbf{C_{Ar}}$), 135.6 (2 C, $\mathbf{C_{Ar}}$), 129.1 (2 C, $\mathbf{C_{Ar}}$), 128.8 (1 C, $\mathbf{C_{Ar}}$), 128.6 (2 C, $\mathbf{C_{Ar}}$), 128.4 (2 C, $\mathbf{C_{Ar}}$), 128.1 (2 C, $\mathbf{C_{Ar}}$), 127.9 (2 C, $\mathbf{C_{Ar}}$), 127.1 (1 C, $\mathbf{C_{Ar}}$), 126.2 (2 C, $\mathbf{C_{Ar}}$), 125.1 (2 C, $\mathbf{C_{Ar}}$), 122.5 (2 C, $\mathbf{C_{Ar}}$), 71.6 (1 C, -**C**H), 55.5 (1 C, -**C**H), 34.4 (2 C, $\mathbf{C_q}$), 30.3 (6 C, -**C**$H_3$). *Characteristic signals for minor diastereomer:* 71.1 (1 C, -**C**H), 55.8 (1 C, -**C**H), 34.4 (2 C, $\mathbf{C_q}$), 30.3 (6 C, -**C**$H_3$).

**HRMS** (ESI-TOF): *m/z:* $[M+H]^+$ calcd for $C_{42}H_{43}N_2O_5^+$ 655.3166 found 655.3170.

**(2*R*, 3*R*)-4-nitrophenyl 2-((diphenylmethylene)amino)-3-(4-hydroxy-3,5-diisopropylphenyl)-3-phenylpropanoate (8c)**

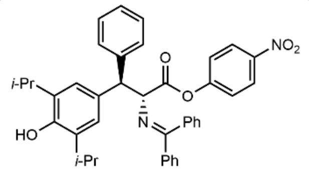


**8c**

Following general procedure F, the product was obtained as a yellow residue in a yield of 23.8 mg (78%) as a mixture of diastereomers (*dr* 75:25, $er_{major}$ 75:25, $er_{minor}$ 54:46).

**HPLC** (YMC-SB, *n*-hexane/IPA = 10/1, flow = 0.5 mL $min^{-1}$, $T_{Column}$ = 10 °C, *l* = 220 nm): $t_R$(major diastereomer): 19.36 min (major), 21.52 min (minor) ; $t_R$(minor diastereomer) = 17.50 min (major), 24.88 min (minor).

***r_f*** (heptanes/EtOAc = 5/1) = 0.31

$[\alpha]_D^{24}$ (c 1, $CHCl_3$) = 62

**$^{1}$H-NMR** (300 MHz, $CDCl_3$, 298 K) *δ* / ppm = 8.12 (d, *J* = 9.2 Hz, 2 H, Ar-**H**), 7.65-7.61 (m, 2 H, Ar-**H**), 7.40-7.30 (m, 12 H, Ar-**H**), 7.04 (s, 2 H, Ar-**H**), 6.70 (d, *J* = 9.2 Hz, 2 H, Ar-**H**), 6.56 (br. s, 1 H, Ar-**H**), 4.91 (d, *J* = 9.1 Hz, 1 H, -C**H**), 4.86 (d, *J* = 9.1 Hz, 1 H, -C**H**), 4.71 (br. s, 1 H, -O**H**), 3.06 (hept., 6.8 Hz, 2 H, -C**H**), 1.18 (d, *J* = 6.8 Hz, 6 H, -C$\mathbf{H_3}$), 1.04 (d, *J* = 6.8 Hz, 6 H, -C$\mathbf{H_3}$). *Characteristic signals for minor diastereomer:* 8.11 (d, *J* = 9.2 Hz, 2 H, Ar-**H**), 6.94 (s, 2 H, Ar-**H**), 6.66 (d, *J* = 9.2 Hz, 2 H, Ar-**H**), 4.93 (d, *J* = 7.8 Hz, 1 H, -C**H**), 4.81 (d, *J* = 7.8 Hz, 1 H, -C**H**), 1.16 (d, *J* = 6.8 Hz, 6 H, -C$\mathbf{H_3}$), 1.08 (d, *J* = 6.8 Hz, 6 H, -C$\mathbf{H_3}$).

**$^{13}$C-NMR** (75 MHz, $CDCl_3$, 298 K) *δ* / ppm = 172.0 (1 C, -**C**OOR), 169.3 (1 C, -**C**=N), 155.6 (1 C, $\mathbf{C_{Ar}}$), 149.1 (1 C, $\mathbf{C_{Ar}}$), 145.5 (1 C, $\mathbf{C_{Ar}}$), 141.0 (1 C, $\mathbf{C_{Ar}}$), 139.3 (1 C, $\mathbf{C_{Ar}}$), 135.8 (1 C, $\mathbf{C_{Ar}}$), 133.4 (2 C, $\mathbf{C_{Ar}}$), 132.0 (1 C, $\mathbf{C_{Ar}}$), 130.8 (1 C, $\mathbf{C_{Ar}}$), 129.2 (2 C, $\mathbf{C_{Ar}}$), 129.1 (2 C, $\mathbf{C_{Ar}}$), 128.8 (1 C, $\mathbf{C_{Ar}}$), 128.7 (2 C, $\mathbf{C_{Ar}}$), 128.4 (2 C, $\mathbf{C_{Ar}}$), 128.1 (2 C, $\mathbf{C_{Ar}}$), 127.9 (2 C, $\mathbf{C_{Ar}}$), 127.1 (1 C, $\mathbf{C_{Ar}}$), 125.1 (2 C, $\mathbf{C_{Ar}}$), 124.8 (2 C, $\mathbf{C_{Ar}}$), 122.5 (2 C, $\mathbf{C_{Ar}}$), 71.5 (1 C, -**C**H), 55.2 (1 C, -**C**H), 27.4 (2 C, -**C**H), 22.8 (6 C, -**C**$H_3$), 22.7 (6 C, -**C**$H_3$). *Characteristic signals for minor diastereomer:* 172.1 (1 C, -**C**OOR), 169.4 (1 C, -**C**=N), 71.0 (1 C, -**C**H), 55.5 (1 C, -**C**H), 22.8 (12 C, -**C**$H_3$).

**HRMS** (ESI-TOF): *m/z:* $[M+H]^+$ calcd for $C_{40}H_{39}N_2O_5^+$ 627.2853 found 627.2858.

### (2*R*, 3*S*)-4-nitrophenyl 3-(3,5-di-tert-butyl-4-hydroxyphenyl)-2-((diphenylmethylene)amino)-3-(*o*-tolyl)propanoate (8d)

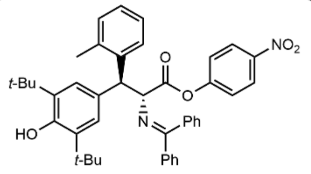


**8d**

Following general procedure F, the product was obtained as a white residue in a yield of mg 21.1 mg (63%) as a mixture of diastereomers (*dr* 85:15, $er_{major}$ 96:4, $er_{minor}$ 24:76).

**HPLC** (YMC-SA, n-hexane/IPA = 40/1, flow = 0.5 mL $min^{-1}$, $T_{Column}$ = 10 °C, *l* = 240 nm): $t_R$(major diastereomer): 15.35 min (major), 17.22 min (minor); $t_R$(minor diastereomer) = 20.55 (minor), 22.62 (major).

$\boldsymbol{r_f}$ (heptanes/EtOAc = 5/1) = 0.40

$[\boldsymbol{\alpha}]_{\boldsymbol{D}}^{\mathbf{24}}$ (c 1, $CHCl_3$) = 219

**$^{1}$H-NMR** (300 MHz, $CDCl_3$, 298 K) *δ* / ppm = 8.12 (d, *J* = 8.8 Hz, 2 H, Ar-**H**), 7.64-7.62 (m, 2 H, Ar-**H**), 7.40-7.32 (m, 7 H, Ar-**H**), 7.15-7.12 (m, 3 H, Ar-**H**), 7.04 (s, 2 H, Ar-**H**), 6.69 (d, *J* = 8.8 Hz, 2 H, Ar-**H**), 6.51 (br. s, 1 H, Ar-**H**), 5.05 (s, 1 H, -O**H**), 5.04 (d, *J* = 9.8 Hz, 1 H, -C**H**), 4.92 (d, *J* = 9.8 Hz, 2 H, -C**H**), 2.33 (s, 3 H, -C$\mathbf{H_3}$), 1.28 (s, 18 H, -C$\mathbf{H_3}$). *Characteristic signals for minor diastereomer:* 8.08 (d, *J* = 8.8 Hz, 2 H, Ar-**H**), 6.60 (d, *J* = 8.8 Hz, 2 H, Ar-**H**), 4.95 (d, *J* = 7.3 Hz, -C**H**), 2.37 (s, 3 H, -C$\mathbf{H_3}$).

**$^{13}$C-NMR** (75 MHz, $CDCl_3$, 298 K) *δ* / ppm = 171.98 (1 C, -**C**OOR), 169.2 (1 C, -**C**=N), 155.5 (1 C, $\mathbf{C_{Ar}}$), 152.5 (1 C, $\mathbf{C_{Ar}}$), 145.3 (1 C, $\mathbf{C_{Ar}}$), 139.2 (1 C, $\mathbf{C_{Ar}}$), 136.9 (1 C, $\mathbf{C_{Ar}}$), 135.3 (2 C, $\mathbf{C_{Ar}}$), 130.8 (1 C, $\mathbf{C_{Ar}}$), 130.6 (1 C, $\mathbf{C_{Ar}}$), 130.1 (1 C, $\mathbf{C_{Ar}}$), 129.5 (1 C, $\mathbf{C_{Ar}}$), 129.0 (2 C, $\mathbf{C_{Ar}}$), 128.6 (1 C, $\mathbf{C_{Ar}}$), 128.2 (2 C, $\mathbf{C_{Ar}}$), 128.0 (2 C, $\mathbf{C_{Ar}}$), 127.8 (2 C, $\mathbf{C_{Ar}}$), 127.1 (1 C, $\mathbf{C_{Ar}}$), 126.7 (1 C, $\mathbf{C_{Ar}}$), 126.3 (2 C, $\mathbf{C_{Ar}}$), 125.7 (1 C,

**C$_{Ar}$**), 125.0 (2 C, **C$_{Ar}$**), 122.3 (2 C, **C$_{Ar}$**), 71.4 (1 C, -**C**H), 50.5 (1 C, -**C**H), 34.2 (2 C, **C$_q$**), 30.2 (6 C, -**C**H$_3$), 20.0 (1 C, -**C**H$_3$).

**HRMS** (ESI-TOF): *m/z:* [M+H]$^+$ calcd for $C_{43}H_{45}N_2O_5^+$ 669.3323 found 669.3322.

**(2R, 3R)- 4-nitrophenyl 3-(3,5-di-tert-butyl-4-hydroxyphenyl)-2-((diphenylmethylene)amino)-3-(*m*-tolyl)propanoate (8e)**

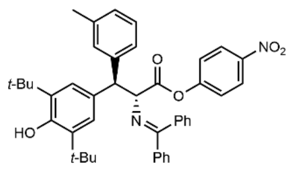


**8e**

Following general procedure F, the product was obtained as a white residue in a yield of 24.4 mg (82%) as a mixture of diastereomers (*dr* 85:15, $er_{major}$ 18:82, $er_{minor}$ 33:67).

**HPLC** (YMC-SA, n-hexane/IPA = 40/1, flow = 0.5 mL min$^{-1}$, $T_{Column}$ = 10 °C, *l* = 240 nm): $t_R$(major diastereomer): 16.24 min (minor), 23.26 min (major); $t_R$(minor diastereomer) = 19.46 (minor), 23.26 20.38 (major).

***r$_f$*** (heptanes/EtOAc = 5/1) = 0.45

$[\alpha]_D^{24}$ (c 1, CHCl$_3$) = 109

**$^1$H-NMR** (300 MHz, CDCl$_3$, 298 K) *δ* / ppm = 8.12 (d, *J* = 8.8 Hz, 2 H, Ar-**H**), 7.63-7.60 (m, 2 H, Ar-**H**), 7.42-7.30 (m, 6 H, Ar-**H**), 7.21-7.70 (m, 6 H, Ar-**H**), 6.69 (d, *J* = 8.8 Hz, 2 H, Ar-**H**), 6.53 (br. s, 1 H, Ar-**H**), 5.09 (s, 1 H, -O**H**), 4.87 (d, *J* = 9.2 Hz, 1 H, -C**H**), 4.79 (d, *J* = 9.2 Hz, 1 H, -C**H**), 2.29 (s, 3 H, -C**H$_3$**), 1.32 (s, 18 H, -C**H$_3$**). *Characteristic signals for minor diastereomer:* 8.10 (d, *J* = 8.8 Hz, 2 H, Ar-**H**), 6.60 (d, *J* = 8.8 Hz, 2 H, Ar-**H**), 5.10 (s, 1 H, -O**H**), 4.93 (d, *J* = 8.1 Hz, 1 H, -C**H**), 4.74 (d, *J* = 8.1 Hz, 1 H, -C**H**), 2.28 (s, 3 H, -C**H$_3$**).

**$^{13}$C-NMR** (75 MHz, CDCl$_3$, 298 K) *δ* / ppm = 171.9 (1 C, -**C**OOR), 169.3 (1 C, -**C**=N), 155.7 (1 C, **C$_{Ar}$**), 152.8 (1 C, **C$_{Ar}$**), 145.5 (1 C, **C$_{Ar}$**), 140.7 (1 C, **C$_{Ar}$**), 139.4 (1 C, **C$_{Ar}$**), 138.2 (1 C, **C$_{Ar}$**),135.9 (1 C, **C$_{Ar}$**), 135.6 (2 C, **C$_{Ar}$**), 130.7 (2 C, **C$_{Ar}$**), 130.3 (1 C, **C$_{Ar}$**), 129.1 (2 C, **C$_{Ar}$**), 128.8 (1 C, **C$_{Ar}$**), 128.4 (2 C, **C$_{Ar}$**), 128.4 (1 C, **C$_{Ar}$**), 128.1 (2 C, **C$_{Ar}$**), 127.9 (2 C, **C$_{Ar}$**), 127.8 (1 C, **C$_{Ar}$**), 126.3 (2 C, **C$_{Ar}$**), 125.8 (1 C, **C$_{Ar}$**), 125.1 (2 C, **C$_{Ar}$**), 122.5 (2 C, **C$_{Ar}$**), 71.6 (1 H, -**C**H), 55.5 (1 H, -**C**H), 34.4 (2 C, **C$_q$**), 30.3 (6 C, -**C**H$_3$). *Characteristic signals for minor diastereomer:* 171.9 (1 C, -**C**OOR), 169.5 (1 C, -**C**=N), 71.2 (1 C, -**C**H), 55.9 (1 C, -**C**H).

**HRMS** (ESI-TOF): *m/z:* [M+H]$^+$ calcd for $C_{43}H_{45}N_2O_5^+$ 669.3323 found 669.3384.

**(2*R*, 3*R*)-4-nitrophenyl 3-(3,5-di-tert-butyl-4-hydroxyphenyl)-2-((diphenylmethylene)amino)-3-(*p*-tolyl)propanoate (8f)**

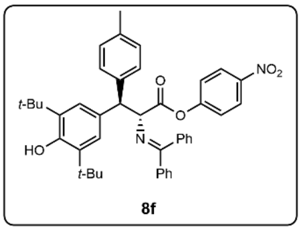

Following general procedure F, the product was obtained as a white residue in a yield of 23.1 mg (69%) as a mixture of diastereomers (*dr* 80:20, $er_{major}$ 96:4, $er_{minor}$ nd).

**HPLC** (YMC-SA, n-hexane/IPA = 40/1, flow = 0.5 mL min$^{-1}$, $T_{Column}$ = 10 °C, *l* = 240 nm): $t_R$(major diastereomer): 14.59 (minor), 19.30 (major); $t_R$(minor diastereomer) = 23.32 (both enantiomers).

$r_f$ (heptanes/EtOAc = 5/1) = 0.38

$[\alpha]_D^{24}$ (c 1, $CHCl_3$) = 112

**$^1$H-NMR** (300 MHz, $CDCl_3$, 298 K) $\delta$ / ppm = 8.12 (d, *J* = 9.0 Hz, 2 H, Ar-**H**), 7.62-7.60 (m, 2 H, Ar-**H**), 7.42-7.32 (m, 7 H, Ar-**H**), 7.23-7.18 (m, 1 H, Ar-**H**), 7.09-7.06 (m, 4 H, Ar-**H**), 6.70 (d, *J* = 9.0 Hz, 2 H, Ar-**H**), 6.54 (br. s, 1 H, Ar-**H**), 5.07 (s, 1 H, -O**H**), 4.87 (d, *J* = 9.4 Hz, 1 H, -C**H**), 4.80 (d, *J* = 9.4 Hz, 1 H, -C**H**), 2.32 (s, 3 H, -C**H$_3$**), 1.31 (s, 18 H, -C**H$_3$**). *Characteristic signals for minor diastereomer:* 8.10 (d, *J* = 9.0 Hz, 2 H, Ar-**H**), 6.60 (d, *J* = 9.0 Hz, 2 H, Ar-**H**), 5.09 (s, 1 H, -O**H**), 4.91 (d, *J* = 7.7 Hz, 1 H, -C**H**), 4.74 (d, *J* = 7.7 Hz, 1 H, -C**H**).

**$^{13}$C-NMR** (75 MHz, $CDCl_3$, 298 K) $\delta$ / ppm = 171.9 (1 C, -**C**OOR), 169.3 (1 C, -**C**=N), 155.7 (1 C, **C$_{Ar}$**), 152.7 (1 C, **C$_{Ar}$**), 145.4 (1 C, **C$_{Ar}$**), 139.4 (1 C, **C$_{Ar}$**), 138.1 (1 C, **C$_{Ar}$**), 136.5 (1 C, **C$_{Ar}$**), 135.9 (1 C, **C$_{Ar}$**), 135.5 (2 C, **C$_{Ar}$**), 130.9 (1 C, **C$_{Ar}$**), 130.7 (1 C, **C$_{Ar}$**), 129.3 (2 C, **C$_{Ar}$**), 129.1 (2 C, **C$_{Ar}$**), 129.0 (2 C, **C$_{Ar}$**), 128.8 (1 C, **C$_{Ar}$**), 128.4 (2 C, **C$_{Ar}$**), 128.1 (2 C, **C$_{Ar}$**), 127.9 (2 C, **C$_{Ar}$**), 126.2 (2 C, **C$_{Ar}$**), 125.1 (2 C, **C$_{Ar}$**), 122.5 (2 C, **C$_{Ar}$**), 71.7 (1 C, -**C**H), 55.2 (1 C, -**C**H), 34.4 (2 C, **C$_q$**), 30.3 (6 C, -**C**$H_3$), 21.2 (1 C, -**C**$H_3$). *Characteristic signals for minor diastereomer:* 169.5 (1 C, -**C**=N), 71.1 (1 C, -**C**H), 55.5 (1 C, -**C**H).

**HRMS** (ESI-TOF): *m/z:* $[M+H]^+$ calcd for $C_{43}H_{45}N_2O_5^+$ 669.3323 found 669.3326.

**(2*R*, 3*S*)-4-nitrophenyl 3-(3,5-di-tert-butyl-4-hydroxyphenyl)-2-((diphenylmethylene)amino)-3-(2-methoxyphenyl)propanoate (8g)**

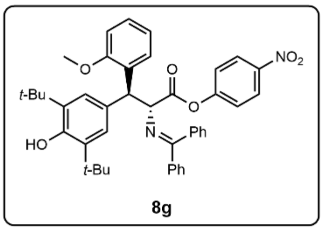

Following general procedure F, the product was obtained as a white residue in a yield of 27.0 mg (79%) as a mixture of diastereomers (*dr* 85:15, $er_{major}$ 94:6, $er_{minor}$ 62:38). Due to overlapping peaks, the diastereomers were separated by semipreparative HPLC prior to HPLC analysis with chiral stationary phase.

**Semipreparative HPLC** (Grace Alltima Silica 10µ, length 250 mm, ID 10 mm, *n*-hexane/EtOAc = 90/10): $t_R$(minor) = 7.40 min, $t_R$(major) = 8.13 min.

**HPLC** (YMC-SB, *n*-hexane/IPA = 10/1, flow = 0.5 mL min$^{-1}$, $T_{Column}$ = 10 °C, *l* = 220 nm): $t_R$(major diastereomer): 15.58 min (major), 17.50 min (minor) ; $t_R$(minor diastereomer) = 15.58 min (major), 20.67 min (minor).

$r_f$ (heptanes/EtOAc = 5/1) = 0.37

$[\alpha]_D^{24}$ (c 1, $CHCl_3$) = 143

**$^1$H-NMR** (300 MHz, $CDCl_3$, 298 K) $\delta$ / ppm = 8.10 (d, $J$ = 9.1 Hz, 2 H, Ar-**H**), 7.62-7.60 (m, 2 H, Ar-**H**), 7.44-7.28 (m, 8 H, Ar-**H**), 7.24-7.15 (m, 2 H, Ar-**H**), 6.97-6.77 (m, 2 H, Ar-**H**), 6.64 (d, $J$ = 9.1 Hz, 2 H, Ar-**H**), 6.56 (br. s, 2 H, Ar-**H**), 5.24 (d, $J$ = 8.4 Hz, 1 H, -C**H**), 5.09 (s, 1 H, -O**H**), 5.07 (d, $J$ = 8.4 Hz, 1 H, -C**H**), 3.67 (s, 3 H, -OC**H₃**), 1.34 (s, 18 H, -C**H₃**). *Characteristic signals for the minor diastereomer:* 8.10 (d, $J$ = 9.1 Hz, 2 H, Ar-**H**), 7.59-7.55 (m, 2 H, Ar-**H**), 6.59 (d, $J$ = 9.1 Hz, 2 H, Ar-**H**), 5.29 (d, $J$ = 7.9 Hz, 1 H, -C**H**), 5.11 (d, $J$ = 7.9 Hz, 1 H, -C**H**), 5.09 (s, 1 H, -O**H**), 3.67 (s, 3 H, -OC**H₃**), 1.32 (s, 18 H, -C**H₃**).

**$^{13}$C-NMR** (75 MHz, $CDCl_3$, 298 K) $\delta$ / ppm = 171.7 (1 C, -**C**OOR), 169.7 (1 C, -**C**=N), 157.0 (1 C, **C$_{Ar}$**), 155.9 (1 C, **C$_{Ar}$**), 152.7 (1 C, **C$_{Ar}$**), 145.4 (1 C, **C$_{Ar}$**), 139.5 (1 C, **C$_{Ar}$**), 136.1 (1 C, **C$_{Ar}$**), 135.4 (2 C, **C$_{Ar}$**), 130.6 (1 C, **C$_{Ar}$**), 130.5 (1 C, **C$_{Ar}$**), 130.2 (1 C, **C$_{Ar}$**), 129.3 (1 C, **C$_{Ar}$**), 129.1 (2 C, **C$_{Ar}$**), 128.6 (1 C, **C$_{Ar}$**),128.3 (2 C, **C$_{Ar}$**), 128.0 (6 C, **C$_{Ar}$**), 126.8 (2 C, **C$_{Ar}$**), 125.1 (2 C, **C$_{Ar}$**), 122.5 (2 C, **C$_{Ar}$**), 120.3 (1 C, **C$_{Ar}$**), 69.9 (1 C, -**C**H), 55.3 (1 C, -**C**H), 48.7 (1 C, -**C**OMe), 34.5 (2 C, **C$_q$**), 30.4 (6 C, -**C**$H_3$). *Characteristic signals for the minor diastereomer:* 171.6 (1 C, -**C**OOR), 70.2 (1 C, -**C**H), 55.5 (1 C, -**C**H), 34.4 (1 C, **C$_q$**), 30.3 (6 C, -**C**$H_3$).

**HRMS** (ESI-TOF): *m/z:* $[M+H]^+$ calcd for $C_{43}H_{45}N_2O_6^+$ 685.3272 found 685.3276.

**(2*R*, 3*R*)-4-nitrophenyl 3-(3,5-di-tert-butyl-4-hydroxyphenyl)-2-((diphenylmethylene)amino)-3-(4-methoxyphenyl)propanoate (8h)**

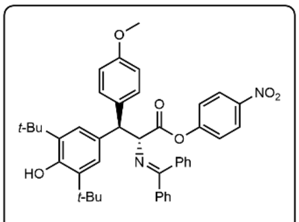


**8h**

Following general procedure F, the product was obtained as a white residue in a yield of 32.5 mg (95%) as a mixture of diastereomers (*dr* 80:20, *er$_{major}$* 94:6, *er$_{minor}$* 40:60). Due to overlapping peaks, the diastereomers were separated by semipreparative HPLC prior to analysis with HPLC with chiral stationary phase.

**Semipreparative HPLC** (Grace Alltima Silica 10µ, length 250 mm, ID 10 mm, *n*-hexane/EtOAc = 95/5): $t_R$(minor) = 15.08 min, $t_R$(major) = 15.93 min.

**HPLC** (YMC-SB, *n*-hexane/IPA = 10/1, flow = 0.5 mL min$^{-1}$, $T_{Column}$ = 10 °C, *l* = 220 nm): $t_R$(major diastereomer): 16.06 min (major), 21.00 min (minor) ; $t_R$(minor diastereomer) = 16.69 min (major), 21.44 min (minor).

$r_f$ (heptanes/EtOAc = 5/1) = 0.31

$[\alpha]_D^{24}$ (c 1, $CHCl_3$) = 118

**$^1$H-NMR** (300 MHz, $CDCl_3$, 298 K) $\delta$ / ppm = 8.13 (d, $J$ = 9.1 Hz, 2 H, Ar-**H**), 7.63-7.60 (m, 2 H, Ar-**H**), 7.41-7.32 (m, 16 H, Ar-**H**), 7.41-7.23 (m, 9 H, Ar-**H**), 7.13 (s, 2 H, Ar-**H**), 6.84 (d, $J$ = 8.7 Hz, 2 H, Ar-**H**), 6.73 (d, $J$ = 9.1 Hz, 2 H, Ar-**H**), 6.54 (br. s, 1 H, Ar-**H**), 5.07 (s, 1 H, -O**H**), 4.84 (d, $J$ = 9.3 Hz, 1 H, -

C**H**), 4.79 (d, $J$ = 9.3 Hz, 1 H, -C**H**), 3.78 (s, 3 H, -OC**H$_3$**), 1.31 (s, 18 H, -C**H$_3$**). *Characteristic signals for minor diastereomer:* 8.11 (d, $J$ = 9.1 Hz, 2 H, Ar-**H**), 7.04 (s, 2 H, Ar-**H**), 6.63 (d, $J$ = 9.1 Hz, 2 H, Ar-**H**), 5.09 (s, 1 H, -O**H**), 4.88 (d, $J$ = 7.6 Hz, 1 H, -C**H**), 4.73 (d, $J$ = 7.6 Hz, 1 H, -C**H**), 3.79 (s, 3 H, -OC**H$_3$**).

**$^{13}$C-NMR** (75 MHz, CDCl$_3$, 298 K) $\delta$ / ppm = 171.9 (1 C, -**C**OOR), 169.3 (1 C, -**C**=N), 158.6 (1 C, **C$_{Ar}$**), 155.6 (1 C, **C$_{Ar}$**), 152.7 (1 C, **C$_{Ar}$**), 145.5 (1 C, **C$_{Ar}$**), 139.3 (1 C, **C$_{Ar}$**), 135.8 (1 C, **C$_{Ar}$**), 135.5 (2 C, **C$_{Ar}$**), 132.9 (1 C, **C$_{Ar}$**), 131.0 (1 C, **C$_{Ar}$**), 130.8 (1 C, **C$_{Ar}$**), 130. 1 (2 C, **C$_{Ar}$**), 129.1 (2 C, **C$_{Ar}$**), 128.8 (1 C, **C$_{Ar}$**), 128.4 (2 C, **C$_{Ar}$**), 128.1 (2 C, **C$_{Ar}$**), 127.9 (2 C, **C$_{Ar}$**), 126.2 (2 C, **C$_{Ar}$**), 125.2 (2 C, **C$_{Ar}$**), 122.5 (2 C, **C$_{Ar}$**), 114.0 (2 C, **C$_{Ar}$**), 71.8 (1 C, -**C**H), 55.4 (1 C, -O**C**H$_3$), 54.7 (1 C, -**C**H), 34.4 (2 C, **C$_q$**), 30.4 (6 C, -**C**H$_3$). *Characteristic signals for minor diastereomer:* 171.9 (1 C, -**C**OOR), 169.5 (1 C, -**C**=N), 71.2 (1 C, -**C**H), 55.0 (1 C, -**C**H), 34.4 (2 C, **C$_q$**), 30.3 (6 C, -**C**H$_3$).

**HRMS** (ESI-TOF): *m/z:* [M+H]$^+$ calcd for $C_{43}H_{45}N_2O_6^+$ 685.3272 found 685.3275

**(2*R*, 3*R*)-4-nitrophenyl 3-(3,5-di-tert-butyl-4-hydroxyphenyl)-3-(4-(dimethylamino)phenyl)-2-((diphenylmethylene)amino)propanoate (8i)**

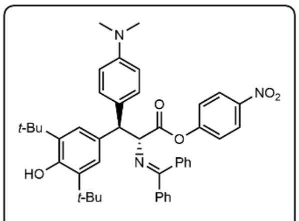


8i

Product **8i** was synthesized according to general procedure F with slight adaptions. Instead of ACN (no conversion was observed in ACN even at room temperature), dry, degassed DCM was used as solvent and the reaction time was increased to 64 h. Product **8i** was obtained as an orange oily residue in a yield of 22.3 mg (64%, 84% conversion) as a mixture of diastereomers (*dr* 75:25, $er_{major}$ 95:5, $er_{minor}$ 64:36) Due to overlapping peaks, the diastereomers were separated by semipreparative HPLC prior to HPLC analysis with chiral stationary phase.

**Semipreparative HPLC** (Grace Alltima Silica 10µ, length 250 mm, ID 10 mm, *n*-hexane/EtOAc = /10): $t_R$(major) = 10.61 min, $t_R$(minor) = 11.66 min.

**HPLC** (YMC-SB, *n*-hexane/IPA = 20/1, flow = 0.5 mL min$^{-1}$, $T_{Column}$ = 10 °C, $l$ = 250 nm): $t_R$(major diastereomer): 16.91 min (major), 22.02 min (minor) ; $t_R$(minor diastereomer) = 16.62 min (major), 21.84 min (minor).

$r_f$ (heptanes/EtOAc = 5/1) = 0.32

$[\alpha]_D^{24}$ (c 1, CHCl$_3$) = 63

**$^1$H-NMR** (300 MHz, CDCl$_3$, 298 K) $\delta$ / ppm = 8.11 (d, $J$ = 9.1 Hz, 2 H, Ar-**H**), 7.65-7.60 (m, 2 H, Ar-**H**), 7.41-7.31 (m, 7 H, Ar-**H**), 7.20 (d, $J$ = 8.6 Hz, 2 H, Ar-**H**), 7.14 (s, 2 H, Ar-**H**), 6.72 (d, $J$ = 9.1 Hz, 2 H, Ar-**H**), 6.68 (d, $J$ = 8.6 Hz, 2 H, Ar-**H**), 6.56 (br. s, 1 H, Ar-**H**), 5.04 (s, 1 H, -O**H**), 4.84 (d, $J$ = 9.5 Hz, 1 H, -C**H**), 4.74 (d, $J$ = 9.5 Hz, 1 H, -C**H**), 2.91 (s, 6 H, -NC**H$_3$**), 1.31 (s, 18 H, -C**H$_3$**). *Characteristic signals for minor diastereomer:* 8.09 (d, $J$ = 9.1 Hz, 2 H, Ar-**H**), 7.07 (s, 2 H, Ar-**H**), 6.60 (d, $J$ = 9.1 Hz, 2 H, Ar-**H**), 5.06 (s, 1 H, -O**H**), 4.88 (d, $J$ = 7.7 Hz, 1 H, -C**H**), 4.67 (d, $J$ = 7.7 Hz, 1 H, -C**H**), 2.92 (s, 6 H, -NC**H$_3$**).

**$^{13}$C-NMR** (75 MHz, $CDCl_3$, 298 K) $\delta$ / ppm = 171.6 (1 C, -**C**OOR), 169.5 (1 C, -**C**=N), 155.8 (1 C, $\mathbf{C_{Ar}}$), 152.6 (1 C, $\mathbf{C_{Ar}}$), 149.8(1 C, $\mathbf{C_{Ar}}$), 145.4 (1 C, $\mathbf{C_{Ar}}$), 139.5 (1 C, $\mathbf{C_{Ar}}$), 136.0 (1 C, $\mathbf{C_{Ar}}$), 135.4 (2 C, $\mathbf{C_{Ar}}$), 131.4 (1 C, $\mathbf{C_{Ar}}$), 130.6 (1 C, $\mathbf{C_{Ar}}$), 130.1 (1 C, $\mathbf{C_{Ar}}$), 129.8 (2 C, $\mathbf{C_{Ar}}$), 129.1 (2 C, $\mathbf{C_{Ar}}$), 128.7 (1 C, $\mathbf{C_{Ar}}$), 128.4 (2 C, $\mathbf{C_{Ar}}$), 128.1 (2 C, $\mathbf{C_{Ar}}$),128.0 (2 C, $\mathbf{C_{Ar}}$), 126.1 (2 C, $\mathbf{C_{Ar}}$), 125.1 (2 C, $\mathbf{C_{Ar}}$), 122.7 (2 C, $\mathbf{C_{Ar}}$), 112.9 (2 C, $\mathbf{C_{Ar}}$), 72.1 (1 C, -**C**H), 54.9 (1 C, -**C**H), 40.8 (2 C, -N**C**$H_3$), 34.4 (2 C, $\mathbf{C_q}$), 30.4 (6 C, -**C**$H_3$). *Characteristic signals for minor diastereomer:* 171.7 (1 C, -**C**OOR), 169.7 (1 C, -**C**=N), 71.5 (1 C, -**C**H), 55.2 (1 C, -**C**H), 40.9 (2 C, -N**C**$H_3$), 34.4 (2 C, $\mathbf{C_q}$), 30.3 (6 C, -**C**$H_3$).

**HRMS** (ESI-TOF): *m/z:* $[M+H]^+$ calcd for $C_{44}H_{48}N_3O_5^+$ 698.3588 found 698.3580.

### (2*R*, 3*R*)-4-nitrophenyl 3-(4-(tert-butyl)phenyl)-3-(3,5-di-tert-butyl-4-hydroxyphenyl)-2-((diphenylmethylene)amino)propanoate (8j)

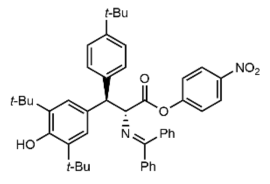

**8j**

Following general procedure F, the product was obtained as a colourless oil in a yield of 26.3 mg (74%) as a mixture of diastereomers. For HPLC analysis, and determination of enantiomeric ratio, derivatization to the morpholine amide was necessary.

***$r_f$*** (heptanes/EtOAc = 5/1) = 0.45

$[\alpha]_D^{24}$ (c 1, $CHCl_3$) = 122

**$^{1}$H-NMR** (300 MHz, $CDCl_3$, 298 K) $\delta$ / ppm = 8.08 (d, *J* = 9.1 Hz, 2 H, Ar-**H**), 7.65-7.58 (m, 2 H, Ar-**H**), 7.43-7.28 (m, 10 H, Ar-**H**), 7.15 (s, 2 H, Ar-**H**), 6.60-6.51 (br. 2 H, Ar-H), 6.58 (d, *J* = 9.1 Hz, 2 H, Ar-**H**), 5.06 (1 H, -O**H**), 4.88 (d, *J* = 9.4 Hz, 1 H, -C**H**), 4.78 (d, *J* = 9.4 Hz, 1 H, -C**H**), 1.31 (s, 18 H, -C$\mathbf{H_3}$), 1.30 (s, 9 H, -C$\mathbf{H_3}$). *Characteristic signals for minor diastereomer:* 7.09 (s, 2 H, Ar-**H**), 6.52 (d, *J* = 9.1 Hz, 2 H, Ar-**H**), 5.10 (s, 1 H, -O**H**), 4.71 (d, *J* = 7.8 Hz, 1 H, -C**H**).

**$^{13}$C-NMR** (75 MHz, $CDCl_3$, 298 K) $\delta$ / ppm = 171.6 (1 C, -**C**OOR), 169.2 (1 C, -**C**=N), 155.4 (1 C, $\mathbf{C_{Ar}}$), 152.5 (1 C, $\mathbf{C_{Ar}}$), 149.8 (1 C, $\mathbf{C_{Ar}}$), 145.2 (1 C, $\mathbf{C_{Ar}}$), 139.2 (1 C, $\mathbf{C_{Ar}}$), 137.6 (1 C, $\mathbf{C_{Ar}}$), 135.7 (1 C, $\mathbf{C_{Ar}}$), 135.4 (2 C, $\mathbf{C_{Ar}}$), 130.6 (1 C, $\mathbf{C_{Ar}}$), 130.5 (1 C, $\mathbf{C_{Ar}}$), 130.0 (1 C, $\mathbf{C_{Ar}}$), 129.0 (2 C, $\mathbf{C_{Ar}}$), 128.7 (2 C, $\mathbf{C_{Ar}}$), 128.3 (2 C, $\mathbf{C_{Ar}}$), 127.9 (2 C, $\mathbf{C_{Ar}}$), 127.8 (2 C, $\mathbf{C_{Ar}}$), 125.9 (2 C, $\mathbf{C_{Ar}}$), 125.3 (2 C, $\mathbf{C_{Ar}}$), 124.9 (2 C, $\mathbf{C_{Ar}}$), 122.4 (2 C, $\mathbf{C_{Ar}}$), 72.0 (1 C, -**C**H), 55.7 (1 C, -**C**H), 34.8 (1 C, $\mathbf{C_q}$), 34.6 (2 C, $\mathbf{C_q}$), 31.7 (3 C, -**C**$H_3$), 30.5 (6 C, -**C**$H_3$). *Characteristic signals for minor diastereomer:* 171.4 (1 C, -**C**OOR), 169.3 (1 C, -**C**=N), 71.5 (1 C, -**C**H), 55.6 (1 C, -**C**H), 34.7 (1 C, $\mathbf{C_q}$), 34.5 (2 C, $\mathbf{C_q}$), 31.7 (3 C, -**C**$H_3$), 30.5 (6 C, -**C**$H_3$).

**HRMS** (ESI-TOF): *m/z:* $[M+H]^+$ calcd for $C_{46}H_{51}N_2O_5^+$ 711.3792 found 711.3796.

**Morpholine amide 9j**

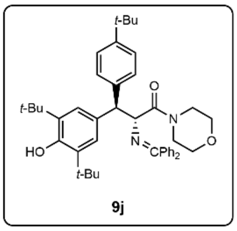


Morpholine amide **9j** was synthesized in order to determine the enantiomeric ratio of product **8j**. The product was obtained as a white residue as a mixture of diastereomers (*dr* 80:20, $er_{major}$ 95:5, $er_{minor}$ nd).

**HPLC** (YMC-SA, *n*-hexane/IPA = 20/1, flow = 0.5 mL min$^{-1}$, $T_{Column}$ = 10 °C, *l* = 240 nm): $t_R$(major diastereomer): 27.07 min (major), 40.35 min (minor) ; $t_R$(minor diastereomer) = 21.33 (both enantiomers).

***r_f*** (heptanes/EtOAc = 2/1) = 0.31

$[\alpha]_D^{24}$ (c 1, $CHCl_3$) = 120

**$^1$H-NMR** (300 MHz, $CDCl_3$, 298 K) *δ* / ppm = 7.47-7.33 (m, 7 H, Ar-**H**), 7.24-7.19 (m, 5 H, Ar-**H**), 7.06 (2, 2 H, Ar-**H**), 6.62 (d, *J* = 6.1 Hz, 2 H, Ar-**H**), 5.01 (s, 1 H, -O**H**), 4.88 (d, *J* = 10.3 Hz, 1 H, -C**H**), 4.84 (d, *J* = 10.3 Hz, 1 H, -C**H**), 3.52-2.90 (br. 8 H, -C**H₂**), 1.32 (s, 18 H, -C**H₃**), 1.26 (s, 18 H, -C**H₃**). *Characteristic signals for minor diastereomer:* 6.83 (d, *J* 6.1 Hz, 2 H, Ar-**H**), 5.03 (s, 1 H, -O**H**), 4.89 (d, *J* = 9.5 Hz, 1 H, -C**H**), 9.51 (d, *J* = 9.5 Hz, 1 H, -C**H**), 1.34 (s, 18 H, -C**H₃**), 1.27 (s, 9 H, -C**H₃**).

**$^{13}$C-NMR** (75 MHz, $CDCl_3$, 298 K) *δ* / ppm = 170.1 (1 C, -**C**ONR$_2$), 169.0 (1 C, -**C**=N), 152.4 (1 C, **C$_{Ar}$**), 149.6 (1 C, **C$_{Ar}$**), 139.6 (1 C, **C$_{Ar}$**), 137.8 (1 C, **C$_{Ar}$**), 137.0 (1 C, **C$_{Ar}$**), 135.4 (2 C, **C$_{Ar}$**), 131.7 (1 C, **C$_{Ar}$**), 130.3 (1 C, **C$_{Ar}$**), 129.0 (2 C, **C$_{Ar}$**), 128.9 (2 C, **C$_{Ar}$**), 128.7 (1 C, **C$_{Ar}$**), 128.4 (2 C, **C$_{Ar}$**), 128.0 (2 C, **C$_{Ar}$**), 127.9 (2 C, **C$_{Ar}$**), 126.3 (2 C, **C$_{Ar}$**), 125.3 (2 C, **C$_{Ar}$**), 68.6 (1 C, -**C**H), 66.8 (1 C, -C**H$_2$**), 66.4 (1 C, -**C**H$_2$), 56.0 (1 C, -**C**H), 45.8 (1 C, -C**H$_2$**), 42.4 (1 C, -C**H$_2$**), 34.4 (3 C, **C$_q$**), 31.5 (3 C, -**C**H$_3$), 30.4 (6 C, -**C**H$_3$). *Characteristic signals for minor diastereomer:* 170.3 (1 C, -**C**ONR$_2$), 69.3 (1 C, -**C**H), 56.5 (1 C, -C**H**), 34.5 (3 C, **C$_q$**), 31.5 (3 C, -**C**H$_3$), 30.5 (6 C, -**C**H$_3$).

**HRMS** (ESI-TOF): *m/z:* $[M+H]^+$ calcd for $C_{44}H_{55}N_2O_3^+$ 659.4207 found 659.4214

**(2*R*, 3*R*)-4-nitrophenyl 3-(3,5-di-tert-butyl-4-hydroxyphenyl)-2-((diphenylmethylene)amino)-3-(4-(trifluoromethyl)phenyl)propanoate (8k)**

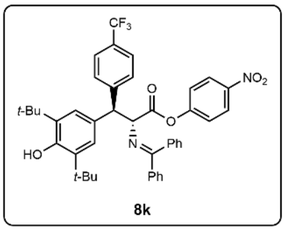


Following general procedure F, the product was obtained as a colourless oil in a yield of 28.2 mg (78%) as a mixture of diastereomers. For HPLC analysis, and determination of enantiomeric ratio, derivatization to the morpholine amide was necessary.

***r_f*** (heptanes/EtOAc = 5/1) = 0.45

$[\alpha]_D^{24}$ (c 1, $CHCl_3$) = 68

**$^1$H-NMR** (300 MHz, $CDCl_3$, 298 K) *δ* / ppm = 8.13 (d, *J* = 9.1 Hz, 2 H, Ar-**H**), 7.72-7.68 (m, 1 H, Ar-**H**), 7.62-7.54 (m, 4 H, Ar-**H**), 7.44-7.32 (m, 8 H, Ar-**H**), 7.16 (s, 2 H, Ar-**H**), 6.71 (d, *J* = 9.1 Hz, 2 H, Ar-**H**), 6.56 (br. s, 1 H, Ar-**H**), 4.90 (s, 2 H, 2x -C**H**), 1.32 (2, 18 H, -C**H₃**). *Characteristic signals for minor*

*diastereomer:* 8.11 (d, *J* = 9.1 Hz, 2 H, Ar-**H**), 7.01 (s, 2 H, Ar-**H**), 6.65 (d, *J* = 9.1 Hz, 2 H, Ar-**H**), 5.14 (s, 1 H, -O**H**), 4.93 (d, *J* = 7.4 Hz, 1 H, -C**H**), 4.84 (d, *J* = 7.4 Hz, 1 H, -C**H**), 1.31 (18 H, -C**H₃**).

**$^{13}$C-NMR** (75 MHz, $CDCl_3$, 298 K) $\delta$ / ppm = 172.1 (1 C, -**C**OOR), 168.6 (1 C, -**C**=N), 155.1 (1 C, $\mathbf{C_{Ar}}$), 152.8 (1 C, $\mathbf{C_{Ar}}$), 145.3 (1 C, $\mathbf{C_{Ar}}$), 138.9 (1 C, $\mathbf{C_{Ar}}$), 135.7 (2 C, $\mathbf{C_{Ar}}$), 135.5 (1 C, $\mathbf{C_{Ar}}$), 130.7 (1 C, $\mathbf{C_{Ar}}$), 130.0 (1 C, $\mathbf{C_{Ar}}$), 129.6 (2 C, $\mathbf{C_{Ar}}$), 129.2 (2 C, $\mathbf{C_{Ar}}$), 128.9 (2 C, $\mathbf{C_{Ar}}$), 128.8 (1 C, $\mathbf{C_{Ar}}$), 128.4 (2 C, $\mathbf{C_{Ar}}$), 128.1 (1 C, $\mathbf{C_{Ar}}$), 128.0 (2 C, $\mathbf{C_{Ar}}$), 127.6 (2 C, $\mathbf{C_{Ar}}$), 126.1 (2 C, $\mathbf{C_{Ar}}$), 125.7 (1 C, $\mathbf{C_{Ar}}$), 125.3 (1 C,-**C**$F_3$), 125.1 (2 C, $\mathbf{C_{Ar}}$), 122.2 (2 C, $\mathbf{C_{Ar}}$), 71.0 (1 C, -**C**H), 55.2 (1 C, -**C**H), 34.6 (2 C, $\mathbf{C_q}$), 30.5 (6 C, -**C**$H_3$). *Characteristic signals for minor diastereomer:* 172.0 (1 C, -**C**OOR), 168.8 (1 C, -**C**=N), 70.7 (1 C, -**C**H), 55.4 (1 C, -**C**H), 34.6 (2 C, $\mathbf{C_q}$), 30.4 (6 C, -**C**$H_3$).

**$^{19}$F-NMR** (282 MHz, $CDCl_3$, 298 K) $\delta$ / ppm = -62.38 (3 F, minor), -62.43 (3 F, major).

**HRMS** (ESI-TOF): *m/z:* $[M+H]^+$ calcd for $C_{43}H_{42}F_3N_2O_5^+$ 723.3040 found 723.3044.

**Morpholine amide 9k**

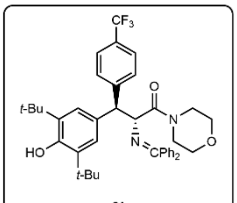

**9k**

Morpholine amide **9k** was synthesized in order to determine the enantiomeric ratio of product **8k**. The product was obtained as a white residue as a mixture of diastereomers (*dr* 65:35, $er_{major}$ 89:11, $er_{minor}$ 21:79).

**HPLC** (YMC-SA, *n*-hexane/IPA = 20/1, flow = 0.5 mL min$^{-1}$, $T_{Column}$ = 10 °C, *l* = 272 nm): $t_R$(major diastereomer): 21.93 min (minor), 34.77 min (major) ; $t_R$(minor diastereomer) = 27.80 min (minor), 29.86 min (major).

$r_f$ (heptanes/EtOAc = 2/1) = 0.31

$[\alpha]_D^{24}$ (c 1, $CHCl_3$) = (c 1, $CHCl_3$) = 99

**$^1$H-NMR** (300 MHz, $CDCl_3$, 298 K) $\delta$ / ppm = 7.44-7.22 (m, 13 H, Ar-**H**), 6.97 (s, 2 H, Ar-**H**), 6.48-6.50 (br. 1 H, Ar-**H**), 5.07 (1 H, -O**H**), 4.96-4.88 (m, 2 H, 2x -C**H**), 3.44-2.64 (br. 8 H, -C**H₂**), 1.27 (s, 18 H, -C**H₃**). *Characteristic signals for minor diastereomer:* 7.00 (s, 2 H, Ar-**H**), 6.91-6.94 (m, 1 H, Ar-**H**), 5.09 (s, 1 H, -O**H**), 1.34 (s, 18 H, -C**H₃**).**$^{13}$C-NMR** (75 MHz, $CDCl_3$, 298 K) $\delta$ / ppm = 170.0 (1 C, -**C**O$NR_2$), 169.4 (1 C, -**C**=N), 152.8 (1 C, $\mathbf{C_{Ar}}$), 145.3 (1 C, $\mathbf{C_{Ar}}$), 139.4 (1 C, $\mathbf{C_{Ar}}$), 136.3 (1 C, $\mathbf{C_{Ar}}$), 135.7 (2 C, $\mathbf{C_{Ar}}$), 130.6 (1 C, $\mathbf{C_{Ar}}$), 130.6 (1 C, $\mathbf{C_{Ar}}$), 129.5 (2 C, $\mathbf{C_{Ar}}$), 129.0 (2 C, $\mathbf{C_{Ar}}$), 128.9 (1 C, $\mathbf{C_{Ar}}$), 128.7 (1 C, $\mathbf{C_{Ar}}$), 128.4 (2 C, $\mathbf{C_{Ar}}$), 128.1 (2 C, $\mathbf{C_{Ar}}$), 127.9 (2 C, $\mathbf{C_{Ar}}$), 126.3 (2 C, $\mathbf{C_{Ar}}$), 125.7 (1 C, $\mathbf{C_{Ar}}$), 125.3 (1 C, $\mathbf{C_{Ar}}$), 125.3 (1 C, -**C**$F_3$), 69.4 (1 C, -**C**H), 66.7 (1 C, -**C**$H_2$), 66.9 (1 C, -**C**$H_2$), 55.7 (1 C, -**C**H), 45.9 (1 C, -**C**$H_2$), 42.7 (1 C, -**C**$H_2$), 34.4 (2 C, $\mathbf{C_q}$), 30.3 (6 C, -**C**$H_3$). *Characteristic signals for minor diastereomer:* 169.6 (1 C, -**C**$NR_2$), 169.5 (1 C, -**C**=N), 67.6 (1 C, -**C**H), 56.6 (1 C, -**C**H), 34.4 (2 C, $\mathbf{C_q}$), 30.4 (6 C, -**C**$H_3$).

**$^{19}$F-NMR** (282 MHz, $CDCl_3$, 298 K) $\delta$ / ppm = -62.32 (3 F, minor), -62.44 (3 F, major).

**HRMS** (ESI-TOF): *m/z:* $[M+H]^+$ calcd for $C_{41}H_{46}F_3N_2O_3^+$ 671.3455 found 671.3450

**(2*R*, 3*S*)-4-nitrophenyl 3-(3,5-di-tert-butyl-4-hydroxyphenyl)-2-((diphenylmethylene)amino)-3-(4-fluorophenyl)propanoate (8l)**

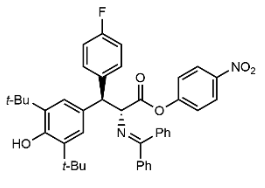

**8l**

Following general procedure F, the product was obtained as a colourless oil in a yield of 24.9 mg (74%) as a mixture of diastereomers. For HPLC analysis, and determination of enantiomeric ratio, derivatization to the morpholine amide was necessary.

***r*$_f$** (heptanes/EtOAc = 5/1) = 0.44

$[\alpha]_D^{24}$ (c 1, $CHCl_3$) = 118

**$^1$H-NMR** (300 MHz, $CDCl_3$, 298 K) $\delta$ / ppm = 8.14 (d, *J* = 8.9 Hz, 2 H, Ar-**H**), 7.62-7.60 (m, 2 H, Ar-**H**), 7.42-7.28 (m, 9 H, Ar-**H**), 7.13-6.97 (m, 4 H, Ar-**H**), 6.74 (d, *J* = 8.9 Hz, 2 H, Ar-**H**), 5.11 (s, 1 H, -O**H**), 4.89-4.67 (m, 2 H, -C**H**), 1.31 (s, 18 H, -C**H$_3$**). *Characteristic signals for minor diastereomer:* 8.12 (d, *J* = 8.9 Hz, 2 H, Ar-**H**), 6.65 (d, *J* = 8.9 Hz, 2 H, Ar-**H**), 6.54 (br. s, 1 H, Ar-**H**), 5.11 (s, 1 H, -O**H**).

**$^{13}$C-NMR** (75 MHz, $CDCl_3$, 298 K) $\delta$ / ppm = 172.2 (1 C, -**C**OOR), 169.1 (1 C, -**C**=N), 161.9 (d, $J_{C\text{-}F}$ = 246 Hz, 1 C, **C$_{Ar}$**-F), 155.5 (1 C, **C$_{Ar}$**), 152.9 (1 C, **C$_{Ar}$**), 145.5 (1 C, **C$_{Ar}$**), 139.2 (1 C, **C$_{Ar}$**), 135.8 (1 C, **C$_{Ar}$**), 135.7 (2 C, **C$_{Ar}$**), 130.9 (1 C, **C$_{Ar}$**), 130.6 (1 C, **C$_{Ar}$**), 130.5 (1 C, **C$_{Ar}$**), 130.2 (1 C, **C$_{Ar}$**), 129.1 (2 C, **C$_{Ar}$**), 128.8 (1 C, **C$_{Ar}$**), 128.5 (2 C, **C$_{Ar}$**), 128.4 (1 C, **C$_{Ar}$**), 128.2 (2 C, **C$_{Ar}$**), 127.8 (2 C, **C$_{Ar}$**), 126.2 (2 C, **C$_{Ar}$**), 125.2 (2 C, **C$_{Ar}$**), 122.4 (2 C, **C$_{Ar}$**), 115.6 (1 C, **C$_{Ar}$**), 115.3 (1 C, **C$_{Ar}$**), 71.5 (1 C, -**C**H), 54.5 (1 C, -**C**H), 34.4 (2 C, **C$_q$**), 30.3 (6 C, -**C**$H_3$). *Characteristic signals for minor diastereomer:* 172.2 (1 C, -**C**OOR), 169.3 (1 C, -**C**=N), 70.9 (1 C, -**C**H), 54.8 (1 C, -**C**H), 34.4 (2 C, **C$_q$**), 30.3 (6 C, -**C**$H_3$).

**$^{19}$F-NMR** (282 MHz, $CDCl_3$, 298 K) $\delta$ / ppm = -115.6 (1 F, Ar-**F**, major), -116.2 (1 F, Ar-**F**, minor).

**HRMS** (ESI-TOF): *m/z:* $[M+H]^+$ calcd for $C_{42}H_{42}FN_2O_5^+$ 673.3072 found 673.3061.

**Morpholine amide 9l**

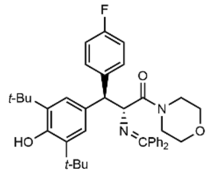

**9l**

Morpholine amide **9l** was synthesized in order to determine the enantiomeric ratio of product **8l**. The product was obtained as a white residue as a mixture of diastereomers (*dr* 80:20, $er_{major}$ 88:12, $er_{minor}$ nd.)

**HPLC** (YMC-SA, *n*-hexane/IPA = 20/1, flow = 0.5 mL min$^{-1}$, $T_{Column}$ = 10 °C, *l* = 272 nm): $t_R$(major diastereomer): 26.29 min (minor), 39.97 min (major) ; $t_R$(minor diastereomer) = 35 min (both enantiomers).

***r*$_f$** (heptanes/EtOAc = 2/1) = 0.31

$[\alpha]_D^{24}$ (c 1, $CHCl_3$) = 78

**$^1$H-NMR** (300 MHz, $CDCl_3$, 298 K) $\delta$ / ppm = 7.52-7.29 (m, 8 H, Ar-**H**), 7.25-7.22 (m, 2 H, Ar-**H**), 7.01-6.93 (m, 5 H, Ar-**H**), 6.48 (br. s, 1 H, Ar-**H**), 5.05 (s, 1 H, -O**H**), 4.89-4.82 (m, 2 H, -C**H**), 3.45-3.20 (br. 8 H, -C**H$_2$**), 1.28 (s, 18 H, -C**H$_3$**). *Characteristic signals for minor diastereomer:* 5.07 (1 H, -O**H**).

**$^{13}$C-NMR** (75 MHz, $CDCl_3$, 298 K) $\delta$ / ppm = 169.6 (1 C, -**C**ONR$_2$), 169.2 (1 C, -**C**=N), 152.6 (1 C, **C$_{Ar}$**), 139.4 (1 C, **C$_{Ar}$**), 136.4 (1 C, **C$_{Ar}$**), 135.5 (2 C, **C$_{Ar}$**), 131.3 (1 C, **C$_{Ar}$**), 130.7 (1 C, **C$_{Ar}$**), 130.6 (1 C, **C$_{Ar}$**), 130.5 (1 C, **C$_{Ar}$**), 129.0 (2 C, **C$_{Ar}$**), 128.9 (1 C, **C$_{Ar}$**), 128.8 (1 C, **C$_{Ar}$**), 128.4 (2 C, **C$_{Ar}$**), 128.0 (2 C, **C$_{Ar}$**), 127.9 (2 C, **C$_{Ar}$**), 126.2 (2 C, **C$_{Ar}$**), 115.3 (1 C, **C$_{Ar}$**), 115.0 (1 C, **C$_{Ar}$**), 69.5 (1 C, -**C**H), 66.9 (1 C, **C**H$_2$), 66.6 (1 C, -**C**H$_2$), 55.1 (1 C, -**C**H), 46.0 (1 C, -**C**H$_2$), 42.6 (1 C, -**C**H$_2$), 34.4 (2 C, **C$_q$**), 30.3 (6 C, -**C**H$_3$). *Characteristic signals for minor diastereomer:* 55.9 (1 C, -**C**H), 34.4 (2 C, **C$_q$**), 30.4 (6 C, -**C**H$_3$).

**$^{19}$F-NMR** (282 MHz, $CDCl_3$, 298 K) $\delta$ / ppm = -116.1 (1 F, Ar-**F**, major), -117.0 (1 F, Ar-**F**, minor).

**HRMS** (ESI-TOF): *m/z:* $[M+H]^+$ calcd for $C_{40}H_{46}FN_2O_3^+$ 621.3487 found 621.3487.

**(2*R*, 3*R*)-4-nitrophenyl 3-(3,5-di-tert-butyl-4-hydroxyphenyl)-2-((diphenylmethylene)amino)-3-(4-chlorophenyl)propanoate (8m)**

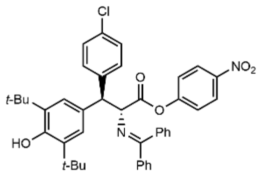


**8m**

Following general procedure F, the product was obtained as a colourless oil in a yield of 27.6 mg (80%) as a mixture of diastereomers. For HPLC analysis, and determination of enantiomeric ratio, derivatization to the morpholine amide was necessary.

***r$_f$*** (heptanes/EtOAc = 5/1) = 0.41

$[\boldsymbol{\alpha}]_D^{24}$ (c 1, $CHCl_3$) = 97

**$^1$H-NMR** (300 MHz, $CDCl_3$, 298 K) $\delta$ / ppm = 8.15 (d, *J* = 9.1 Hz, 2 H, Ar-**H**), 7.63-7.51 (m, 3 H, Ar-**H**), 7.43-7.27 (m, 10 H, Ar-**H**), 7.14 (s, 2 H, Ar-**H**), 6.76 (d, *J* = 9.1 Hz, 2 H, Ar-**H**), 6.55 (br. s, 1 H, Ar-**H**), 5.12 (s, 1 H, -O**H**), 4.86 (d, *J* = 9.0 Hz, 1 H, -C**H**), 4.82 (d, *J* = 9.0 Hz, 1 H, -C**H**), 1.32 (s, 18 H, -C**H$_3$**). *Characteristic signals for minor diastereomer:* 8.13 (d, *J* = 9.1 Hz, 2 H, Ar-**H**), 7.01 (s, 2 H, Ar-**H**), 6.67 (d, *J* = 9.1 Hz, 2 H, Ar-**H**), 5.13 (s, 1 H, -O**H**), 4.90 (d, *J* = 7.4 Hz, 1 H, -C**H**), 4.77 (d, *J* = 7.4 Hz, -C**H**), 1.32 (s, 18 H, -C**H$_3$**).

**$^{13}$C-NMR** (75 MHz, $CDCl_3$, 298 K) $\delta$ / ppm = 172.2 (1 C, -**C**OOR), 169.0 (1 C, -**C**=N), 155.5 (1 C, **C$_{Ar}$**), 152.9 (1 C, **C$_{Ar}$**), 145.5 (1 C, **C$_{Ar}$**), 139.6 (1 C, **C$_{Ar}$**), 139.2 (1 C, **C$_{Ar}$**), 135.7 (2 C, **C$_{Ar}$**), 132.8 (1 C, **C$_{Ar}$**), 130.9 (2 C, **C$_{Ar}$**), 130.4 (2 C, **C$_{Ar}$**), 129.1 (2 C, **C$_{Ar}$**), 128.9 (1 C, **C$_{Ar}$**), 128.7 (2 C, **C$_{Ar}$**), 128.5 (2 C, **C$_{Ar}$**), 128.4 (1 C, **C$_{Ar}$**), 128.2 (2 C, **C$_{Ar}$**), 127.8 (2 C, **C$_{Ar}$**), 126.2 (2 C, **C$_{Ar}$**), 125.2 (2 C, **C$_{Ar}$**), 122.4 (2 C, **C$_{Ar}$**), 71.2 (1 C, -**C**H), 54.6 (1 C, -**C**H), 34.4 (2 C, **C$_q$**), 30.3 (6 C, -**C**H$_3$). *Characteristic signals for minor diastereomer:* 172.3 (1 C, -**C**OOR), 169.2 (1 C, -**C**=N), 70.7 (1 C, -**C**H), 54.9 (1 C, -**C**H), 34.4 (2 C, **C$_q$**), 30.3 (6 C, -**C**H$_3$).

**HRMS** (ESI-TOF): *m/z:* $[M+H]^+$ calcd for $C_{42}H_{42}ClN_2O_5^+$ 689.2777 found 689.2773.

**Morpholine amide 9m**

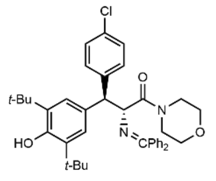

9m

Morpholine amide **9m** was synthesized in order to determine the enantiomeric ratio of product **8m**. The product was obtained as a white residue as a mixture of diastereomers (*dr* 80:20, $er_{major}$ 94:6, $er_{minor}$ 86:14).

**HPLC** (YMC-SA, *n*-hexane/IPA = 20/1, flow = 0.5 mL min$^{-1}$, $T_{Column}$ = 10 °C, *l* = 272 nm): $t_R$(major diastereomer): 27.81 min (minor), 41.54 min (major) ; $t_R$(minor diastereomer) = 34.53 min (minor), 35.51 min (major).

$r_f$ (heptanes/EtOAc = 2/1) = 0.31

$[\alpha]_D^{24}$ (c 1, $CHCl_3$) = 34

**$^1$H-NMR** (300 MHz, $CDCl_3$, 298 K) *δ* / ppm = 7.52-7.29 (m, 8 H, Ar-**H**), 7.22-7.18 (m, 5 H, Ar-**H**), 6.96 (s, 2 H, Ar-**H**), 6.49 (br. s, 1 H, Ar-**H**), 5.05 (s, 1 H, -O**H**), 4.90-4.81 (m, 2 H, -C**H**), 3.48-2.78 (br., 8 H, -C**H$_2$**), 1.27 (s, 18 H, -C**H$_3$**). *Characteristic signals for minor diastereomer:* 6.99 (s, 2 H, Ar-**H**), 5.07 (s, 1 H, -O**H**), 1.33 (s, 18 H, -C**H$_3$**).

**$^{13}$C-NMR** (75 MHz, $CDCl_3$, 298 K) *δ* / ppm = 169.6 (1 C, -**C**ONR$_2$), 169.3 (1 C, -**C**=N), 152.6 (1 C, **C$_{Ar}$**), 139.5 (1 C, **C$_{Ar}$**), 139.4 (1 C, **C$_{Ar}$**), 136.4 (1 C, **C$_{Ar}$**), 135.5 (2 C, **C$_{Ar}$**), 132.6 (1 C, **C$_{Ar}$**), 131.0 (1 C, **C$_{Ar}$**), 130.5 (2 C, **C$_{Ar}$**), 129.0 (2 C, **C$_{Ar}$**), 128.8 (1 C, **C$_{Ar}$**), 128.6 (1 C, **C$_{Ar}$**), 128.5 (2 C, **C$_{Ar}$**), 128.4 (2 C, **C$_{Ar}$**), 128.0 (2 C, **C$_{Ar}$**), 127.9 (2 C, **C$_{Ar}$**), 126.2 (2 C, **C$_{Ar}$**), 69.4 (1 C, -**C**H), 66.9 (1 C, -**C**H$_2$), 66.6 (1 C, -**C**H$_2$), 55.2 (1 C, -**C**H), 46.0 (1 C, -**C**H$_2$), 42.6 (1 C, -**C**H$_2$), 34.4 (2 C, **C$_q$**), 30.3 (6 C, -**C**H$_3$). *Characteristic signals for minor diastereomer:* 169.4 (1 C, -**C**ONR$_2$), 67.6 (1 C, -**C**H), 34.4 (2 C, **C$_q$**), 30.4 (6 C, -**C**H$_3$).

**HRMS** (ESI-TOF): *m/z:* $[M+H]^+$ calcd for $C_{40}H_{46}ClN_2O_3^+$ 637.3191 found 637.3196.

**(2*R*, 3*R*)-4-nitrophenyl 3-(3,5-di-tert-butyl-4-hydroxyphenyl)-2-((diphenylmethylene)amino)-3-(4-bromophenyl)propanoate (8n)**

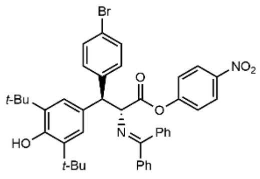

8n

Following general procedure F, the product was obtained as a colourless oil in a yield of 19.1 mg (52%) as a mixture of diastereomers. For HPLC analysis, and determination of enantiomeric ratio, derivatization to the morpholine amide was necessary.

$r_f$ (heptanes/EtOAc = 5/1) = 0.36

$[\alpha]_D^{24}$ (c 1, $CHCl_3$) = 112

**$^1$H-NMR** (300 MHz, $CDCl_3$, 298 K) *δ* / ppm = 8.15 (d, *J* = 8.8 Hz, 2 H, Ar-**H**), 7.44-7.29 (m, 9 H, Ar-**H**), 7.19 (d, *J* = 8.8 Hz, 1 H, Ar-**H**), 7.13 (s, 2 H, Ar-**H**), 6.75 (d, *J* = 8.8 Hz, 2 H, Ar-**H**), 5.11 (1 H, -O**H**), 4.84 (d, *J* = 9.0 Hz, 1 H, -C**H**), 4.79 (d, *J* = 9.0 Hz, 1 H, -C**H**), 1.31 (s, 18 H, -C**H$_3$**). *Characteristic signals for minor diastereomer:* 8.13 (d, *J* = 8.8 Hz, 2 H, Ar-**H**), 7.61 (d, *J* = 7.6 Hz, 2 H, Ar-**H**), 6.99 (s, 2 H, Ar-**H**), 6.66 (d, *J* = 8.8 Hz, 2 H, Ar-**H**), 5.12 (s, 1 H, -O**H**), 4.88 (d, *J* = 7.3 Hz, 1 H, -C**H**), 4.74 (d, *J* = 7.3 Hz, 1 H, -C**H**).

**$^{13}$C-NMR** (75 MHz, $CDCl_3$, 298 K) *δ* / ppm = 172.2 (1 C, -**C**OOR), 169.2 (1 C, -**C**=N), 155.5 (1 C, $\mathbf{C_{Ar}}$), 152.9 (1 C, $\mathbf{C_{Ar}}$), 145.5 (1 C, $\mathbf{C_{Ar}}$), 140.1 (1 C, $\mathbf{C_{Ar}}$), 139.2 (1 C, $\mathbf{C_{Ar}}$), 136.0 (1 C, $\mathbf{C_{Ar}}$), 135.8 (2 C, $\mathbf{C_{Ar}}$), 131.7 (2 C, $\mathbf{C_{Ar}}$), 131.2 (1 C, $\mathbf{C_{Ar}}$), 130.9 (1 C, $\mathbf{C_{Ar}}$), 130.7 (2 C, $\mathbf{C_{Ar}}$), 130.1 (1 C, $\mathbf{C_{Ar}}$), 129.1 (2 C, $\mathbf{C_{Ar}}$), 128.9 (1 C, $\mathbf{C_{Ar}}$), 128.5 (2 C, $\mathbf{C_{Ar}}$), 128.2 (2 C, $\mathbf{C_{Ar}}$), 127.8 (2 C, $\mathbf{C_{Ar}}$), 126.2 (2 C, $\mathbf{C_{Ar}}$), 125.2 (2 C, $\mathbf{C_{Ar}}$), 122.4 (2 C, $\mathbf{C_{Ar}}$), 71.1 (1 C, -**C**H), 54.7 (1 C, -**C**H), 34.5 (2 C, $\mathbf{C_q}$), 30.3 (6 C, **C**$H_3$). *Characteristic signals for minor diastereomer:* 172.3 (1 C, -**C**OOR), 169.0 (1 C, -**C**=N), 70.7 (1 C, -**C**H), 55.0 (1 C, -**C**H), 34.4 (1 C, $\mathbf{C_q}$), 30.3 (6 C, -**C**$H_3$).

**HRMS** (ESI-TOF): *m/z:* $[M+H]^+$ calcd for $C_{42}H_{42}BrN_2O_5^+$ 733.2272 found 733.2276.

### Morpholine amide 9n

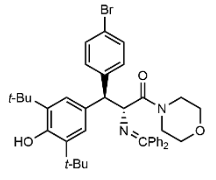

**9n**

Morpholine amide **9n** was synthesized in order to determine the enantiomeric ratio of product **8n**. The product was obtained as a white residue as a mixture of diastereomers (*dr* 80:20, $er_{major}$ 91:9, $er_{minor}$ 82:18).

**HPLC** (YMC-SA, *n*-hexane/IPA = 20/1, flow = 0.5 mL $min^{-1}$, $T_{Column}$ = 10 °C, *l* = 272 nm): $t_R$(major diastereomer): 29.58 min (minor), 43.44 min (major) ; $t_R$(minor diastereomer) = 34.10 min (minor), 36.56 min (major).

$r_f$ (heptanes/EtOAc = 2/1) = 0.31

$[\alpha]_D^{24}$ (c 1, $CHCl_3$) = 60

**$^1$H-NMR** (300 MHz, $CDCl_3$, 298 K) *δ* / ppm = 7.52-7.29 (m, 9 H, Ar-**H**), 7.24-7.20 (m, 1 H, Ar-**H**), 7.15 (d, *J* = 8.6 Hz, 2 H, Ar-**H**), 6.96 (s, 2 H, Ar-**H**), 6.49 (br. s, 1 H, Ar-**H**), 5.05 (s, 1 H, -O**H**), 4.90-4.80 (m, 2 H, -C**H**), 3.42-2.62 (br., 8 H, -C$\mathbf{H_2}$), 1.27 (s, 18 H, -C$\mathbf{H_3}$). *Characteristic signals for minor diastereomer:* 7.11 (d, *J* = 6.4 Hz, 2 H, Ar-**H**), 7.00 (s, 2 H, Ar-**H**), 5.07 (s, 1 H, -O**H**), 1.33 (s, 18 H, -C$\mathbf{H_3}$).

**$^{13}$C-NMR** (75 MHz, $CDCl_3$, 298 K) *δ* / ppm = 169.5 (1 C, -**C**ONR$_2$), 169.3 (1 C, -**C**=N), 152.7 (1 C, $\mathbf{C_{Ar}}$), 140.1 (1 C, $\mathbf{C_{Ar}}$), 139.4 (1 C, $\mathbf{C_{Ar}}$), 136.4 (1 C, $\mathbf{C_{Ar}}$), 135.9 (1 C, $\mathbf{C_{Ar}}$), 135.6 (2 C, $\mathbf{C_{Ar}}$), 131.5 (2 C, $\mathbf{C_{Ar}}$), 130.9 (2 C, $\mathbf{C_{Ar}}$), 130.5 (1 C, $\mathbf{C_{Ar}}$), 129.0 (2 C, $\mathbf{C_{Ar}}$), 128.8 (1 C, $\mathbf{C_{Ar}}$), 128.4 (2 C, $\mathbf{C_{Ar}}$), 128.0 (2 C, $\mathbf{C_{Ar}}$), 127.9 (2 C, $\mathbf{C_{Ar}}$), 126.2 (2 C, $\mathbf{C_{Ar}}$), 120.7 (1 C, $\mathbf{C_{Ar}}$), 69.4 (1 C, -**C**H), 66.9 (1 C, -**C**H$_2$), 66.7 (1 C, -**C**H$_2$), 55.3 (1 C, -**C**H), 46.0 (1 C, -**C**H$_2$), 42.6 (1 C, -**C**H$_2$), 34.4 (2 C, $\mathbf{C_q}$), 30.3 (6 C, -**C**H$_3$). *Characteristic signals for minor diastereomer:* 170.10 (1 C, -**C**ONR$_2$), 67.6 (1 C, -**C**H), 56.2 (1 C, -**C**H), 34.4 (2 C, $\mathbf{C_q}$), 30.4 (6 C, -**C**H$_3$).

**HRMS** (ESI-TOF): *m/z:* $[M+H]^+$ calcd for $C_{40}H_{46}BrN_2O_3^+$ 681.2686 found 681.2686

**(2*R*, 3*S*)-4-nitrophenyl 3-(3,5-di-tert-butyl-4-hydroxyphenyl)-2-((diphenylmethylene)amino)-3-(naphthalen-1-yl)propanoate (8o)**

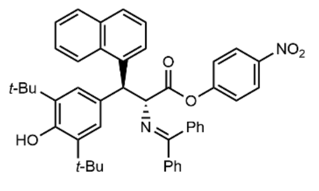

**8o**

0.05 mmol scale: Following general procedure F, the product was obtained as an off-white residue in a yield of 22.9 mg (65%) as a mixture of diastereomers (*dr* 95:5, $er_{major}$ 97:3). 1.0 mmol scale: Obtained as an off-white solid in a yield of 66%, *dr* 96:4, $er_{major}$ 97:3. Further enantioenrichment could be achieved by a single recrystallisation step from *n*-hexane/EtOAc, yielding **8o** as a single diastereomer with an *er* of 98.3:1.7.

**HPLC** (YMC-SA, n-hexane/IPA = 10/1, flow = 0.5 mL min$^{-1}$, $T_{Column}$ = 10 °C, *l* = 240 nm): $t_R$(major diastereomer): 12.27 (minor), 13.31 (major); $t_R$(minor diastereomer) = 18.44 (minor), 22.07 (major).

$r_f$ (heptanes/EtOAc = 5/1) = 0.40

$[\alpha]_D^{24}$ (c 1, $CHCl_3$) = 239

**$^1$H-NMR** (300 MHz, $CDCl_3$, 298 K) $\delta$ / ppm = 8.22 (dd, $J_1$ = 3.3 Hz, $J_2$ = 6.2 Hz, 1 H, Ar-**H**), 8.04 (d, *J* = 9.2 Hz, 2 H, Ar-**H**), 7.85 (dd, $J_1$ = 3.3 Hz, $J_2$ = 6.2 Hz, 1 H, Ar-**H**), 7.75 (dd, $J_1$ = 1.0 Hz, $J_2$ = 7.7 Hz, 1 H, Ar-**H**), 7.67-7.64 (m, 2 H, Ar-**H**), 7.48-7.24 (m, 13 H, Ar-**H**), 6.58 (d, *J* = 9.2 Hz, 2 H, Ar-**H**), 6.41 (br. s, 1 H, Ar-**H**), 5.72 (d, *J* = 8.9 Hz, 1 H, -C**H**), 5.08 (d, *J* = 8.9 Hz, 1 H, -C**H**), 5.08 (s, 1 H, -O**H**), 1.30 (s, 18 H, -C**H$_3$**).

**$^{13}$C-NMR** (75 MHz, $CDCl_3$, 298 K) $\delta$ / ppm = 172.1 (1 C, -**C**OOR), 169.3 (1 C, -**C**=N), 155.6 (1 C, $\mathbf{C_{Ar}}$), 152.8 (1 C, $\mathbf{C_{Ar}}$), 145.4 (1 C, $\mathbf{C_{Ar}}$), 139.3 (1 C, $\mathbf{C_{Ar}}$), 137.0 (1 C, $\mathbf{C_{Ar}}$), 135.7 (1 C, $\mathbf{C_{Ar}}$), 135.6 (2 C, $\mathbf{C_{Ar}}$), 134.2 (1 C, $\mathbf{C_{Ar}}$), 132.0 (1 C, $\mathbf{C_{Ar}}$), 130.8 (1 C, $\mathbf{C_{Ar}}$), 130.3 (1 C, $\mathbf{C_{Ar}}$), 129.1 (2 C, $\mathbf{C_{Ar}}$), 128.9 (1 C, $\mathbf{C_{Ar}}$), 128.6 (1 C, $\mathbf{C_{Ar}}$), 128.3 (2 C, $\mathbf{C_{Ar}}$), 128.1 (2 C, $\mathbf{C_{Ar}}$), 127.8 (2 C, $\mathbf{C_{Ar}}$), 127.7 (1 C, $\mathbf{C_{Ar}}$), 126.5 (1 C, $\mathbf{C_{Ar}}$), 126.4 (2 C, $\mathbf{C_{Ar}}$), 125.8 (1 C, $\mathbf{C_{Ar}}$), 125.3 (1 C, $\mathbf{C_{Ar}}$), 125.1 (2 C, $\mathbf{C_{Ar}}$), 125.0 (1 C, $\mathbf{C_{Ar}}$), 123.8 (1 C, $\mathbf{C_{Ar}}$), 122.4 (2 C, $\mathbf{C_{Ar}}$), 71.4 (1 C, -**C**H), 49.9 (1 C, -**C**H), 34.4 (2 C, $\mathbf{C_q}$), 30.4 (6 C, -**C**H$_3$).

**HRMS** (ESI-TOF): *m/z:* $[M+H]^+$ calcd for $C_{46}H_{45}N_2O_5^+$ 705.3323 found 705.3326.

**(2R, 3R)-4-nitrophenyl 3-(3,5-di-tert-butyl-4-hydroxyphenyl)-2-((diphenylmethylene)amino)-3-(naphthalen-2-yl)propanoate (8p)**

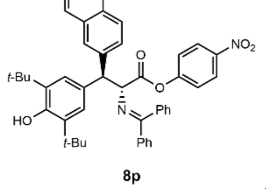

**8p**

Following general procedure F, the product was obtained as an off-white residue in a yield of 31.0 mg (88%) as a mixture of diastereomers (*dr* 80:20, $er_{major}$ 94:6, $er_{minor}$ 16:84).

**HPLC** (YMC-SB, *n*-hexane/IPA = 10/1, flow = 0.5 mL min$^{-1}$, $T_{Column}$ = 10 °C, *l* = 220 nm): $t_R$(major diastereomer): 16.11 (major), 21.97 (minor); $t_R$(minor diastereomer) = 15.34 (minor), 20.70 (major).

$r_f$ (heptanes/EtOAc = 5/1) = 0.42

$[\alpha]_D^{24}$ (c 1, $CHCl_3$) = 102

**$^1$H-NMR** (300 MHz, $CDCl_3$, 298 K) *δ* / ppm = 8.03 (d, *J* = 8.9 Hz, 2 H, Ar-**H**), 7.81-7.75 (m, 4 H, Ar-**H**), 7.65-7.60 (m, 2 H, Ar-**H**), 7.47-7.30 (m, 9 H, Ar-**H**), 7.22 (s, 2 H, Ar-**H**), 6.65 (d, *J* = 8.9 Hz, 2 H, Ar-**H**), 6.55 (br. s, 2 H, Ar-**H**), 5.10 (s, 1 H, -O**H**), 5.07-4.96 (m, 2 H, -C**H**), 1.31 (s, 18 H, -C**H$_3$**). *Characteristic signals for minor diastereomer:* 8.07 (d, *J* = 8.9 Hz, 2 H, Ar-**H**), 7.12 (2 H, Ar-**H**), 6.63 (d, *J* = 8.9 Hz, 2 H, Ar-**H**), 5.11 (s, 1 H, -O**H**), 5.07-4.96 (m, 2 H, -C**H**).

**$^{13}$C-NMR** (75 MHz, $CDCl_3$, 298 K) *δ* / ppm = 172.0 (1 C, -**C**OOR), 169.3 (1 C, -**C**=N), 155.5 (1 C, **C$_{Ar}$**), 152.8 (1 C, **C$_{Ar}$**), 145.4 (1 C, **C$_{Ar}$**), 139.3 (1 C, **C$_{Ar}$**), 138.5 (1 C, **C$_{Ar}$**), 135.9 (1 C, **C$_{Ar}$**), 135.7 (2 C, **C$_{Ar}$**), 133.5 (1 C, **C$_{Ar}$**), 132.5 (1 C, **C$_{Ar}$**), 130.8 (1 C, **C$_{Ar}$**), 130.6 (1 C, **C$_{Ar}$**), 129.1 (2 C, **C$_{Ar}$**), 128.8 (1 C, **C$_{Ar}$**), 128.5 (2 C, **C$_{Ar}$**), 128.2 (1 C, **C$_{Ar}$**), 128.1 (2 C, **C$_{Ar}$**), 128.0 (1 C, **C$_{Ar}$**), 127.9 (2 C, **C$_{Ar}$**), 127.7 (1 C, **C$_{Ar}$**), 127.5 (1 C, **C$_{Ar}$**), 127.3 (1 C, **C$_{Ar}$**), 126.4 (2 C, **C$_{Ar}$**), 126.3 (1 C, **C$_{Ar}$**), 125.9 (1 C, **C$_{Ar}$**), 125.1 (2 C, **C$_{Ar}$**), 122.4 (2 C, **C$_{Ar}$**), 71.5 (1 C, -**C**H), 55.5 (1 C, -**C**H), 34.4 (2 C, **C$_q$**), 30.4 (6 C, -**C**H$_3$). *Characteristic signals for minor diastereomer:* 172.1 (1 C, -**C**OOR), 169.5 (1 C, -**C**=N), 71.0 (1 C, -**C**H), 55.9 (1 C, -**C**H), 34.4 (1 C, **C$_q$**), 30.3 (6 C, -**C**H$_3$).

**HRMS** (ESI-TOF): *m/z:* $[M+H]^+$ calcd for $C_{46}H_{45}N_2O_5^+$ 705.3323 found 705.3327.

**(2*R*, 3*R*)-4-nitrophenyl 3-(3,5-di-tert-butyl-4-hydroxyphenyl)-2-((diphenylmethylene)amino)-3-(thiophen-2-yl)propanoate (8q)**

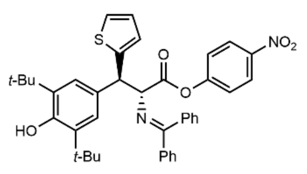

**8q**

Following general procedure F, the product was obtained as a colourless oil in a yield of 31.4 mg (95%) as a mixture of diastereomers (*dr* 55:45, *er$_{major}$* 93:7, *er$_{minor}$* 68:32).

**HPLC** (YMC-SA, *n*-hexane/IPA = 10/1, flow = 0.5 mL min$^{-1}$, $T_{Column}$ = 10 °C, *l* = 220 nm): *t$_R$*(major diastereomer): 13.91 min (major), 20.27 min (minor); *t$_R$*(minor diastereomer) = 15.24 min (major), 17.51 min (minor).

***r$_f$*** (heptanes/EtOAc = 5/1) = 0.47

$[\alpha]_D^{24}$ (c 1, $CHCl_3$) = 70.5

**$^1$H-NMR** (300 MHz, $CDCl_3$, 298 K) *δ* / ppm°= 8.16 (d, *J* = 8.3 Hz, 2 H, Ar-**H**), 7.61 (d, *J* = 7.9 Hz, 2 H, Ar-**H**), 7.43-7.31 (m, 6 H, Ar-**H**), 7.21 (s, 2 H, Ar-**H**), 7.21-7.17 (m, 1 H, Ar-**H**), 6.98-6.95 (m, 2 H, Ar-**H**), 6.82 (d, *J* = 8.3 Hz, 2 H, Ar-**H**), 6.64 (br. 2 H, Ar-**H**), 5.12-5.09 (m, 2 H, -C**H** + -O**H**), 4.78 (d, *J* = 8.8 Hz, 1 H, -C**H**), 4.74 (d, *J* = 5.6 Hz, 1 H, -C**H**), 1.34 (s, 18 H). *Characteristic signals for minor diastereomer:* 8.13 (d, *J* = 8.8 Hz, 2 H, Ar-**H**), 7.75 (d, *J* = 7.9 Hz, 2 H, Ar-**H**), 7.11 (s, 2 H, Ar-**H**), 6.73 (d, *J* = 8.8 Hz, 2 H, Ar-**H**), 6.51 (br. 2 H, Ar-**H**), 1.30 (s, 18 H).

**$^{13}$C-NMR** (75 MHz, $CDCl_3$, 298 K) *δ* / ppm = 172.3 (1 C, -**C**OOR), 168.9 (1 C, -**C**=N), 155.6 (1 C, **C$_{Ar}$**), 153.0 (1 C, **C$_{Ar}$**), 145.5 (1 C, **C$_{Ar}$**), 143.8 (1 C, **C$_{Ar}$**), 139.3 (1 C, **C$_{Ar}$**), 135.8 (1 C, **C$_{Ar}$**), 135.6 (1 C, **C$_{Ar}$**), 130.8 (1 C, **C$_{Ar}$**), 130.2 (1 C, **C$_{Ar}$**), 129.2 (2 C, **C$_{Ar}$**), 128.9 (1 C, **C$_{Ar}$**), 128.5 (2 C, **C$_{Ar}$**), 128.1 (2 C, **C$_{Ar}$**), 127.9 (2 C, **C$_{Ar}$**), 127.5 (1 C, **C$_{Ar}$**), 126.7 (2 C, **C$_{Ar}$**), 126.1 (2 C, **C$_{Ar}$**), 125.9 (2 C, **C$_{Ar}$**), 125.2 (1 C, **C$_{Ar}$**), 122.5 (2 C, **C$_{Ar}$**), 72.5 (1 C, -**C**H), 50.9 (1 C, -**C**H), 34.5 (2 C, **C$_q$**), 30.4 (6 C, -**C**H$_3$). *Characteristic*

*signals for minor diastereomer:*172.3 (1 C, -**C**OOR), 168.9 (1 C, -**C**=N), 155.6 (1 C, $\mathbf{C_{Ar}}$), 153.0 (1 C, $\mathbf{C_{Ar}}$),145.5 (1 C, $\mathbf{C_{Ar}}$), 143.0 (1 C, $\mathbf{C_{Ar}}$), 139.1 (1 C, $\mathbf{C_{Ar}}$), 71.1 (1 C, -**C**H), 51.3 (1 C, -**C**H), 34.4 (2 C, $\mathbf{C_q}$), 30.3 (6 C, -**C**$H_3$).

**HRMS** (ESI-TOF): *m/z:* $[M+H]^+$ calcd for $C_{40}H_{41}N_2O_5S^+$ 661.2731 found 661.2735.

**(2*R*, 3*S*)-4-nitrophenyl 3-(3,5-di-tert-butyl-4-hydroxyphenyl)-2-((diphenylmethylene)amino)-3-(pyridin-3-yl)propanoate (8r)**

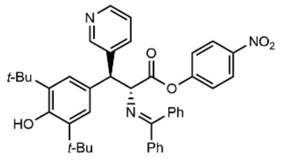

**8r**

Following general procedure F, the product was obtained as a colourless oil in a yield of 21.3 mg (65%) as a mixture of diastereomers (*dr* 80:20, $er_{major}$ 78:22). Due to overlapping peaks, the diastereomers were separated by semipreparative HPLC prior to HPLC analysis with chiral stationary phase.

**Semipreparative HPLC** (Grace Alltima Silica 10µ, length 250 mm, ID 10 mm, *n*-hexane/EtOAc = 75/25): $t_R$(minor) = 13.38 min, $t_R$(major) = 14.49 min.

**HPLC** (YMC-SB, *n*-hexane/IPA = 10/1, flow = 0.5 mL min$^{-1}$, $T_{Column}$ = 10 °C, *l* = 220 nm): $t_R$(major diastereomer): 13.03 min (minor), 15.65 min (major)

$r_f$ (heptanes/EtOAc = 5/1) = 0.05

$[\alpha]_D^{24}$ (c 1, $CHCl_3$) = 77

**$^1$H-NMR** (300 MHz, $CDCl_3$, 298 K) *δ* / ppm = 8.60 (d, *J* = 1.9 Hz, 1 H, Ar-**H**), 8.49-8.47 (m, 1 H, Ar-**H**), 8.13 (d, *J* = 9.1 Hz, 2 H, Ar-**H**), 7.63-7.60 (m, 3 H, Ar-**H**), 7.43-7.30 (m, 6 H, Ar-**H**), 7.24-7.10 (m, 1 H, Ar-**H**), 7.22 (s, 2 H, Ar-**H**), 6.75 (d, *J* = 9.1 Hz, 2 H, Ar-**H**), 6.58-6.56 (br. 2 H, Ar-**H**), 5.16 (s, 1 H, -O**H**), 4.90 (d, *J* = 8.5 Hz, 1 H, -C**H**), 4.85 (d, *J* = 8.5 Hz, 1 H, -C**H**), 1.34 (s, 18 H, -C$\mathbf{H_3}$). *Characteristic signals for minor diastereomer:* 8.84 (d, *J* = 1.8 Hz, 1 H, Ar-**H**), 8.13 (d, *J* = 9.1 Hz, 2 **H**, Ar-**H**), 7.02 (s, 2 H, Ar-**H**), 6.71 (d, *J* = 9.1 Hz, 2 H, Ar-**H**), 4.91 (d, *J* = 7.1 Hz, 1 H, -C**H**), 4.80 (d, *J* = 7.1 Hz, 1 H, -C**H**), 1.31 (s, 18 H, -C$\mathbf{H_3}$).

**$^{13}$C-NMR** (75 MHz, $CDCl_3$, 298 K) *δ* / ppm = 172.5 (1 C, -**C**OOR), 168.8 (1 C, -**C**=N), 155.3 (1 C, $\mathbf{C_{Ar}}$), 153.1 (1 C, $\mathbf{C_{Ar}}$), 150.2 (1 C, $\mathbf{C_{Ar}}$), 148.3 (1 C, $\mathbf{C_{Ar}}$), 145.5 (1 C, $\mathbf{C_{Ar}}$), 139.0 (1 C, $\mathbf{C_{Ar}}$), 136.5 (1 C, $\mathbf{C_{Ar}}$), 135.9 (2 C, $\mathbf{C_{Ar}}$), 135.6 (1 C, $\mathbf{C_{Ar}}$), 131.0 (1 C, $\mathbf{C_{Ar}}$), 129.6 (1 C, $\mathbf{C_{Ar}}$),129.1 (2 C, $\mathbf{C_{Ar}}$), 129.0 (1 C, $\mathbf{C_{Ar}}$), 128.6 (2 C, $\mathbf{C_{Ar}}$), 128.2 (2 C, $\mathbf{C_{Ar}}$), 127.7 (2 C, $\mathbf{C_{Ar}}$), 126.3 (2 C, $\mathbf{C_{Ar}}$), 125.8 (1 C, $\mathbf{C_{Ar}}$), 125.2 (2 C, $\mathbf{C_{Ar}}$), 123.4 (1 C, $\mathbf{C_{Ar}}$), 122.4 (2 C, $\mathbf{C_{Ar}}$), 70.6 (1 C, -**C**H), 52.8 (1 C, -**C**H), 34.5 (2 C, $\mathbf{C_q}$), 30.3 (6 C, -**C**$H_3$). *Characteristic signals for minor diastereomer:* 172.6 (1 C, -**C**OOR), 169.0 (1 C, -**C**=N), 70.5 (1 C, -**C**H), 53.2 (1 C, -**C**H), 34.4 (2 C, $\mathbf{C_q}$), 30.2 (6 C, -**C**$H_3$).

**HRMS** (ESI-TOF): *m/z:* $[M+H]^+$ calcd for $C_{41}H_{42}N_3O_5^+$ 656.3119 found 656.3146.

**(2*S*, 3*S*)-4-nitrophenyl 3-(3,5-di-tert-butyl-4-hydroxyphenyl)-2-((diphenylmethylene)amino)butanoate (8s)**

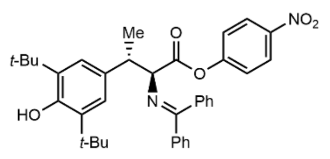


**8s**

The product was synthesized with slight adaptions to general procedure F. Instead of TM ·HCl (**ITU 5**), BTM (**ITU 4**) was used as catalyst and the reaction time was extended to 64 h. The product was obtained as a colourless oil in a yield of 16.0 mg (54%, 60% conversion) as a mixture of diastereomers (*dr* 85:15, $er_{major}$ 96:4, $er_{minor}$ 36:64).

**HPLC** (YMC-SA, *n*-hexane/IPA = 20/1, flow = 0.5 mL min$^{-1}$, $T_{Column}$ = 10 °C, *l* = 272 nm): $t_R$(major diastereomer): 19.99 min (major), 48.45 min (minor); $t_R$(minor diastereomer): 17.30 min (minor), 20.88 min (major).

$r_f$ (heptanes/EtOAc = 5/1) = 0.54

$[\alpha]_D^{24}$ (c 1, $CHCl_3$) = -25

**$^1$H-NMR** (300 MHz, CDCl3, 298 K) *δ* / ppm = 8.24 (d, *J* = 9.1 Hz, 2 H, Ar-**H**), 7.63-7.57 (m, 2 H, Ar-**H**),7.42-7.30 (m, 5 H, Ar-**H**), 7.16 (d, *J* = 9.1 Hz, 2 H, Ar-**H**), 7.06 (s, 2 H, Ar-**H**), 6.68 (br, 2 H, Ar-**H**), 5.09 (1 H, -O**H**), 4.26 (d, *J* = 8.0 Hz, 1 H, -C**H**), 3.61 (m, 1 H, -C**H**), 1.40 (d, *J* = 7.1 Hz, 3 H, -C**H$_3$**), 1.37 (s, 18 H, -C**H$_3$**) *Characteristic signals for minor diastereomer:* 8.20 (d, *J* = 9.1 Hz, 2 H, Ar-**H**), 7.72-7.69 (m, 2 H, Ar-**H**), 7.00 (d, *J* = 9.1 Hz, 2 H, Ar-**H**), 6.95 (s, 2 H, Ar-**H**), 5.10 (s, 1 H, -O**H**), 4.38 (d, *J* = 5.6 Hz, 1 H, -C**H**), 1.53 (d, *J* = 7.1 Hz, 3 H, -**C**H$_3$), 1.32 (s, 18 H, -C**H$_3$**).

**$^{13}$C-NMR** (75 MHz, $CDCl_3$, 298 K) *δ* / ppm = 171.2 (1 C, -**C**OOR), 169.7 (1 C, -**C**=N), 155.7 (1 C **C$_{Ar}$**), 152.7 (1 C **C$_{Ar}$**), 145.5 (1 C **C$_{Ar}$**), 139.3 (1 C **C$_{Ar}$**), 136.1 (1 C **C$_{Ar}$**), 135.4 (2 C **C$_{Ar}$**), 132.6 (1 C **C$_{Ar}$**), 130.7 (1 C **C$_{Ar}$**), 130.2 (1 C **C$_{Ar}$**), 129.0 (2 C **C$_{Ar}$**), 128.5 (2 C **C$_{Ar}$**), 128.1 (2 C **C$_{Ar}$**), 127.8 (2 C **C$_{Ar}$**), 125.3 (2 C **C$_{Ar}$**), 125.3 (2 C **C$_{Ar}$**), 122.6 (2 C **C$_{Ar}$**), 72.9 (1 C, -**C**H), 43.6 (1 C, -**C**H), 34.4 (2 C, **C$_q$**), 30.3 (6 C, -**C**H$_3$), 17.7 (1 C, -**C**H$_3$) *Characteristic signals for minor diastereomer:* 171.65 (1 C, -**C**OOR), 169.6 (1 C, -**C**=N), 71.4 (1 C, -**C**H), 44.1 (1 C, -**C**H), 34.4 (2 C, **C$_q$**), 30.3 (6 C, -**C**H$_3$), 16.7 (1 C, -**C**H$_3$).

**HRMS** (ESI-TOF): *m/z:* $[M+H]^+$ calcd for $C_{37}H_{41}N_2O_5$ 593.3010 found 593.3012

**(2*R*, 3*R*)-4-nitrophenyl 3-(3,5-di-tert-butyl-4-hydroxyphenyl)-2-((diphenylmethylene)amino)-4,4,4-trifluorobutanoate (8t)**

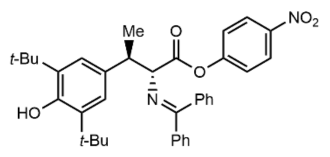


**8t**

Following general procedure F, the product was obtained as a colourless oil in a yield of 21.3 mg (94%) as a mixture of diastereomers (*dr* 83:17, $er_{major}$ 53:47*,* $er_{minor}$ 60:40).

**HPLC** (YMC-SB, *n*-hexane/IPA = 10/1, flow = 0.5 mL min$^{-1}$, $T_{Column}$ = 10 °C, *l* = 240 nm): $t_R$(major diastereomer): 13.92 min (major), 16.93 min (minor); $t_R$(minor diastereomer): 12.85 min (major), 15.48 min (minor).

$r_f$ (heptanes/EtOAc = 5/1) = 0.46

$[\alpha]_D^{24}$ (c 1, $CHCl_3$) = 5.9

**$^1$H-NMR** (300 MHz, CDCl3, 298 K) *δ* / ppm = 8.19 (d, *J* = 9.1 Hz, 2 H, Ar-**H**), 7.63-7.60 (m, 2 H, Ar-**H**), 7.49-7.32 (m, 8 H, Ar-**H**), 7.06-7.01 (m, 2 H, Ar-**H**), 6.91 (d, *J* = 9.1 Hz, 2 H, Ar-**H**), 5.28 (s, 1 H, -O**H**), 4.87 (d, *J* = 6.4 Hz, 1 H, -C**H**), 4.19-4.11 (m, 1 H, -C**H**$CF_3$), 1.42 (s, 18 H, -C**$H_3$**). *Characteristic signals for minor diastereomer:* 8.12 (d, *J* = 9.1 Hz, 2 H, Ar-**H**), 7.75-7.73 (m, 2 H, Ar-**H**), 6.67 (d, *J* = 9.1 Hz, 2 H, Ar-**H**), 5.29 (s, 1 H, -O**H**), 4.88 (d, *J* = 7.9 Hz, 1 H, -C**H**), 4.33-4.20 (m, 1 H, -C**H**$CF_3$), 1.33 (s, 18 H, -C**$H_3$**).

**$^{13}$C-NMR** (75 MHz, $CDCl_3$, 298 K) *δ* / ppm = 173.1 (1 C, -**C**OOR), 167.9 (1 C, -**C**=N), 155.3 (1 C, **$C_{Ar}$**), 154.3 (1 C, **$C_{Ar}$**), 145.7 (1 C, **$C_{Ar}$**), 139.2 (1 C, **$C_{Ar}$**), 135.9 (1 C, **$C_{Ar}$**), 135.7 (1 C, **$C_{Ar}$**), 131.0 (1 C, **$C_{Ar}$**), 129.3 (2 C, **$C_{Ar}$**), 129.2 (1 C, **$C_{Ar}$**), 128.8 (1 C, **$C_{Ar}$**), 128.1 (1 C, **$C_{Ar}$**), 127.8 (1 C, **$C_{Ar}$**), 127.5 (1 C, **$C_{Ar}$**), 125.3 (1 C, **$C_{Ar}$**), 122.4 (1 C, **$C_{Ar}$**), 65.7 (1 C, -**C**H), 53.1 (d, *J* = 26.0 Hz,1 C, -**C**H$CF_3$), 34.5 (2 C, **$C_q$**), 30.4 (6 C, -**C**$H_3$). *Characteristic signals for minor diastereomer:* 173.3 (1 C, -**C**OOR), 167.4 (1 C, -**C**=N), 67.5 (1 C,- **C**H), 34.4 (2 C, **$C_q$**), 30.2 (6 C, -**C**$H_3$).

**$^{19}$F-NMR** (282 MHz, $CDCl_3$, 298 K) *δ* / ppm = -64.6 (3 F, -C**$F_3$**, minor), -65.4 (3 F, -C**$F_3$**, major).

**HRMS** (ESI-TOF): *m/z:* $[M+H]^+$ calcd for $C_{37}H_{38}F_3N_2O_5^+$ 647.2727 found 647.2729

## 4.5 Synthesis of Chroman-2-one derivatives 14

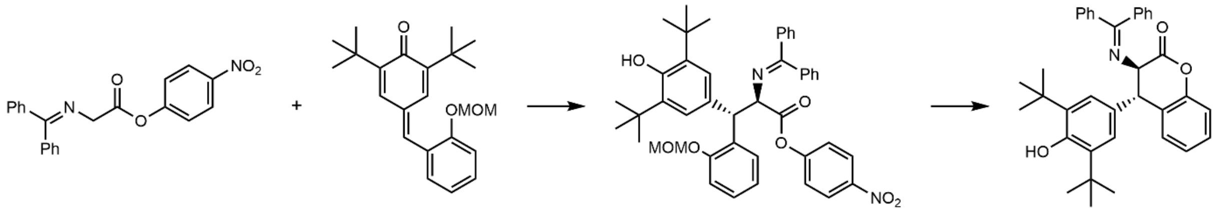


**1** **13b** **15** **14a**

At -40 °C (using a circulation chiller), Schiff base **1** (36.0 mg, 0.1 mmol, 1 eq) and TM·HCl (2.4 mg, 10 mol%) were dissolved in freshly degassed ACN (0.1 mol $L^{-1}$). Then, DIPEA (17.4 µL, 0.1 mmol, 1 eq) was added and the mixture was stirred for 5 min. The respective MOM protected quinone methide (0.1 mmol, 1 eq) was added in one portion and the mixture was stirred for 64 h at -40 °C. After this, the circulation chiller was turned off, and the mixture was allowed to reach rt over 1 h. It was filtered over $Na_2SO_4$ and washed with 2 mL DCM trice. The filtrate was concentrated on the rotary evaporator and the crude product was purified by column chromatography on deactivated silica (heptanes followed by heptanes/EtOAc 10/1 and 5/1). The deprotection was done according to a known procedure [38]. The product from the first step (1 eq) was dissolved in 2 mL DCM and added to $MgBr_2 \cdot Et_2O$ (35 eq) in a pressure resistant Schlenk flask. Then, 1 mL of $Me_2S$ was added, the flask was closed and the mixture heated to 40 °C for 6 h. After cooling to rt, the mixture was filtered over a plug of deactivated silica and thoroughly washed with DCM. The filtrate was concentrated via water jet vacuum (using a $H_2O_2$ washing bottle between the water jet pump and the flask containing the filtrate in order to oxidize remaining $Me_2S$ and to avoid stench) to yield the cyclization products in satisfying purity.

**(3*R*, 4*S*)-4-(3,5-di-tert-butyl-4-hydroxyphenyl)-3-((diphenylmethylene)amino)chroman-2-one (14a)**

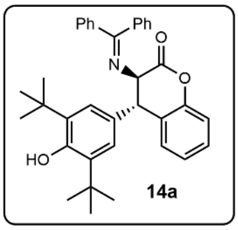


The product was obtained as a mixture of diastereomers (*dr* 90:10, $er_{major}$ 90:10, $er_{minor}$ 78:22) as a yellow oily residue in a yield of 63% after both steps.

**HPLC:** (YMC-SB, *n*-hexane/IPA = 20/1, flow = 0.5 mL $min^{-1}$, $T_{Column}$ = 10 °C, *l* = 240 nm): $t_R$(major diastereomer): 11.79 min (major), 14.46 min (minor) ; $t_R$(minor diastereomer) = 11.22 min (minor), 12.85 min (major).

$r_f$ (heptanes/EtOAc = 10/1) = 0.44

$[\alpha]_D^{24}$ (c 1, $CHCl_3$) = -26

**$^1$H-NMR** (300 MHz, $CDCl_3$, 298 K) *δ* / ppm°= 7.49-7.42 (m, 2 H, Ar-**H**), 7.37-7.28 (m, 5 H, Ar-**H**), 7.25-6.96 (m, 5 H, Ar-**H**), 6.83 (br, 2 H, Ar-**H**), 6.82 (s, 2 H, Ar-**H**), 5.12 (s, 1 H, -O**H**), 4.70 (d, *J* = 11.5 Hz, 1 H, -C**H**),4.33 (d, *J* = 11.5 Hz, 1 H, -C**H**), 1.34 (s, 18 H, -C**H₃**) *Characteristic signals for minor diastereomer:* 6.93 (s, 2 H, Ar-**H**), 6.68 (br, 2 H, Ar-**H**), 5.17 (1 H, -O**H**), 4.50 (d, *J* = 4.9 Hz, 1 H, -C**H**), 4.33 (d, *J* = 4.9 Hz, 1 H, -C**H**), 1.31 (s, 18 H, -C**H**$_3$).

**$^{13}$C-NMR** (75 MHz, $CDCl_3$, 298 K) $\delta$ / ppm = 172.7 (1 C, -**C**OOR), 167.4 (1 C, -**C**=N), 153.1 (1 C, **C$_{Ar}$**), 151.4 (1 C, **C$_{Ar}$**), 139.3 (1 C, **C$_{Ar}$**), 136.0 (2 C, **C$_{Ar}$**), 135.8 (1 C, **C$_{Ar}$**), 130.6 (1 C, **C$_{Ar}$**), 129.1 (2 C, **C$_{Ar}$**), 128.9 (1 C, **C$_{Ar}$**), 128.6 (1 C, **C$_{Ar}$**), 128.6 (2 H, **C$_{Ar}$**), 128.6 (1 C, **C$_{Ar}$**), 128.0 (2 C, **C$_{Ar}$**), 127.9 (2 C, **C$_{Ar}$**), 126.8 (1 C, **C$_{Ar}$**), 126.0 (2 C, **C$_{Ar}$**), 125.3 (1 C, **C$_{Ar}$**), 124.5 (1 C, **C$_{Ar}$**), 116.7 (1 C, **C$_{Ar}$**), 66.5 (1 C, -**C**H), 48.8 (1 C, -**C**H), 34.4 (2 C, **C$_q$**), 30.4 (6 C, -**C**$H_3$). *Characteristic signals for minor diastereomer:* 172.0 (1 C, -**C**OOR), 167.0 (1 C, -**C**=N), 153.3 (1 C, **C$_{Ar}$**), 151.8 (1 C, **C$_{Ar}$**), 65.6 (1 C, -**C**H), 48.2 (1 C, -**C**H), 34.4 (2 C, **C$_q$**), 30.3 (6 C, -**C**$H_3$).

**HRMS** (ESI-TOF): *m/z:* $[M+H]^+$ calcd for $C_{36}H_{38}NO_3^+$ 532.2846 found 532.2840

**(1*S*, 2*R*)-1-(3,5-di-tert-butyl-4-hydroxyphenyl)-2-((diphenylmethylene)amino)-1,2-dihydro-3H-benzo[f]chromen-3-one (14b)**

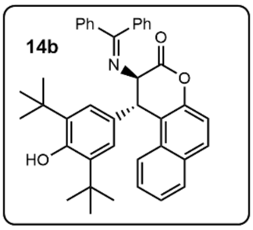


The product was obtained as a mixture of diastereomers (*dr* 85:15, $er_{major}$ 95:5, $er_{minor}$ 92:8) as a yellow oily residue in a yield of 45% after both steps.

**HPLC:** (Chiralcel AD-H, *n*-hexane/IPA = 20/1, flow = 0.5 mL min$^{-1}$, $T_{Column}$ = 10 °C, *l* = 220 nm): $t_R$(major diastereomer): 16.41 min (minor), 27.31 min (major) ; $t_R$(minor diastereomer) = 13.74 min (minor), 14.55 min (major).

$r_f$ (heptanes/EtOAc = 10/1) = 0.45

$[\alpha]_D^{24}$ (c 1, $CHCl_3$) = 106

**$^1$H-NMR** (300 MHz, $CDCl_3$, 298 K) $\delta$ / ppm°= 7.91-7.88 (m, 2 H, Ar-**H**), 7.70-7.67 (m, 1 H, Ar-**H**), 7.59-7.52 (m, 3 H, Ar-**H**), 7.47-7.32 (m, 8 H, Ar-**H**), 7.19-7.15 (m, 2 H, Ar-**H**), 6.54 (s, 2 H, Ar-**H**), 5.01 (s, 1H, -O**H**), 4.64 (d, *J* = 2.4 Hz, 1 H, -C**H**), 4.53 (d, *J* = 2.4 Hz, 1 H, -C**H**), 1.21 (s, 18 H, -C**H$_3$**).

**$^{13}$C-NMR** (75 MHz, $CDCl_3$, 298 K) $\delta$ / ppm = 171.9 (1 C, -**C**OOR), 166.8 (1 C, -**C**=N), 153.2 (1 C, **C$_{Ar}$**), 149.7 (1 C, **C$_{Ar}$**), 138.8 (1 C, **C$_{Ar}$**), 136.4 (2 C, **C$_{Ar}$**), 135.9 (1 C, **C$_{Ar}$**), 131.9 (1 C, **C$_{Ar}$**), 131.3 (1 C, **C$_{Ar}$**), 130.8 (1 C, **C$_{Ar}$**), 129.5 (1 C, **C$_{Ar}$**), 129.2 (1 C, **C$_{Ar}$**), 129.1 (2 C, **C$_{Ar}$**), 129.0 (2 C, **C$_{Ar}$**), 128.8 (2 C, **C$_{Ar}$**), 128.1 (2 C, **C$_{Ar}$**), 128.0 (2 C, **C$_{Ar}$**), 127.0 (1 C, **C$_{Ar}$**), 124.9 (1 C, **C$_{Ar}$**), 123.9 (2 C, **C$_{Ar}$**), 123.6 (1 C, **C$_{Ar}$**), 117.3 (1 C, **C$_{Ar}$**), 116.7 (1 C, **C$_{Ar}$**), 68.3 (1 C, -**C**H), 47.0 (1 C, -**C**H), 34.3 (2 C, **C$_q$**), 30.2 (6 C, -**C**$H_3$).

**HRMS** (ESI-TOF): *m/z:* $[M+H]^+$ calcd for $C_{40}H_{40}NO_3^+$ 582.3003 found 582.3009

## 4.6 Further transformations

The further transformations described herein were carried out with the product from the reaction between glycine Schiff base **1** and 1-naphthyl quinone methide **7o**. The starting material had a *dr* of 95:5 (calcd from crude NMR spectra and HPLC chromatogram) and an $er_{major}$ of 97:3. In any case, stereocenters were not affected by the transformation reactions.

### Peptide Synthesis

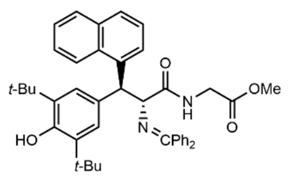

**9a**

α-functionalized *p*-$NO_2$ phenyl ester **8o** (35 mg, 0.05 mmol, 1 eq) was dissolved in 1 mL ACN. Then, DIPEA (8.7 µL, 0.05 mmol, 1 eq), DMAP (1.2 mg, 20 mol%) and methyl glycinate hydrochloride (12.6 mg, 0.1 mmol, 2 eq) were added. As soon as TLC (heptanes/EtOAc 5/1) indicated complete conversion, it was filtered over $Na_2SO_4$ and the solvent was evaporated. The crude product was purified by column chromatography on silica (heptanes/EtOAc 10/1 followed by 5/1 and 2/1) to yield dipeptide **9a** as a white solid (26.5 mg, 81%) without erosion of er and dr (*dr* 95:5, $er_{major}$ 97:3).

**HPLC** (YMC-SA, n-hexane/IPA = 10/1, flow = 0.5 mL min$^{-1}$, $T_{Column}$ = 10 °C, *l* = 240 nm): $t_R$(major diastereomer): 12.85 min (major), 14.70 min (minor).

$r_f$ (heptanes/EtOAc = 2/1) = 0.35

$[\alpha]_D^{24}$ (c 1, $CHCl_3$) = 45

**$^1$H-NMR** (300 MHz, $CDCl_3$, 298 K) *δ* / ppm = 8.07 (d, *J* = 8.5 Hz, 1 H, Ar-**H**), 7.96 (d, *J* = 8.0 Hz, 1 H, Ar-**H**), 7.67-7.61 (m, 3 H, Ar-**H**), 7.53 (s, 2 H, Ar-**H**), 7.42-7.29 (m, 7 H, Ar-**H**), 7.10-7.05 (m, 1 H, Ar-**H**), 6.99-6.88 (m, 3 H, Ar-**H**), 6.10 (br. s, 1 H, Ar-**H**), 5.59 (d, *J* = 5.3 Hz, 1 H, -C**H**), 5.06 (s, 1 H, -O**H**), 4.80 (d, *J* = 5.3 Hz, 1 H, -C**H**), 4.09 (dd, $J_1$ = 5.6 Hz, $J_2$ = 18.4 Hz, 1 H, -C$\mathbf{H_2}$), 3.75 (dd, $J_1$ = 4.6 Hz, $J_2$ = 18.4 Hz, 1 H, -C$\mathbf{H_2}$), 3.64 (s, 3 H, -OC$\mathbf{H_3}$), 1.41 (s, 18 H, -C$\mathbf{H_3}$).

**$^{13}$C-NMR** (75 MHz, $CDCl_3$, 298 K) *δ* / ppm = 172.2 (1 C, -**C**OOR), 171.7 (1 C, -**C**=N), 170.2 (1 C, -**C**OOR), 152.4 (1 C, $\mathbf{C_{Ar}}$), 139.5 (1 C, $\mathbf{C_{Ar}}$), 137.6 (1 C, $\mathbf{C_{Ar}}$), 135.2 (2 C, $\mathbf{C_{Ar}}$), 134.7 (1 C, $\mathbf{C_{Ar}}$), 134.0 (1 C, $\mathbf{C_{Ar}}$), 131.7 (1 C, $\mathbf{C_{Ar}}$), 131.3 (1 C, $\mathbf{C_{Ar}}$), 130.6 (1 C, $\mathbf{C_{Ar}}$), 129.0 (2 C, $\mathbf{C_{Ar}}$), 128.7 (1 C, $\mathbf{C_{Ar}}$), 128.2 (1 C, $\mathbf{C_{Ar}}$), 128.1 (2 C, $\mathbf{C_{Ar}}$), 127.9 (2 C, $\mathbf{C_{Ar}}$), 127.5 (1 C, $\mathbf{C_{Ar}}$), 127.1 (2 C, $\mathbf{C_{Ar}}$), 127.0 (2 C, $\mathbf{C_{Ar}}$), 127.0 (1 C, $\mathbf{C_{Ar}}$), 126.1 (1 C, $\mathbf{C_{Ar}}$), 125.3 (1 C, $\mathbf{C_{Ar}}$), 125.1 (1 C, $\mathbf{C_{Ar}}$), 123.2 (1 C, $\mathbf{C_{Ar}}$), 70.2 (1 C, -**C**H), 52.3 (1 C, -O**C**$H_3$), 50.5 (1 C, -**C**H), 41.1 (1 C, -**C**$H_2$), 34.5 (2 C, $\mathbf{C_q}$), 30.6 (6 C, -**C**$H_3$).

**HRMS** (ESI-TOF): *m/z:* [M+H]$^+$ calcd for $C_{43}H_{47}N_2O_4^+$ 655.3530 found 655.3530.

**Thiolester synthesis**

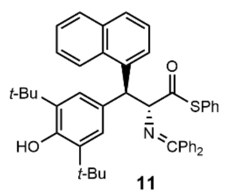
11

α-functionalized *p*-$NO_2$ phenyl ester **8o** (35 mg, 0.05 mmol, 1 eq) was dissolved in 1 mL ACN. Then, DMAP (1.2 mg, 20 mol%) and thiophenol (51 µL, 0.5 mmol, 10 eq) were added. The resulting mixture was stirred overnight, filtered over $Na_2SO_4$ and the solvents were evaporated. The crude product was purified by column chromatography on silica (heptanes followed by heptanes/EtOAc 10/1 and 5/1) to yield thiolester **11** as a white solid (25.7 mg, 76%) without erosion of er and dr (*dr* 95:5, $er_{major}$ 96:4).

**HPLC** (YMC-SA, n-hexane/IPA = 10/1, flow = 0.5 mL min$^{-1}$, $T_{Column}$ = 10 °C, *l* = 240 nm): $t_R$(major diastereomer): 8.25 min (minor), 8.88 min (major).

$\boldsymbol{r_f}$ (heptanes/EtOAc = 5/1) = 0.31

$[\boldsymbol{\alpha}]_{\boldsymbol{D}}^{\mathbf{24}}$ (c 1, $CHCl_3$) = 129

**$^1$H-NMR** (300 MHz, $CDCl_3$, 298 K) *δ* / ppm = 8.05 (d, *J* = 8.5 Hz, 1 H, Ar-**H**), 7.84 (d, *J* = 7.6 Hz, 1 H, Ar-**H**), 7.75-7.70 (m, 3 H, Ar-**H**), 7.57 (s, 2 H, Ar-**H**), 7.46-7.27 (m, 11 H, Ar-**H**), 7.17-6.94 (m, 5 H, Ar-**H**), 6.12 (br. s, 1 H, Ar-**H**), 5.71 (d, *J* = 6.2 Hz, 1 H, -C**H**), 5.09 (s, 1 H, -O**H**), 4.93 (d, *J* = 6.2 Hz, -C**H**), 1.42 (s, 18 H, -C**H$_3$**).

**$^{13}$C-NMR** (75 MHz, $CDCl_3$, 298 K) *δ* / ppm = 199.0 (1 C, -**C**OSPh), 172.0 (1 C, -**C**=N), 152.6 (1 C, **C$_{Ar}$**), 139.3 (1 C, **C$_{Ar}$**), 137.1 (1 C, **C$_{Ar}$**), 135.2 (2 C, **C$_{Ar}$**), 135.0 (1 C, **C$_{Ar}$**), 134.6 (2 C, **C$_{Ar}$**), 134.1 (1 C, **C$_{Ar}$**), 131.8 (1 C, **C$_{Ar}$**), 130.7 (1 C, **C$_{Ar}$**), 130.6 (1 C, **C$_{Ar}$**), 129.2 (2 C, **C$_{Ar}$**), 129.1 (3 C, **C$_{Ar}$**), 128.8 (1 C, **C$_{Ar}$**), 128.6 (1 C, **C$_{Ar}$**), 128.2 (1 C, **C$_{Ar}$**), 128.1 (2 C, **C$_{Ar}$**), 127.9 (2 C, **C$_{Ar}$**), 127.6 (1 C, **C$_{Ar}$**), 127.4 (2 C, **C$_{Ar}$**), 127.1 (3 C, **C$_{Ar}$**), 126.2 (1 C, **C$_{Ar}$**), 125.4 (1 C, **C$_{Ar}$**), 125.2 (1 C, **C$_{Ar}$**), 123.2 (1 C, **C$_{Ar}$**), 76.2 (1 C, -**C**H), 50.6 (1 C, -**C**H), 34.5 (2 C, **C$_q$**), 30.6 (6 C, -**C**$H_3$).

**HRMS** (ESI-TOF): *m/z:* $[M+H]^+$ calcd for $C_{46}H_{46}NO_2S^+$ 676.3244 found 676.3243.

**Amide synthesis**

α-functionalized *p*-$NO_2$ phenyl ester **8o** (35 mg, 0.05 mmol, 1 eq) was dissolved in 1 mL ACN. Then, the respective amine (benzylamine: 11 µL, 0.1 mmol, 2 eq; p-Cl benzylamine: 10.5 µL, 0.1 mmol, 2 eq; morpholine: 43 µL, 0.5 mmol, 10 eq) and DMAP (1.2 mg, 20 mol%) were added. The resulting mixtures were stirred for 3 h, filtered over $Na_2SO_4$ and the solvents were evaporated. The crude products were purified by column chromatography on deactivated silica (heptanes/EtOAc 10/1 followed by 5/1 and 2/1) to yield the pure amides.

**Benzylamide 9c**

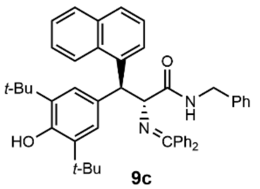


**9c**

Obtained as a white solid in a yield of 23.6 mg (70%) without erosion of er and dr (*dr* 95:5, $er_{major}$ 96:4).

**HPLC** (YMC-SA, n-hexane/IPA = 10/1, flow = 0.5 mL min$^{-1}$, $T_{Column}$ = 10 °C, $l$ = 240 nm ): $t_R$(major diastereomer): 15.46 min (major), 16.98 min (minor).

$r_f$ (heptanes/EtOAc = 2/1) = 0.50.

$[\alpha]_D^{24}$ (c 1, CHCl$_3$) = 83

**$^1$H-NMR** (300 MHz, CDCl$_3$, 298 K) $\delta$ / ppm = 8.13 (d, $J$ = 8.5 Hz, 1 H, Ar-**H**), 7.82 (d, $J$ = 7.7 Hz, 1 H, Ar-**H**), 7.68 (d, $J$ = 7.7 Hz, 1 H, Ar-**H**), 7.60-7.58 (m, 4 H, Ar-**H**), 7.44-7.29 (m, 7 H, Ar-**H**), 7.14-7.03 (m, 4 H, Ar-**H**), 6.89-6.76 (m, 5 H, Ar-**H**), 6.02 (br. s, 1 H, Ar-**H**), 5.63 (d, $J$ = 5.1 Hz, 1 H, -C**H**), 5.13 (s, 1 H, -O**H**), 4.86 (d, $J$ = 5.1 Hz, 1 H, -C**H$_3$**), 4.62 (dd, $J_1$ = 7.4 Hz, $J_2$ = 15.3 Hz, 1 H, -C**H$_2$**), 4.14 (dd, $J_1$ = 4.9 Hz, $J_2$ = 15.3 Hz), 1.40 (s, 18 H, -C**H$_3$**).

**$^{13}$C-NMR** (75 MHz, CDCl$_3$, 298 K) $\delta$ / ppm = 172.0 (1 C, -**C**ONHR), 171.3 (1 C, -**C**=N), 152.7 (1 C, **C$_{Ar}$**), 139.5 (1 C, **C$_{Ar}$**), 138.3 (1 C, **C$_{Ar}$**), 137.5 (1 C, **C$_{Ar}$**), 135.3 (2 C, **C$_{Ar}$**), 134.7 (1 C, **C$_{Ar}$**), 134.1 (1 C, **C$_{Ar}$**), 131.8 (1 C, **C$_{Ar}$**), 131.1 (1 C, **C$_{Ar}$**), 130.6 (1 C, **C$_{Ar}$**), 128.9 (2 C, **C$_{Ar}$**), 128.8 (1 C, **C$_{Ar}$**), 128.6 (2 C, **C$_{Ar}$**), 128.1 (3 C, **C$_{Ar}$**), 127.9 (2 C, **C$_{Ar}$**), 127.5 (2 C, **C$_{Ar}$**), 127.3 (1 C, **C$_{Ar}$**), 127.1 (2 C, **C$_{Ar}$**), 127.1 (2 C, **C$_{Ar}$**), 127.0 (2 C, **C$_{Ar}$**), 126.2 (1 C, **C$_{Ar}$**), 125.3 (1 C, **C$_{Ar}$**), 125.2 (1 C, **C$_{Ar}$**), 123.3 (1 C, **C$_{Ar}$**), 70.9 (1 C, -**C**H), 51.1 (1 C, -**C**H$_2$), 43.0 (1 C, -**C**H), 34.5 (2 C, **C$_q$**), 30.7 (6 C, -**C**H$_3$).

**HRMS** (ESI-TOF): *m/z:* [M+H]$^+$ calcd for $C_{47}H_{49}N_2O_2^+$ 673.3789 found 673.3788.

***p*-Cl Benzylamide 9d**

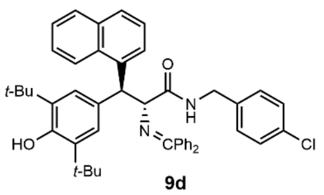


**9d**

Obtained as a white solid in a yield of 30.5 mg (86%) without erosion of er and dr (*dr* 95:5, $er_{major}$ 97:3).

**HPLC** (YMC-SA, n-hexane/IPA = 10/1, flow = 0.5 mL min$^{-1}$, $T_{Column}$ = 10 °C, $l$ = 240 nm ): $t_R$(major diastereomer): 13.39 min (minor); 16.09 min (major).

$r_f$ (heptanes/EtOAc = 2/1) = 0.49

$[\alpha]_D^{24}$ (c 1, CHCl$_3$) = 16

**$^1$H-NMR** (300 MHz, CDCl$_3$, 298 K) $\delta$ / ppm = 8.10 (d, $J$ = 8.5 Hz, 1 H, Ar-**H**), 7.82 (d, $J$ = 7.3 Hz, 1 H, Ar-**H**), 7.68 (d, $J$ = 7.7 Hz, 1 H, Ar-**H**), 7.61-7.58 (m, 4 H, Ar-**H**), 7.44-7.28 (m, 7 H, Ar-**H**), 7.07-7.01 (m, 1 H, Ar-**H**), 7.03 (d, $J$ = 8.5 Hz, 2 H, Ar-**H**), 6.87-6.83 (m, 3 H, Ar-**H**), 6.62 (d, $J$ = 8.5 Hz, 2 H, Ar-**H**), 6.00 (br. s, 1 H, Ar-**H**), 5.60 (d, $J$ = 5.1 Hz, 1 H, -C**H**), 5.16 (s, 1 H, -O**H**), 4.86 (d, $J$ = 5.1 Hz, 1 H, -C**H**), 4.67 (dd, $J_1$ = 7.9 Hz, $J_2$ = 15.4 Hz, 1 H, -C**H$_2$**), 3.99 (dd, $J_1$ = 4.7 Hz, $J_2$ = 15.4 Hz, 1 H, -C**H$_2$**), 1.40 (s, 18 H, -C**H$_3$**).

**$^{13}$C-NMR** (75 MHz, $CDCl_3$, 298 K) $\delta$ / ppm = 172.1 (1 C, -**C**ONHR), 171.5 (1 C, -**C**=N), 152.7 (1 C, **$C_{Ar}$**), 139.5 (1 C, **$C_{Ar}$**), 137.2 (1 C, **$C_{Ar}$**), 136.9 (1 C, **$C_{Ar}$**), 135.3 (2 C, **$C_{Ar}$**), 134.6 (1 C, **$C_{Ar}$**), 134.1 (1 C, **$C_{Ar}$**), 132.7 (1 C, **$C_{Ar}$**), 131.7 (1 C, **$C_{Ar}$**), 130.9 (1 C, **$C_{Ar}$**), 130.7 (1 C, **$C_{Ar}$**), 128.9 (2 C, **$C_{Ar}$**), 128.8 (1 C, **$C_{Ar}$**), 128.7 (2 C, **$C_{Ar}$**), 128.4 (2 C, **$C_{Ar}$**), 128.1 (3 C, **$C_{Ar}$**), 127.9 (2 C, **$C_{Ar}$**), 127.5 (2 C, **$C_{Ar}$**), 127.3 (1 C, **$C_{Ar}$**), 127.1 (1 C, **$C_{Ar}$**), 127.0 (2 C, **$C_{Ar}$**), 126.2 (1 C, **$C_{Ar}$**), 125.4 (1 C, **$C_{Ar}$**), 125.2 (1 C, **$C_{Ar}$**), 123.1 (1 C, **$C_{Ar}$**), 70.8 (1 C, -**C**H), 51.2 (1 C, -**C**$H_2$), 42.3 (1 C, -**C**H), 34.5 (2 C, **$C_q$**), 30.6 (6 C, -**C**$H_3$).

**HRMS** (ESI-TOF): *m/z:* $[M+H]^+$ calcd for $C_{47}H_{48}ClN_2O_2^+$ 707.3399 found 707.3399.

### Morpholine amide 9b

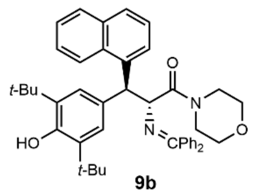

**9b**

Obtained as a white solid in a yield of 26.1 mg (80%) erosion of er and dr (*dr* 95:5, $er_{major}$ 97:3).

**HPLC** (YMC-SA, n-hexane/IPA = 10/1, flow = 0.5 mL $min^{-1}$, $T_{Column}$ = 10 °C, *l* = 272 nm ): $t_R$(major diastereomer): 17.68 min (major), 32.23 min (minor).

$r_f$ (heptanes/EtOAc = 2/1) = 0.28

$[\alpha]_D^{24}$ (c 1, $CHCl_3$) = 201

**$^1$H-NMR** (300 MHz, $CDCl_3$, 298 K) $\delta$ / ppm = 8.25 (d, *J* = 8.2 Hz, 1 H, Ar-**H**), 7.81-7.77 (m, 1 H, Ar-**H**), 7.71-7.68 (m, 1 H, Ar-**H**), 7.60-7.58 (m, 2 H, Ar-**H**), 7.48-7.28 (m, 10 H, Ar-**H**), 7.06 (s, 2 H, Ar-**H**), 6.45 (br. s 2 H, Ar-**H**), 5.76 (d, *J* = 9.6 Hz, 1 H, -C**H**), 5.09 (d, *J* = 9.6 Hz, 1 H, -C**H**), 4.99 (s, 1 H, -O**H**), 3.42-3.13 (br. m, 8 H, -C**$H_2$**), 1.22 (s, 18 H, -C**$H_3$**).

**$^{13}$C-NMR** (75 MHz, $CDCl_3$, 298 K) $\delta$ / ppm = 169.7 (1 C, -**C**$ONR_2$), 169.0 (1 C, -**C**=N), 152.5 (1 C, **$C_{Ar}$**), 139.5 (1 C, **$C_{Ar}$**), 137.2 (1 C, **$C_{Ar}$**), 136.5 (1 C, **$C_{Ar}$**), 135.3 (2 C, **$C_{Ar}$**), 134.3 (1 C, **$C_{Ar}$**), 132.3 (1 C, **$C_{Ar}$**), 131.3 (1 C, **$C_{Ar}$**), 130.4 (1 C, **$C_{Ar}$**), 129.0 (2 C, **$C_{Ar}$**), 228.8 (1 C, **$C_{Ar}$**), 128.7 (1 C, **$C_{Ar}$**), 128.3 (2 C, **$C_{Ar}$**), 128.0 (2 C, **$C_{Ar}$**), 127.9 (2 C, **$C_{Ar}$**), 127.5 (1 C, **$C_{Ar}$**), 126.4 (2 C, **$C_{Ar}$**), 126.3 (1 C, **$C_{Ar}$**), 125.6 (1 C, **$C_{Ar}$**), 125.1 (1 C, **$C_{Ar}$**), 124.6 (1 C, **$C_{Ar}$**), 124.3 (1 C, **$C_{Ar}$**), 69.4 (1 C, -**C**H), 66.8 (1 C, -**C**$H_2$), 66.6 (1 C, -**C**$H_2$), 50.3 (1 C, -**C**$H_2$), 46.0 (1 C, -**C**$H_2$), 42.6 (1 C, -**C**H), 34.3 (2 C, **$C_q$**), 30.3 (6 C, -**C**$H_3$).

**HRMS** (ESI-TOF): *m/z:* $[M+H]^+$ calcd for $C_{44}H_{49}N_2O_3^+$ 653.3738 found 653.3731.

### Methyl ester synthesis

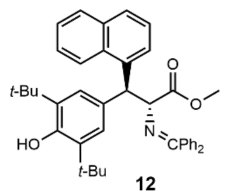

**12**

α-functionalized p-$NO_2$ phenyl ester **8o** (35 mg, 0.05 mmol, 1 eq) was dissolved in 5 mL MeOH and DMAP (40 mol%, 4.9 mg) was added. The mixture was stirred at rt for 24 h and then the mixture was concentrated on the rotary evaporator. The crude was submitted to column chromatography on silica (heptanes/EtOAc 10/1 followed by 5/1) to give methyl ester **12** as an off-white residue in quantitative yield (30 mg, *dr* 96:4, *er* 96:4).

**HPLC:** (YMC-SA, n-hexane/IPA = 10/1, flow = 0.5 mL min$^{-1}$, $T_{Column}$ = 10 °C, *l* = 272 nm ): $t_R$(major diastereomer): 10.14 min (major), 10.69 min (minor).

$r_f$ (heptanes/EtOAc = 10/1) = 0.24

$[\alpha]_D^{24}$ (c 1, $CHCl_3$) = 156

**$^1$H-NMR** (300 MHz, $CDCl_3$, 298 K) $\delta$ / ppm = 8.23-8.20 (m, 1 H, Ar-**H**), 7.83-7.78 (m, 1 H, Ar-**H**), 7.68 (d, *J* = 7.7 Hz, 1 H, Ar-**H**), 7.62-7.59 (m, 2 H, Ar-**H**), 7.51-7.28 (m, 11 H, Ar-**H**), 7.09 (s, 2 H, Ar-**H**), 6.45 (br.s, 1 H, Ar-**H**), 5.62 (d, *J* = 9.4 Hz, 1 H, -C**H**), 4.97 (s, 1 H, -O**H**), 4.89 (d, *J* = 9.4 Hz, 1 H, -C**H**), 3.47 (s, 3 H, -OC**H₃**), 1.26 (s, 18 H, -C**H₃**).

**$^{13}$C-NMR** (75 MHz, $CDCl_3$, 298 K) $\delta$ / ppm = 171.9 (1 C, -**C**OOR), 171.0 (1 C, -**C**=N), 152.4 (1 C, $\mathbf{C_{Ar}}$), 139.6 (1 C, $\mathbf{C_{Ar}}$), 137.6 (1 C, $\mathbf{C_{Ar}}$), 136.0 (1 C, $\mathbf{C_{Ar}}$), 135.2 (2 C, $\mathbf{C_{Ar}}$), 134.2 (1 C, $\mathbf{C_{Ar}}$), 132.1 (1 C, $\mathbf{C_{Ar}}$), 131.0 (1 C, $\mathbf{C_{Ar}}$), 130.4 (1 C, $\mathbf{C_{Ar}}$), 130.2 (1 C, $\mathbf{C_{Ar}}$), 129.1 (2 C, $\mathbf{C_{Ar}}$), 128.7 (1 C, $\mathbf{C_{Ar}}$), 128.4 (2 C, $\mathbf{C_{Ar}}$), 128.1 (2 C, $\mathbf{C_{Ar}}$), 128.0 (2 C, $\mathbf{C_{Ar}}$), 128.0 (2 C, $\mathbf{C_{Ar}}$), 127.2 (1 C, $\mathbf{C_{Ar}}$), 126.2 (2 C, $\mathbf{C_{Ar}}$), 126.1 (1 C, $\mathbf{C_{Ar}}$), 125.4 (1 C, $\mathbf{C_{Ar}}$), 125.0 (1 C, $\mathbf{C_{Ar}}$), 124.5 (1 C, $\mathbf{C_{Ar}}$), 124.2 (1 C, $\mathbf{C_{Ar}}$), 71.3 (1 C, -**C**H), 52.2 (1 C, -O**C**H₃), 49.6 (1 C, -**C**H), 34.3 (2 C, $\mathbf{C_q}$), 30.3 (6 C, -**C**H₃).

**HRMS** (ESI-TOF): *m/z:* $[M+H]^+$ calcd for $C_{41}H_{44}NO_3^+$: 598.3316, found 598.3318.

**Imine Hydrolysis**

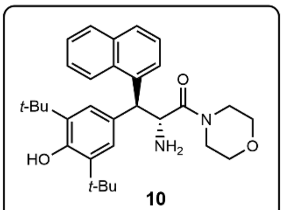

**10**

According to literature [39], morpholine amide **9b** (65.3 mg, 0.1 mmol *dr* 96:4 $er_{major}$ 96:4), was dissolved in 2 mL THF (0.05 mol L$^{-1}$) and cooled to 0 °C. Then, 2 mL 1 M HCl were added dropwise and the mixture was stirred at r.t. overnight. The reaction was quenched with 4 mL of a saturated $NaHCO_3$ solution and extracted with DCM three times. The combined organic layers were dried over $Na_2SO_4$, filtered, and then concentrated on the rotary evaporator. The crude product was purified by column chromatography on silica (heptanes/EtOAc 2/1 followed by pure EtOAc, and then flushing the column with MeOH to elute remaining product). Amine **10** was obtained as a white foam in a yield of 43.8 mg (90%).

**HPLC** (YMC-SA, *n*-hexane/IPA = 1/1, flow = 1.0 mL min$^{-1}$, $T_{Column}$ = 10 °C, *l* = 240 nm ): $t_R$(major diastereomer): 9.40 min (major), 12.87 min (minor).

$r_f$ (EtOAc/MeOH 1/1) = 0.20

$[\alpha]_D^{24}$ (c 1, $CHCl_3$) = -262

**$^1$H-NMR** (300 MHz, $CDCl_3$, 298 K) $\delta$ / ppm = 8.32 (d, *J* = 8.5 Hz, 1 H, Ar-**H**), 7.81 (dd, $J_1$ = 1.3 Hz, $J_2$ = 8.0 Hz, 1 H, Ar-**H**), 7.70 (d, *J* = 8.0 Hz, 1 H, Ar-**H**), 7.56-7.35 (m, 4 H, Ar-**H**), 7.18 (s, 2 H, Ar-**H**), 5.15 (br. 1 H, -O**H**), 5.04 (d, *J* = 9.9 Hz, 1 H, -C**H**), 4.49 (d, *J* = 9.9 Hz, 1 H, -C**H**), 3.35-3.12 (m, 5 H, -C**H₂**), 2.88-2.83 (m, 1 H, -C**H₂**), 2.61-2.55 (m, 1 H, -C**H₂**), 2.46-2.40 (m, 1 H, -C**H₂**), 2.00 (br., 2 H, -N**H₂**), 1.42 (s, 18 H, -C**H₃**).

**$^{13}$C-NMR** (75 MHz, $CDCl_3$, 298 K) $\delta$ / ppm = 172.4 (1 C, -**C**$NR_2$), 152.8 (1 C, -$\mathbf{C}_{\mathbf{Ar}}$), 138.9 (1 C, -$\mathbf{C}_{\mathbf{Ar}}$), 136.4 (2 C, -$\mathbf{C}_{\mathbf{Ar}}$), 134.0 (1 C, -$\mathbf{C}_{\mathbf{Ar}}$), 132.0 (1 C, -$\mathbf{C}_{\mathbf{Ar}}$), 131.4 (1 C, -$\mathbf{C}_{\mathbf{Ar}}$), 128.7 (1 C, -$\mathbf{C}_{\mathbf{Ar}}$), 127.4 (1 C, -$\mathbf{C}_{\mathbf{Ar}}$), 126.2 (1 C, -$\mathbf{C}_{\mathbf{Ar}}$), 125.8 (1 C, -$\mathbf{C}_{\mathbf{Ar}}$), 125.6 (2 C, -$\mathbf{C}_{\mathbf{Ar}}$), 125.2 (1 C, -$\mathbf{C}_{\mathbf{Ar}}$), 125.1 (1 C, -$\mathbf{C}_{\mathbf{Ar}}$), 124.0 (1 C, -$\mathbf{C}_{\mathbf{Ar}}$), 66.2 (1 C, -**C**$H_2$), 65.9 (1 C, -**C**$H_2$), 54.8 (1 C, -**C**H), 52.1 (1 C, -**C**H), 46.0 (1 C, -**C**$H_2$), 42.1 (1 C, -**C**$H_2$), 34.5 (2 C, $\mathbf{C}_{\mathbf{q}}$), 30.4 (6 C, -**C**$H_3$).

**HRMS** (ESI-TOF): *m/z:* $[M+H]^+$ calcd for $C_{31}H_{41}N_2O_3^+$ 489.3112 found 489.3110.

## 4.7 Relative and Absolute Configuration of p-QM-based 1,6-addition products

In order to determine relative and absolute configuration of the alkylation products, comparison with literature and X-ray crystallography was performed. As can be seen from figure 1, methyl ester **12b** was synthesized according to the general protocols. The obtained $^{1}$H-NMR spectrum was compared with the $^{1}$H-NMR spectrum of the same compound obtained following Zhang's protocol [40]. As the configuration of the major diastereomer was assigned to be like (R/R as major enantiomer) by Zhang et al., also the like diastereomer should be the major enantiomer following the protocol reported in here. Also, the same (+) rotation supports that the absolute configuration is the same as reported by Zhang.

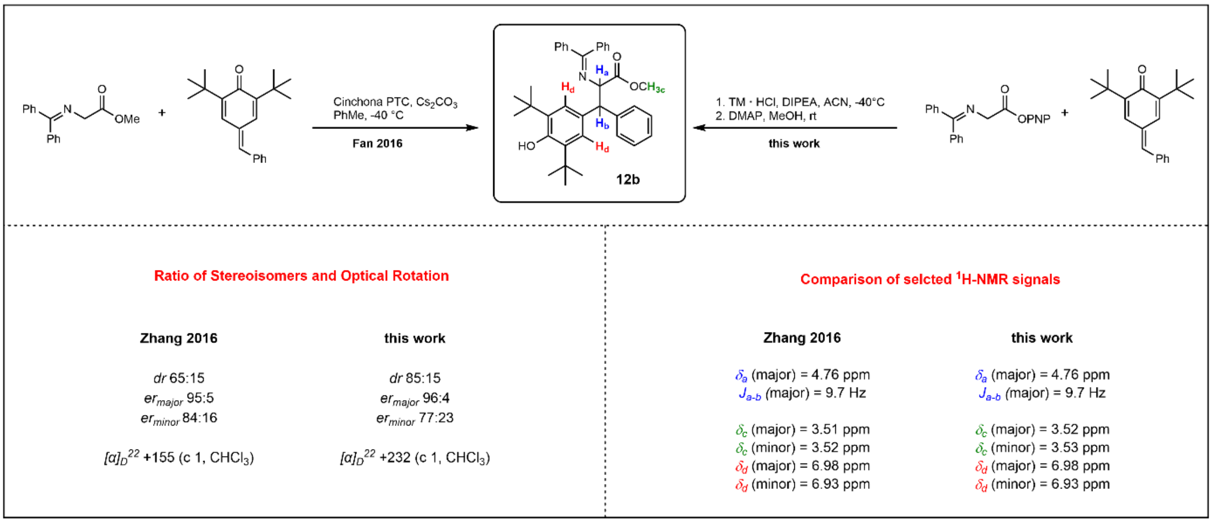


**Figure 1:** Comparison of NMR data and optical rotation of methyl ester **12b** with Zhang's work.

To further proof the absolute configuration, X-ray crystallography was performed. Compound **9d** was crystallized by dissolving the compound in DCM and carefully layering with $Et_2O$ followed by slow evaporation of the solvents. Single-crystal structure analysis was carried out at room temperature on a Bruker D8 Quest ECO diffractometer with graphite-monochromated MoKα radiation (λ = 0.71073 Å). The structures were solved by direct methods (SHELXS-2013/1 [41] and refined by full-matrix least-squares on $F^2$ (SHELXL-2018/3 [42]). The H atoms were calculated geometrically, and a riding model was applied in the refinement process. Crystallographic details for **9d** can be found in Table 1. CCDC 2239320 contain the supplementary crystallographic data. This information can be obtained free of charge via https://www.ccdc.cam.ac.uk/structures/

**Table 1.** Crystal data for the structures of **9d**.

| Compound | **9d** |
|---|---|
| Empirical formula | $C_{47}H_{47}ClN_2O_2$ |
| Formula weight | 707.31 |
| Crystal system | orthorhombic |
| Space group | $P2_12_12_1$ |
| Temp/K | 296 |
| *a* (Å) | 9.949(5) |
| *b* (Å) | 19.830(9) |
| *c* (Å) | 20.437(8) |
| *α* (°) | 90 |
| *β* (°) | 90 |
| *γ* (°) | 90 |
| $V$ (Å$^3$) | 4032(2) |
| *Z* | 4 |
| $D_{calc}$ (g/cm$^3$) | 1.168 |
| Reflns collected | 81072 |
| Indep. reflns | 7028 |
| Obs. reflns [I > 2σ(I)] | 4901 |
| Param. refin./restr. | 476 |
| Absorption correction | multi-scan |
| Flack parameter | 0.07(2) |
| $R_1$ | 0.047 |
| $wR_2$ | 0.106 |
| CCDC | 2239320 |

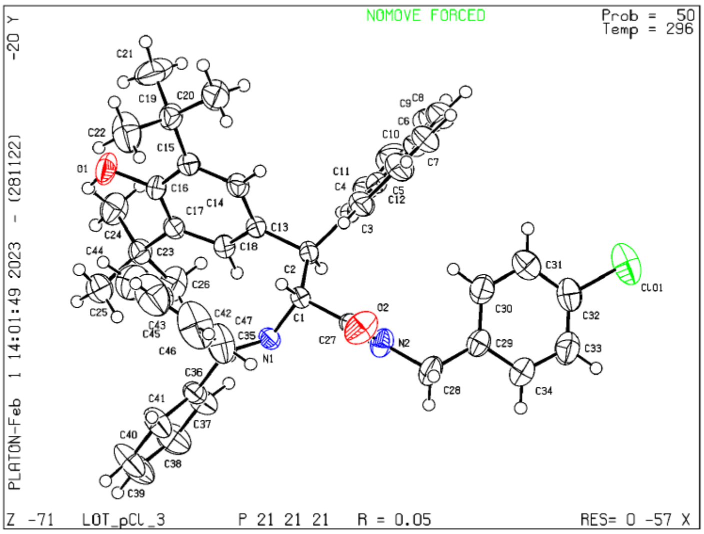

# 5 1,4-Conjugate additions of Glycine Schiff base aryl ester to Michael acceptors

## 5.1 General procedure G: Michael addition reactions of Glycine Schiff base esters

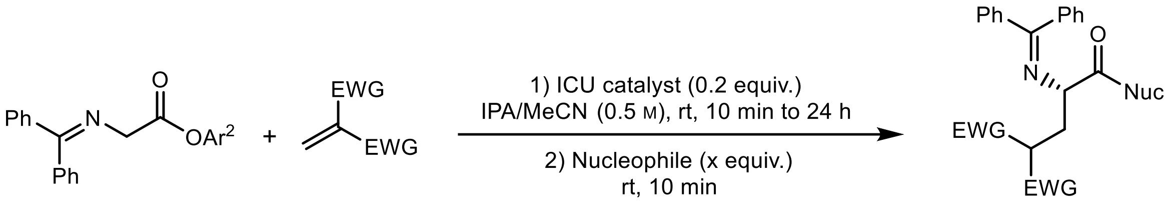


The Schiff base (1.0 equiv.), the Michael acceptor (1.0 equiv.) and the catalyst (0.2 equiv.) were added to a flame dried vial. IPA (0.5 M) and MeCN (1.3 M) were then added, and the reaction was stirred at rt and monitored by $^1$H NMR. When all the Michael acceptor was consumed, the nucleophile was added, and the reaction was stirred for 10 min at rt. The reaction mixture was diluted with EtOAc (40 volumes) and aq. NaOH 1 M solution (40 volumes). The aq. layer was extracted with EtOAc (×2 vol). The organic layer was then washed with aq. NaOH 1 M solution (×2 vol), brine (×1 vol), dried ($MgSO_4$), filtered, and concentrated *in vacuo*. The crude residue was purified by column chromatography using the solvent system stated.

## 5.2 General procedure H: Michael addition reactions of Glycine Schiff base esters using slow addition

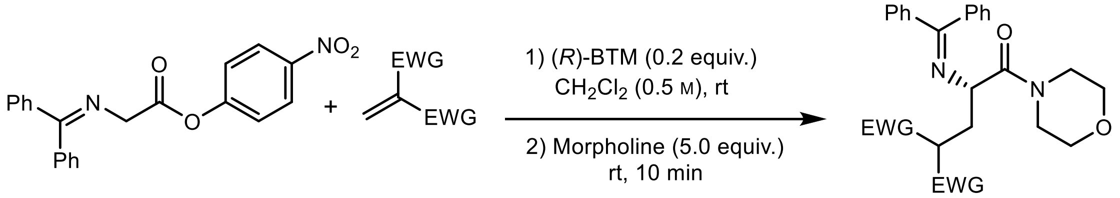


The Schiff base (1.5 equiv.) and (*R*)-BTM (0.05 equiv.) were dissolved in $CH_2Cl_2$ (3 M in Schiff base). The electrophile (1.0 equiv.) was dissolved in $CH_2Cl_2$ (0.67 M) and the solution was slowly added to the reaction mixture via syringe pump over the requisite time. Half the previous volume of $CH_2Cl_2$ was added to the syringe and it was added to the reaction mixture over half the previous time. The reaction was then left to stir the same amount of time at rt. Morpholine (5.0 equiv.) was then added, and the reaction was stirred for 10 min at rt. The reaction mixture was diluted with EtOAc (40 volumes) and aq. NaOH 1 M solution (40 volumes). The aq. layer was extracted with EtOAc (×2 vol). The organic layer was then washed with aq. NaOH 1M solution (×2 vol) and brine (×1 vol) then dried ($MgSO_4$), filtered, and concentrated *in vacuo*. The crude residue was purified by column chromatography using the solvent system stated.

## 5.3 Products of asymmetric 1,4-additions of Glycine Schiff base aryl ester to Michael acceptors

### (*S*)-*N*-Benzyl-2-((diphenylmethylene)amino)-4,4-bis(phenylsulfonyl)butanamide (17)

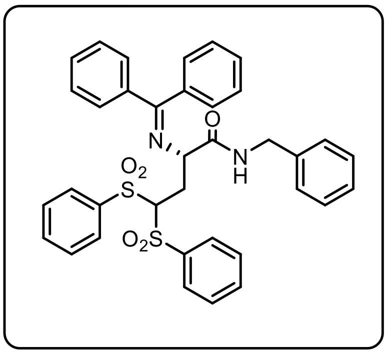

Following general procedure G, 4-nitrophenyl 2-((diphenylmethylene)amino)acetate **1** (72.1 mg, 0.2 mmol, 1.5 equiv.), (ethene-1,1-diyldisulfonyl)dibenzene **16** (40.1 mg, 0.13 mmol), (*R*)-BTM (6.6 mg, 0.026 mmol) and $CH_2Cl_2$ (260 µL) at rt for 24 h, then benzylamine (14 µL, 0.13 mmol, 1 equiv.) for 24 h at rt, gave after purification by column chromatography (30:70, $Et_2O$:$CH_2Cl_2$, $R_f$ = 0.8), the title compound (58 mg, 70%) as a pale yellow solid.

**HPLC analysis:** Chiralcel OD-H (90:10 hexane:IPA, flow rate 2 mL/min, 254 nm, 30 °C) $t_R$: 37.2 min, $t_R$: 45.8 min, 7:93 er

$[\alpha]_D^{20}$ –14.1 (*c* 0.5, $CHCl_3$)

**$^1$H-NMR** (400 MHz, $CD_2Cl_2$) $\delta_H$: 2.46-2.57 (1H, m, C**H$^a$**HCH($SO_2$Ph)$_2$), 2.74-2.83 (1H, m, CH**H$^b$**CH($SO_2$Ph)$_2$), 4.29-4.45 (3H, m, NHC**H**$_2$Ph, C**H**CONH), 5.52 (1H, dd, *J* 7.0, 3.6, $CH_2$C**H**($SO_2$Ph)$_2$), 7.15-7.20 (2H, m, C**H** Ph ×2), 7.26-7.33 (3H, m, C**H** Ph ×3), 7.34-7.42 (4H, C**H** Ph ×4), 7.45-7.58 (8H, m, C**H** Ph ×8), 7.58-7.63 (2H, m, C**H** Ph ×2), 7.65-7.72 (2H, m, C**H** Ph ×2), 7.76-7.80 (2H, m, C**H** Ph ×2), 7.80-7.85 (2H, m, C**H** Ph ×2)

**$^{13}$C-NMR** (126 MHz, $CD_2Cl_2$) $\delta_C$: 29.9 (**C**$H_2$CH($SO_2$Ph)$_2$), 42.8 (NH**C**$H_2$Ph), 62.7 (C**H**CONH), 78.3 ($CH_2$**C**H($SO_2$Ph)$_2$), 127.3 (**C**H Ph), 127.7 (**C**H Ph ×2), 127.7 (**C**H Ph ×2), 128.2 (**C**H Ph ×4), 128.6 (**C**H Ph), 128.8 (**C**H Ph ×2), 128.9 (**C**H Ph ×2), 129.0 (**C**H Ph ×2), 129.0 (**C**H Ph ×2), 129.0 (C**H** Ph ×2), 129.3 (**C**H Ph ×2), 129.4 (**C**H Ph), 131.1 (**C**H Ph), 134.3 (**C**H Ph), 134.4 (**C**H Ph), 135.1 (**C** Ph), 137.8 (**C** Ph), 138.3 (**C** Ph), 138.5 (**C** Ph), 138.8 (**C** Ph), 171.4 (**C**=O), 172.0 (**C**=N)

**HRMS** (ESI$^+$) $C_{36}H_{32}N_2O_5S_2$ [M+H]$^+$ found 637.1812 requires 637.1825 (–2.0 ppm)

**Melting point:** 74 °C ($Et_2O$/$CH_2Cl_2$)

**(*S*)-2-Amino-*N*-benzyl-4,4-bis(phenylsulfonyl)butanamide (18)**

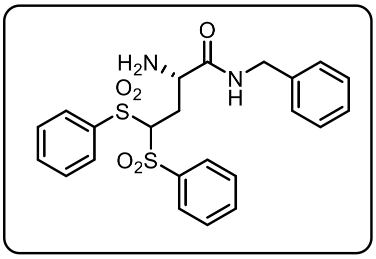


Following general procedure G, 4-nitrophenyl 2-((diphenylmethylene)amino)acetate **1** (72.1 mg, 0.2 mmol, 1.5 equiv.), (ethene-1,1-diyldisulfonyl)dibenzene **16** (40.1 mg, 0.13 mmol), (*R*)-BTM (1.8 mg, 0.0065 mmol, 0.05 equiv.) and $CH_2Cl_2$ (260 µL) at rt for 24 h, then benzylamine (71 µL, 0.65 mmol, 5.0 equiv.) for 24 h at rt, gave after purification by column chromatography (50:50, $Et_2O$:$CH_2Cl_2$, $R_f$ = 0.1), the title compound (38.6 mg, 63%) as a yellow oil.

**HPLC analysis:** Chiralcel OD-H (80:20 hexane:IPA, flow rate 2 mL/min, 254 nm, 30 °C) $t_R$: 40.3 min, $t_R$: 49.5 min, 92:8 er

$[\alpha]_D^{20}$ +23.5 (*c* 0.5, $CHCl_3$)

**$^1$H-NMR** (400 MHz, $CDCl_3$) $\delta_H$: 2.34-2.43 (1H, m, C**H$^a$**HCH($SO_2$Ph)$_2$), 2.52-2.60 (1H, m, CH**H$^b$**CH($SO_2$Ph)$_2$), 3.79 (1H, t, *J* 7.49, $NH_2$C**H**CO), 4.32 (1H, dd, *J* 14.97, 5.96, C**H$^a$**HPh), 4.39 (1H, dd, *J* 14.65, 6.28, CH**H$^b$**Ph), 5.49 (1H, t, *J* 5.52, $CH_2$C**H**($SO_2$Ph)$_2$), 7.22-7.37 (5H, m, C**H** Ph ×5), 7.5-7.57 (4H, m, C**H** Ph ×4), 7.63-7.70 (2H, m, C**H** Ph ×2), 7.88-7.95 (4H, m, C**H** Ph ×4)

**$^{13}$C-NMR** 126 MHz, $CDCl_3$) $\delta_C$: 31.3 (**C**$H_2$CH($SO_2$Ph)$_2$), 43.3 (**C**$H_2$Ph), 52.6 ($NH_2$**C**HCO), 79.6 ($CH_2$**C**H($SO_2$Ph)$_2$) 127.6 (**C**H Ph), 127.8 (**C**H Ph ×2), 128.8 (**C**H Ph ×2), 129.1 (**C**H Ph ×2), 129.2 (**C**H Ph ×2), 129.5 (**C**H Ph ×2), 129.6 (**C**H Ph ×2), 134.5 (**C**H Ph ×2), 137.7 (**C** Ph), 137.9 (**C** Ph), 138.0 (**C** Ph), 173.4 (**C**=O)

**HMRS** (ESI$^+$) $C_{23}H_{24}N_2O_5S_2$ $[M+H]^+$ found 473.1186 requires 473.1199 (–2.7 ppm)

**IR $\nu_{max}$** (film) 1655 (C=O amide), 1312 and 1327 (S=O), 1148 (C-N amine), 1078 (C-N amide)

**(*S*)-2-((Diphenylmethylene)amino)-1-morpholino-4,4-bis(phenylsulfonyl)butan-1-one (20a)**

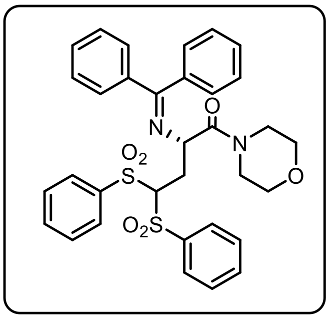

Following general procedure H, 4-nitrophenyl 2-((diphenylmethylene)amino)acetate **1** (108.1 mg, 0.3 mmol), and (*R*)-BTM (2.5 mg, 0.01 mmol) in $CH_2Cl_2$ (100 µL) and (ethene-1,1-diyldisulfonyl)dibenzene **16** (69.7 mg, 0.2 mmol, 1 equiv.) in $CH_2Cl_2$ (300 µL) for 1 h at rt, then $CH_2Cl_2$ (200 µL) for 1 h at rt, and finally morpholine (87 µL, 1.0 mmol) for 10 min at rt, gave after purification by column chromatography (30:70, $Et_2O$:$CH_2Cl_2$, $R_f$ = 0.4), the title compound (47 mg, 89%) as a pale yellow solid.

**HPLC analysis:** Chiralcel OD-H (80:20 hexane:IPA, flow rate 1 mL/min, 254 nm, 40 °C) $t_R$: 18.9 min, $t_R$: 21.3 min, 2:98 er

$[\alpha]_D^{20}$ +60.9 (*c* 1.0, $CHCl_3$)

**$^1$H-NMR** (500 MHz, $CD_2Cl_2$) $\delta_H$: 2.62-2.70 (1H, m, C**H**$^a$HCH($SO_2$Ph)$_2$), 2.72-2.80 (1H, m, CH**H**$^b$CH($SO_2$Ph)$_2$), 2.98-3.06 (1H, m, N(C**H**$^a$H)($CH_2$)O), 3.24-3.31 (1H, N($CH_2$)(C**H**$^a$H)O), 3.31-3.54 (5H, m, N(CH**H**$^b$)($CH_2$)O, N(C**H**$_2$)($CH_2$)O, N($CH_2$)(CH**H**$^b$)O and N($CH_2$)(C**H**$^a$H)O), 3.55-3.62 (1H, m, N($CH_2$)(CH**H**$^b$)O), 4.79 (1H, dd, *J* 9.5, 4.8, C**H**CO), 5.09 (1H, dd, *J* 7.8, 3.6, $CH_2$C**H**($SO_2$Ph)$_2$), 7.13-7.18 (2H, m, C**H** Ph ×2), 7.35 (2H, app t, *J* 7.5, C**H** Ph ×2), 7.42-7.49 (3H, m, C**H** Ph ×3), 7.51-7.57 (3H, m, C**H** Ph ×3), 7.60 (2H, app t, *J* 8.0, C**H** Ph ×2), 7.64 (2H, app t, *J* 7.4, C**H** Ph×2), 7.75 (1H, app t, *J* 7.6, C**H** Ph), 7.78 (1H, app t, *J* 7.8, C**H** Ph), 7.87-7.95 (4H, m, C(2)**H** Ph ×4)

**$^{13}$C-NMR** (126 MHz, $CD_2Cl_2$) $\delta_C$: 30.3 (**C**$H_2$CH($SO_2$Ph)$_2$), 42.2 (N(**C**$H_2$)($CH_2$)O), 45.5 (N(**C**$H_2$)($CH_2$)O), 59.3 (**C**H$CO_2$), 66.2 (N($CH_2$)(**C**$H_2$)O), 66.5 (N($CH_2$)(**C**$H_2$)O), 79.7 ($CH_2$**C**H($SO_2$Ph)$_2$), 127.5 (**C**H Ph ×2), 128.1 (**C**H Ph ×2), 128.5 (**C**H Ph ×2), 128.8 (**C**H Ph ×2), 129.0 (**C**H Ph), 129.1 (**C**H Ph ×2), 129.4 (**C**H Ph ×6), 130.8 (**C**H Ph), 134.5 (**C**H Ph), 134.6 (**C**H Ph), 136.5 (**C** Ph), 138.0 (**C** Ph), 138.5 (**C** Ph), 138.7 (**C** Ph), 169.6 (**C**=O), 172.0 (**C**=N)

**HRMS** (ESI$^+$) $C_{33}H_{32}N_2O_6S_2$ $[M+H]^+$ found 617.1752 requires 617.1775 (–3.7 ppm)

**Melting point:** 88 °C ($Et_2O$/$CH_2Cl_2$)

**(*S*)-2-((Diphenylmethylene)amino)-4,4-bis(phenylsulfonyl)-1-(pyrrolidin-1-yl)butan-1-one (20b)**

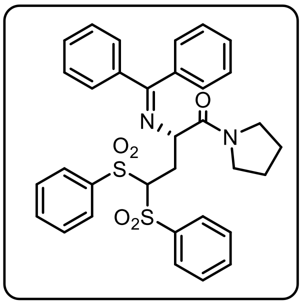


Following general procedure G, 4-nitrophenyl 2-((diphenylmethylene)amino)acetate **1** (72.1 mg, 0.2 mmol, 1.5 equiv.), (ethene-1,1-diyldisulfonyl)dibenzene **16** (40.1 mg, 0.13 mmol), (*R*)-BTM (6.6 mg, 0.026 mmol) and $CH_2Cl_2$ (260 µL) at rt for 24 h, then pyrrolidine (11 µL, 0.13 mmol, 1.0 equiv.) for 24 h at rt, gave after purification by column chromatography (30:70, $Et_2O$:$CH_2Cl_2$, $R_f$ = 0.5), the title compound (69 mg, 88%) as a pale yellow solid.

**HPLC analysis:** Chiralcel OD-H (85:15 hexane:IPA, flow rate 1 mL/min, 254 nm, 30 °C) $t_R$: 20.7 min, $t_R$: 35.1 min, 9:91 er

$[\alpha]_D^{20}$ +42.7 (*c* 0.5, $CHCl_3$)

**¹H-NMR** (400 MHz, $CD_2Cl_2$) $\delta_H$: 1.56-1.82 (4H, m, $N(CH_2)_2(C\mathbf{H}_2)_2$), 2.53-2.63 (1H, m, $N(C\mathbf{H}^a H)(CH_2)$), 2.63-2.73 (1H, m, $C\mathbf{H}^a HCH(SO_2Ph)_2$), 2.80-2.90 (1H, m, $CH\mathbf{H}^b CH(SO_2Ph)_2$), 3.17-3.34 (3H, m, $N(CH\mathbf{H}^b)(CH_2)$ and $N(C\mathbf{H}_2)_2(CH_2)_2$), 4.66-4.72 (1H, m, C**H**$CO_2$), 5.23-5.28 (1H, m, $CH_2C\mathbf{H}(SO_2Ph)_2$), 7.14-7.21 (2H, C**H** Ph ×2), 7.27-7.34 (C**H** Ph ×2), 7.39-7.52 (6H, m, C**H** Ph ×6), 7.52-7.62 (4H, m, C**H** Ph ×4), 7.65-7.75 (2H, m, C**H** Ph ×2), 7.89-7.99 (4H, m, C**H** Ph ×4)

**¹³C-NMR** (126 MHz, $CD_2Cl_2$) $\delta_C$: 23.9 ($N(CH_2)(\mathbf{C}H_2)$), 25.9 ($N(CH_2)(\mathbf{C}H_2)$), 30.2 ($\mathbf{C}H_2CH(SO_2Ph)_2$), 45.9 ($N(\mathbf{C}H_2)(CH_2)$), 46.0 ($N(\mathbf{C}H_2)(CH_2)$), 60.6 (**C**$HCO_2$), 79.8 ($CH_2\mathbf{C}H(SO_2Ph)_2$), 127.4 (**C**H Ph ×2), 128.0 (**C**H Ph ×2), 128.6 (**C**H Ph ×2), 128.7 (**C**H Ph), 128.8 (**C**(H Ph ×2), 129.1 (**C**H Ph ×2), 129.2 (**C**H Ph ×2), 129.4 (**C**H Ph ×2), 129.6 (**C**H Ph), 130.7 (**C**H Ph), 134.4 (**C**H Ph), 134.5 (**C**H Ph), 137.0 (**C** Ph), 137.9 (**C** Ph), 138.7 (**C** Ph ×2), 169.7 (**C**=O), 171.7 (**C**=N)

**HRMS** ($ESI^+$) $C_{33}H_{32}N_2O_5S_2$ $[M+H]^+$ found 601.1801 requires 601.1825 (–4.0 ppm)

**Melting point:** 64-68 °C ($Et_2O/CH_2Cl_2$)

***tert*-Butyl (*S*)-4-(2-((diphenylmethylene)amino)-4,4-bis(phenylsulfonyl)butanoyl)piperazine-1-carboxylate (20c)**

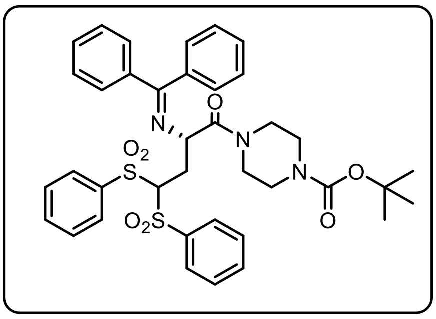


Following general procedure G, 4-nitrophenyl 2-((diphenylmethylene)amino)acetate **1** (72.1 mg, 0.2 mmol, 1.5 equiv.), (ethene-1,1-diyldisulfonyl)dibenzene **16** (40.1 mg, 0.13 mmol), (*R*)-BTM (6.6 mg, 0.026 mmol) and $CH_2Cl_2$ (260 µL) at rt for 24 h, followed by *N*-boc-piperazine (121.1 mg, 0.65 mmol) for 24 h at rt, gave after purification by column chromatography (50:50, EtOAc:Hexane, $R_f$ = 0.4) the title compound (76 mg, 81%) as a pale yellow solid.

**HPLC analysis:** Chiralcel OD-H (85:15 hexane:IPA, flow rate 1 mL/min, 254 nm, 40 °C) $t_R$: 23.0 min, $t_R$: 29.1 min, 9:91 er

$[\alpha]_D^{20}$ +32.7 (*c* 0.5, $CHCl_3$)

**¹H-NMR** (500 MHz, $CD_2Cl_2$) $\delta_H$: 1.46 (9H, s, $OC(C\mathbf{H}_3)_3$), 2.59-2.75 (2H, m, $C\mathbf{H}_2CH(SO_2Ph)_2$), 2.90-2.99 (1H, m, $N(C\mathbf{H}^a H)(CH_2)NBoc$), 2.99-3.07 (1H, m, $N(CH\mathbf{H}^b)(CH_2)NBoc$), 3.13-3.44 (6H, m,

N(C**H**$_2$)(CH$_2$)NBoc and N(CH$_2$)$_2$(C**H**$_2$)$_2$NBoc), 4.75 (1H, dd, *J* 9.7, 5.0, C**H**CO$_2$), 5.05 (1H, dd, *J* 7.4, 4.2, CH$_2$C**H**(SO$_2$Ph)$_2$), 7.09-7.14 (2H, m, C**H** Ph ×2), 7.32 (2H, app t, *J* 7.6, C**H** Ph ×2), 7.38-7.46 (3H, m, C**H** Ph ×3), 7.47-7.51 (3H, m, C**H** Ph ×3), 7.53-7.63 (4H, m, C**H** Ph ×4), 7.71 (1H, app t, *J* 7.1, C**H** Ph), 7.76 (1H, app t, *J* 7.6, C**H** Ph), 7.85 (2H, m, C**H** SO$_2$Ph ×2), 7.89 (2H, m, C**H** SO$_2$Ph ×2)

**$^{13}$C-NMR** (126 MHz, CD$_2$Cl$_2$) $\delta_C$: 28.0 (OC(**C**H$_3$)$_3$), 30.2 (**C**H$_2$CH(SO$_2$Ph)$_2$), 41.7 (N(CH$_2$)$_2$(**C**H$_2$)$_2$NBoc), 44.8 ((N(**C**H$_2$)$_2$(CH$_2$)$_2$NBoc), 59.8 (**C**HCO$_2$), 79.7 (CH$_2$**C**H(SO$_2$Ph)$_2$), 79.8 (**C**(CH$_3$)$_3$), 127.5 (**C**H Ph ×2), 128.1 (**C**H Ph ×2), 128.5 (**C**H Ph ×2), 128.8 (**C**H Ph ×2), 128.9 (**C**H Ph), 129.1 (**C**H Ph ×4), 129.3 (**C**H Ph ×2), 129.4 (**C**H Ph ×2), 130.8 (**C**(4')H Ph), 134.6 (**C**H Ph), 134.6 (**C**H), 136.4 (**C** Ph), 138.1 (**C** Ph), 138.5 (**C** Ph), 138.7 (**C** Ph), 154.2 (**C**=O carbamate), 169.6 (**C**=O amide), 171.9 (**C**=N)

**HRMS** (ESI$^+$) $C_{38}H_{41}N_3O_7S_2$ [M+H]$^+$ found 716.2439 requires 716.2459 (–2.8 ppm)

**Melting point:** 82-86 °C (EtOAC/Hexane)

**Methyl (*S*)-2-((diphenylmethylene)amino)-4,4-bis(phenylsulfonyl)butanoate (20d)**

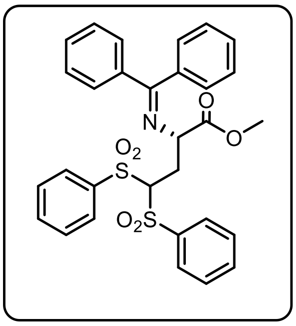


Following general procedure G, 4-nitrophenyl 2-((diphenylmethylene)amino)acetate **1** (72.1 mg, 0.2 mmol, 1.5 equiv.), (ethene-1,1-diyldisulfonyl)dibenzene **16** (40.1 mg, 0.13 mmol), (*R*)-BTM (6.6 mg, 0.026 mmol) and CH$_2$Cl$_2$ (260 µL) at rt for 24 h, then DMAP (3.2 mg, 0.026 mmol, 0.2 equiv.) and MeOH (0.5 mL, 0.3 M) for 24 h at rt, gave after purification by column chromatography (50:50, EtOAc:PE, $R_f$ = 0.5), the title compound (59 mg, 81%) as a white solid.

**HPLC analysis:** Chiralpak IB (95:5 hexane:IPA, flow rate 1 mL/min, 254 nm, 30 °C) $t_R$: 32.5 min, $t_R$: 37.4 min, 9:91 er

$[\alpha]_D^{20}$ –48.9 (*c* 0.75, CHCl$_3$)

**$^1$H-NMR** (500 MHz, CD$_2$Cl$_2$) $\delta_H$: 2.73-2.88 (2H, m, C**H**$_2$CH(SO$_2$Ph)$_2$, 3.66 (3H, s, OC**H**$_3$), 4.52-4.56 (1H, m, C**H**CO$_2$), 4.81-4.85 (1H, m, CH$_2$C**H**(SO$_2$Ph)$_2$), 7.19 (2H, m, C**H** Ph ×2), 7.38 (2H, app t, *J* 7.4, C**H** Ph ×2), 7.46-7.51 (3H, m, C**H** Ph ×3), 7.52-7.59 (5H, m, C**H** Ph ×5), 7.62 (2H, app t, *J* 8.3, C**H** Ph ×2), 7.72 (1H, app t, *J* 7.7 (C**H** Ph), 7.77 (1H, app t, *J* 7.8, C**H** Ph), 7.83-7.87 (2H, m, C**H** Ph ×2), 7.88-7.92 (2H, m, C**H** Ph ×2)

**$^{13}$C-NMR** (126 MHz, CD$_2$Cl$_2$) $\delta_C$: 29.8 (**C**H$_2$CH(SO$_2$Ph)$_2$), 52.4 (O**C**H$_3$), 61.8 (**C**HCO$_2$), 79.5 (CH$_2$**C**H(SO$_2$Ph)$_2$), 127.9 (**C**H Ph ×2), 128.1 (**C**H Ph ×2), 128.6 (**C**H Ph ×2), 128.9 (**C**H Ph ×2) 129.1

(**C**H Ph ×3), 129.3 (**C**H Ph ×2), 129.5 (**C**H Ph ×2), 129.6 (**C**H Ph ×2), 130.9 (**C**H Ph), 133.9 (**C**H Ph), 134.2 (**C**H Ph), 135.6 (**C** Ph), 137.8 (**C** Ph), 137.9 (**C** Ph), 138.9 (**C** Ph), 171.0 (**C**=N), 172.9 (**C**=O)

**HRMS** ($ESI^+$) $C_{30}H_{27}NO_6S_2$ $[M+H]^+$ found 562.1333 requires 562.1353 (–3.6 ppm)

**Melting point:** 48-50 °C (EtOAc/PE)

**(*S*)-2-Benzoyl-4-((diphenylmethylene)amino)-5-morpholino-1-phenylpentane-1,5-dione (20e)**

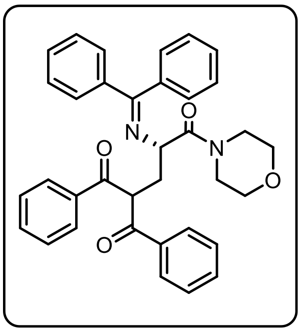


Following general procedure G, 4-nitrophenyl 2-((diphenylmethylene)amino)acetate **1** (72.1 mg, 0.2 mmol), 2-methylene-1,3-diphenylpropane-1,3-dione **S37** (47.3 mg, 0.2 mmol), (2*S*,3*R*)-HyperSe (14.2 mg, 0.04 mmol,) IPA (400 µL) and MeCN (150 µL) at rt for 30 min, followed by morpholine (87 µL, 1 mmol) for 10 min at rt, gave after purification by column chromatography (30:70, $Et_2O$:$CH_2Cl_2$, $R_f$ = 0.5), the title compound (88.6 mg, 81%) as a white solid.

**HPLC analysis:** Chiralcel OD-H (90:10 hexane:IPA, flow rate 1 mL/min, 254 nm, 30 °C) $t_R$: 16.4 min, $t_R$: 23.1 min, 89:11 er

$[\alpha]_D^{20}$ –81.1 (*c* 1.0, $CHCl_3$)

**$^1$H-NMR** (400 MHz, $CD_2Cl_2$) $\delta_H$: 2.63 (2H, t, *J* 6.2, C**H**$_2$CH(COPh)$_2$), 3.03-3.12 (1H, m, N(C**H**$^a$H)(CH$_2$)O), 3.30-3.64 (7H, m, N(CH**H**$^b$)(CH$_2$)O, N(C**H**$_2$)(CH$_2$)O and N(CH$_2$)$_2$(C**H**$_2$)$_2$O), 4.37 (1H, t, *J* 6.3, C**H**CO), 5.86 (1H, t, *J* 6.5, CH$_2$C**H**(COPh)$_2$), 6.99 (2H, m, C**H** Ph ×2), 7.37-7.51 (8H, m, C**H** Ph ×8), 7.53-7.63 (3H, m, C**H** Ph ×3), 7.65-7.74 (m, 3H, C**H** Ph ×3), 8.0 (2H, m, C**H** Ph ×2), 8.16 (2H, m, C**H** Ph ×2)

**$^{13}$C-NMR** (126 MHz, $CD_2Cl_2$) $\delta_C$: 33.2 (**C**H$_2$CH(COPh)$_2$), 42.0 (N(**C**H$_2$)(CH$_2$)O), 45.5 (N(**C**H$_2$)(CH$_2$)O), 52.6 (CH$_2$**C**H(COPh)$_2$), 60.1 (**C**HCO), 66.3 (N(CH$_2$)(**C**H$_2$)O), 66.6 (N(CH$_2$)(**C**H$_2$)O), 127.3 (**C**H Ph ×2), 128.1 (**C**H Ph ×2), 128.5 (**C**H Ph ×2), 128.6 (**C**H Ph ×2), 128.6 (**C**H Ph ×2), 128.7 (**C**H Ph), 128.8 (**C**H Ph ×2), 128.8 (**C**H Ph ×2), 129.0 (**C**H Ph ×2), 130.6 (**C**H Ph), 133.7 (**C**H Ph), 133.8 (**C**H Ph), 135.5 (**C** Ph), 136.0 (**C** Ph), 136.6 (**C** Ph), 138.9 (**C** Ph), 170.4 (**C**=O amide), 171.2 (**C**=N), 196.1 (**C**=O ketone), 196.3 (**C**=O, ketone)

**HRMS** ($ESI^+$) $C_{35}H_{32}N_2O_4$ $[M+H]^+$ found 545.2426 requires 545.2435 (–1.7 ppm)

**Melting point:** 60-62 °C ($Et_2O$/$CH_2Cl_2$)

**Di-*tert*-butyl (*S*)-2-(2-((diphenylmethylene)amino)-3-morpholino-3-oxopropyl)malonate (20f)**

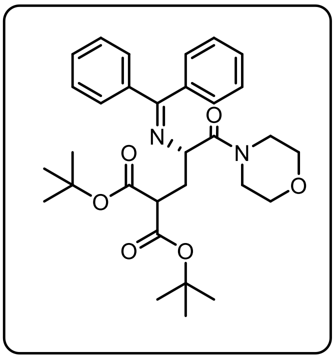

Following general procedure G, 4-nitrophenyl 2-((diphenylmethylene)amino)acetate **1** (72.1 mg, 0.2 mmol), di-*tert*-butyl 2-methylenemalonate **S39** (45.7 mg, 0.2 mmol), (2*S*,3*R*)-HyperSe (14.2 mg, 0.04 mmol), IPA (400 µL, 0.5 M) and MeCN (100 µL) for 24 h at rt, followed by morpholine (87.1 mg, 1.0 mmol) for 10 min at rt, gave after purification by column chromatography (50:50, EtOAc:Hexane, $R_f$ = 0.5), the title compound (64.2 mg, 60%) as a colourless liquid.

**HPLC analysis:** Chiralpak AD-H (98:2 hexane:IPA, flow rate 1 mL/min, 254 nm, 30 °C) $t_R$: 38.30 min, $t_R$: 44.6 min, 5:95 er

$[\alpha]_D^{20}$ –26.8 (*c* 1.0, $CHCl_3$)

**$^1$H-NMR** (500 MHz, $CD_2Cl_2$) $\delta_H$: 1.38 (9H, s, C(C**H**$_3$)$_3$), 1.42 (9H, s, C(C**H**$_3$)$_3$), 2.26-2.34 (1H, m, C**H**$^a$HCH(COO$^t$Bu)$_2$), 2.37-2.44 (1H, m, CH**H**$^b$CH(COO$^t$Bu)$_2$), 3.07-3.14 (1H, m, N(C**H**$^a$H)(CH$_2$)O), 3.30-3.41 (2H, m, (N(C**H$_2$**)(CH$_2$)O), 3.43 (1H, dd, *J* 9.7, 4.9, CH$_2$C**H**(COO$^t$Bu)$_2$), 3.46-3.55 (3H, m, N(CH**H**$^b$)(CH$_2$)O and N(CH)(C**H**$_2$)O), 3.56-3.66 (2H, m, N(CH)(C**H**$_2$)O), 4.22 (1H, dd, *J* 9.7, 4.3, C**H**CON), 7.10-7.15 (2H, m, C**H** Ph ×2), 7.38 (2H, app t, J 7.7, C**H** Ph ×2), 7.44-7.52 (4H, m, C**H** Ph ×4), 7.66-7.70 (C**H** Ph ×2)

**$^{13}$C-NMR** (126 MHz, $CD_2Cl_2$) $\delta_C$: 27.6 (C(**C**H$_3$)$_3$), 32.9 (**C**H$_2$CH(COO$^t$Bu)$_2$), 42.2 (N(**C**H$_2$)(CH$_2$)O), 45.6 (N(**C**H$_2$)(CH$_2$)O), 50.7 (CH$_2$**C**H(COO$^t$Bu)$_2$), 60.5 (**C**HCON), 66.4 (N(CH$_2$)$_2$(**C**H$_2$)O), 66.7 (N(CH$_2$)(**C**H$_2$)O), 81.5 (**C**(CH$_3$)$_3$), 127.5 (**C**H Ph ×2), 128.0 (**C**H Ph ×2), 128.6 (**C**H Ph ×2), 128.6 (**C**H Ph ×2), 128.7 (**C**H Ph), 130.5 (**C**H Ph), 136.8 (**C** Ph), 139.1 (**C** Ph), 168.3 (**C**=O ester), 168.5 (**C**=O ester), 170.4 (**C**=O amide), 170.8 (**C**=N)

**HRMS** (ESI$^+$) $C_{31}H_{40}N_2O_6$ $[M+H]^+$ found 537.2944 requires 537.2959 (–2.8 ppm)

**IR $\nu_{max}$** (film): 2976, 1721 (C=O ester), 1653 (C=N imine), 1447, 1368, 1275, 1246 (C-O ester), 1138, 1028, 847

**(*S*)-2-((Diphenylmethylene)amino)-4-(4-methoxybenzoyl)-5-(4-methoxyphenyl)-1-morpholinopentane-1,5-dione (20g)**

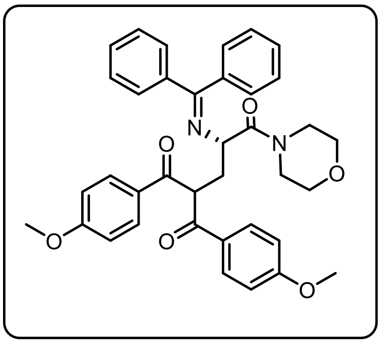

Following general procedure G, 4-nitrophenyl 2-((diphenylmethylene)amino)acetate **1** (72.1 mg, 0.2 mmol), 1,3-bis(4-methoxyphenyl)-2-methylenepropane-1,3-dione **S40** (59.3 mg, 0.2 mmol), (2*S*,3*R*)-HyperSe (14.2 mg, 0.04 mmol), IPA (400 µL) and MeCN (150 µL) at rt for 2 h, followed by morpholine (87 µL, 1.0 mmol) for 10 min at rt, gave after purification by column chromatography (30:70, $Et_2O:CH_2Cl_2$, $R_f$ = 0.5), the title compound (89 mg, 74%) as a yellow solid.

**HPLC analysis:** Chiralpak AD-H (80:20 Hexane:IPA, flow rate 2 mL/min, 254 nm, 30 °C) $t_R$: 35.1 min, $t_R$: 49.8 min, 14:86 er

$[\alpha]_D^{20}$ –109.1 (*c* 1.0, $CHCl_3$)

**$^1$H-NMR** (500 MHz, $CD_2Cl_2$) $\delta_H$: 2.50-2.62 (2H, m, $(COAr)_2CHC\mathbf{H}_2$), 3.01-309 (1H, m, $N(C\mathbf{H}^aH)_2(CH_2)_2O$), 3.26-3.56 (7H, m, $N(CH\mathbf{H}^b)_2(CH_2)_2O$, $(C\mathbf{H}_2)(CH_2)O$, $(CH_2)_2(C\mathbf{H}_2)_2O$), 3.82 (3H, s, $OC\mathbf{H}_3$), 3.89 (3H, s, $OC\mathbf{H}_3$), 4.28-4.34 (1H, m, C**H**CON), 5.61-5.67 (1H, m, $(COAr)_2C\mathbf{H}CH_2$), 6.87 (2H, d, *J* 8.6, C**H** Ar ×2), 6.93 (2H, m, C**H** Ar ×2), 7.00 (2H, d, *J* 8.6, C**H** Ar ×2), 7.34-7.47 (6H, C**H** Ar ×6), 7.69 (2H, d, *J* 5.9, C**H** Ar ×2), 7.92 (2H, d, *J* 8.9, C**H** Ar ×2), 8.11 (2H, d, *J* 8.4, C**H** Ar ×2)

**$^{13}$C-NMR** (126 MHz, $CD_2Cl_2$) $\delta_C$: 33.4 ($(COAr)_2CH\mathbf{C}H_2$), 42.0 ($N(\mathbf{C}H_2)(CH_2)O$), 45.5 ($N(\mathbf{C}H_2)(CH_2)O$), 52.2 ($(COAr)_2\mathbf{C}HCH_2$), 55.5 ($O\mathbf{C}H_3$), 55.6 ($O\mathbf{C}H_3$), 60.3 (**C**HCON), 66.4 ($N(CH_2)(\mathbf{C}H_2)O$), 66.6 ($N(CH_2)(\mathbf{C}H_2)O$), 113.9 (**C**H Ar ×2), 114.1 (**C**H Ar ×2), 127.3 (**C**H Ar ×2), 128.1 (**C**(3’)H Ar ×2), 128.5 (**C** Ar), 128.5 (**C**H Ar ×2), 128.6 (**C**H Ar ×2), 128.7 (**C**H Ar), 129.1 (**C** Ar), 130.7 (**C**H Ar), 130.8 (**C**H Ar ×2), 131.2 (**C**H Ar ×2), 136.6 (**C** Ar), 139.0 (**C** Ar), 163.8 (**C** Ar), 164.0 (**C** Ar), 170.5 (**C**=N), 171.1 (**C**=O amide), 194.5 (**C**=O ketone), 194.9 (**C**=O ketone)

**HRMS** ($ESI^+$) $C_{37}H_{36}N_2O_6$ $[M+H]^+$ found 605.2634 requires 605.2646 (–2.0 ppm), [MNa] found 627.2447 requires 627.2471 (–3.8 ppm)

**Melting point:** 54-56 °C ($Et_2O/CH_2Cl_2$)

**(*S*)-2-(4-(Dimethylamino)benzoyl)-1-(4-(dimethylamino)phenyl)-4-((diphenylmethylene)amino)-5-morpholinopentane-1,5-dione (20h)**

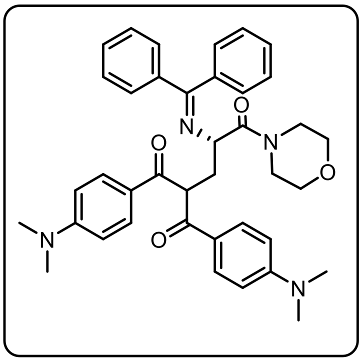

Following general procedure G, 4-nitrophenyl 2-((diphenylmethylene)amino)acetate **1** (72.1 mg, 0.2 mmol), 1,3-bis(4-(dimethylamino)phenyl)-2-methylenepropane-1,3-dione **S41** (64.4 mg, 0.2 mmol), (2*S*,3*R*)-HyperSe (14.2 mg, 0.04 mmol), IPA (400 µL) and MeCN (150 µL) at rt for 1 h, followed by morpholine (87 µL, 1.0 mmol) and $CH_2Cl_2$ (minimum volume, until unsoluble are dissolved ≈ 300 µL) for 30 min at rt, gave after purification by column chromatography (70:30, $CH_2Cl_2$:$Et_2O$, $R_f$ = 0.3) the title compound (38.6 mg, 31%) as a yellow solid.

**HPLC analysis:** Chiralpak AD-H (80:20 hexane:IPA, flow rate 1 mL/min, 254 nm, 40 °C) $t_R$: 38.9 min, $t_R$: 43.5 min, 9:91 er

$[\alpha]_D^{20}$ +242.5 (*c* 1.0, $CHCl_3$)

**$^1$H-NMR** (400 MHz, $CD_2Cl_2$) $\delta_H$: 2.49-2.57 (1H, m, C**H**$^a$HCH(COAr)$_2$), 2.56-2.65 (1H, m, CH**H**$^b$CH(COAr)$_2$), 3.03 (6H, s, N(C**H**$_3$)$_2$), 3.06-3.10 (1H, m, N(C**H**$^a$H)(CH$_2$)O), 3.12 (6H, m, N(C**H**$_3$)$_2$), 3.29-3.38 (1H, m, N(CH$_2$)(C**H**$^a$H)O), 3.39-3.47 (2H, m, N(C**H**$_2$)(CH$_2$)O), 3.47-3.61 (5H, m, N(CH**H**$^b$)(CH$_2$)O, N(CH$_2$)(C**H**$_2$)O and N(CH$_2$)(CH**H**$^b$)O), 4.32 (1H, dd, *J* 9.4, 3.7, C**H**CON), 5.50 (1H, dd, *J* 9.1, 4.2, CH$_2$C**H**(COAr)$_2$), 6.60 (2H, d, *J* 9.0, C**H** Ar ×2), 6.75 (2H, d, *J* 8.9, C**H** Ar ×2), 6.90-6.95 (2H, m, C**H** Ar ×2), 7.37-7.50 (6H, m, C(2)**H** Ar ×6), 7.75 (2H, m, C**H** Ar ×2), 7.83 (2H, d, *J* 8.9, C**H** Ar ×2), 8.06 (2H, d, *J* 8.9, C**H** Ar ×2)

**$^{13}$C-NMR** (126 MHz, $CD_2Cl_2$) $\delta_C$: 33.8 (**C**H$_2$CH(COAr)$_2$), 39.7 (N(**C**H$_3$)$_2$), 39.8 (N(**C**H$_3$)$_2$), 42.0 (N(**C**H$_2$)(CH$_2$)O), 45.5 (N(**C**H$_2$)(CH$_2$)O), 51.7 (CH$_2$**C**H(COAr)$_2$), 60.7 (**C**HCON), 66.5 (N(CH$_2$)(**C**H$_2$)O), 66.7 (N(CH$_2$)(**C**H$_2$)O), 110.7 (**C**H Ar ×2), 110.9 (**C**H Ar ×2), 123.3 (**C** Ar), 124.2 (**C** Ar), 127.4 (**C**H Ar ×2), 128.1 (**C**H Ph ×2), 128.5 (**C**H Ar ×2), 128.5 (**C**H Ar), 128.5 (**C**H Ar ×2), 130.4 (**C**H Ar), 130.5 (**C**H Ar ×2), 131.0 (**C**H Ar ×2), 136.8 (**C** Ar), 139.2 (**C** Ar), 153.6 (**C** Ar), 153.8 (**C** Ar), 170.8 (**C**=O amide), 170.8 (**C**=N), 193.9 (**C**=O ketone), 194.5 (**C**=O ketone)

**HRMS** (ESI$^+$) $C_{39}H_{42}N_4O_4$ [M+H]$^+$ found 631.3270 requires 631.3279 (–1.4 ppm)

**Melting point:** 104-106 °C ($CH_2Cl_2$/$Et_2O$)

**(*S*)-2-(3,4-Dimethoxybenzoyl)-1-(3,4-dimethoxyphenyl)-4-((diphenylmethylene)amino)-5-morpholinopentane-1,5-dione (20i)**

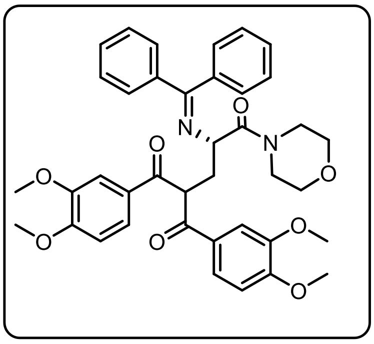

Following general procedure G, 4-nitrophenyl 2-((diphenylmethylene)amino)acetate **1** (72.1 mg, 0.2 mmol), 1,3-bis(3,4-dimethoxyphenyl)-2-methylenepropane-1,3-dione **S42** (71.3 mg, 0.2 mmol), (2*S*,3*R*)-HyperSe (14.2 mg, 0.04 mmol), IPA (400 µL) and MeCN (150 µL) at rt for 45 min, followed by morpholine (87 µL, 1.0 mmol) for 10 min at rt, gave after purification by column chromatography (70:30, $CH_2Cl_2$:$Et_2O$, $R_f$ = 0.3), the title compound (77.3 mg, 58%) as a white solid.

**HPLC analysis:** Chiralpak IA (80:20 hexane:IPA, flow rate 1 mL/min, 254 nm, 30 °C) $t_R$: 38.2 min, $t_R$: 47.2 min, 86:14 er

$[\alpha]_D^{20}$ +107.8 (*c* 1.0, $CHCl_3$)

**$^1$H-NMR** (400 MHz, $CD_2Cl_2$) $\delta_H$: 2.53-2.70 (2H, m, C**H**$_2$CH(COAr)$_2$), 3.04-3.14 (1H, m, N(C**H**$^a$H)(CH$_2$)O), 3.30-3.42 (2H, m, N(CH**H**$^b$)(CH$_2$)O and N(CH$_2$)(C**H**$^a$H)O), 3.43-3.64 (5H, m, N(C**H$_2$**)(CH$_2$)O, N(CH$_2$)(CH**H**$^b$)O and N(CH$_2$)(C**H**$_2$)O), 3.79 (3H, s, COC**H**$_3$), 3.89 (6H, d, *J* 2.7, COC**H**$_3$ and COC**H**$_3$), 3.97 (3H, s, COC**H**$_3$), 4.34-4.40 (1H, m, C**H**CON), 5.68-5.75 (1H, m, CH$_2$C**H**(COAr)$_2$), 6.83 (1H, d, *J* 8.9, C**H** Ar), 6.97-7.02 (3H, m, C**H** Ar ×3), 7.38 (2H, t, *J* 7.6, C**H** Ar ×2), 7.41-7.51 (5H, m, C**H** Ar ×5), 7.59 (1H, d, *J* 1.6, C**H** Ar), 7.64 (1H, dd, *J* 8.7, 1.6, C**H** Ar), 7.66-7.70 (2H, m, C**H** Ar ×2), 7.90 (1H, dd, *J* 8.6, 2.0, C**H** Ar)

**$^{13}$C-NMR** (126 MHz, $CD_2Cl_2$) $\delta_C$: 33.7 (**C**H$_2$CH(COAr)$_2$), 42.1 (N(**C**H$_2$)(CH$_2$)O), 45.6 (N(**C**H$_2$)(CH$_2$)O), 52.1 (CH$_2$**C**H(COAr)$_2$), 55.7 (O**C**H$_3$), 55.9 (O**C**H$_3$), 55.9 (O**C**H$_3$), 56.0 (O**C**H$_3$), 60.4 (**C**HCON), 66.4 (N(CH$_2$)(**C**H$_2$)O), 66.7 (N(CH$_2$)(**C**H$_2$)O), 110.4 (**C**H Ar), 110.5 (**C**H Ar), 110.8 (**C**H Ar), 111.1 (**C**H Ar), 123.2 (**C**H Ar), 123.5 (**C**H Ar), 127.4 (**C**H Ar ×2), 128.0 (**C**H Ar ×2), 128.5 (**C**H Ar ×2), 128.6 (**C**H Ar ×2), 128.6 (**C** Ar), 128.7 (**C**H Ar), 129.3 (**C** Ar), 130.5 (**C**H Ar), 136.6 (**C** Ar), 139.1 (**C** Ar), 149.4 (**C**OCH$_3$ Ar), 149.5 (**C**OCH$_3$ Ar), 153.8 (**C**OCH$_3$ Ar), 154.1 (**C**OCH$_3$ Ar), 170.5 (**C**=O amide), 170.9 (**C**=N), 194.4 (**C**=O ketone), 194.9 (**C**=O ketone)

**HRMS** (ESI$^+$) $C_{39}H_{40}N_2O_8$ $[M+H]^+$ found 665.2837 requires 665.2857 (–3.0 ppm)

**Melting point:** 64-68 °C ($CH_2Cl_2$/$Et_2O$)

**(*S*)-2-(4-Bromobenzoyl)-1-(4-bromophenyl)-4-((diphenylmethylene)amino)-5-morpholinopentane-1,5-dione (20j)**

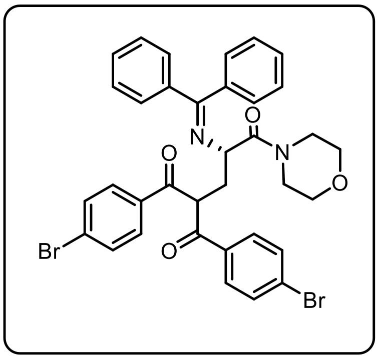

Following general procedure G, 4-nitrophenyl 2-((diphenylmethylene)amino)acetate **1** (72.1 mg, 0.2 mmol), 1,3-bis(4-bromophenyl)-2-methylenepropane-1,3-dione **S43** (157.6 mg, 0.4 mmol, 2 equiv.), (2*S*,3*R*)-HyperSe (14.2 mg, 0.04 mmol), IPA (400 µL) and MeCN (150 µL) at rt for 10 min, followed by morpholine (87 µL, 1.0 mmol) for 10 min at rt, gave after purification by column chromatography (70:30, $CH_2Cl_2$:$Et_2O$, $R_f$ = 0.7), the title compound (108.4 mg, 77%) as a white solid.

**HPLC analysis:** Chiralpak IB (80:20 hexane : IPA, flow rate 1 mL/min, 254 nm, 30 °C) $t_R$: 8.0 min, $t_R$: 8.4 min, 13:87 er

$[\alpha]_D^{20}$ +65.9 (*c* 1.0, $CHCl_3$)

**$^1$H-NMR** (400 MHz, $CD_2Cl_2$) $\delta_H$: 2.62 (2H, t, *J* 6.2, C**H**$_2$CH(COAr)$_2$), 2.99-3.08 (1H, m, N(C**H**$^a$H)(CH$_2$)O), 3.28-3.38 (2H, m, N(CH**H**$^b$)(CH$_2$)O and N(CH$_2$)(C**H**$^a$H)O), 3.39-3.49 (2H, m, N(C**H**$_2$)(CH$_2$)O), 3.50-3.63 (3H, m, N(CH$_2$)(C**H**$_2$)O and N(CH$_2$)(CH**H**$^b$)O), 4.39 (1H, t, *J* 5.6, C**H**CON), 5.86 (1H, t, *J* 6.0, CH$_2$C**H**(COAr)$_2$), 7.04-7.07 (2H, m, C**H** Ar ×2), 7.38 (2H, app t, *J* 8.3, C**H** Ar ×2), 7.43-7.51 (4H, m, C**H** Ar ×4), 7.59 (2H, d, *J* 8.9, C**H** Ar ×2), 7.61-7.66 (2H, m, C**H** Ar ×2), 7.70 (2H, d, *J* 8.5, C**H** Ar ×2), 7.89 (2H, d, *J* 8.9, C**H** Ar ×2), 8.00 (2H, d, *J* 8.7, C**H** Ar ×2)

**$^{13}$C-NMR** (126 MHz, $CD_2Cl_2$) $\delta_C$: 33.1 (**C**H$_2$CH(COAr)$_2$), 42.1 (N(**C**H$_2$)(CH$_2$)O), 45.5 (N(**C**H$_2$)(CH$_2$)O), 52.6 (CH$_2$**C**H(COAr)$_2$), 59.8 (**C**HCON), 66.3 (N(CH$_2$)(**C**H)O), 66.6 (N(CH$_2$)(**C**H)O), 127.3 (**C**H Ar ×2), 128.1 (**C**H Ph Ar ×2), 128.5 (**C**H Ph Ar ×2), 128.6 (**C** Ar), 128.7 (**C**H Ar ×2), 128.8 (**C** Ar), 128.8 (**C**H Ar), 130.2 (**C**H Ar ×2), 130.3 (**C**H Ar ×2), 130.7 (**C**H Ar), 132.1 (**C**H Ar ×2), 132.2 (**C**H Ar ×2),134.5 (**C** Ar), 134.8 (**C** Ar), 136.5 (**C** Ar), 138.9 (**C** Ar), 170.2 (**C**=O amide), 171.2 (**C**=N), 195.0 (**C**=O ketone), 195.2 (**C**=O ketone)

**HRMS** (ESI$^+$) $C_{35}H_{30}Br_2N_2O_4$ $[M+H]^+$ found 701.0642 requires 701.0645 (–0.4 ppm)

**Melting point:** 65-68 °C ($CH_2Cl_2$/$Et_2O$)

**(*S*)-2-(4-Chlorobenzoyl)-1-(4-chlorophenyl)-4-((diphenylmethylene)amino)-5-morpholinopentane-1,5-dione (20k)**

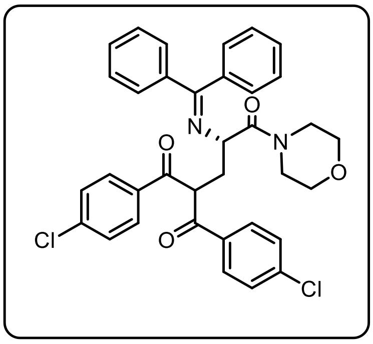


Following general procedure G, 4-nitrophenyl 2-((diphenylmethylene)amino)acetate **1** (72.1 mg, 0.2 mmol), 1,3-bis(4-chlorophenyl)-2-methylenepropane-1,3-dione **S44** (122 mg, 0.4 mmol, 2 equiv.), (2*S*,3*R*)-HyperSe (14.2 mg, 0.04 mmol), IPA (400 µL) and MeCN (150 µL) at rt for 10 min, followed by morpholine (87 µL, 1.0 mmol) for 10 min at rt, gave after purification by column chromatography (50:50, EtOAc:Hexane, $R_f$ = 0.4), the title compound (73.6 mg, 60%) as a white.

**HPLC analysis:** Chiralpak IB (85:15 hexane:IPA, flow rate 1 mL/min, 254 nm, 30 °C) $t_R$: 8.8 min, $t_R$: 9.3 min, 14:86 er

$[\alpha]_D^{20}$ +68.7 (*c* 1.0, $CHCl_3$)

**$^1$H-NMR** (400 MHz, $CD_2Cl_2$) $\delta_H$: 2.62 (2H, t, *J* 6.0, C**H**$_2$CH(COAr)$_2$), 2.99-3.09 (1H, m, N(C**H**$^a$H)(CH$_2$)O), 3.28-3.38 (2H, N(CH**H**$^b$)(CH$_2$)O and N(CH$_2$)(C**H**$^a$H)O), 3.42-3.49 (2H, m, N(C**H**$_2$)(CH$_2$)O), 3.50-3.64 (3H, m, N(CH$_2$)(C**H**$_2$)O and N(CH$_2$)(CH**H**$^b$)O), 4.38 (1H, t, *J* 5.8, C**H**CON), 5.86 (1H, t, *J* 5.4, CH$_2$C**H**(COAr)$_2$), 7.02-7.08 (2H, m, C**H** Ar ×2), 7.35-7.51 (8H, C**H** Ar ×8), 7.62-7.67 (2H, m, C**H** Ar ×2), 7.97 (2H, d, *J* 8.6, C**H** Ar ×2), 8.09 (2H, d, *J* 8.5, C**H** Ar ×2)

**$^{13}$C-NMR** (126 MHz, $CD_2Cl_2$) $\delta_C$: 33.1 (**C**H$_2$CH(COAr)$_2$), 42.0 (N(**C**H$_2$)(CH$_2$)O), 45.5 (N(**C**H$_2$)(CH$_2$)O), 52.6 (CH$_2$**C**H(COAr)$_2$), 59.8 (**C**HCON), 66.3 (N(CH$_2$)(**C**H$_2$)O), 66.6 (N(CH$_2$)(**C**H$_2$)O), 127.3 (**C**(3)H Ar ×2), 128.1 (**C**H Ar ×2), 128.5 (**C**H Ar ×2), 128.7 (**C**H Ar ×2), 128.9 (**C**H Ar), 129.1 (**C**H Ar ×2), 129.2 (**C**H Ar ×2), 130.1 (**C**H Ar ×2), 130.3 (**C**H Ar ×2), 130.7 (**C**H Ar), 134.0 (**C** Ar), 134.3 (**C** Ar), 136.5 (**C** Ar), 138.8 (**C** Ar), 139.9 (**C** Ar), 140.0 (**C** Ar), 170.2 (**C**=O amide), 171.3 (**C**=N), 194.8 (**C**=O ketone), 195.0 (**C**=O ketone)

**HRMS** (ESI$^+$) $C_{35}H_{30}Cl_2N_2O_4$ [M+H]$^+$ found 613.1655 requires 613.1655 (±0 ppm)

**Melting point:** 64-68 °C (EtOAc/hexane)

**(*S*)-2-(2-Naphthoyl)-4-((diphenylmethylene)amino)-5-morpholino-1-(naphthalen-2-yl)pentane-1,5-dione (20l)**

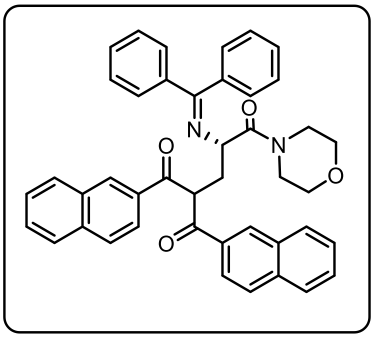

Following general procedure G, 4-nitrophenyl 2-((diphenylmethylene)amino)acetate **1** (72.1 mg, 0.2 mmol), 2-methylene-1,3-di(naphthalen-2-yl)propane-1,3-dione **S45** (67.3 mg, 0.2 mmol), (2*S*,3*R*)-HyperSe (14.2 mg, 0.04 mmol), IPA (400 µL) and MeCN (150 µL) at rt for 30 min, followed by morpholine (87 µL, 1.0 mmol) for 10 min at rt, gave after purification by column chromatography (30:70, $Et_2O$:$CH_2Cl_2$, $R_f$ = 0.6), the title compound (95.2 mg, 74%) as a white solid.

**HPLC analysis:** Chiralpak IA (80:20 Hexane:IPA, flow rate 1 mL/min, 254 nm, 30 °C) $t_R$: 23.7 min, $t_R$: 29.4 min, 89:11 er

$[\alpha]_D^{20}$ –157.5 (*c* 1.0, $CHCl_3$)

**$^1$H-NMR** (500 MHz, $CD_2Cl_2$) $\delta_H$: 2.77 (2H, t, *J* 6.2, C**H**$_2$CH(COAr)$_2$), 3.04-3.15 (1H, m, N(C**H**$^a$H)(CH$_2$)O), 3.29-3.65 (7H, m, N(CH**H**$^b$)(CH$_2$)O, N(C**H**$_2$)(CH$_2$)O and N(CH$_2$)$_2$(C**H**$_2$)$_2$O), 4.45 (1H, t, *J* 8.1, C**H**CON), 6.19 (1H, t, *J* 6.9, CH$_2$C**H**(COAr)$_2$), 6.99 (2H, m, C**H** Ar ×2), 7.37-7.44 (4H, m, C**H** Ar ×4), 7.44-7.55 (3H, m, C**H** Ar ×3), 7.63 (2H, m, C**H** Ar ×2), 7.70 (1H, t, *J* 8.5, C**H** Ar), 7.77 (2H, d, *J* 8.4, C**H** Ar ×2), 7.81 (1H, d, *J* 9.7, C**H** Ar), 7.90 (2H, d, *J* 9.1, C**H** Ar ×2), 7.94-8.02 (2H, m, C**H** Ar ×2), 8.05 (2H, t, *J* 7.5, C**H** Ar ×2), 8.15 (1H, d, *J* 8.5, C**H** Ar), 8.61 (1H, s, C**H** Ar), 8.83 (1H, s, C**H** Ar)

**$^{13}$C-NMR** (126 MHz, $CD_2Cl_2$) $\delta_C$: 33.6 (**C**H$_2$CH(COAr)$_2$), 42.1 (N(**C**H$_2$)$_2$(CH$_2$)$_2$O), 45.6 (N(**C**H$_2$)$_2$(CH$_2$)$_2$O), 52.9 (CH$_2$**C**H(COAr)$_2$), 60.2 (**C**HCON), 66.3 (N(CH$_2$)$_2$(**C**H$_2$)$_2$O), 66.6 (N(CH$_2$)$_2$(**C**H$_2$)$_2$O), 124.1 (**C**H Ar), 124.2 (**C**H Ar), 126.8 (**C**H Ar), 127.0 (**C**H Ar), 127.3 (**C**H Ar ×2), 127.7 (**C**H Ar), 127.8 (**C**H Ar), 128.1 (**C**H Ar ×2), 128.6-18.9 (m, **C**H Ar ×5), 129.7 (**C**H Ar), 129.8 (**C**H Ar), 130.5 (**C**H Ar), 130.6 (**C**H Ar), 130.8 (**C**H Ar), 132.5 (**C** Ar), 132.6 (**C** Ar), 133.1 (**C** Ar), 133.5 (**C** Ar), 135.7 (**C** Ar), 135.8 (**C** Ar), 136.6 (**C** Ar), 139.0 (**C** Ar), 170.4 (**C**=N), 171.2 (**C**=O amide), 196.1 (**C**=O ketone), 196.3 (**C**=O ketone)

**HRMS** (ESI$^+$) $C_{43}H_{36}N_2O_4$ $[M+H]^+$ found 645.2743 requires 645.2748 (–0.8 ppm)

**Melting point:** 70-73 °C ($Et_2O$/$CH_2Cl_2$)

**(*S*)-2-(1-Naphthoyl)-4-((diphenylmethylene)amino)-5-morpholino-1-(naphthalen-1-yl)pentane-1,5-dione (20m)**

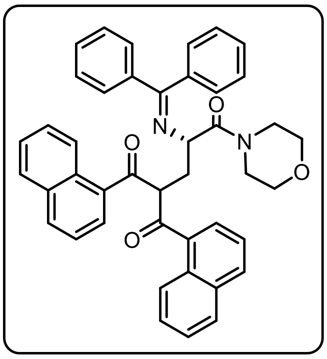

Following general procedure G, 4-nitrophenyl 2-((diphenylmethylene)amino)acetate **1** (72.1 mg, 0.2 mmol), 2-methylene-1,3-di(naphthalen-1-yl)propane-1,3-dione **S46** (134.6 mg, 0.4 mmol, 2.0 equiv.), (2*S*,3*R*)-HyperSe (14.2 mg, 0.04 mmol), IPA (400 µL) and MeCN (150 µL) at rt for 10 min, followed by morpholine (87 µL, 1.0 mmol) for 10 min at rt, gave after purification by column chromatography (70:30, $CH_2Cl_2$:$Et_2O$, $R_f$ = 0.6), the title compound (88 mg, 68%) as a light pink solid.

**HPLC analysis:** Chiralcel OD-H (90:10 hexane : IPA, flow rate 1 mL/min, 254 nm, 30 °C) $t_R$: 24.7 min, $t_R$: 30.3 min, 24:76 er

$[\alpha]_D^{20}$ –24.5 (*c* 1.0, $CHCl_3$)

**$^1$H-NMR** (400 MHz, $CD_2Cl_2$) $\delta_H$: 2.71-2.83 (2H, m, C**H**$_2$CH(COAr)$_2$), 3.02-3.09 (1H, m, N(C**H**$^a$H)(CH$_2$)O), 3.23-3.29 (1H, m, N(CH$_2$)(C**H**$^a$H)O), 3.30-3.37 (1H, m, N(CH**H**$^b$)(CH$_2$)O), 3.42-3.61 (5H, m, N(C**H**$_2$)(CH$_2$)O, N(CH$_2$)(CH**H**$^b$)O and N(CH$_2$)(C**H**$_2$)O), 4.43 (1H, dd, *J* 7.3, 5.3, C**H**CON), 6.16 (1H, dd, *J* 7.3, 4.9, CH$_2$C**H**(COAr)$_2$), 7.01-7.05 (2H, C**H** Ar ×2), 7.31-7.54 (12H, C**H** Ar ×12), 7.69-7.74 (C**H** Ar ×2), 7.84-7.89 (2H, m, C**H** Ar ×2), 7.94 (1H, d, *J* 9.3, C**H** Ar), 7.98 (1H, d, *J* 9.3, C**H** Ar), 8.07 (1H, d, *J* 7.0, C**H** Ar), 8.17 (1H, d, *J* 7.0, C**H** Ar), 8.32-8.36 (1H, m, C**H** Ar), 8.40 (1H, d, *J* 9.1, C**H** Ar)

**$^{13}$C-NMR** (126 MHz, $CD_2Cl_2$) $\delta_C$: 33.1 (**C**H$_2$CH(COAr)$_2$), 42.0 (N(**C**H$_2$)(CH$_2$)O), 45.6 (N(**C**H$_2$)(CH$_2$)O), 59.3 (CH$_2$**C**H(COAr)$_2$), 60.1 (**C**HCON), 66.2 (N(CH$_2$)(**C**H$_2$)O), 66.6 (N(CH$_2$)(**C**H$_2$)O), 124.3 (**C**H Ar), 124.4 (**C**H Ar), 125.4 (**C**H Ar), 125.5 (**C**H Ar), 126.5 (**C**H Ar), 126.5 (**C**H Ar), 127.3 (**C**H Ar ×2), 127.9 (**C**H Ar), 127.9 (**C**H Ar), 128.1 (**C**H Ar ), 128.3 (**C**H Ar), 128.3 (**C**H Ar), 128.5 (**C**H Ar), 128.6 (**C**(2)H Ar ×2), 128.7 (**C**H Ar), 128.8 (**C**H Ar), 130.2 (**C** Ar), 130.2 (**C** Ar), 130.7 (**C**H Ar), 132.9 (**C**H Ar), 133.0 (**C**H Ar), 133.9 (**C** Ar),135.0 (**C** Ar), 135.6 (**C** Ar), 136.6 (**C** Ar), 138.9 (**C** Ar), 170.5 (**C**=N), 199.4 (**C**=O ketone), 199.5 (**C**=O ketone)

**HRMS** (ESI$^+$) $C_{43}H_{36}N_2O_4$ $[M+H]^+$ found 645.2735 requires 645.2748 (–2.0 ppm)

**Melting point:** 62-65 °C ($CH_2Cl_2$/$Et_2O$)

**(*S*)-2-((Diphenylmethylene)amino)-1-morpholino-5-(thiophen-2-yl)-4-(thiophene-2-carbonyl)pentane-1,5-dione (20n)**

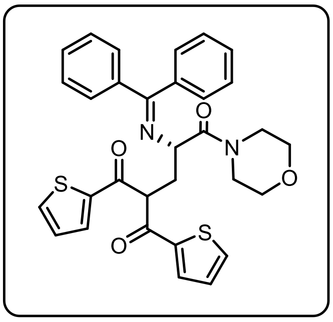

Following general procedure G, 4-nitrophenyl 2-((diphenylmethylene)amino)acetate **1** (72.1 mg, 0.2 mmol), 2-methylene-1,3-di(thiophen-2-yl)propane-1,3-dione **S47** (49.6 mg, 0.2 mmol), (2*S*,3*R*)-HyperSe (14.2 mg, 0.04 mmol) and EtOAc (400 µL) at rt for 24 h, followed by morpholine (87 µL, 1.0 mmol) for 10 min at rt, gave after purification by column chromatography (30:70, $Et_2O$:$CH_2Cl_2$, $R_f$ = 0.5) the title compound (85.9 mg, 77%) as a white solid.

**HPLC analysis:** Chiralpak IA (80:20 hexane:IPA, flow rate 1 mL/min, 254 nm, 30 °C) $t_R$: 16.2 min, $t_R$: 24.9 min, 58:42 er

$[\alpha]_D^{20}$ +12.5 (*c* 1.0, $CHCl_3$)

**$^1$H-NMR** (500 MHz, $CD_2Cl_2$) $\delta_H$: 2.56-2.64 (1H, m, C**H$^a$**HCH(COAr)$_2$), 2.67-2.74 (1H, m, CH**H$^b$**CH(COAr)$_2$), 2.99-3.08 (1H, m, N(C**H$^a$**H)(CH$_2$)O), 3.24-3.37 (2H, m, N(CH**H$^b$**)(CH$_2$)O and N(CH$_2$)(C**H$^a$**H)$_2$O), 3.45-3.52 (3H, m, N(C**H$_2$**)(CH$_2$)O and N(CH$_2$)(CH**H$^b$**)O), 3.53-3.63 (2H, m, N(CH$_2$)(C**H$_2$**)O), 4.32 (1H, dd, *J* 8.0, 4.8, C**H**CON), 5.55 (1H, dd, *J* 8.7, 4.6, CH$_2$C**H**(COAr)$_2$), 6.97-7.01 (2H, m, C**H** Ar ×2), 7.12 (1H, dd, *J* 4.5, 3.9, C**H** Ar), 7.25 (1H, dd, *J* 4.5, 4.1, C**H** Ar), 7.34-7.39 (2H, m, C**H** Ar ×2), 7.42-7.51 (4H, m, C**H** Ar ×4), 7.62-7.67 (2H, m, C**H** Ar ×2), 7.72 (1H, dd, *J* 4.5, 0.9, C**H** Ar), 7.79 (1H, dd, *J* 5.0, 1.1, C**H** Ar), 7.88 (1H, dd, *J* 3.9, 1.0, C**H** Ar), 8.09 (1H, dd, *J* 3.8, 0.8, C**H** Ar)

**$^{13}$C-NMR** (126 MHz, $CD_2Cl_2$) $\delta_C$: 33.9 (**C**H$_2$CH(COAr)$_2$), 42.1 (N(**C**H$_2$)(CH$_2$)O), 45.5 (N(**C**H$_2$)(CH$_2$)O), 55.2 (CH$_2$**C**H(COAr)$_2$), 60.0 (**C**HCON), 66.3 (N(CH$_2$)(**C**H$_2$)O), 66.6 (N(CH$_2$)(**C**H$_2$)O), 127.3 (**C**(3)H Ar ×2), 128.1 (**C**H Ar ×2), 128.5 (**C**H Ar), 128.5 (**C**H Ar), 128.5 (**C**H Ar ×2), 128.6 (**C**H Ar ×2), 128.8 (**C**H Ar), 130.6 (**C**H Ar), 133.2 (**C**H Ar), 133.6 (**C**H Ar), 134.8 (**C**H Ar), 135.2 (**C**H Ar), 136.4 (**C** Ar), 138.9 (**C** Ar), 142.9 (**C** Ar), 143.6 (**C** Ar), 170.0 (**C**=O amide), 171.1 (**C**=N), 187.8 (**C**=O ketone), 188.4 (**C**=O ketone)

**HRMS** (ESI$^+$) $C_{31}H_{28}N_2O_4S_2$ $[M+H]^+$ found 557.1558 requires 557.1563 (–0.9 ppm)

**Melting point:** 59-60 °C ($Et_2O$/$CH_2Cl_2$)

**Isopropyl (*S*)-2-((diphenylmethylene)amino)-5-oxo-5-(thiophen-2-yl)-4-(thiophene-2-carbonyl)pentanoate (S51)**

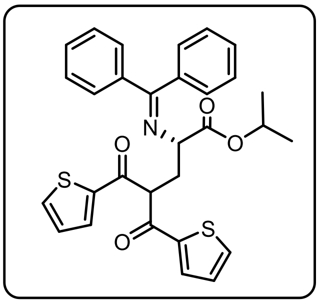


Following general procedure G, 4-nitrophenyl 2-((diphenylmethylene)amino)acetate **1** (72.1 mg, 0.2 mmol), 2-methylene-1,3-di(thiophen-2-yl)propane-1,3-dione **S47** (49.6 mg, 0.2 mmol), (2*S*,3*R*)-HyperSe (14.2 mg, 0.04 mmol), IPA (400 µL, 0.5 M) and MeCN (150 µL) at rt for 24 h, followed by morpholine (87 µL, 1.0 mmol) for 10 min at rt, gave after purification by column chromatography (30:70, $Et_2O$:$CH_2Cl_2$, $R_f$ = 0.8), the title compound (72.5 mg, 68%) as a white solid.

**HPLC analysis:** Chiralpak IB (95:5 Hexane:IPA, flow rate 1 mL/min, 254 nm, 30 °C) $t_R$: 13.2 min, $t_R$: 15.8 min, 34:66 er

$[\alpha]_D^{20}$ –1.6 (*c* 1.0, $CHCl_3$)

**$^1$H-NMR** (500 MHz, $CD_2Cl_2$) $\delta_H$: 1.24 (6H, t, *J* 6.7, $CH(CH_3)_2$), 2.57-2.68 (1H, m, C**H**$^a$HCH(COAr)$_2$), 2.81-2.90 (1H, m, CH**H**$^b$CH(COAr)$_2$), 4.10-4.19 (1H, m, C**H**$CO_2$), 5.02 (1H, m, C**H**$(CH_3)_2$), 5.32-5.36 (1H, m, $CH_2$C**H**(COAr)$_2$), 7.04 (2H, m, C**H** Ar ×2), 7.12 (1H, t, *J* 4.7, C**H** Ar), 4.72 (1H, t, *J* 4.3, C**H** Ar), 7.32-7.48 (6H, m, C**H** Ar ×6), 7.59 (2H, m, C**H** Ar ×2), 7.72 (1H, d, *J* 5.4, C**H** Ar), 7.78 (1H, d, *J* 4.5, C**H** Ar), 7.83 (1H, d, *J* 3.5, C**H** Ar), 8.02 (1H, d, *J* 3.3, C**H** Ar)

**$^{13}$C-NMR** (126 MHz, $CD_2Cl_2$) $\delta_C$: 21.5 (CH(**C**$H_3$)$_2$), 33.2 (**C**$H_2$CH(COAr)$_2$), 55.4 ($CH_2$**C**H(COAr)$_2$), 63.1 (C**H**$CO_2$), 68.8 (**C**H$(CH_3)_2$), 127.5 (**C**H Ar ×2), 128.1 (**C**H Ph Ar ×2), 128.4 (**C**H Ar ×3), 128.6 (**C**H Ar), 128.7 (**C**H Ar ×3), 130.5 (**C**H Ar), 132.9 (**C**H Ar), 133.5 (**C**H Ar), 134.7 (**C**H Ar), 135.1 (**C**H Ar), 135.8 (**C** Ar), 139.0 (**C** Ar), 142.9 (**C** Ar), 143.7 (**C** Ar), 170.9 (**C**=O ester), 188.2 (**C**=N), 186.9 (**C**=O ketone), 188.2 (**C**=O ketone)

**HRMS** (ESI$^+$) $C_{30}H_{27}NO_4S_2$ $[M+H]^+$ found 530.1447 requires 530.1454 (–1.3 ppm), $[M+Na]^+$ found 552.1262 requires 552.1279 (–3.1 ppm)

**Melting point:** 40 °C ($CH_2Cl_2$/$Et_2O$)

**(*S*)-2-((Diphenylmethylene)amino)-1-morpholino-5-(thiophen-3-yl)-4-(thiophene-3-carbonyl)pentane-1,5-dione (20o)**

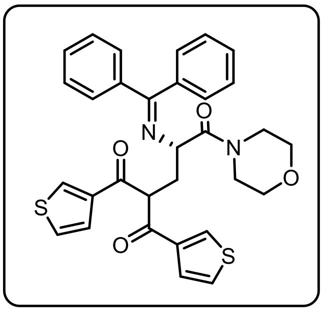

Following general procedure G, 4-nitrophenyl 2-((diphenylmethylene)amino)acetate **1** (72.1 mg, 0.2 mmol), 2-methylene-1,3-di(thiophen-3-yl)propane-1,3-dione **S48** (49.7 mg, 0.2 mmol), (2*S*,3*R*)-HyperSe (14.2 mg, 0.04 mmol), IPA (400 µL) and MeCN (150 µL) at rt for 30 min, followed by morpholine (87 µL, 1.0 mmol) and $CH_2Cl_2$ (minimum volume, until insoluble were dissolved ≈ 100 µL) for 30 min at rt, gave after purification by column chromatography (70:30, $CH_2Cl_2$:$Et_2O$, $R_f$ = 0.6), the title compound (67.3 mg, 60%) as a white solid.

**HPLC analysis:** Chiralpak IA (90:10 hexane:IPA, flow rate 1 mL/min, 254 nm, 30 °C) $t_R$: 30.8 min, $t_R$: 46.4 min, 85:15 er

$[\alpha]_D^{20}$ +66.4 (*c* 1.0, $CHCl_3$)

**$^1$H-NMR** (400 MHz, $CD_2Cl_2$) $\delta_H$: 2.53-2.61 (1H, m, C**H**$^a$HCH(COAr)$_2$), 2.63-2.73 (1H, m, CH**H**$^b$CH(COAr)$_2$), 2.98-3.08 (1H, m, N(C**H**$^a$H)(CH$_2$)O), 3.23-3.37 (2H, m, N(CH**H**$^b$)(CH$_2$)O and N(CH$_2$)(C**H**$^a$H)O), 3.41-3.63 (5H, m, N(C**H$_2$**)(CH$_2$)O, N(CH$_2$)(CH**H**$^b$)O) and N(CH$_2$)(C**H**$_2$)O), 4.34 (1H, dd, *J* 7.3, 5.4, C**H**CON), 5.49 (1H, dd, *J* 8.1, 4.6, CH$_2$C**H**(COAr)$_2$), 7.00-7.07 (2H, m, C**H** Ar ×2), 7.33-7.39 (3H, m, C**H** Ar ×3), 7.42 (1H, dd, *J* 4.9, 2.8, C**H** Ar), 7.44-7.49 (4H, m, C**H** Ar ×4), 7.53 (1H, m, C**H** Ar), 7.60-7.65 (3H, m, C**H** Ar ×3), 8.25 (1H, d, *J* 1.9, C**H** Ar), 8.42 (1H, d, *J* 1.9, C**H** Ar)

**$^{13}$C-NMR** (126 MHz, $CD_2Cl_2$) $\delta_C$: 33.3 (**C**H$_2$CH(COAr)$_2$), 42.1 (N(**C**H$_2$)(CH$_2$)O), 45.6 (N(**C**H$_2$)(CH$_2$)O), 56.3 (CH$_2$**C**H(COAr)$_2$), 60.1 (**C**HCON), 66.3 (N(CH$_2$)(**C**H$_2$)O), 66.6 (N(CH$_2$)(**C**H$_2$)O), 126.8 (**C**H Ar), 127.0 (**C**H Ar), 127.1 (**C**H Ar), 127.2 (**C**H Ar), 127.3 (**C**H Ar ×2), 128.1 (**C**H Ar ×2), 128.5 (**C**H Ar ×2), 128.6 (**C**H Ar ×2), 128.8 (**C**H Ar), 130.6 (**C**H Ar), 133.5 (**C**H Ar), 133.9 (**C**H Ar), 136.4 (**C** Ar), 138.9 (**C** Ar), 140.8 (**C** Ar), 141.5 (**C** Ar), 170.3 (**C**=O amide), 171.1 (**C**=N), 189.7 (**C**=O ketone), 190.1 (**C**=O ketone)

**HRMS** (ESI$^+$) $C_{31}H_{28}N_2O_4S_2$ [M+H]$^+$ found 557.1547 requires 557.1563 (– 2.9 ppm)

**Melting point:** 65-70 °C ($CH_2Cl_2$/$Et_2O$)

**(*S*)-2-((Diphenylmethylene)amino)-4-(furan-2-carbonyl)-5-(furan-2-yl)-1-morpholinopentane-1,5-dione (20p)**

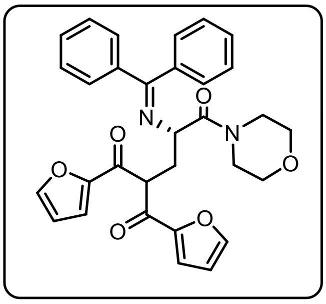

Following general procedure G, 4-nitrophenyl 2-((diphenylmethylene)amino)acetate **1** (72.1 mg, 0.2 mmol), 1,3-di(furan-2-yl)-2-methylenepropane-1,3-dione **S49** (43.2 mg, 0.2 mmol), (2*S*,3*R*)-HyperSe (14.2 mg, 0.04 mmol), IPA (400 µL) and MeCN (150 µL) at rt for 30 min, followed by morpholine (87 µL, 1.0 mmol) for 10 min at rt, gave after purification by column chromatography (90:10, $CH_2Cl_2$:$Et_2O$, $R_f$ = 0.4), the title compound (66.7 mg, 64%) as a white solid.

**HPLC analysis:** Chiralpak IB (80:20 hexane:IPA, flow rate 1 mL/min, 254 nm, 30 °C) $t_R$: 19.6 min, $t_R$: 26.9 min, 16:84 er

$[\alpha]_D^{20}$ +100 (*c* 1.0, $CHCl_3$)

**$^1$H-NMR** (400 MHz, $CD_2Cl_2$) $\delta_H$: 2.50-2.59 (1H, m, C**H**$^a$HCH(COAr)$_2$), 2.59-2.67 (1H, m, CH**H**$^b$CH(COAr)$_2$), 3.05-3.15 (1H, m, N(C**H**$^a$H)(CH$_2$)O), 3.31-3.40 (2H, m, N(CH**H**$^b$)(CH$_2$)O and N(CH$_2$)(C**H**$^a$H)O), 3.45-3.65 (5H, m, N(C**H**$_2$)(CH$_2$)O, N(CH$_2$)(CH**H**$^b$)O and N(CH$_2$)(C**H**$_2$)O), 4.29 (1H, dd, *J* 8.6, 4.6, C**H**CON), 5.35-5.39 (1H, m, CH$_2$C**H**(COAr)$_2$), 6.56 (1H, dd, *J* 3.4, 1.6, C**H** Ar), 6.64 (1H, dd, *J* 3.5, 1.5, C**H** Ar), 6.97-7.01 (2H, m, C**H** Ar ×2), 7.31 (1H, d, *J* 3.7, C**H** Ar), 7.37 (2H, t, *J* 7.8, C**H** Ar ×2), 7.42-7.50 (5H, m, C**H** Ar ×5), 7.58-7.60 (1H, m, C**H** Ar), 7.61-7.65 (2H, m C**H** Ar ×2), 7.69-7.71 (1H, m, C**H** Ar)

**$^{13}$C-NMR** (126 MHz, $CD_2Cl_2$) $\delta_C$: 32.9 (**C**H$_2$CH(COAr)$_2$), 42.2 (N(**C**H$_2$)(CH$_2$)O), 45.6 (N(**C**H$_2$)(CH$_2$)O), 53.4 (CH$_2$**C**H(COAr)$_2$), 60.2 (**C**HCON), 66.4 (N(CH$_2$)(**C**H$_2$)O), 66.7 (N(CH$_2$)(**C**H$_2$)O), 112.6 (**C**H Ar), 112.7 (**C**H Ar), 118.7 (**C**H Ar), 119.2 (**C**H Ar), 127.3 (**C**H Ar ×2), 128.0 (**C**H Ar ×2), 128.5 (**C**H Ar ×2), 128.6 (**C**H Ar ×2), 128.8 (**C**H Ar), 130.5 (**C**H Ar), 136.5 (**C** Ar), 138.9 (**C** Ar), 147.2 (**C**H Ar), 147.5 (**C**H Ar), 151.5 (**C** Ar), 152.0 (**C** Ar), 170.3 (**C**=O amide), 170.9 (**C**=N), 183.5 (**C**=O ketone), 183.9 (**C**=O ketone)

**HRMS** (ESI$^+$) $C_{31}H_{28}N_2O_6$ [M+H]$^+$ found 525.2002 requires 525.2020 (–3.4 ppm)

**Melting point:** 60-65 °C ($Et_2O$/$CH_2Cl_2$)

**(*S*)-1-(Benzo[d][1,3]dioxol-5-yl)-2-(benzo[d][1,3]dioxole-5-carbonyl)-4-((diphenylmethylene)amino)-5-morpholinopentane-1,5-dione (20q)**

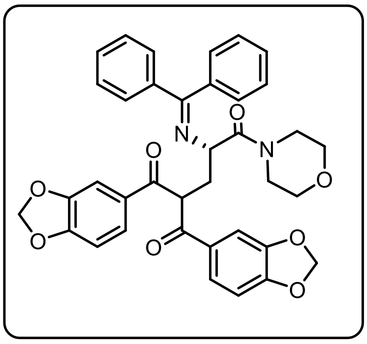

Following general procedure G, 4-nitrophenyl 2-((diphenylmethylene)amino)acetate **1** (72.1 mg, 0.2 mmol), 1,3-bis(benzo[d][1,3]dioxol-5-yl)-2-methylenepropane-1,3-dione **S50** (64.9 mg, 0.2 mmol), (2*S*,3*R*)-HyperSe (14.2 mg, 0.04 mmol), IPA (400 µL) and MeCN (150 µL) at rt for 45 min, followed by morpholine (87 µL, 1.0 mmol) for 10 min at rt, gave after purification by column chromatography (70:30, $CH_2Cl_2$:$Et_2O$, $R_f$ = 0.5), the title compound (93.7 mg, 74%) as a white solid.

**HPLC analysis:** Chiralpak IA (80:20 hexane:IPA, flow rate 1 mL/min, 254 nm, 30 °C) $t_R$: 32.6 min, $t_R$: 48.6 min, 90:10 er

$[\alpha]_D^{20}$ +106.3 (*c* 1.0, $CHCl_3$)

**$^1$H-NMR** (400 MHz, $CD_2Cl_2$) $\delta_H$: 2.53-2.65 (2H, m, C**H₂**CH(COAr)₂), 3.02-3.12 (1H, m, N(C**H**$^a$H)(CH₂)O), 3.30-3.64 (7H, m, N(CH**H**$^b$)(CH₂)O, N(C**H**₂)(CH₂)O and N(CH₂)₂(C**H**₂)₂O), 4.35 (1H, dd, *J* 7.7, 4.8, C**H**CON), 5.62 (1H, dd, *J* 7.6, 5.2, CH₂C**H**(COAr)₂), 6.04-6.08 (2H, m, OC**H**₂O Ar), 6.12 (2H, s, OC**H₂**O Ar'), 6.82 (1H, d, *J* 8.3, C**H** Ar), 6.96 (1H, d, *J* 8.3, C**H** Ar), 7.0-7.4 (2H, m, C**H** Ar ×2), 7.37-7.51 (7H, m, C**H** Ar ×7), 7.59 (1H, d, *J* 1.2, C**H** Ar), 7.61 (1H, dd, *J* 8.3, 1.3, C**H** Ar), 7.69-7.73 (2H, m, C**H** Ar ×2), 7.81 (1H, dd, *J* 8.2, 1.8, C**H** Ar)

**$^{13}$C-NMR** (126 MHz, $CD_2Cl_2$) $\delta_C$: 33.4 (**C**H₂CH(COAr)₂), 42.1 (N(**C**H₂)(CH₂)O), 45.5 (N(**C**H₂)(CH₂)O), 52.4 (CH₂**C**H(COAr)₂), 60.2 (**C**HCON), 66.3 (N(CH₂)(**C**H₂)O), 66.6 (N(CH₂)(**C**H₂)O), 102.2 (O**C**H₂O Ar), 102.3 (O**C**H₂O Ar), 108.0 (**C**H Ar), 108.1 (**C**H Ar), 108.1 (**C**H Ar), 108.4 (**C**H Ar), 125.0 (**C**H Ar), 125.3 (**C**H Ar), 127.3 (**C**(3)H Ar ×2), 128.1 (**C**H Ar ×2), 128.5 (**C**H Ar ×2), 128.6 (**C**H Ar ×2), 128.7 (**C**H Ar), 130.3 (**C** Ar), 130.6 (**C**H Ar), 130.9 (**C** Ar), 136.6 (**C** Ar), 139.0 (**C** Ar), 148.4 (**C** Ar), 148.6 (**C** Ar), 152.2 (**C** Ar), 152.4 (**C** Ar), 170.4 (**C**=O amide), 171.1 (**C**=N), 194.0 (**C**=O ketone), 194.3 (**C**=O ketone)

**HRMS** (ESI$^+$) $C_{37}H_{32}N_2O_8$ $[M+H]^+$ found 633.2231 requires 633.2231 (±0 ppm)

**Melting point:** 74-76 °C ($CH_2Cl_2$/$Et_2O$)

**(*S*)-2-((Diphenylmethylene)amino)-1-morpholino-5-phenylpentane-1,5-dione (20r)**

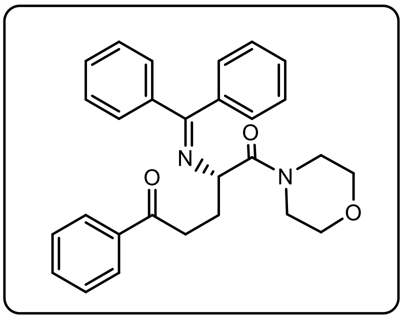

Following general procedure G, 4-nitrophenyl 2-((diphenylmethylene)amino)acetate **1** (72.1 mg, 0.2 mmol), 1-phenylprop-2-en-1-one **S51** (26.4 mg, 0.2 mmol), (2*S*,3*R*)-HyperSe (14.2 mg, 0.04 mmol) and EtOAc (400 μL, 0.5 M) at rt for 2 h, followed by morpholine (87 μL, 1.0 mmol) for 10 min at rt, gave after purification by column chromatography (30:70, $Et_2O$:$CH_2Cl_2$, R*f* = 0.3), the title compound as a yellow solid (27.7 mg, 31%).

**HPLC analysis:** Chiralpak AS-H (90:10 hexane:IPA, flow rate 1 mL/min, 254 nm, 30 °C) $t_R$: 9.4 min, $t_R$: 16.5 min, 34:66 er

$[\alpha]_D^{20}$ –14.0 (*c* 0.5, $CHCl_3$)

**$^1$H-NMR** (400 MHz, $CD_2Cl_2$) $\delta_H$: 2.22-2.40 (2H, m, $PhCOCH_2C\mathbf{H_2}$), 3.04-3.15 (1H, m, $PhCOC\mathbf{H^a}HCH_2$), 3.15-3.26 (1H, m, $PhCOCH\mathbf{H^b}CH_2$), 3.26-3.38 (1H, m, $N(C\mathbf{H^a}H)(CH_2)O$), 3.39-3.48 (1H, m, $N(CH_2)(C\mathbf{H^a}H)O$), 3.48-3.73 (6H, m, $N(CH\mathbf{H^b})(CH_2)O$, $N(CH_2)(CH\mathbf{H^b})O$, $N(C\mathbf{H_2})(CH_2)O$ and $N(CH_2)(C\mathbf{H_2})O$), 4.32-4.40 (1H, m, C**H**CON), 7.05-7.14 (2H, m, C**H** Ph x2), 7.34-7.41 (2H, m, C**H** Ph x2), 7.41-7.55 (6H, m, C**H** Ph x6), 7.56-7.63 (1H, m, C**H** Ph x1), 7.63-7.70 (2H, m, C**H** Ph x2), 7.03-8.01 (C**H** Ph x2)

**$^{13}$C-NMR** (126 MHz, $CD_2Cl_2$) $\delta_C$: 28.4 ($PhCOCH_2\mathbf{C}H_2$), 34.6 ($PhCO\mathbf{C}H_2CH_2$), 42.3 ($N(\mathbf{C}H_2)(CH_2)O$), 45.8 ($N(\mathbf{C}H_2)(CH_2)O$), 62.7 (**C**HCON), 66.7 ($N(CH_2)(\mathbf{C}H_2)O$), 66.8 ($N(CH_2)(\mathbf{C}H_2)O$), 127.5 (**C**H Ph x2), 127.9 (**C**H Ph x2), 128.0 (**C**H Ph x2), 128.5 (**C**H Ph x2), 128.5 (**C**H Ph x2), 128.6 (**C**H Ph x2), 128.7 (**C**H Ph x2), 130.4 (**C**H Ph), 133.0 (**C**H Ph), 136.7 (**C** Ph), 137.0 (**C** Ph), 139.2 (**C** Ph), 170.1 (**C**=N), 170.9 (**C**=O amide), 199.5 (**C**=O ketone)

**HRMS** ($ESI^+$) $C_{28}H_{28}N_2O_3$ $[M+H]^+$ found 441.2165 requires 441.2173 (–1.8 ppm)

**Melting point:** 127-130 °C ($Et_2O/CH_2Cl_2$)

**(*S*)-3-((diphenylmethylene)amino)-4-morpholino-4-oxobutane-1-sulfonyl fluoride (20s)**

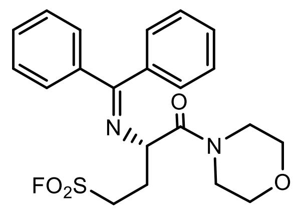


Following general procedure G, 4-nitrophenyl 2-((diphenylmethylene)amino)acetate **1** (72.1 mg, 0.2 mmol), ethenesulfonyl fluoride (22 mg, 0.2 mmol), (2*S*,3*R*)-HyperSe (14.2 mg, 0.04 mmol), IPA (400 µL) and MeCN (150 µL) for 10 min at rt, then morpholine (87 µL, 1.0 mmol) for 10 min at rt, gave after purification by column chromatography (30:70, $Et_2O$:$CH_2Cl_2$, $R_f$ = 0.5), the title compound as a white solid (41 mg, 47%).

**HPLC analysis:** Chiralpak IA (90:10 hexane:IPA, flow rate 1 mL/min, 254 nm, 30 °C) $t_R$: 14.2 min, $t_R$: 16.2 min, 73:27 er

$[\alpha]_D^{20}$ +5.0 (*c* 1.0, MeOH)

**$^1$H-NMR** (400 MHz, $CD_2Cl_2$) $\delta_H$: 2.38-2.51 (2H, m, C**H**$_2$CH$_2$SO$_2$F, 3.04-3.17 (1H, m, N(C**H**$^a$H)(CH$_2$)O), 3.24-3.34 (1H, m, N(CH**H**$^b$)(CH$_2$)O), 3.34-3.44 (1H, m, N(CH$_2$)(C**H**$^a$H)O), 3.44-3.68 (5H, m, N(C**H**$_2$)(CH$_2$)O), N(CH$_2$)(C**H**$_2$)O and N(CH$_2$)(CH**H**$^b$)O), 3.37-3.87 (2H, m, CH$_2$C**H**$_2$SO$_2$F), 4.46 (1H, app t, *J* 6.0, C**H**CO), 7.14-7.22 (2H, m, C**H** Ph x2), 7.40 (2H, app t, *J* 8.1, C**H** Ph x2), 7.48 (1H, app t, *J* 6.0, C**H** Ph), 7.52-7.60 (3H, m, C**H** Ph x3), 7.68 (2H, m, C**H** Ph x2)

**$^{13}$C-NMR** (126 MHz, $CD_2Cl_2$) $\delta_C$: 27.9 (**C**H$_2$CH$_2$SO$_2$F), 42.4 (N(**C**H$_2$)(CH$_2$)O), 45.7 (N(**C**H$_2$)(CH$_2$)O), 47.7 (d, $^2J_{C\text{-}F}$ 16.2, CH$_2$**C**H$_2$SO$_2$F), 59.7 (**C**HCO), 66.3 (N(CH$_2$)(**C**H$_2$)O), 66.7 (N(CH$_2$)(**C**H$_2$)O), 127.3 (**C**H Ph x2), 128.2 (**C**H Ph x2), 128.6 (**C**H Ph x2), 128.9 (**C**H Ph x2), 129.2 (**C**H Ph), 130.9 (**C**H Ph), 136.1 (**C** Ph), 138.7 (**C** Ph), 169.2 (**C**=O), 171.6 (**C**=N)

**HRMS** (ESI$^+$) $C_{21}H_{23}FN_2O_4S$ $[M+H]^+$ found 419.1426 requires 419.1435 (–2.1 ppm)

**Melting point:** 63-65 °C ($Et_2O$/$CH_2Cl_2$)

**Ethyl 2-oxo-6-phenyl-2H-pyran-4-carboxylate (20t)**

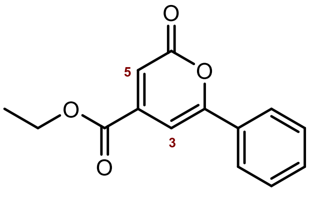


To a flame-dried Schlenk tube containing 4-nitrophenyl 2-((diphenylmethylene)amino)acetate **1** (103 mg, 0.285 mmol, 1.0 equiv.), ethyl (*E*)-4-oxo-4-phenylbut-2-enoate (58.2 mg, 0.285 mmol, 1.0 equiv.)

and *rac*-tetramisole.HCl (6.91 mg, 0.029 mmol, 0.1 equiv.) under argon, was added $CH_2Cl_2$ (2.9 mL, 0.1 M), followed by DIPEA (123 µL, 0.712 mmol, 2.5 equiv.). The reaction was stirred at rt for 24 h. Some 0.1 M solution of aq. HCl (5 mL) was then added. The aqueous layer was extracted with $CH_2Cl_2$ (10 mL ×3). The combined organic layers were dried on $MgSO_4$, filtered, and concentrated under reduced pressure. The crude residue was purified by column chromatography (30:70, $Et_2O$:PE, $R_f$ = 0.4) to give the title compound as a light-yellow solid (23.6 mg, 36%) with spectroscopic data in accordance with the literature [43].

**$^1$H-NMR** (400 MHz, $CD_2Cl_2$) $\delta_H$: 1.42 (3H, t, *J* 7.1, C**H$_3$**), 4.42 (2H, q, *J* 7.1, C**H**$_2$), 6.90 (1H, d, *J* 1.3, C=C(3)**H**), 7.12 (1H, d, *J* 1.3, C=C(5)**H**), 7.46–7.50 (3H, m, C**H** Ph x3), 7.86–7.90 (2H, m, C**H** Ph x2)

**Melting point:** 73-74 °C ($Et_2O$/PE)

## 5.4 Product derivatisations

**(*S*)-2-Amino-1-morpholino-4,4-bis(phenylsulfonyl)butan-1-one (21)**

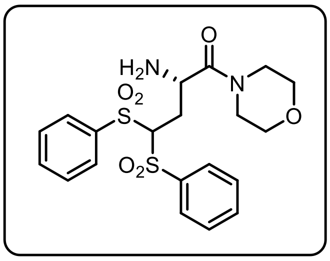

Following a modified procedure by Guerrero-Corella and co-workers [44], 2-((diphenylmethylene)amino)-1-morpholino-4,4-bis(phenylsulfonyl)butan-1-one **20a** (80.2 mg, 0.13 mmol, 1.0 equiv.) was dissolved in THF (260 µL, 0.5 M) and the reaction was cooled to –10 °C. Aq. HCl 10% in water (203 µL, 5.0 equiv.) was added slowly to the reaction mixture. The reaction was warmed to 0 °C and stirred for 3 h at 0 °C. $NaHCO_3$ sat. aq. solution was added (15 mL). The aq. layer was extracted with EtOAc (15 mL ×3). The combined organic layers were dried ($MgSO_4$), filtered, and concentrated *in vacuo*. The crude residue was purified by column chromatography (10:90, MeOH:$CH_2Cl_2$, $R_f$ = 0.5) to give the title compound (41.2 mg, 71%) as a white solid.

**HPLC analysis:** Chiralpak AD-H (80:20 hexane:IPA, flow rate 2 mL/min, 254 nm, 30 °C) $t_R$: 47.6 min, $t_R$: 60.1 min, 97:3 er

$[\alpha]_D^{20}$ +6.9 (*c* 1.0, $CHCl_3$)

**$^1$H-NMR** (500 MHz, $CDCl_3$) $\delta_H$: 2.05 (1H, dd, *J* 14.8, *J* 11.3, C**H$^a$**HCH($SO_2$Ph)$_2$), 2.40 (1H, ddd, *J* 15.3, *J* 9.6, *J* 2.9, CH**H$^b$**CH($SO_2$Ph)$_2$), 3.58-3.77 (8H, m, N(C**H**$_2$)$_2$($CH_2$)$_2$O and N($CH_2$)$_2$(C**H**$_2$)$_2$O), 4.14 (1H, dd, *J* 11.7, *J* 2.6, C**H**CO), 5.45 (1H, d, *J* 8.9, $CH_2$C**H**($SO_2$Ph)$_2$), 7.56 (2H, app t, *J* 8.1, C**H** Ph ×2), 7.61 (2H, app t, *J* 8.0, C**H** Ph ×2), 7.69 (1H, app t, *J* 7.4, C**H** Ph), 7.74 (1H, app t, *J* 7.3, C**H** Ph), 7.86 (2H, m, C**H** Ph ×2), 8.0 (2H, m, C**H** Ph ×2)

**$^{13}$C-NMR** (126 MHz, $CDCl_3$) $\delta_C$: 30.6 (**C**$H_2CH(SO_2Ph)_2$), 42.7 (N(**C**$H_2$)($CH_2$)O), 45.5 (N(**C**$H_2$)($CH_2$)O), 49.0 (**C**HCO), 66.8 (N($CH_2$)(**C**$H_2$)O), 79.2 ($CH_2$**C**H($SO_2Ph)_2$), 129.2 (**C**H Ph ×6), 129.6 (**C**H Ph ×2), 134.6 (**C**H Ph ×2), 137.1 (**C** Ph), 138.8 (**C** Ph), 173.0 (**C**=O)

**HRMS** (ESI$^+$) $C_{20}H_{24}N_2O_6S_2$ $[M+H]^+$ found 453.1144 requires 453.1149 (–1.1 ppm)

**Melting point:** 58-60 °C (MeOH/$CH_2Cl_2$)

**(*S*)-4-Bromo-*N*-(1-morpholino-1-oxo-4,4-bis(phenylsulfonyl)butan-2-yl)benzenesulfonamide (22)**

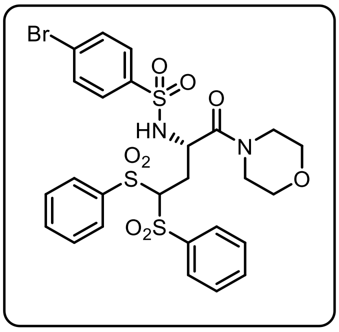

(*S*)-2-Amino-1-morpholino-4,4-bis(phenylsulfonyl)butan-1-one **21** (67.9 mg, 0.15 mmol, 1.0 equiv.), 4-bromobenzenesulfonyl chloride (57.5 mg, 0.225 mmol, 1.5 equiv.) and DIPEA (52 µL, 0.3 mmol, 2.0 equiv.) were dissolved in anhydrous $CH_2Cl_2$ (600 µL, 0.25 M). The reaction was stirred for 16 h at rt. The reaction was dissolved in EtOAc (15 mL). It was washed with aq. NaOH 1 M (15 mL ×1). The aq. layer was back extracted with EtOAc (15 mL ×2). The combined organic layers were washed with aq. NaOH 1 M (15 mL ×2), brine (15 mL ×1), dried ($MgSO_4$), filtered and concentrated *in vacuo*. The crude residue was purified by column chromatography (30:70, $Et_2O$:$CH_2Cl_2$, $R_f$ = 0.6) to yield the title compound (30 mg, 35%) as a white solid.

**HPLC analysis:** AS-H (80:20 hexane:IPA, flow rate 1 mL/min, 254 nm, 30 °C) $t_R$: 35.6 min, $t_R$: 48.6 min, 8:92 er

$[\alpha]_D^{20}$ +107.1 (*c* 1.0, $CHCl_3$)

**$^1$H-NMR** (500 MHz, $CDCl_3$) $\delta_H$: 2.15 (1H, dd, *J* 15.4, 12.1, C**H$^a$**HCH($SO_2Ph)_2$), 2.48 (1H, ddd, *J* 15.8, 9.4, 2.3, CH**H$^b$**CH($SO_2Ph)_2$), 3.24-3.43 (5H, m, N($CH_2$)(C**H$_2$**)O, N(C**H$_2$**)($CH_2$)O and N(C**H$^a$**H)($CH_2$)O), 3.47-3.54 (1H, m, N(CH**H$^b$**)($CH_2$)O), 3.58-3.68 (2H, m, N($CH_2$)(C**H$_2$**)O), 4.42-4.49 (**C**HCON), 5.19 (1H, d, *J* 9.0, $CH_2$C**H**($SO_2Ph)_2$), 5.65 (1H, d, *J* 9.0, N**H**$SO_2$Ar), 7.55-7.61 (2H, m, C**H** Ar ×2), 7.62-7.67 (2H, m, C**H** Ar ×2), 7.69 (4H, s, C**H** Ar ×4), 7.70-7.83 (2H, m, C**H** Ar ×2), 7.78-7.82 (2H, m, C**H** Ar ×2), 8.15-8.19 (2H, m, C**H** Ar ×2)

**$^{13}$C-NMR** (126 MHz, $CDCl_3$) $\delta_C$: 29.4 (**C**$H_2CH(SO_2Ph)_2$), 42.9 (N(C**H$_2$**)($CH_2$)O), 45.5 (N(C**H$_2$**)($CH_2$)O), 51.8 (**C**HCON), 66.3 (N($CH_2$)(C**H$_2$**)O), 66.4 (N($CH_2$)(C**H$_2$**)O), 77.3 ($CH_2$**C**H($SO_2Ph)_2$), 128.5 (**C** Ar), 129.2 (**C**H Ar ×2), 129.3 (**C**H Ar ×2), 129.3 (**C**H Ar ×2), 129.4 (**C**H Ar ×2), 129.5 (**C**H Ar ×2), 132.5 (**C**H Ar ×2), 134.8 (**C**H Ar), 134.9 (**C**H Ar), 136.5 (**C** Ar), 137.2 (**C** Ar), 138.6 (**C** Ar), 167.1 (**C**=O)

**HRMS** (ESI$^+$) $C_{26}H_{27}BrN_2O_8S_3$ $[M+Na]^+$ found 692.9994 requires 693.0011 (–2.5 ppm)

**Melting point:** 94-96 °C ($Et_2O/CH_2Cl_2$)

**Di-tert-butyl (*S*)-2-(2-amino-3-morpholino-3-oxopropyl)malonate 23**

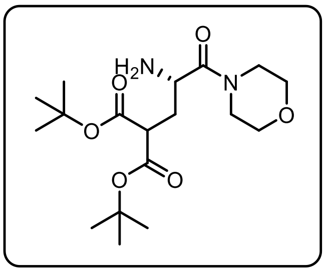

Following a procedure by Guerrero-Corella and co-workers [44], di-tert-butyl 2-(2-((diphenylmethylene)amino)-3-morpholino-3-oxopropyl)malonate **20f** (69.7 mg, 0.13 mmol, 1.0 equiv.) was dissolved in THF (260 µL, 0.5 M) and the reaction was cooled to –10 °C. Aq. HCl 10% in water (203 µL, 0.65 mmol, 5.0 equiv.) was added slowly to the reaction mixture. The reaction was warmed to 0 °C and stirred for 3 h at 0 °C. $NaHCO_3$ sat. aq. solution was added (15 mL). The aq. layer was extracted with EtOAc (15 mL ×3). The combined organic layers were dried ($MgSO_4$), filtered, and concentrated *in vacuo*. The crude residue was purified by column chromatography (5:95, MeOH:$CH_2Cl_2$, $R_f$ = 0.2) to give the title compound (34.2 mg, 71%) as a colourless liquid.

**HPLC analysis:** Chiralpak IA (95:5 hexane:IPA, flow rate 1 mL/min, 254 nm, 30 °C) $t_R$: 14.7 min, $t_R$: 17.3 min, 96:4 er

$[\alpha]_D^{20}$ –19.3 (*c* 1.0, $CHCl_3$)

**$^1$H-NMR** (500 MHz, $CDCl_3$) $\delta_H$: 1.47 (9H, s, C(C**H**$_3$)$_3$), 1.48 (9H, s, C(C**H**$_3$)$_3$), 1.65-1.73 (1H, m, C**H$^a$**HCH(COO$^t$Bu)$_2$), 2.04-2.12 (1H, m, CH**H$^b$**CH(COO$^t$Bu)$_2$), 3.52-3.62 (2H, m, N(C**H**$_2$)(CH$_2$)O), 3.63-3.79 (8H, m, N(C**H**$_2$)(CH$_2$)O, N(CH$_2$)$_2$(C**H**$_2$)$_2$O, CH$_2$C**H**(COO$^t$Bu)$_2$, C**H**CON)

**$^{13}$C-NMR** (126 MHz, $CDCl_3$) $\delta_C$: 27.9 (C(**C**H$_3$)$_3$), 28.0. (C(**C**H$_3$)$_3$), 33.9 (**C**H$_2$CH(COO$^t$Bu)$_2$), 42.5 (N(**C**H$_2$)(CH$_2$)O), 45.5 (N(**C**H$_2$)(CH$_2$)O), 49.1 (CH$_2$**C**H(COO$^t$Bu)$_2$), 50.2 (**C**HCON), 66.8 (N(CH$_2$)(**C**H$_2$)O), 66.9 (N(CH$_2$)(**C**H$_2$)O), 81.7 (**C**(CH$_3$)$_3$), 81.7 (**C**(CH$_3$)$_3$), 168.9 (**C**=O ester), 169.0 (**C**=O ester), 174.0 (**C**=O amide)

**HRMS** (ESI$^+$) $C_{18}H_{32}N_2O_6$ [M+H]$^+$ found 373.2325 requires 373.2333 (–2.1 ppm)

**IR $\nu_{max}$** (liquid) 2976, 1719 (C=O ester), 1643 (C=O amide), 1366, 1256, 1136, 1113, 843

**(*S*)-(5-(morpholine-4-carbonyl)-2-phenyl-4,5-dihydro-1H-pyrrole-1,3-diyl)bis(phenylmethanone) (24)**

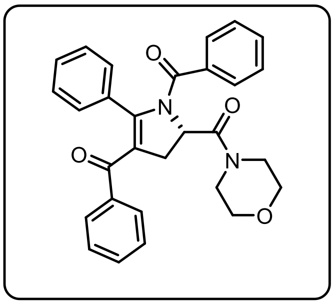

(*S*)-2-Benzoyl-4-((diphenylmethylene)amino)-5-morpholino-1-phenylpentane-1,5-dione **20e** (49.8 mg, 0.09 mmol, 1.0 equiv.) was dissolved in THF (180 μL, 0.5 M). Aq. HCl 1 M in water (900 μL, 0.9 mmol, 10 equiv.) was added. The reaction was stirred for 10 min at rt. Sat. aq. $NaHCO_3$ solution was added (15 mL). It was extracted with EtOAc (15 mL ×3). The combined organic layers were dried ($MgSO_4$), filtered and concentrated *in vacuo*. The crude residue was dissolved in MeCN (180 μL, 0.5 M). $NEt_3$ (25 μL, 0.18 mmol, 2.0 equiv.) and benzoyl chloride (63 μL, 0.54 mmol, 6.0 equiv.) were added. The reaction was stirred at 60 °C for 16 h. The reaction mixture was cooled to rt and concentrated *in vacuo.* The crude residue was purified by column chromatography (70:30, $Et_2O$:$CH_2Cl_2$, $R_f$ = 0.2) to yield the title compound (20.6 mg, 49%) as a white solid.

**HPLC analysis:** Chiralpak IA (80:20 hexane:IPA, flow rate 2 mL/min, 254 nm, 30 °C) $t_R$: 23.1 min, $t_R$: 47.7 min, 91:9 er

$[\alpha]_D^{20}$ +90.9 (*c* 1.0, $CHCl_3$)

**$^1$H-NMR** (500 MHz, $CDCl_3$) $\delta_H$: 2.92 (1H, dd, *J* 15.5, 2.6, C**H^a**HCH), 3.56-3.91 (9H, m, CH**H^b**CH, $N(CH_2)_2(C\mathbf{H}_2)_2O$, $N(C\mathbf{H}_2)_2(CH_2)_2O$), 5.46 (1H, dd, *J* 10.3, 1.4, $CH_2C\mathbf{H}$), 6.69 (2H, app t, *J* 7.7, C**H** Ph ×2), 6.77 (1H, app t, J 7.2, C**H** Ph), 6.91-7.04 (6H, m, C**H** Ph ×6), 7.07-7.14 (C**H** Ph ×2), 7.23-7.27 (2H, m, (C**H** Ph ×2), 7.30-7.34 (2H, m, C**H** Ph ×2)

**$^{13}$C-NMR** (126 MHz, $CDCl_3$) $\delta_C$: 35.4 ($\mathbf{C}H_2CH$), 42.6 ($N(\mathbf{C}H_2)(CH_2)O$), 45.9 ($N(\mathbf{C}H_2)(CH_2)O$), 60.2 ($CH_2\mathbf{C}H$), 66.4 ($N(CH_2)(\mathbf{C}H_2)O$), 66.9 ($N(CH_2)(\mathbf{C}H_2)O$), 121.4 (PhC=**C**(COPh)), 127.3 (**C**H Ph ×2), 127.5 (**C**H Ph ×4), 127.6 (**C**H Ph ×2), 128.5 (**C**H Ph ×2), 128.8 (**C**H Ph ×2), 128.9 (**C**H Ph), 130.5 (**C** Ph), 130.6 (**C**H Ph), 131.2 (**C**H Ph), 135.1 (**C** Ph), 137.9 (**C** Ph), 151.3 (Ph**C**=C(COPh), 168.8 (**C**=O amide morpholine), 169.5 (**C**=O phenyl amide), 195.1 (**C**=O ketone)

**HRMS** ($ESI^+$) $C_{29}H_{26}N_2O_4$ $[M+H]^+$ found 467.1962 requires 467.1965 (–0.6 ppm), $[M+Na]^+$ found 489.1781 requires 489.1790 (–1.8 ppm)

**Melting point:** 176–178 °C ($Et_2O$/$CH_2Cl_2$)

## 5.5 Optimisation tables for Michael addition reactions

### *5.5.1. Optimisation of reaction with electrophile 16*

**Optimisation to a single product**

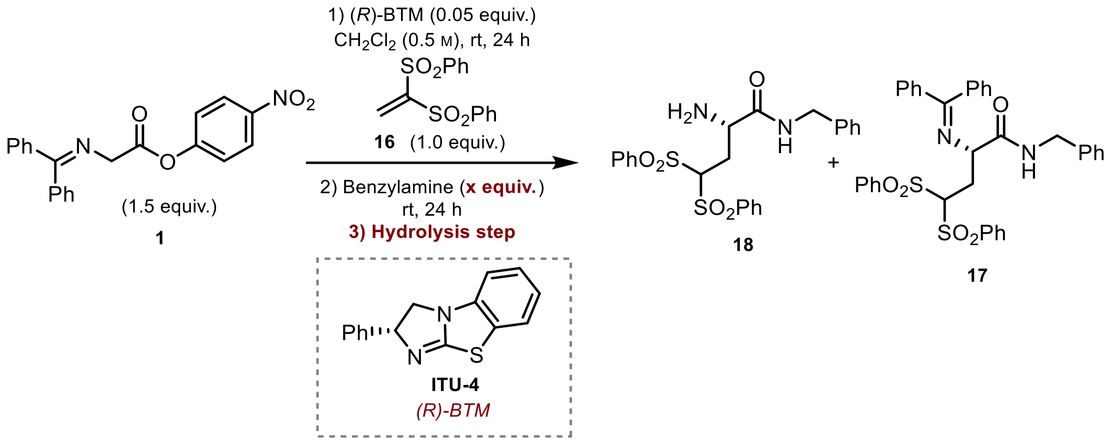


| *Entry* | *Anhydrous conditions* | *Benzylamine* | *Hydrolysis step* | *NMR yield 18*[a] | *Er 18* | *NMR yield 17*[a] | *Er 17* |
|---|---|---|---|---|---|---|---|
| 1 | None | 5.0 equiv. | None | 67% | 92:8 | 13% | - |
| 2 | Anhydrous $CH_2Cl_2$ + distilled benzylamine | 5.0 equiv. | None | 44% | 93:7 | 53% | - |
| 3 | Distilled $CH_2Cl_2$ + distilled benzylamine | 5.0 equiv. | None | 70% | 95:5 | 29% | - |
| 4 | None | 5.0 equiv. | Water, 4 h, rt | 47% | 89:11 | 32% | - |
| 5 | None | 5.0 equiv. | $NH_4Cl$ sat, 4h, rt | 58% | 88:12 | 40% | - |
| 6 | None | 5.0 equiv. | Citric Acid 0.5 M, 4 h, rt | 41% | 92:8 | 26% | - |
| 7 | None | 5.0 equiv. | HCl 2M in $Et_2O$, 4 h, rt | 48% | 94:6 | 18% | - |
| **8** | **None** | **1.0 equiv.** | **None** | **-** | **-** | **81%** | **93:7** |
| 9 | None | 1.5 equiv. | None | 24% | - | 79% | 92:8 |
| 10 | None | 2 equiv. | None | 33% | - | 63% | 93:7 |

[a]Measured using 1,3,5-trimethoxybenzene as internal standard.

***Table 2: Optimisation to a single product***

## Optimisation of the Lewis base catalyst

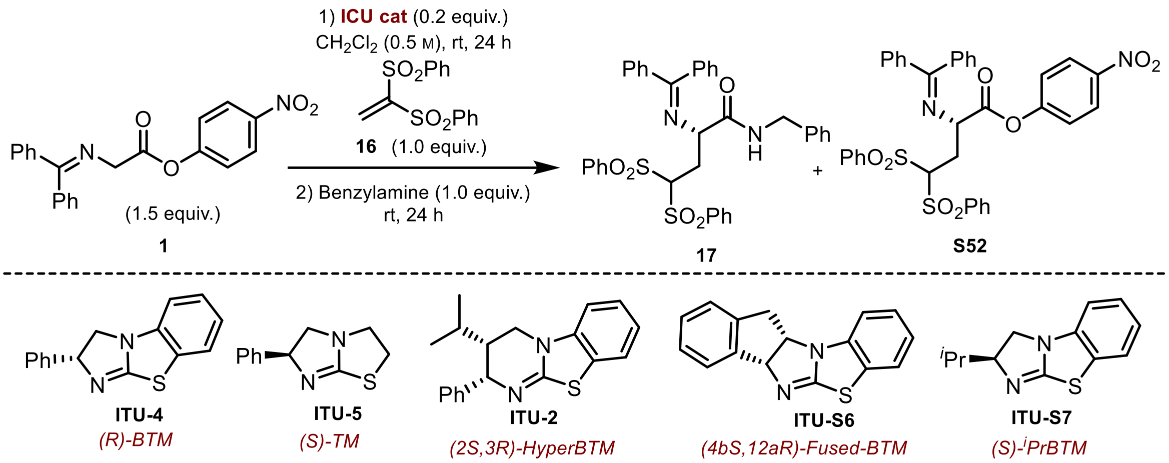


| *Entry* | *ICU cat* | *Isolated yield 17* | *NMR $^1$H yield S52*[a] | *Tot yield Michael addition* | *Er 17* |
|---|---|---|---|---|---|
| **1** | **(*R*)-BTM** | **70%** | **-** | **70%** | **93:7** |
| 2 | (*S*)-TM | 59% | - | 59% | 13:87 |
| 3 | (2*S*,3*R*)-HyperBTM | 57% | 20% | 77% | 90:10 |
| 4 | (4b*S*,12a*R*)-Fused-BTM | 50% | 23% | 73% | 63:37 |
| 5 | (*S*)-$^i$PrBTM | 42% | 14% | 58% | 28:72 |
| 6 | None | 36% | 38%[b] | 74% | 50:50 |

[a]NMR yield calculated with 1,3,5-trimethoxybenzene as internal standard; [b]Isolated yield after column chromatography.

***Table 3: Catalyst screen***

## Optimisation of the concentration and temperature

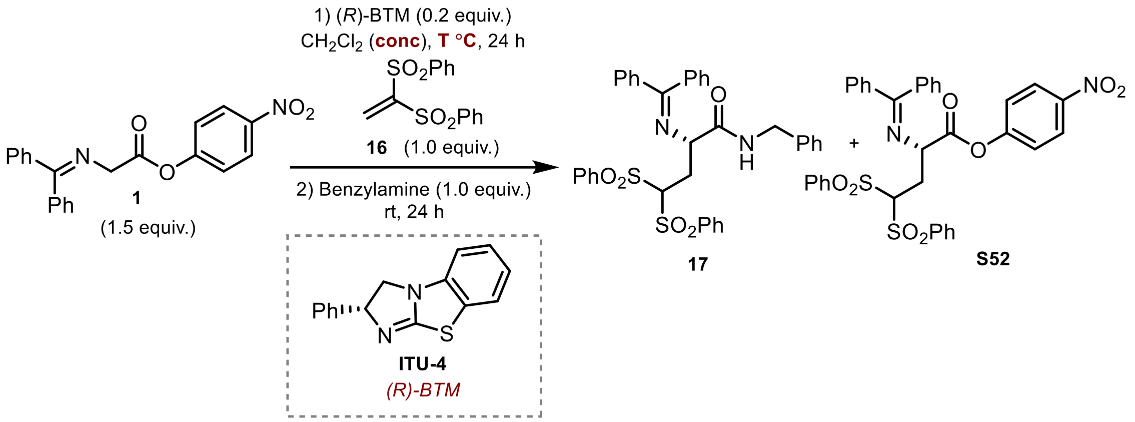


| Entry | Temperature | Concentration | Isolated yield 17 | NMR yield S52[a] | Tot yield Michael addition | Er 17 |
|---|---|---|---|---|---|---|
| **1** | **rt** | **0.5 M** | **70%** | **-** | **70%** | **93:7** |
| 2 | 0 °C | 0.5 M | 72% | - | 72% | 95:5 |
| 3 | –20 °C | 0.5 M | 55% | 20% | 75% | 95:5 |
| 4 | –78 °C | 0.5 M | 22%[a] | 29% | 51% | - |
| 5 | rt | 0.1 M | 52%[a] | 34% | 86% | 93:7 |
| 6 | rt | 0.05 M | 48% | 31% | 79% | 93:7 |

[a]NMR yield calculated with 1,3,5-trimethoxybenzene as internal standard.

***Table 4: Concentration and temperature screen***

## Slow addition investigations

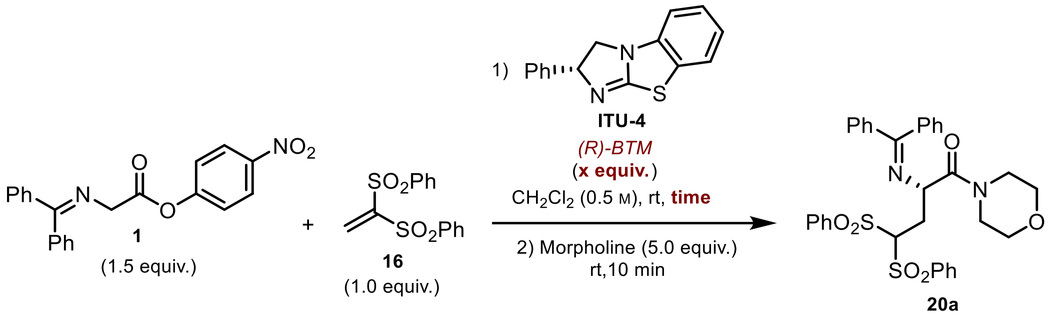


| Entry | Conditions | Catalyst loading | Isolated yield 20a | er 20a |
|---|---|---|---|---|
| 1 | 10 min | 0.2 equiv. | 96% | 93:7 |
| 2 | Slow addition **16**, 10 min | 0.2 equiv. | 72% | 98:2 |
| 3 | Slow addition **1**, 10 min | 0.2 equiv. | 92% | 93:7 |
| 4 | Slow addition **16**, 10 min + flush | 0.2 equiv. | 99% | 97:3 |
| 5 | Slow addition **16**, 30 min + flush | 0.1 equiv. | 94% | 98:2 |
| **6** | **Slow addition 16, 1 h + flush** | **0.05 equiv.** | **89%** | **98:2** |

***Table 5: Slow addition of one of the reagents to the reaction mixture***

## 5.5.2. *Optimisation of reaction with electrophile* ***S39***

### Optimisation of solvent, temperature and starting material equivalents

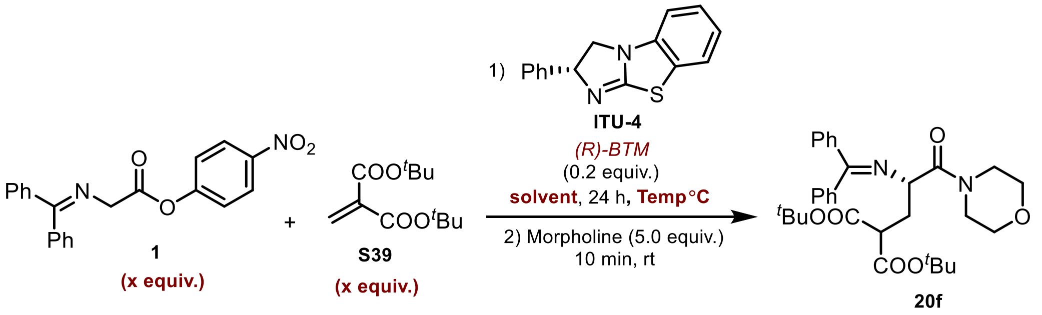


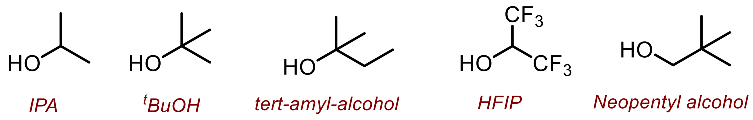


| *Entry* | *Equiv. 1* | *Equiv. S39* | *Solvent* | *Temperature* | *Isolated yield 20f* | *er 20f* |
|---|---|---|---|---|---|---|
| 1 | 1.5 | 1.0 | $CH_2Cl_2$ | rt | 66% | 64:36 |
| 2 | 1.5 | 1.0 | $CH_2Cl_2$ | 0 °C | 62%[a] | 68:32 |
| 3 | 1.5 | 1.0 | $CH_2Cl_2$ | –20 °C | 82% | 72:28 |
| 4 | 1.5 | 1.0 | $CH_2Cl_2$ | –40 °C | 70% | 76:24 |
| 5 | 1.5 | 1.0 | MeCN | rt | 47% | 68:32 |
| 6 | 1.5 | 1.0 | MTBE | rt | 53% | 62:38 |
| 7 | 1.5 | 1.0 | DMSO | rt | 59% | 65:35 |
| 8 | 1.5 | 1.0 | Ethylacetoacetate | rt | 43% | 67:33 |
| 9 | 1.5 | 1.0 | IPA | rt | 56% | 77:23 |
| 10 | 1.2 | 1.0 | IPA | rt | 59% | 77:23 |
| **11** | **1.0** | **1.0** | **IPA** | **rt** | **60%** | **78:22** |
| 12 | 1.0 | 1.5 | IPA | rt | 62% | 78:22 |
| 13 | 1.0 | 2.0 | IPA | rt | 71% | 76:24 |
| 14 | 1.0 | 1.0 | IPA | –20 °C | - | - |
| 15 | 1.0 | 1.0 | IPA/MeCN | –20 °C | 58% | 78:22 |
| 16 | 1.0 | 1.0 | IPA/DMF | –20 °C | 82% | 72:28 |
| 17 | 1.0 | 1.0 | $^{t}$BuOH | rt | 57% | 70:30 |
| 18 | 1.0 | 1.0 | Tert-amyl-alcohol | rt | 76% | 73:37 |
| 19 | 1.0 | 1.0 | HFIP | rt | - | - |
| 20 | 1.0 | 1.0 | DCM/neopentyl alcohol | rt | 60% | 73:27 |

[a]NMR yield calculated with 1,3,5-trimethoxybenzene as internal standard.

***Table 6: Optimisation of reactions conditions with electrophile S39***

**Optimisation of the Lewis base catalyst**

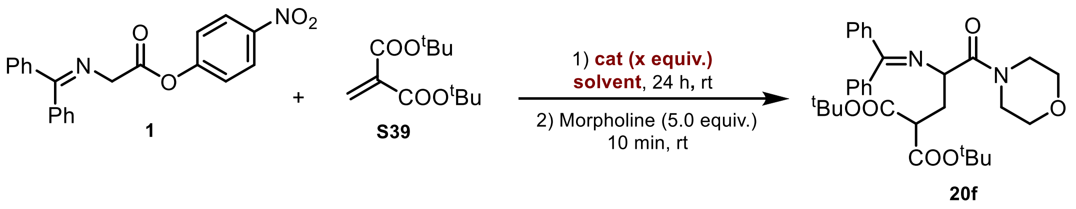


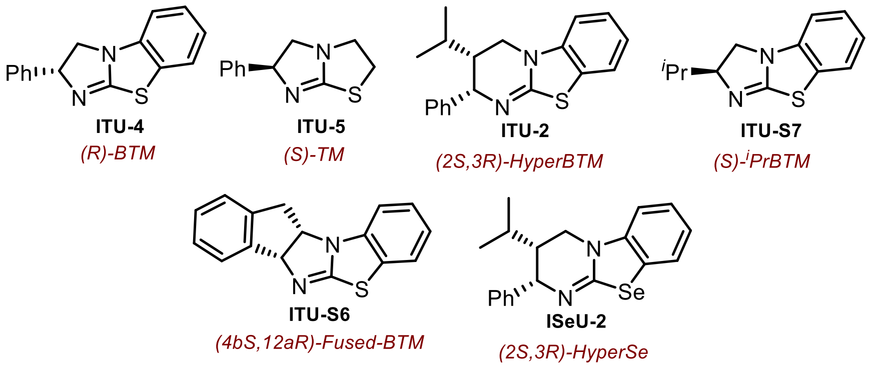


| *Entry* | *Catalyst* | *Amount catalyst* | *Solvent* | *Isolated yield 20f* | *Er 20f* |
|---|---|---|---|---|---|
| 1 | (*R*)-BTM | 0.2 equiv. | IPA | 60% | 78:22 |
| 2 | (*S*)-Tetramisole | 0.2 equiv. | IPA | 42% | 27:73 |
| 3 | (2*S*,3*R*)-HyperBTM | 0.2 equiv. | IPA | 44% | 72:28 |
| 4 | (*S*)-$^{i}$PrBTM | 0.2 equiv. | IPA | 61% | 58:42 |
| 5 | (4b*S*,12a*R*)-Fused-BTM | 0.2 equiv. | IPA | 63% | 40:60 |
| 6 | (2*S*,3*R*)-HyperSe | 0.2 equiv. | IPA | 48% | 95:5 |
| **7** | **(2*S*,3*R*)-HyperSe** | **0.2 equiv.** | **IPA/MeCN** | **60%** | **95:5** |
| 8 | (2*S*,3*R*)-HyperSe | 0.2 equiv. | MeCN | 82% | 87:13 |
| 9 | (2*S*,3*R*)-HyperSe | 0.2 equiv. | IPA/MeCN degassed[a] | 54%[b] | 94:6 |
| 10 | (2*S*,3*R*)-HyperSe | 0.1 equiv. | IPA/MeCN | 62% | 91:9 |
| 11 | (2*S*,3*R*)-HyperSe | 0.05 equiv. | IPA/MeCN | 58% | 90:10 |
| 12 | (2*S*,3*R*)-HyperSe | 0.025 equiv. | IPA/MeCN | 49% | 89:11 |

[a]Degassed using freeze, pump, thaw method; [b]NMR yield calculated with 1,3,5-trimethoxybenzene as internal standard.

***Table 7: Optimisation of the Lewis base catalyst and solvent system***

## 5.6 Identification of side-products observed during the optimisation

**4-Nitrophenyl (*S*)-2-((diphenylmethylene)amino)-4,4-bis(phenylsulfonyl)butanoate (S52)**

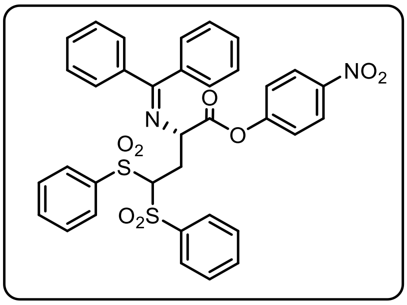

Following general procedure D, 4-nitrophenyl 2-((diphenylmethylene)amino)acetate **1** (72.1 mg, 0.2 mmol, 1.5 equiv.), (ethene-1,1-diyldisulfonyl)dibenzene **16** (40.1 mg, 0.13 mmol), (*R*)-BTM (6.6 mg, 0.026 mmol) and $CH_2Cl_2$ (260 µL, 0.5 M) at rt for 24 h, gave after purification by column chromatography (50:50, PE:EtOAc, $R_f$ = 0.5) the title compound (71 mg, 81%) as a pale yellow solid.

$[\alpha]_D^{20}$ –82.4 (*c* 0.75, $CHCl_3$)

**$^1$H-NMR** (400 MHz, $CD_2Cl_2$) $\delta_H$: 2.92-3.04 (2H, m, C**H**$_2$CH($SO_2$Ph)$_2$), 4.83 (1H, dd, *J* 7.1, 5.5, C**H**$CO_2$), 5.10 (1H, dd, *J* 6.4, 5.0, $CH_2$C**H**($SO_2$Ph)$_2$), 7.22-7.30 (4H, m, C**H** Ar ×4), 7.40 (2H, app t, *J* 7.9, C**H** Ar ×2), 7.49-7.60 (8H, m, C**H** Ar ×8), 7.63 (2H, app t, *J* 7.9, C**H** Ar ×2), 7.71 (1H, app t, *J* 7.5, C**H** Ar), 7.78 (1H, app t, *J* 7.5, C**H** Ar), 7.86 (2H, m, C**H** Ar ×2), 7.93 (2H, m, C**H** Ar ×2), 8.28 (2H, d, *J* 9.1, C**H** Ar ×2)

**$^{13}$C-NMR** (126 MHz, $CD_2Cl_2$) $\delta_C$: 29.5 (**C**$H_2$CH($SO_2$Ph)$_2$), 61.5 (**C**H$CO_2$), 79.0 ($CH_2$**C**H($SO_2$Ph)$_2$), 122.5 (**C**H Ar ×2), 125.1 (**C**H Ar ×2), 127.6 (**C**H Ar ×2), 128.2 (**C**H Ar ×2), 128.9 (**C**H Ar ×2), 129.0 (**C**H Ar ×2), 129.1 (**C**H Ar ×2), 129.3 (**C**H Ar ×3), 129.4 (**C**H Ar ×2), 129.6 (**C**H Ar ×2), 131.2 (**C**H Ar), 134.7 (**C**H Ar), 134.7 (**C**H Ar), 135.5 (**C** Ar), 137.6 (**C** Ar), 137.9 (**C** Ar), 138.6 (**C** Ar), 145.6 (**C** Ar), 155.1 (**C** Ar), 168.7 (**C**=O), 174.0 (**C**=N)

**HRMS** ($ESI^+$) $C_{35}H_{28}N_2O_8S_2$ $[M+H]^+$ found 669.1337 requires 669.1360 (–3.4 ppm)

**Melting point:** 58-62 °C (EtOAC/PE)

## 5.7 Absolute configuration

X-ray diffraction data for compound **22** were collected at 125 K using a Rigaku MM-007HF High Brilliance RA generator/confocal optics with XtaLAB P200 diffractometer [Cu Kα radiation (λ = 1.54187 Å)]. Intensity data were collected using ω steps accumulating area detector images spanning at least a hemisphere of reciprocal space. Data were collected using CrystalClear (*CrystalClear-SM Expert* v2.1. Rigaku Americas, *The Woodlands, Texas, USA*, and Rigaku Corporation, *Tokyo, Japan*, 2015) and processed (including correction for Lorentz, polarization and absorption) using CrysAlisPro (*CrysAlisPro* v1.171.41.93a. Rigaku Oxford Diffraction, Rigaku Corporation, *Oxford, U.K.*, 2020). The structure was solved by dual-space methods (SHELXT [45]) and refined by full-matrix least-squares against $F^2$ (SHELXL-2018/3 [42]). Non-hydrogen atoms were refined anisotropically, and hydrogen atoms were refined using a riding model, except for amine hydrogens which were located from the difference Fourier map and refined isotropically subject to a distance restraint. One $–SO_2Ph$ group in each of two independent molecules showed some disorder. This was modelled in each case across two sites. Some restraints to bond lengths and thermal motion were required. All calculations were performed using the Olex2 interface [46]. Selected crystallographic data are presented in the Table below. CCDC 2243587 contains the supplementary crystallographic data for this paper. These data can be obtained free of charge from The Cambridge Crystallographic Data Centre via www.ccdc.cam.ac.uk/structures.

**Data for 22**

| | ***22*** |
|---|---|
| Formula | $C_{26}H_{27}BrN_2O_8S_3$ |
| Fw | 671.58 |
| Crystal description | Colourless rod |
| Crystal size (mm³) | 0.51×0.06×0.05 |
| Space group | $P2_12_12_1$ |
| *a* [Å] | 7.91764(5) |
| *b* [Å] | 29.21393(15) |
| *c* [Å] | 37.57516(18) |
| vol [Å]³ | 8691.33(8) |
| Z | 12 |
| *ρ* (calc) [g/cm³] | 1.540 |
| *μ* [mm⁻¹] | 4.397 |
| F(000) | 4128 |
| reflections collected | 102722 |
| independent reflections ($R_{int}$) | 17760 (0.0329) |
| parameters, restraints | 1185, 98 |
| GoF on $F^2$ | 1.046 |
| $R_1$ [$I > 2\sigma(I)$] | 0.0260 |
| $wR_2$ (all data) | 0.0677 |
| absolute structure parameter | –0.009(3) |
| largest diff. peak/hole [e/Å³] | 0.808, –0.818 |

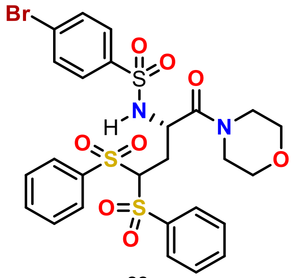


22

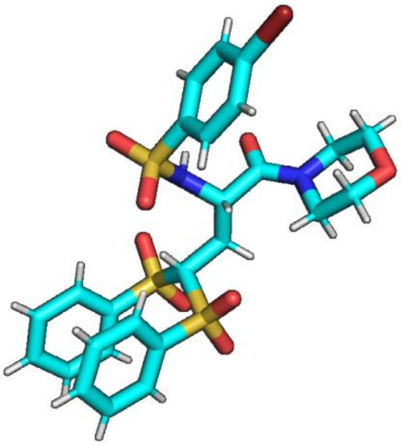

## References


[1] C. M. Young, A. Elmi, D. J. Pascoe, R. K. Morris, C. McLaughlin, A. M. Woods, A. B. Frost, A. de La Houpliere, K. B. Ling, T. K. Smith et al., *Angewandte Chemie (International ed. in English)* **2020**, *59* (*9)*, 3705–3710.

[2] L. C. Morrill, J. Douglas, T. Lebl, A. M. Z. Slawin, D. J. Fox, A. D. Smith, *Chemical Science* **2013**, *4* (*11)*, 4146.

[3] I. Shiina, K. Nakata, K. Ono, Y. Onda, M. Itagaki, *Journal of the American Chemical Society* **2010**, *132* (*33)*, 11629–11641.

[4] D. Daniels, S. Smith, T. Lebl, P. Shapland, A. Smith, *Synthesis* **2014**, *47* (*01)*, 34–41.

[5] *Organic Syntheses* **1964**, *44* (*51)*.

[6] Maximilian Tiffner, *Bifunctional Ammonium Salt Ion-Pairing Catalysts for (Stereoselective) Organic Reactions*, Johannes Kepler Universität Linz, **2018**.

[7] J. Voskuhl, M. Waller, S. Bandaru, B. A. Tkachenko, C. Fregonese, B. Wibbeling, P. R. Schreiner, B. J. Ravoo, *Organic & Biomolecular Chemistry* **2012**, *10* (*23)*, 4524–4530.

[8] T. Benkovics, I. A. Guzei, T. P. Yoon, *Angewandte Chemie (International ed. in English)* **2010**, *49* (*48)*, 9153–9157.

[9] H. Yamamoto, A. Saitoh, K. Ohkawa, *Macromolecular Bioscience* **2003**, *3* (*7)*, 354–363.

[10] M. Perez, P.-G. Echeverria, E. Martinez-Arripe, M. Ez Zoubir, R. Touati, Z. Zhang, J.-P. Genet, P. Phansavath, T. Ayad, V. Ratovelomanana-Vidal, *European Journal of Organic Chemistry* **2015**, *2015* (*27)*, 5949–5958.

[11] Y. Miyazawa, Z. Wang, M. Matsumoto, S. Hatano, I. Antol, E. Kayahara, S. Yamago, M. Abe, *Journal of the American Chemical Society* **2021**, *143* (*19)*, 7426–7439.

[12] Y.-M. Li, S.-J. Lou, Q.-H. Zhou, L.-W. Zhu, L.-F. Zhu, L. Li, *European Journal of Organic Chemistry* **2015**, *2015* (*14)*, 3044–3047.

[13] K. Zielke, O. Kováč, M. Winter, J. Pospíšil, M. Waser, *Chemistry - a European Journal* **2019**, *25* (*34)*, 8163–8168.

[14] T. Neveselý, C. G. Daniliuc, R. Gilmour, *Organic Letters* **2019**, *21* (*23)*, 9724–9728.

[15] L.-H. Zou, D. L. Priebbenow, L. Wang, J. Mottweiler, C. Bolm, *Advanced Synthesis & Catalysis* **2013**, *355* (*13)*, 2558–2563.

[16] J. Kuang, T. Zhou, T. You, J. Chen, C. Su, Y. Xia, *Organic & Biomolecular Chemistry* **2019**, *17* (*16)*, 3940–3944.

[17] K. Kumpan, A. Nathubhai, C. Zhang, P. J. Wood, M. D. Lloyd, A. S. Thompson, T. Haikarainen, L. Lehtiö, M. D. Threadgill, *Bioorganic & Medicinal Chemistry* **2015**, *23* (*13)*, 3013–3032.

[18] S. Matsubara, K. Nomura, K. Asano, T. Kurahashi, *HETEROCYCLES* **2008**, *76* (*2)*, 1381.

[19] T. Yamada, T. Nagata, K. D. Sugi, K. Yorozu, T. Ikeno, Y. Ohtsuka, D. Miyazaki, T. Mukaiyama, *Chemistry (Weinheim an der Bergstrasse, Germany)* **2003**, *9* (*18)*, 4485–4509.

[20] N. V. Dubrovina, V. I. Tararov, A. Monsees, R. Kadyrov, C. Fischer, A. Börner, *Tetrahedron: Asymmetry* **2003**, *14* (*18)*, 2739–2745.

[21] Z. He, X. Qi, S. Li, Y. Zhao, G. Gao, Y. Lan, Y. Wu, J. Lan, J. You, *Angewandte Chemie (International ed. in English)* **2015**, *54* (*3)*, 855–859.

[22] A. Ishii, J. Nakayama, J. Kazami, Y. Ida, T. Nakamura, M. Hoshino, *The Journal of Organic Chemistry* **1991**, *56* (*1)*, 78–82.

[23] S. Sulzer-Mossé, A. Alexakis, J. Mareda, G. Bollot, G. Bernardinelli, Y. Filinchuk, *Chemistry (Weinheim an der Bergstrasse, Germany)* **2009**, *15* (*13)*, 3204–3220.

[24] M. Kirihara, T. Goto, T. Noguchi, M. Suzuki, Y. Ishizuka, S. Naito, *Chemical & Pharmaceutical Bulletin* **2013**, *61* (*4)*, 460–463.

[25] A. Quintard, A. Alexakis, C. Mazet, *Angewandte Chemie (International ed. in English)* **2011**, *50* (*10)*, 2354–2358.

[26] L. A. Carpino, *The Journal of Organic Chemistry* **1973**, *38* (*15)*, 2600–2603.

[27] A. Bugarin, K. D. Jones, B. T. Connell, *Chemical Communications* **2010**, *46* (*10)*, 1715–1717.

[28] D. G. Stark, L. C. Morrill, D. B. Cordes, A. M. Z. Slawin, T. J. C. O'Riordan, A. D. Smith, *Chemistry, an Asian journal* **2016**, *11* (*3)*, 395–400.

[29] D. J. Smith, G. P. A. Yap, J. A. Kelley, J. P. Schneider, *The Journal of Organic Chemistry* **2011**, *76* (*6)*, 1513–1520.

[30] N. Kaur, P. Singh, P. Banerjee, *Advanced Synthesis & Catalysis* **2021**, *363* (*11)*, 2813–2824.

[31] J. Li, K. Wang, J. Wu, H. Zhang, Y. Chen, Q. Liu, J. Xu, W. Yi, *European Journal of Organic Chemistry* **2022**, *2022* (*18)*.

[32] Y. Lou, P. Cao, T. Jia, Y. Zhang, M. Wang, J. Liao, *Angewandte Chemie (International ed. in English)* **2015**, *54* (*41)*, 12134–12138.

[33] L. Roiser, M. Waser, *Organic Letters* **2017**, *19* (*9)*, 2338–2341.

[34] L. I. Pilkington, D. Barker, *European Journal of Organic Chemistry* **2014**, *2014* (*5)*, 1037–1046.

[35] S. Companys, P. A. Peixoto, C. Bosset, S. Chassaing, K. Miqueu, J.-M. Sotiropoulos, L. Pouységu, S. Quideau, *Chemistry (Weinheim an der Bergstrasse, Germany)* **2017**, *23* (*54)*, 13309–13313.

[36] J. N. Arokianathar, W. C. Hartley, C. McLaughlin, M. D. Greenhalgh, D. Stead, S. Ng, A. M. Z. Slawin, A. D. Smith, *Molecules* **2021**, *26* (*21)*, 6333.

[37] M. Winter, R. Schütz, A. Eitzinger, A. R. Ofial, M. Waser, *European Journal of Organic Chemistry* **2020**, *2020* (*25)*, 3812–3817.

[38] S. Marchart, A. Gromov, J. Mulzer, *Angewandte Chemie (International ed. in English)* **2010**, *49* (*11)*, 2050–2053.

[39] M. Tiffner, J. Novacek, A. Busillo, K. Gratzer, A. Massa, M. Waser, *RSC Advances* **2015**, *5* (*96)*, 78941–78949.

[40] X.-Z. Zhang, Y.-H. Deng, X. Yan, K.-Y. Yu, F.-X. Wang, X.-Y. Ma, C.-A. Fan, *The Journal of Organic Chemistry* **2016**, *81* (*13)*, 5655–5662.

[41] G. M. Sheldrick, *Acta Crystallographica. Section A, Foundations of Crystallography* **2008**, *64* (*Pt 1)*, 112–122.

[42] G. M. Sheldrick, *Acta Crystallographica. Section C, Structural Chemistry* **2015**, *71* (*Pt 1)*, 3–8.

[43] M. A. Marangoni, C. E. Bencke, H. G. Bonacorso, M. A. Martins, N. Zanatta, *Tetrahedron Letters* **2018**, *59* (*2)*, 121–124.

[44] A. Guerrero-Corella, F. Esteban, M. Iniesta, A. Martín-Somer, M. Parra, S. Díaz-Tendero, A. Fraile, J. Alemán, *Angewandte Chemie (International ed. in English)* **2018**, *57* (*19)*, 5350–5354.

[45] G. M. Sheldrick, *Acta Crystallographica. Section A, Foundations and Advances* **2015**, *71* (*Pt 1)*, 3–8.

[46] O. V. Dolomanov, L. J. Bourhis, R. J. Gildea, J. A. K. Howard, H. Puschmann, *Journal of Applied Crystallography* **2009**, *42* (*2)*, 339–341.

# 6 Appendix: Analytical data

**1,3-Bis(4-(dimethylamino)phenyl)propane-1,3-dione (S26)**

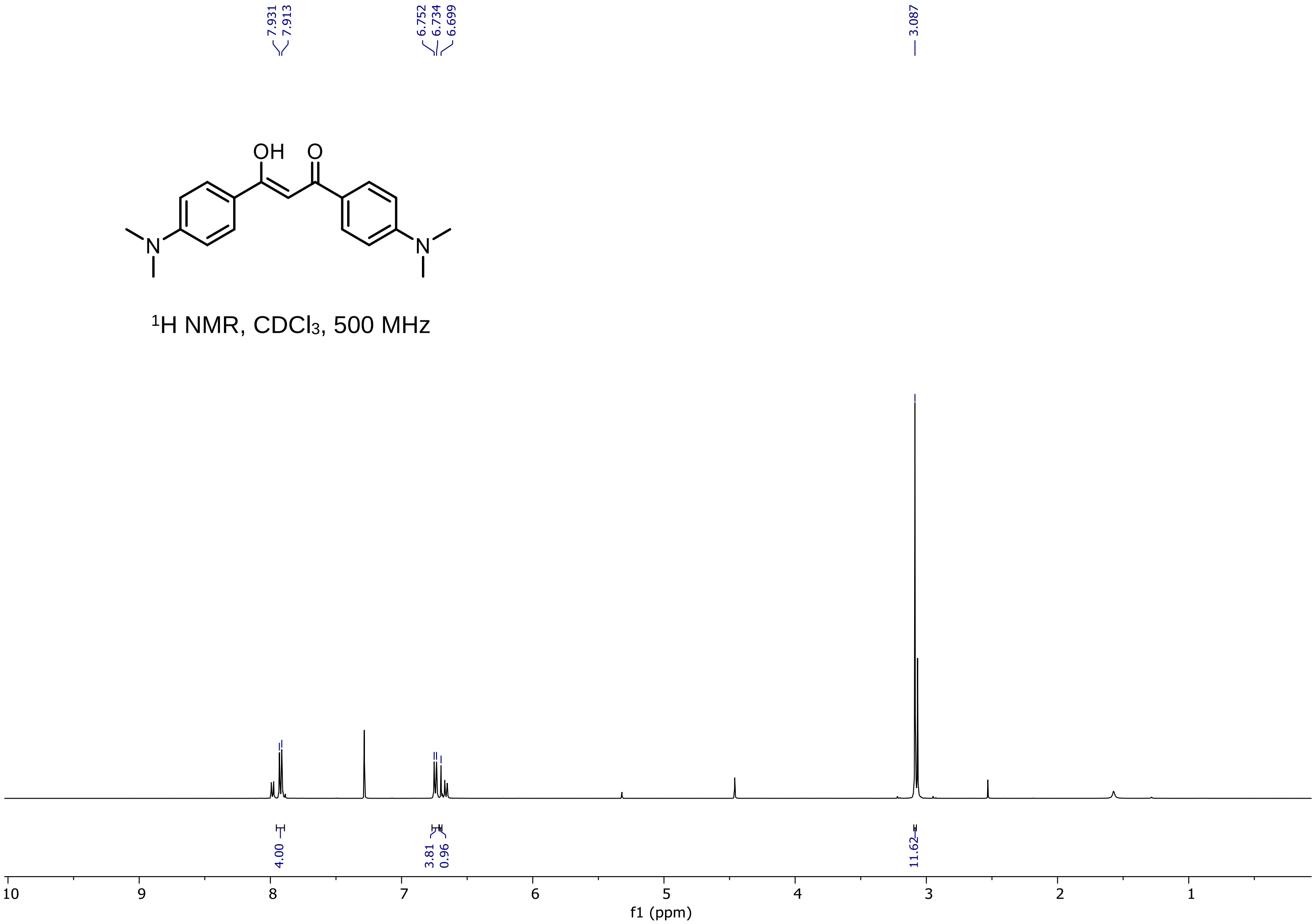


$^{1}H$ NMR, $CDCl_3$, 500 MHz

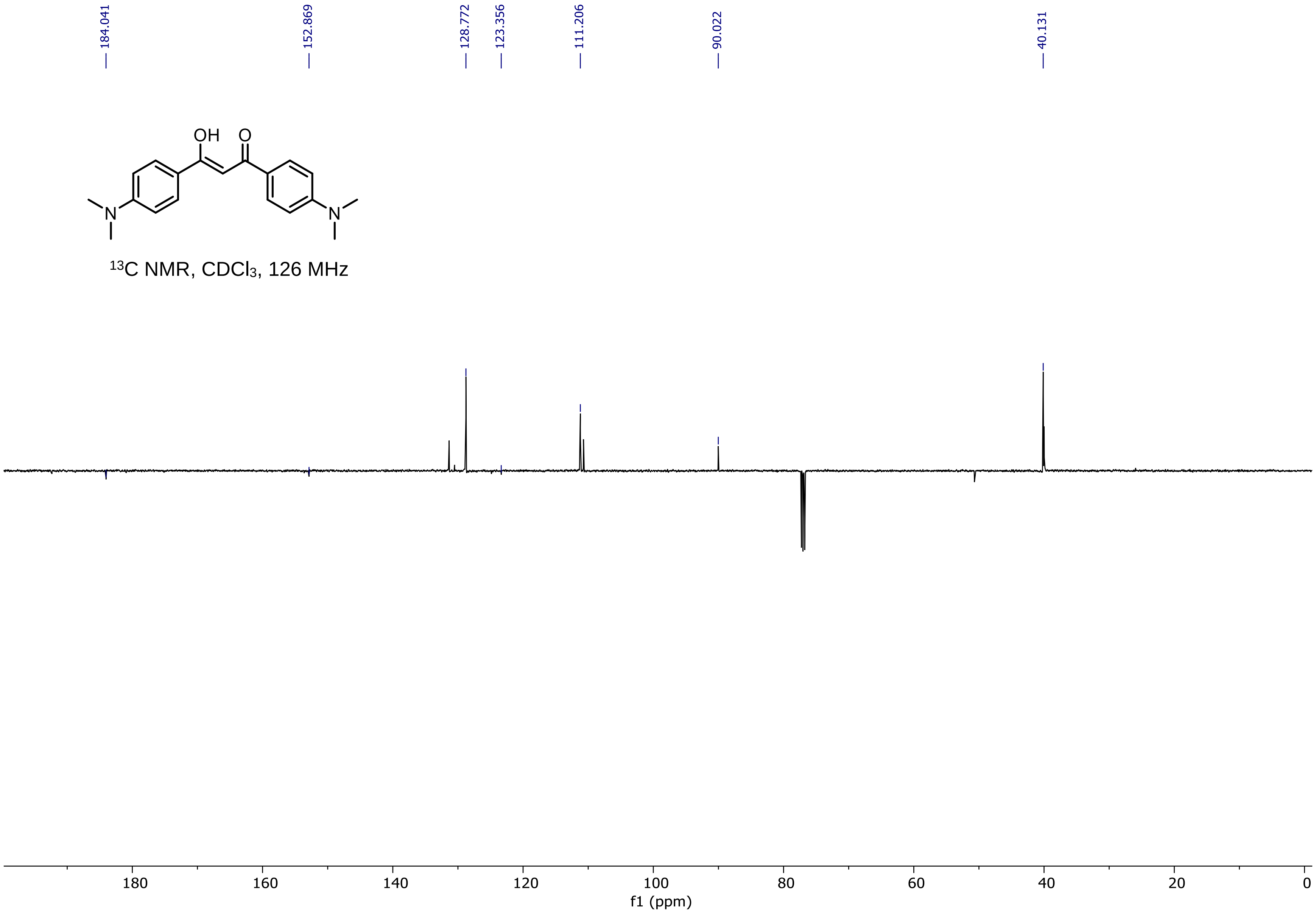
184.041
152.869
128.772
123.356
111.206
90.022
40.131
OH
O
N
N
13C NMR, CDCl3, 126 MHz
180
160
140
120
100
80
60
40
20
0
f1 (ppm)

**1,3-Bis(3,4-dimethoxyphenyl)propane-1,3-dione (S27)**

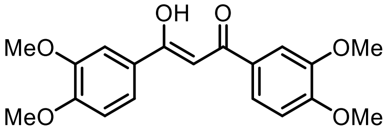


$^{1}$H NMR, $CDCl_3$, 500 MHz

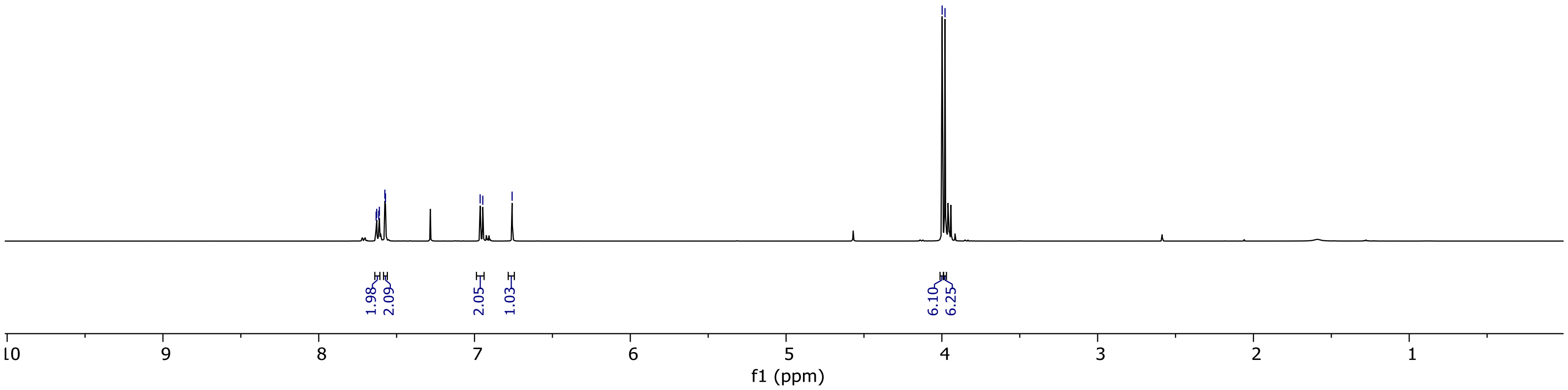

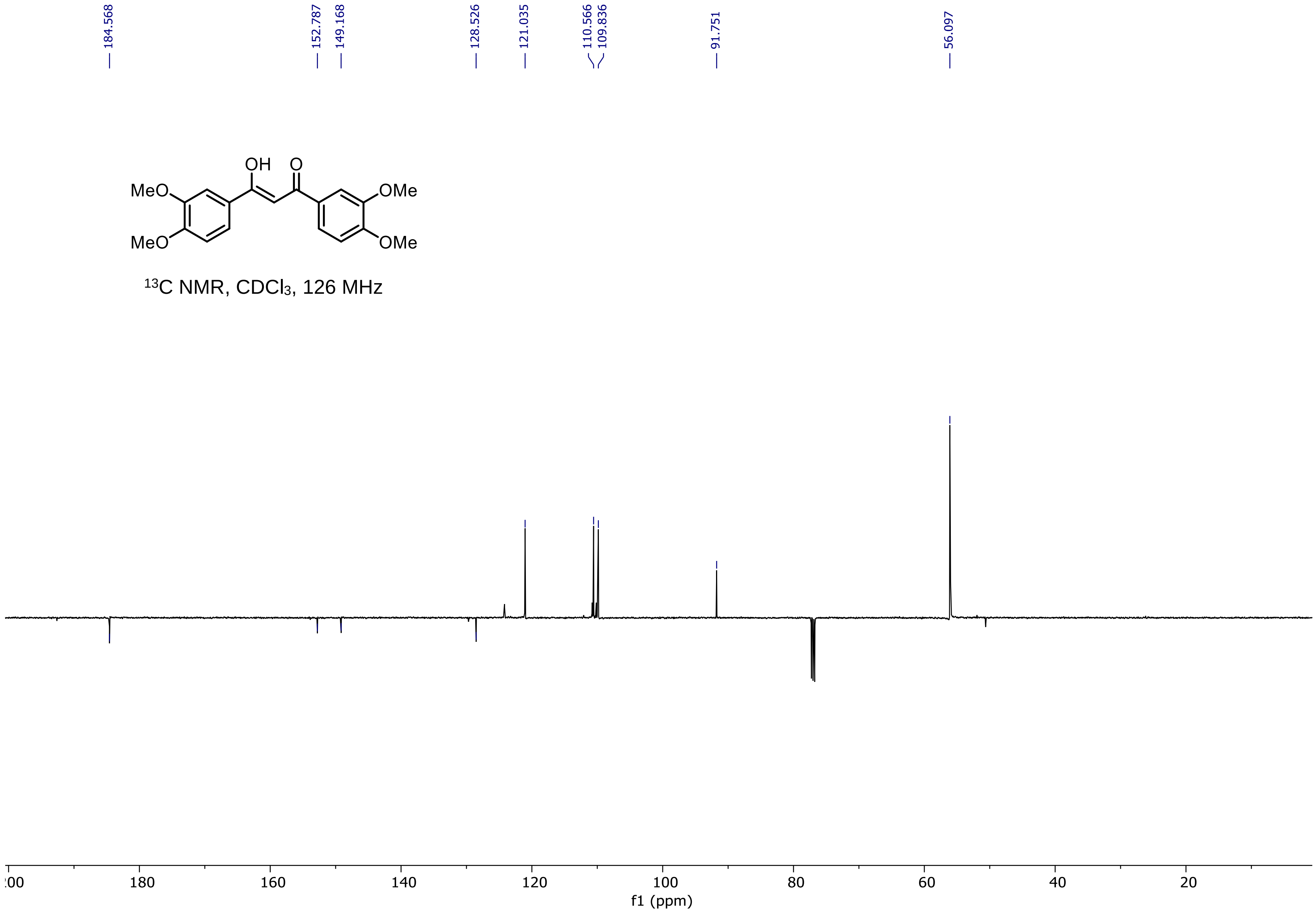


$^{13}C$ NMR, $CDCl_3$, 126 MHz

**1,3-Di(naphthalen-2-yl)propane-1,3-dione (S30)**

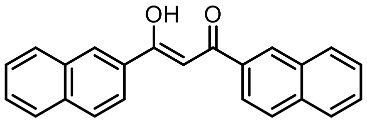


$^1$H NMR, $CDCl_3$, 500 MHz

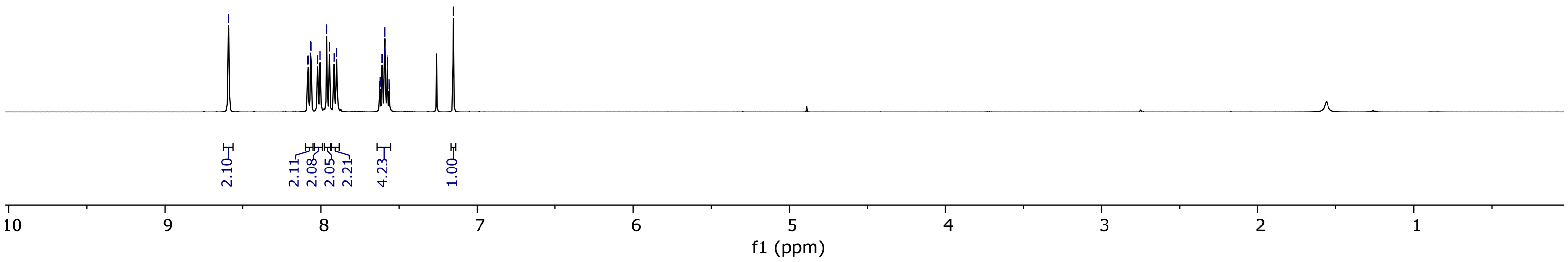

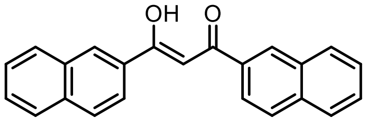

$^{13}C$ NMR, $CDCl_3$, 126 MHz

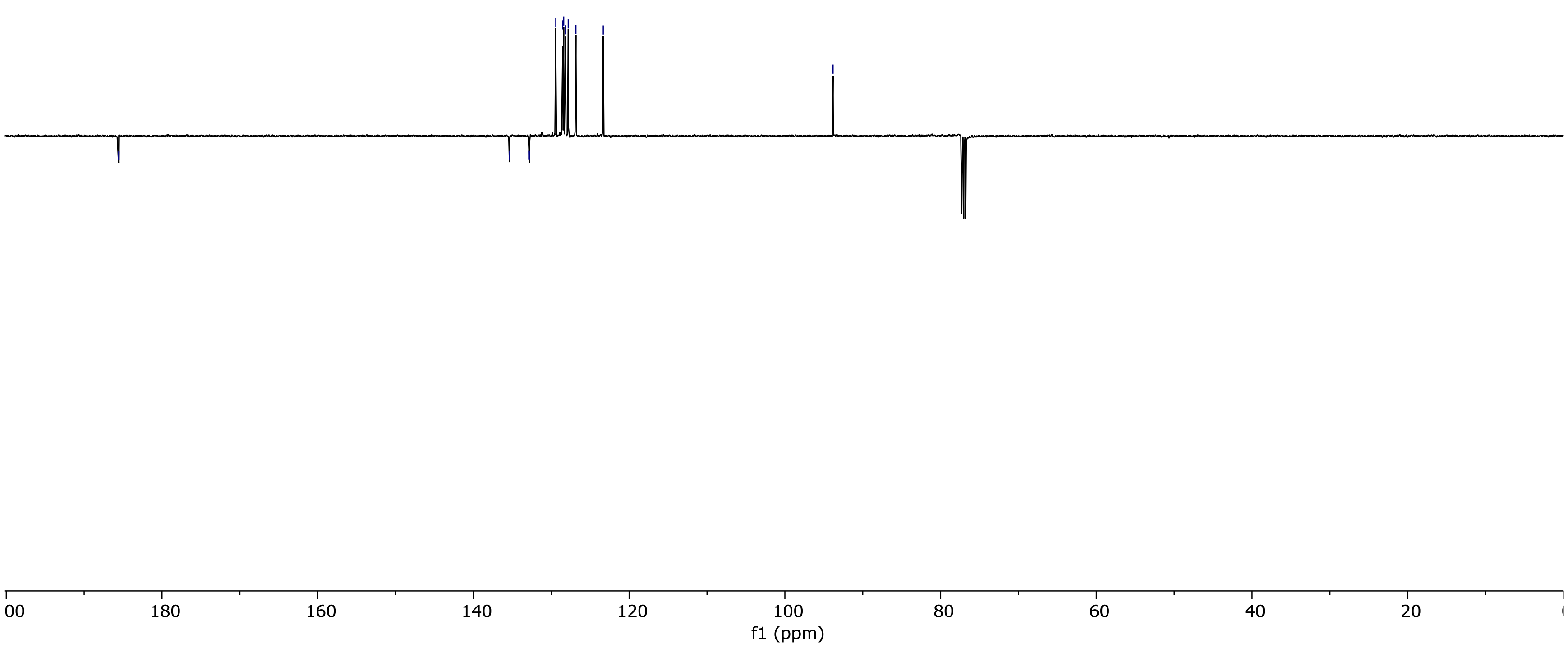

**1,3-Di(naphthalen-1-yl)propane-1,3-dione (S31)**

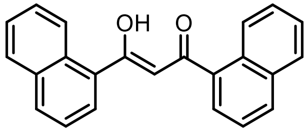


$^1$H NMR, $CDCl_3$, 500 MHz

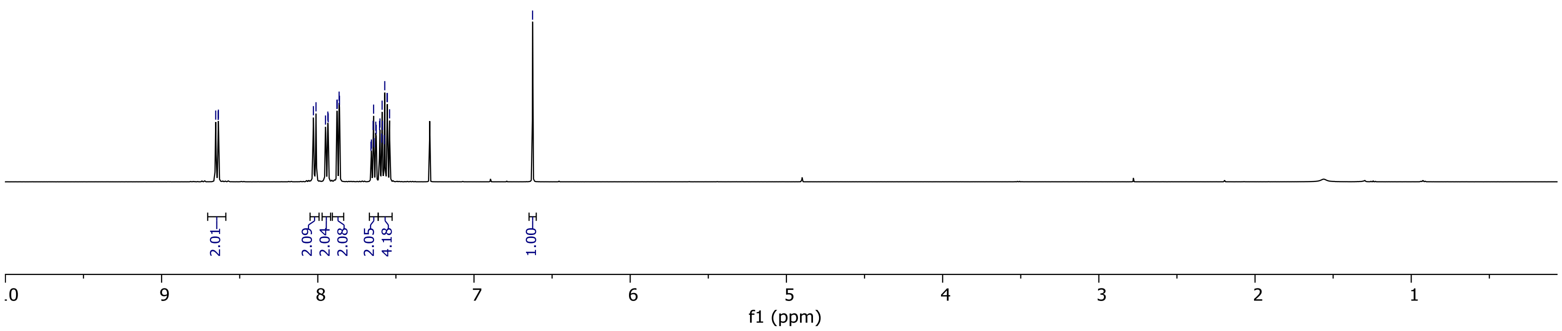

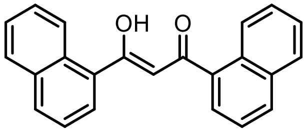

$^{13}C$ NMR, $CDCl_3$, 126 MHz

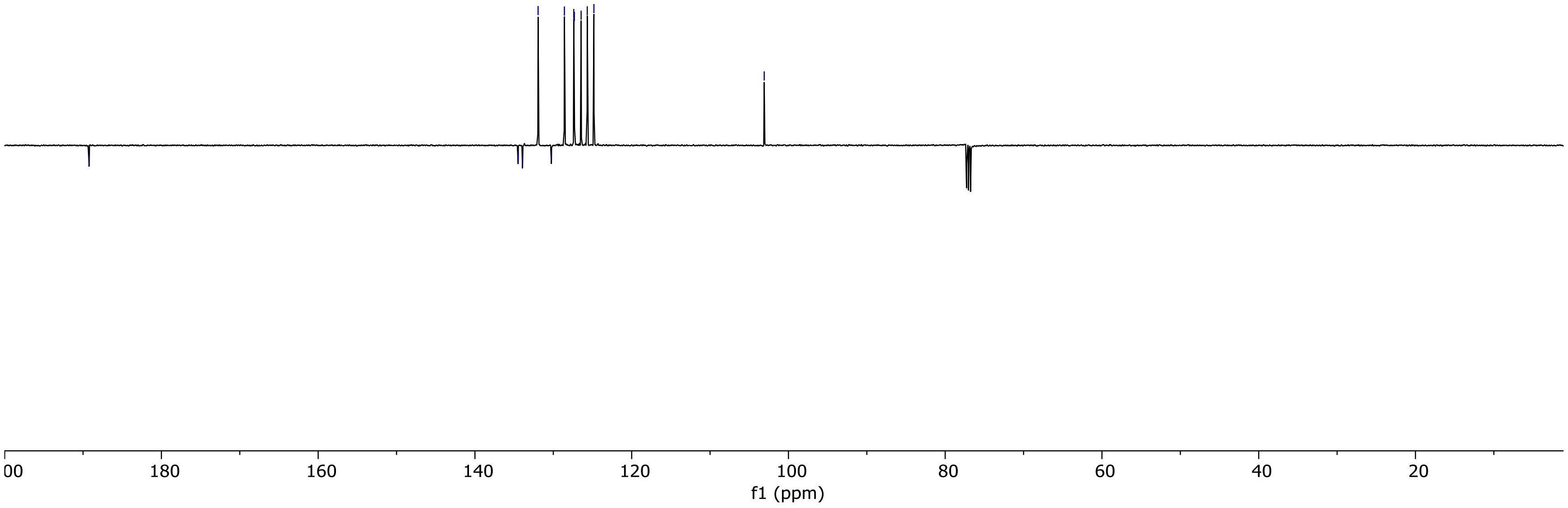

**1,3-Di(thiophen-2-yl)propane-1,3-dione (S32)**

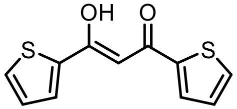

$^1$H NMR, $CDCl_3$, 500 MHz

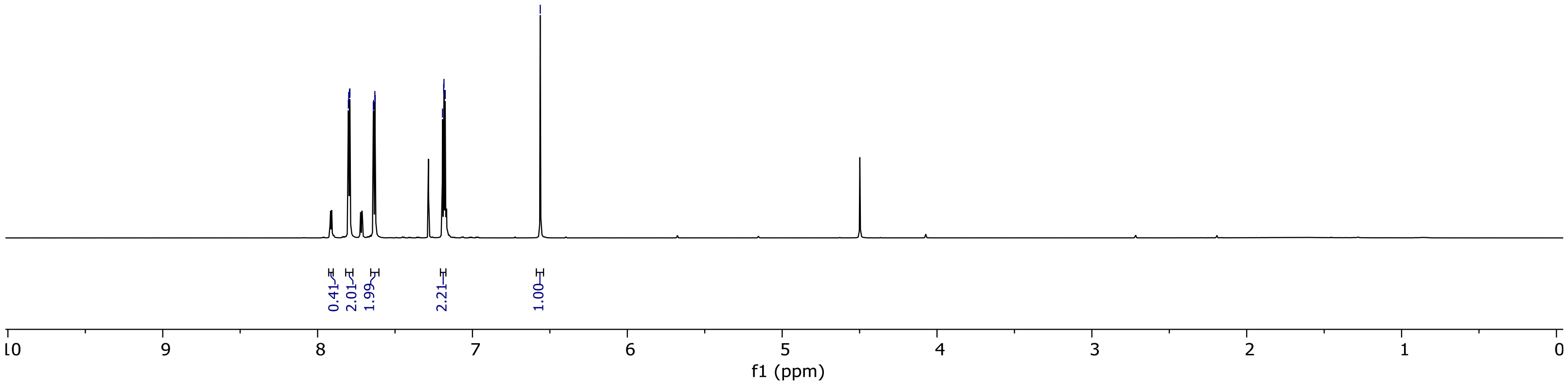

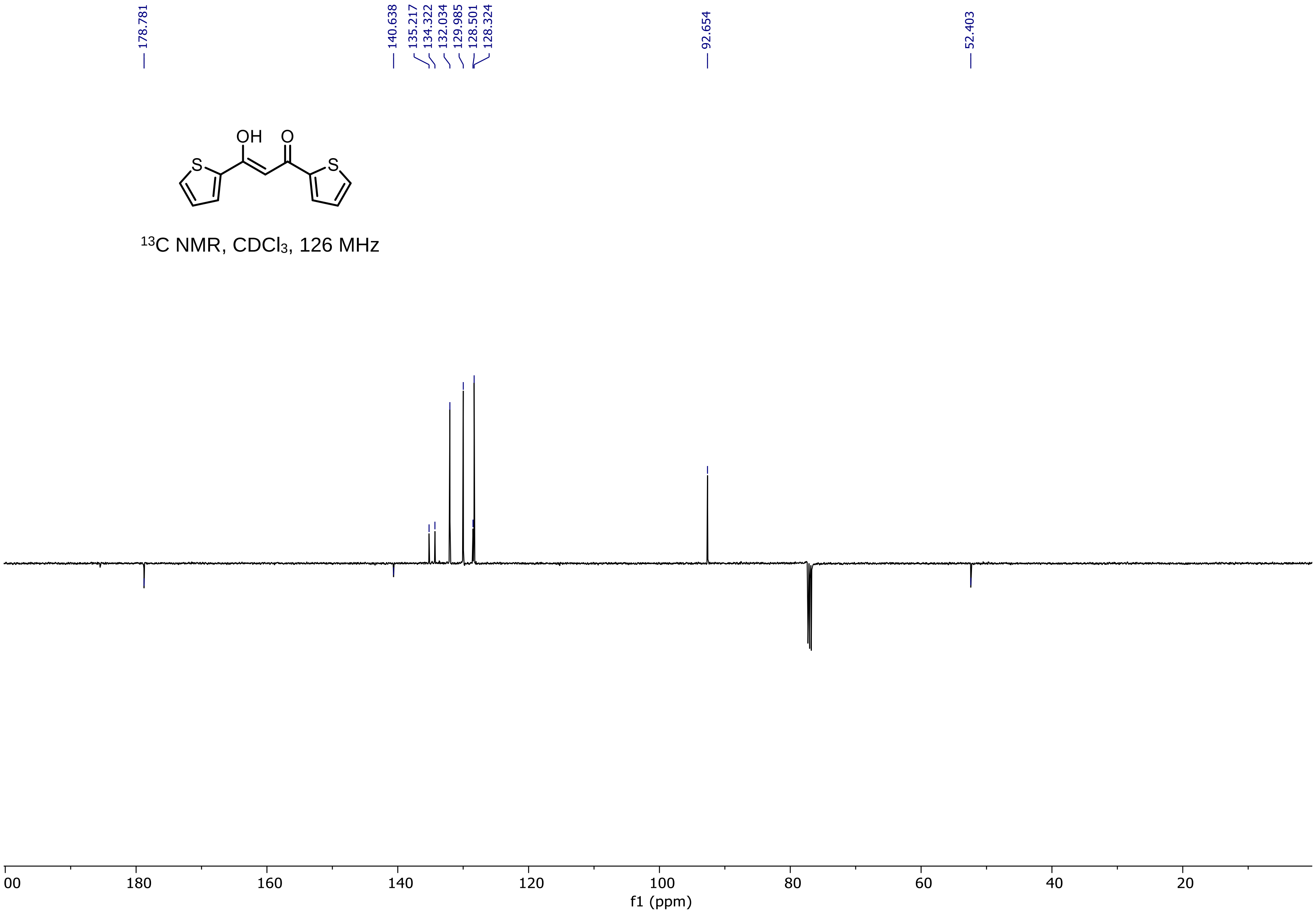
178.781
140.638
135.217
134.322
132.034
129.985
128.501
128.324
92.654
52.403
OH
O
S
S
13C NMR, CDCl3, 126 MHz
00
180
160
140
120
100
80
60
40
20
f1 (ppm)

**1,3-Di(thiophen-3-yl)propane-1,3-dione (S33)**

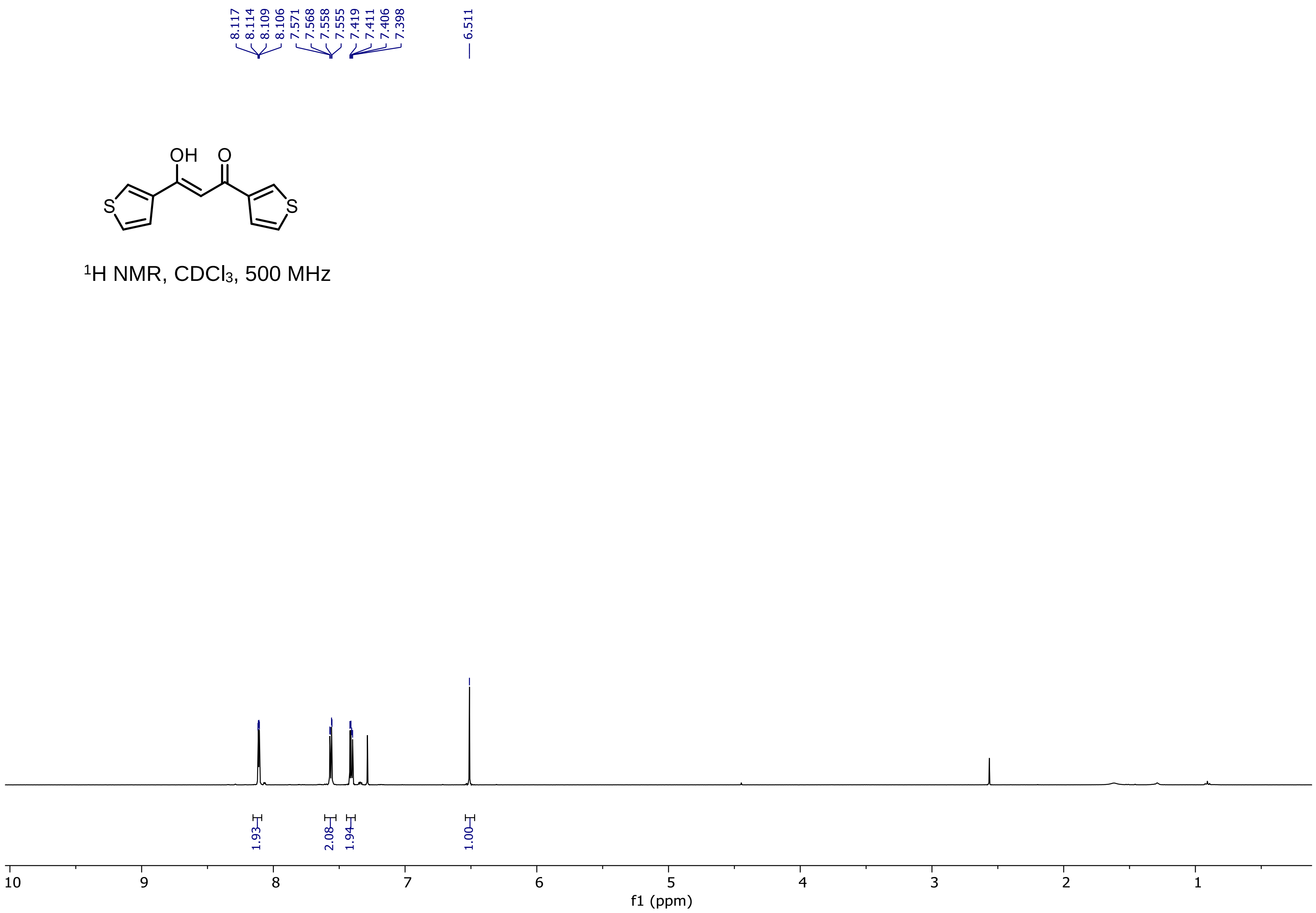

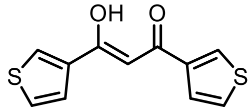

$^{13}C$ NMR, $CDCl_3$, 126 MHz

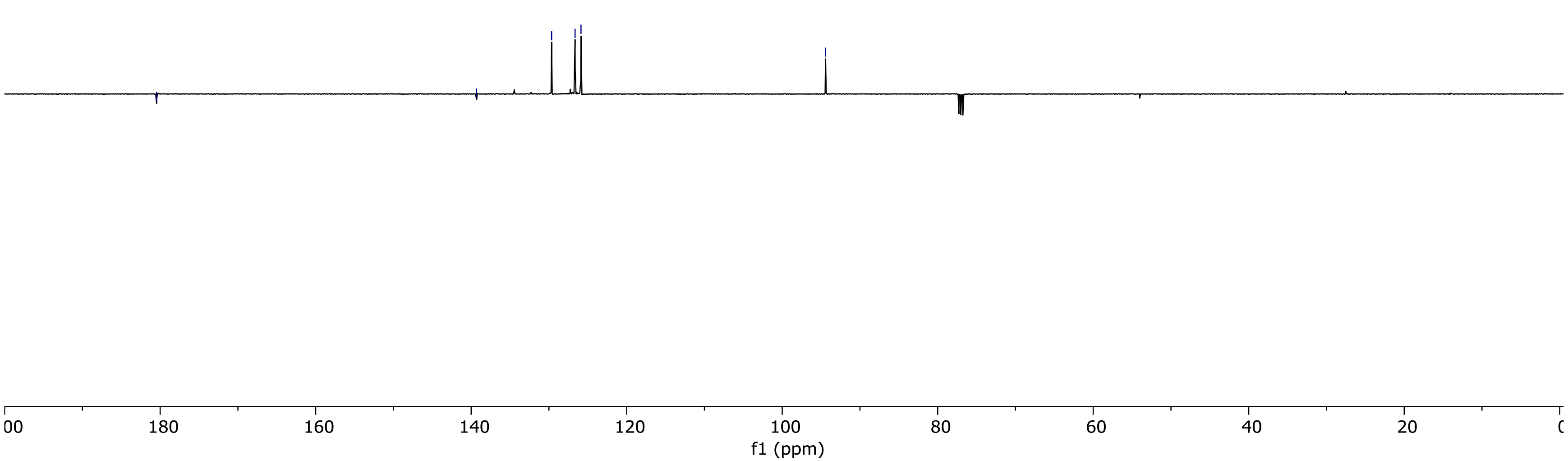

**1,3-Di(furan-2-yl)propane-1,3-dione (S34)**

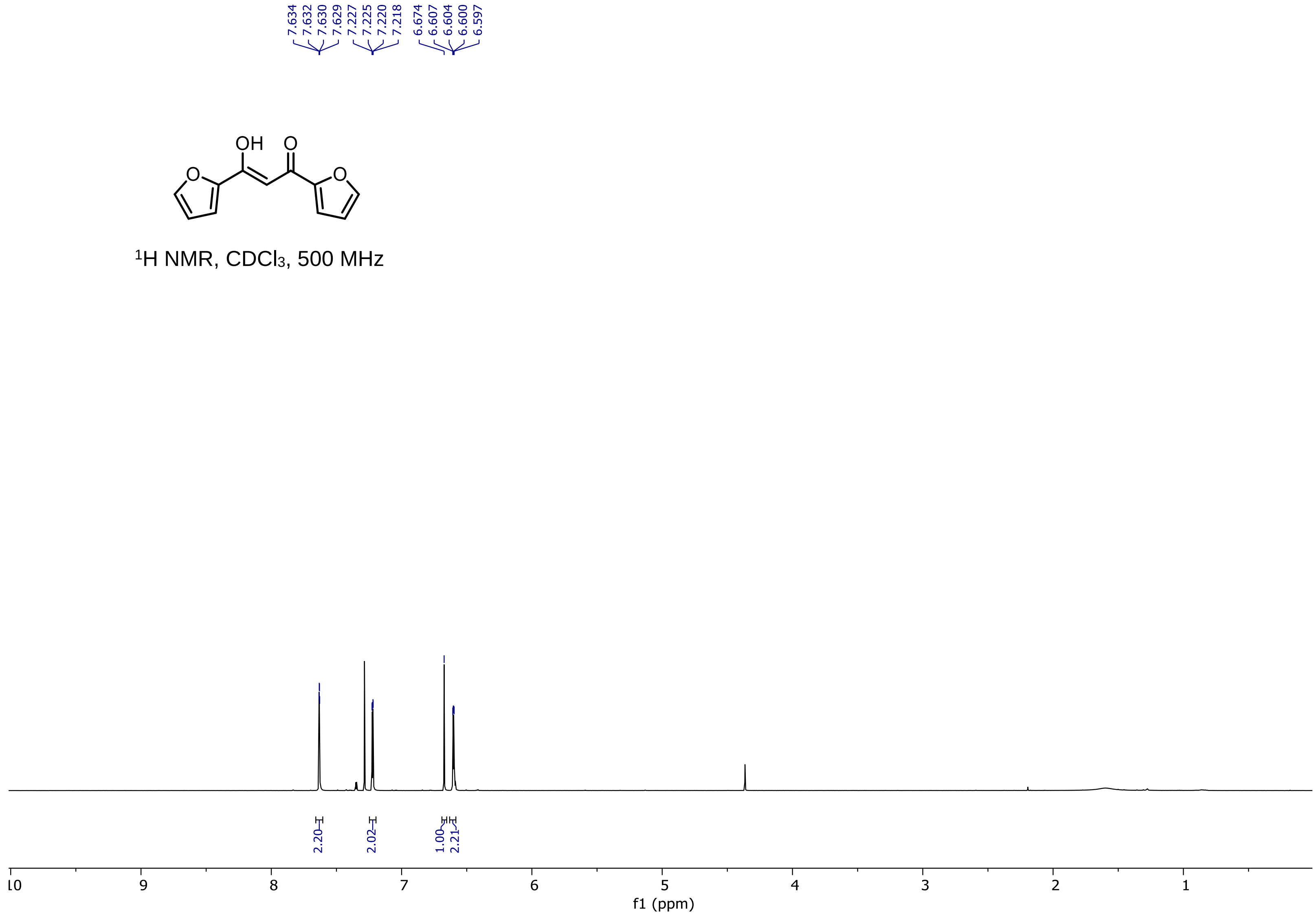

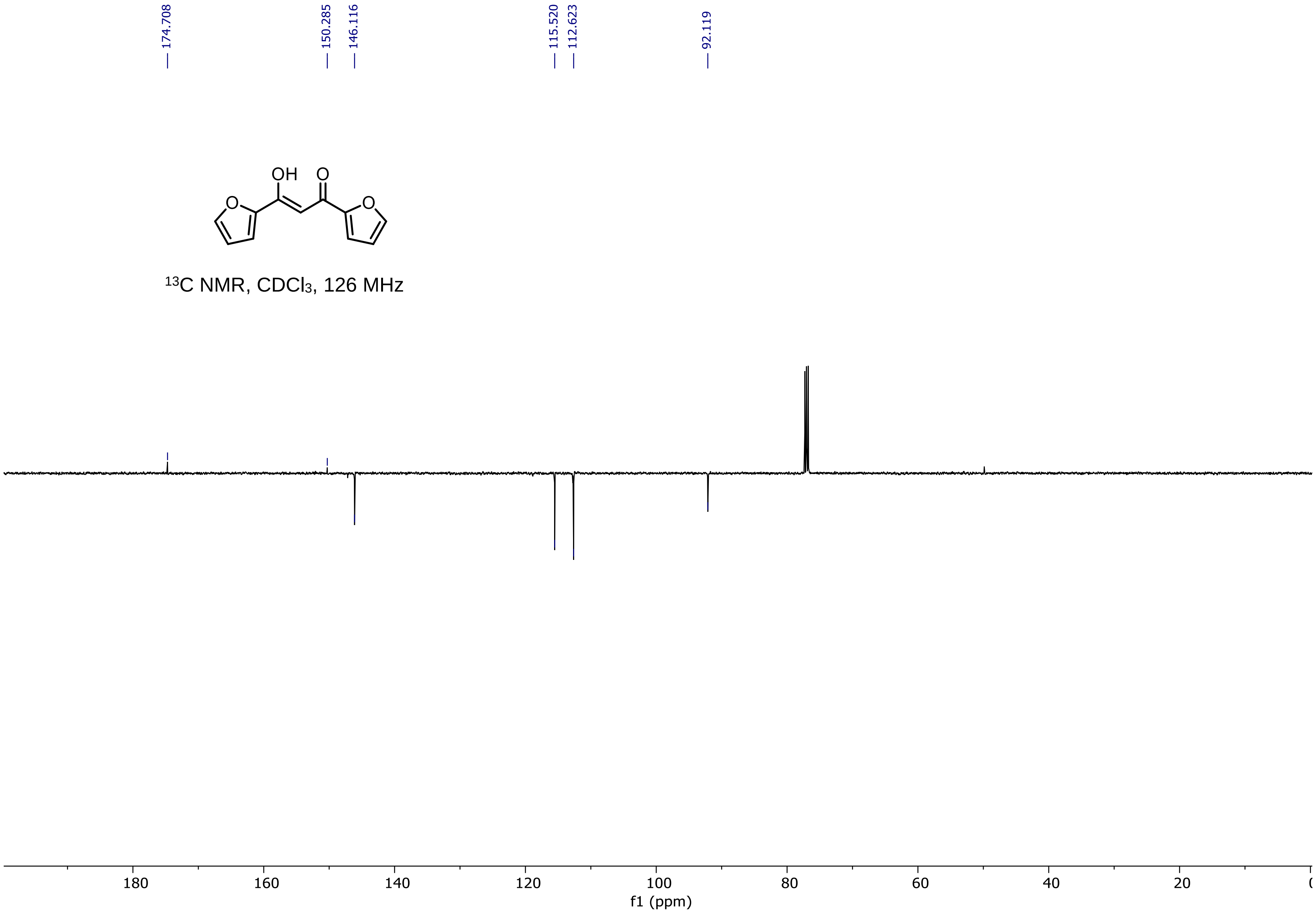

174.708
150.285
146.116
115.520
112.623
92.119
OH
O
O
O
13C NMR, CDCl3, 126 MHz
180
160
140
120
100
80
60
40
20
f1 (ppm)

**1,3-Bis(benzo[d][1,3]dioxol-5-yl)propane-1,3-dione (S35)**

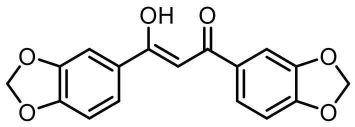


$^{1}$H NMR, $CDCl_3$, 500 MHz

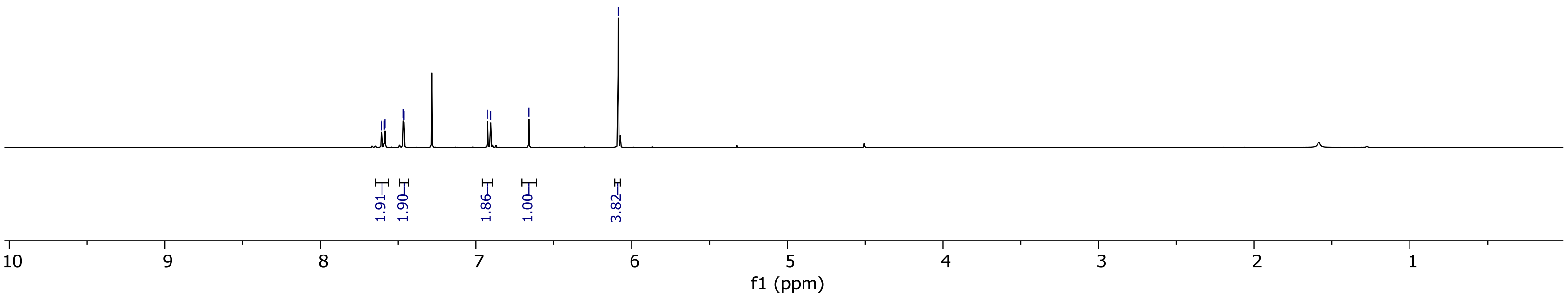

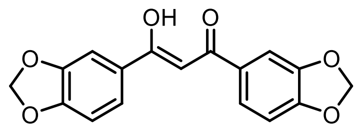

$^{13}C$ NMR, $CDCl_3$, 126 MHz

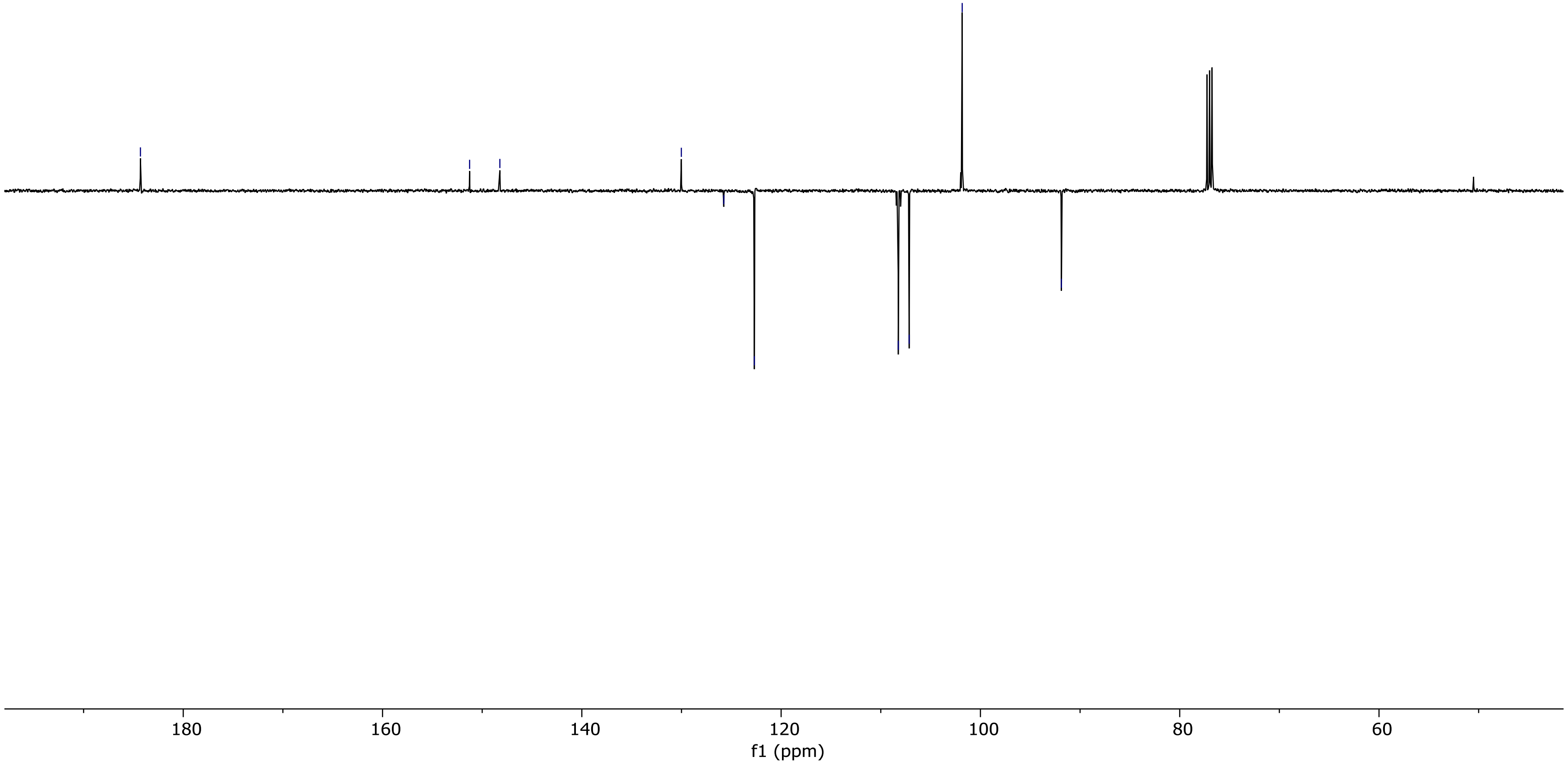

**2-Methylene-1,3-diphenylpropane-1,3-dione (S37)**

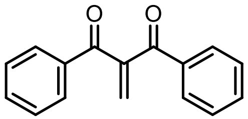

$^1$H NMR, $CDCl_3$, 500 MHz

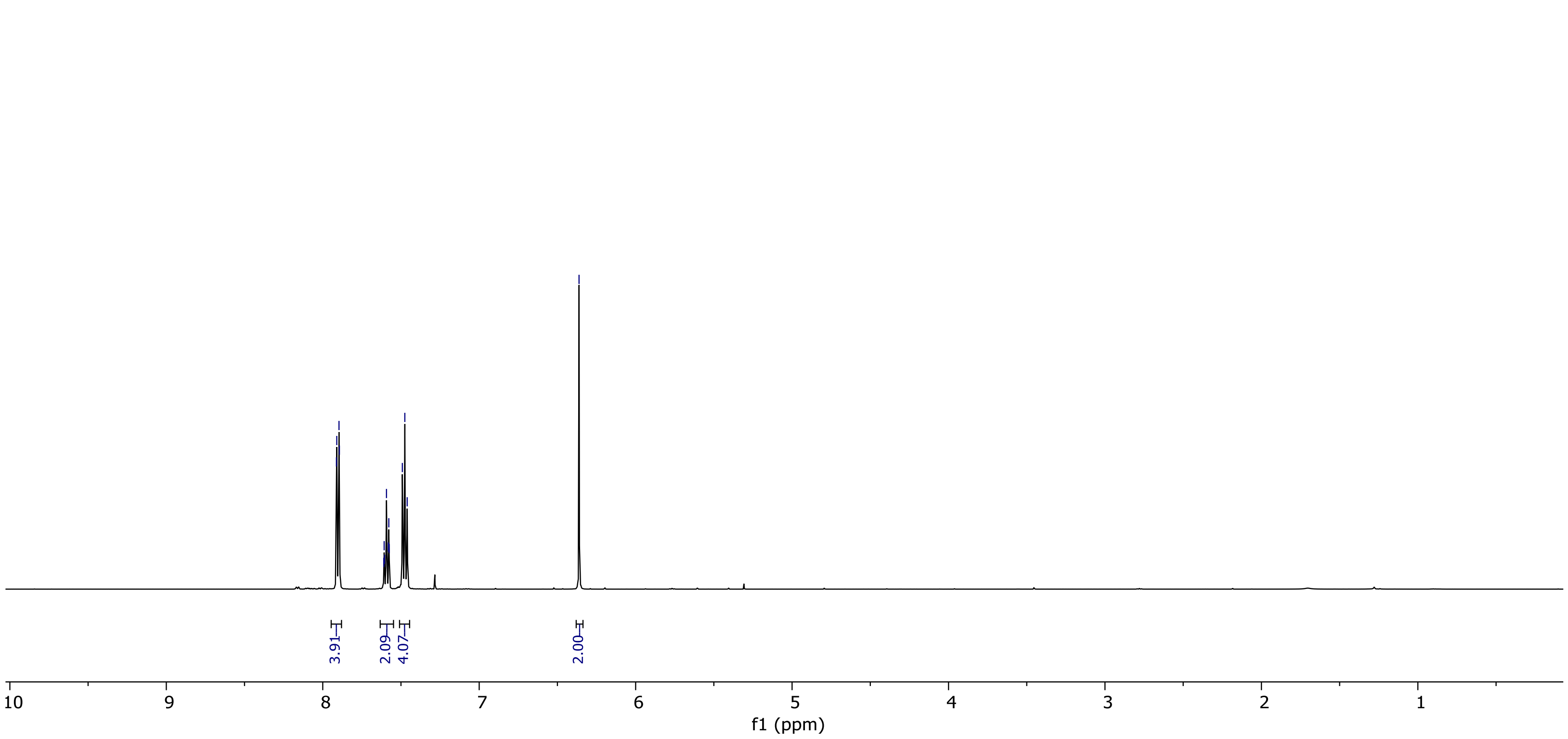

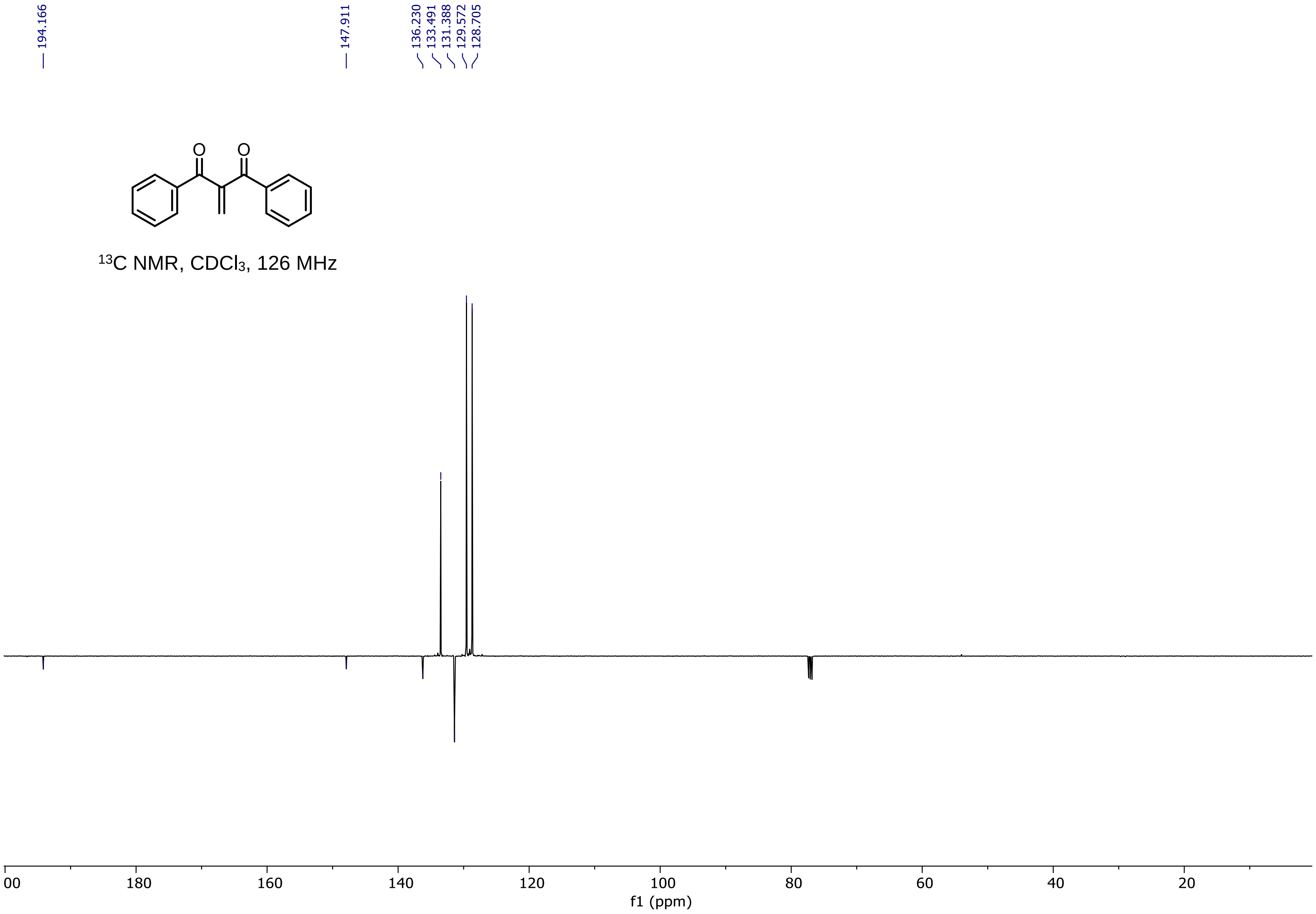
194.166
147.911
136.230
133.491
131.388
129.572
128.705
$^{13}C$ NMR, $CDCl_3$, 126 MHz
00
180
160
140
120
100
80
60
40
20
f1 (ppm)

**Di-tert-butyl 2-methylenemalonate (S39)**

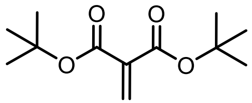

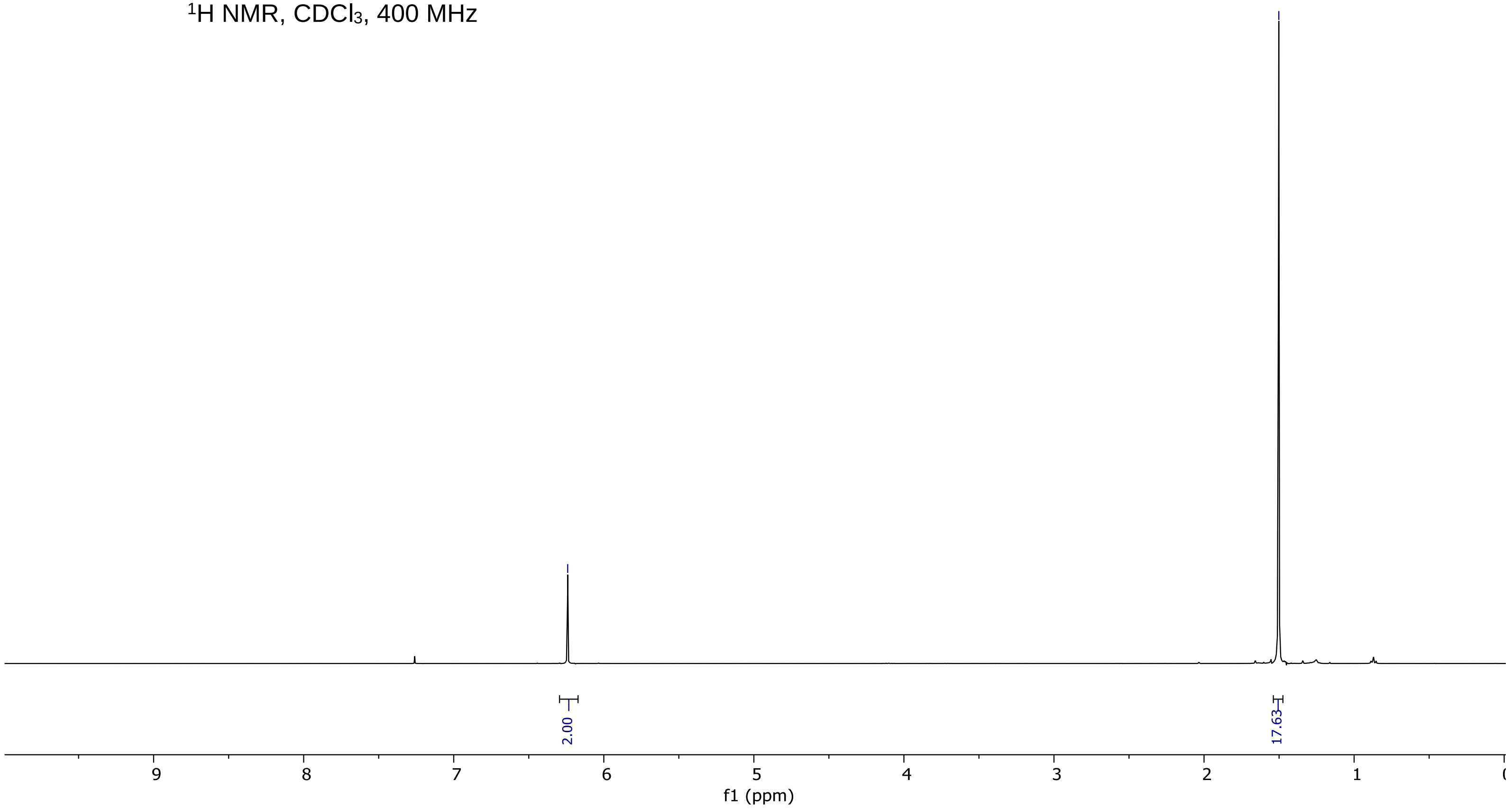

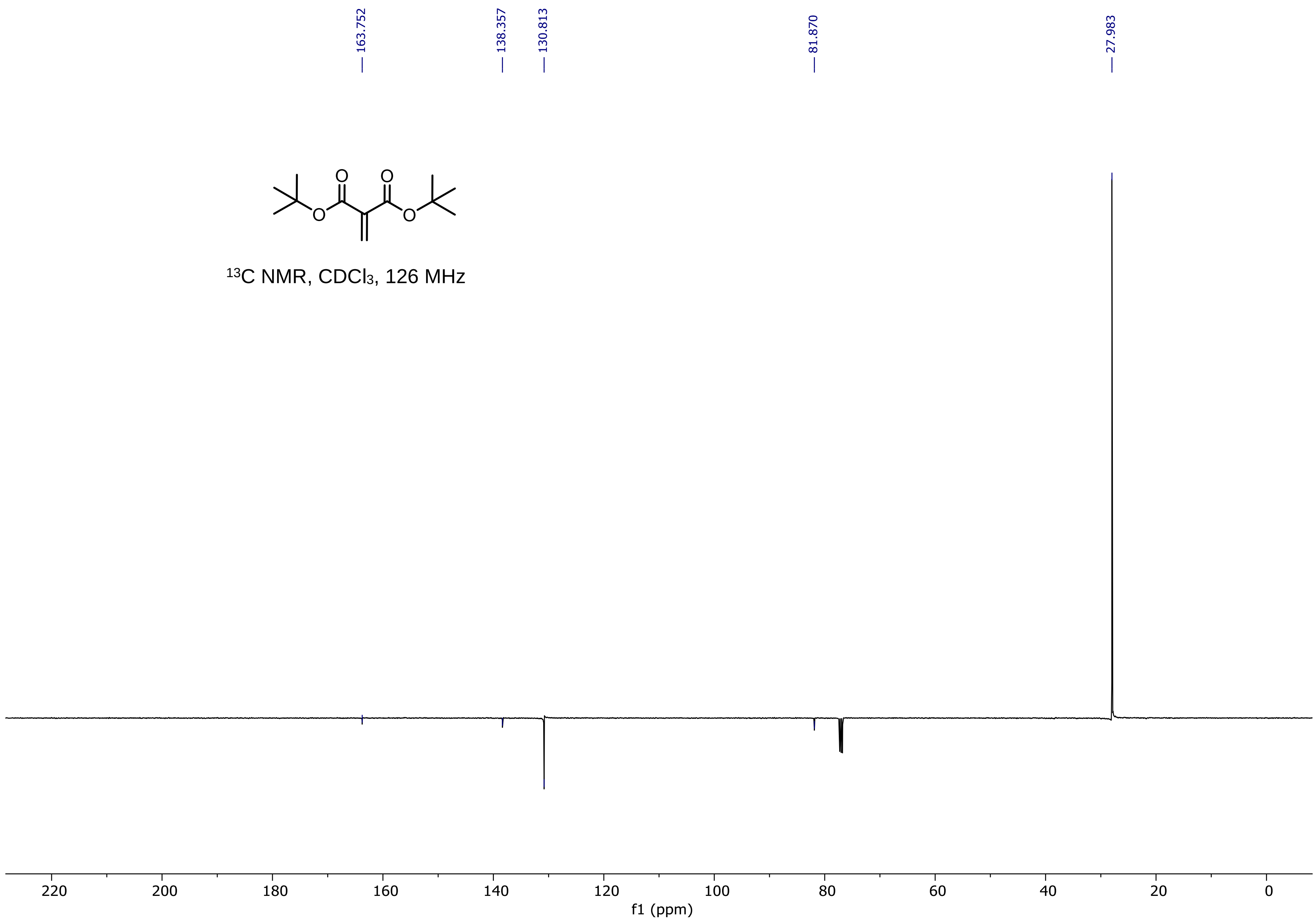
163.752
138.357
130.813
81.870
27.983
13C NMR, CDCl3, 126 MHz
220
200
180
160
140
120
100
80
60
40
20
0
f1 (ppm)

**1,3-Bis(4-methoxyphenyl)-2-methylenepropane-1,3-dione (S40)**

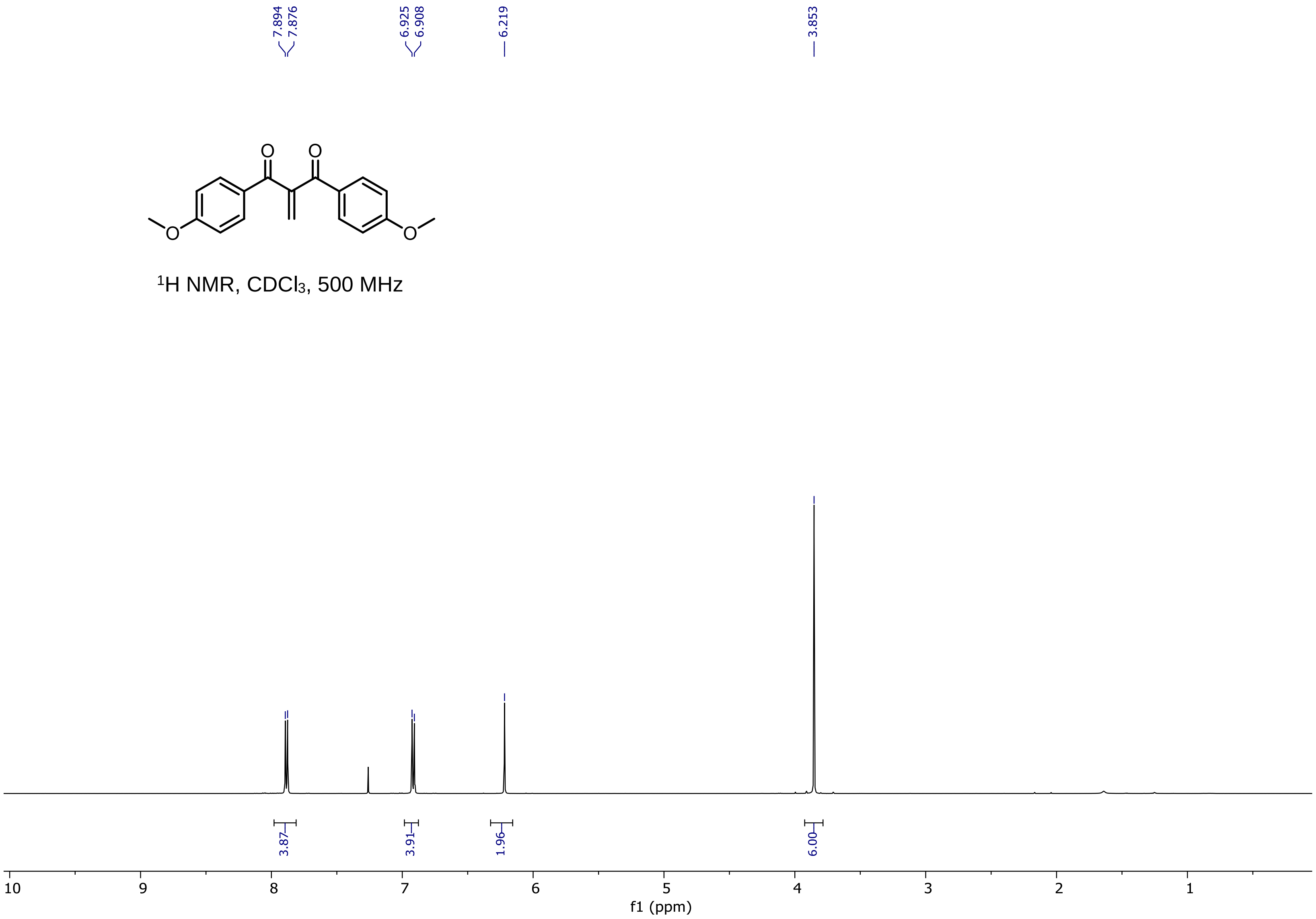

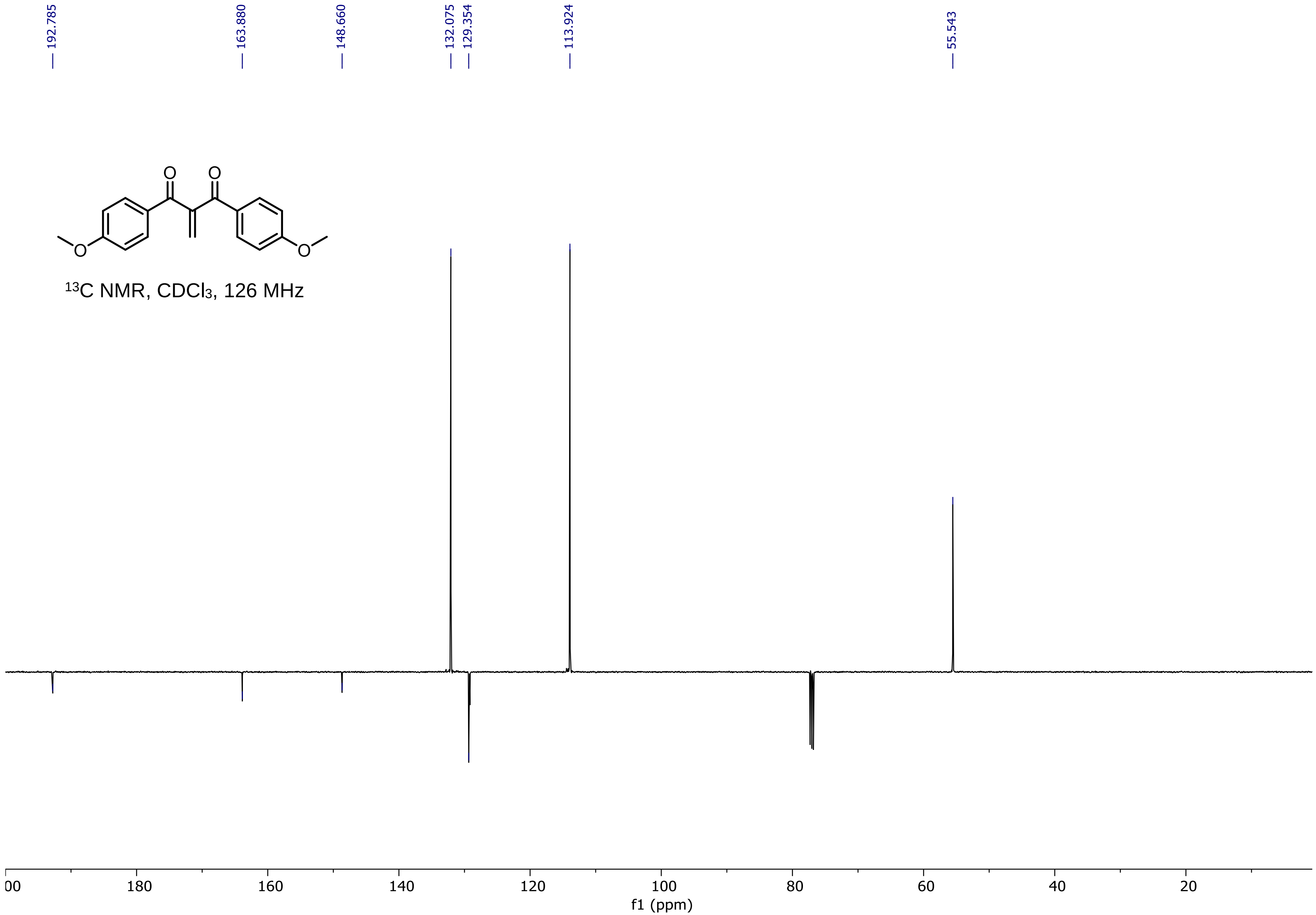
192.785
163.880
148.660
132.075
129.354
113.924
55.543
O
O
O
O
13C NMR, CDCl3, 126 MHz
00
180
160
140
120
100
80
60
40
20
f1 (ppm)

**1,3-Bis(4-(dimethylamino)phenyl)-2-methylenepropane-1,3-dione (S41)**

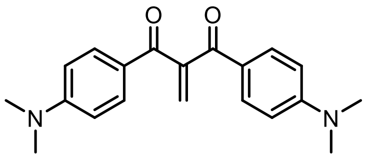


$^1$H NMR, $CDCl_3$, 500 MHz

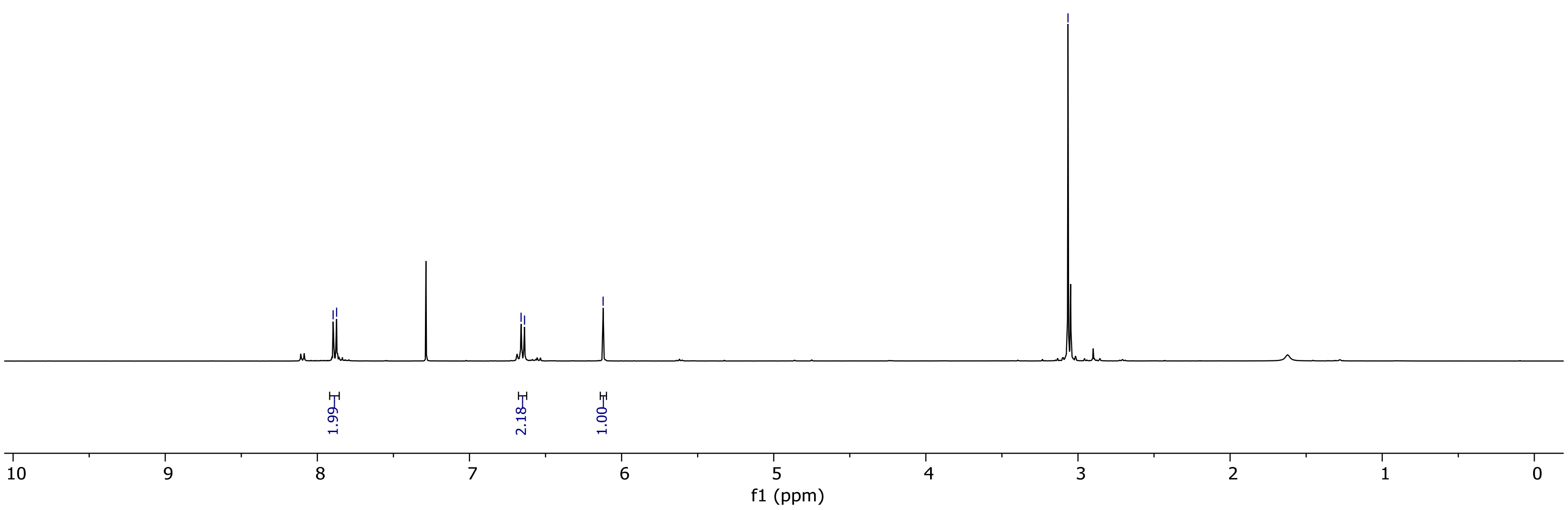

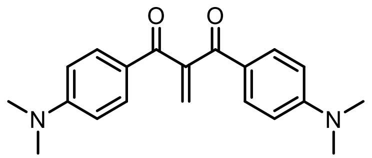

$^{13}C$ NMR, $CDCl_3$, 126 MHz

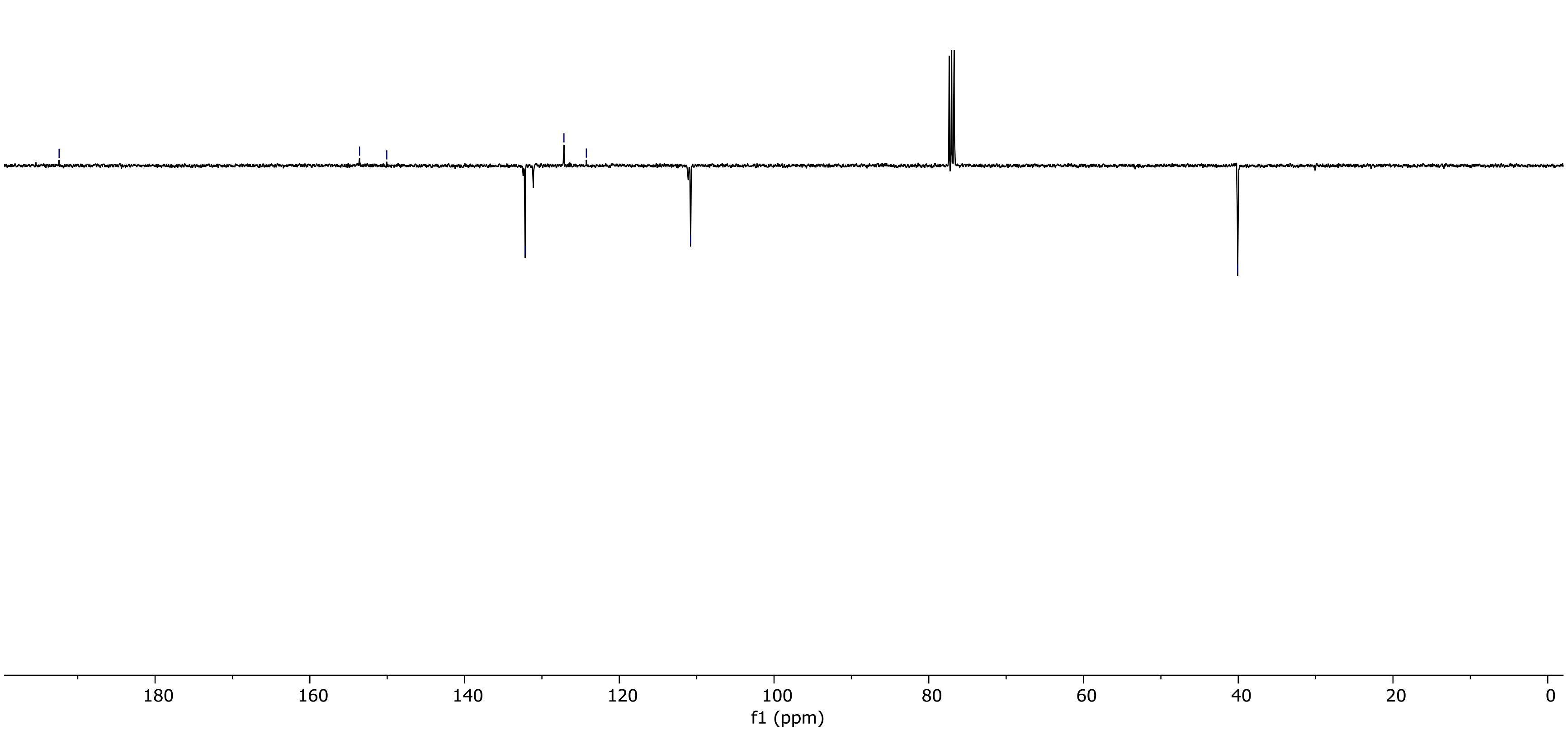

**1,3-Bis(3,4-dimethoxyphenyl)-2-methylenepropane-1,3-dione (S42)**

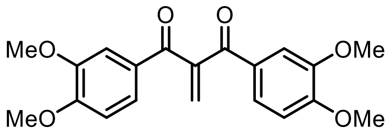


$^1$H NMR, $CDCl_3$, 500 MHz

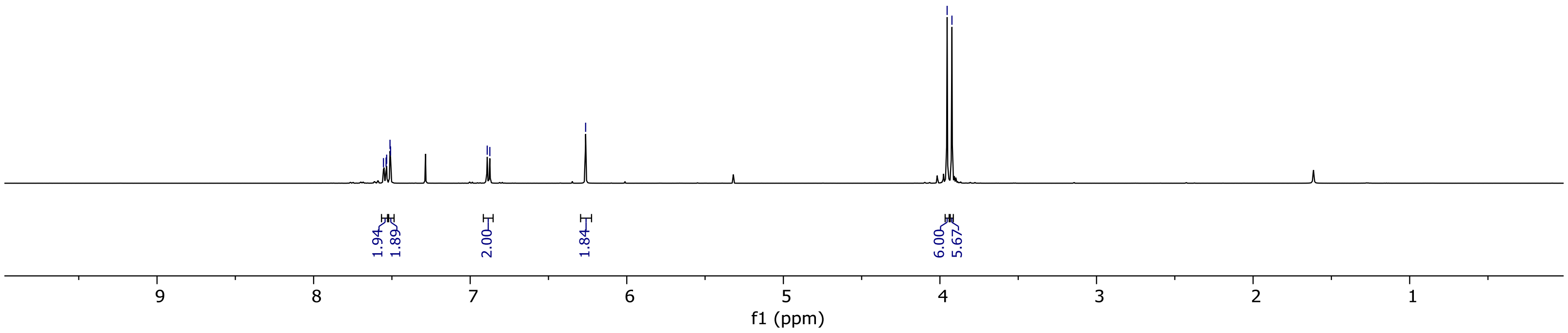

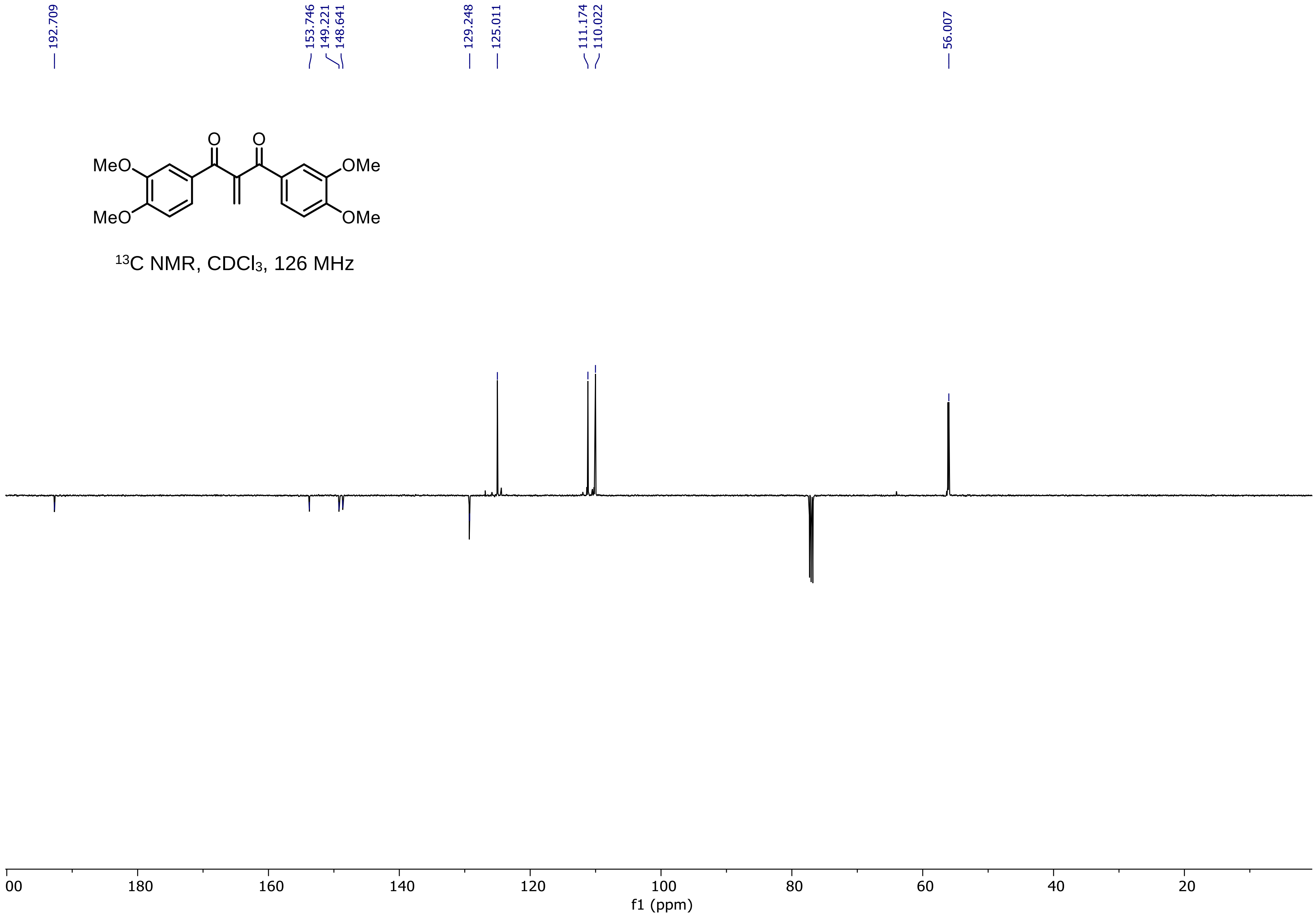
192.709
153.746
149.221
148.641
129.248
125.011
111.174
110.022
56.007
MeO
MeO
O
O
OMe
OMe
13C NMR, CDCl3, 126 MHz
00
180
160
140
120
100
80
60
40
20
f1 (ppm)

**1,3-Bis(4-bromophenyl)-2-methylenepropane-1,3-dione (S43)**

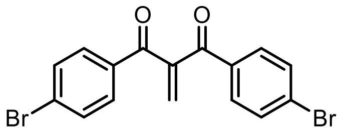


$^{1}H$ NMR, $CDCl_3$, 500 MHz

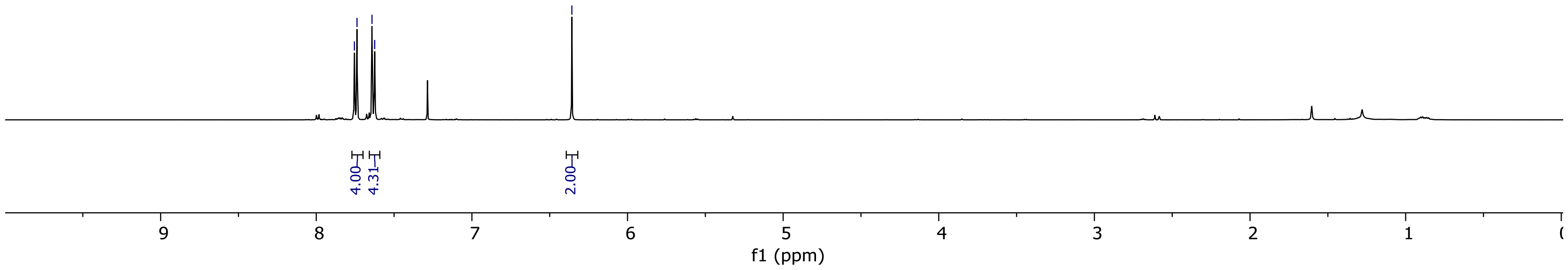

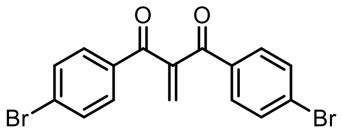


$^{13}C$ NMR, $CDCl_3$, 126 MHz

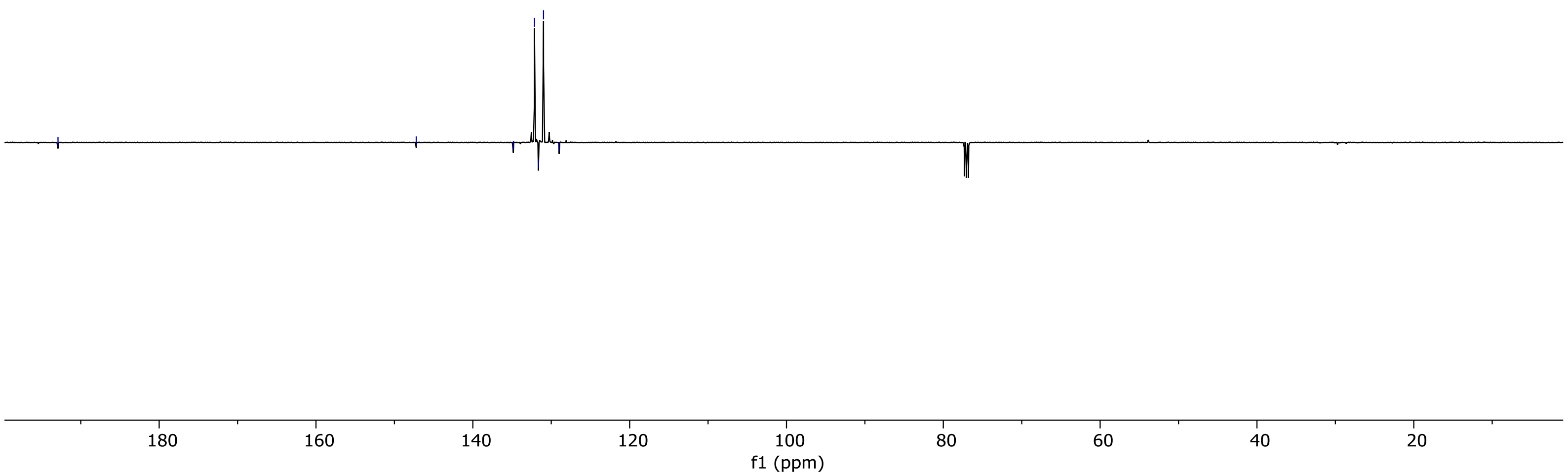

**1,3-Bis(4-chlorophenyl)-2-methylenepropane-1,3-dione (S44)**

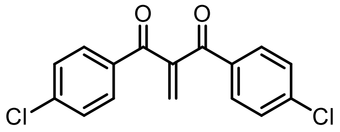

$^1$H NMR, $CDCl_3$, 500 MHz

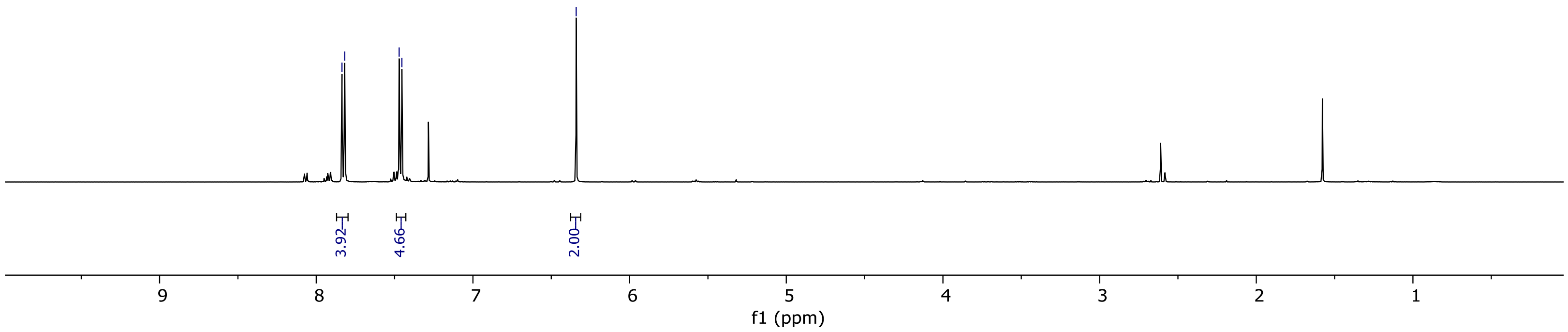

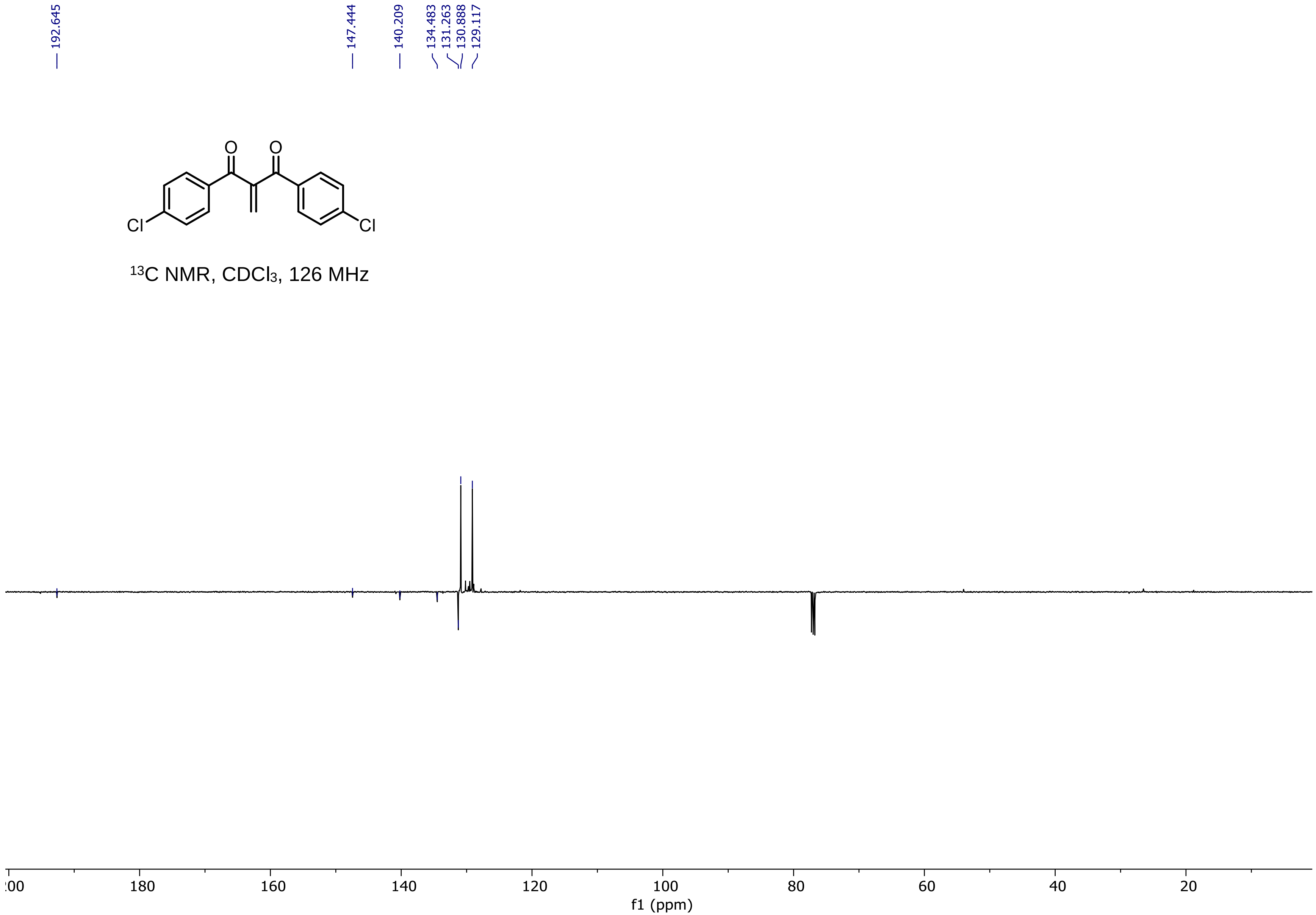


$^{13}C$ NMR, $CDCl_3$, 126 MHz

**2-Methylene-1,3-di(naphthalen-2-yl)propane-1,3-dione (S45)**

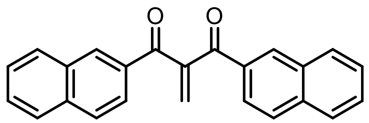

$^{1}$H NMR, $CDCl_3$, 500 MHz

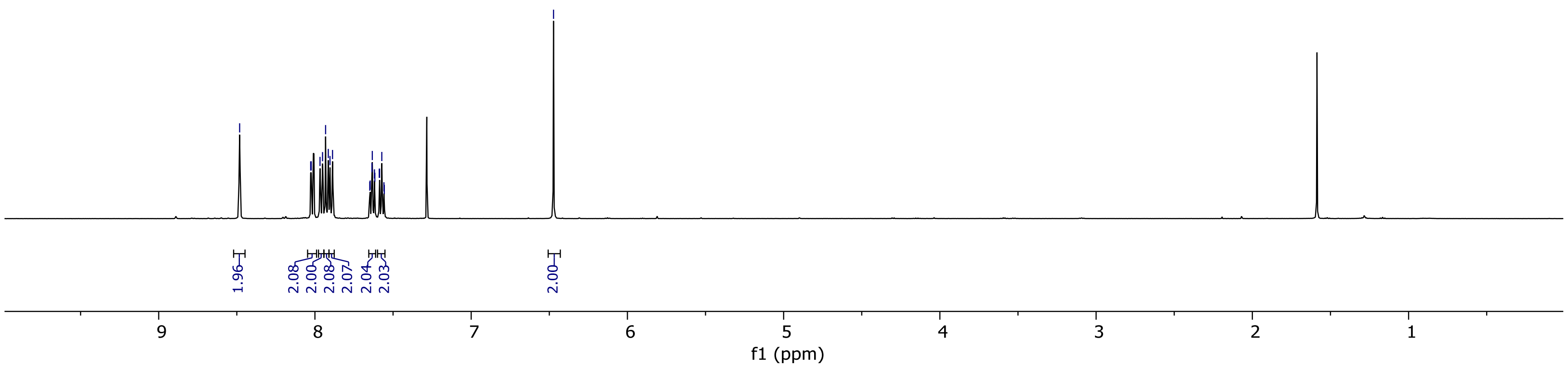

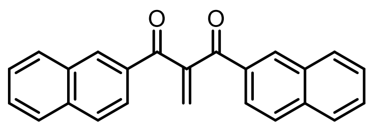


$^{13}C$ NMR, $CDCl_3$, 126 MHz

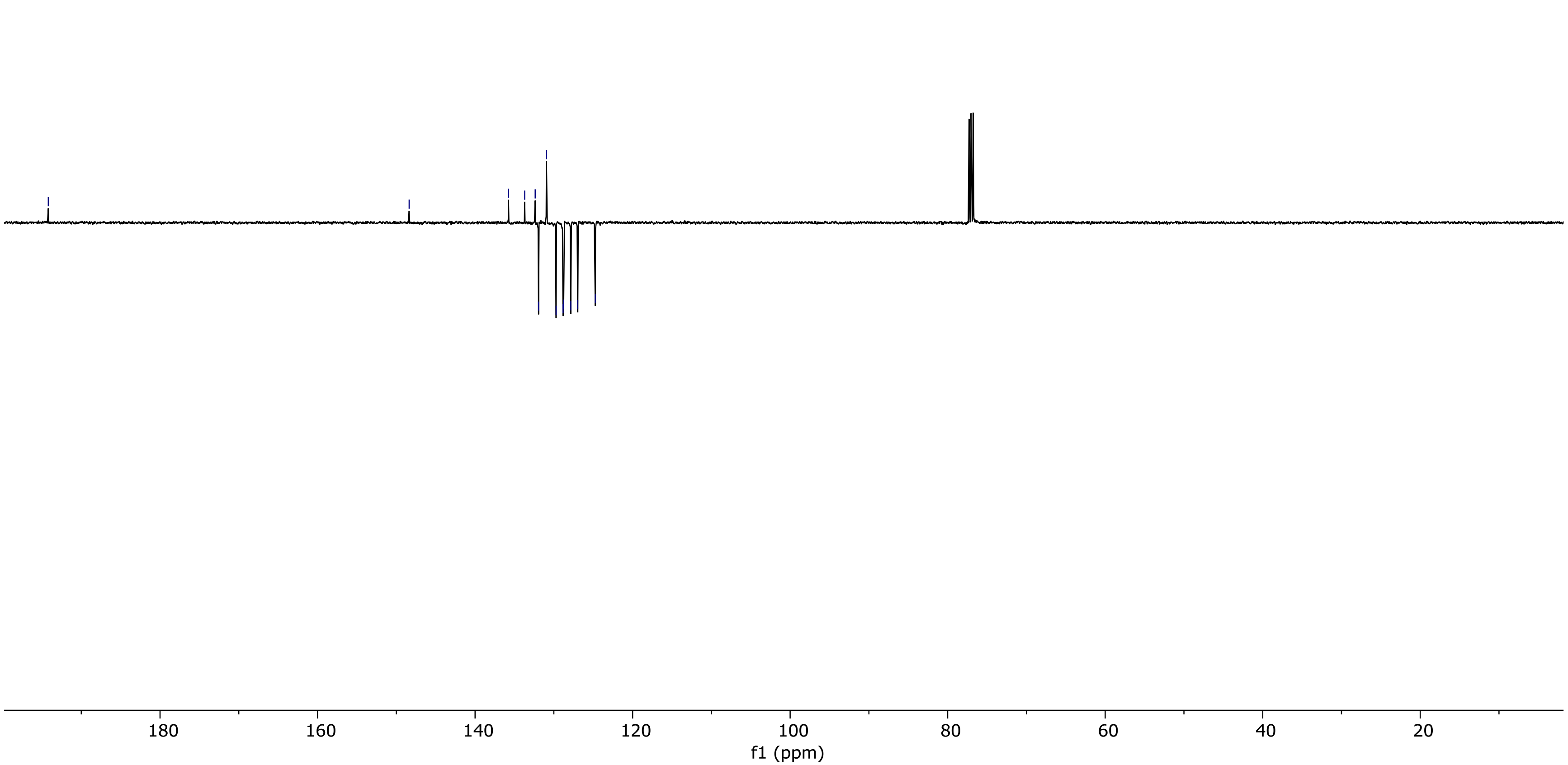

**2-Methylene-1,3-di(naphthalen-1-yl)propane-1,3-dione (S46)**

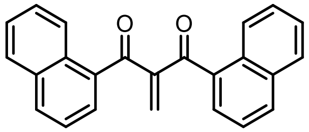


$^1$H NMR, $CDCl_3$, 500 MHz

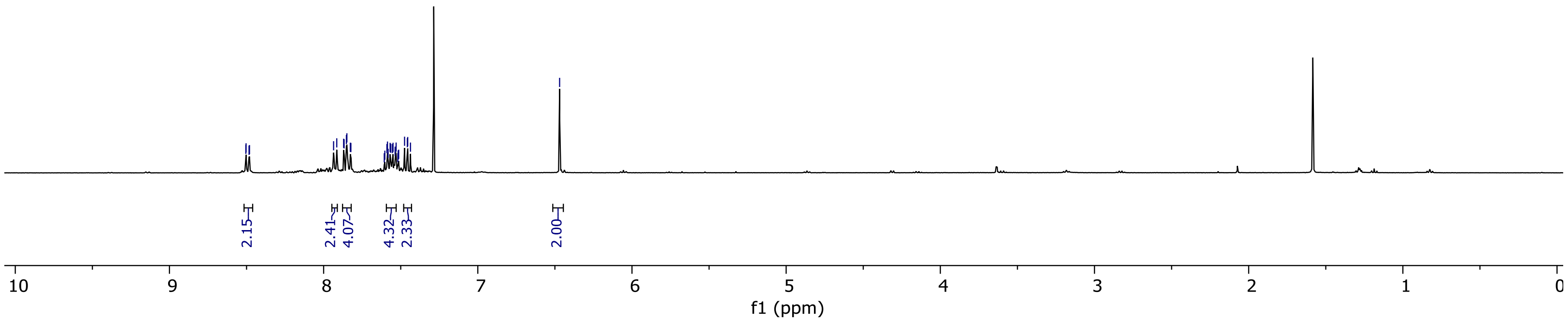

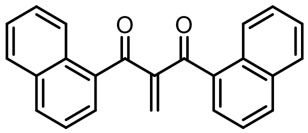

$^{13}C$ NMR, $CDCl_3$, 126 MHz

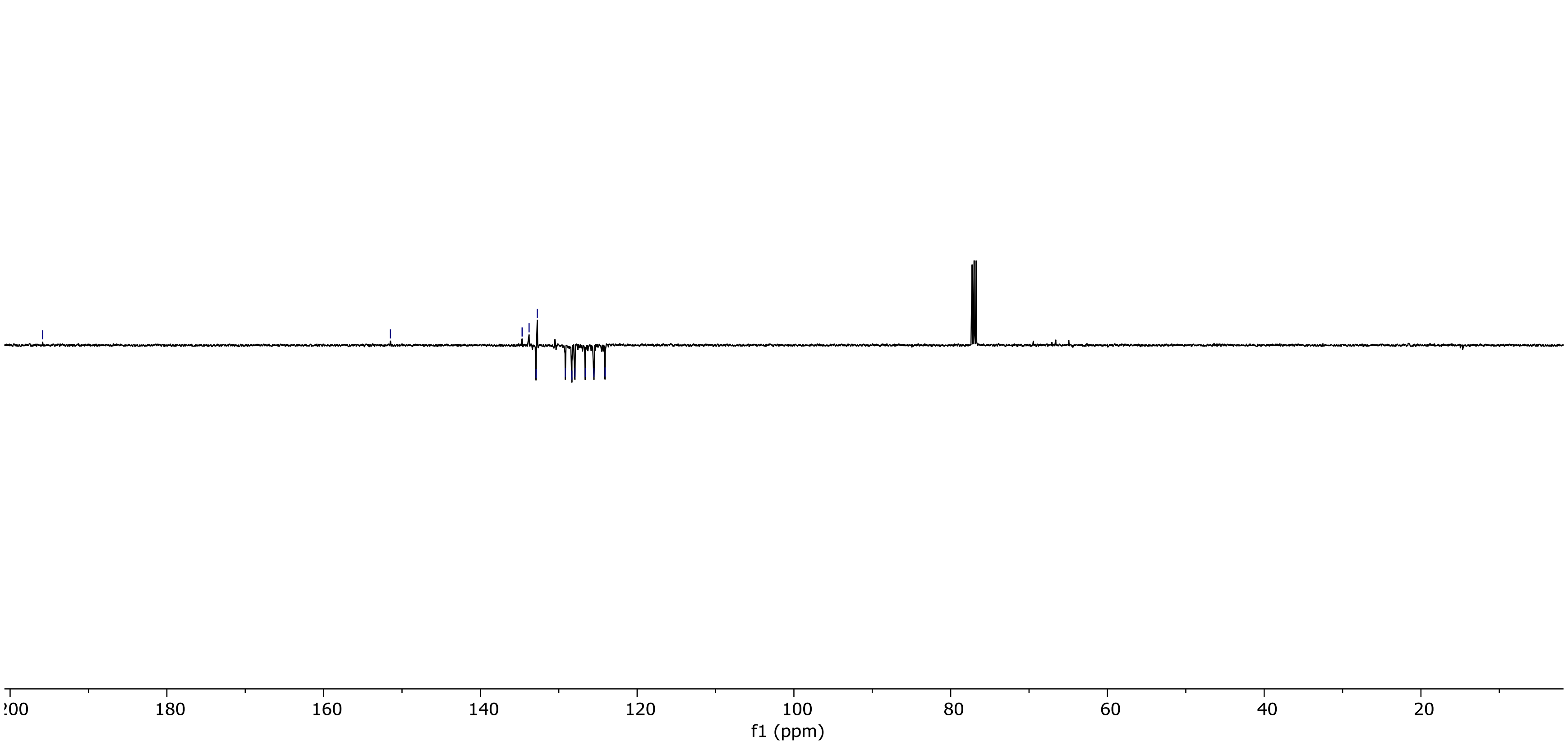

**2-Methylene-1,3-di(thiophen-2-yl)propane-1,3-dione (S47)**

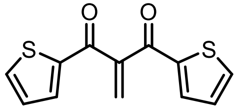


$^{1}$H NMR, $CDCl_3$, 500 MHz

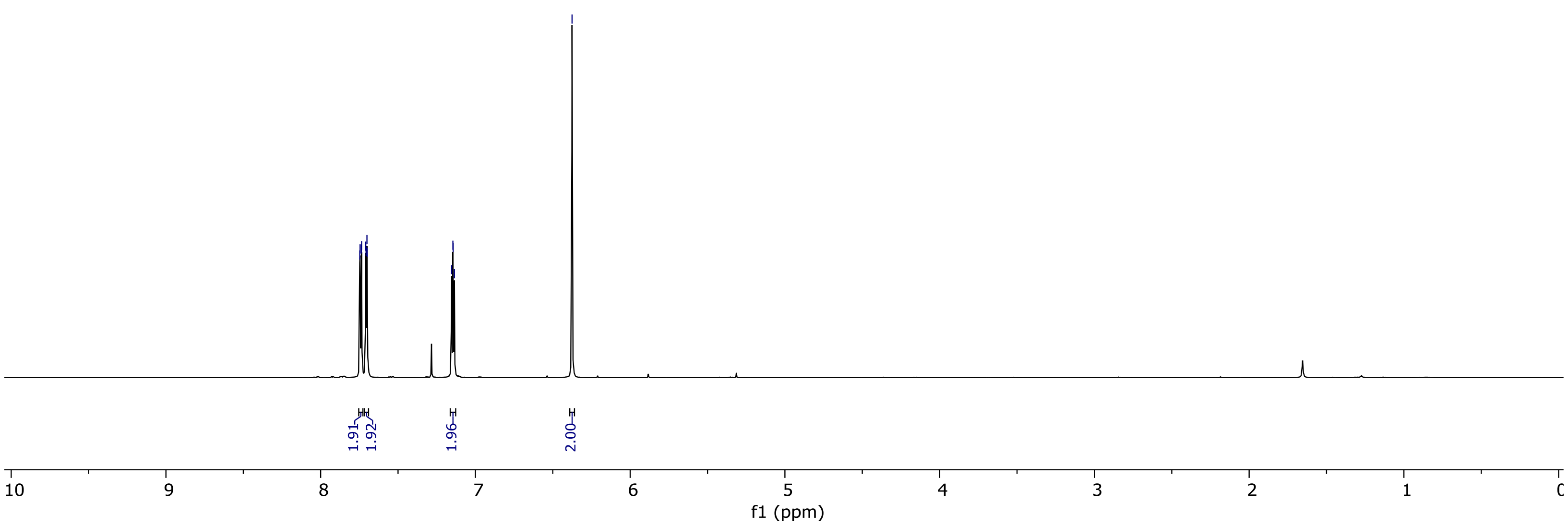

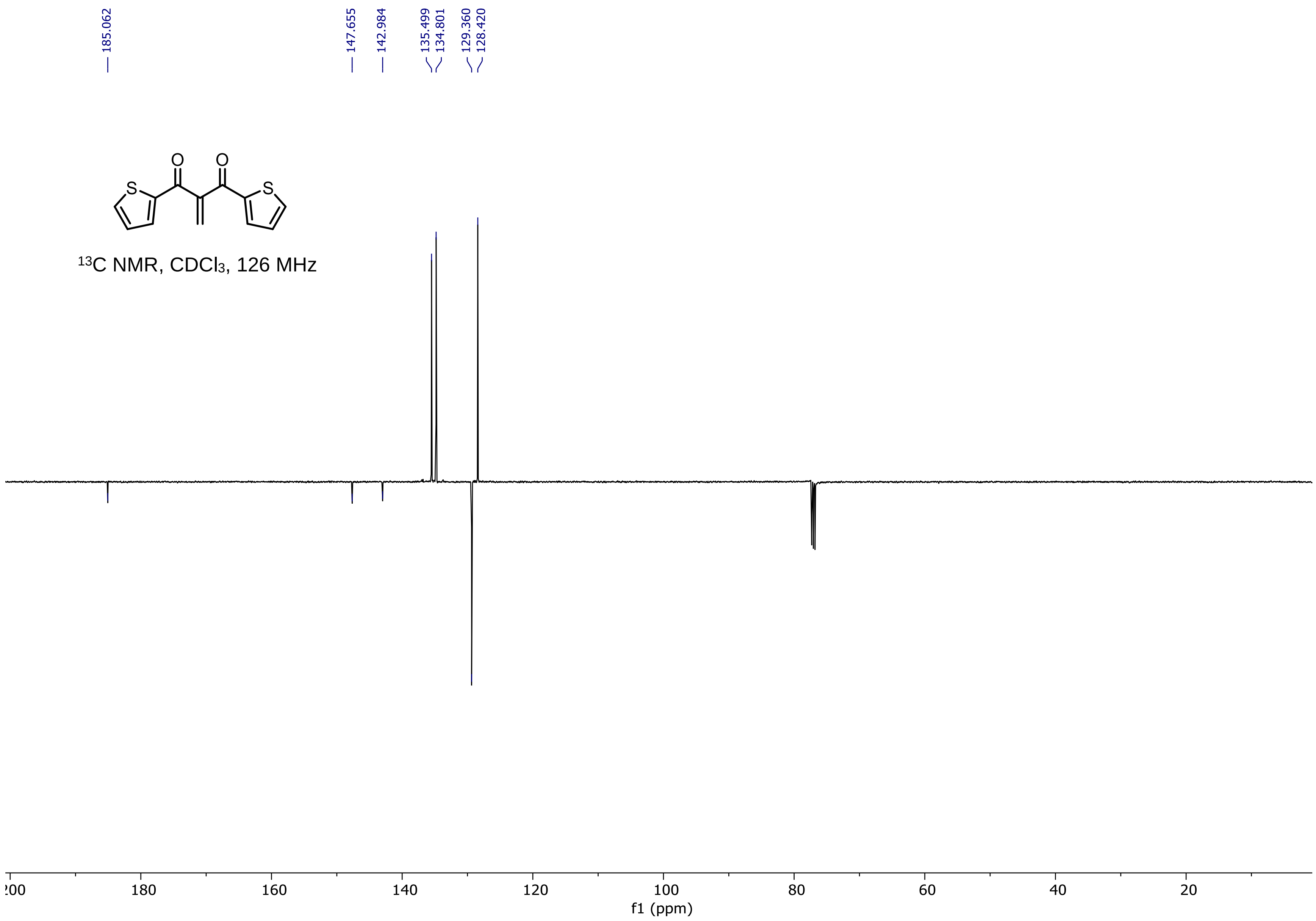
185.062
147.655
142.984
135.499
134.801
129.360
128.420
O
O
S
S
$^{13}$C NMR, CDCl$_3$, 126 MHz
200
180
160
140
120
100
80
60
40
20
f1 (ppm)

**2-Methylene-1,3-di(thiophen-3-yl)propane-1,3-dione (S48)**

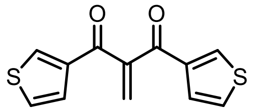


$^1H$ NMR, $CDCl_3$, 500 MHz

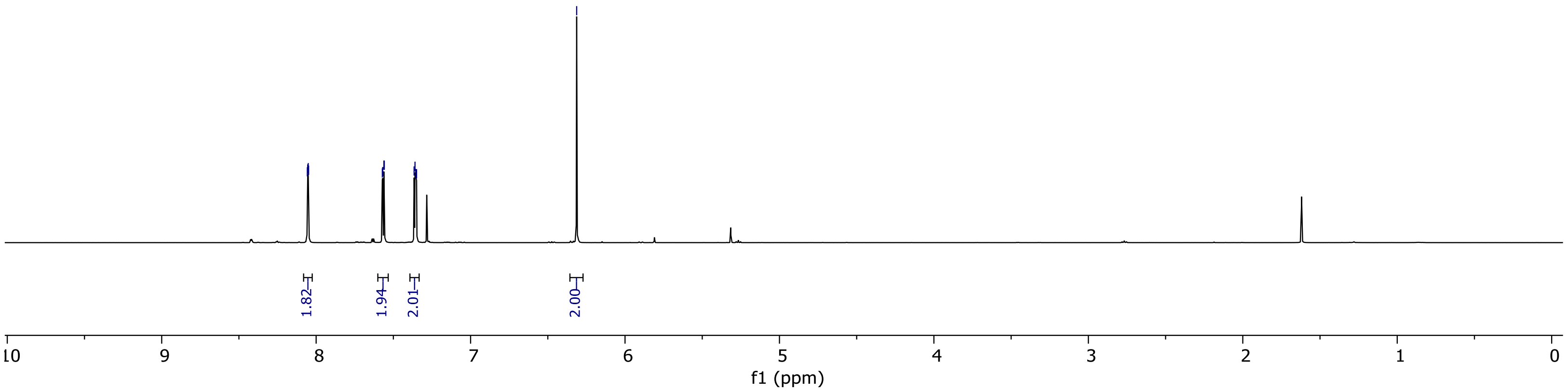

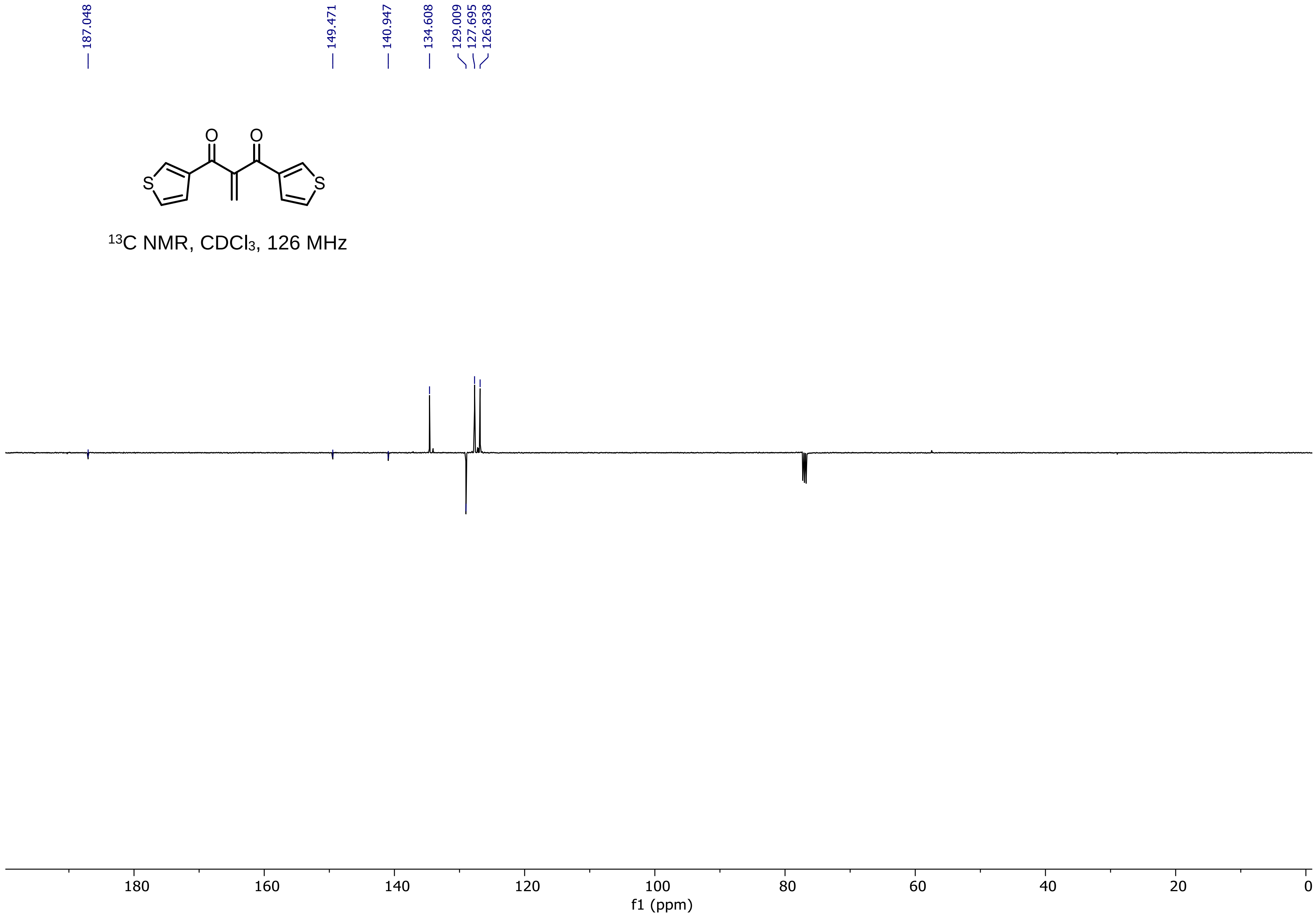
187.048
149.471
140.947
134.608
129.009
127.695
126.838
O
O
S
S
$^{13}$C NMR, $CDCl_3$, 126 MHz
180
160
140
120
100
80
60
40
20
0
f1 (ppm)

**1,3-Di(furan-2-yl)-2-methylenepropane-1,3-dione (S49)**

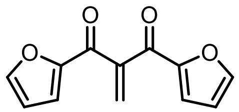

$^1H$ NMR, $CDCl_3$, 500 MHz

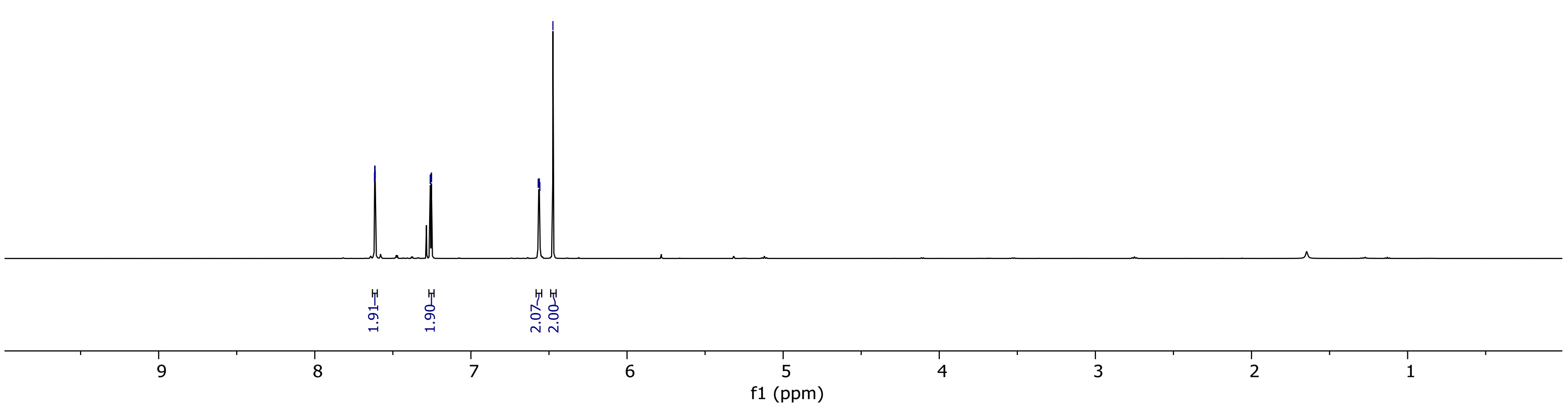

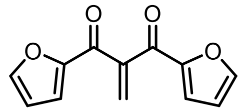


$^{13}C$ NMR, $CDCl_3$, 126 MHz

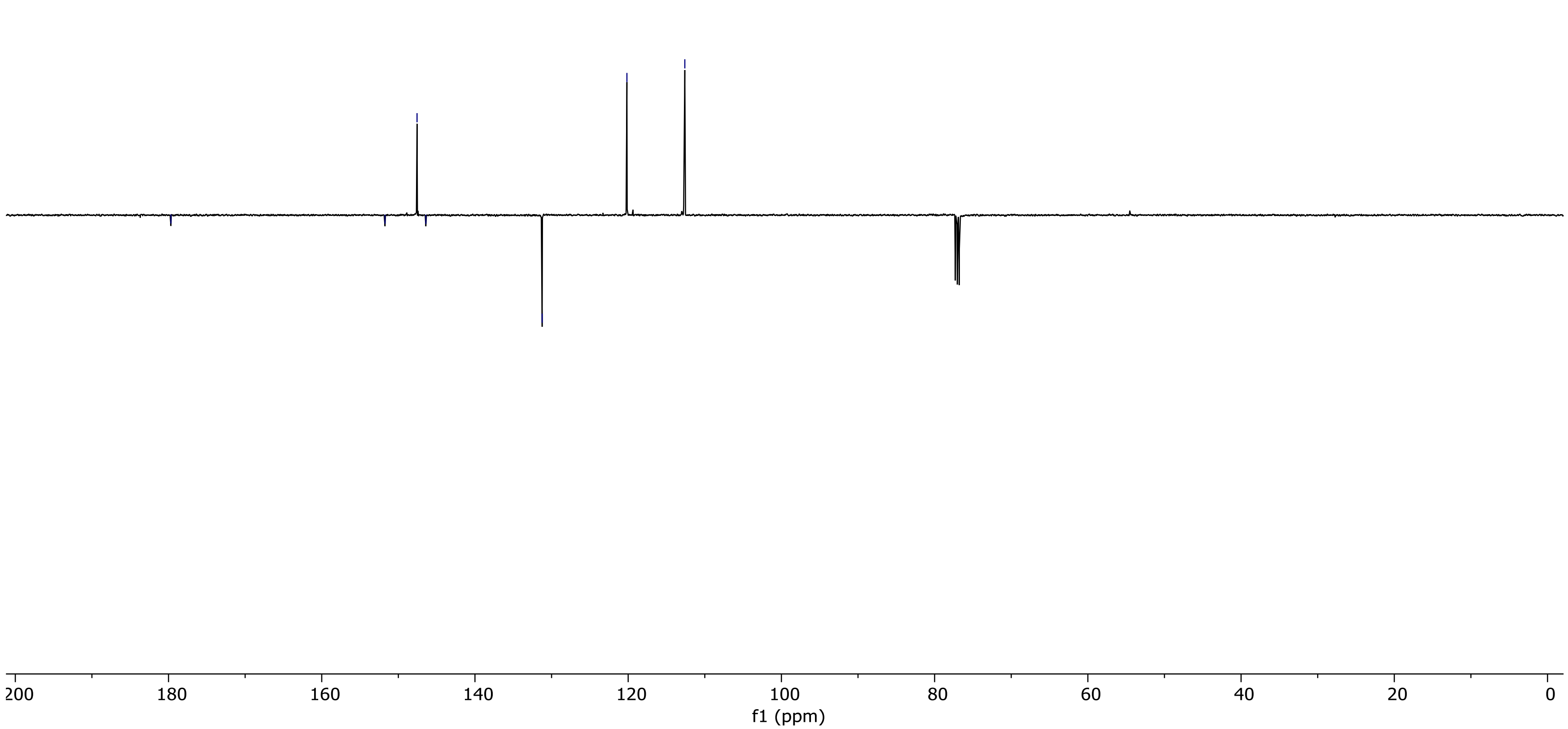

**1,3-Bis(benzo[d][1,3]dioxol-5-yl)-2-methylenepropane-1,3-dione (S50)**

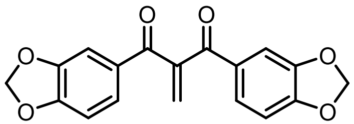

$^{1}$H NMR, $CDCl_3$, 500 MHz

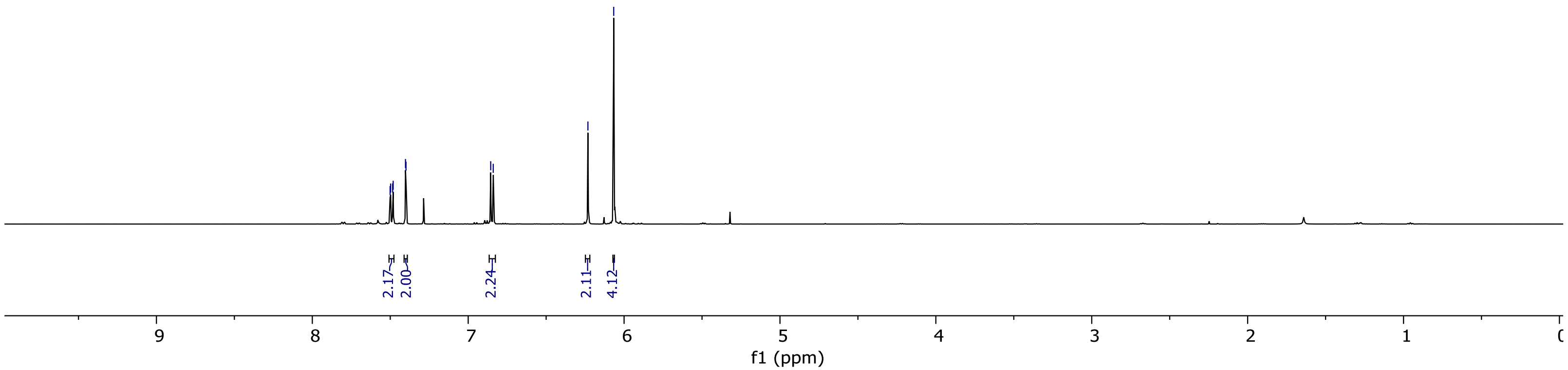

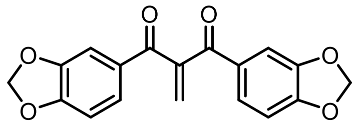

$^{13}C$ NMR, $CDCl_3$, 126 MHz

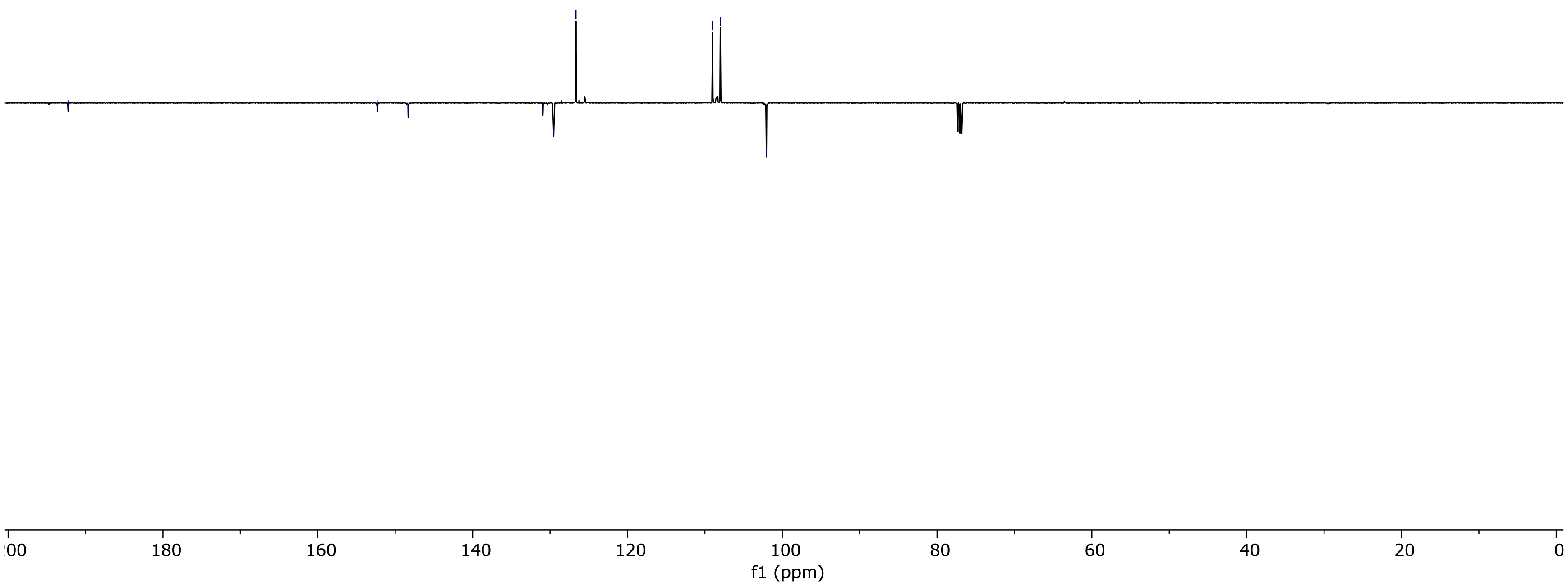

**(*S*)-*N*-Benzyl-2-((diphenylmethylene)amino)-4,4-bis(phenylsulfonyl)butanamide (17)**

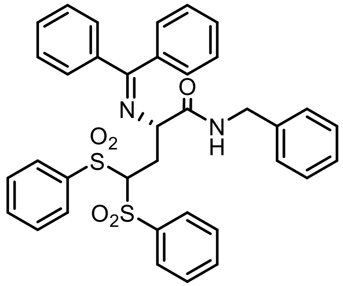

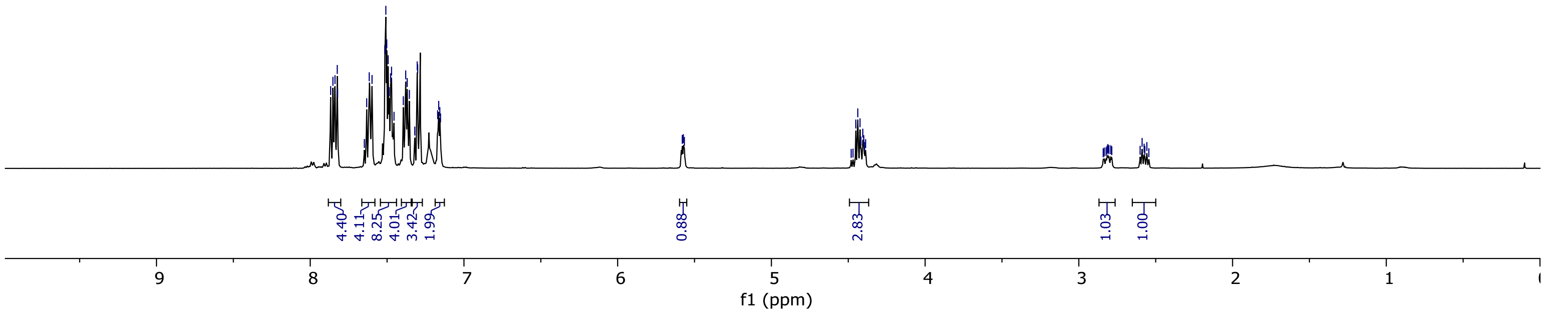


$^1$H NMR, $CD_2Cl_2$, 400 MHz

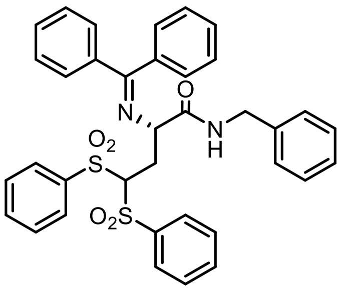

$^{13}C$ NMR, $CD_2Cl_2$, 126 MHz

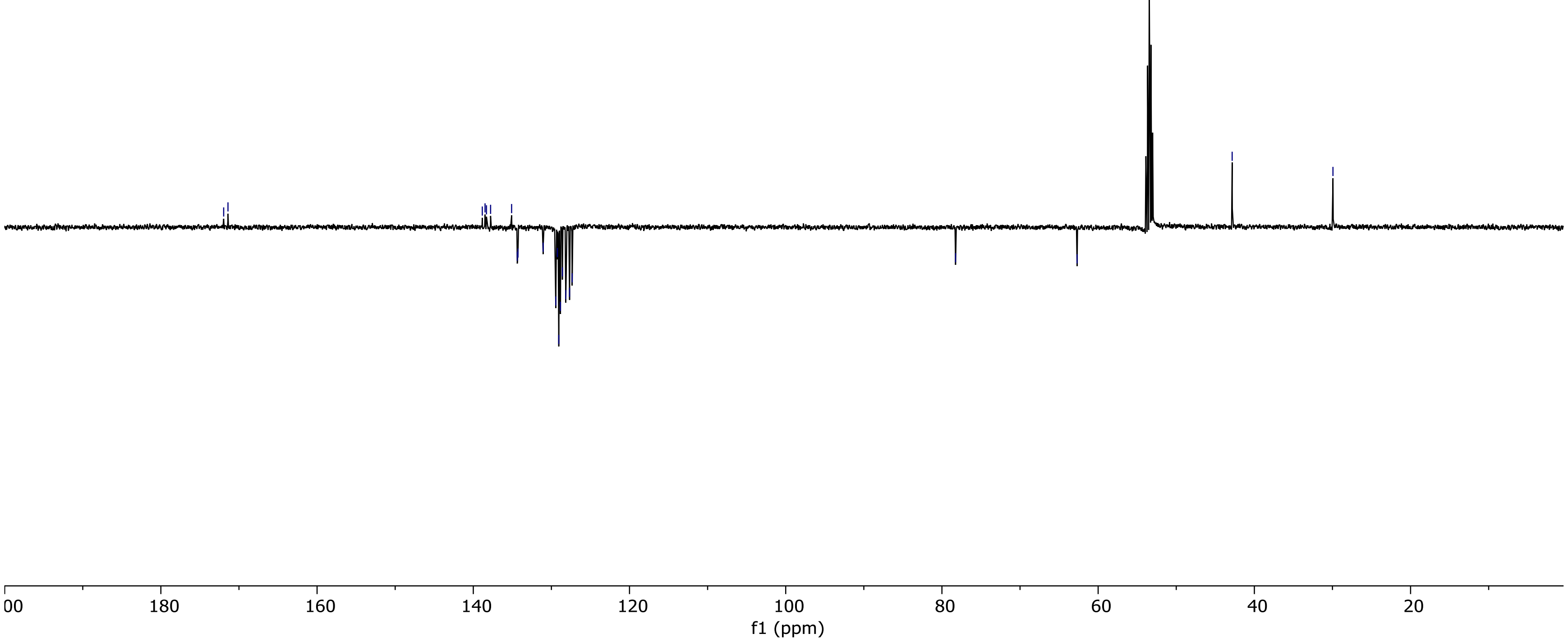

HPLC data for **17**: Chiralcel OD-H (90:10 hexane:IPA, flow rate 2 mL/min, 254 nm, 30 °C) $t_R$: 37.2 min, $t_R$: 45.8 min, 7:93 er

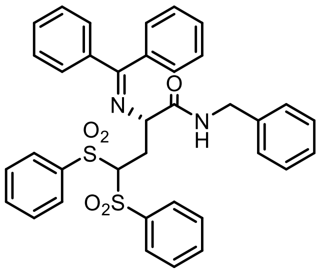

Racemic sample (left), enantioenriched sample (right)

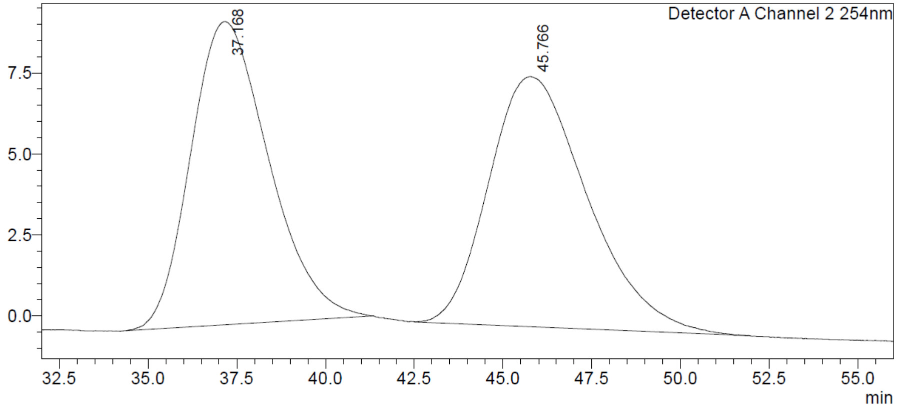


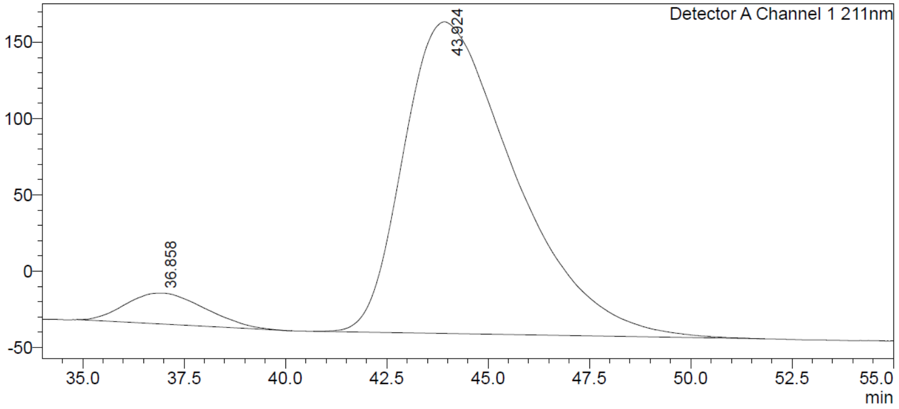


| Peak# | Ret. Time | Area% |
|---|---|---|
| 1 | 37.154 | 40.059 |
| 2 | 45.638 | 59.941 |
| Total | | 100.000 |

| Peak# | Ret. Time | Area% |
|---|---|---|
| 1 | 36.858 | 6.604 |
| 2 | 43.924 | 93.396 |
| Total | | 100.000 |

**(*S*)-2-Amino-*N*-benzyl-4,4-bis(phenylsulfonyl)butanamide (18)**

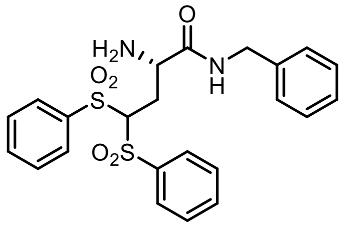


$^1H$ NMR, $CDCl_3$, 400 MHz

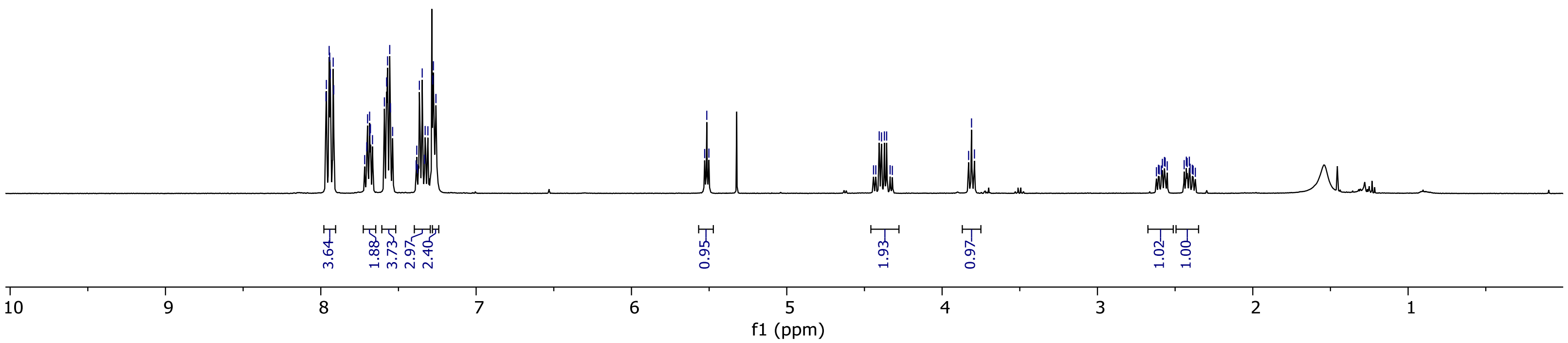

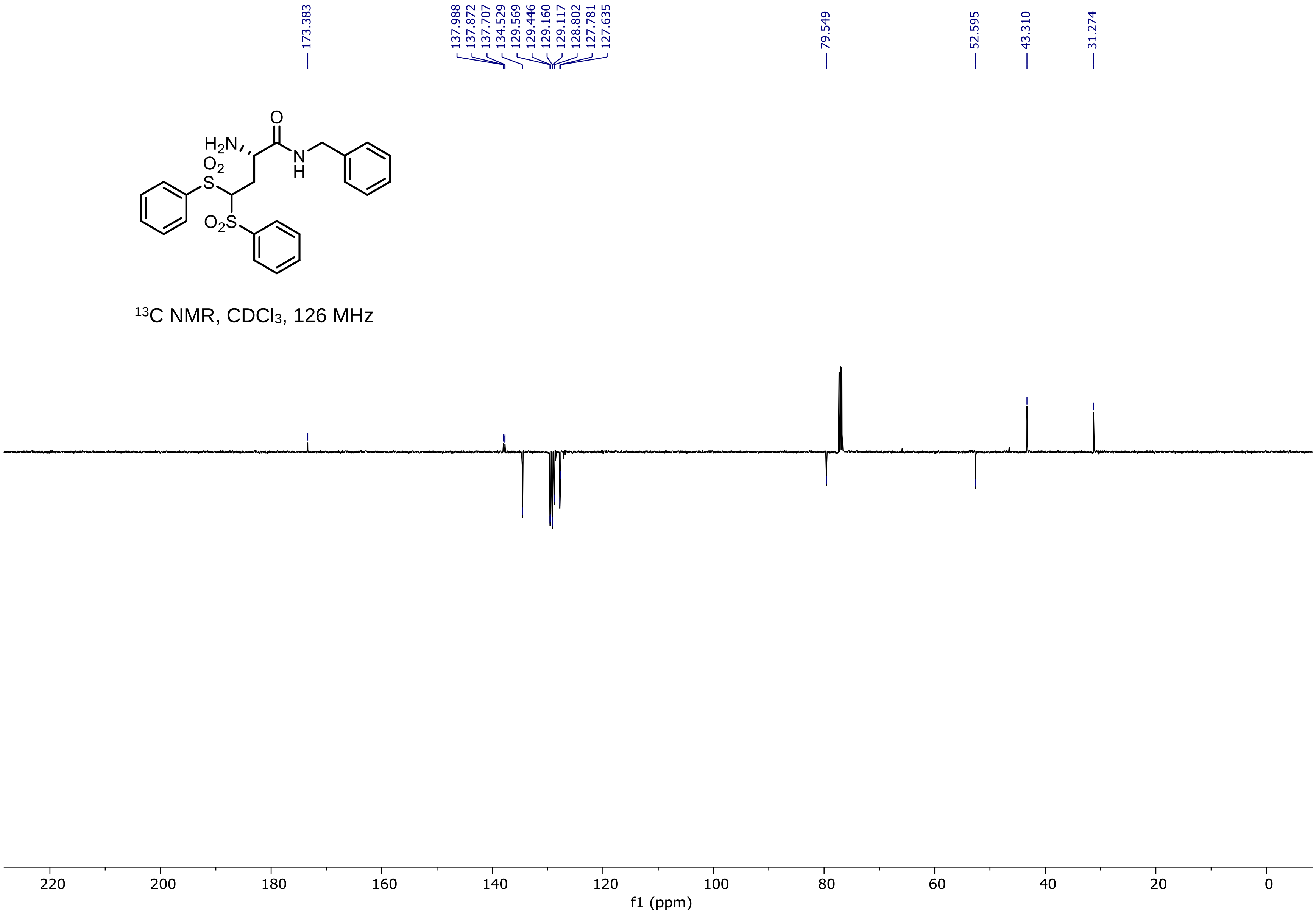
173.383
137.988
137.872
137.707
134.529
129.569
129.446
129.160
129.117
128.802
127.781
127.635
79.549
52.595
43.310
31.274
$^{13}$C NMR, $CDCl_3$, 126 MHz
220
200
180
160
140
120
100
80
60
40
20
0
f1 (ppm)

HPLC data for **18**: Chiralcel OD-H (80:20 hexane:IPA, flow rate 2 mL/min, 254 nm, 30 °C) $t_R$: 40.3 min, $t_R$: 49.5 min, 92:8 er

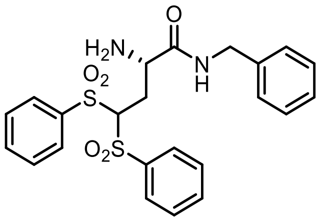


Racemic sample (left), enantioenriched sample (right)

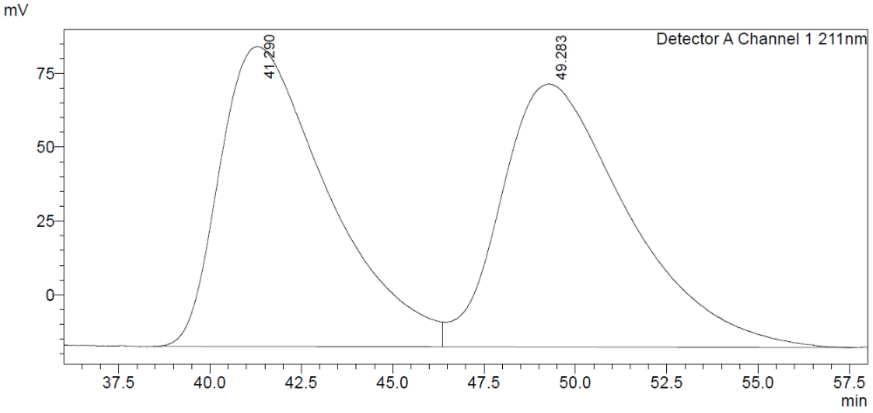


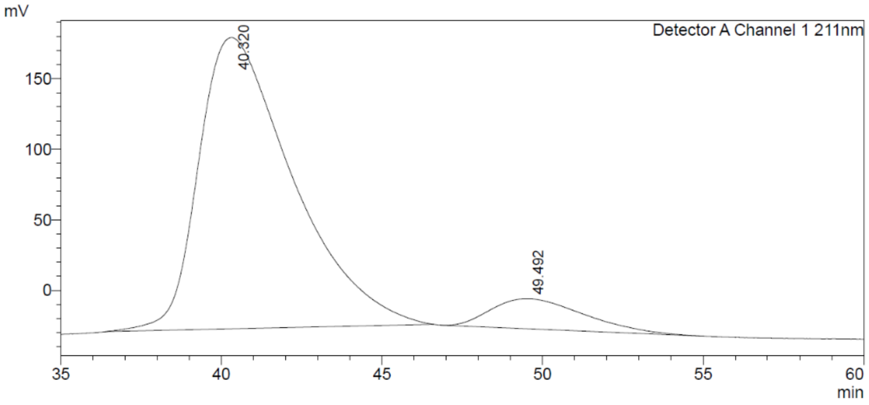


| Peak# | Ret. Time | Area% |
|---|---|---|
| 1 | 40.320 | 90.725 |
| 2 | 49.492 | 9.275 |
| Total | | 100.000 |

**(*S*)-2-((Diphenylmethylene)amino)-1-morpholino-4,4-bis(phenylsulfonyl)butan-1-one (20a)**

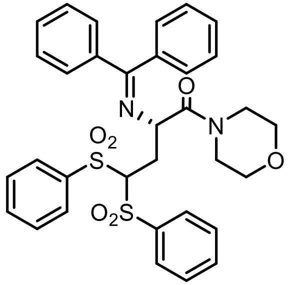


$^{1}$H NMR, $CD_2Cl_2$, 400 MHz

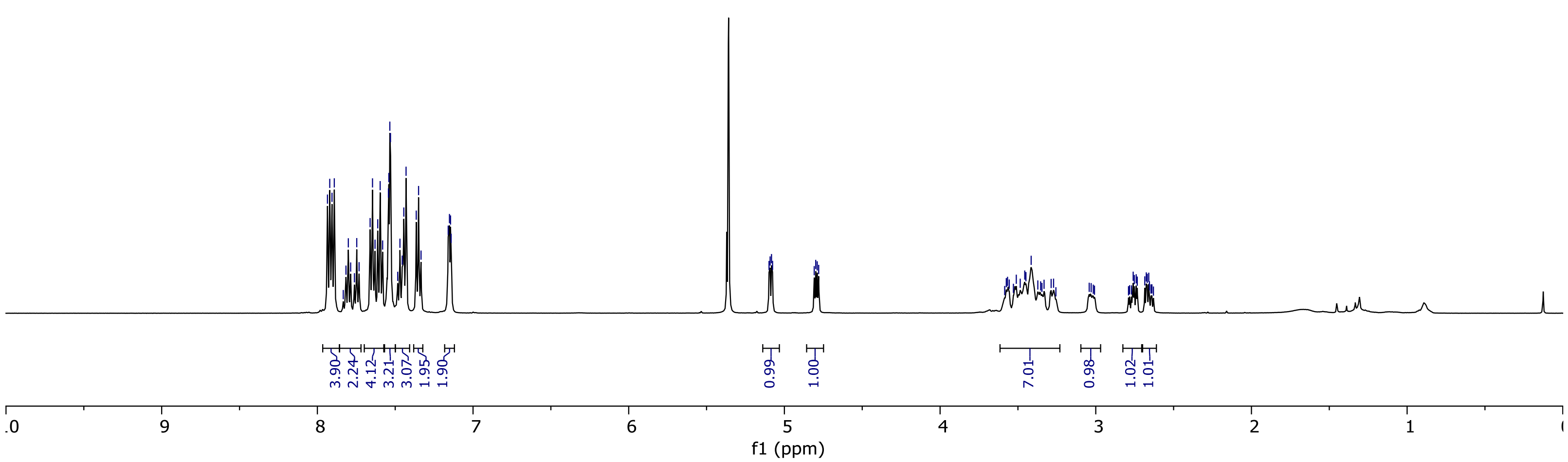

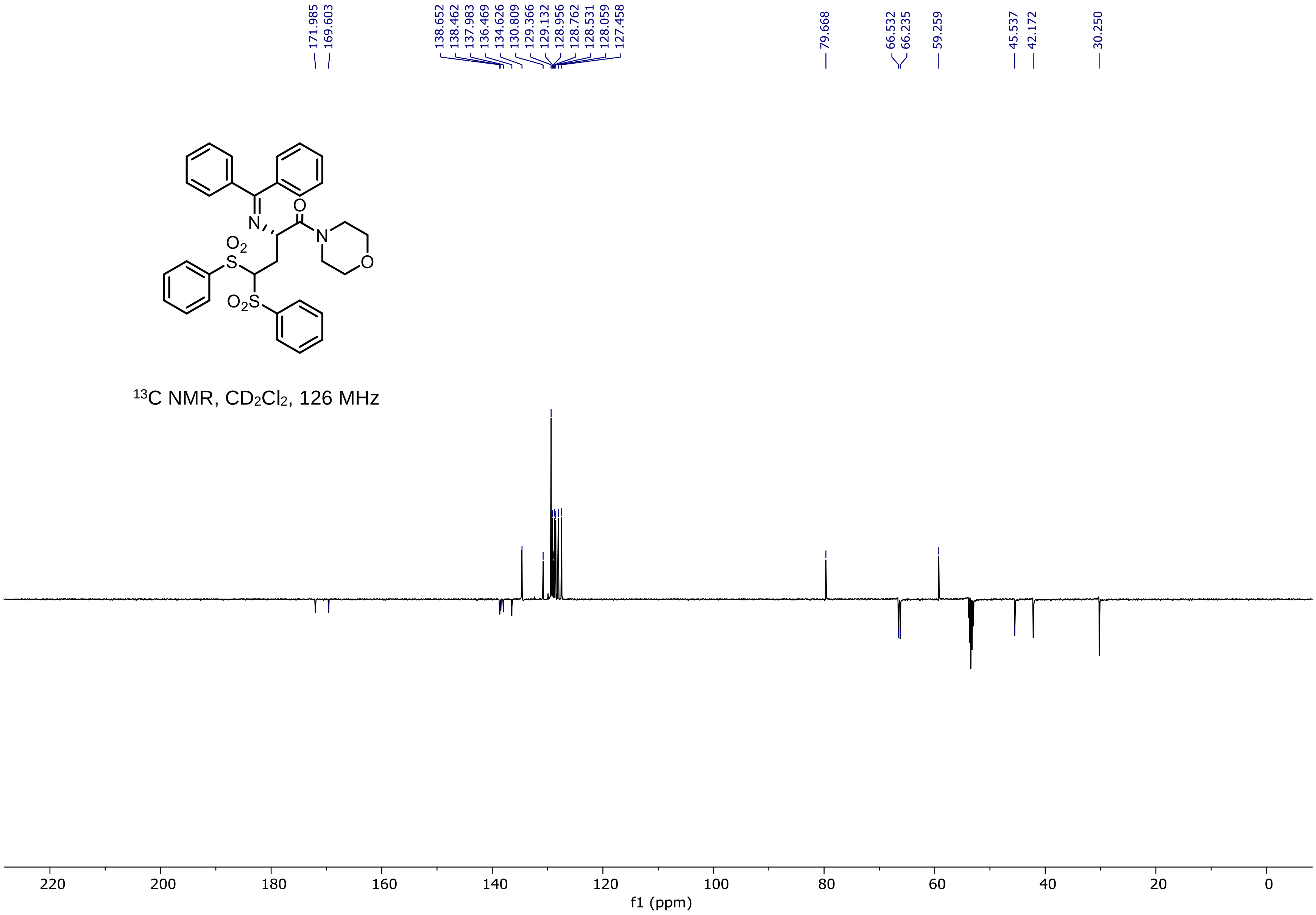


$^{13}C$ NMR, $CD_2Cl_2$, 126 MHz

HPLC data for **20a**: Chiralcel OD-H (80:20 hexane:IPA, flow rate 1 mL/min, 254 nm, 40 °C) $t_R$: 18.9 min, $t_R$: 21.3 min, 2:98 er

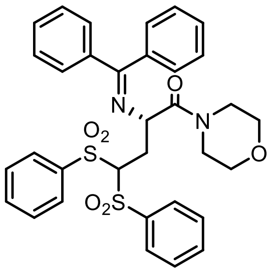


Racemic sample (left), enantioenriched sample (right)

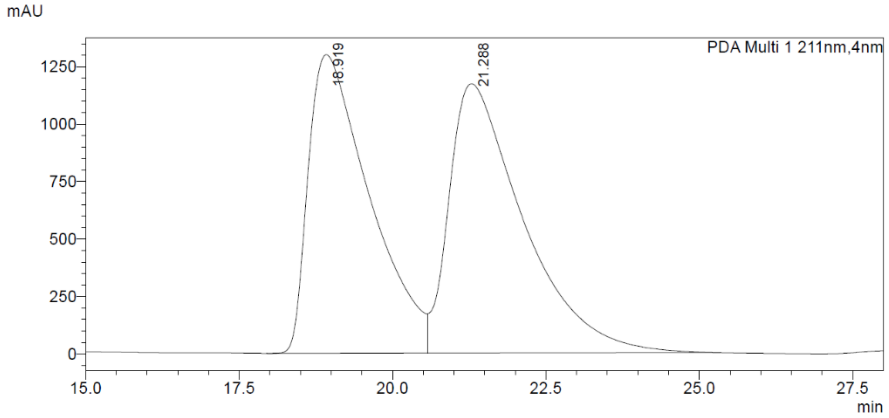


PDA Ch1 211nm

| Peak# | Ret. Time | Area% |
|---|---|---|
| 1 | 18.919 | 47.791 |
| 2 | 21.288 | 52.209 |
| Total | | 100.000 |

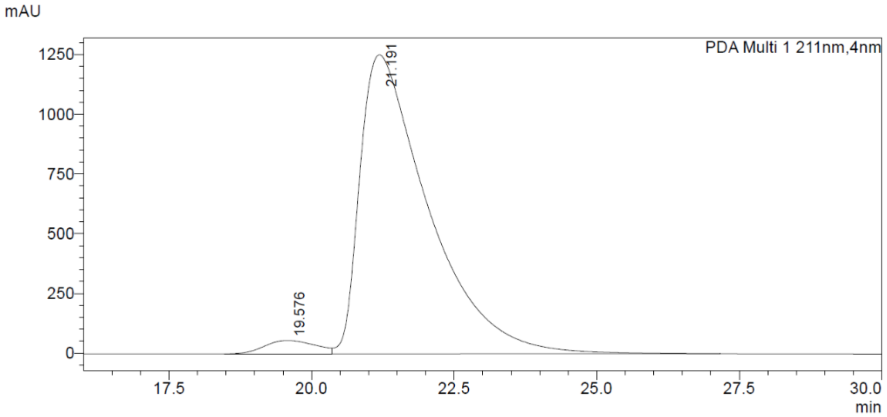


PDA Ch1 211nm

| Peak# | Ret. Time | Area% |
|---|---|---|
| 1 | 19.576 | 3.207 |
| 2 | 21.191 | 96.793 |
| Total | | 100.000 |

**(*S*)-2-((Diphenylmethylene)amino)-4,4-bis(phenylsulfonyl)-1-(pyrrolidin-1-yl)butan-1-one (20b)**

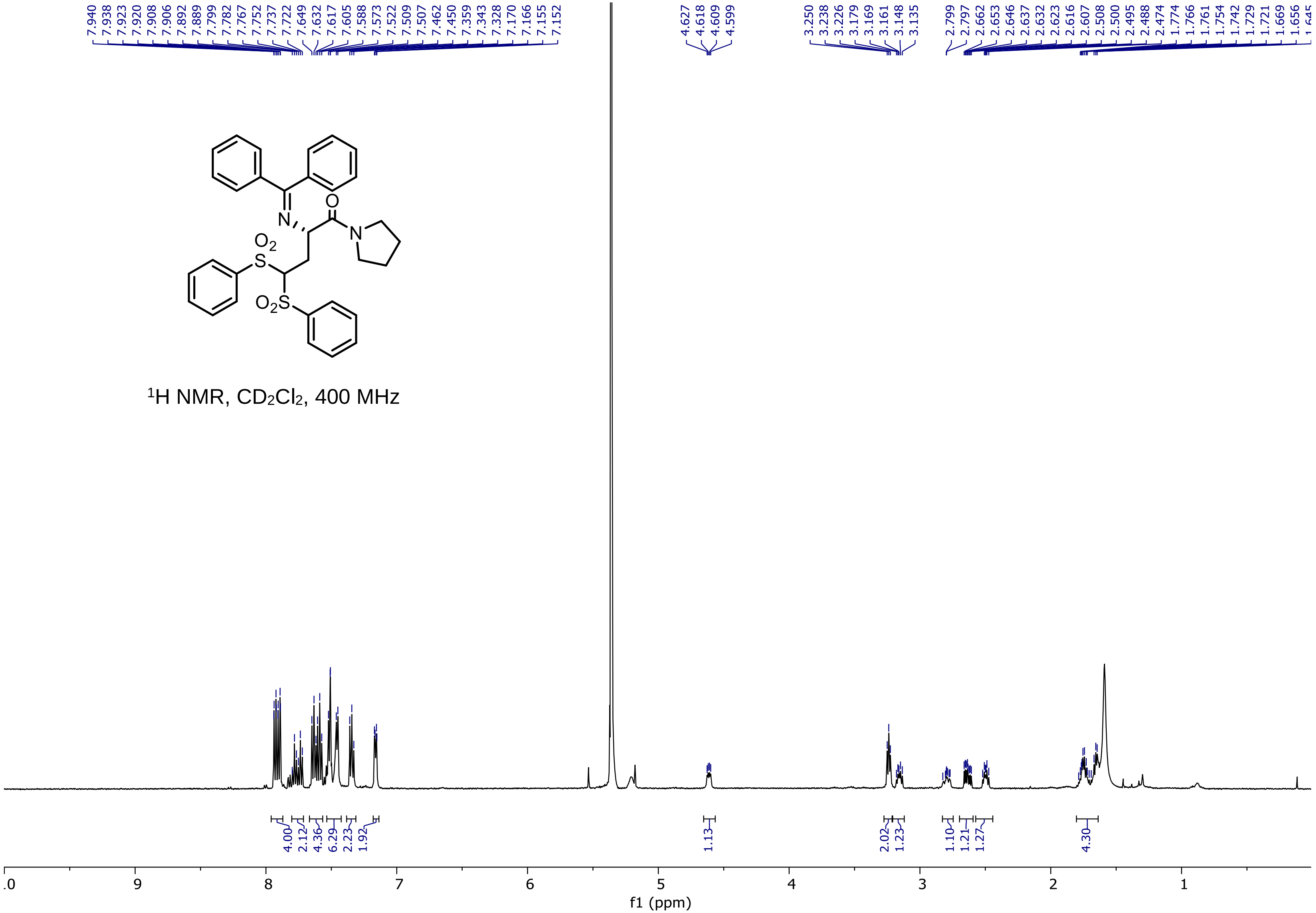

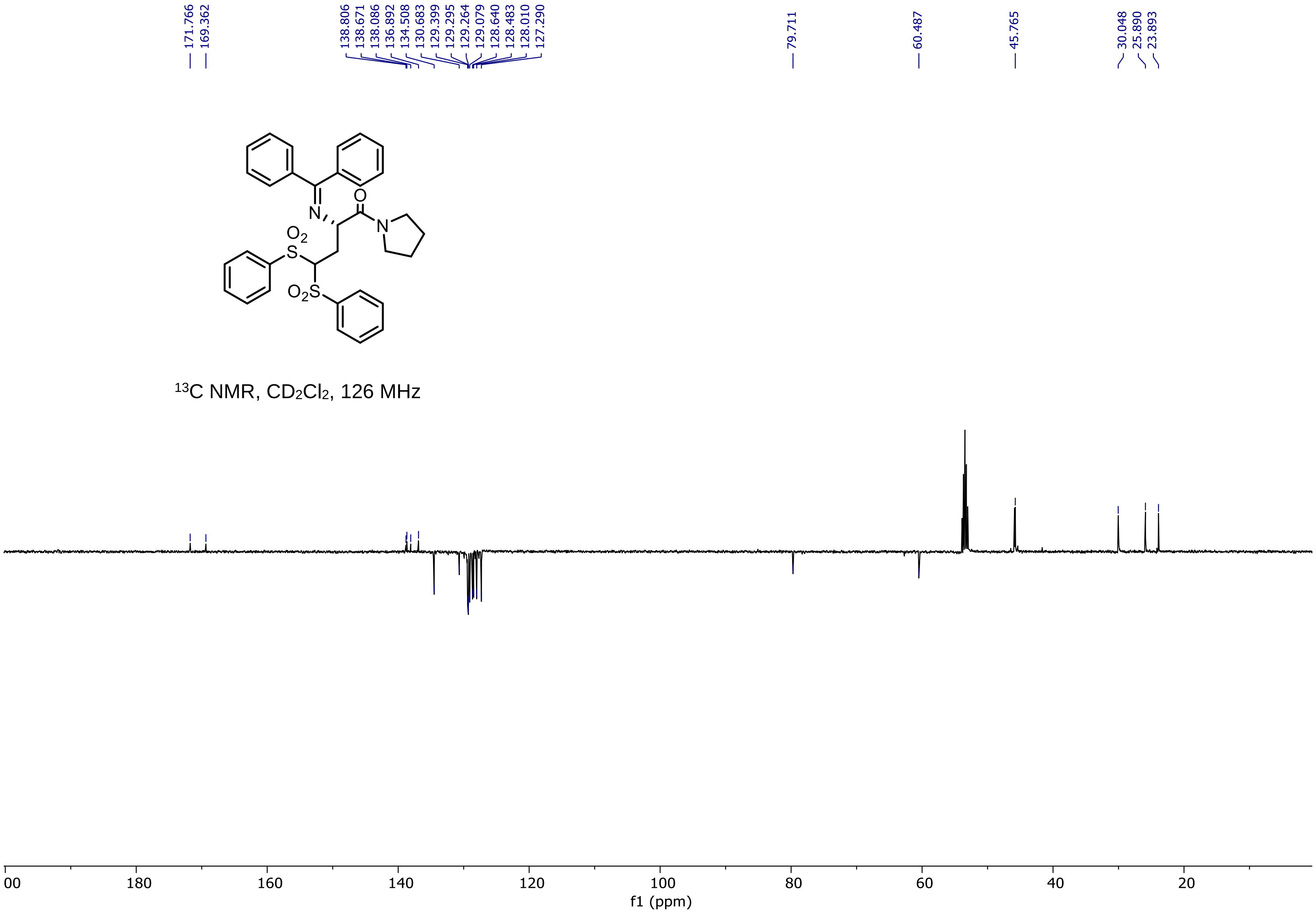


$^{13}C$ NMR, $CD_2Cl_2$, 126 MHz

HPLC data for **20b**: Chiralcel OD-H (85:15 hexane:IPA, flow rate 1 mL/min, 254 nm, 30 °C) $t_R$: 20.7 min, $t_R$: 35.1 min, 9:91 er;

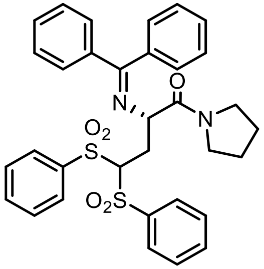


Racemic sample (left), enantioenriched sample (right)

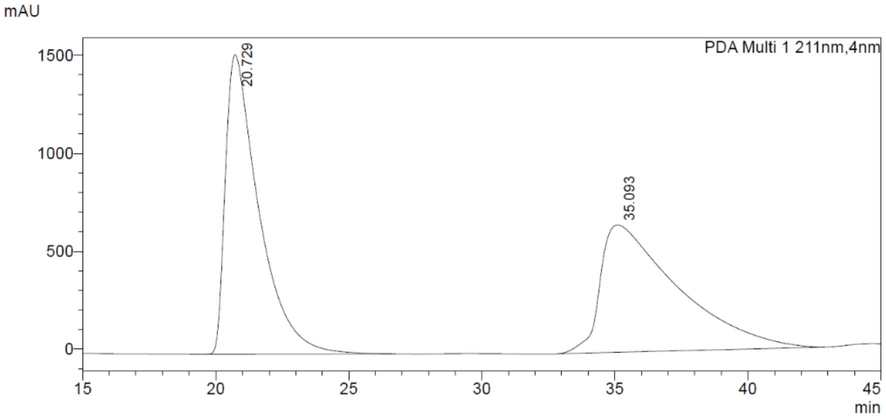


PDA Ch1 211nm

| Peak# | Ret. Time | Area% |
|---|---|---|
| 1 | 20.729 | 50.467 |
| 2 | 35.093 | 49.533 |
| Total | | 100.000 |

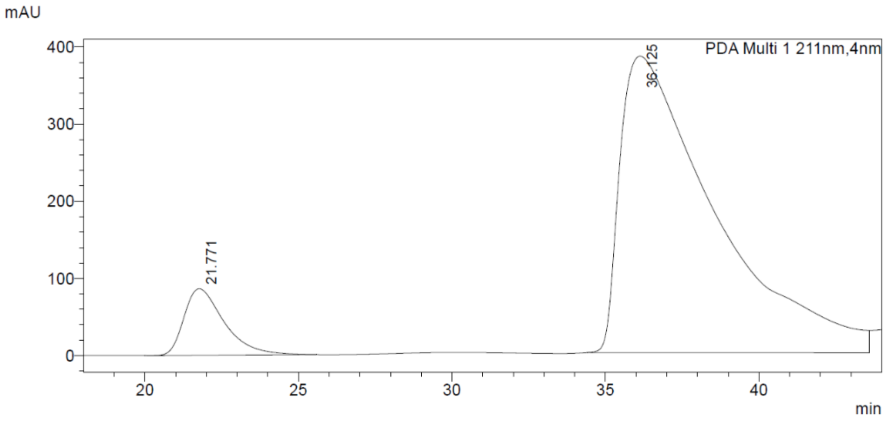


PDA Ch1 211nm

| Peak# | Ret. Time | Area% |
|---|---|---|
| 1 | 21.771 | 8.657 |
| 2 | 36.125 | 91.343 |
| Total | | 100.000 |

**tert-Butyl (S)-4-(2-((diphenylmethylene)amino)-4,4-bis(phenylsulfonyl)butanoyl)piperazine-1-carboxylate (20c)**

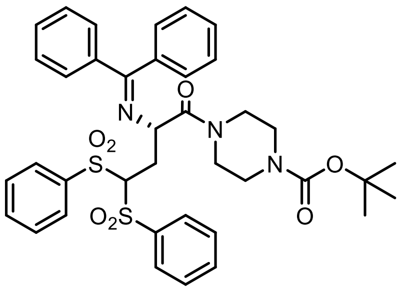

$^{1}$H NMR, $CD_2Cl_2$, 400 MHz

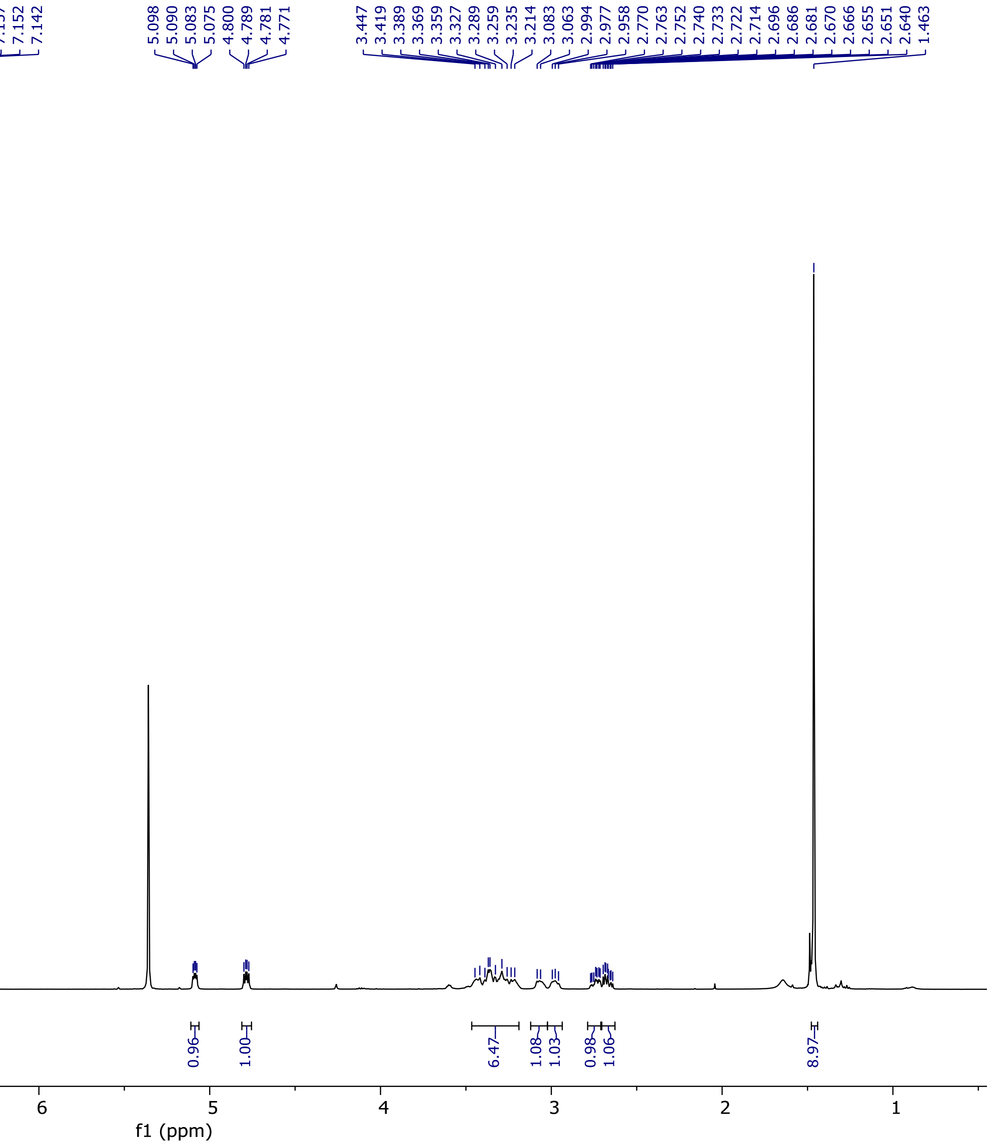

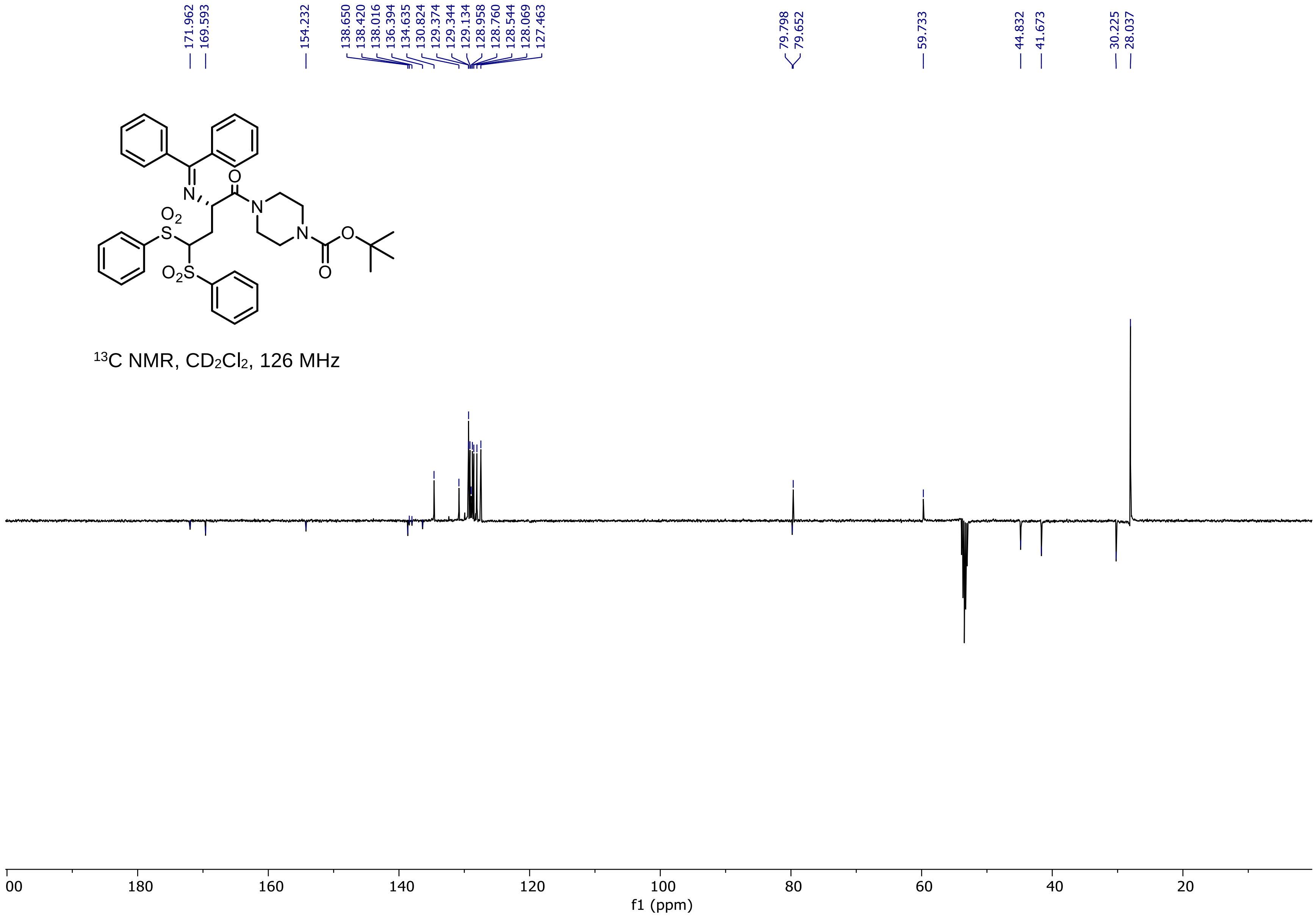
171.962
169.593
154.232
138.650
138.420
138.016
136.394
134.635
130.824
129.374
129.344
129.134
128.958
128.760
128.544
128.069
127.463
79.798
79.652
59.733
44.832
41.673
30.225
28.037
$^{13}$C NMR, $CD_2Cl_2$, 126 MHz
00
180
160
140
120
100
80
60
40
20
f1 (ppm)

HPLC data for **20c**: Chiralcel OD-H (85:15 hexane:IPA, flow rate 1 mL/min, 254 nm, 40 °C) $t_R$: 23.0 min, $t_R$: 29.1 min, 9:91 er

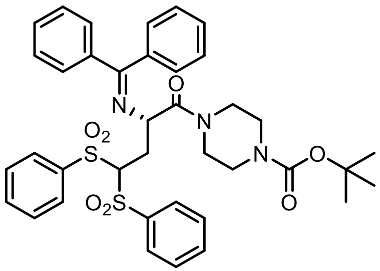

Racemic sample (left), enantioenriched sample (right)

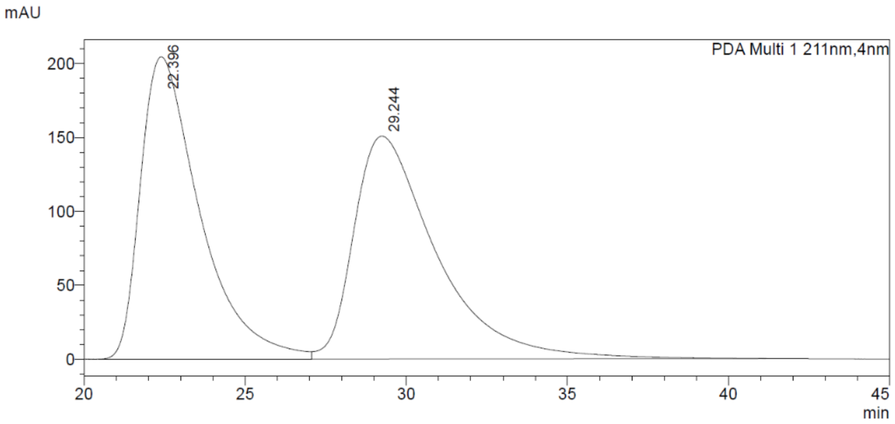


PDA Ch1 211nm

| Peak# | Ret. Time | Area% |
|---|---|---|
| 1 | 22.396 | 49.318 |
| 2 | 29.244 | 50.682 |
| Total | | 100.000 |

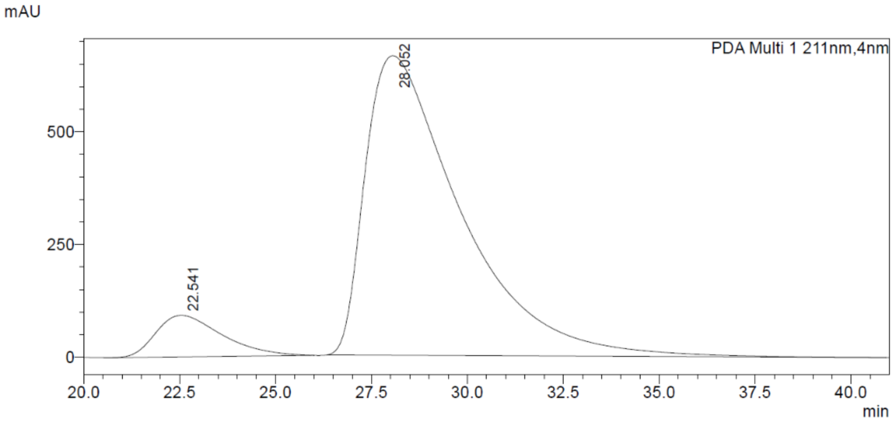


PDA Ch1 211nm

| Peak# | Ret. Time | Area% |
|---|---|---|
| 1 | 22.541 | 8.745 |
| 2 | 28.052 | 91.255 |
| Total | | 100.000 |

**Methyl (*S*)-2-((diphenylmethylene)amino)-4,4-bis(phenylsulfonyl)butanoate (20d)**

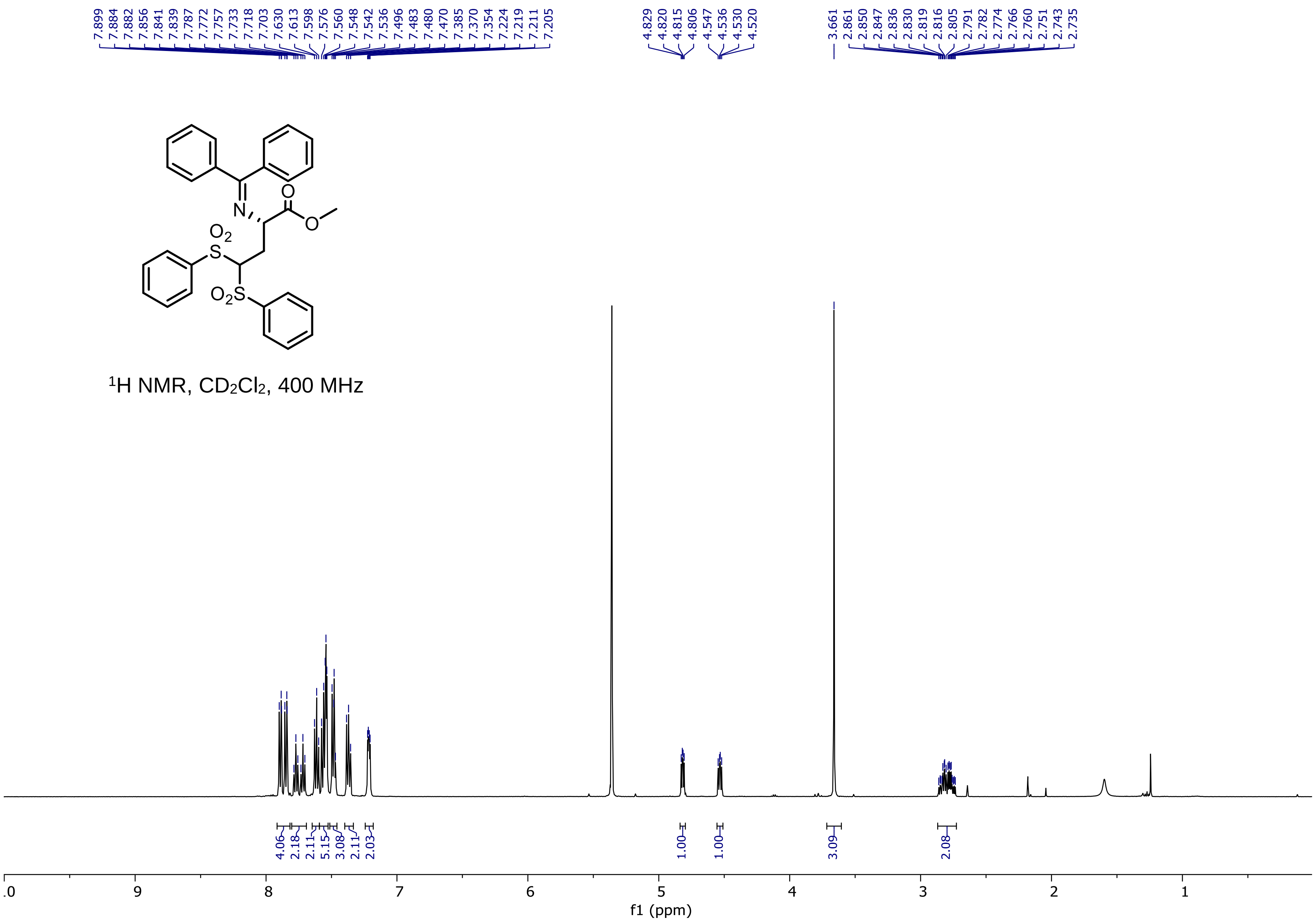

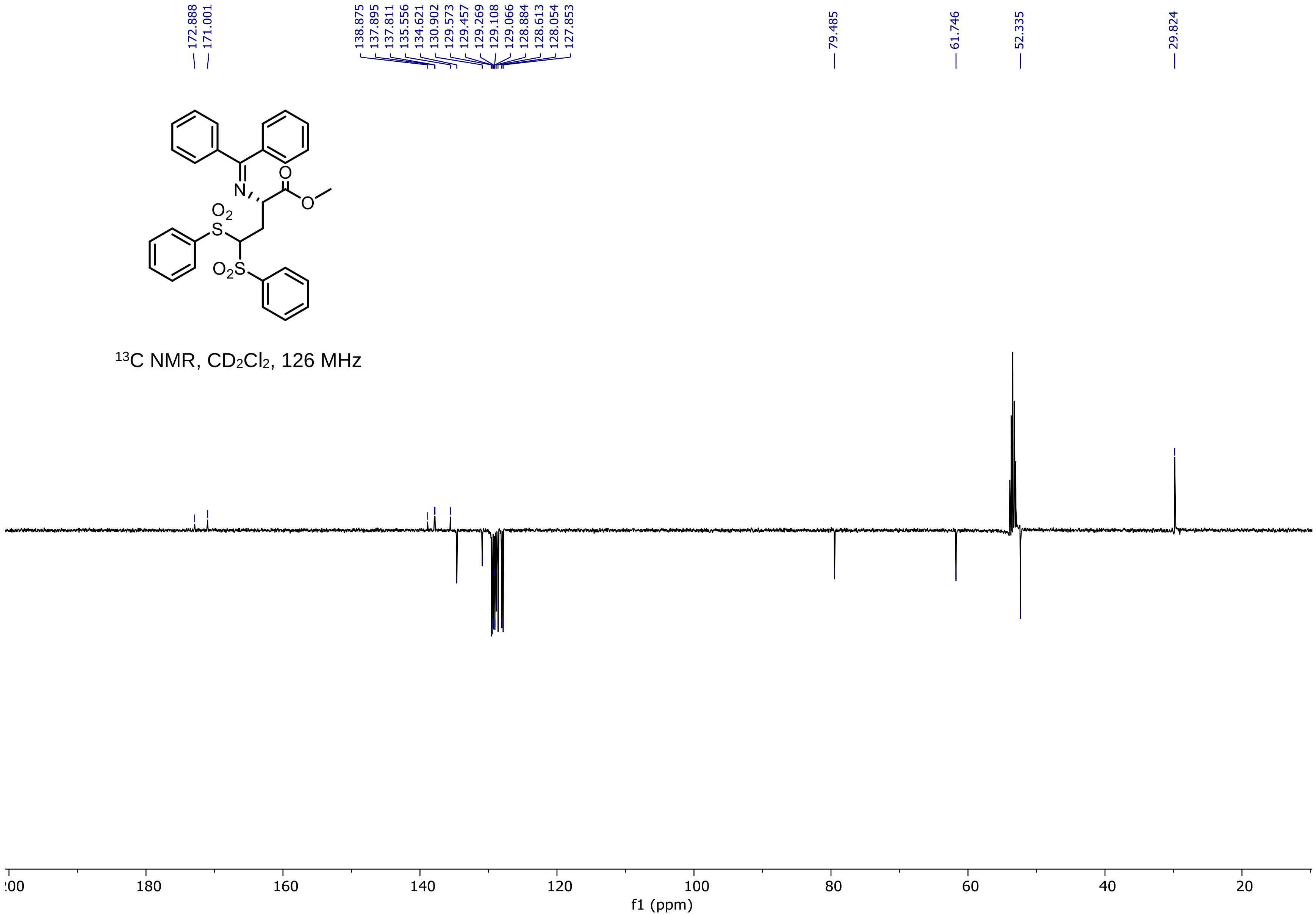
172.888
171.001
138.875
137.895
137.811
135.556
134.621
130.902
129.573
129.457
129.269
129.108
129.066
128.884
128.613
128.054
127.853
79.485
61.746
52.335
29.824
O
N
O
O2
S
O2S
$^{13}$C NMR, $CD_2Cl_2$, 126 MHz
180
160
140
120
100
80
60
40
20
f1 (ppm)

HPLC data for **20d**: Chiralpak IB (95:5 hexane:IPA, flow rate 1 mL/min, 254 nm, 30 °C) $t_R$: 32.5 min, $t_R$: 37.4 min, 9:91 er

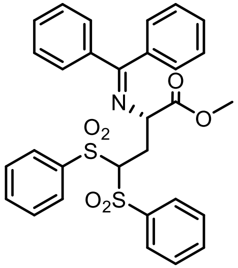


Racemic sample (left), enantioenriched sample (right)

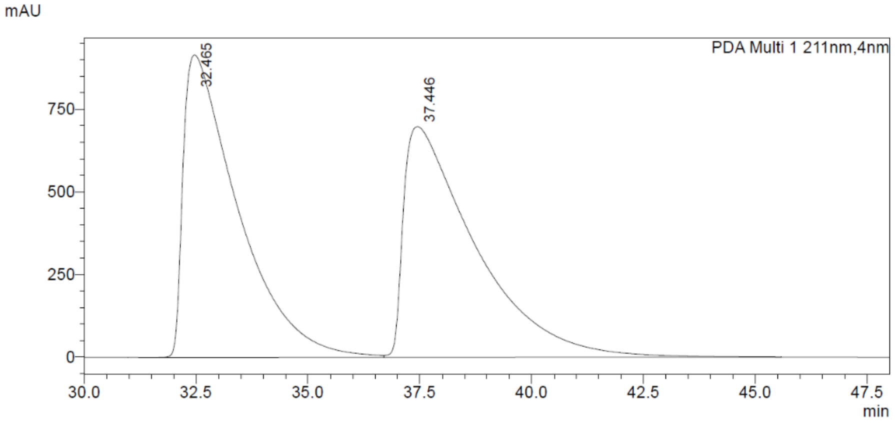


PDA Ch1 211nm

| Peak# | Ret. Time | Area% |
|---|---|---|
| 1 | 32.465 | 50.009 |
| 2 | 37.446 | 49.991 |
| Total | | 100.000 |

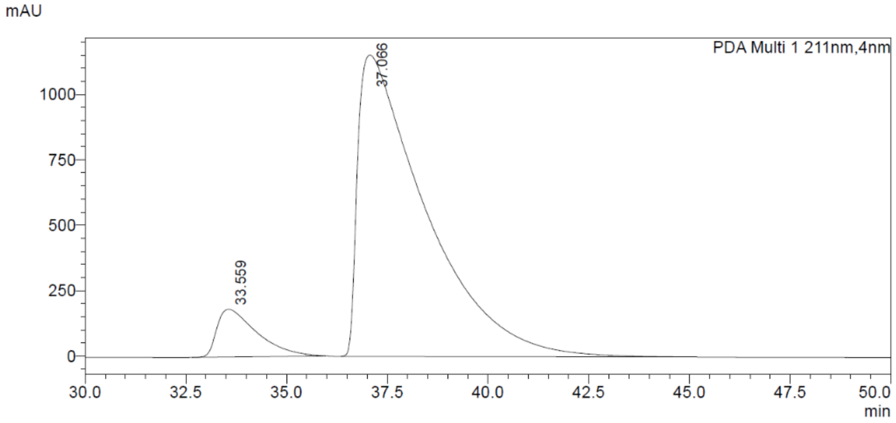


PDA Ch1 211nm

| Peak# | Ret. Time | Area% |
|---|---|---|
| 1 | 33.559 | 8.521 |
| 2 | 37.066 | 91.479 |
| Total | | 100.000 |

**(*S*)-2-Benzoyl-4-((diphenylmethylene)amino)-5-morpholino-1-phenylpentane-1,5-dione (20e)**

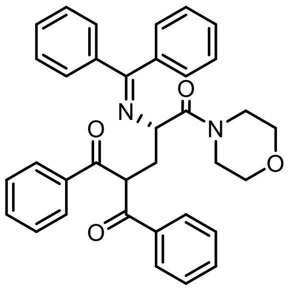

$^1$H NMR, $CD_2Cl_2$, 400 MHz

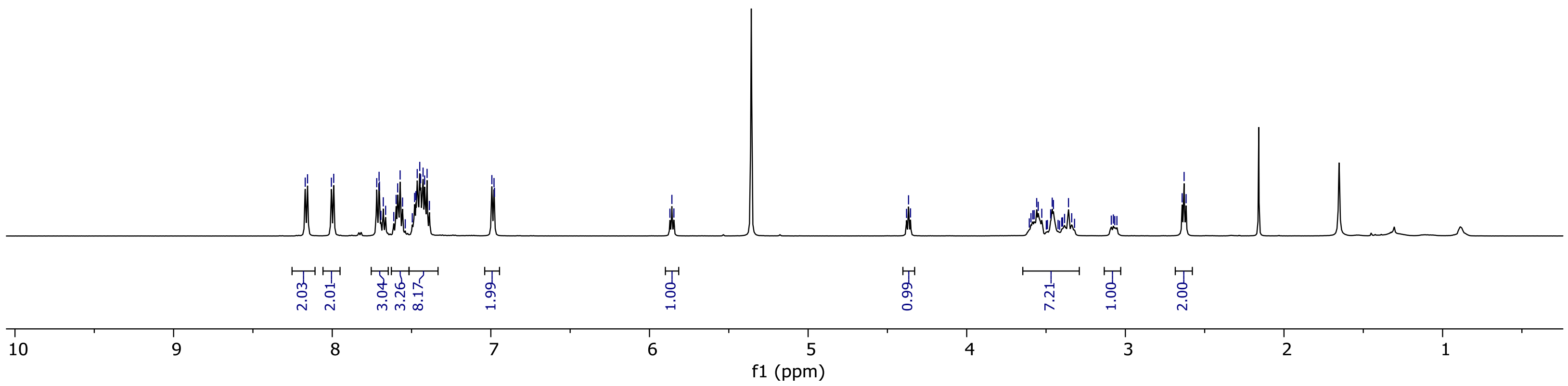

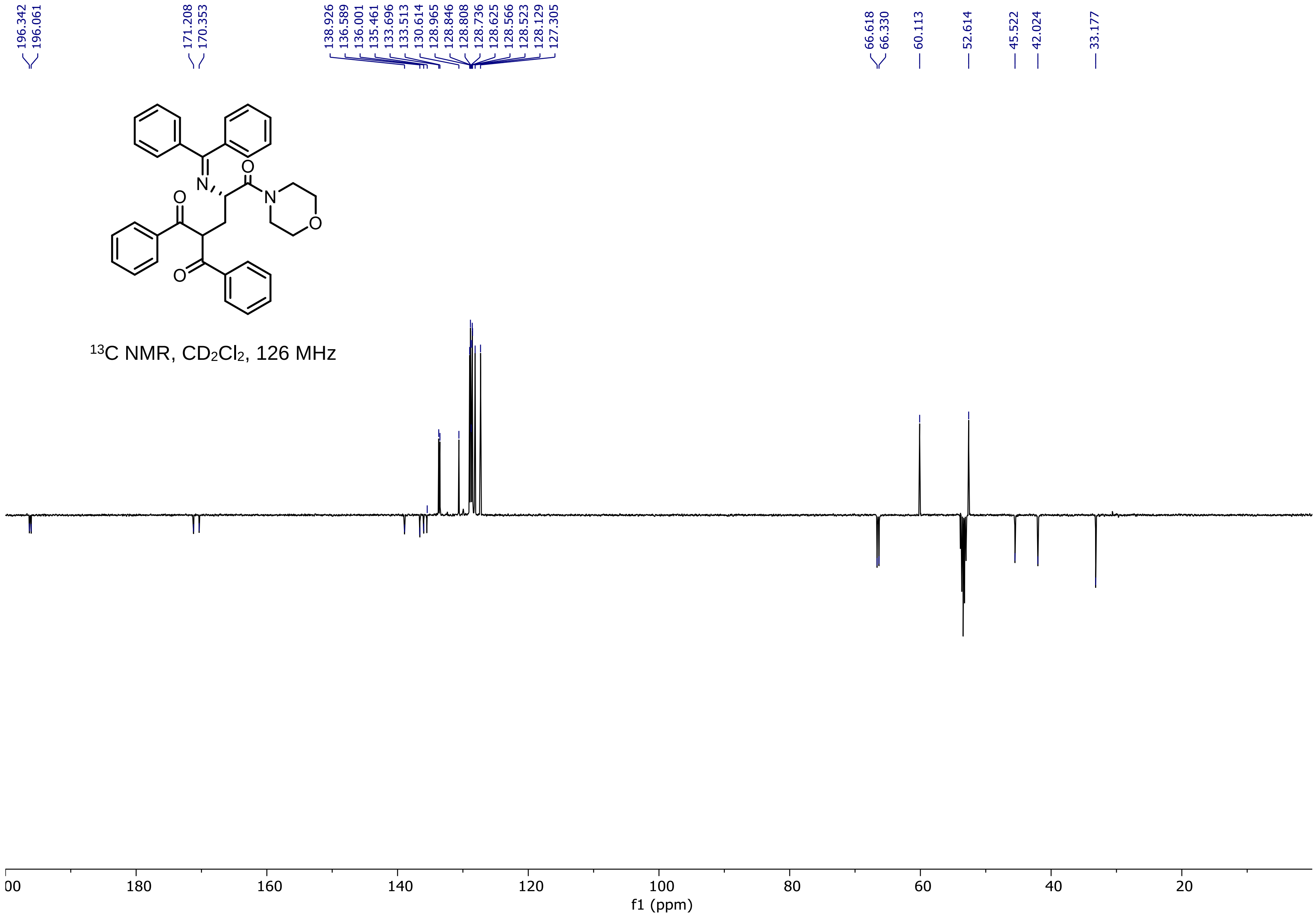
196.342
196.061
171.208
170.353
138.926
136.589
136.001
135.461
133.696
133.513
130.614
128.965
128.846
128.808
128.736
128.625
128.566
128.523
128.129
127.305
66.618
66.330
60.113
52.614
45.522
42.024
33.177
N
O
O
O
N
O
O
$^{13}$C NMR, $CD_2Cl_2$, 126 MHz
f1 (ppm)

HPLC data for **20e**: Chiralcel OD-H (90:10 hexane:IPA, flow rate 1 mL/min, 254 nm, 30 °C) $t_R$: 16.4 min, $t_R$: 23.1 min, 89:11 er

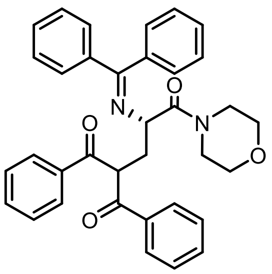

Racemic sample (left), enantioenriched sample (right)

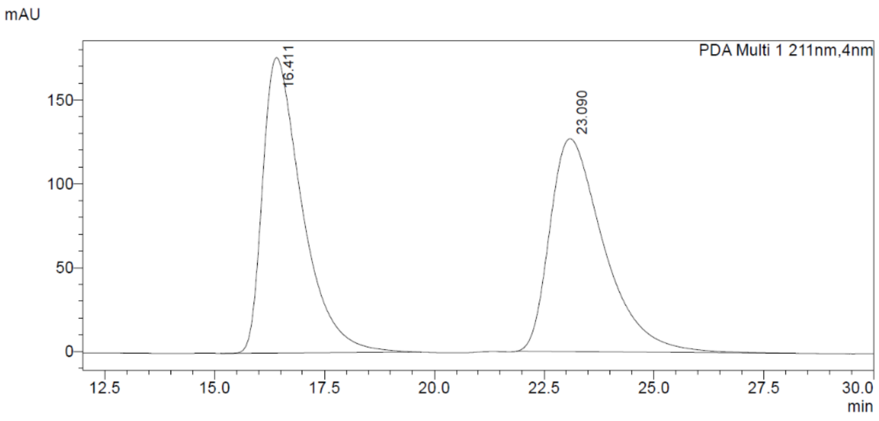


PDA Ch1 211nm

| Peak# | Ret. Time | Area% |
|---|---|---|
| 1 | 16.411 | 50.366 |
| 2 | 23.090 | 49.634 |
| Total | | 100.000 |

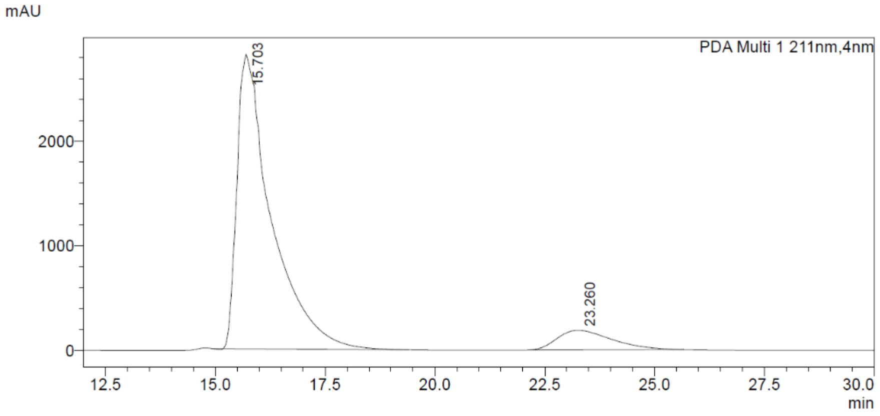


PDA Ch1 211nm

| Peak# | Ret. Time | Area% |
|---|---|---|
| 1 | 15.703 | 90.499 |
| 2 | 23.260 | 9.501 |
| Total | | 100.000 |

**Di-*tert*-butyl (*S*)-2-(2-((diphenylmethylene)amino)-3-morpholino-3-oxopropyl)malonate (20f)**

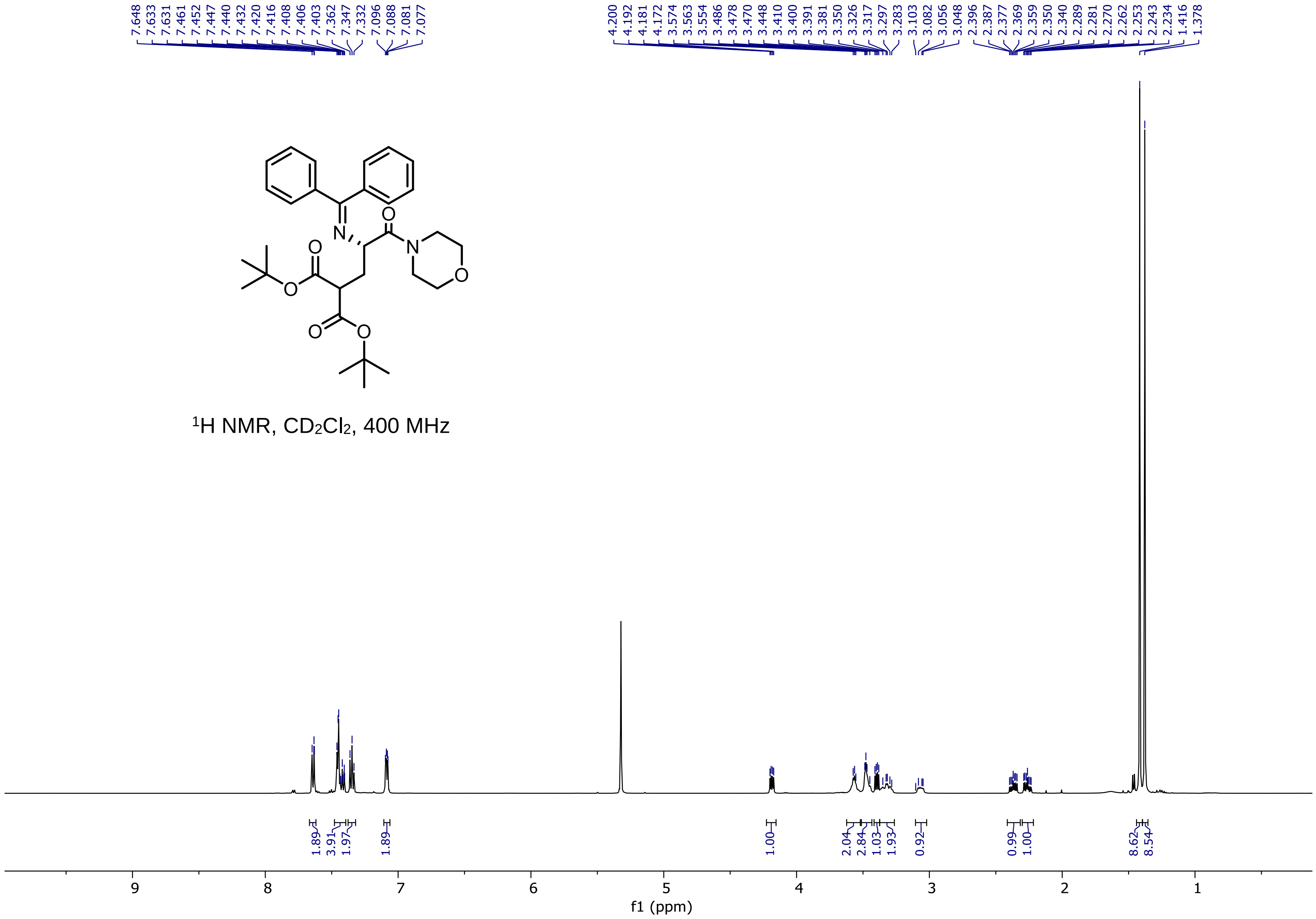

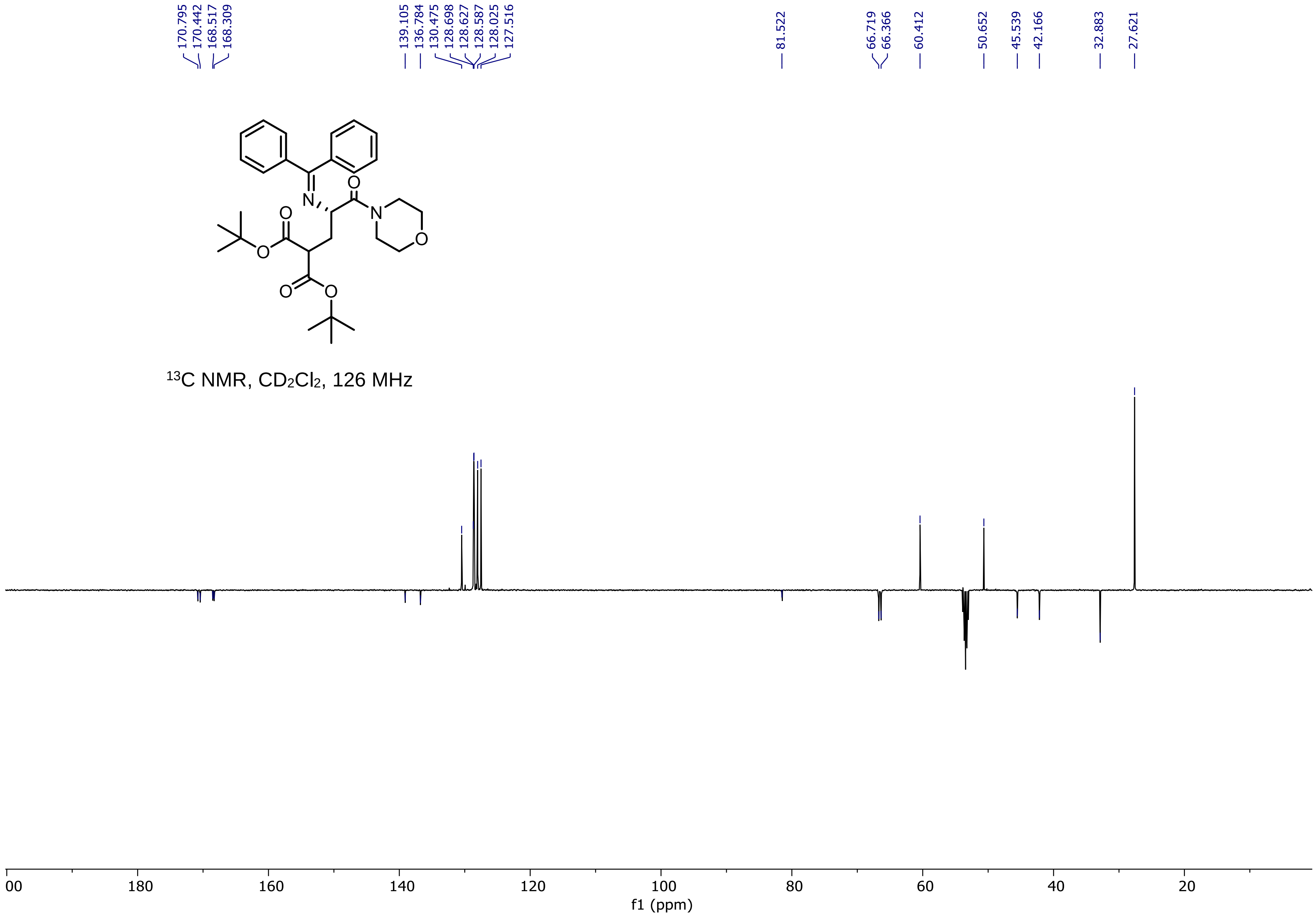

170.795
170.442
168.517
168.309
139.105
136.784
130.475
128.698
128.627
128.587
128.025
127.516
81.522
66.719
66.366
60.412
50.652
45.539
42.166
32.883
27.621
$^{13}$C NMR, $CD_2Cl_2$, 126 MHz
00
180
160
140
120
100
80
60
40
20
f1 (ppm)

HPLC data for **20f**: Chiralpak AD-H (98:2 hexane:IPA, flow rate 1 mL/min, 254 nm, 30 °C) tR: 38.30 min, tR: 44.6 min, 5:95 er

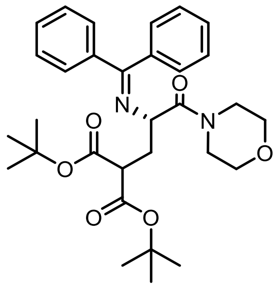

Racemic sample (left), enantioenriched sample (right)

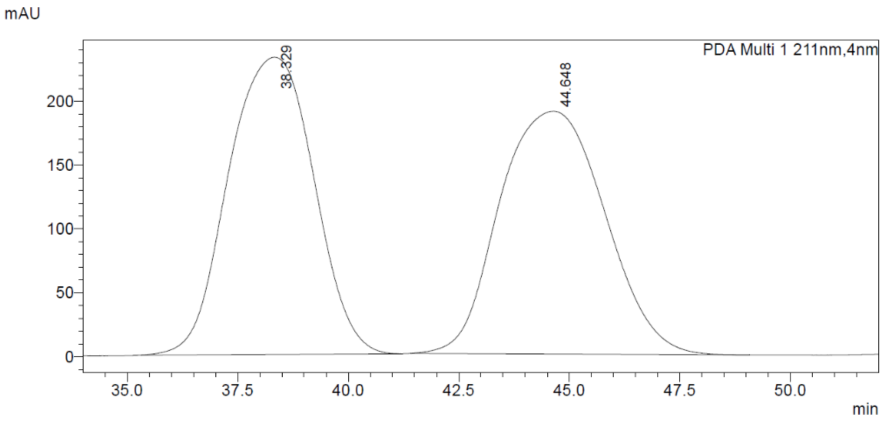


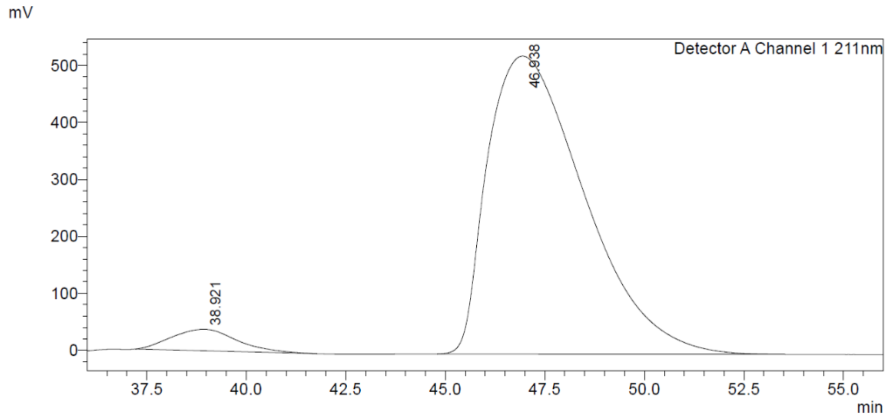


PDA Ch1 211nm

| Peak# | Ret. Time | Area% |
|---|---|---|
| 1 | 38.329 | 49.825 |
| 2 | 44.648 | 50.175 |
| Total | | 100.000 |

**(*S*)-2-((Diphenylmethylene)amino)-4-(4-methoxybenzoyl)-5-(4-methoxyphenyl)-1-morpholinopentane-1,5-dione (20g)**

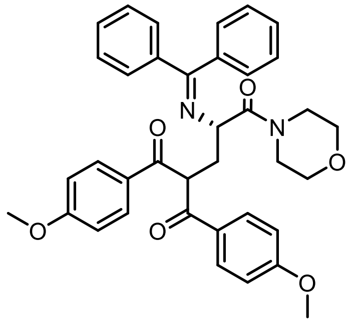

$^{1}$H NMR, $CD_2Cl_2$, 400 MHz

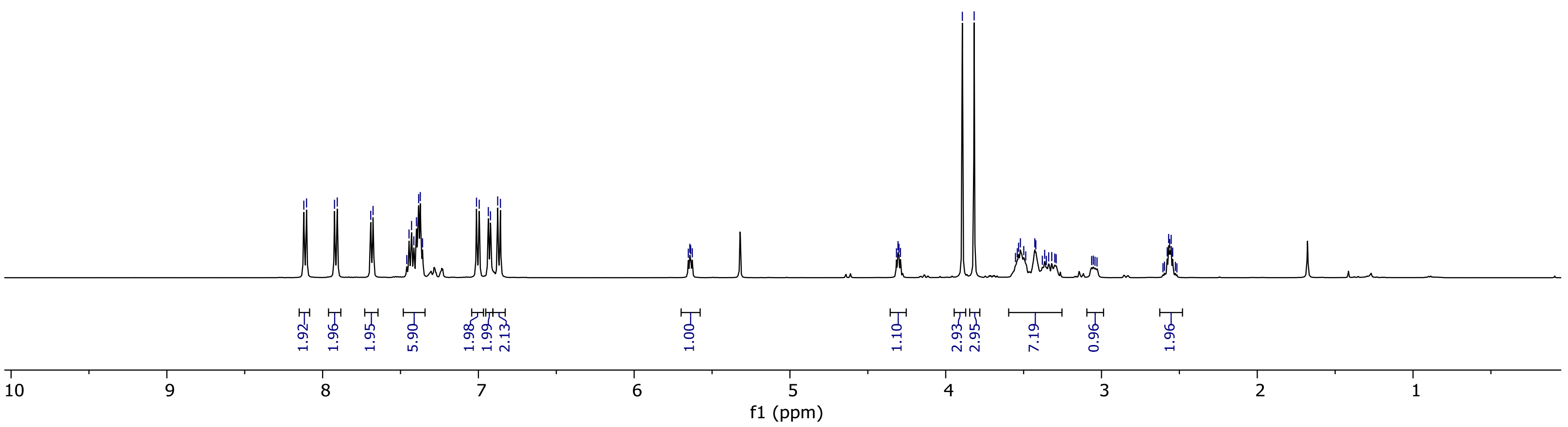

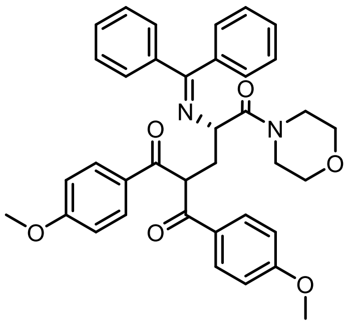

$^{13}C$ NMR, $CD_2Cl_2$, 126 MHz

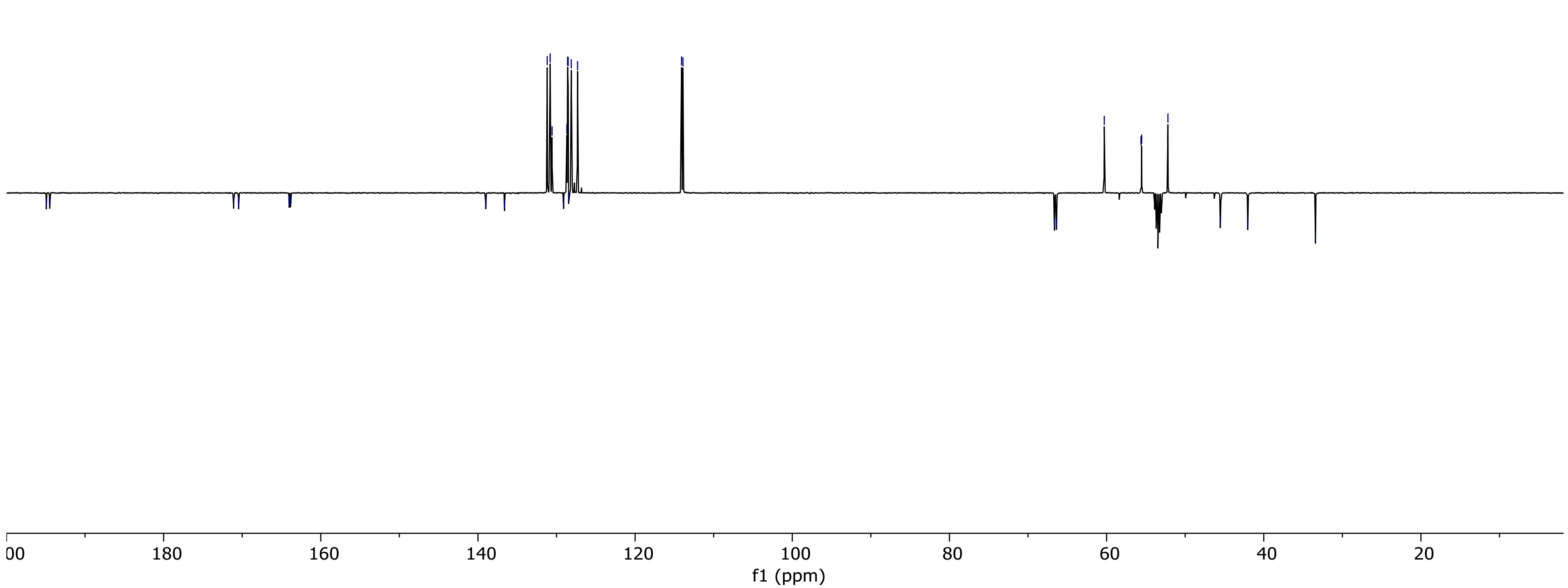

HPLC data for **20g**: Chiralpak AD-H (80:20 Hexane:IPA, flow rate 2 mL/min, 254 nm, 30 °C) $t_R$: 35.1 min, $t_R$: 49.8 min, 14:86 er

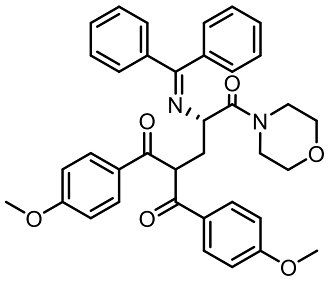

Racemic sample (left), enantioenriched sample (right)

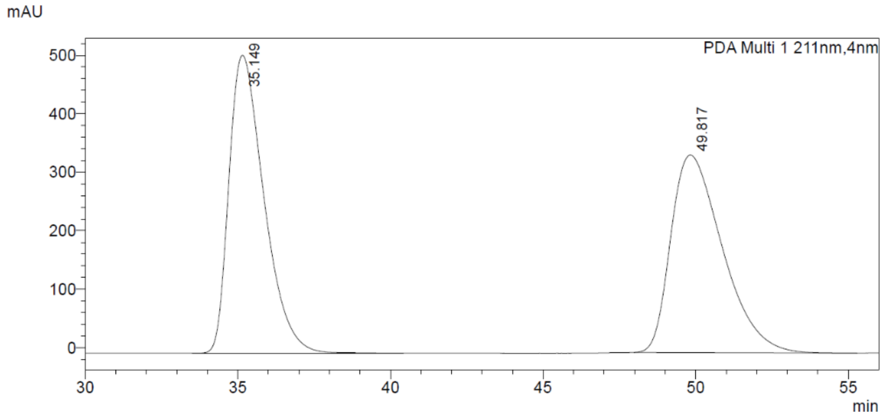


PDA Ch1 211nm

| Peak# | Ret. Time | Area% |
|---|---|---|
| 1 | 35.149 | 50.612 |
| 2 | 49.817 | 49.388 |
| Total | | 100.000 |

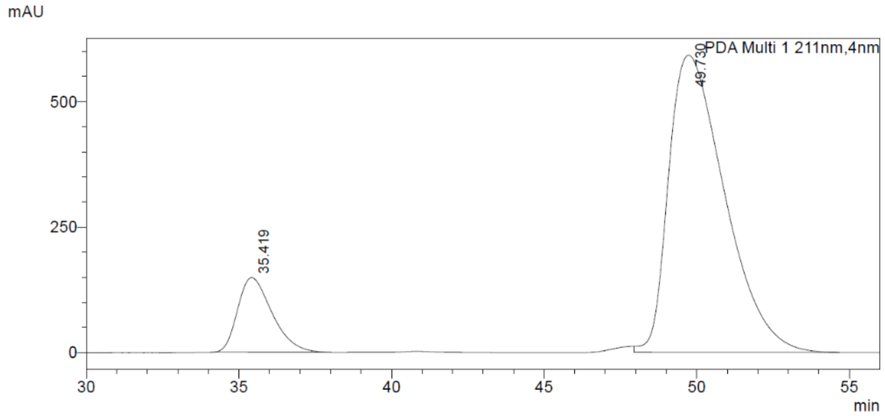


PDA Ch1 211nm

| Peak# | Ret. Time | Area% |
|---|---|---|
| 1 | 35.419 | 13.269 |
| 2 | 49.730 | 86.731 |
| Total | | 100.000 |

**(*S*)-2-(4-(Dimethylamino)benzoyl)-1-(4-(dimethylamino)phenyl)-4-((diphenylmethylene)amino)-5-morpholinopentane-1,5-dione (20h)**

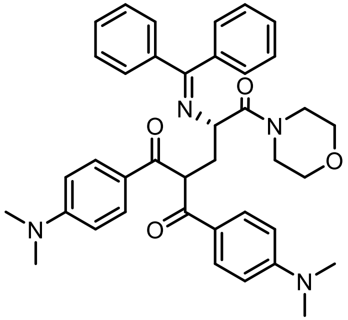

$^{1}$H NMR, $CD_2Cl_2$, 400 MHz

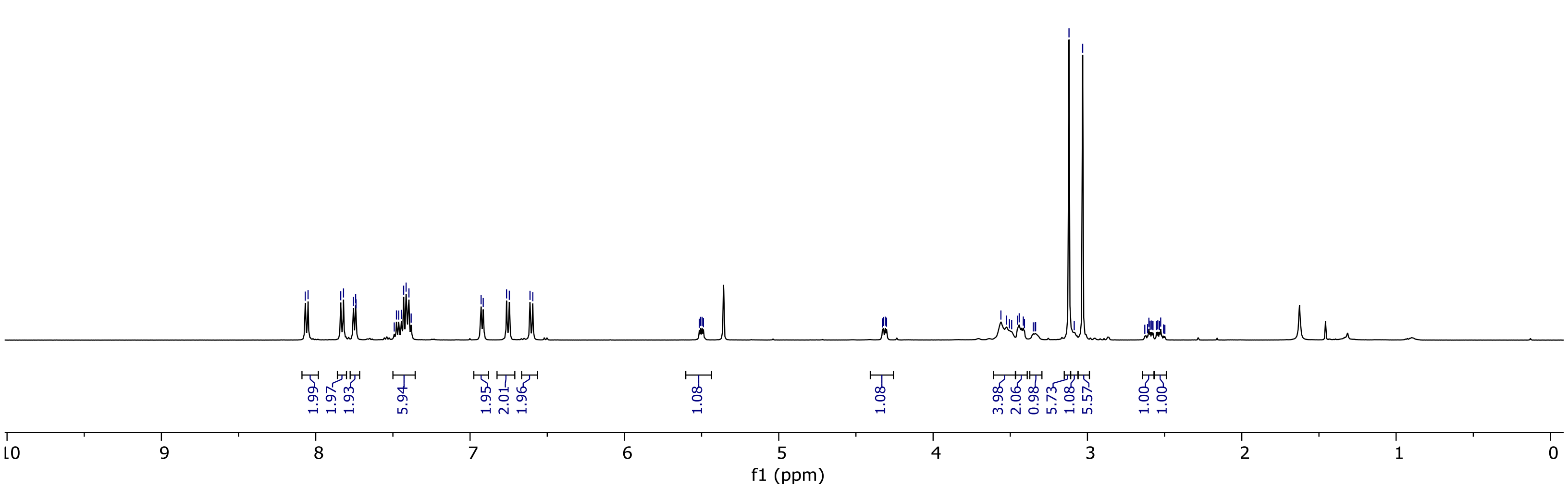

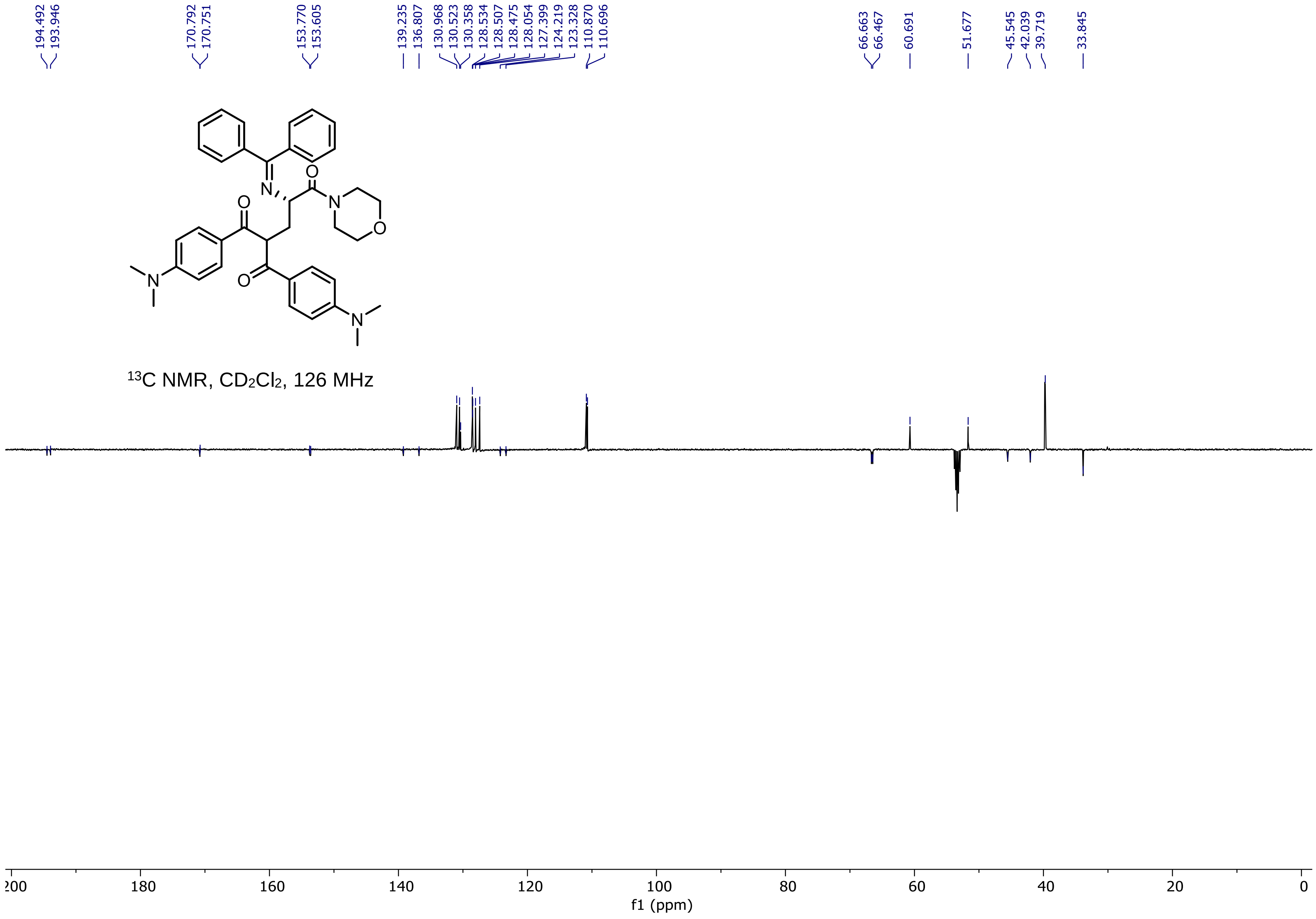
194.492
193.946
170.792
170.751
153.770
153.605
139.235
136.807
130.968
130.523
130.358
128.534
128.507
128.475
128.054
127.399
124.219
123.328
110.870
110.696
66.663
66.467
60.691
51.677
45.545
42.039
39.719
33.845
$^{13}$C NMR, $CD_2Cl_2$, 126 MHz
200
180
160
140
120
100
80
60
40
20
0
f1 (ppm)

HPLC data for **20h**: Chiralpak AD-H (80:20 hexane:IPA, flow rate 1 mL/min, 254 nm, 40 °C) $t_R$: 38.9 min, $t_R$: 43.5 min, 9:91 er

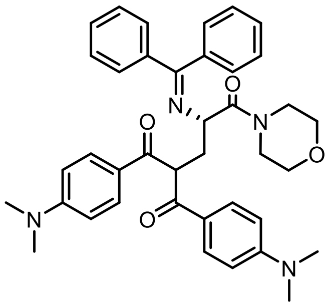


Racemic sample (left), enantioenriched sample (right)

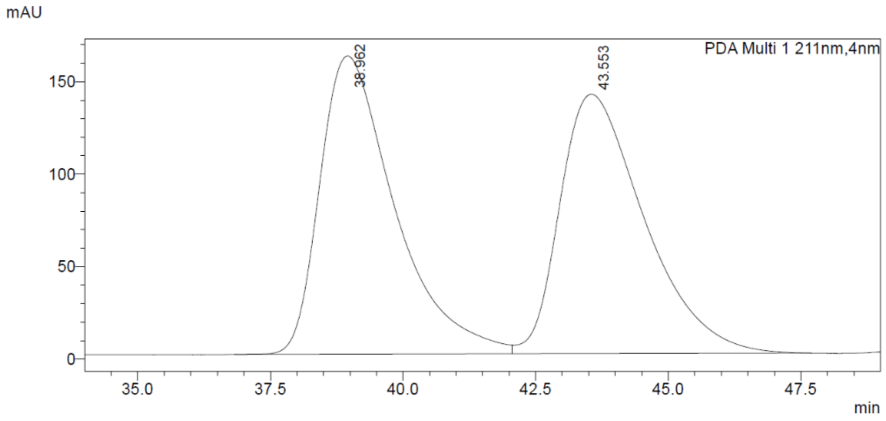


PDA Ch1 211nm

| Peak# | Ret. Time | Area% |
|---|---|---|
| 1 | 38.962 | 50.513 |
| 2 | 43.553 | 49.487 |
| Total | | 100.000 |

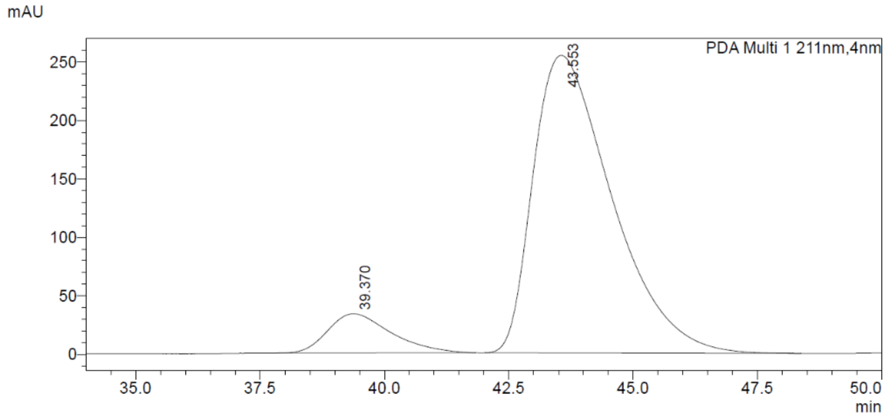


PDA Ch1 211nm

| Peak# | Ret. Time | Area% |
|---|---|---|
| 1 | 39.370 | 9.567 |
| 2 | 43.553 | 90.433 |
| Total | | 100.000 |

**(*S*)-2-(3,4-Dimethoxybenzoyl)-1-(3,4-dimethoxyphenyl)-4-((diphenylmethylene)amino)-5-morpholinopentane-1,5-dione (20i)**

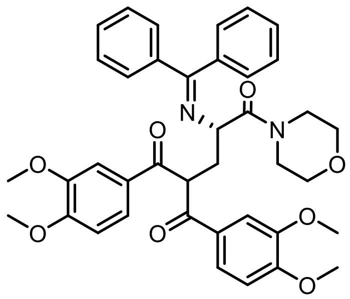

$^1$H NMR, $CD_2Cl_2$, 400 MHz

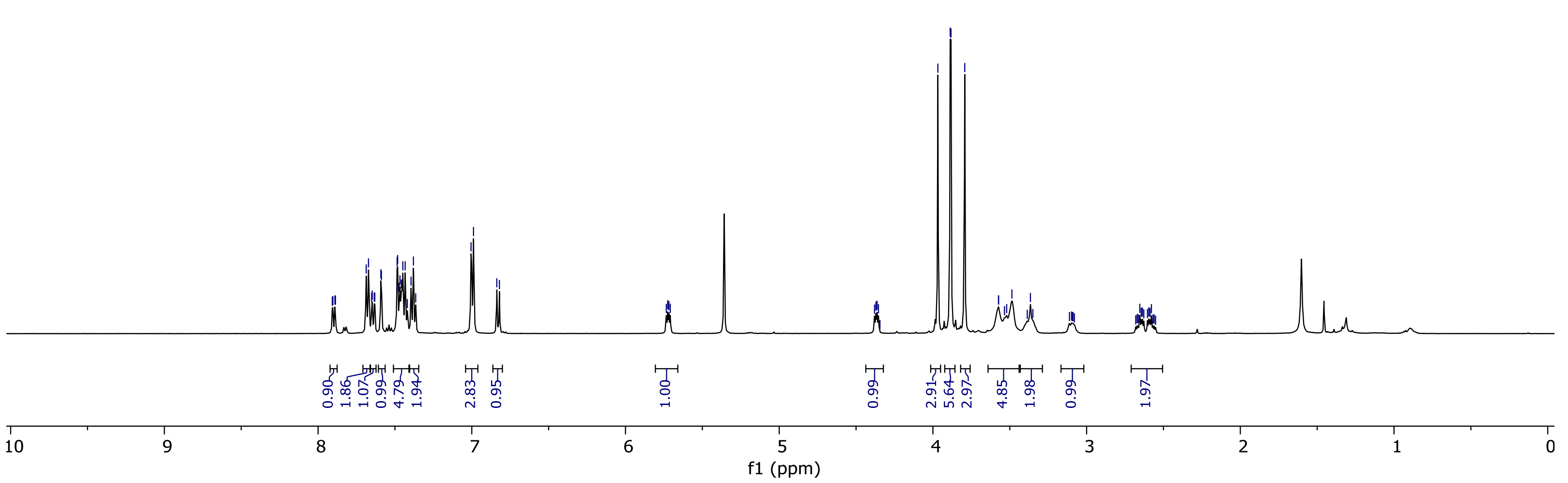

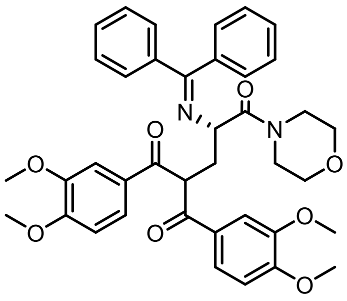

$^{13}C$ NMR, $CD_2Cl_2$, 126 MHz

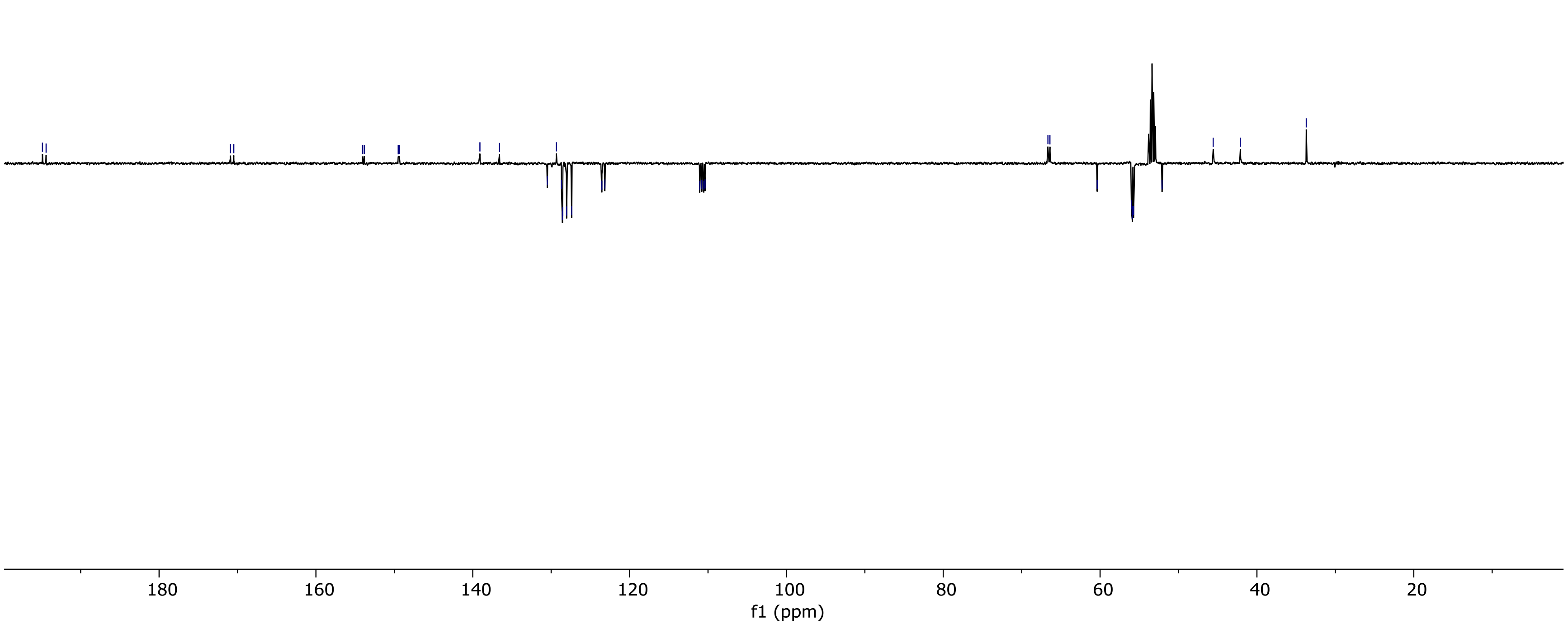

HPLC data for **20i**: Chiralpak IA (80:20 hexane:IPA, flow rate 1 mL/min, 254 nm, 30 °C) $t_R$: 38.2 min, $t_R$: 47.2 min, 86:14 er

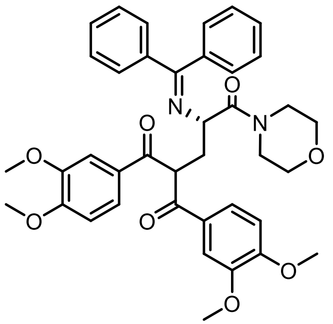

Racemic sample (left), enantioenriched sample (right)

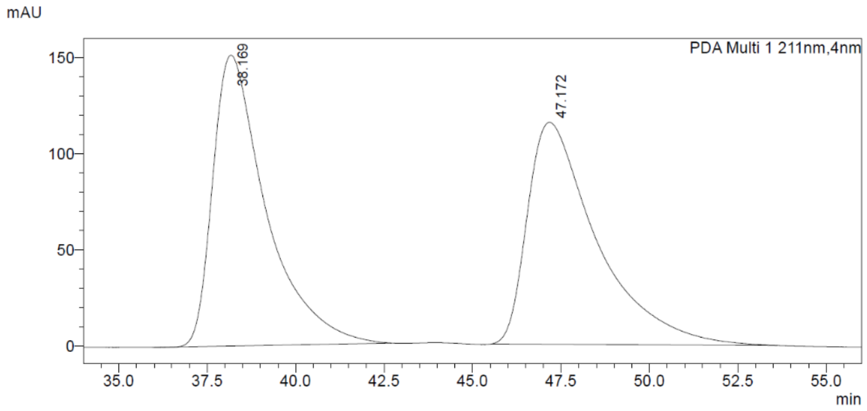


PDA Ch1 211nm

| Peak# | Ret. Time | Area% |
|---|---|---|
| 1 | 38.169 | 50.101 |
| 2 | 47.172 | 49.899 |
| Total | | 100.000 |

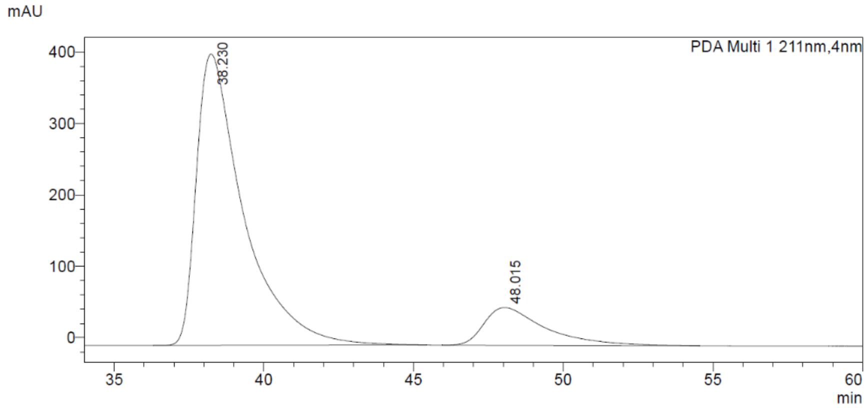


PDA Ch1 211nm

| Peak# | Ret. Time | Area% |
|---|---|---|
| 1 | 38.230 | 85.697 |
| 2 | 48.015 | 14.303 |
| Total | | 100.000 |

**(*S*)-2-(4-Bromobenzoyl)-1-(4-bromophenyl)-4-((diphenylmethylene)amino)-5-morpholinopentane-1,5-dione (20j)**

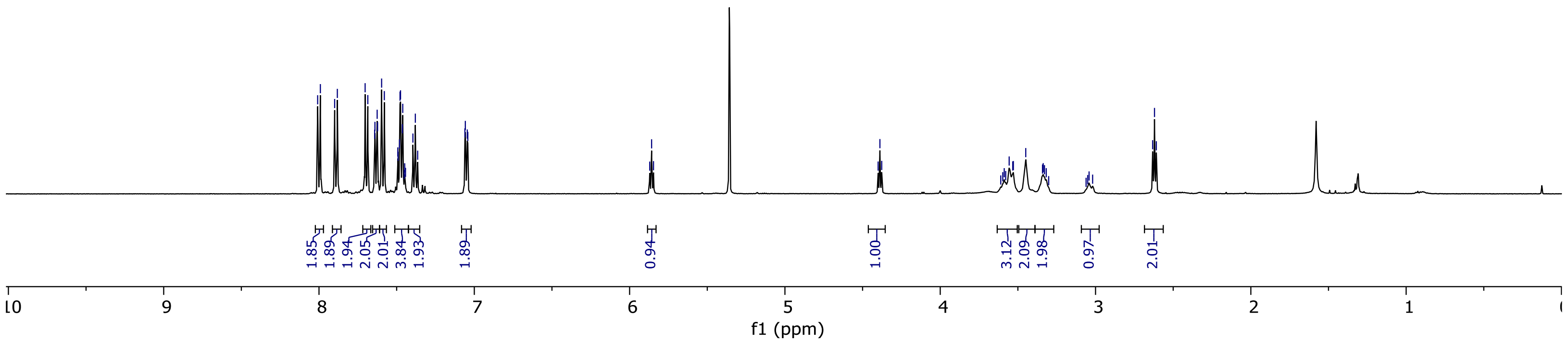


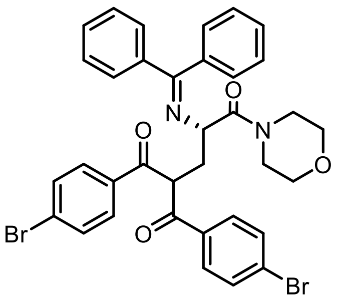


$^1$H NMR, $CD_2Cl_2$, 400 MHz

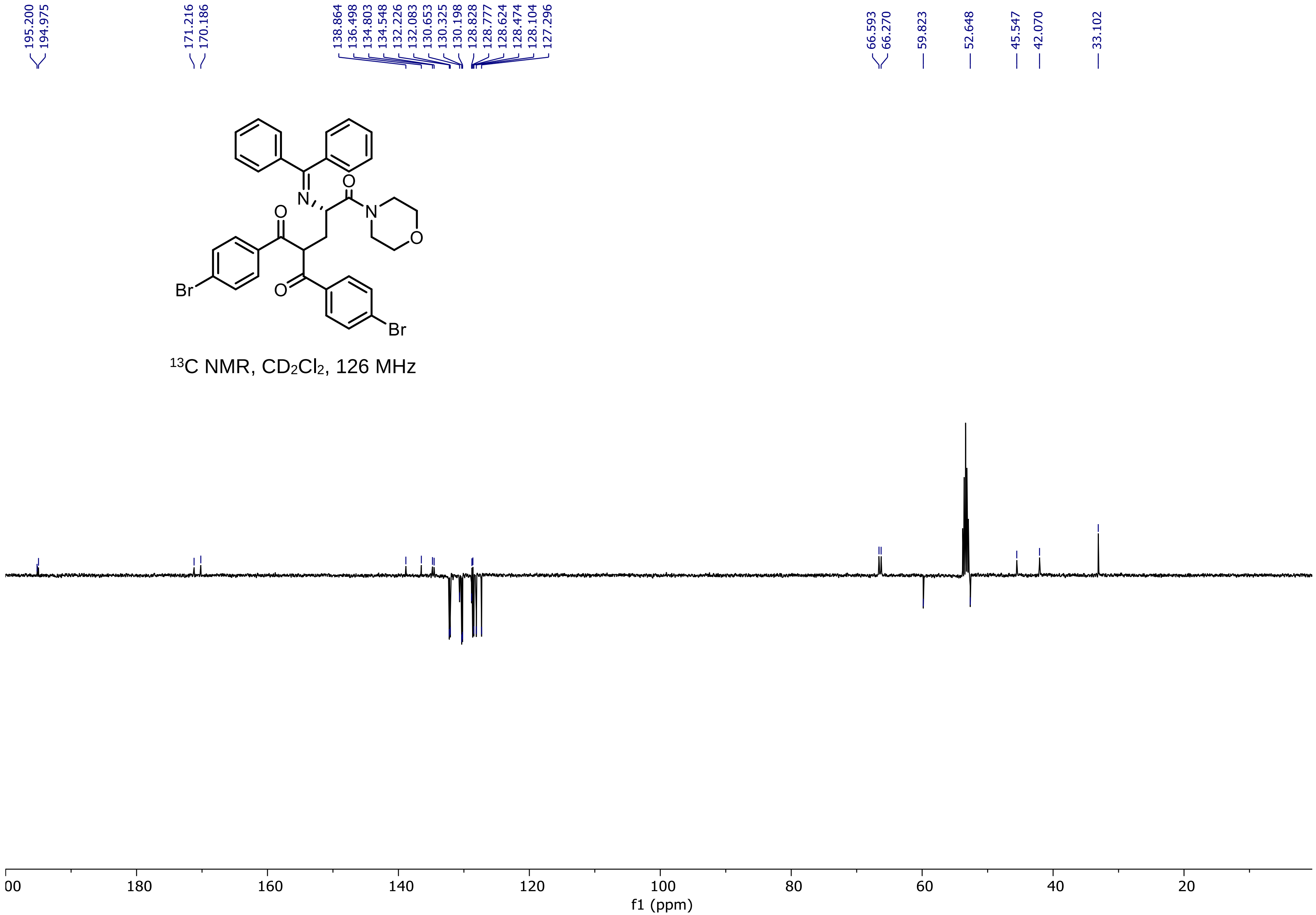
195.200
194.975
171.216
170.186
138.864
136.498
134.803
134.548
132.226
132.083
130.653
130.325
130.198
128.828
128.777
128.624
128.474
128.104
127.296
66.593
66.270
59.823
52.648
45.547
42.070
33.102
Br
Br
O
O
O
N
N
O
$^{13}C$ NMR, $CD_2Cl_2$, 126 MHz
00
180
160
140
120
100
80
60
40
20
f1 (ppm)

HPLC data for **20j**: Chiralpak IB (80:20 hexane : IPA, flow rate 1 mL/min, 254 nm, 30 °C) $t_R$: 8.0 min, $t_R$: 8.4 min, 13:87 er

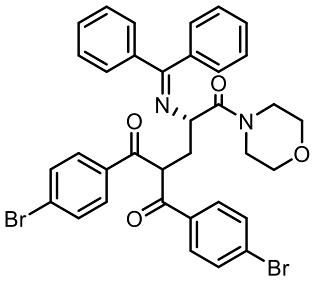


Racemic sample (left), enantioenriched sample (right)

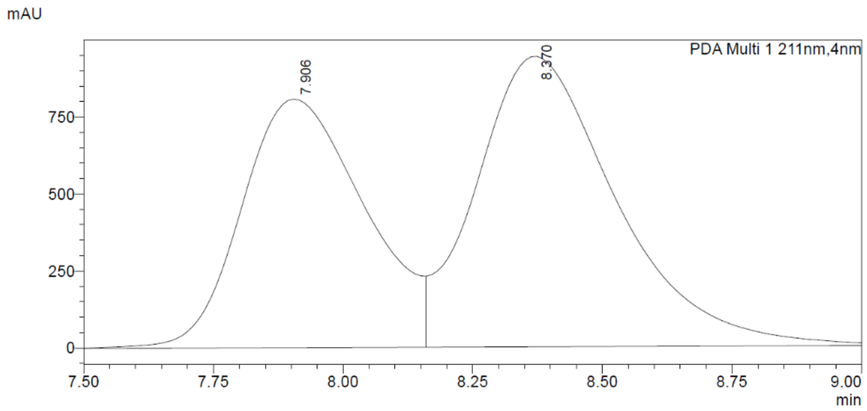


PDA Ch1 211nm

| Peak# | Ret. Time | Area% |
|---|---|---|
| 1 | 7.906 | 43.093 |
| 2 | 8.370 | 56.907 |
| Total | | 100.000 |

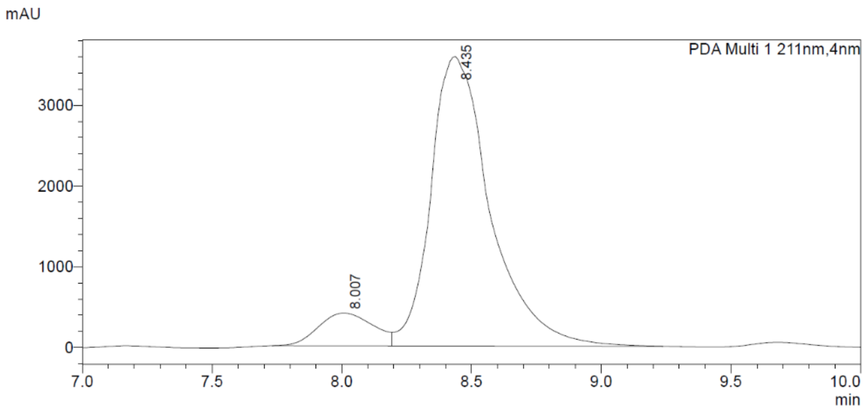


PDA Ch1 211nm

| Peak# | Ret. Time | Area% |
|---|---|---|
| 1 | 8.007 | 9.464 |
| 2 | 8.435 | 90.536 |
| Total | | 100.000 |

**(*S*)-2-(4-Chlorobenzoyl)-1-(4-chlorophenyl)-4-((diphenylmethylene)amino)-5-morpholinopentane-1,5-dione (20k)**

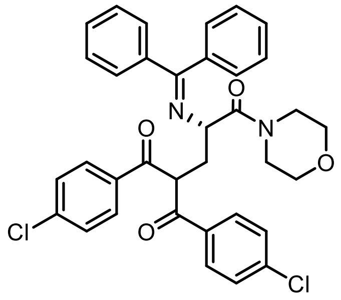


$^{1}$H NMR, $CD_2Cl_2$, 400 MHz

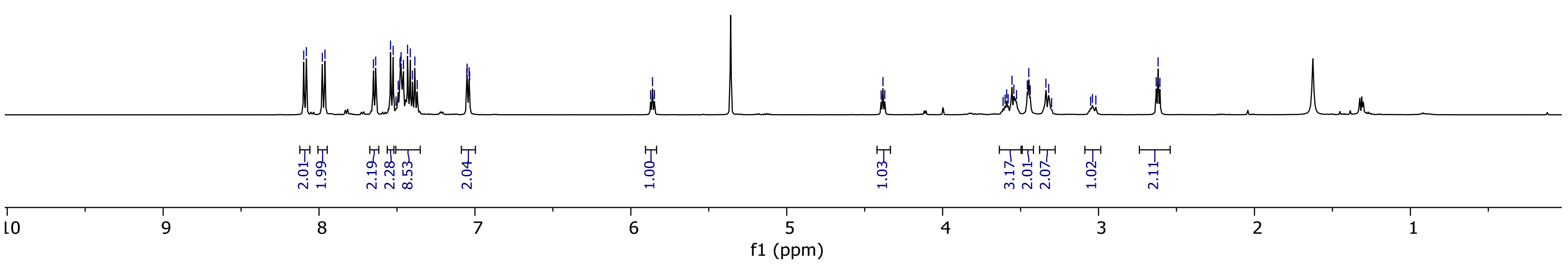

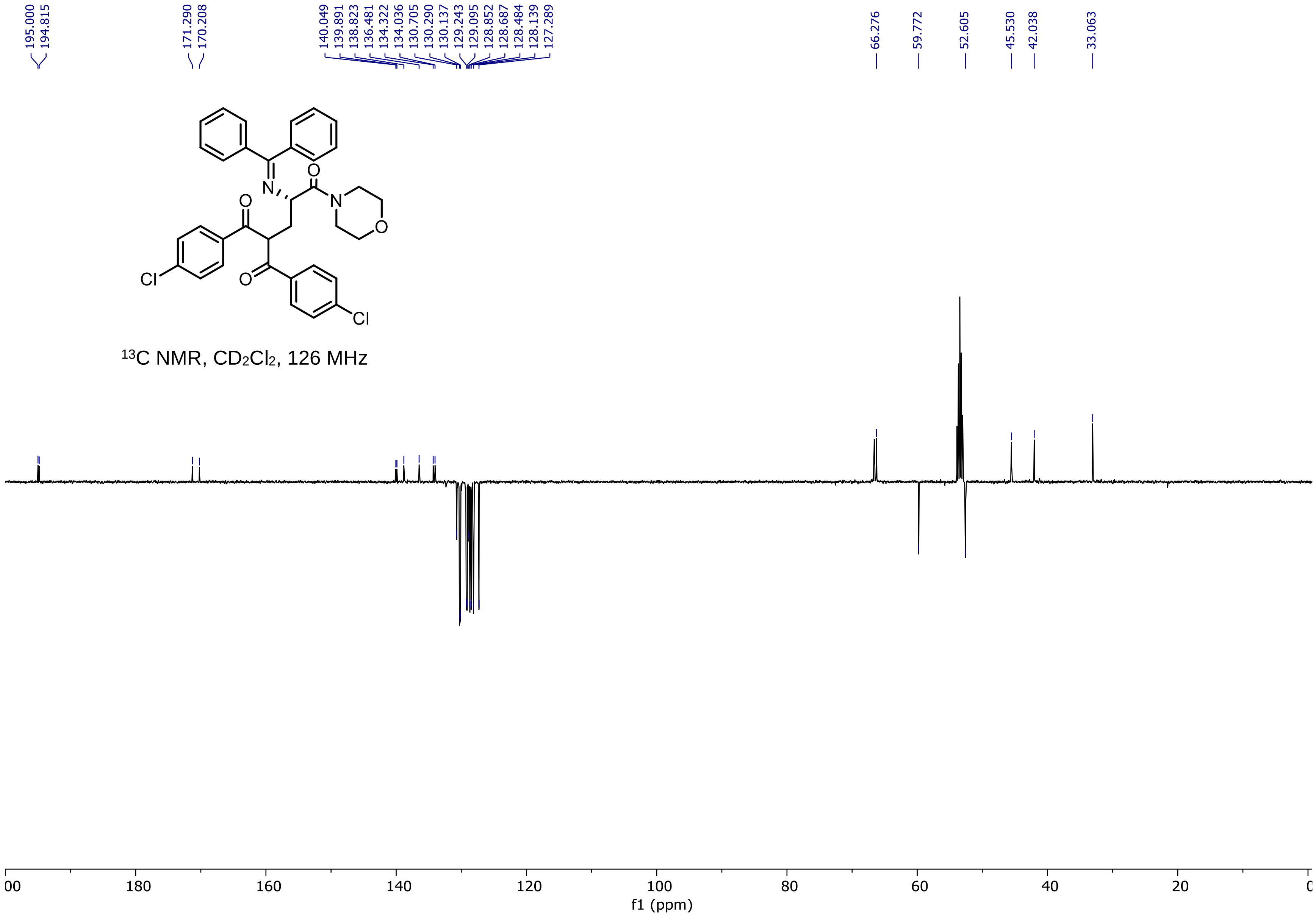
195.000
194.815
171.290
170.208
140.049
139.891
138.823
136.481
134.322
134.036
130.705
130.290
130.137
129.243
129.095
128.852
128.687
128.484
128.139
127.289
66.276
59.772
52.605
45.530
42.038
33.063
N
O
O
O
N
O
Cl
Cl
13C NMR, CD2Cl2, 126 MHz
180
160
140
120
100
80
60
40
20
f1 (ppm)

HPLC data for **20k**: Chiralpak IB (85:15 hexane : IPA, flow rate 1 mL/min, 254 nm, 30 °C) $t_R$: 8.8 min, $t_R$: 9.3 min, 14:86 er

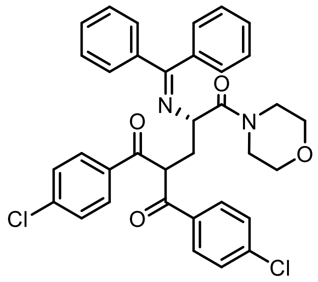


Racemic sample (left), enantioenriched sample (right)

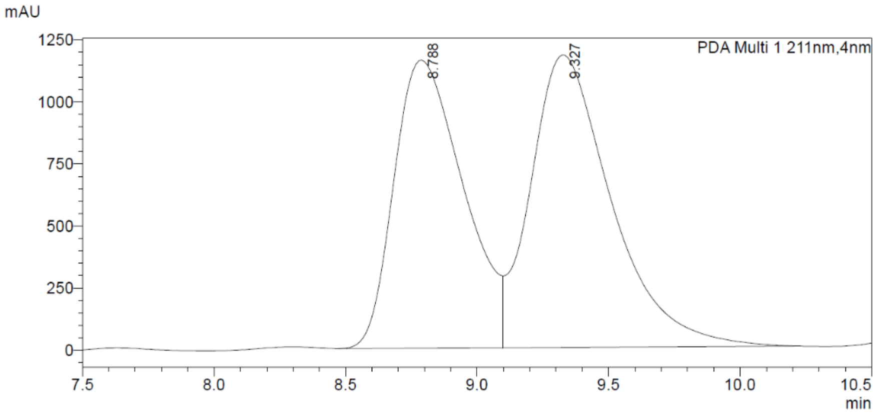


PDA Ch1 211nm

| Peak# | Ret. Time | Area% |
|---|---|---|
| 1 | 8.788 | 46.037 |
| 2 | 9.327 | 53.963 |
| Total | | 100.000 |

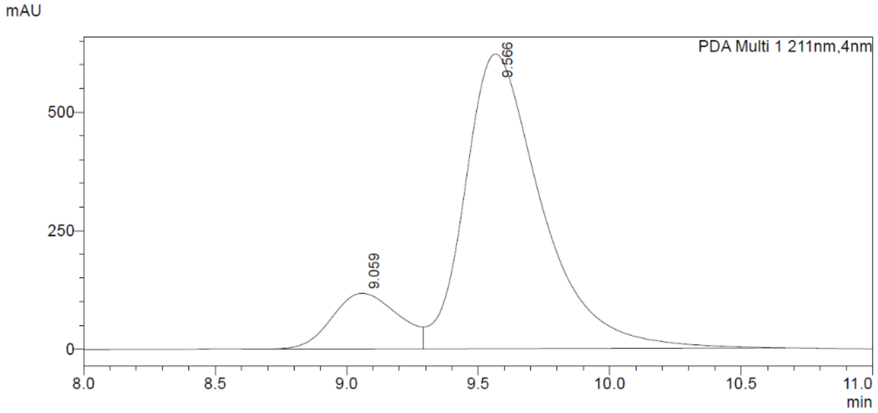


PDA Ch1 211nm

| Peak# | Ret. Time | Area% |
|---|---|---|
| 1 | 9.059 | 14.152 |
| 2 | 9.566 | 85.848 |
| Total | | 100.000 |

**(*S*)-2-(2-Naphthoyl)-4-((diphenylmethylene)amino)-5-morpholino-1-(naphthalen-2-yl)pentane-1,5-dione (20l)**

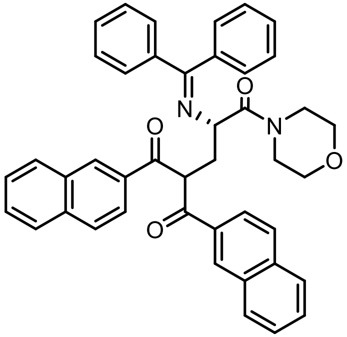

$^1$H NMR, $CD_2Cl_2$, 400 MHz

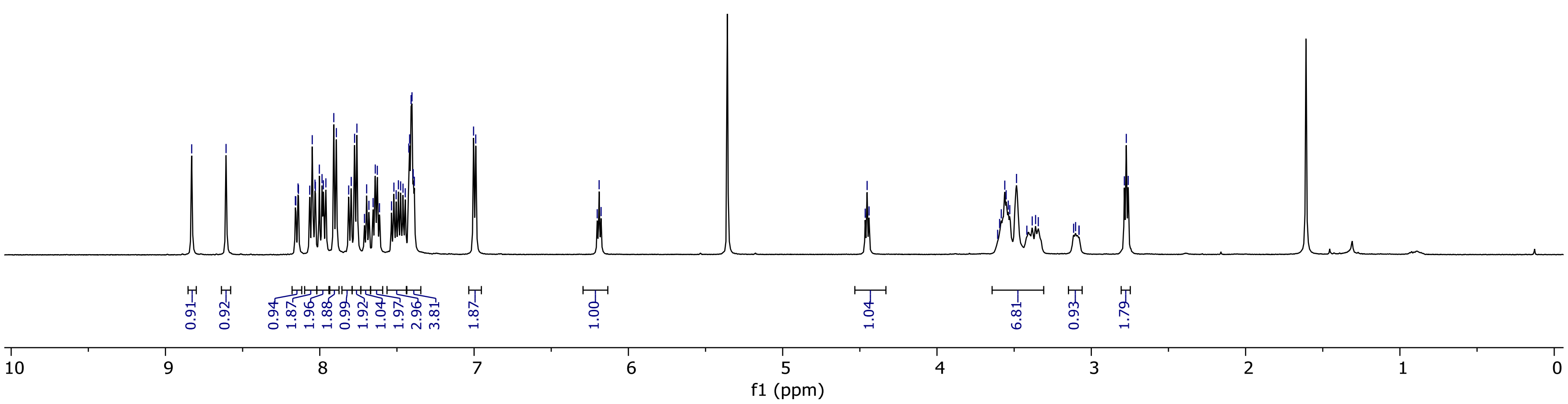

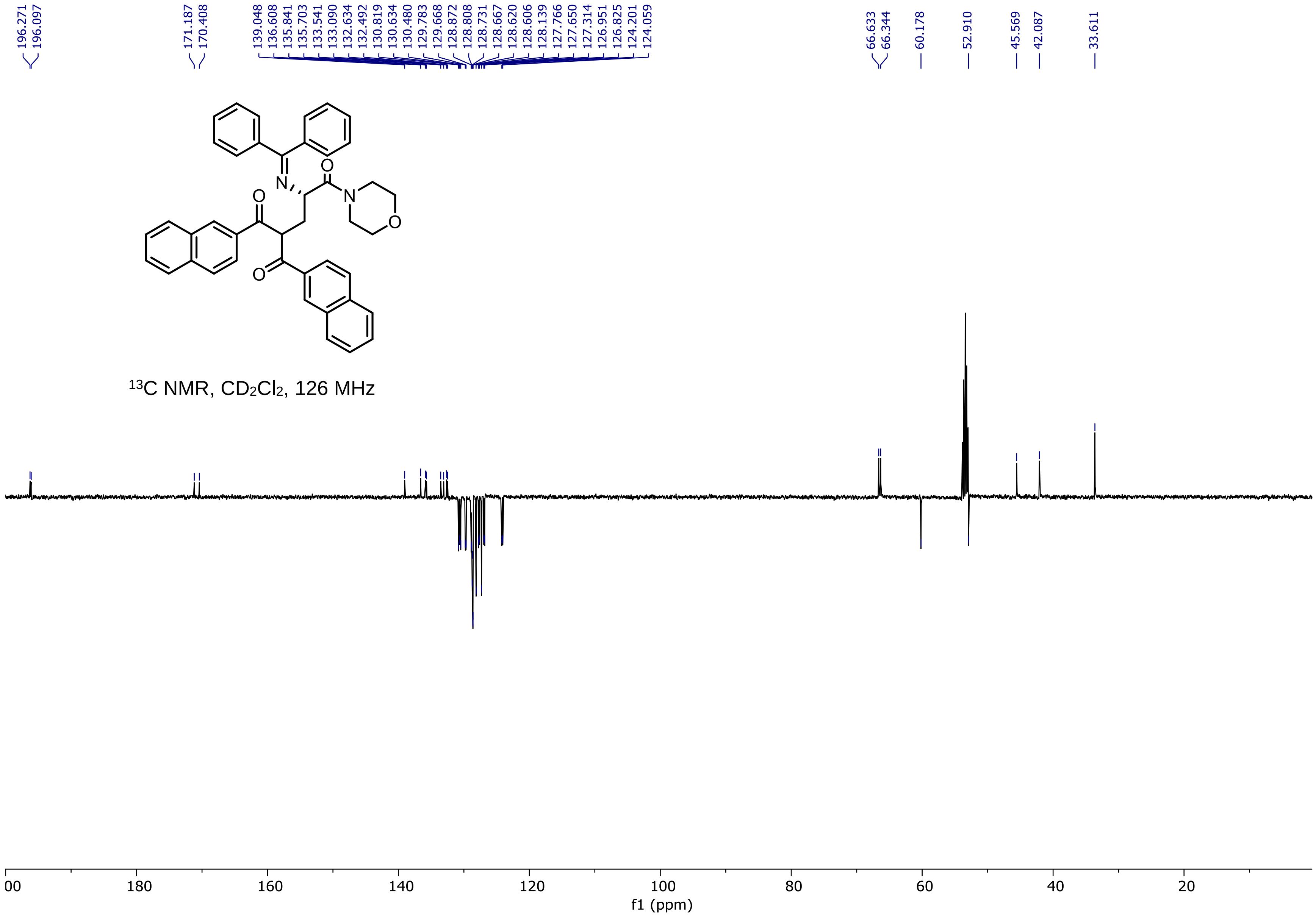

196.271
196.097
171.187
170.408
139.048
136.608
135.841
135.703
133.541
133.090
132.634
132.492
130.819
130.634
130.480
129.783
129.668
128.872
128.808
128.731
128.667
128.620
128.606
128.139
127.766
127.650
127.314
126.951
126.825
124.201
124.059
66.633
66.344
60.178
52.910
45.569
42.087
33.611
$^{13}$C NMR, $CD_2Cl_2$, 126 MHz
00
180
160
140
120
100
80
60
40
20
f1 (ppm)

HPLC data for **20l**: Chiralpak IA (80:20 Hexane:IPA, flow rate 1 mL/min, 254 nm, 30 °C) $t_R$: 23.7 min, $t_R$: 29.4 min, 89:11 er

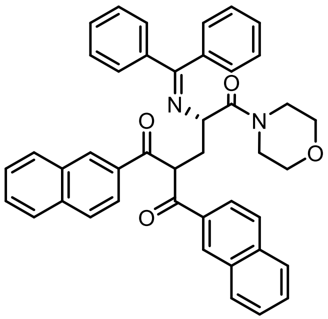


Racemic sample (left), enantioenriched sample (right)

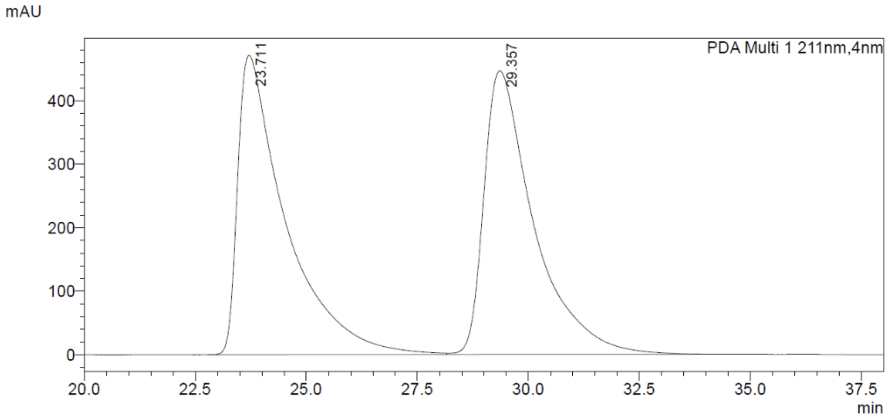


PDA Ch1 211nm

| Peak# | Ret. Time | Area% |
|---|---|---|
| 1 | 23.711 | 50.286 |
| 2 | 29.357 | 49.714 |
| Total | | 100.000 |

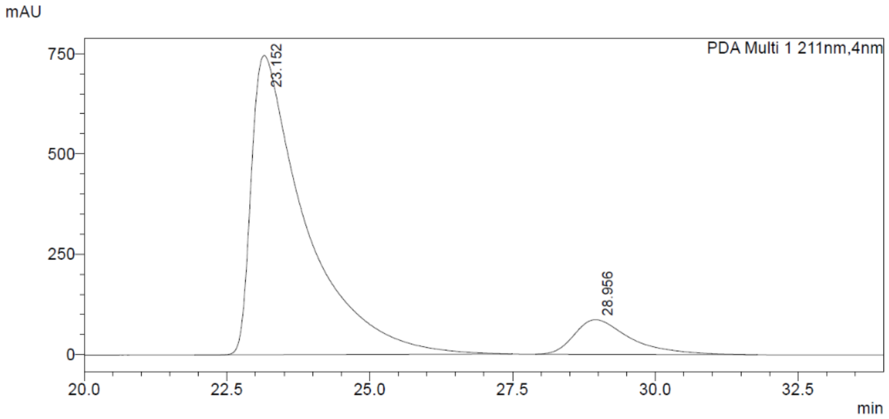


PDA Ch1 211nm

| Peak# | Ret. Time | Area% |
|---|---|---|
| 1 | 23.152 | 89.329 |
| 2 | 28.956 | 10.671 |
| Total | | 100.000 |

**(*S*)-2-(1-Naphthoyl)-4-((diphenylmethylene)amino)-5-morpholino-1-(naphthalen-1-yl)pentane-1,5-dione (20m)**

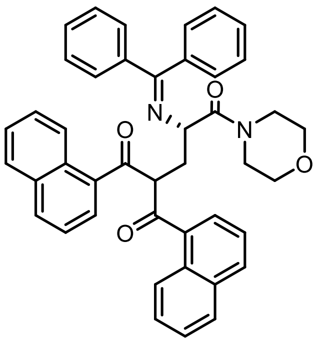


$^1$H NMR, $CD_2Cl_2$, 400 MHz

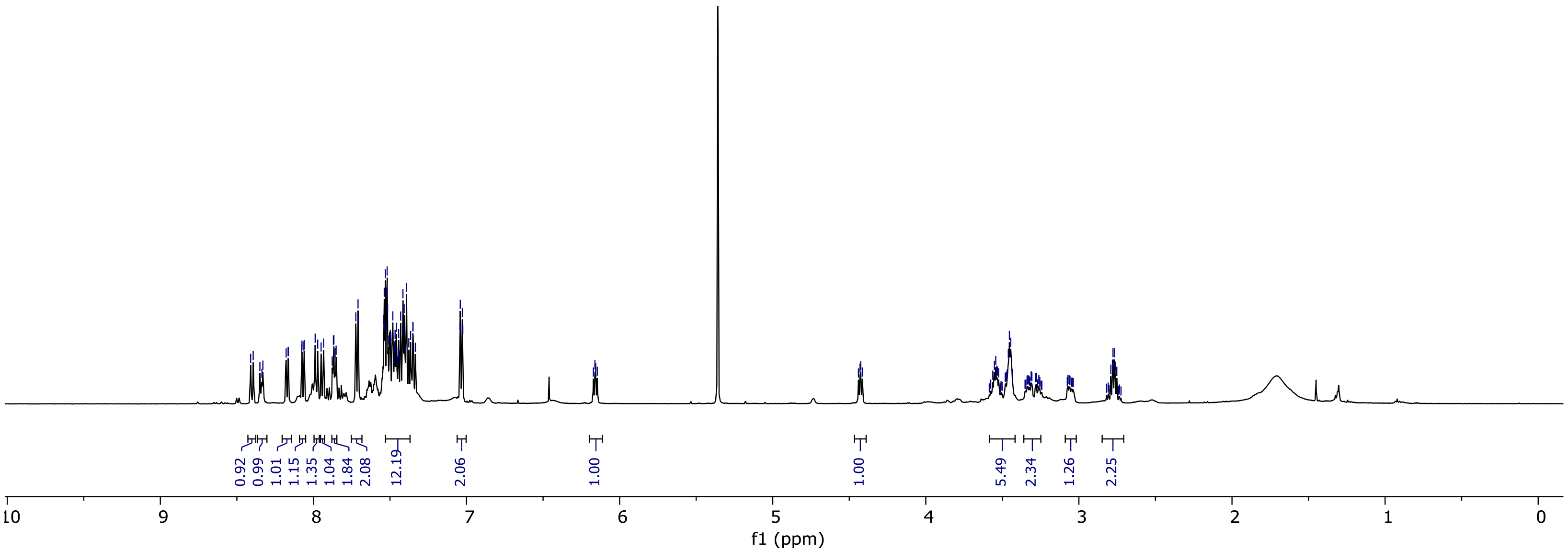

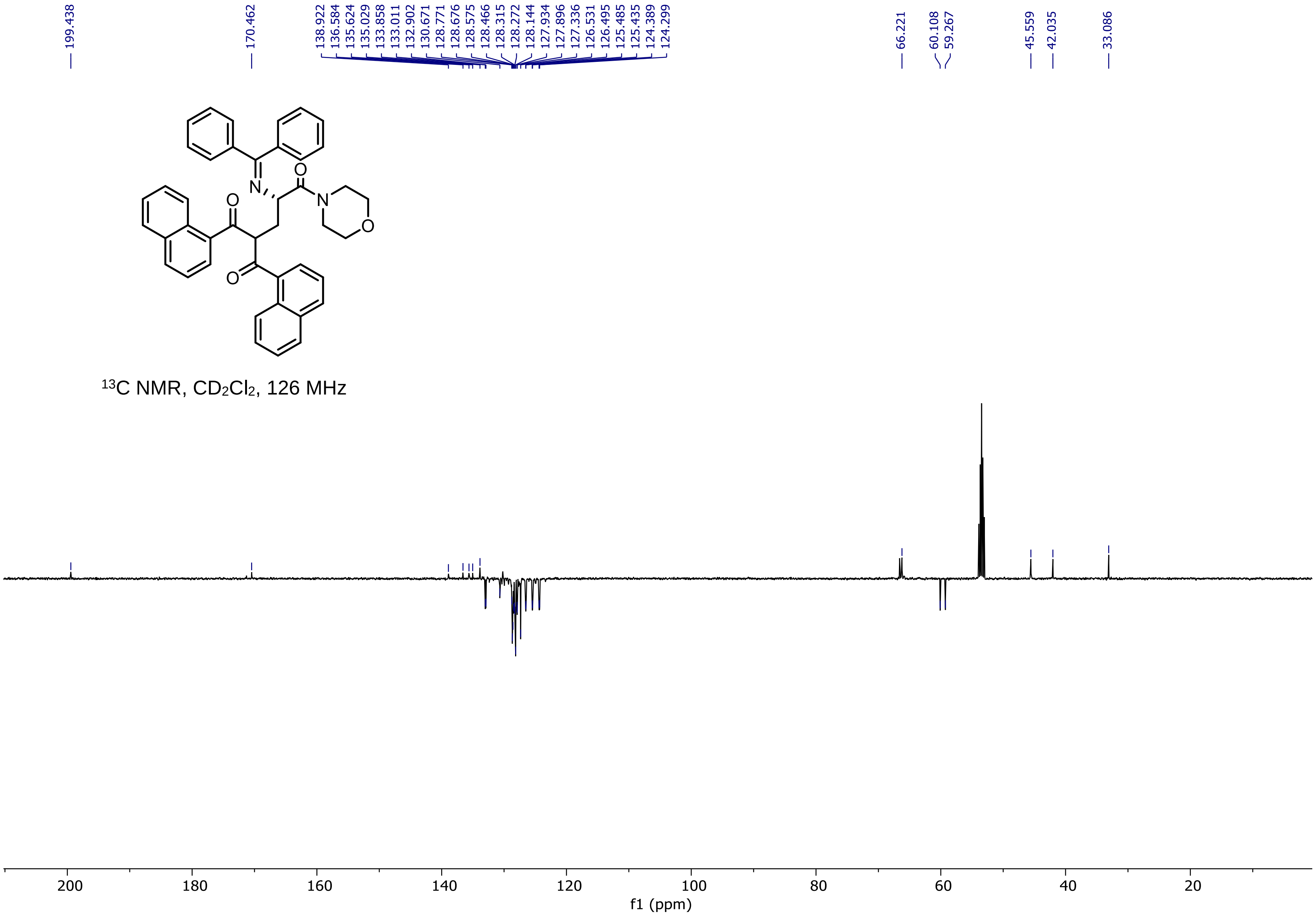
199.438
170.462
138.922
136.584
135.624
135.029
133.858
133.011
132.902
130.671
128.771
128.676
128.575
128.466
128.315
128.272
128.144
127.934
127.896
127.336
126.531
126.495
125.485
125.435
124.389
124.299
66.221
60.108
59.267
45.559
42.035
33.086
O
N
O
N
O
O
$^{13}$C NMR, $CD_2Cl_2$, 126 MHz
200
180
160
140
120
100
80
60
40
20
f1 (ppm)

HPLC data for **20m**: Chiralcel OD-H (90:10 hexane : IPA, flow rate 1 mL/min, 254 nm, 30 °C) $t_R$: 24.7 min, $t_R$: 30.3 min, 24:76 er

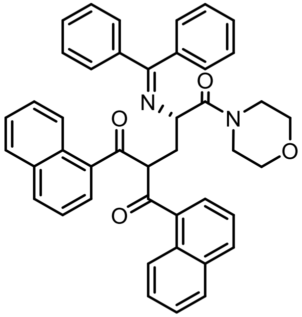

Racemic sample (left), enantioenriched sample (right)

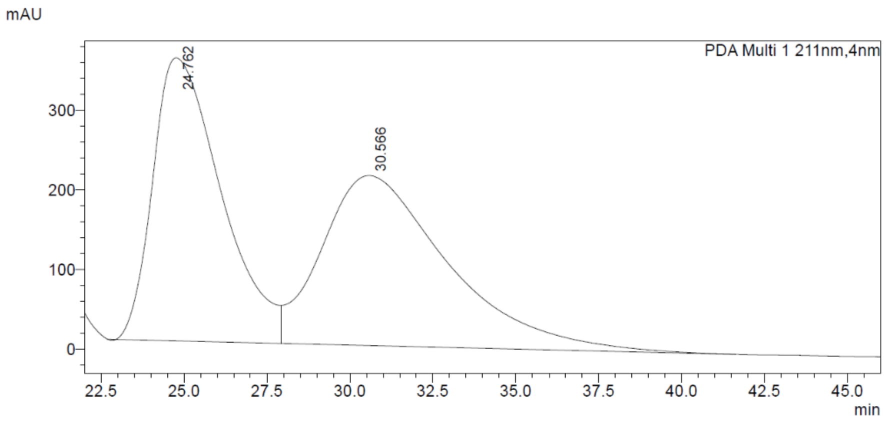


PDA Ch1 211nm

| Peak# | Ret. Time | Area% |
|---|---|---|
| 1 | 24.762 | 47.433 |
| 2 | 30.566 | 52.567 |
| Total | | 100.000 |

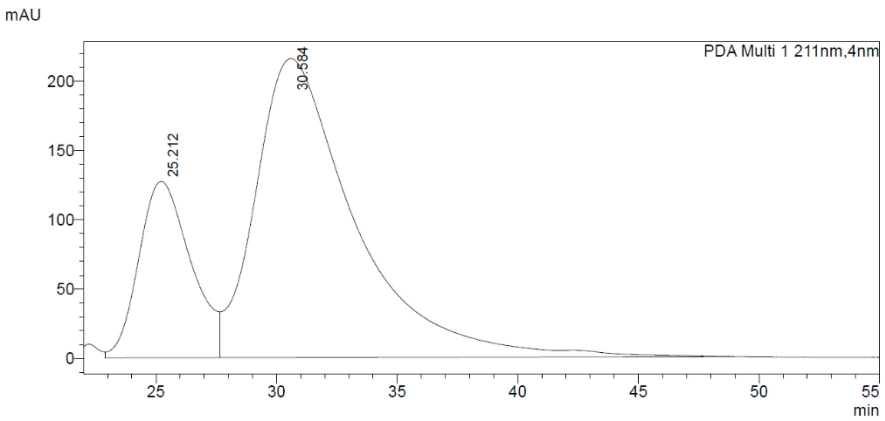


PDA Ch1 211nm

| Peak# | Ret. Time | Area% |
|---|---|---|
| 1 | 25.212 | 23.539 |
| 2 | 30.584 | 76.461 |
| Total | | 100.000 |

**(*S*)-2-((Diphenylmethylene)amino)-1-morpholino-5-(thiophen-2-yl)-4-(thiophene-2-carbonyl)pentane-1,5-dione (20n)**

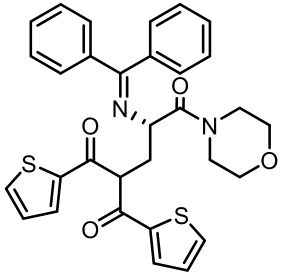

$^1$H NMR, $CD_2Cl_2$, 400 MHz

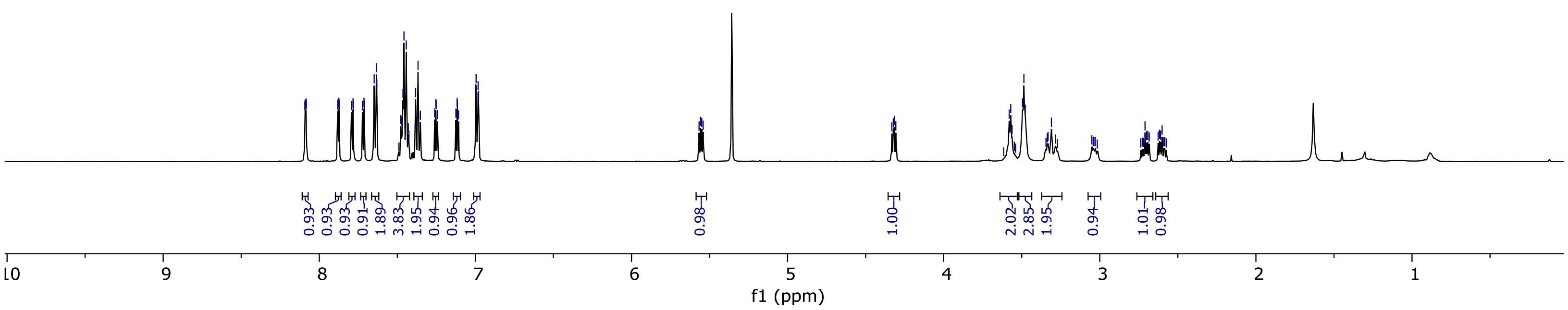

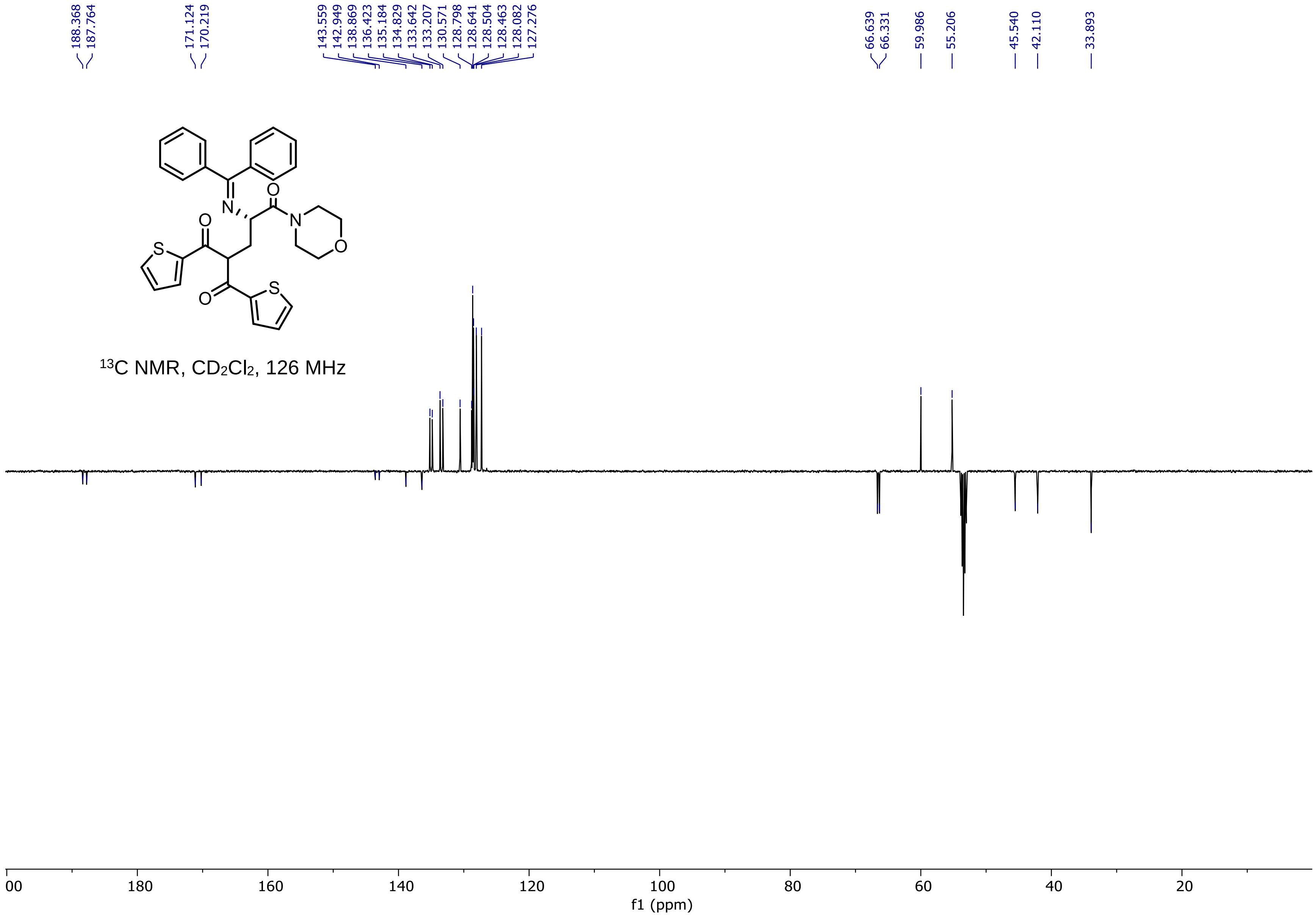

188.368
187.764
171.124
170.219
143.559
142.949
138.869
136.423
135.184
134.829
133.642
133.207
130.571
128.798
128.641
128.504
128.463
128.082
127.276
66.639
66.331
59.986
55.206
45.540
42.110
33.893
$^{13}$C NMR, $CD_2Cl_2$, 126 MHz
00
180
160
140
120
100
80
60
40
20
f1 (ppm)

HPLC data for **20n**: Chiralpak IA (80:20 hexane:IPA, flow rate 1 mL/min, 254 nm, 30 °C) $t_R$: 16.2 min, $t_R$: 24.9 min, 58:42 er

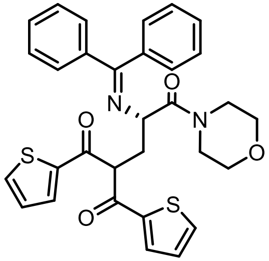


Racemic sample (left), enantioenriched sample (right)

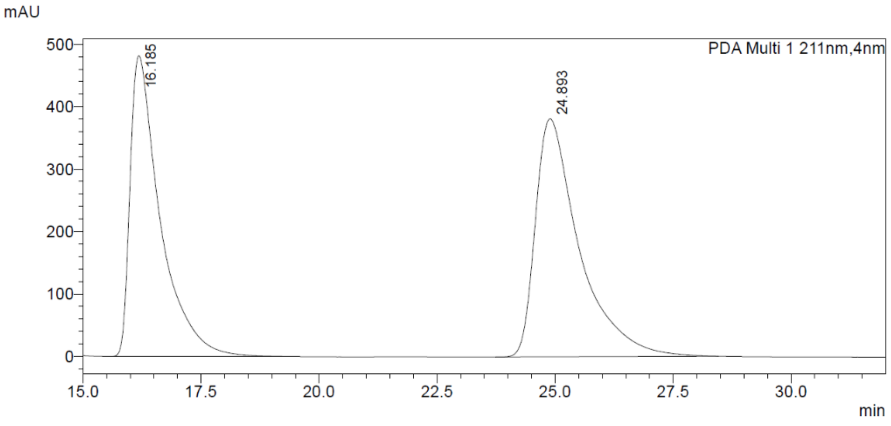


PDA Ch1 211nm

| Peak# | Ret. Time | Area% |
|---|---|---|
| 1 | 16.185 | 46.440 |
| 2 | 24.893 | 53.560 |
| Total | | 100.000 |

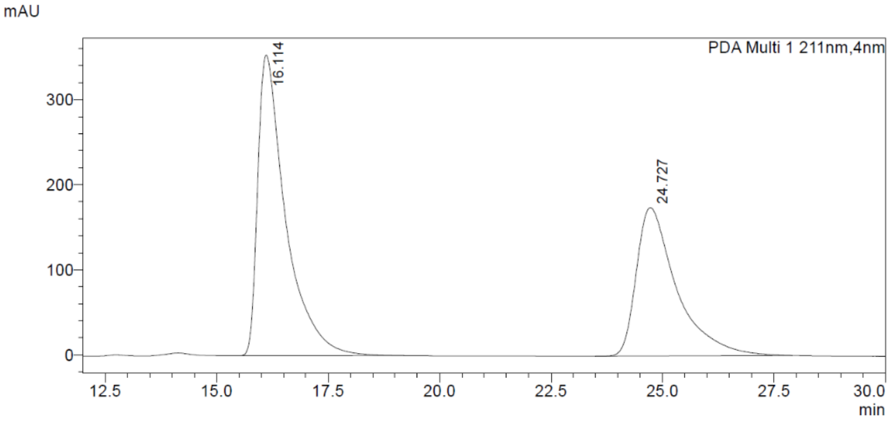


PDA Ch1 211nm

| Peak# | Ret. Time | Area% |
|---|---|---|
| 1 | 16.114 | 58.387 |
| 2 | 24.727 | 41.613 |
| Total | | 100.000 |

**Isopropyl (*S*)-2-((diphenylmethylene)amino)-5-oxo-5-(thiophen-2-yl)-4-(thiophene-2-carbonyl)pentanoate (S51)**

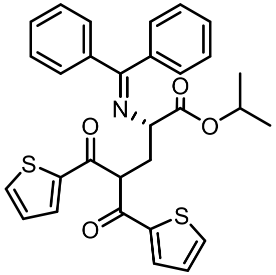


$^{1}H$ NMR, $CD_2Cl_2$, 400 MHz

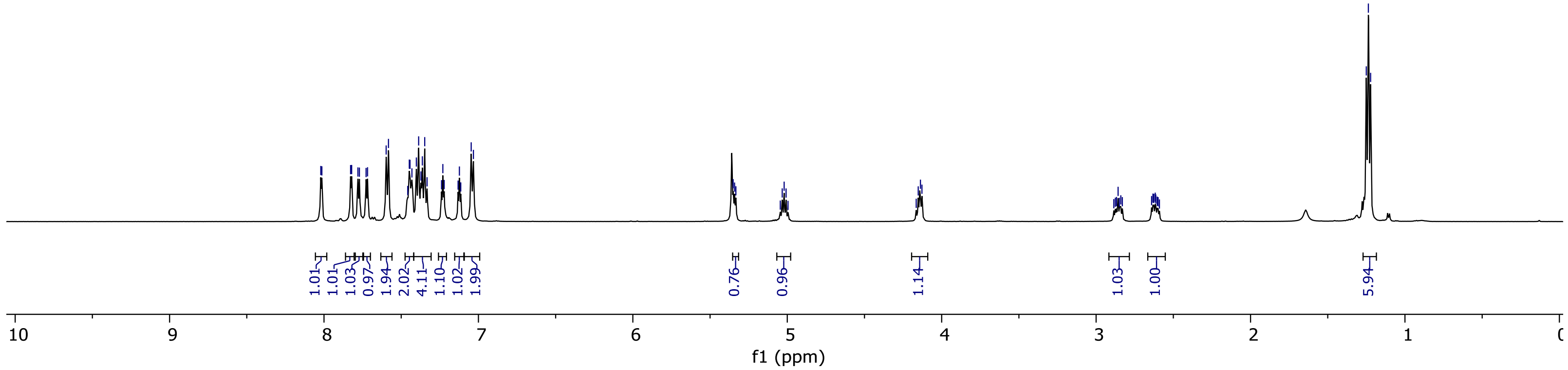

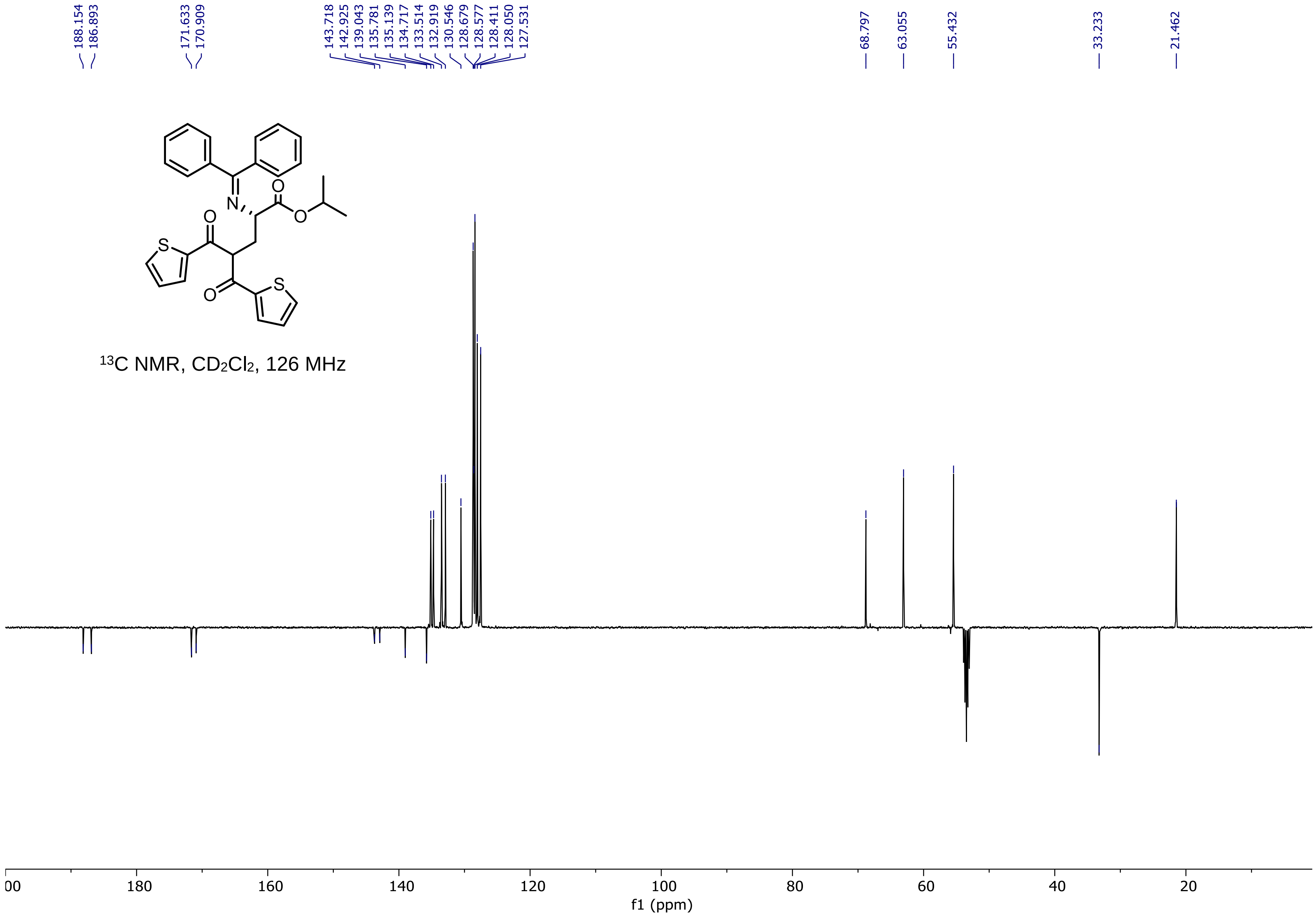
188.154
186.893
171.633
170.909
143.718
142.925
139.043
135.781
135.139
134.717
133.514
132.919
130.546
128.679
128.577
128.411
128.050
127.531
68.797
63.055
55.432
33.233
21.462
O
N
O
S
S
13C NMR, CD2Cl2, 126 MHz
200
180
160
140
120
100
80
60
40
20
f1 (ppm)

HPLC data for **S51**: Chiralpak IB (95:5 Hexane:IPA, flow rate 1 mL/min, 254 nm, 30 °C) $t_R$: 13.2 min, $t_R$: 15.8 min, 34:66 er

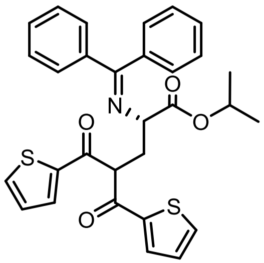

Racemic sample (left), enantioenriched sample (right)

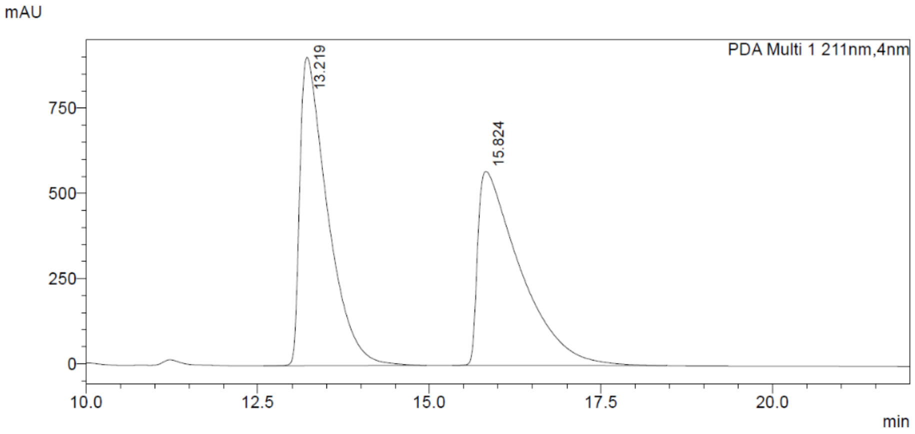


PDA Ch1 211nm

| Peak# | Ret. Time | Area% |
|---|---|---|
| 1 | 13.219 | 50.789 |
| 2 | 15.824 | 49.211 |
| Total | | 100.000 |

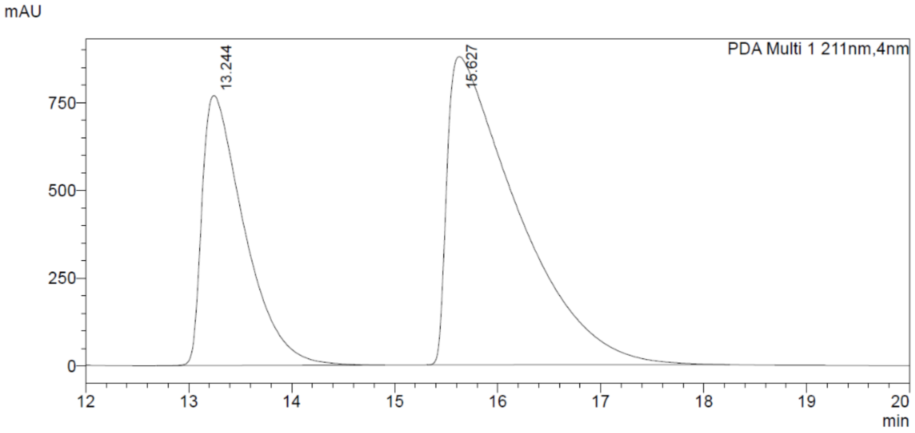


PDA Ch1 211nm

| Peak# | Ret. Time | Area% |
|---|---|---|
| 1 | 13.244 | 34.572 |
| 2 | 15.627 | 65.428 |
| Total | | 100.000 |

**($S$)-2-((Diphenylmethylene)amino)-1-morpholino-5-(thiophen-3-yl)-4-(thiophene-3-carbonyl)pentane-1,5-dione (20o)**

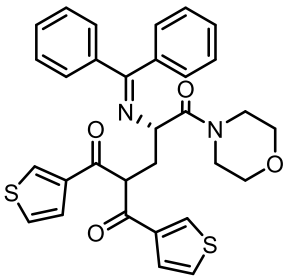

$^{1}$H NMR, $CD_2Cl_2$, 400 MHz

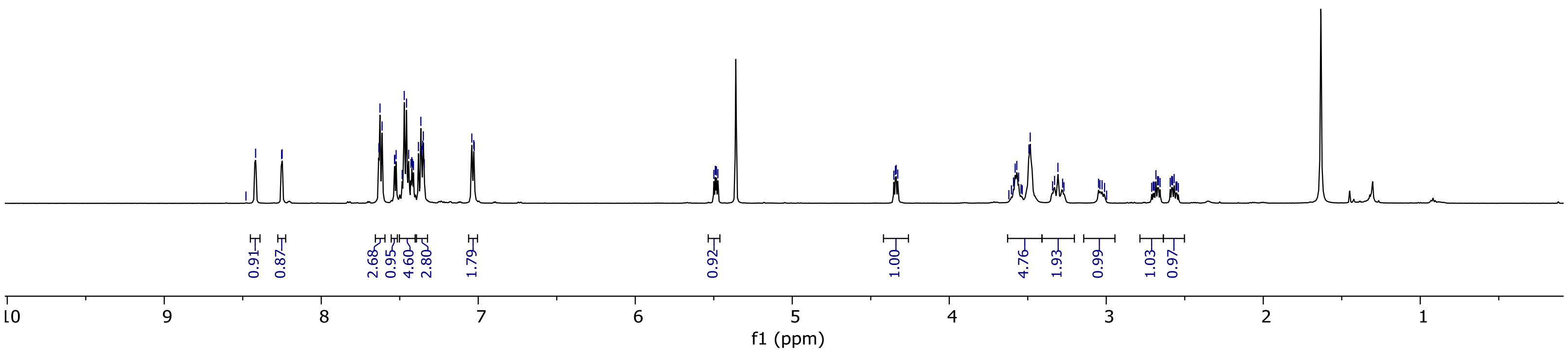

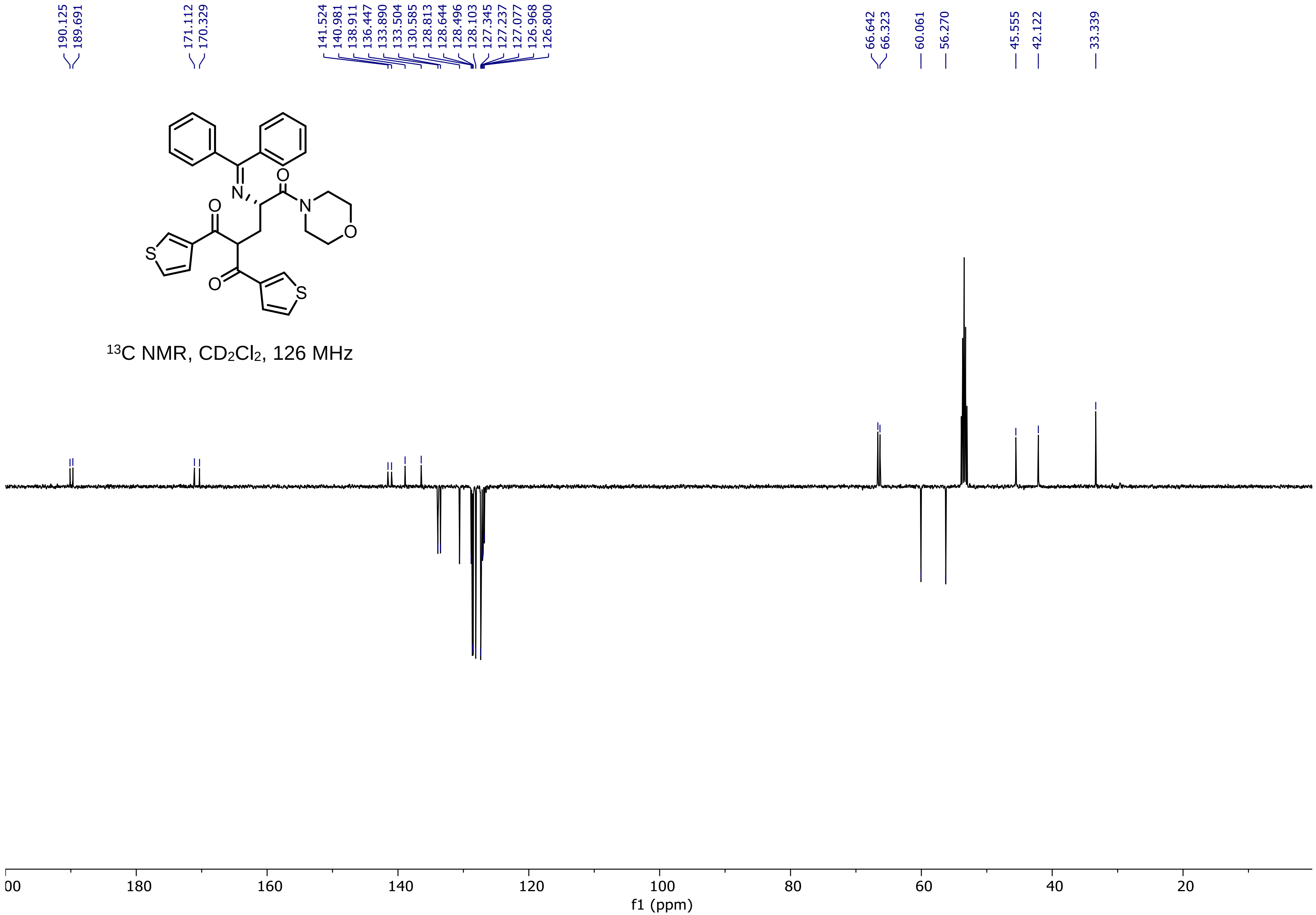
190.125
189.691
171.112
170.329
141.524
140.981
138.911
136.447
133.890
133.504
130.585
128.813
128.644
128.496
128.103
127.345
127.237
127.077
126.968
126.800
66.642
66.323
60.061
56.270
45.555
42.122
33.339
O
N
O
N
O
S
O
S
$^{13}$C NMR, $CD_2Cl_2$, 126 MHz
200
180
160
140
120
100
80
60
40
20
f1 (ppm)

HPLC data for **20o**: Chiralpak IA (90:10 hexane:IPA, flow rate 1 mL/min, 254 nm, 30 °C) $t_R$: 30.8 min, $t_R$: 46.4 min, 85:15 er

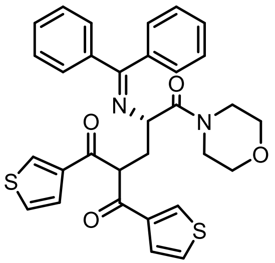

Racemic sample (left), enantioenriched sample (right)

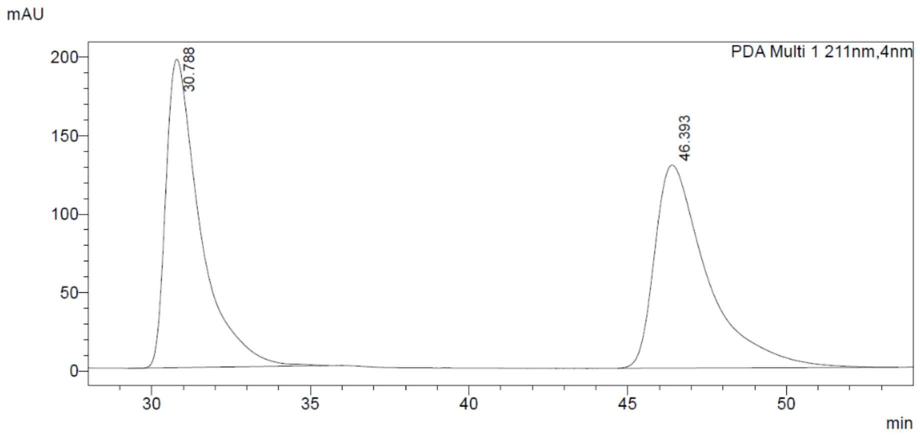


PDA Ch1 211nm

| Peak# | Ret. Time | Area% |
|---|---|---|
| 1 | 30.788 | 49.779 |
| 2 | 46.393 | 50.221 |
| Total | | 100.000 |

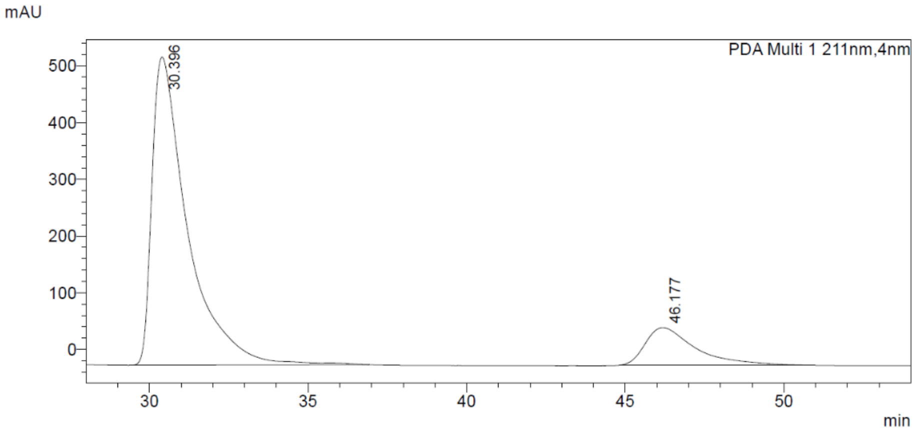


PDA Ch1 211nm

| Peak# | Ret. Time | Area% |
|---|---|---|
| 1 | 30.396 | 85.300 |
| 2 | 46.177 | 14.700 |
| Total | | 100.000 |

**(*S*)-2-((Diphenylmethylene)amino)-4-(furan-2-carbonyl)-5-(furan-2-yl)-1-morpholinopentane-1,5-dione (20p)**

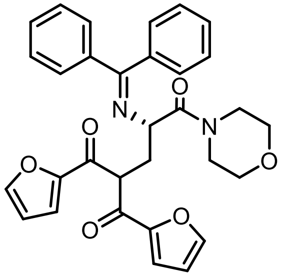

$^{1}$H NMR, $CD_2Cl_2$, 400 MHz

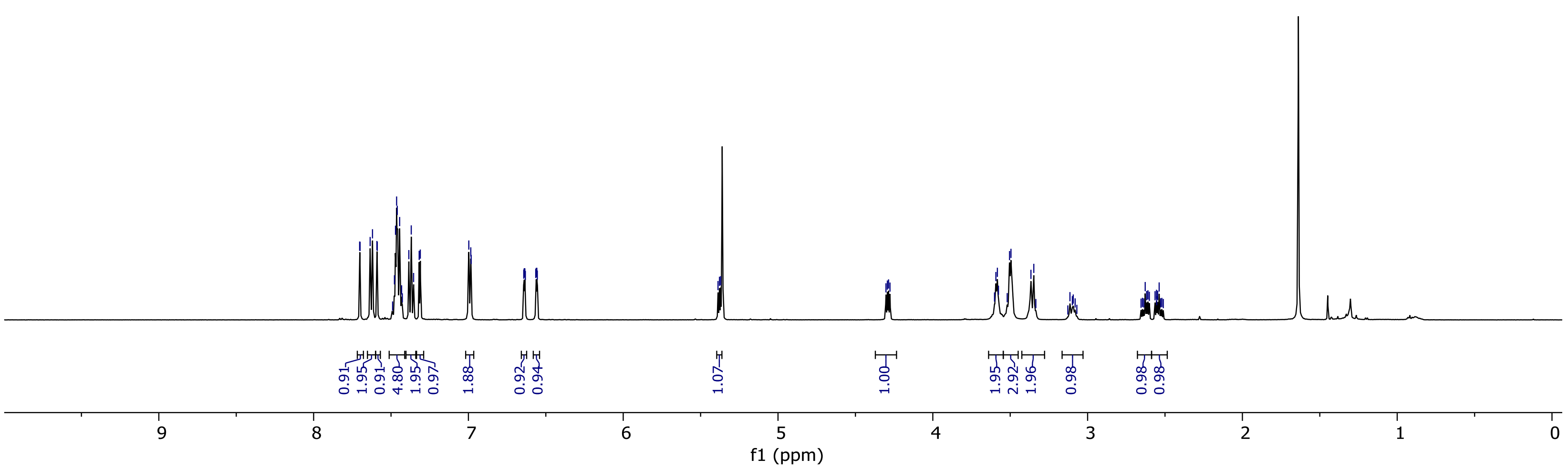

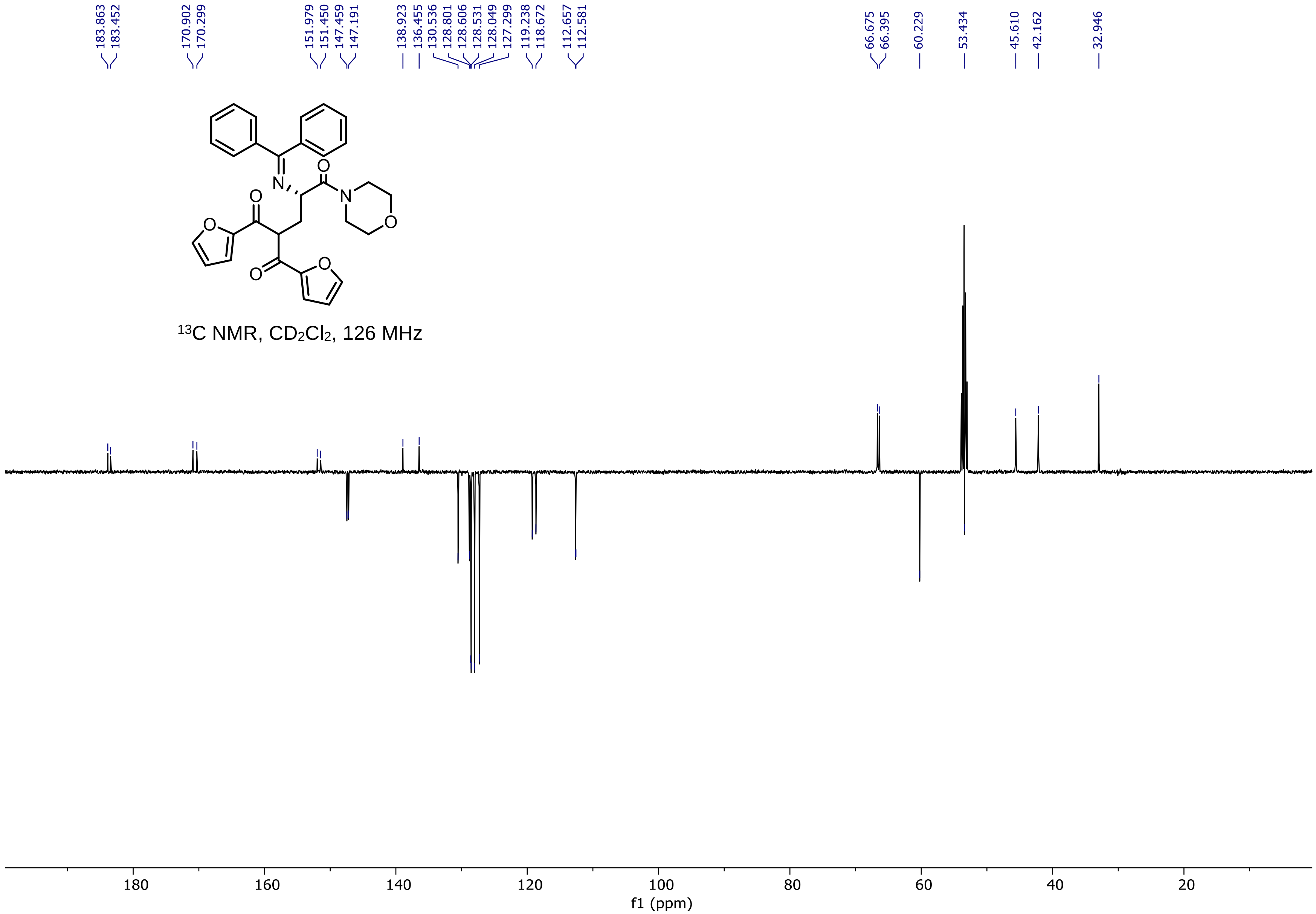
183.863
183.452
170.902
170.299
151.979
151.450
147.459
147.191
138.923
136.455
130.536
128.801
128.606
128.531
128.049
127.299
119.238
118.672
112.657
112.581
66.675
66.395
60.229
53.434
45.610
42.162
32.946
$^{13}C$ NMR, $CD_2Cl_2$, 126 MHz
180
160
140
120
100
80
60
40
20
f1 (ppm)

HPLC data for **20p**: Chiralpak IB (80:20 hexane : IPA, flow rate 1 mL/min, 254 nm, 30 °C) $t_R$: 19.6 min, $t_R$: 26.9 min, 16:84 er

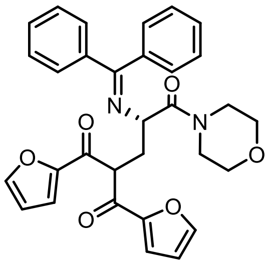

Racemic sample (left), enantioenriched sample (right)

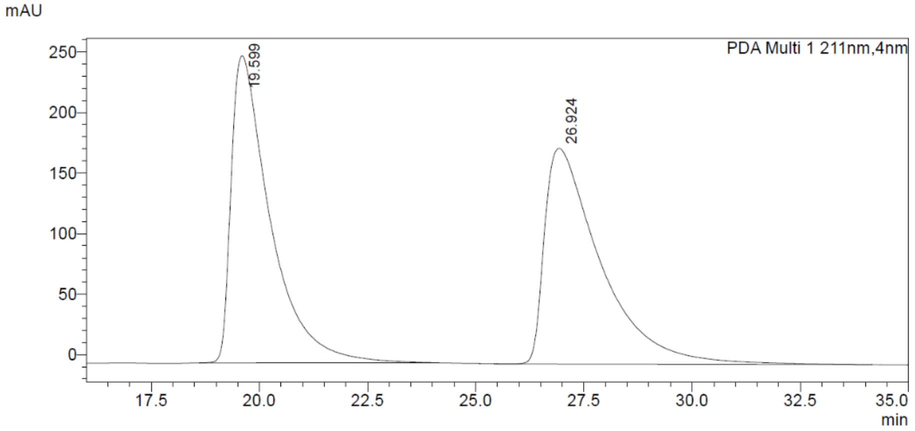


PDA Ch1 211nm

| Peak# | Ret. Time | Area% |
|---|---|---|
| 1 | 19.599 | 49.811 |
| 2 | 26.924 | 50.189 |
| Total | | 100.000 |

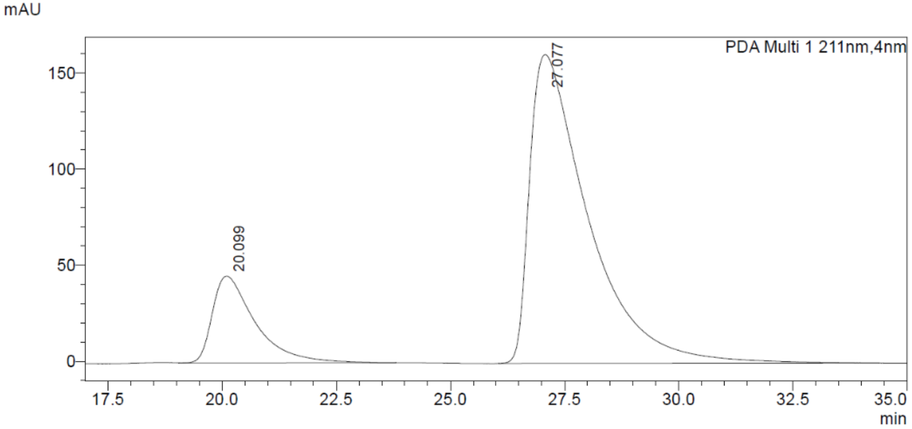


PDA Ch1 211nm

| Peak# | Ret. Time | Area% |
|---|---|---|
| 1 | 20.099 | 16.965 |
| 2 | 27.077 | 83.035 |
| Total | | 100.000 |

**(*S*)-1-(Benzo[d][1,3]dioxol-5-yl)-2-(benzo[d][1,3]dioxole-5-carbonyl)-4-((diphenylmethylene)amino)-5-morpholinopentane-1,5-dione (20q)**

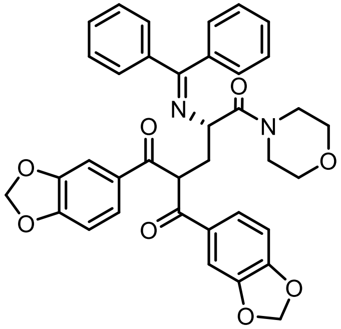


$^{1}H$ NMR, $CD_2Cl_2$, 400 MHz

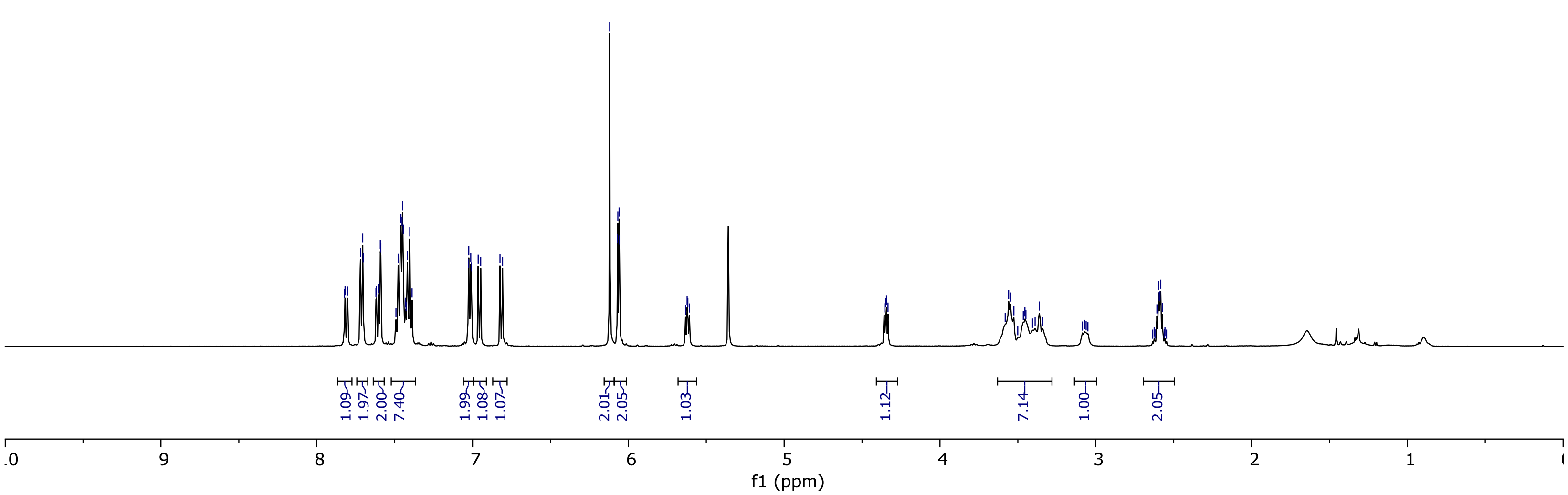

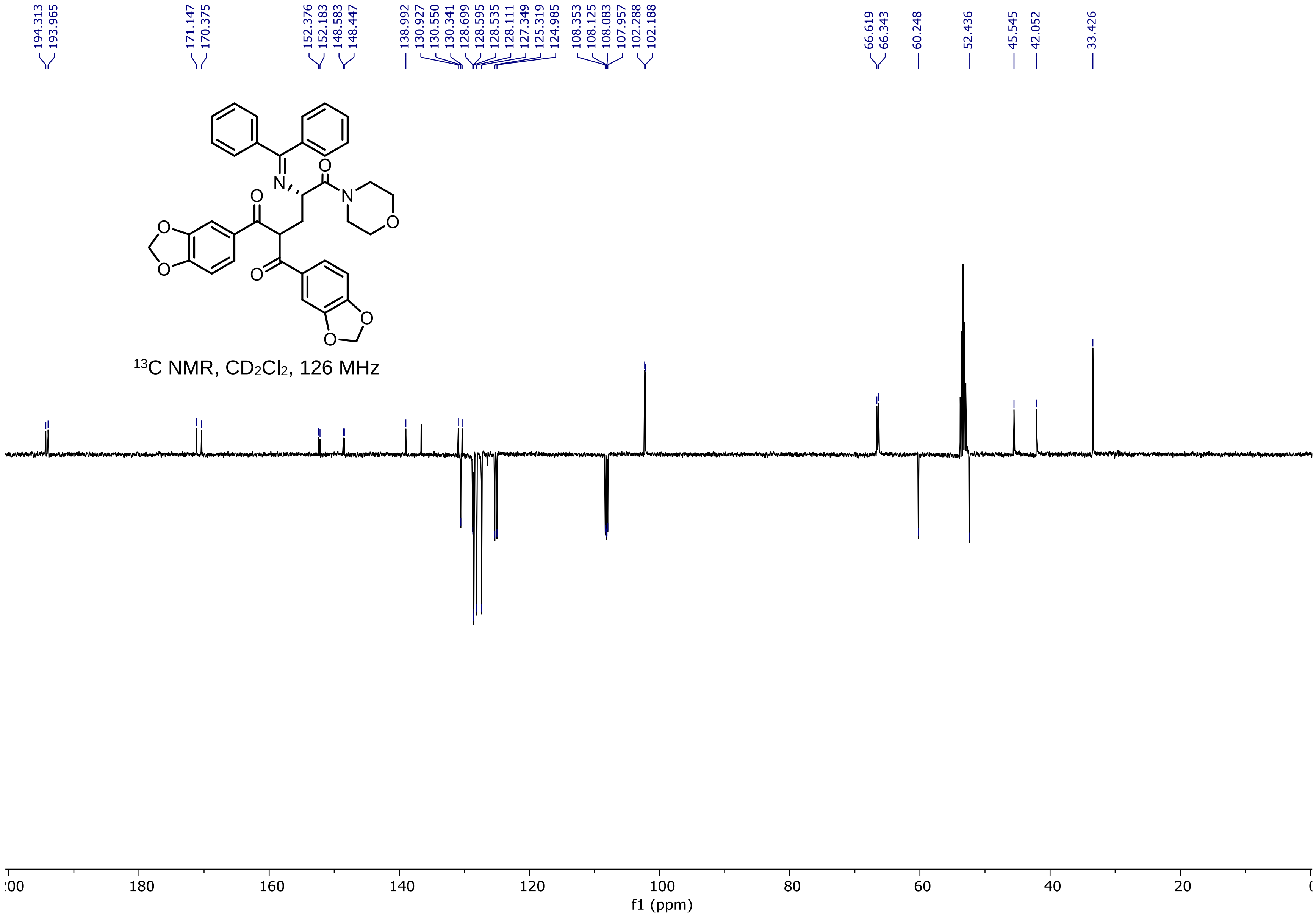


$^{13}C$ NMR, $CD_2Cl_2$, 126 MHz

HPLC data for **20q**: Chiralpak IA (80:20 hexane:IPA, flow rate 1 mL/min, 254 nm, 30 °C) $t_R$: 32.6 min, $t_R$: 48.6 min, 90:10 er

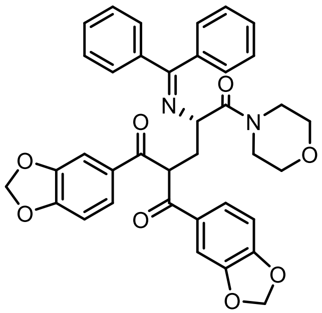

Racemic sample (left), enantioenriched sample (right)

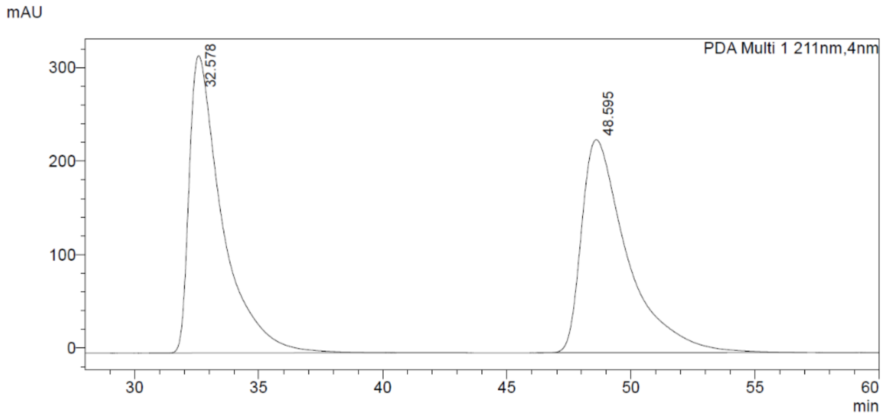


PDA Ch1 211nm

| Peak# | Ret. Time | Area% |
|---|---|---|
| 1 | 32.578 | 50.078 |
| 2 | 48.595 | 49.922 |
| Total | | 100.000 |

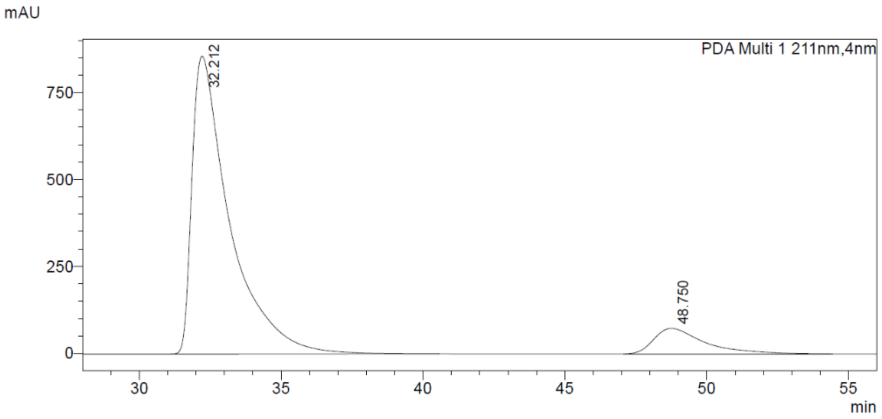


PDA Ch1 211nm

| Peak# | Ret. Time | Area% |
|---|---|---|
| 1 | 32.212 | 89.680 |
| 2 | 48.750 | 10.320 |
| Total | | 100.000 |

**(*S*)-2-Amino-1-morpholino-4,4-bis(phenylsulfonyl)butan-1-one (21)**

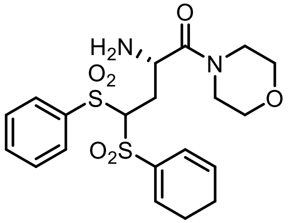


$^1H$ NMR, $CDCl_3$, 400 MHz

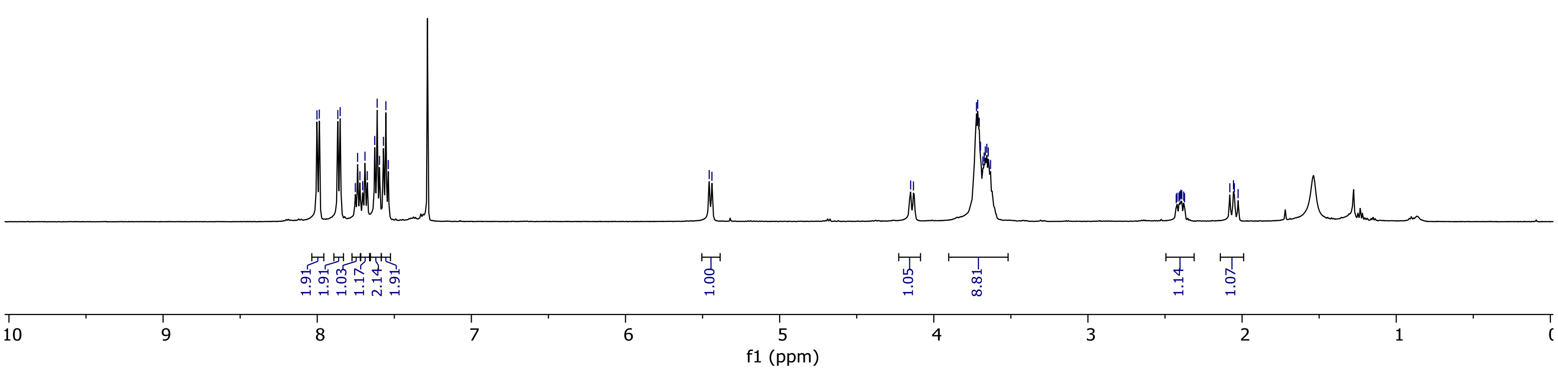

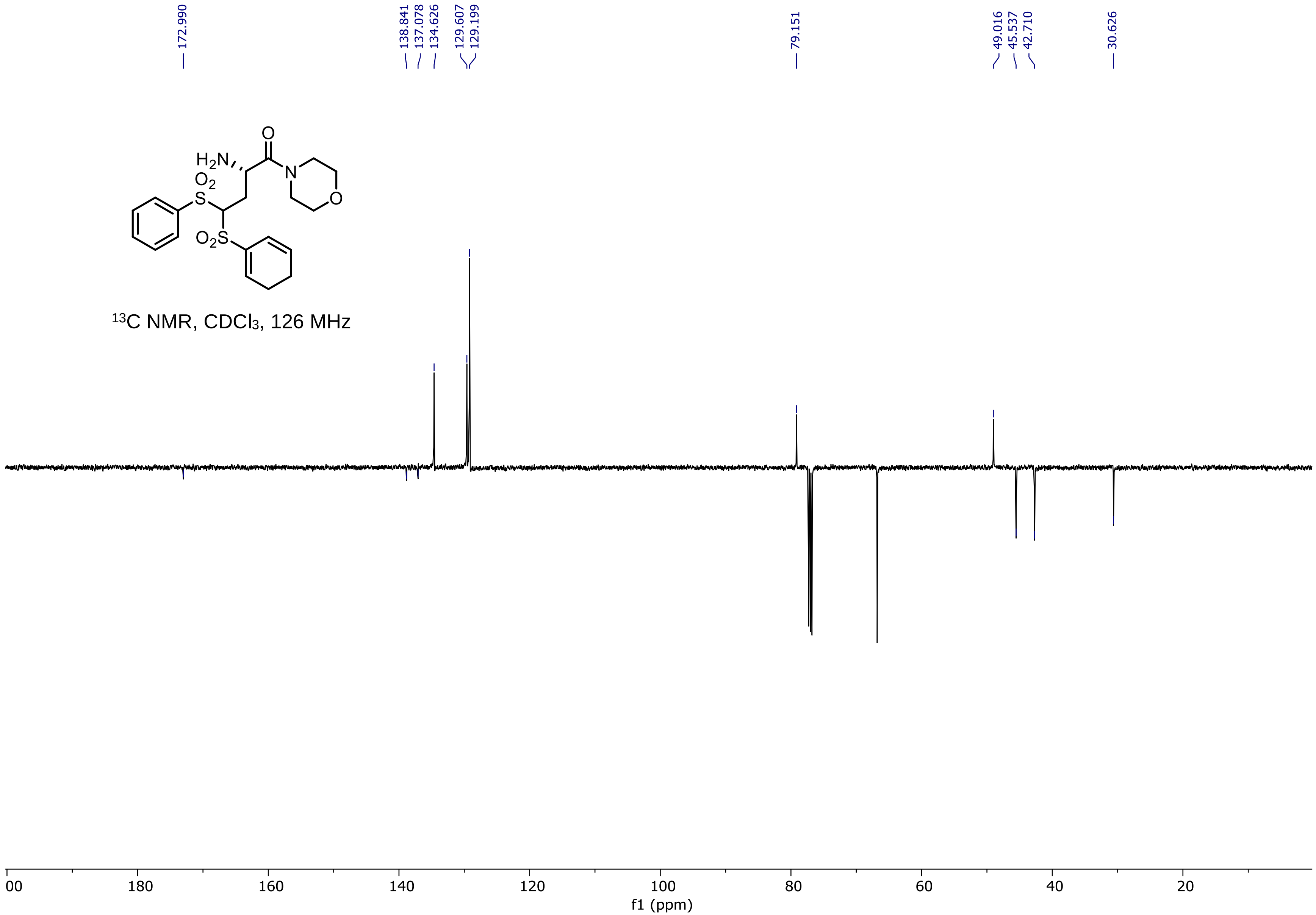
172.990
138.841
137.078
134.626
129.607
129.199
79.151
49.016
45.537
42.710
30.626
H2N
O2
S
O
N
O
O2S
13C NMR, CDCl3, 126 MHz
00
180
160
140
120
100
80
60
40
20
f1 (ppm)

HPLC data for **21**: Chiralpak AD-H (80:20 hexane:IPA, flow rate 2 mL/min, 254 nm, 30 °C) $t_R$: 47.6 min, $t_R$: 60.1 min, 97:3 er

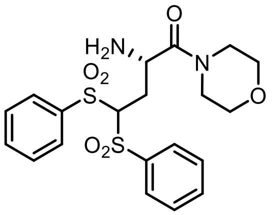

Racemic sample (left), enantioenriched sample (right)

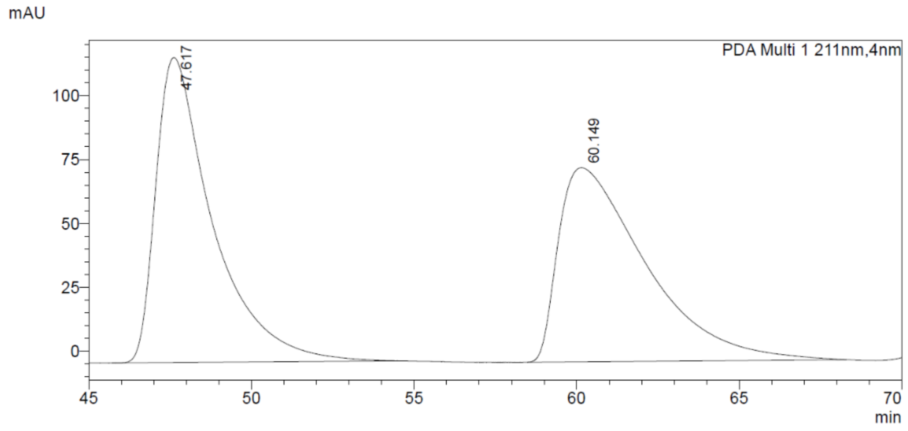


PDA Ch1 211nm

| Peak# | Ret. Time | Area% |
|---|---|---|
| 1 | 47.617 | 50.513 |
| 2 | 60.149 | 49.487 |
| Total | | 100.000 |

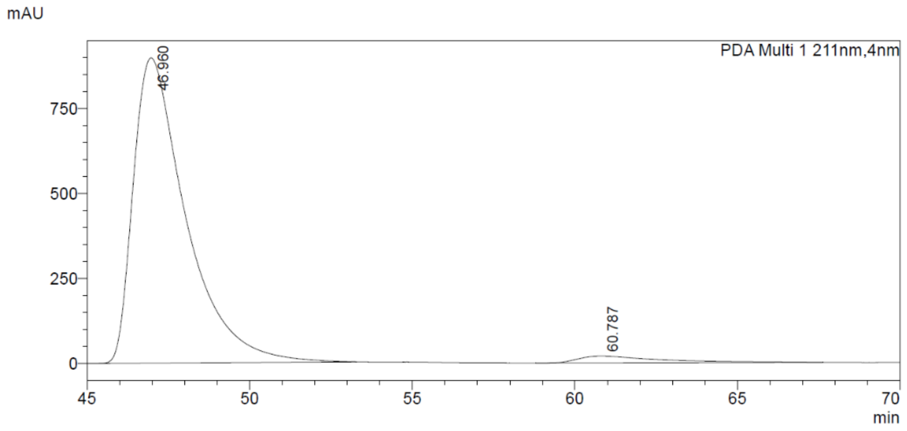


PDA Ch1 211nm

| Peak# | Ret. Time | Area% |
|---|---|---|
| 1 | 46.960 | 96.714 |
| 2 | 60.787 | 3.286 |
| Total | | 100.000 |

## (*S*)-4-Bromo-*N*-(1-morpholino-1-oxo-4,4-bis(phenylsulfonyl)butan-2-yl)benzenesulfonamide (22)

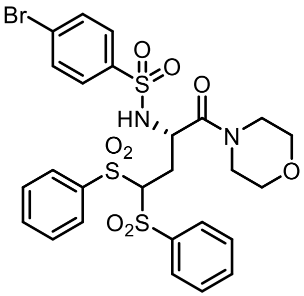


$^1$H NMR, $CDCl_3$, 400 MHz

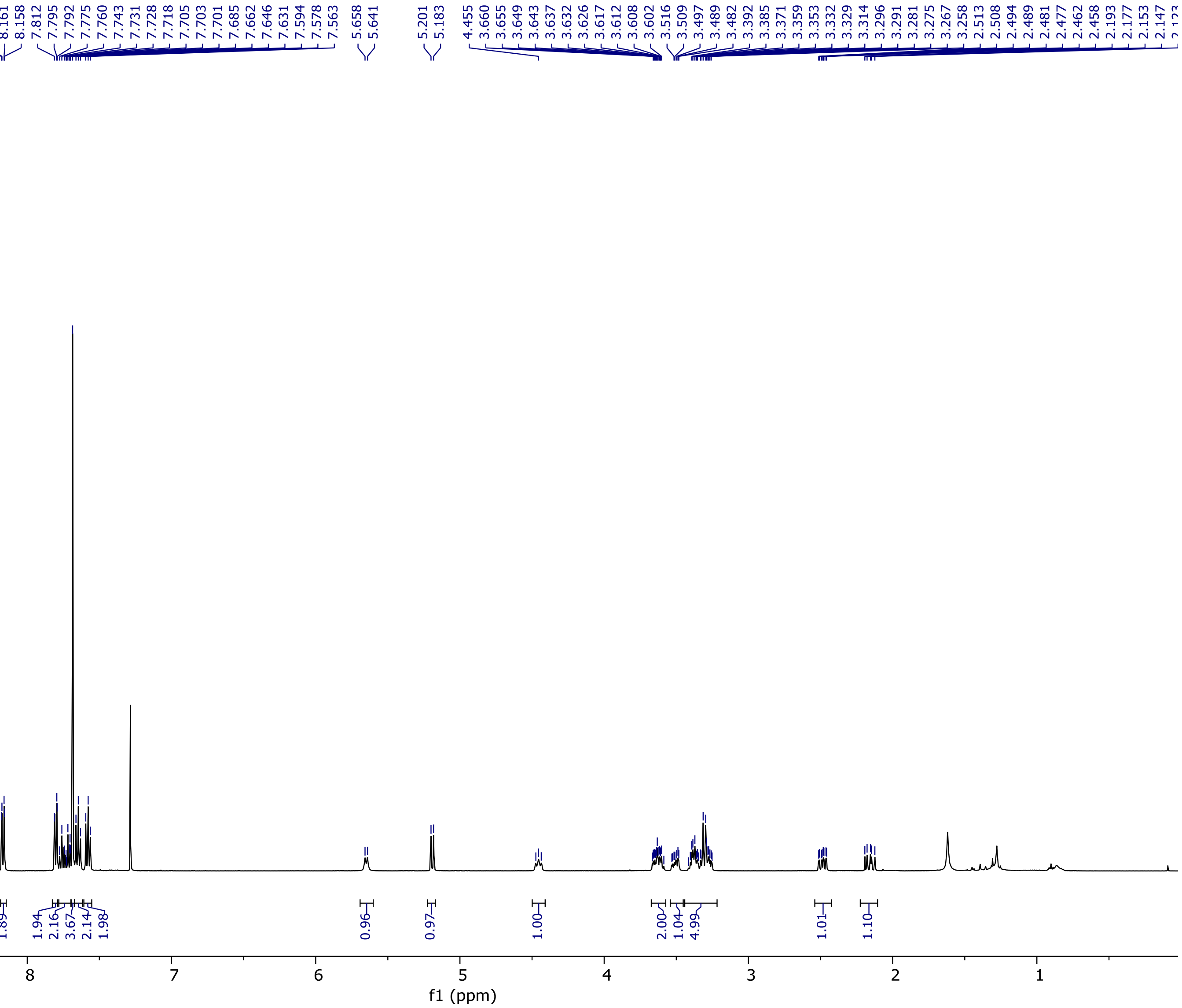

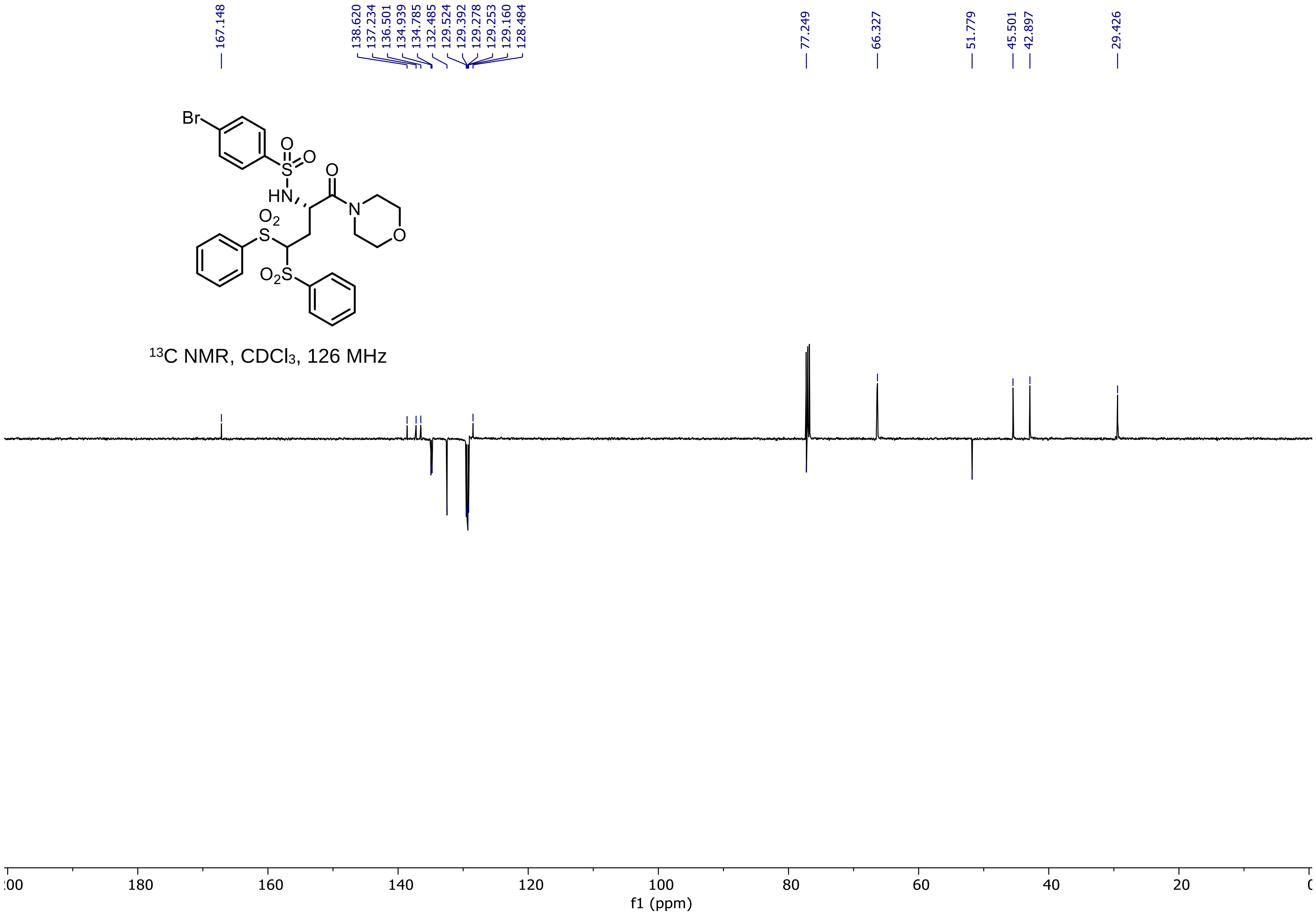
167.148
138.620
137.234
136.501
134.939
134.785
132.485
129.524
129.392
129.278
129.253
129.160
128.484
77.249
66.327
51.779
45.501
42.897
29.426
Br
HN
O₂
O₂S
O
N
13C NMR, CDCl3, 126 MHz
200
180
160
140
120
100
80
60
40
20
0
f1 (ppm)

HPLC data for **22**: Chiralpak AS-H (80:20 hexane:IPA, flow rate 1 mL/min, 254 nm, 30 °C) $t_R$: 35.6 min, $t_R$: 48.6 min, 8:92 er

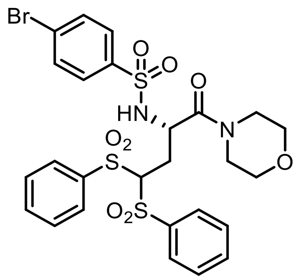


Racemic sample (left), enantioenriched sample (right)

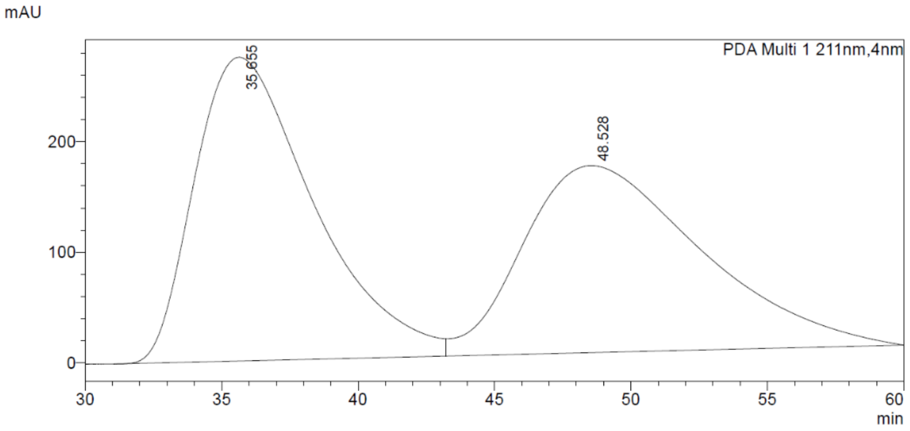


| PDA Ch1 211nm | | |
|---|---|---|
| Peak# | Ret. Time | Area% |
| 1 | 35.655 | 51.968 |
| 2 | 48.528 | 48.032 |
| Total | | 100.000 |

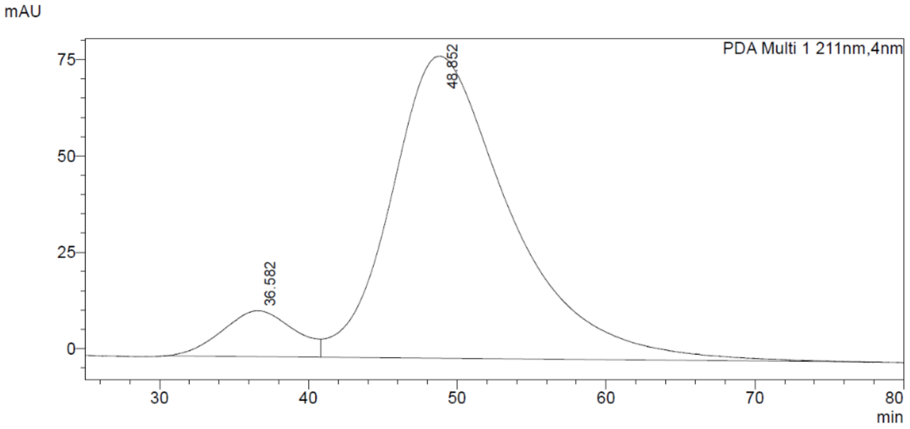


| PDA Ch1 211nm | | |
|---|---|---|
| Peak# | Ret. Time | Area% |
| 1 | 36.582 | 8.454 |
| 2 | 48.852 | 91.546 |
| Total | | 100.000 |

**Di-tert-butyl (*S*)-2-(2-amino-3-morpholino-3-oxopropyl)malonate (23)**

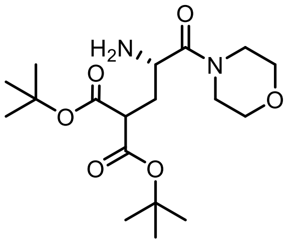

$^1$H NMR, $CDCl_3$, 400 MHz

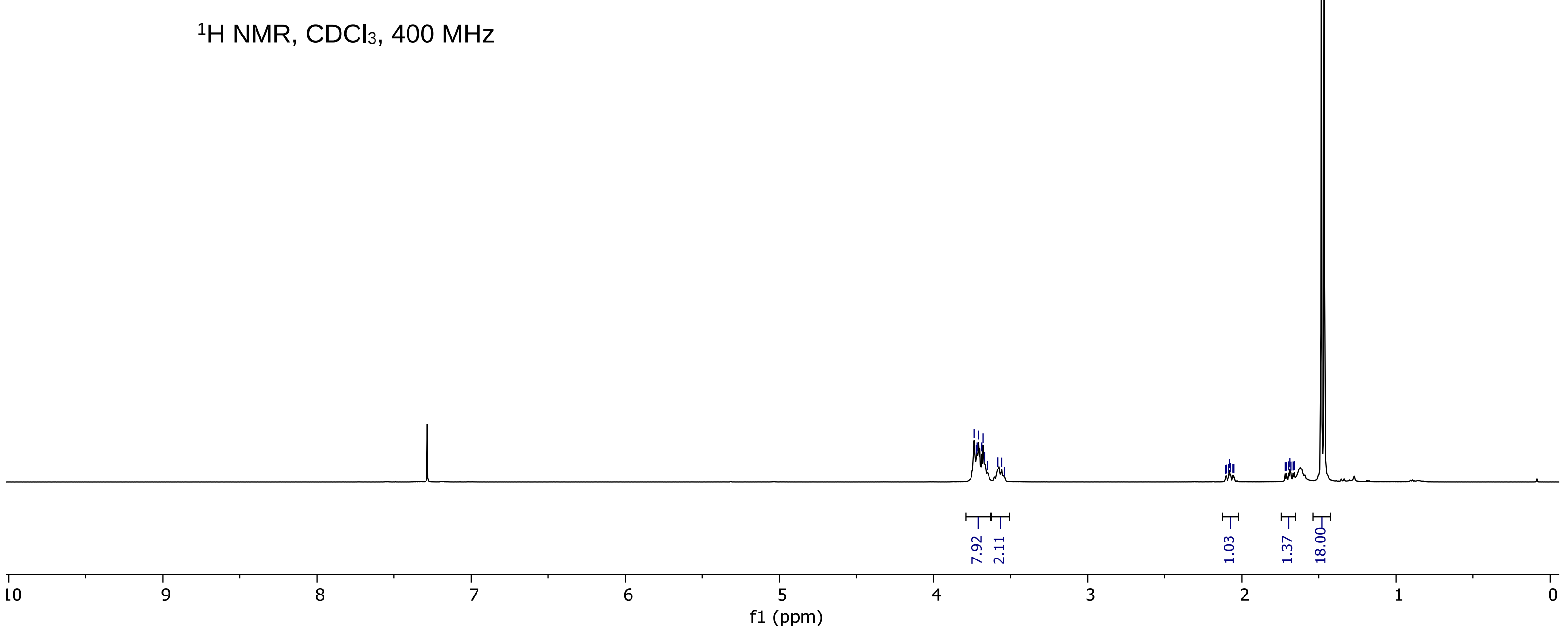

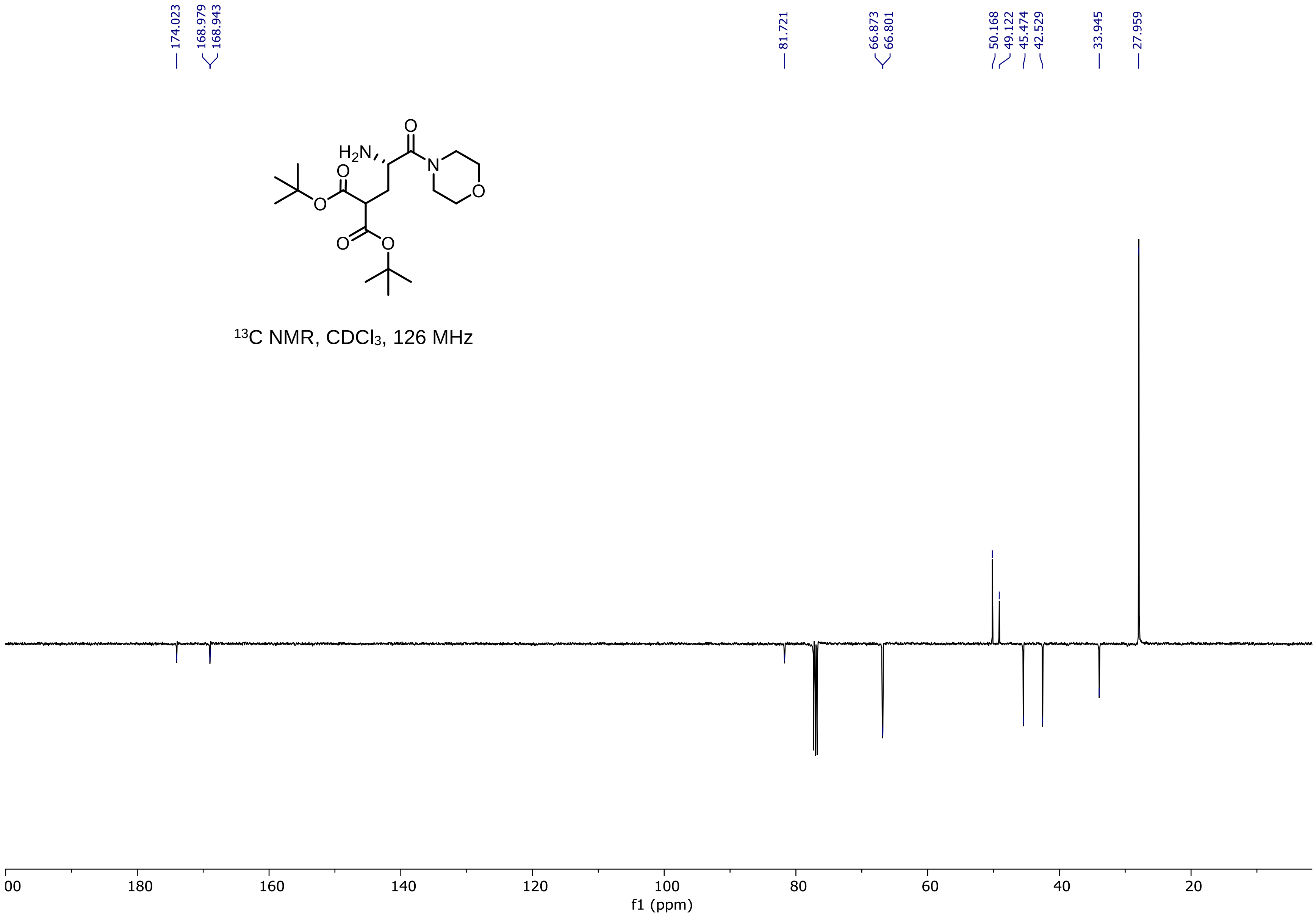
174.023
168.979
168.943
81.721
66.873
66.801
50.168
49.122
45.474
42.529
33.945
27.959
H2N
O
N
$^{13}$C NMR, $CDCl_3$, 126 MHz
00
180
160
140
120
100
80
60
40
20
f1 (ppm)

HPLC data for **23**: Chiralpak IA (95:5 hexane:IPA, flow rate 1 mL/min, 254 nm, 30 °C) $t_R$: 14.7 min, $t_R$: 17.3 min, 96:4 er

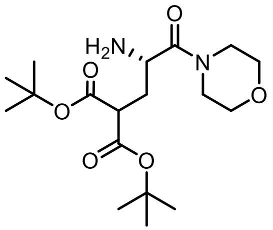


Racemic sample (left), enantioenriched sample (right)

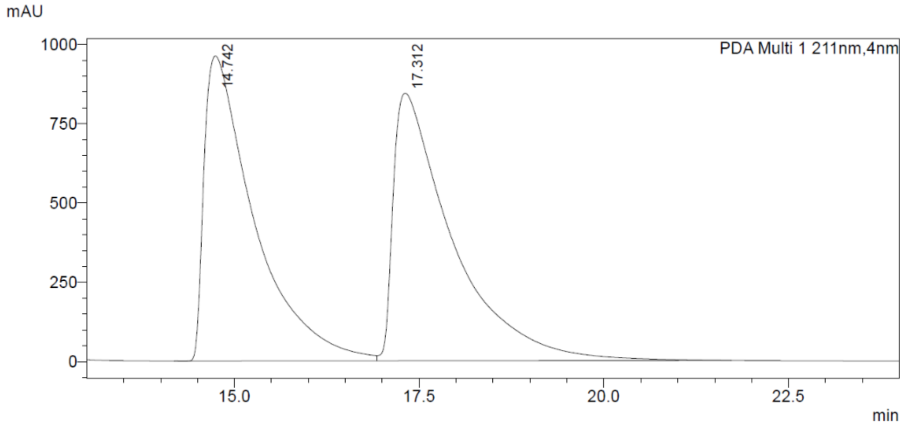


PDA Ch1 211nm

| Peak# | Ret. Time | Area% |
|---|---|---|
| 1 | 14.742 | 49.125 |
| 2 | 17.312 | 50.875 |
| Total | | 100.000 |

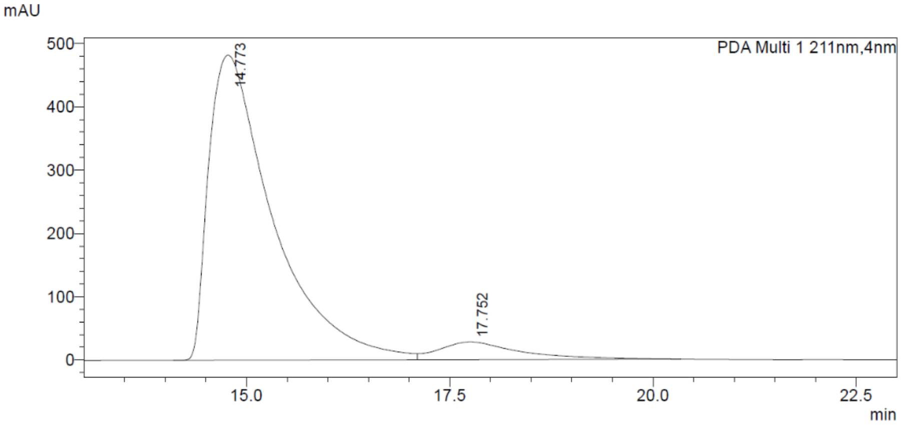


PDA Ch1 211nm

| Peak# | Ret. Time | Area% |
|---|---|---|
| 1 | 14.773 | 93.231 |
| 2 | 17.752 | 6.769 |
| Total | | 100.000 |

**(*S*)-(5-(morpholine-4-carbonyl)-2-phenyl-4,5-dihydro-1H-pyrrole-1,3-diyl)bis(phenylmethanone) (24)**

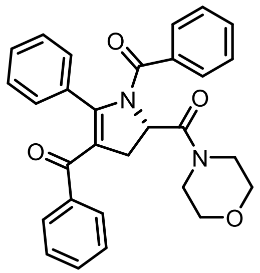


$^{1}$H NMR, $CDCl_3$, 400 MHz

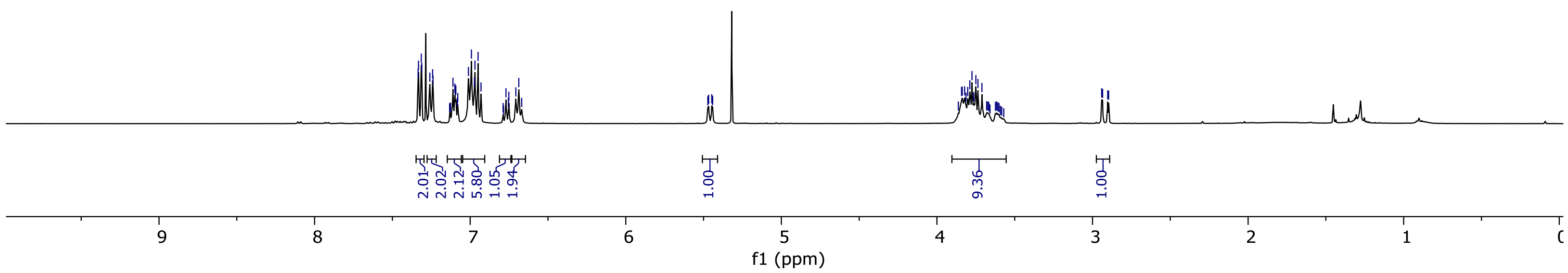

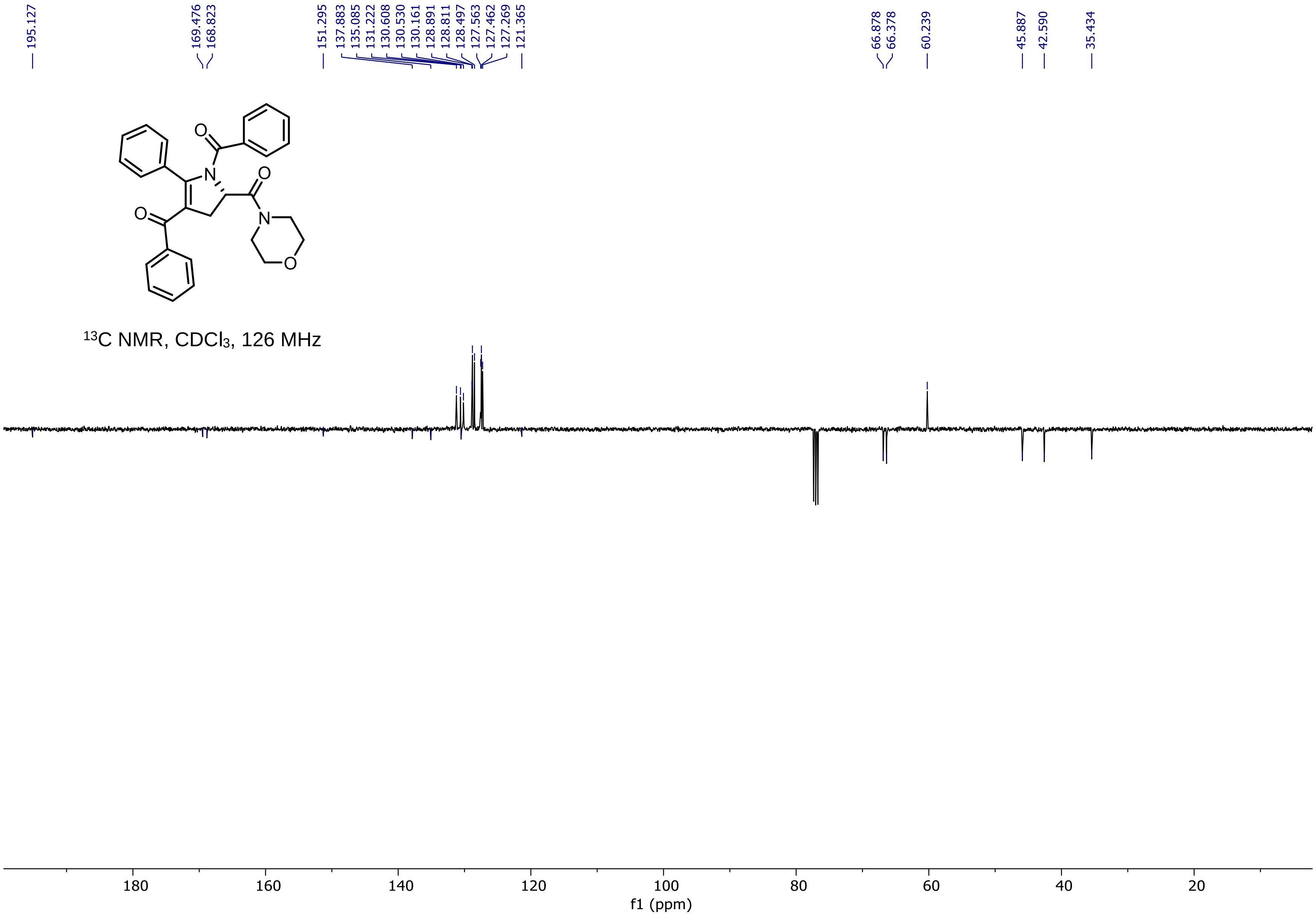
195.127
169.476
168.823
151.295
137.883
135.085
131.222
130.608
130.530
130.161
128.891
128.811
128.497
127.563
127.462
127.269
121.365
66.878
66.378
60.239
45.887
42.590
35.434
O
N
O
O
N
O
$^{13}$C NMR, CDCl$_3$, 126 MHz
180
160
140
120
100
80
60
40
20
f1 (ppm)

HPLC data for **24**: Chiralpak IA (80:20 hexane:IPA, flow rate 2 mL/min, 254 nm, 30 °C) $t_R$: 23.1 min, $t_R$: 47.7 min, 91:9 er

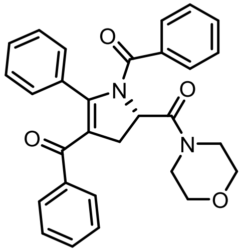

Racemic sample (left), enantioenriched sample (right)

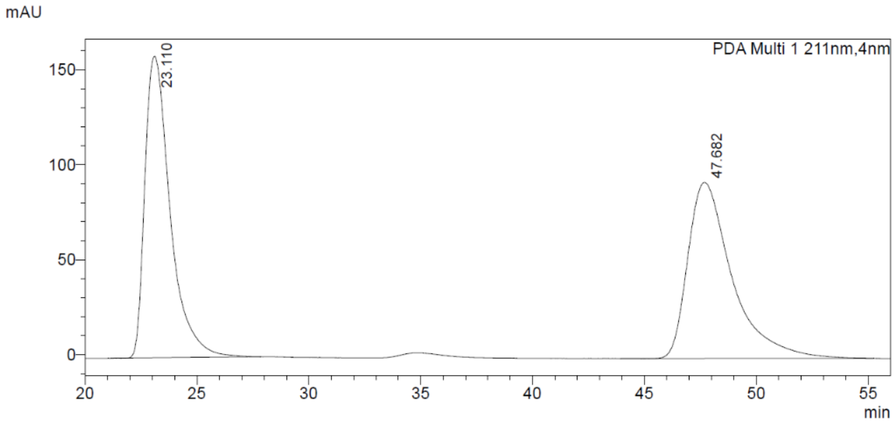


PDA Ch1 211nm

| Peak# | Ret. Time | Area% |
|---|---|---|
| 1 | 23.110 | 49.514 |
| 2 | 47.682 | 50.486 |
| Total | | 100.000 |

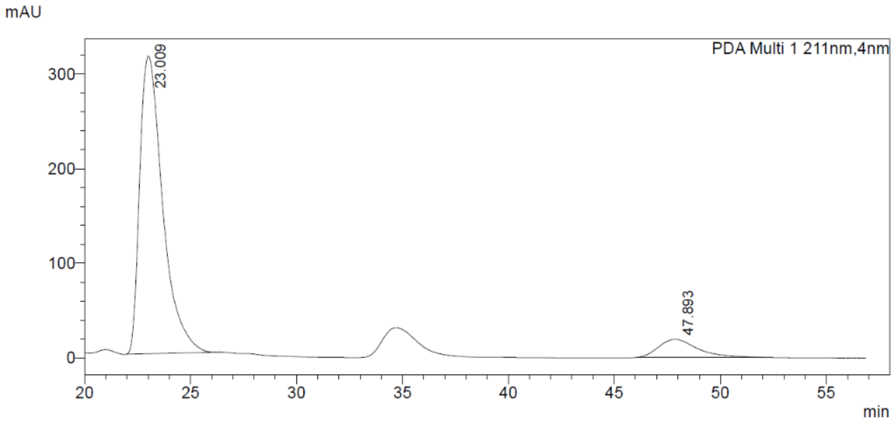


PDA Ch1 211nm

| Peak# | Ret. Time | Area% |
|---|---|---|
| 1 | 23.009 | 90.658 |
| 2 | 47.893 | 9.342 |
| Total | | 100.000 |

**4-Nitrophenyl (*S*)-2-((diphenylmethylene)amino)-4,4-bis(phenylsulfonyl)butanoate (S52)**

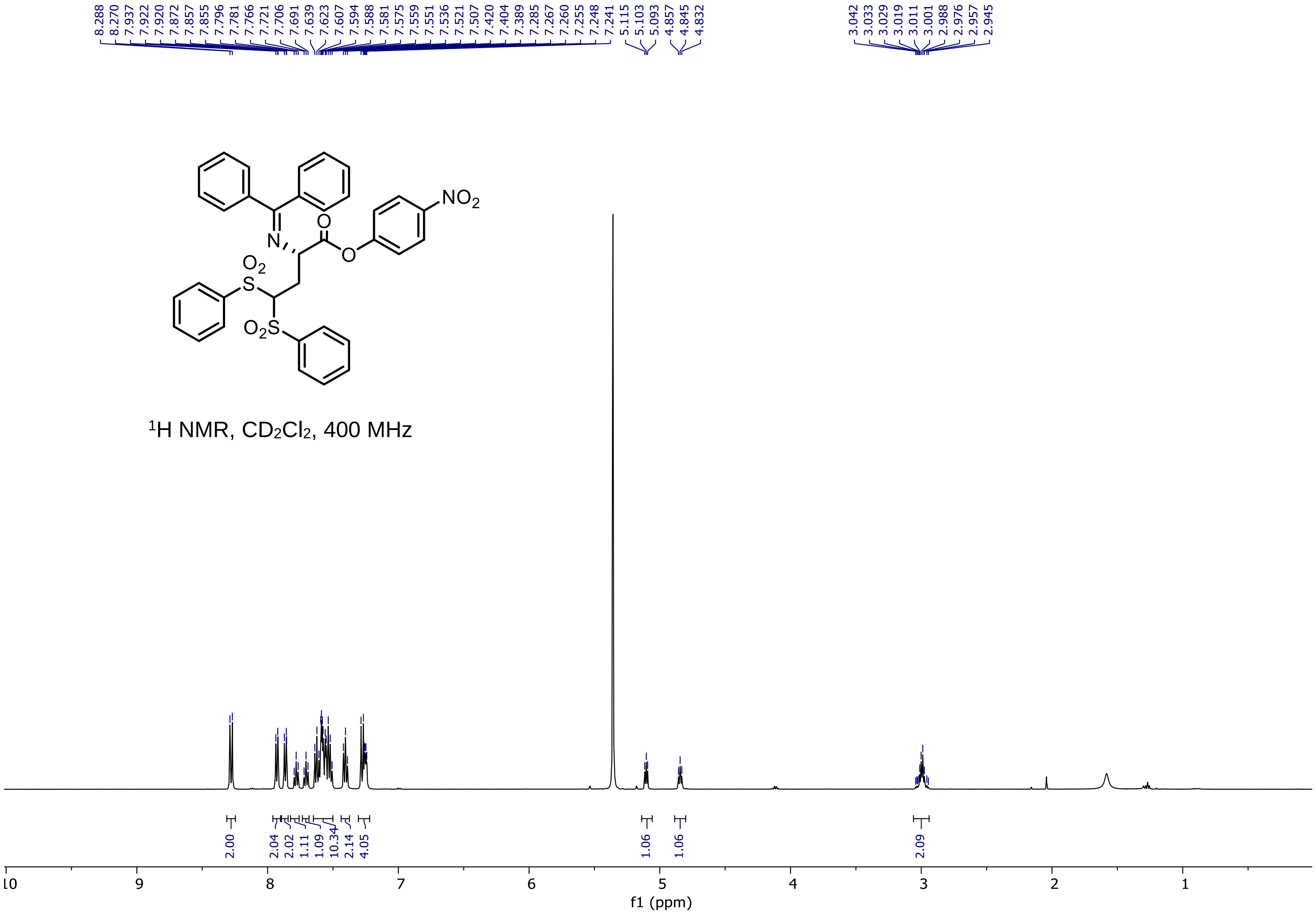

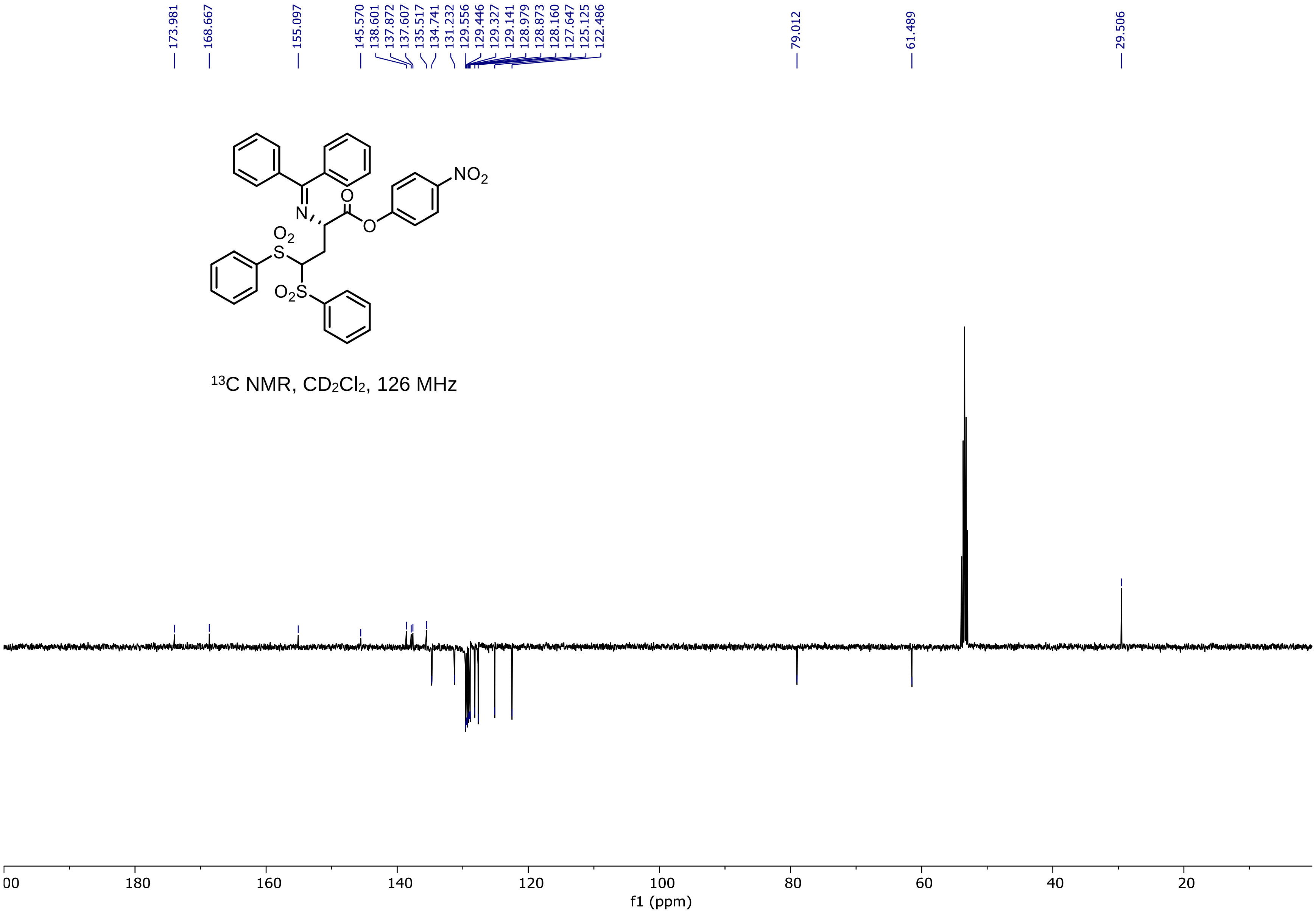
173.981
168.667
155.097
145.570
138.601
137.872
137.607
135.517
134.741
131.232
129.556
129.446
129.327
129.141
128.979
128.873
128.160
127.647
125.125
122.486
79.012
61.489
29.506
NO2
O
N
O
O2
S
O2S
13C NMR, CD2Cl2, 126 MHz
00
180
160
140
120
100
80
60
40
20
f1 (ppm)

## Hydrochloride Salt 5

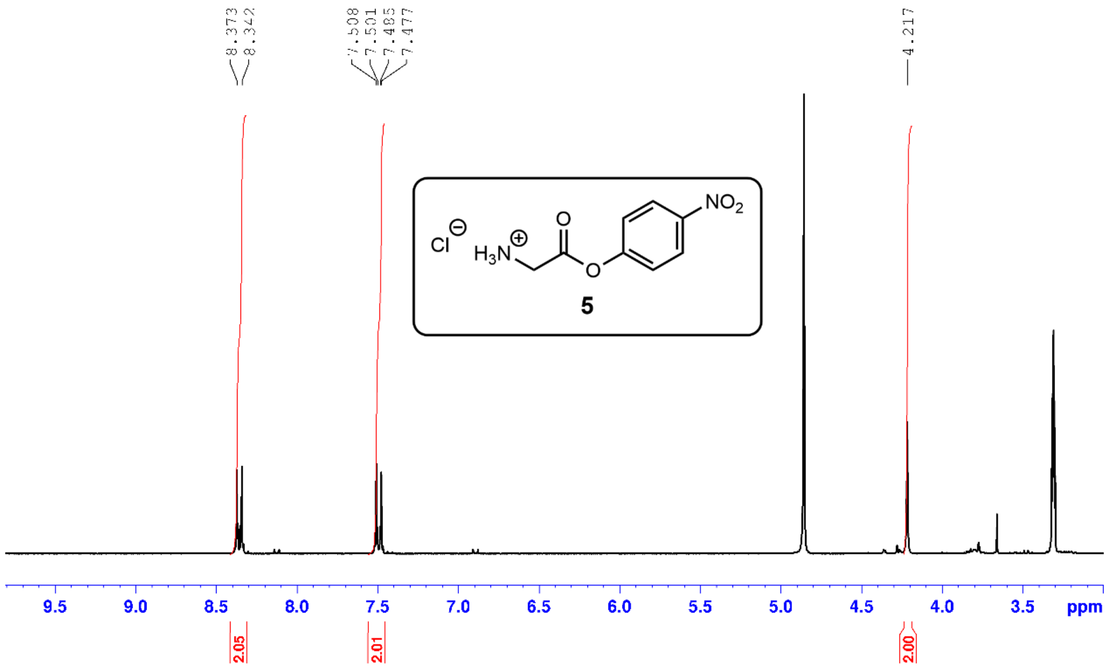


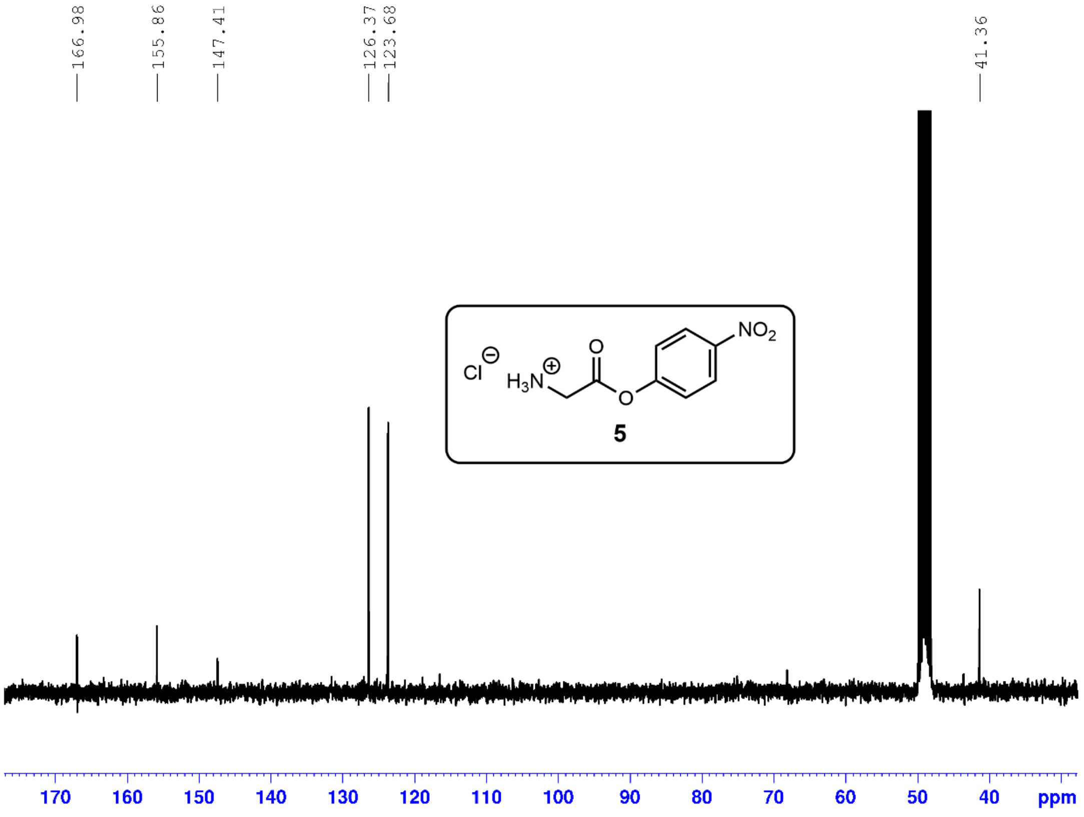

**Glycine Schiff Base Aryl Ester 1**

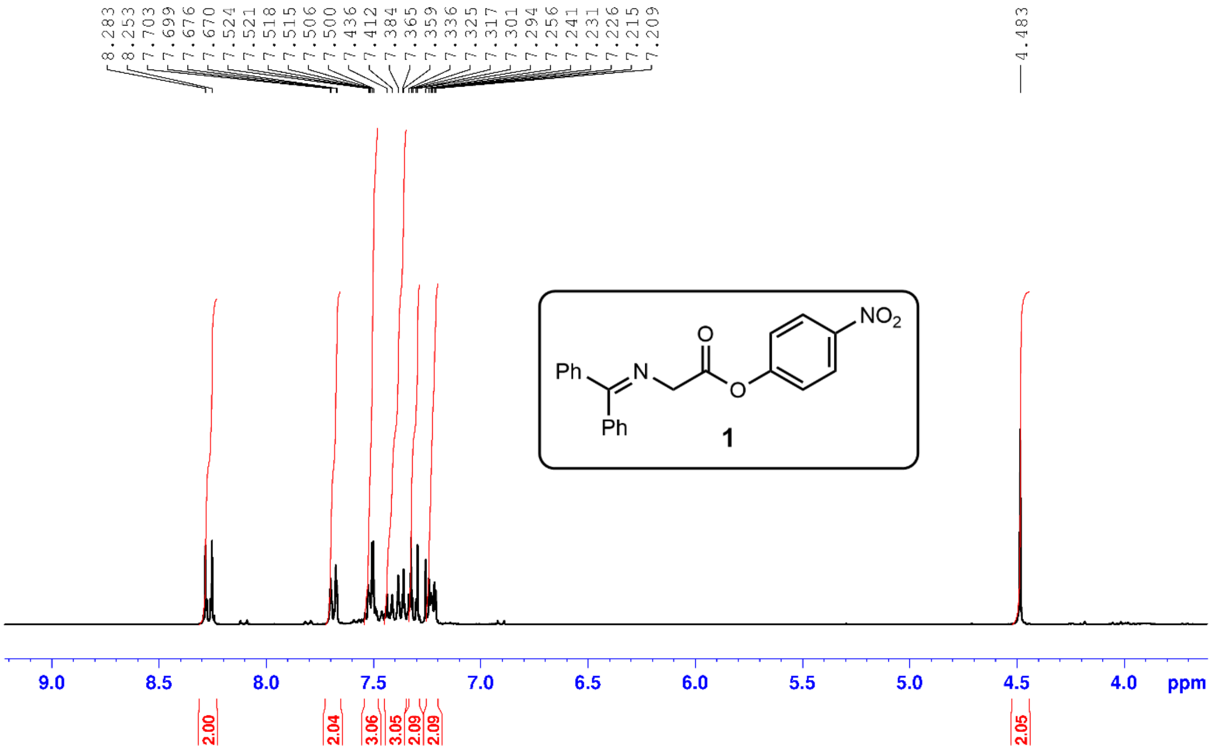


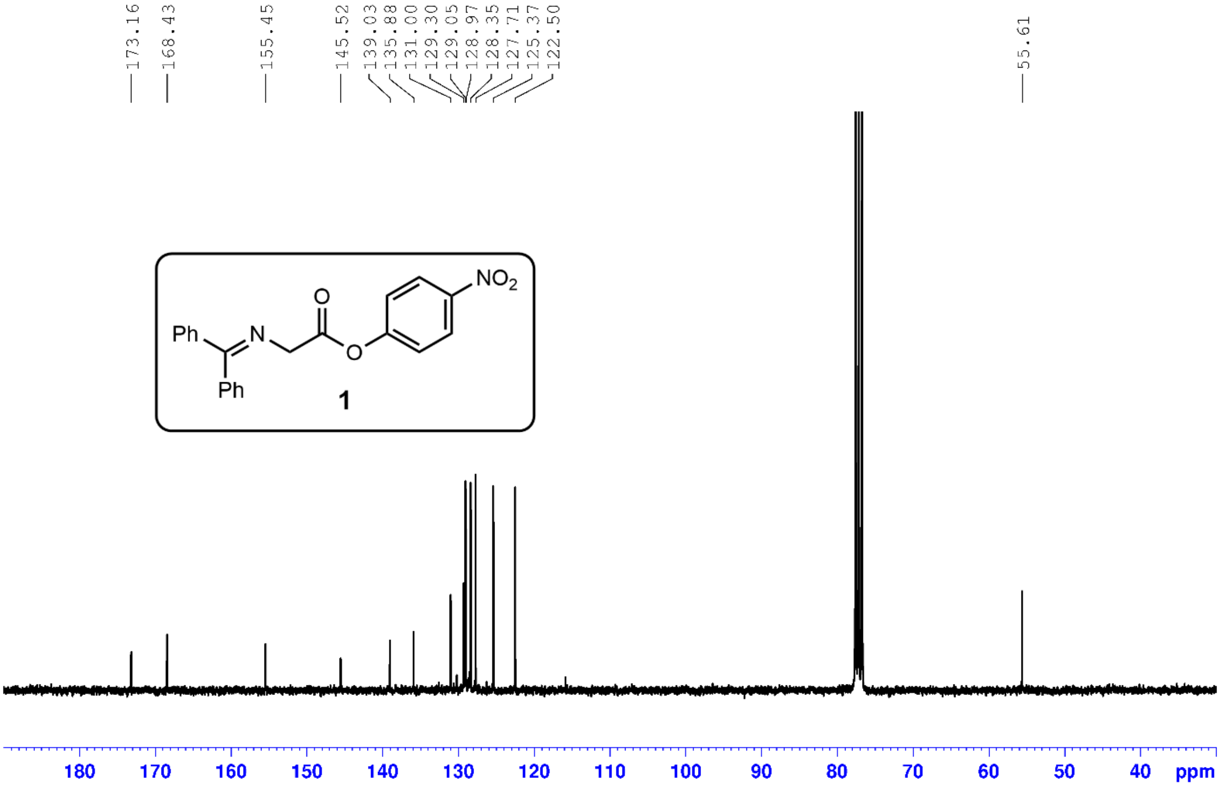

**2,6-di-tert-butyl-4-(2-(methoxymethoxy)benzylidene)cyclohexa-2,5-dien-1-one 13b**

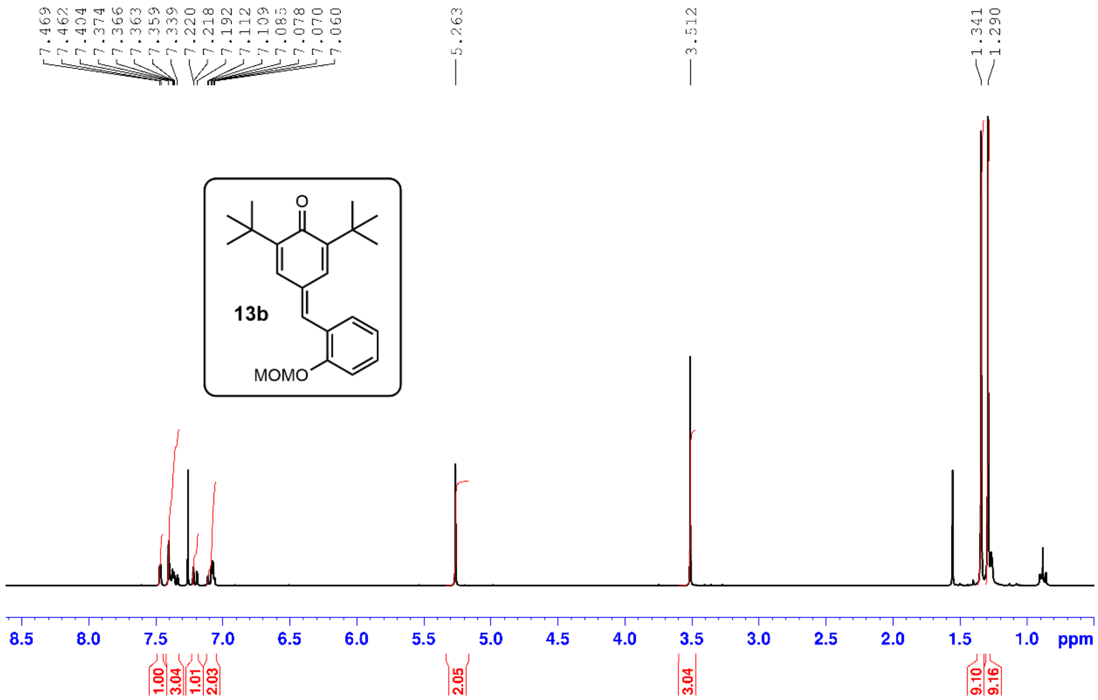


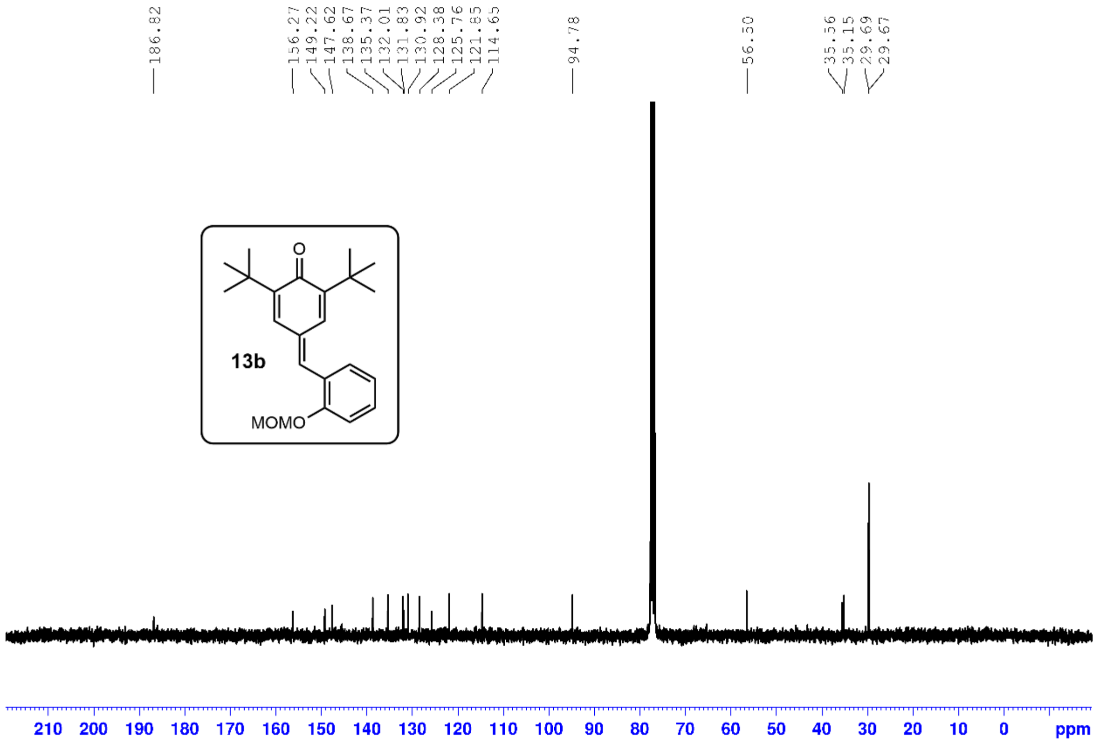

**2,6-di-tert-butyl-4-((2-(methoxymethoxy)naphthalen-1-yl)methylene)cyclohexa-2,5-dien-1-one 13c**

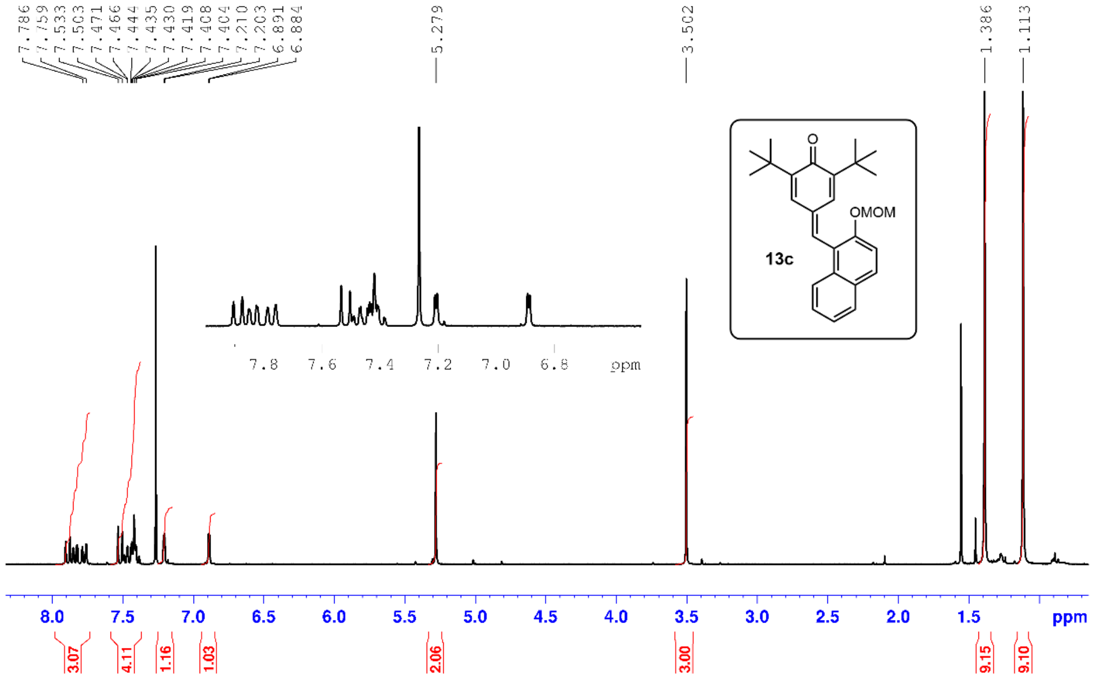


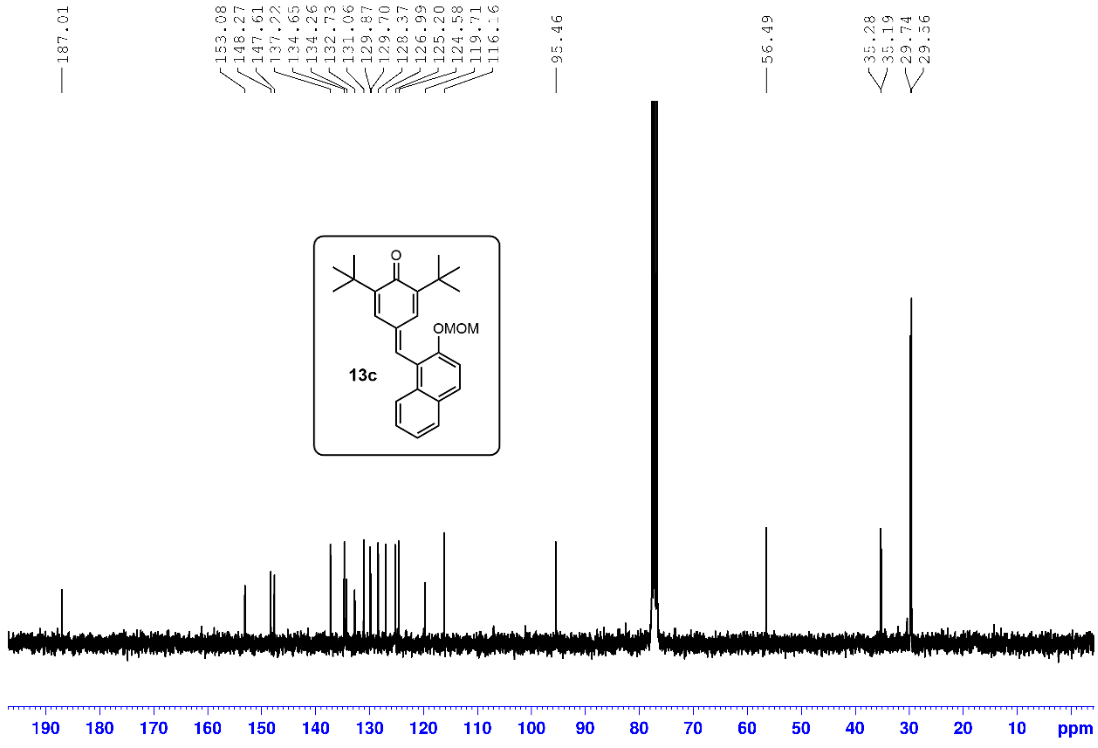

**4-nitrophenyl 3-(3-(tert-butyl)-4-hydroxy-5-methylphenyl)-2-((diphenylmethylene)amino)-3-phenylpropanoate 8a**

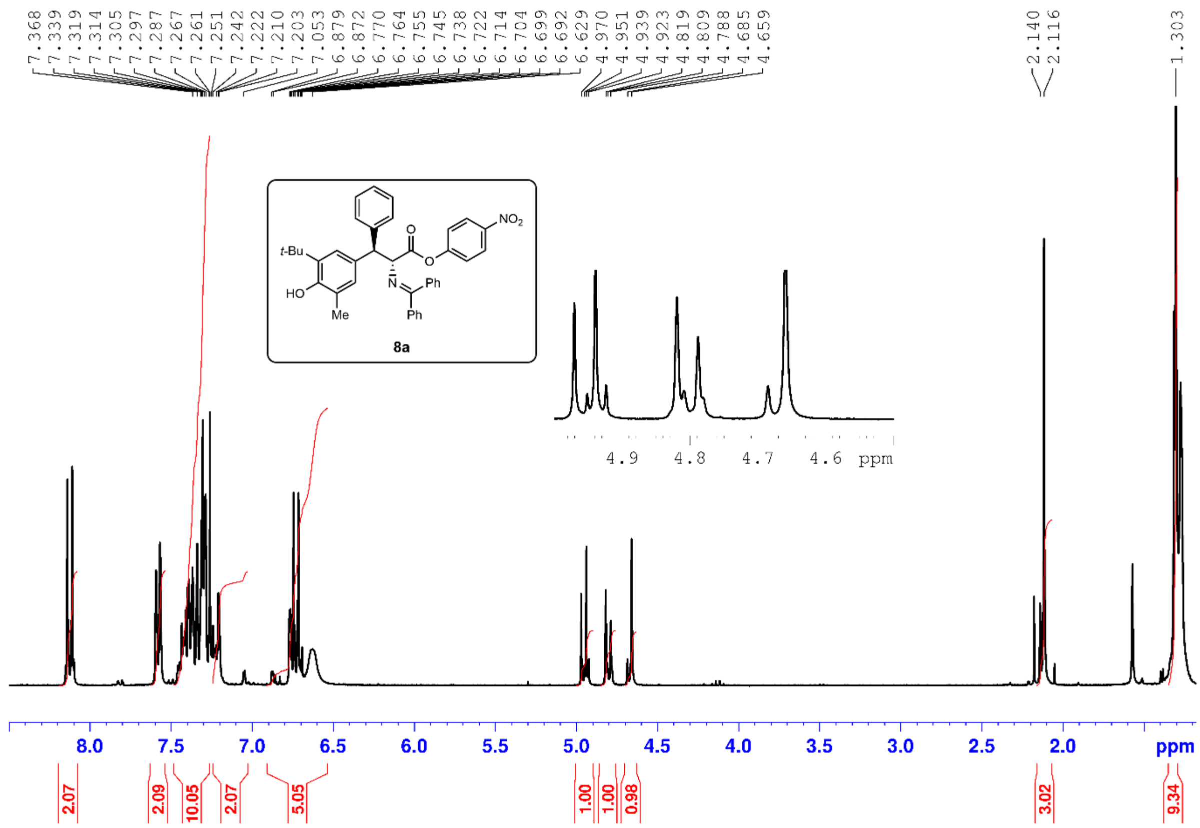


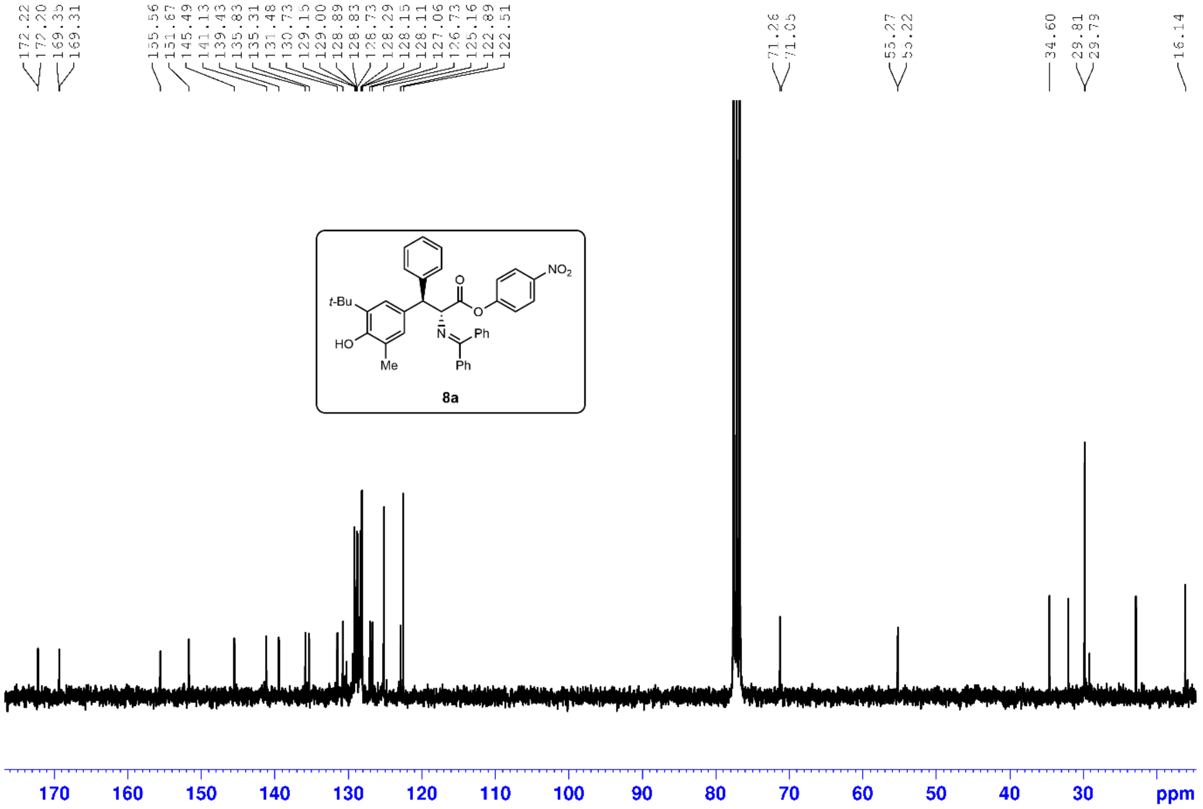

**Chromatogram**

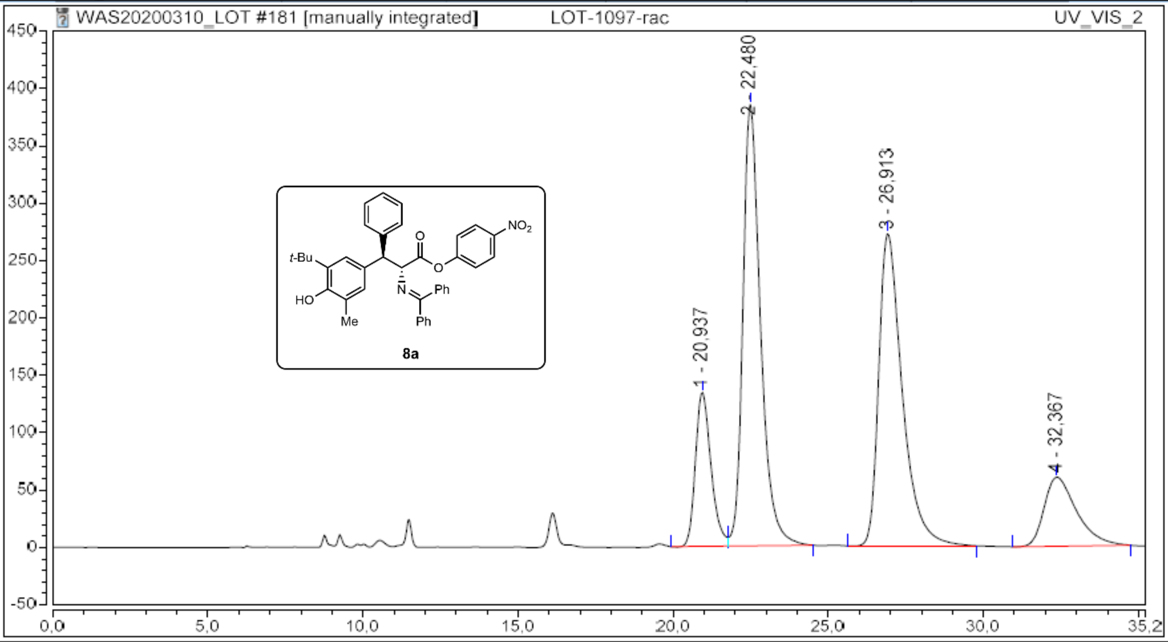


**Integration Results**

| No. | Peak Name | Retention Time min | Area mAU*min | Height mAU | Relative Area % | Relative Height % | Amount n.a. |
|---|---|---|---|---|---|---|---|
| 1 | | 20,937 | 78,216 | 134,470 | 12,15 | 15,79 | n.a. |
| 2 | | 22,480 | 253,680 | 384,778 | 39,39 | 45,18 | n.a. |
| 3 | | 26,913 | 239,080 | 272,240 | 37,12 | 31,97 | n.a. |
| 4 | | 32,367 | 73,042 | 60,107 | 11,34 | 7,06 | n.a. |
| **Total:** | | | **644,018** | **851,595** | **100,00** | **100,00** | |

**Chromatogram**

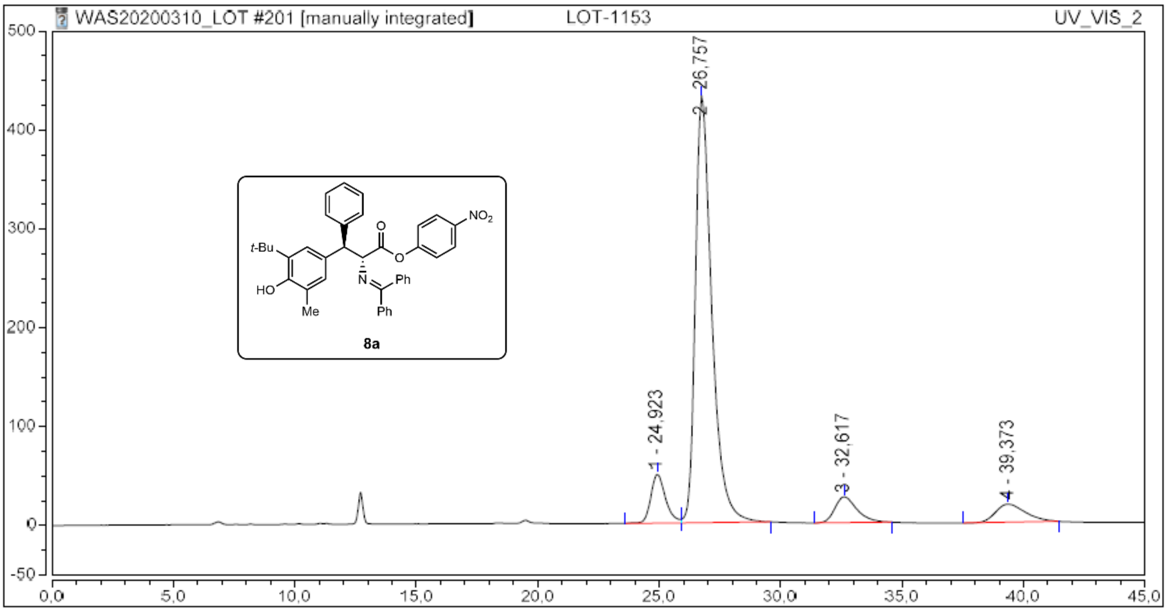


**Integration Results**

| No. | Peak Name | Retention Time min | Area mAU*min | Height mAU | Relative Area % | Relative Height % | Amount n.a. |
|---|---|---|---|---|---|---|---|
| 1 | | 24,923 | 34,893 | 49,524 | 8,15 | 9,46 | n.a. |
| 2 | | 26,757 | 339,487 | 429,546 | 79,32 | 82,08 | n.a. |
| 3 | | 32,617 | 27,583 | 26,026 | 6,44 | 4,97 | n.a. |
| 4 | | 39,373 | 26,020 | 18,248 | 6,08 | 3,49 | n.a. |
| | | | **427,983** | **523,344** | **100,00** | **100,00** | |

**4-nitrophenyl 3-(3,5-di-tert-butyl-4-hydroxyphenyl)-2-((diphenylmethylene)amino)-3-phenylpropanoate 8b**

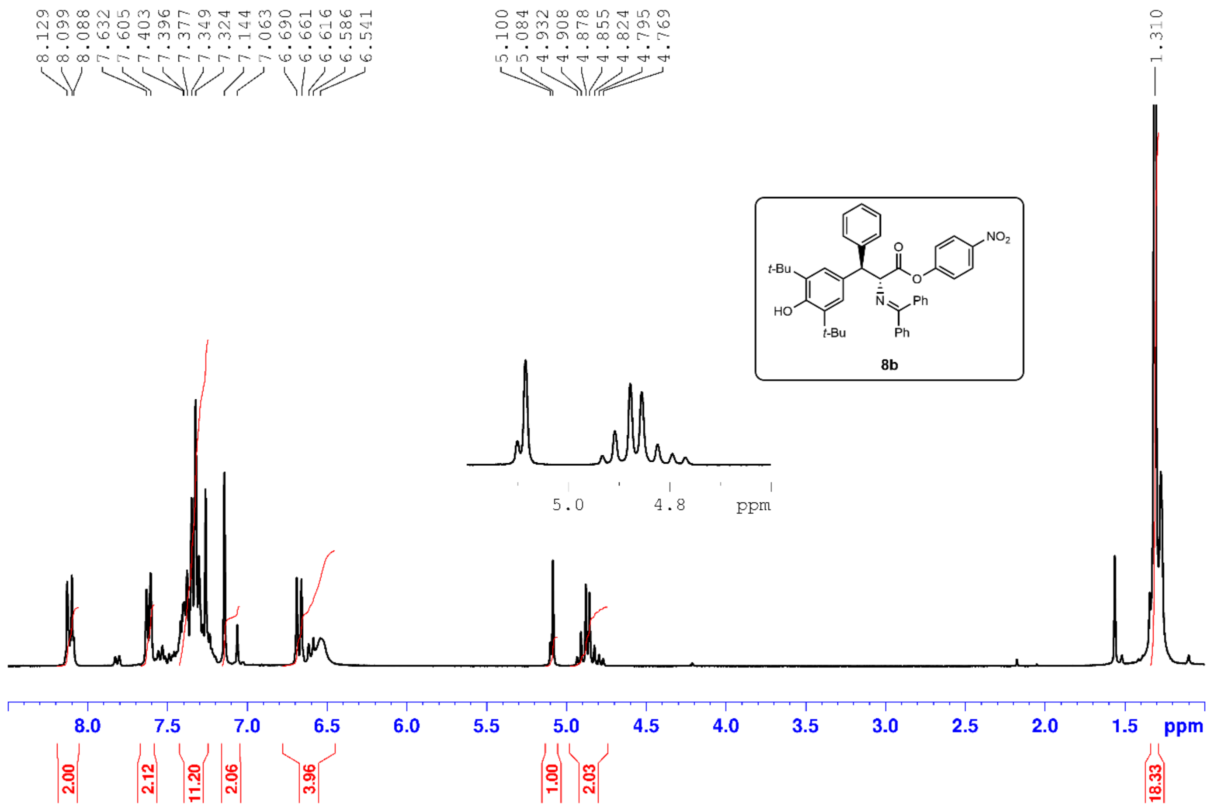

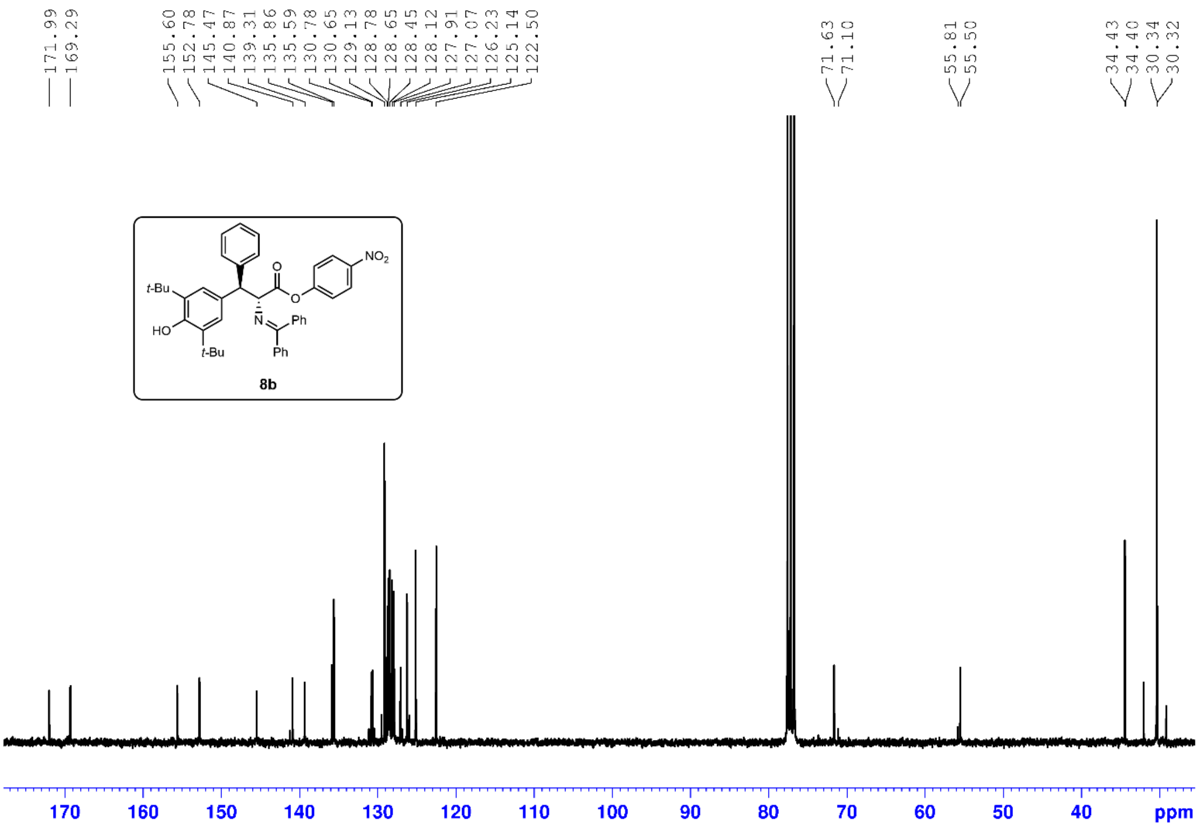

## Chromatogram

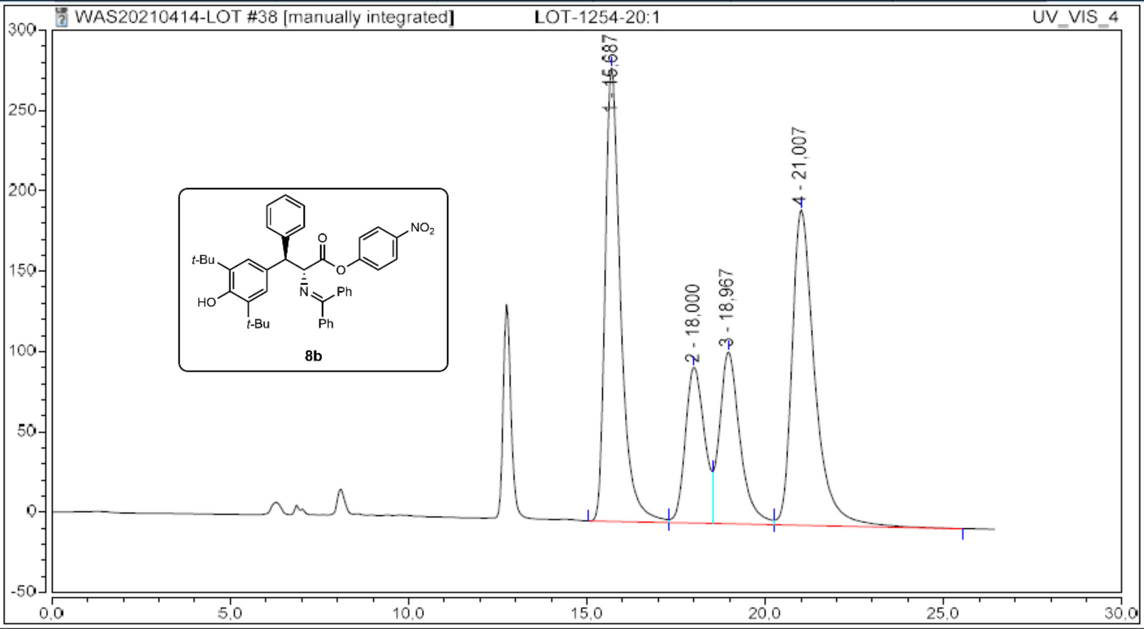


## Integration Results

| No. | Peak Name | Retention Time min | Area mAU*min | Height mAU | Relative Area % | Relative Height % | Amount n.a. |
|---|---|---|---|---|---|---|---|
| 1 | | 15,687 | 142,200 | 282,420 | 33,90 | 41,33 | n.a. |
| 2 | | 18,000 | 60,037 | 97,136 | 14,31 | 14,22 | n.a. |
| 3 | | 18,967 | 71,630 | 107,168 | 17,08 | 15,69 | n.a. |
| 4 | | 21,007 | 145,596 | 196,526 | 34,71 | 28,76 | n.a. |
| **Total:** | | | **419,463** | **683,249** | **100,00** | **100,00** | |

## Chromatogram

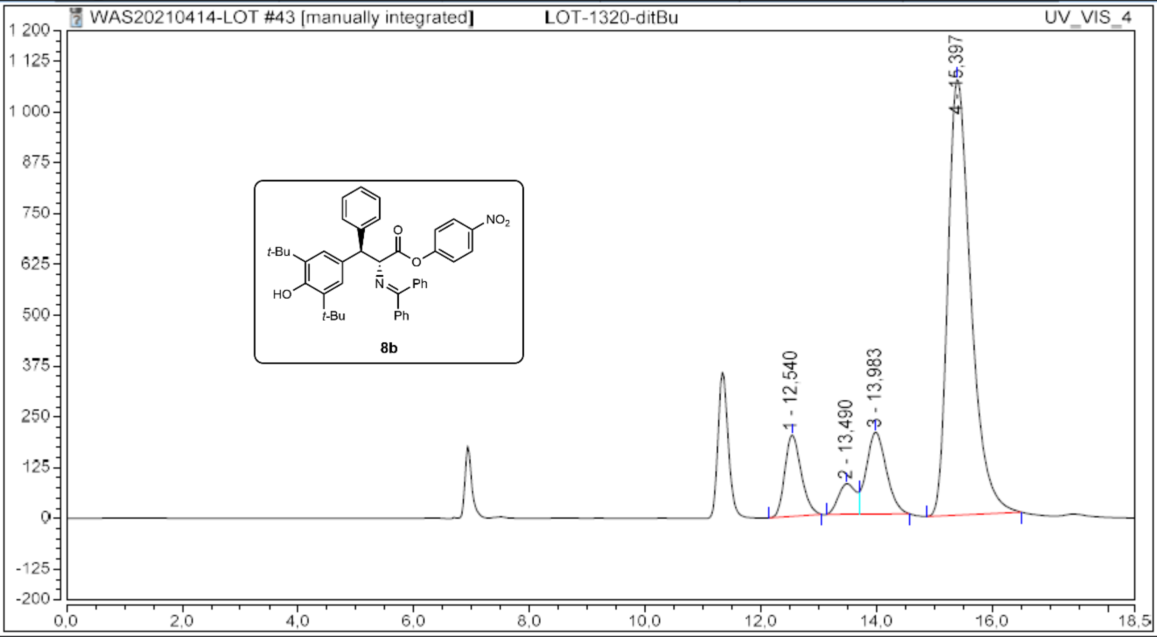


## Integration Results

| No. | Peak Name | Retention Time min | Area mAU*min | Height mAU | Relative Area % | Relative Height % | Amount n.a. |
|---|---|---|---|---|---|---|---|
| 1 | | 12,540 | 65,890 | 200,514 | 9,87 | 12,93 | n.a. |
| 2 | | 13,490 | 26,497 | 75,221 | 3,97 | 4,85 | n.a. |
| 3 | | 13,983 | 80,253 | 201,624 | 12,02 | 13,00 | n.a. |
| 4 | | 15,397 | 494,904 | 1073,627 | 74,14 | 69,22 | n.a. |
| **Total:** | | | **667,544** | **1550,986** | **100,00** | **100,00** | |

**4-nitrophenyl 2-((diphenylmethylene)amino)-3-(4-hydroxy-3,5-diisopropylphenyl)-3-phenylpropanoate 8c**

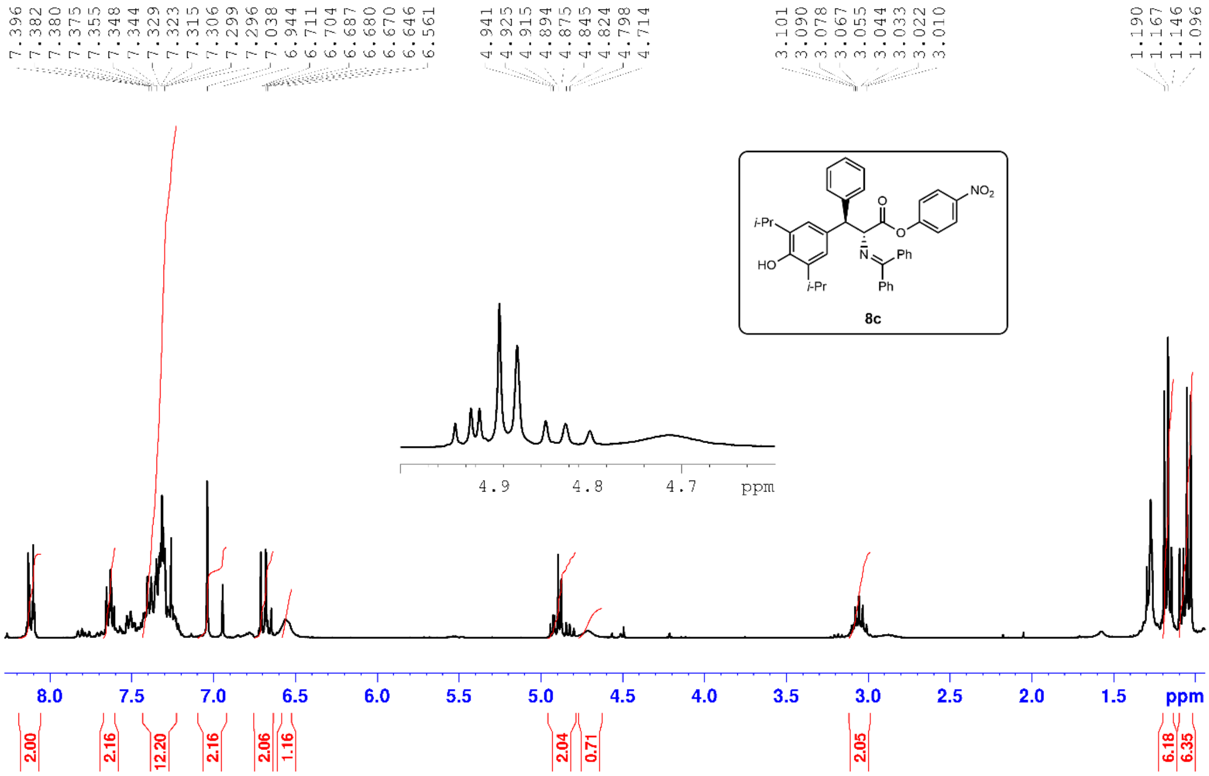


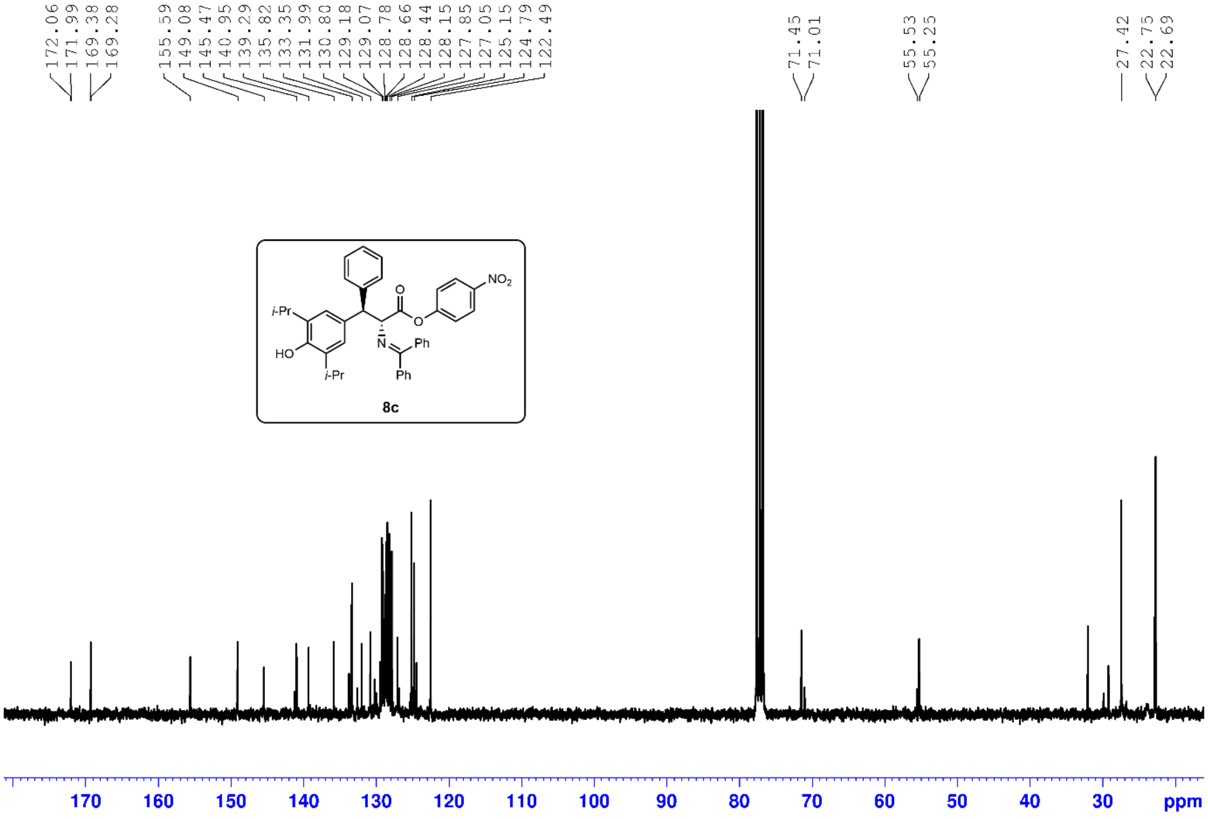

## Chromatogram

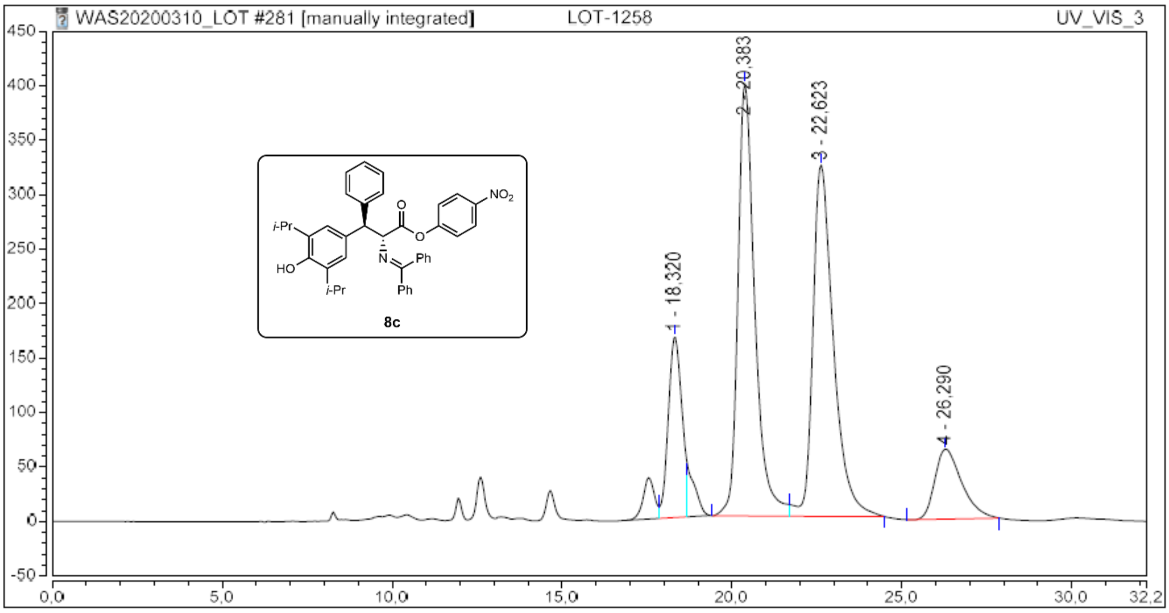


## Integration Results

| No. | Peak Name | Retention Time min | Area mAU*min | Height mAU | Relative Area % | Relative Height % | Amount n.a. |
|---|---|---|---|---|---|---|---|
| 1 | | 18,320 | 77,214 | 166,518 | 13,04 | 17,51 | n.a. |
| 2 | | 20,383 | 229,414 | 397,118 | 38,76 | 41,77 | n.a. |
| 3 | | 22,623 | 226,476 | 322,912 | 38,26 | 33,96 | n.a. |
| 4 | | 26,290 | 58,855 | 64,272 | 9,94 | 6,76 | n.a. |
| | | | **591,960** | **950,820** | **100,00** | **100,00** | |

## Chromatogram

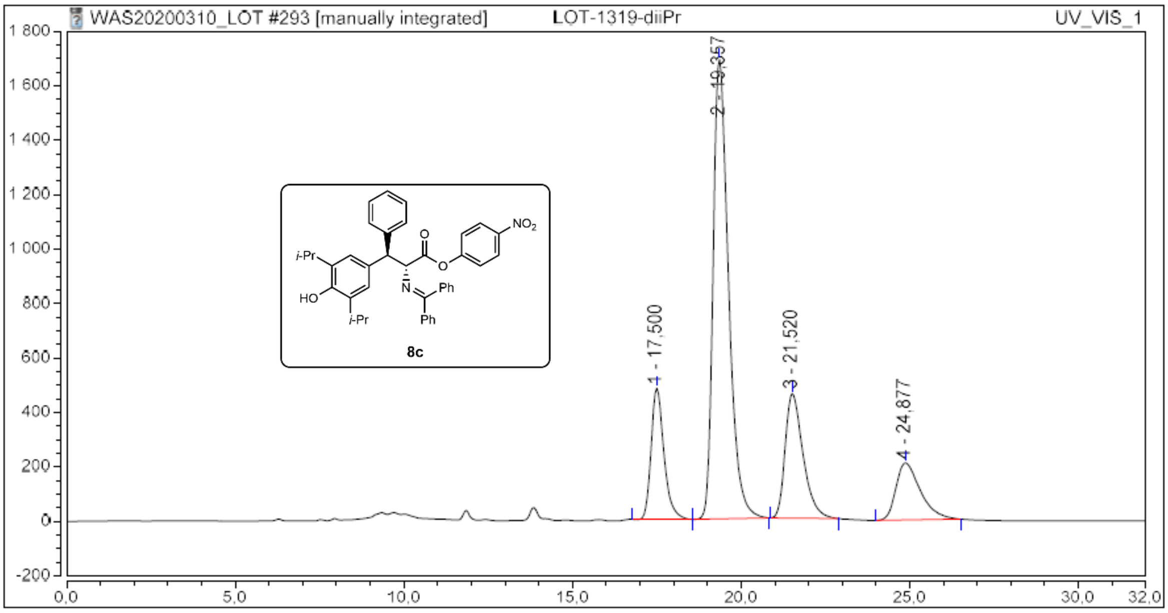


## Integration Results

| No. | Peak Name | Retention Time min | Area mAU*min | Height mAU | Relative Area % | Relative Height % | Amount n.a. |
|---|---|---|---|---|---|---|---|
| 1 | | 17,500 | 212,791 | 482,056 | 13,62 | 16,99 | n.a. |
| 2 | | 19,357 | 891,913 | 1687,335 | 57,07 | 59,48 | n.a. |
| 3 | | 21,520 | 281,615 | 457,753 | 18,02 | 16,14 | n.a. |
| 4 | | 24,877 | 176,455 | 209,443 | 11,29 | 7,38 | n.a. |
| | | | **1562,774** | **2836,587** | **100,00** | **100,00** | |

**4-nitrophenyl 3-(3,5-di-tert-butyl-4-hydroxyphenyl)-2-((diphenylmethylene)amino)-3-(o-tolyl)propanoate 8d**

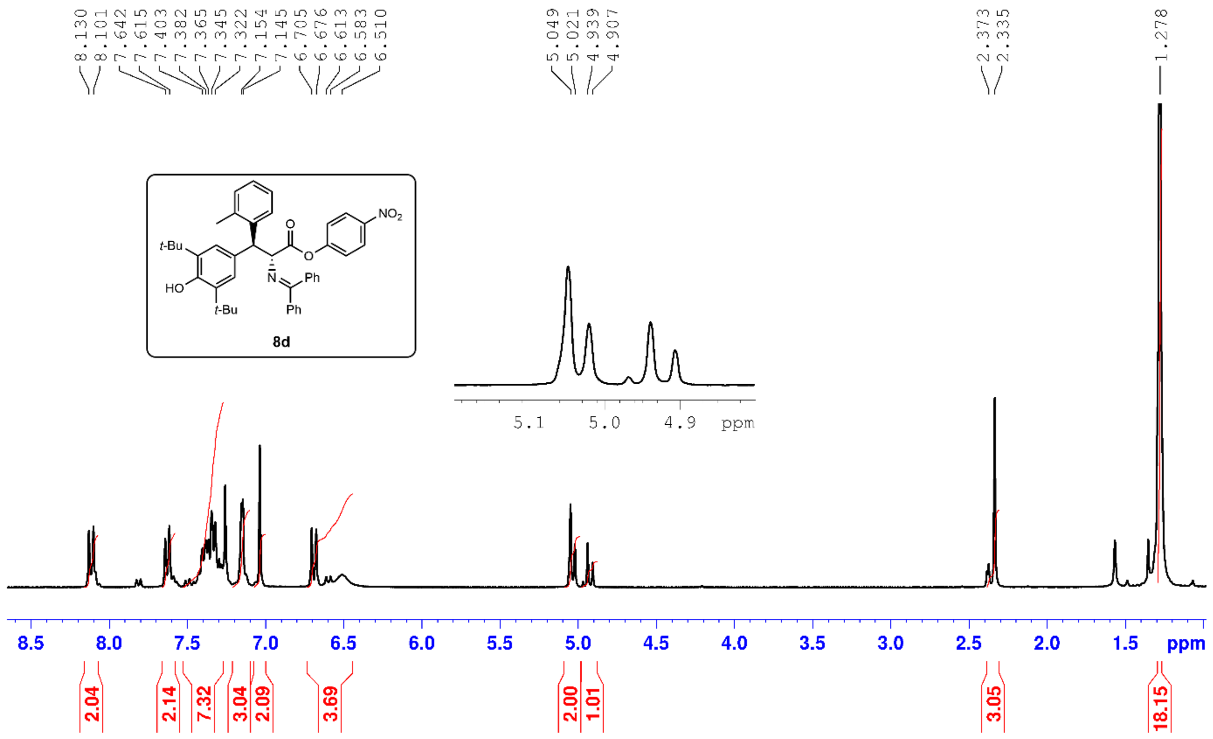

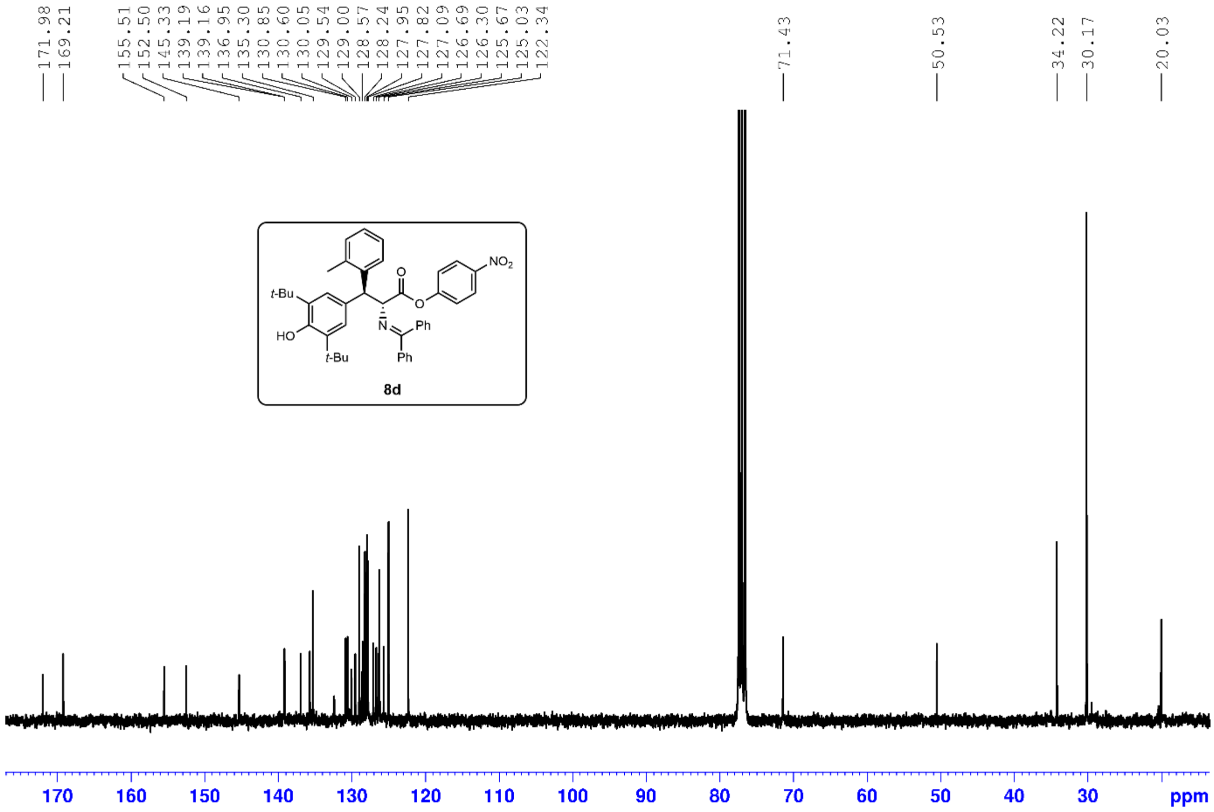

## Chromatogram

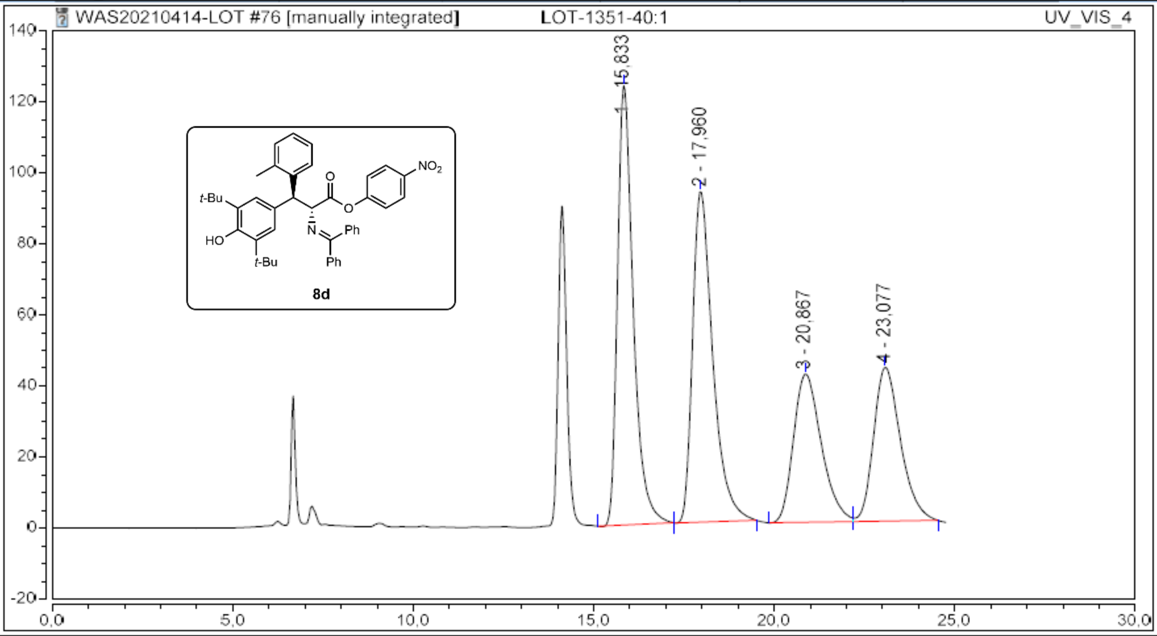


## Integration Results

| No. | Peak Name | Retention Time<br>min | Area<br>mAU*min | Height<br>mAU | Relative Area<br>% | Relative Height<br>% | Amount<br>n.a. |
|---|---|---|---|---|---|---|---|
| 1 | | 15,833 | 62,532 | 123,703 | 31,64 | 40,95 | n.a. |
| 2 | | 17,960 | 60,421 | 93,278 | 30,57 | 30,88 | n.a. |
| 3 | | 20,867 | 37,282 | 41,771 | 18,86 | 13,83 | n.a. |
| 4 | | 23,077 | 37,410 | 43,318 | 18,93 | 14,34 | n.a. |
| **Total:** | | | **197,644** | **302,070** | **100,00** | **100,00** | |

## Chromatogram

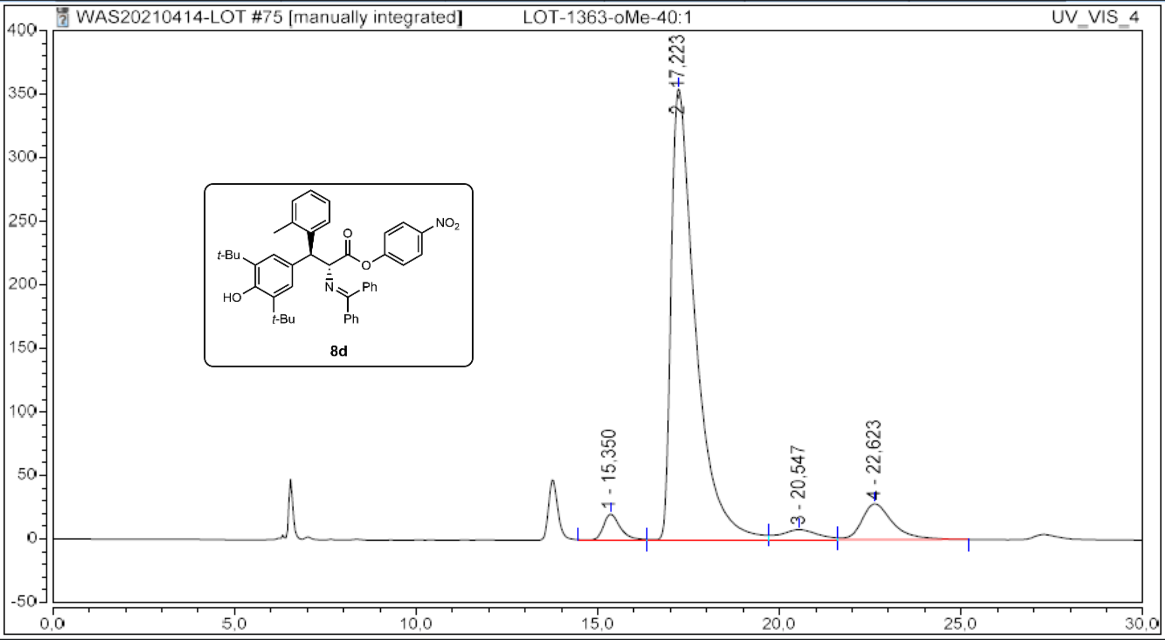


## Integration Results

| No. | Peak Name | Retention Time<br>min | Area<br>mAU*min | Height<br>mAU | Relative Area<br>% | Relative Height<br>% | Amount<br>n.a. |
|---|---|---|---|---|---|---|---|
| 1 | | 15,350 | 10,368 | 20,048 | 3,23 | 4,88 | n.a. |
| 2 | | 17,223 | 274,140 | 354,714 | 85,37 | 86,39 | n.a. |
| 3 | | 20,547 | 8,967 | 7,751 | 2,79 | 1,89 | n.a. |
| 4 | | 22,623 | 27,654 | 28,073 | 8,61 | 6,84 | n.a. |
| **Total:** | | | **321,129** | **410,586** | **100,00** | **100,00** | |

**4-nitrophenyl 3-(3,5-di-tert-butyl-4-hydroxyphenyl)-2-((diphenylmethylene)amino)-3-(*m*-tolyl)propanoate 8e**

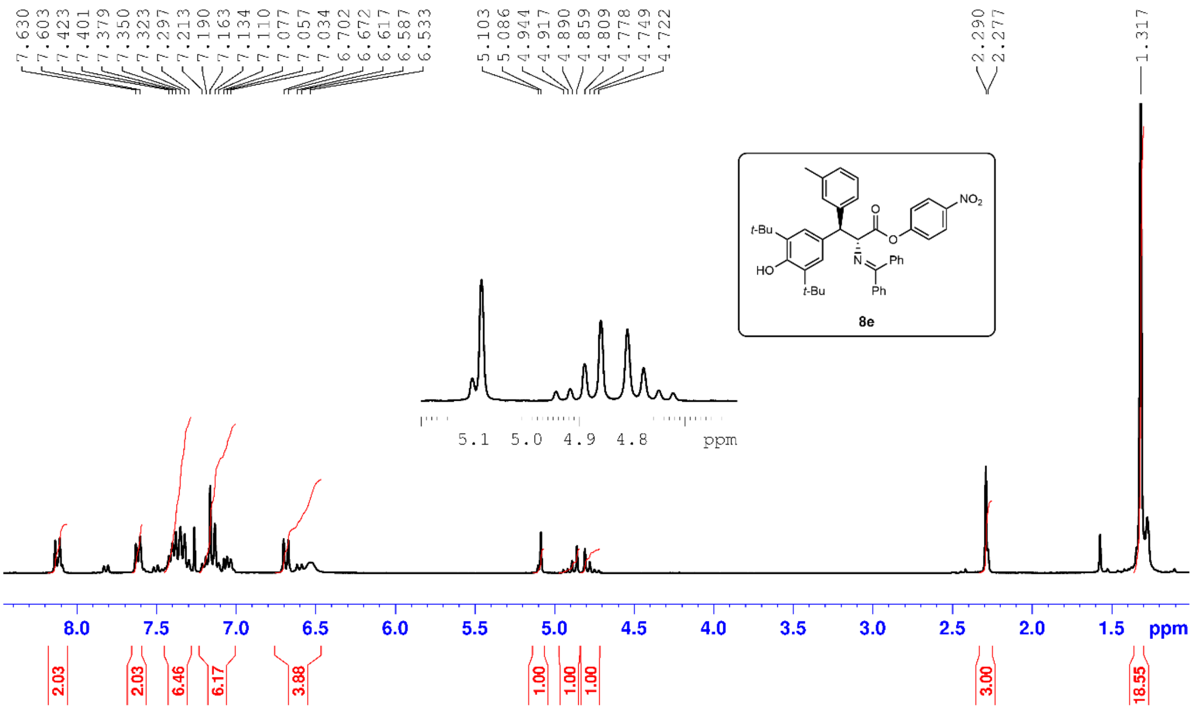


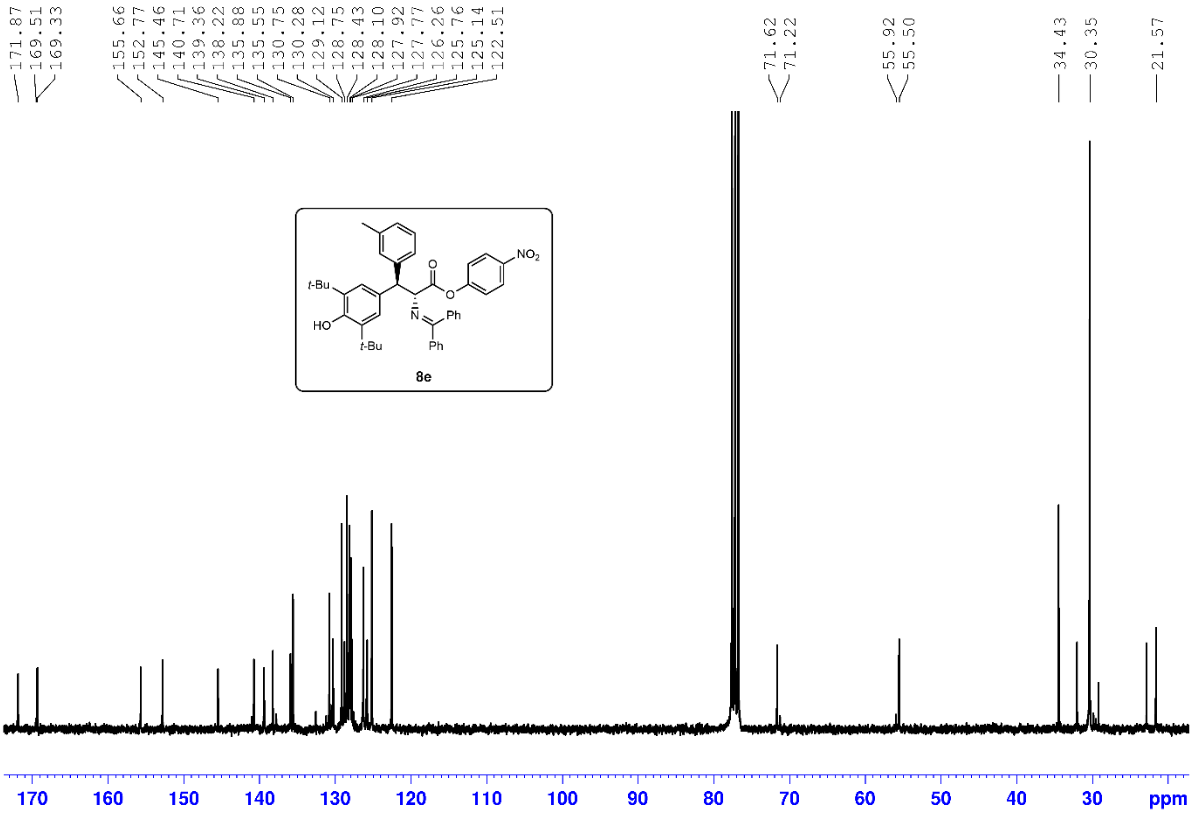

**Chromatogram**

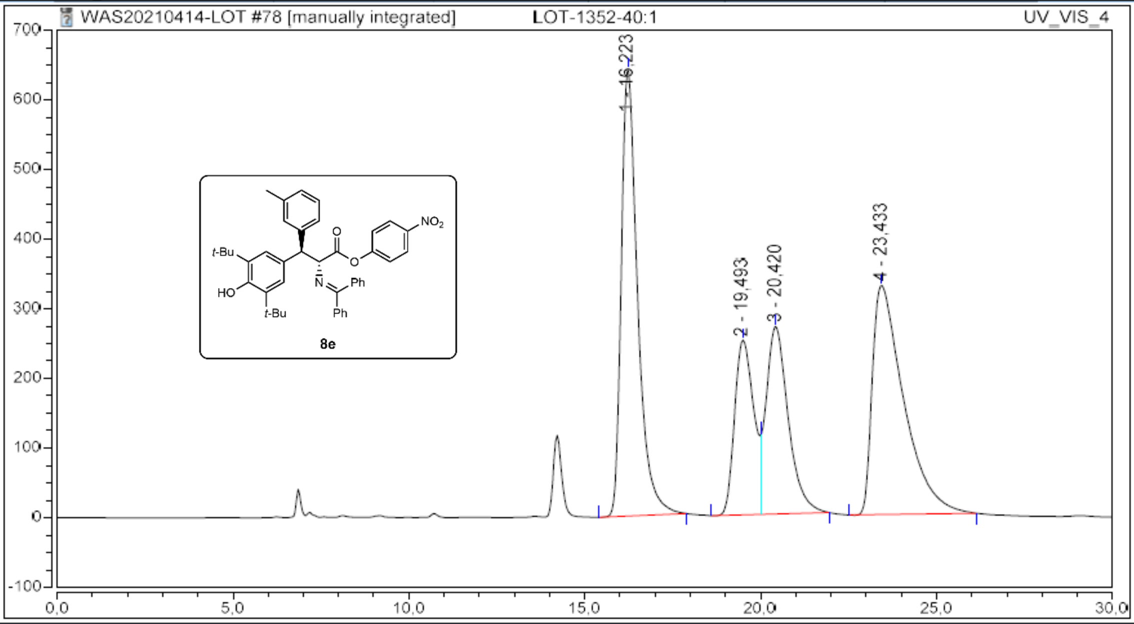


**Integration Results**

| No. | Peak Name | Retention Time min | Area mAU*min | Height mAU | Relative Area % | Relative Height % | Amount n.a. |
|---|---|---|---|---|---|---|---|
| 1 | | 16,223 | 345,434 | 642,423 | 33,21 | 43,06 | n.a. |
| 2 | | 19,493 | 160,623 | 250,841 | 15,44 | 16,81 | n.a. |
| 3 | | 20,420 | 194,350 | 269,528 | 18,68 | 18,07 | n.a. |
| 4 | | 23,433 | 339,776 | 329,186 | 32,67 | 22,06 | n.a. |
| **Total:** | | | **1040,184** | **1491,978** | **100,00** | **100,00** | |

**Chromatogram**

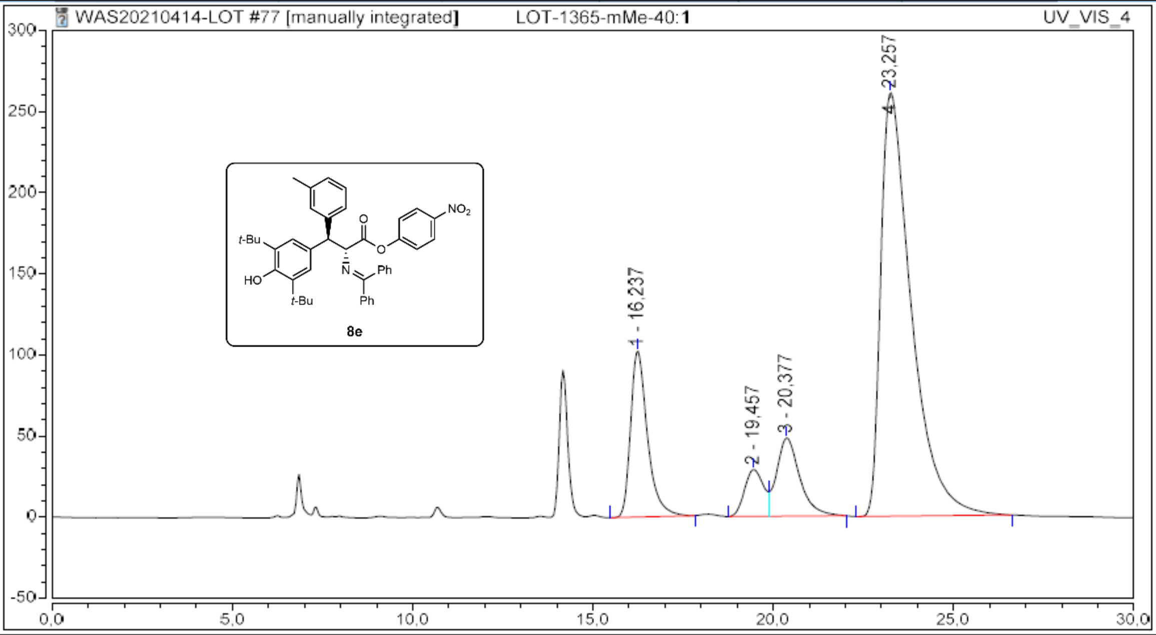


**Integration Results**

| No. | Peak Name | Retention Time min | Area mAU*min | Height mAU | Relative Area % | Relative Height % | Amount n.a. |
|---|---|---|---|---|---|---|---|
| 1 | | 16,237 | 55,557 | 102,531 | 15,16 | 23,25 | n.a. |
| 2 | | 19,457 | 17,779 | 28,841 | 4,85 | 6,54 | n.a. |
| 3 | | 20,377 | 35,780 | 48,263 | 9,76 | 10,94 | n.a. |
| 4 | | 23,257 | 257,303 | 261,377 | 70,22 | 59,27 | n.a. |
| **Total:** | | | **366,419** | **441,011** | **100,00** | **100,00** | |

**4-nitrophenyl 3-(3,5-di-tert-butyl-4-hydroxyphenyl)-2-((diphenylmethylene)amino)-3-(*p*-tolyl)propanoate 8f**

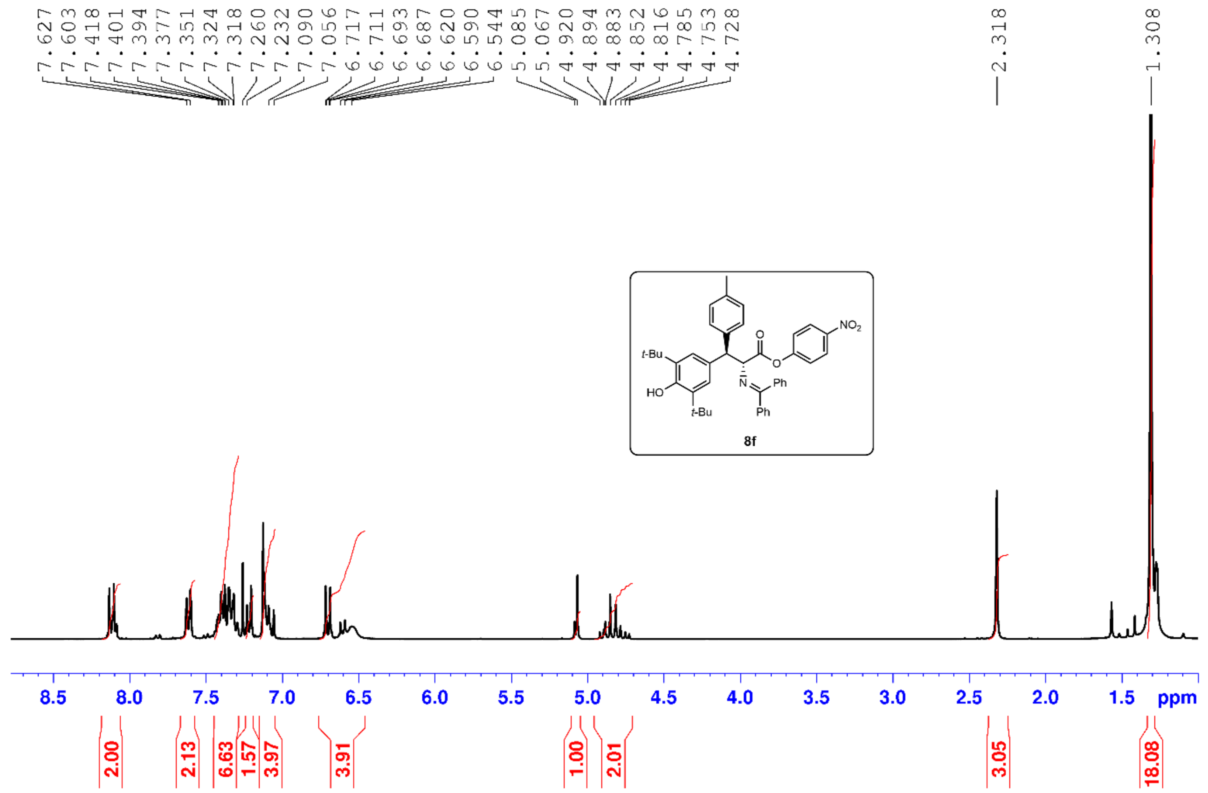


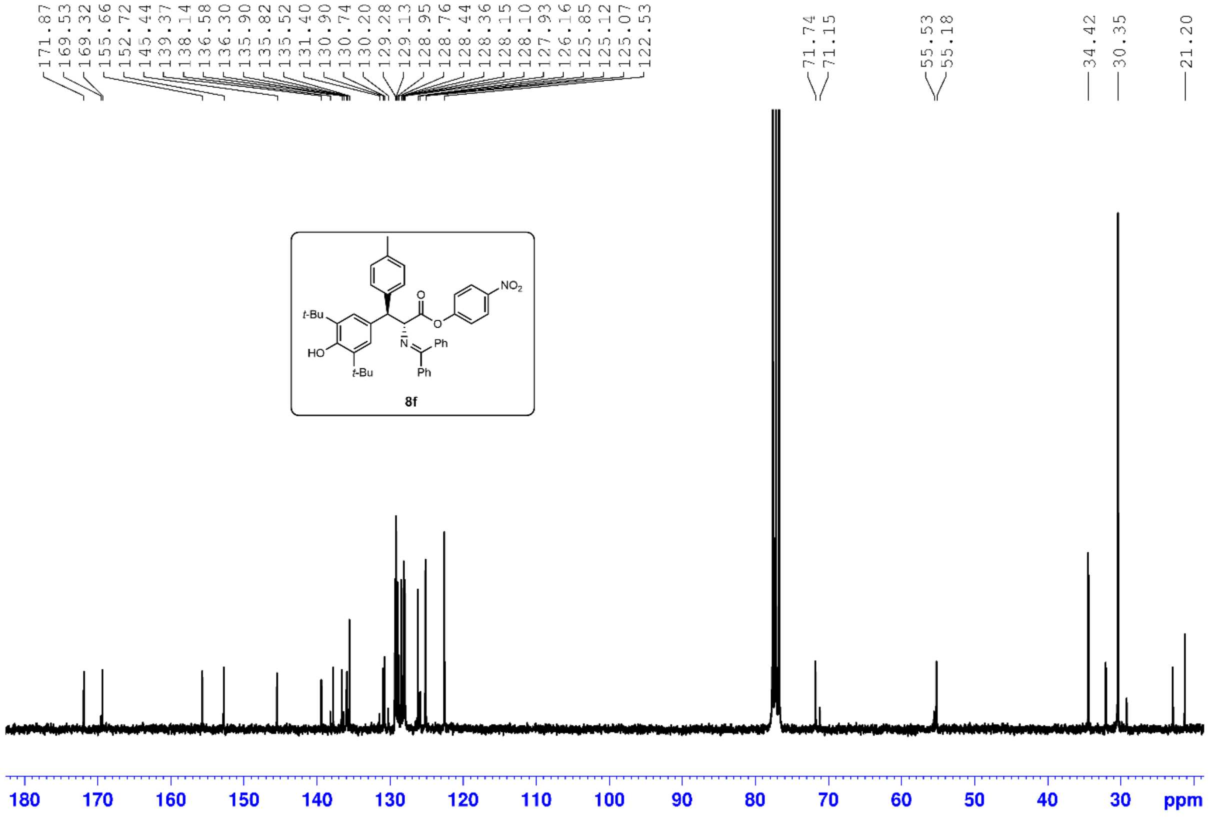

**Chromatogram**

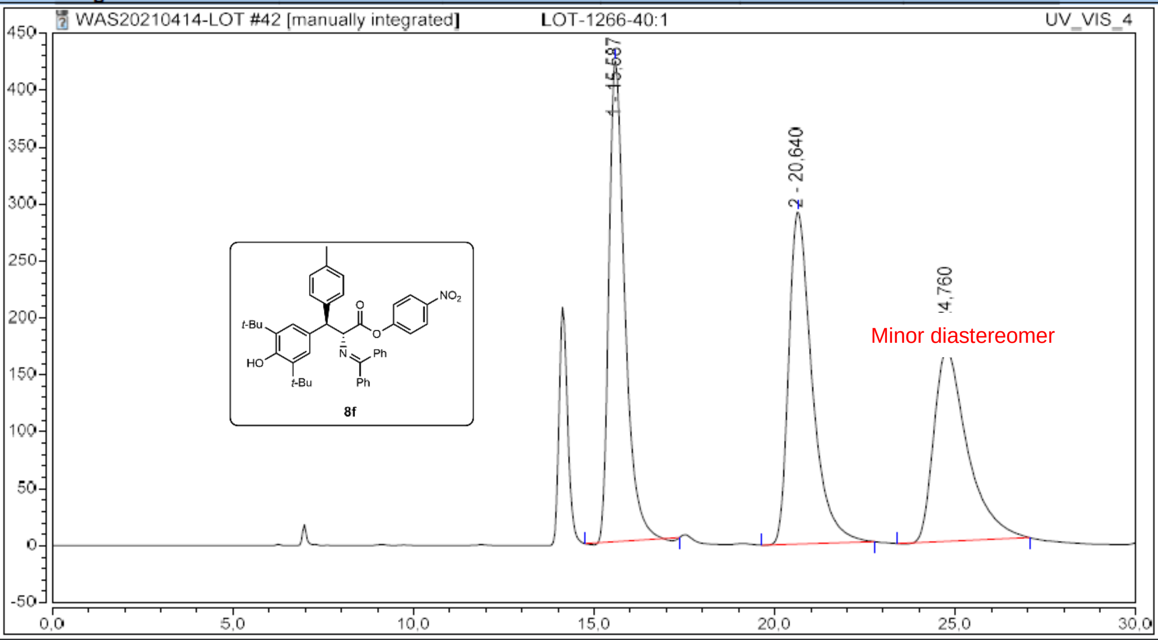


**Integration Results**

| No. | Peak Name | Retention Time min | Area mAU*min | Height mAU | Relative Area % | Relative Height % | Amount n.a. |
|---|---|---|---|---|---|---|---|
| 1 | | 15,587 | 214,820 | 422,375 | 34,93 | 47,88 | n.a. |
| 2 | | 20,640 | 219,342 | 291,974 | 35,66 | 33,10 | n.a. |
| 3 | | 24,760 | 180,824 | 167,665 | 29,40 | 19,01 | n.a. |
| 4 | | 46,070 | 0,071 | 0,083 | 0,01 | 0,01 | n.a. |
| **Total:** | | | **615,057** | **882,097** | **100,00** | **100,00** | |

**Chromatogram**

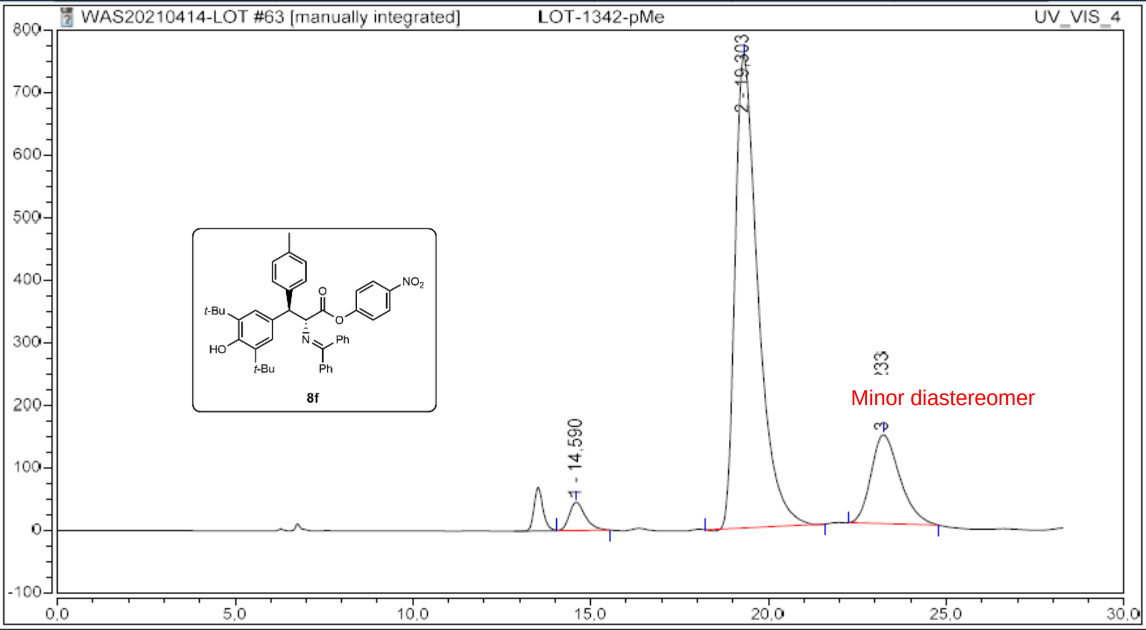


**Integration Results**

| No. | Peak Name | Retention Time min | Area mAU*min | Height mAU | Relative Area % | Relative Height % | Amount n.a. |
|---|---|---|---|---|---|---|---|
| 1 | | 14,590 | 23,536 | 45,163 | 3,25 | 4,79 | n.a. |
| 2 | | 19,303 | 566,738 | 755,125 | 78,25 | 80,12 | n.a. |
| 3 | | 23,233 | 134,003 | 142,174 | 18,50 | 15,09 | n.a. |
| **Total:** | | | **724,277** | **942,462** | **100,00** | **100,00** | |

**4-nitrophenyl 3-(3,5-di-tert-butyl-4-hydroxyphenyl)-2-((diphenylmethylene)amino)-3-(2-methoxyphenyl)propanoate 8g**

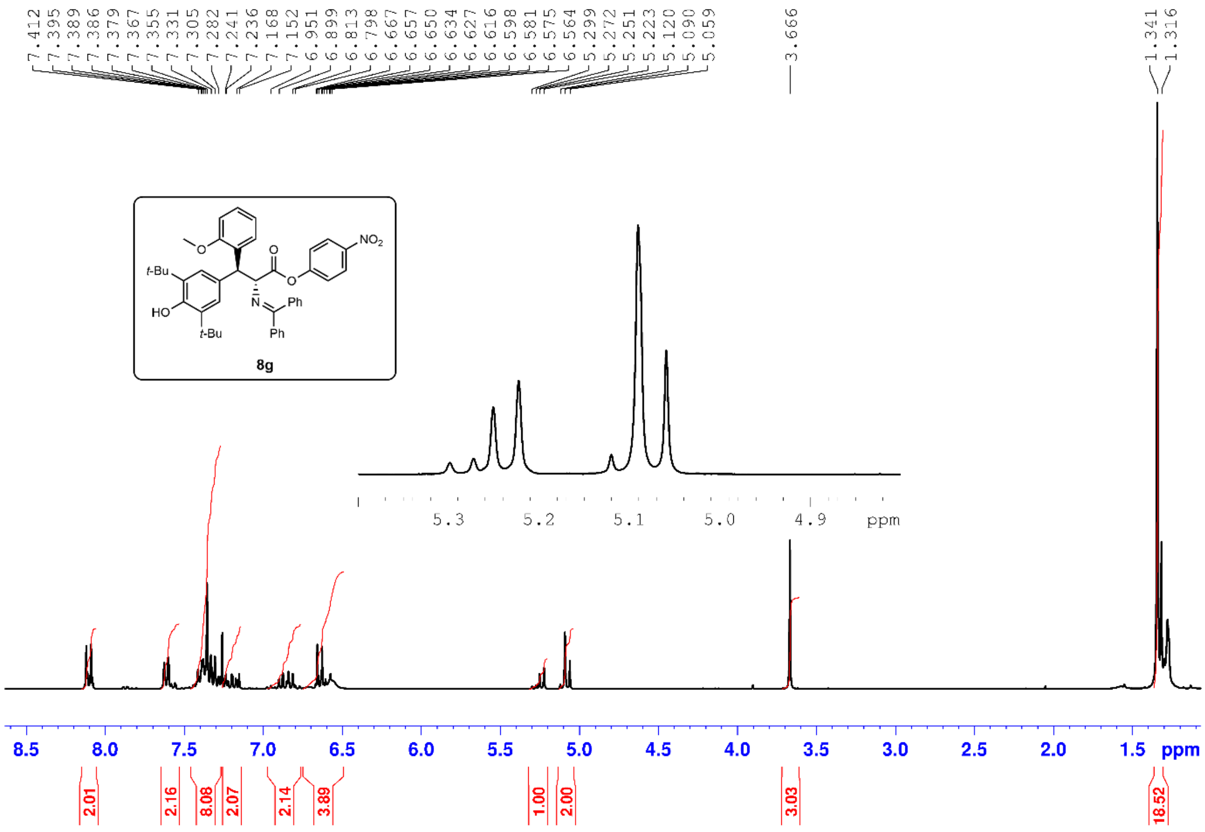


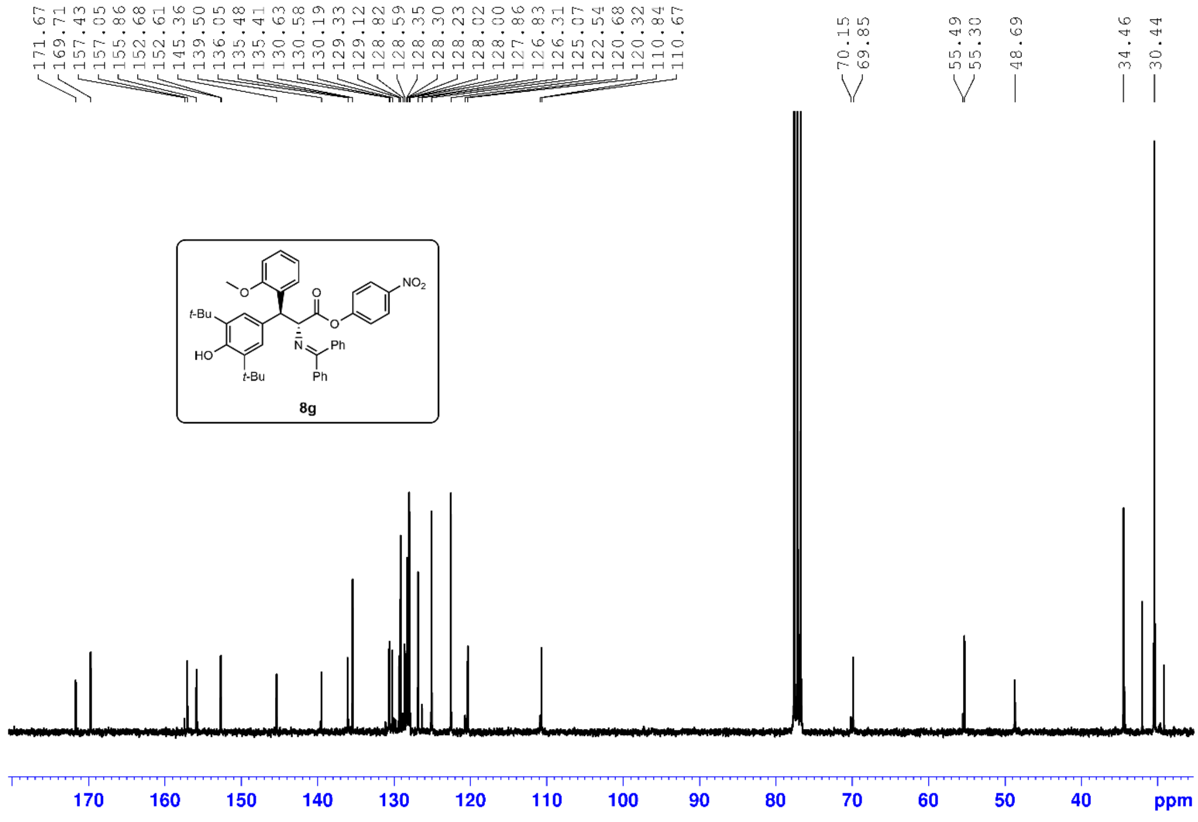

**Semipreparative HPLC** (Separation of the diastereomers of **8g**):

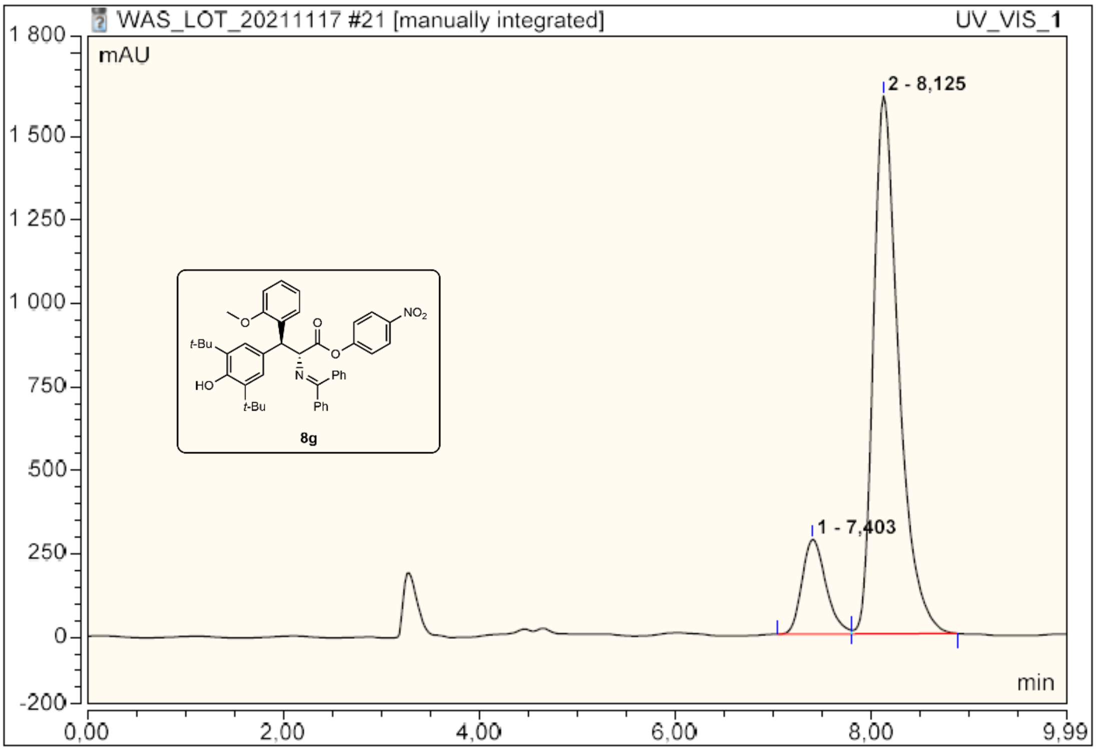


| No. | Ret.Time min | Peak Name | Height mAU | Area mAU*min | Rel.Area % | Amount n.a. | Type |
|---|---|---|---|---|---|---|---|
| 1 | 7,40 | | 283,606 | 80,314 | 14,24 | n.a. | BM * |
| 2 | 8,12 | | 1610,836 | 483,555 | 85,76 | n.a. | MB* |
| Total: | | | 1894,442 | 563,869 | 100,00 | 0,000 | |

**Major diastereomer:**

**Chromatogram**

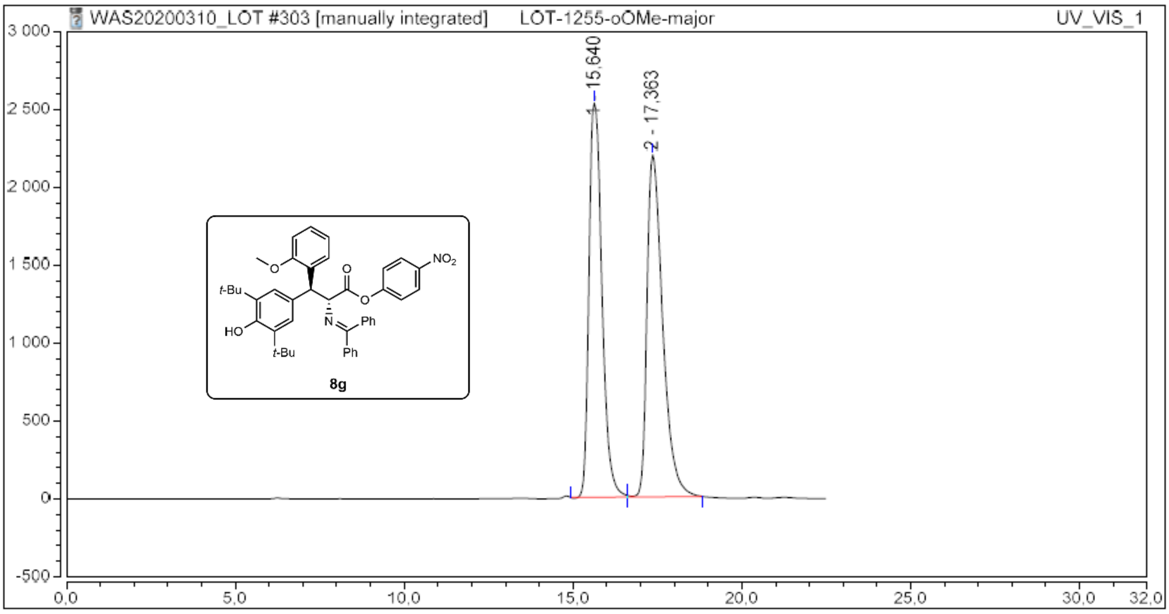


**Integration Results**

| No. | Peak Name | Retention Time min | Area mAU*min | Height mAU | Relative Area % | Relative Height % | Amount n.a. |
|---|---|---|---|---|---|---|---|
| 1 | | 15,640 | 1123,880 | 2530,678 | 48,81 | 53,55 | n.a. |
| 2 | | 17,363 | 1178,855 | 2195,320 | 51,19 | 46,45 | n.a. |
| | | | **2302,735** | **4725,998** | **100,00** | **100,00** | |

**Chromatogram**

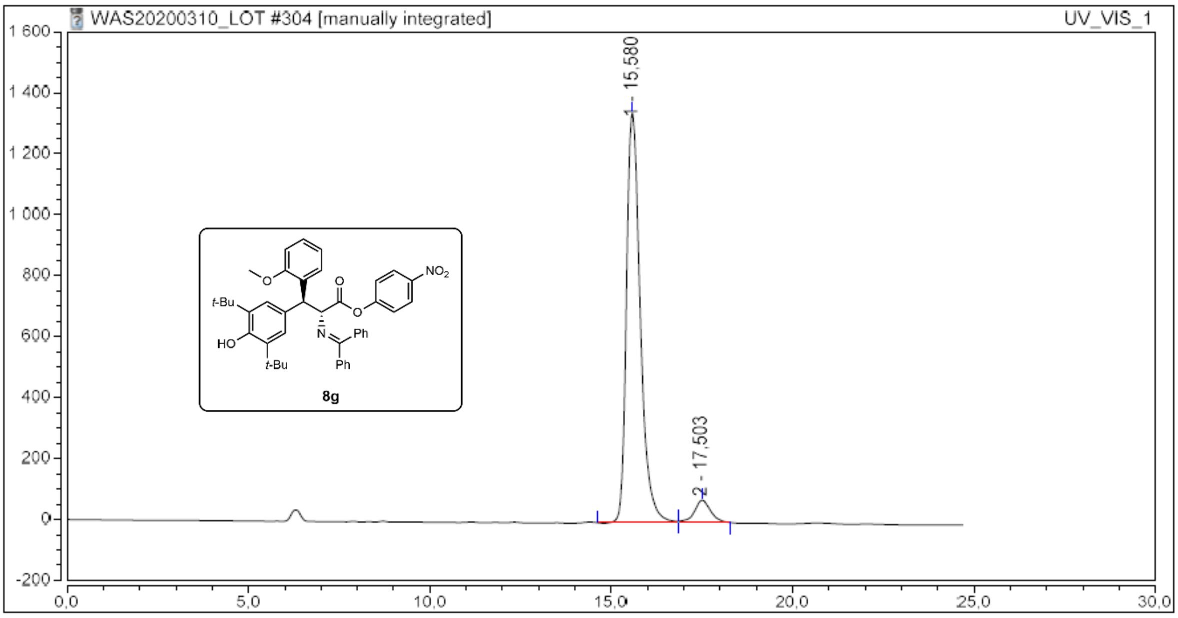


**Integration Results**

| No. | Peak Name | Retention Time min | Area mAU*min | Height mAU | Relative Area % | Relative Height % | Amount n.a. |
|---|---|---|---|---|---|---|---|
| 1 | | 15,580 | 595,493 | 1344,441 | 93,90 | 94,82 | n.a. |
| 2 | | 17,503 | 38,658 | 73,508 | 6,10 | 5,18 | n.a. |
| | | | **634,151** | **1417,949** | **100,00** | **100,00** | |

**Minor diastereomer:**

Chromatogram

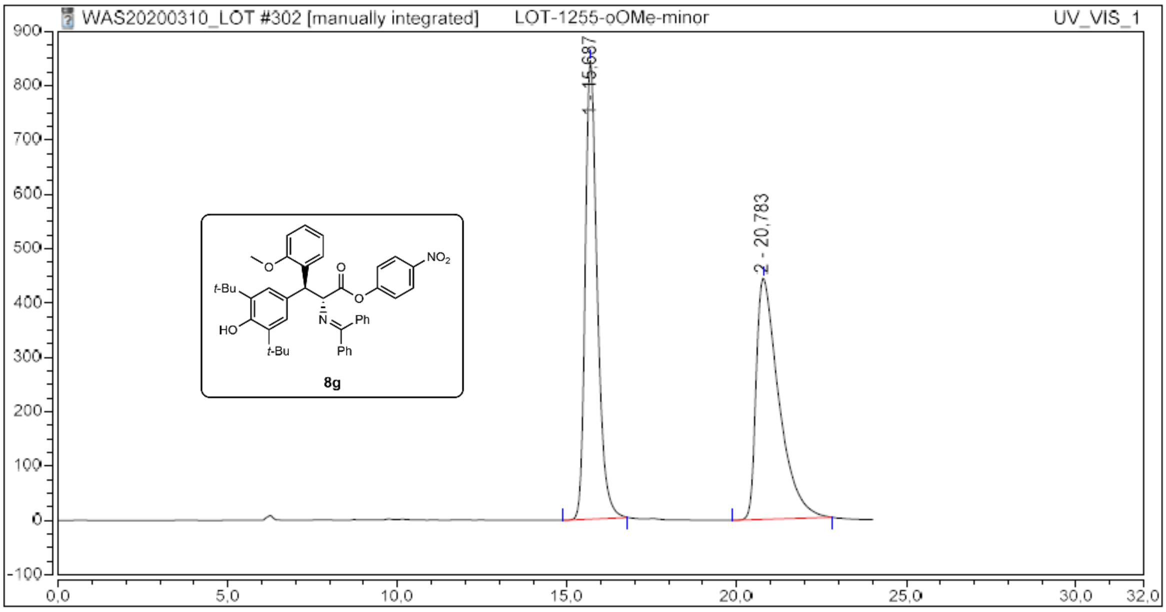


Integration Results

| No. | Peak Name | Retention Time min | Area mAU*min | Height mAU | Relative Area % | Relative Height % | Amount n.a. |
|---|---|---|---|---|---|---|---|
| 1 | | 15,687 | 346,483 | 843,778 | 50,25 | 65,51 | n.a. |
| 2 | | 20,783 | 343,092 | 444,301 | 49,75 | 34,49 | n.a. |
| | | | **689,576** | **1288,079** | **100,00** | **100,00** | |

Chromatogram

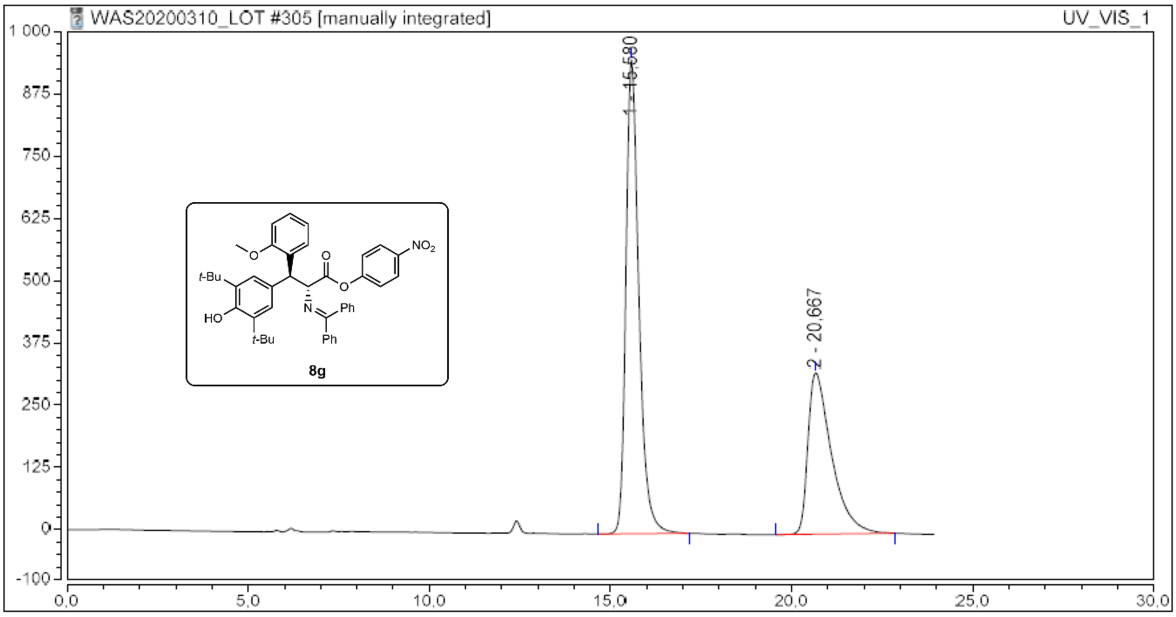


Integration Results

| No. | Peak Name | Retention Time min | Area mAU*min | Height mAU | Relative Area % | Relative Height % | Amount n.a. |
|---|---|---|---|---|---|---|---|
| 1 | | 15,580 | 395,420 | 951,324 | 61,79 | 74,60 | n.a. |
| 2 | | 20,667 | 244,526 | 323,975 | 38,21 | 25,40 | n.a. |
| | | | **639,945** | **1275,299** | **100,00** | **100,00** | |

**4-nitrophenyl 3-(3,5-di-tert-butyl-4-hydroxyphenyl)-2-((diphenylmethylene)amino)-3-(4-methoxyphenyl)propanoate**

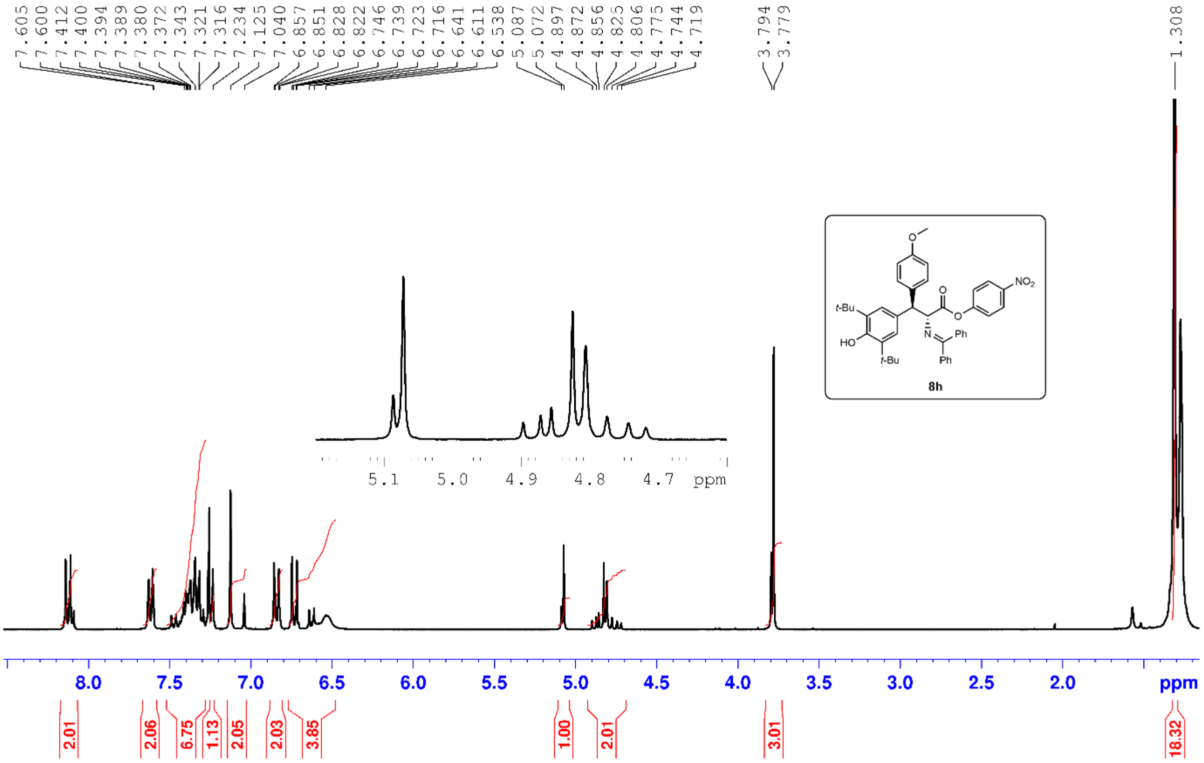


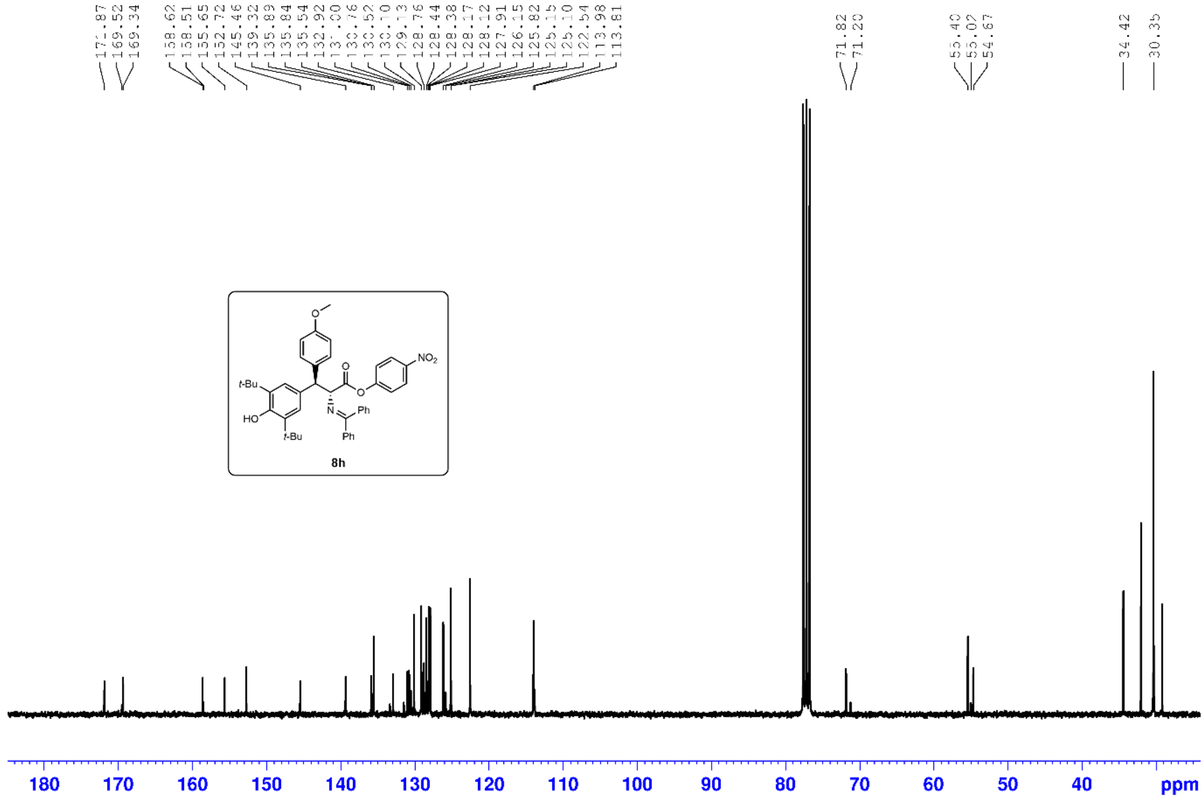

**Semipreparative HPLC** (Separation of the diastereomers of **8h**)::

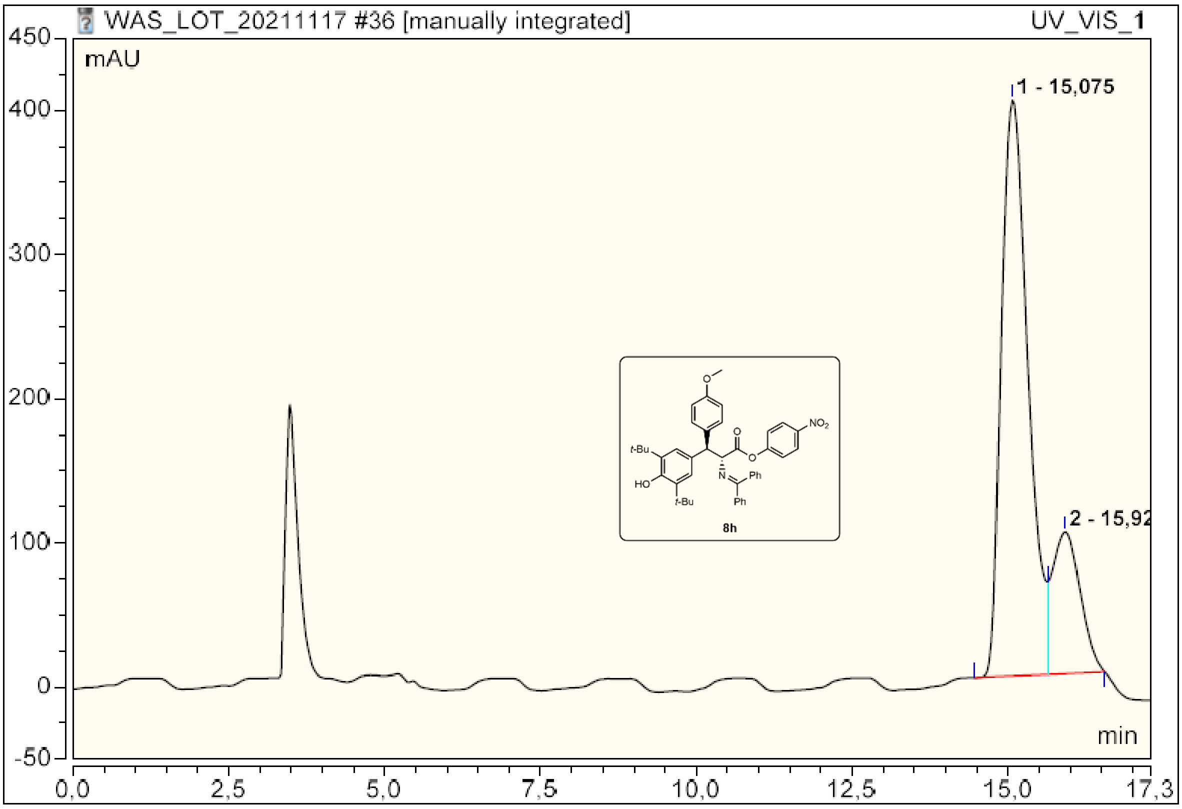


| No. | Ret.Time min | Peak Name | Height mAU | Area mAU*min | Rel.Area % | Amount n.a. | Type |
|---|---|---|---|---|---|---|---|
| 1 | 15,07 | | 399,863 | 198,450 | 79,78 | n.a. | BM * |
| 2 | 15,93 | | 98,498 | 50,311 | 20,22 | n.a. | MB* |
| Total: | | | 498,361 | 248,761 | 100,00 | 0,000 | |

**Major diastereomer:**

Chromatogram

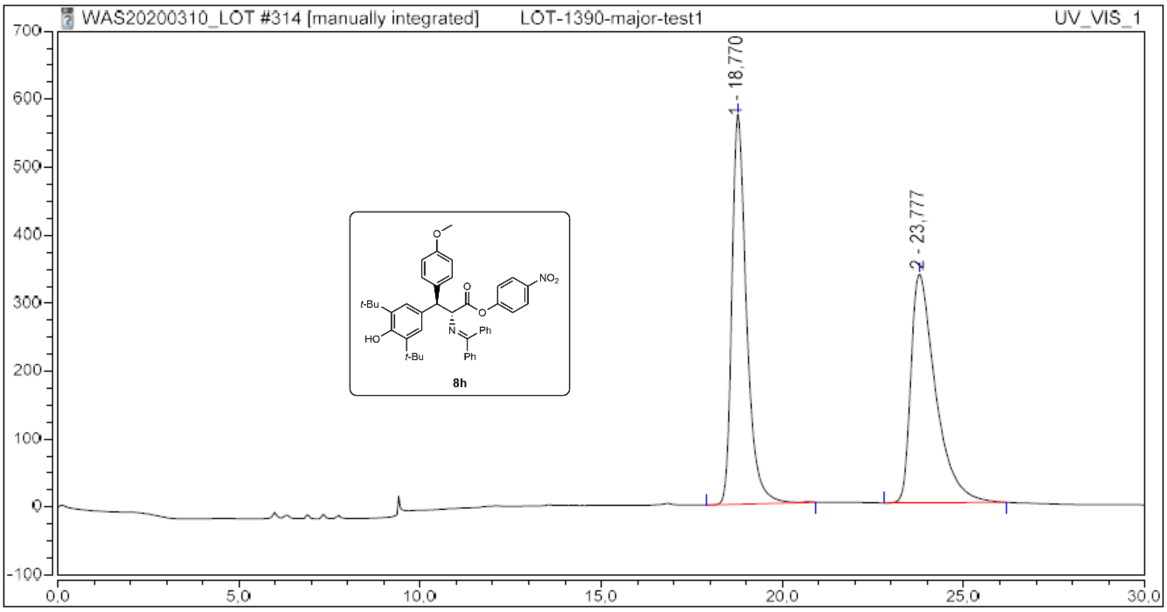


Integration Results

| No. | Peak Name | Retention Time min | Area mAU*min | Height mAU | Relative Area % | Relative Height % | Amount n.a. |
|---|---|---|---|---|---|---|---|
| 1 | | 18,770 | 269,064 | 573,780 | 50,31 | 63,02 | n.a. |
| 2 | | 23,777 | 265,730 | 336,634 | 49,69 | 36,98 | n.a. |
| | | | **534,794** | **910,414** | **100,00** | **100,00** | |

Chromatogram

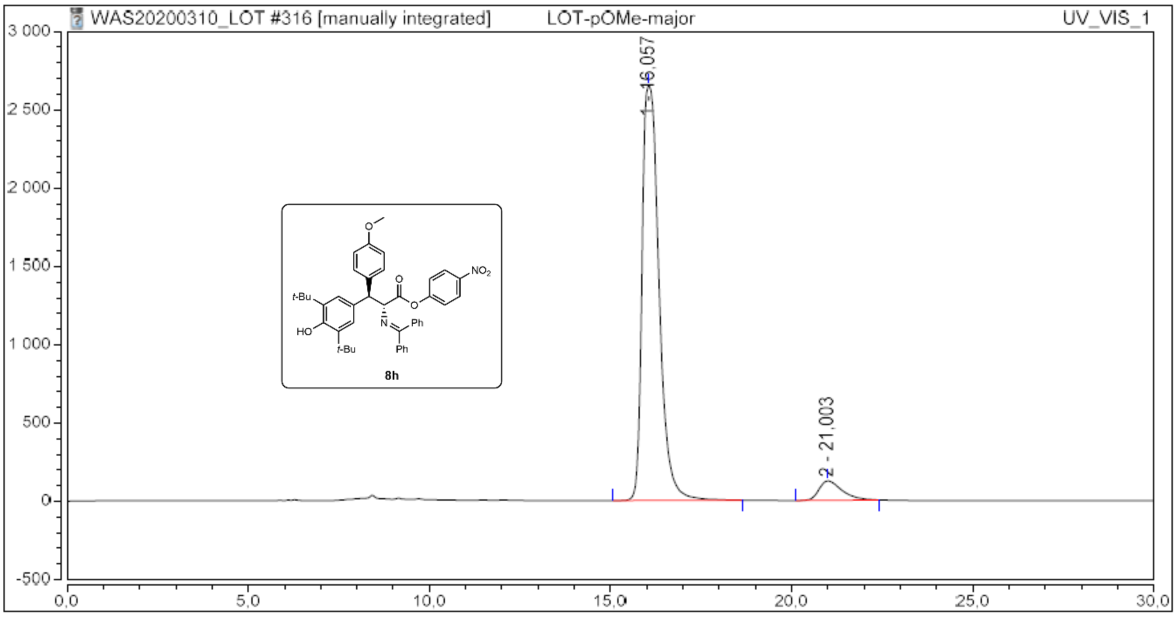


Integration Results

| No. | Peak Name | Retention Time min | Area mAU*min | Height mAU | Relative Area % | Relative Height % | Amount n.a. |
|---|---|---|---|---|---|---|---|
| 1 | | 16,057 | 1432,964 | 2650,227 | 94,05 | 95,55 | n.a. |
| 2 | | 21,003 | 90,586 | 123,509 | 5,95 | 4,45 | n.a. |
| | | | **1523,549** | **2773,736** | **100,00** | **100,00** | |



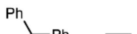

**Minor diastereomer:**

**Chromatogram**

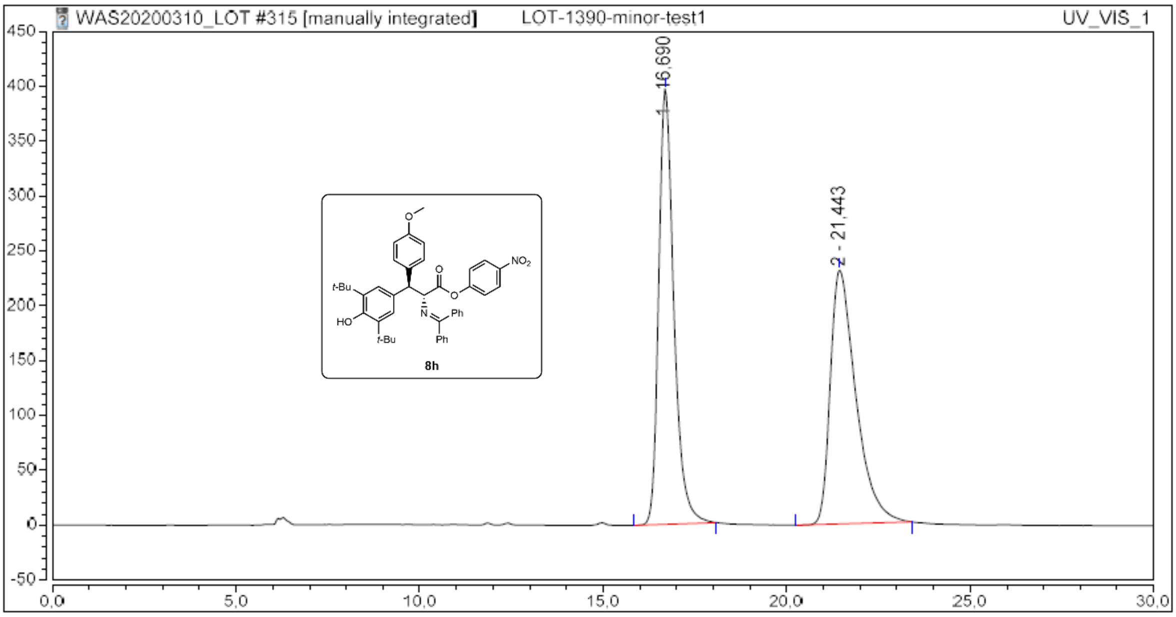


**Integration Results**

| No. | Peak Name | Retention Time min | Area mAU*min | Height mAU | Relative Area % | Relative Height % | Amount n.a. |
|---|---|---|---|---|---|---|---|
| 1 | | 16,690 | 190,391 | 397,062 | 50,48 | 63,17 | n.a. |
| 2 | | 21,443 | 186,788 | 231,471 | 49,52 | 36,83 | n.a. |
| | | | **377,179** | **628,534** | **100,00** | **100,00** | |

**Chromatogram**

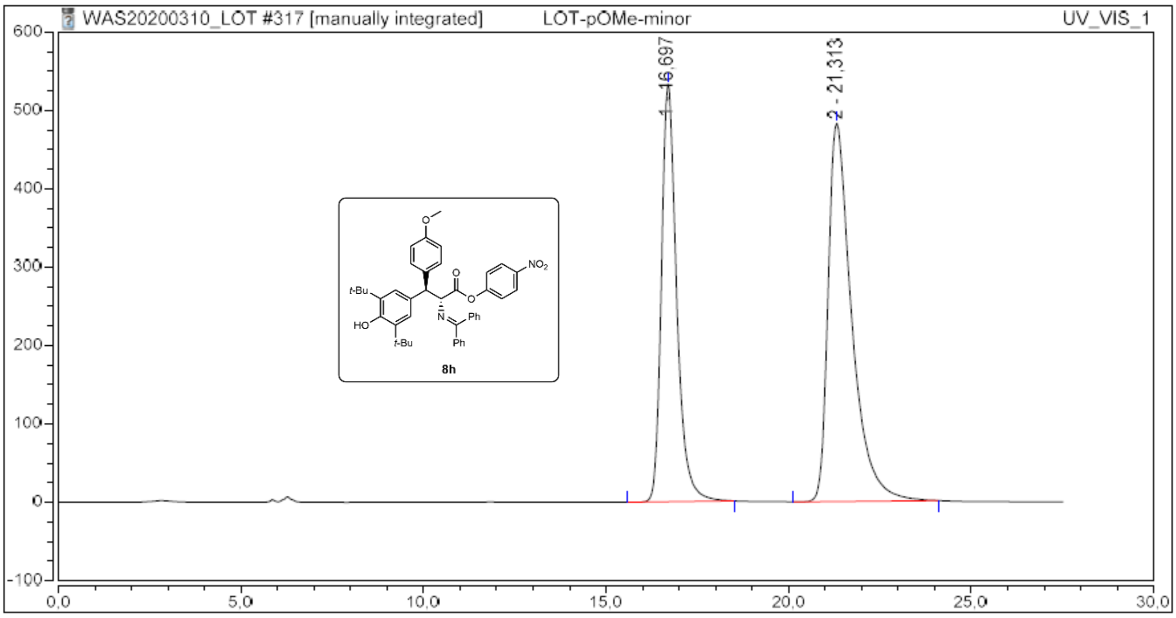


**Integration Results**

| No. | Peak Name | Retention Time min | Area mAU*min | Height mAU | Relative Area % | Relative Height % | Amount n.a. |
|---|---|---|---|---|---|---|---|
| 1 | | 16,697 | 257,975 | 533,049 | 41,61 | 52,47 | n.a. |
| 2 | | 21,313 | 362,063 | 482,849 | 58,39 | 47,53 | n.a. |
| | | | **620,038** | **1015,898** | **100,00** | **100,00** | |

**4-nitrophenyl 3-(3,5-di-tert-butyl-4-hydroxyphenyl)-3-(4-(dimethylamino)phenyl)-2-((diphenylmethylene)amino)propanoate 8i**

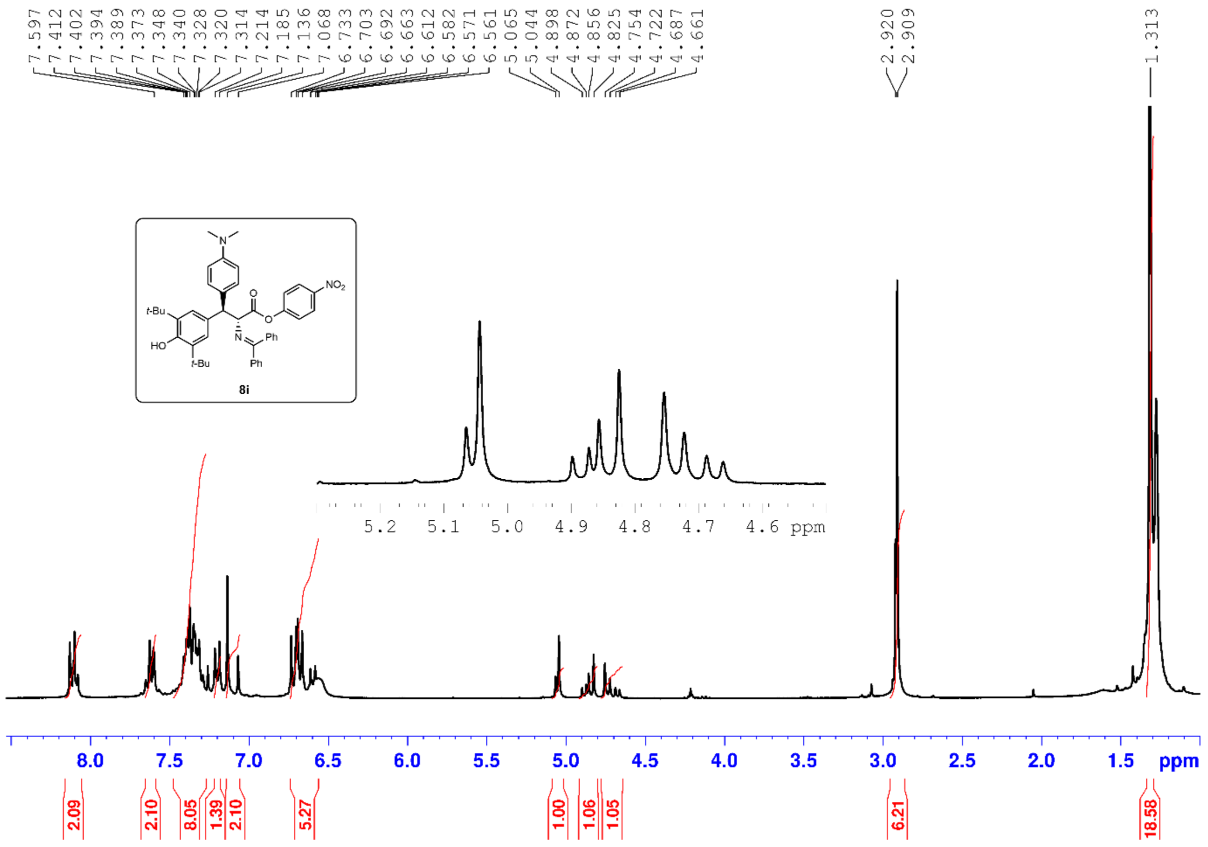


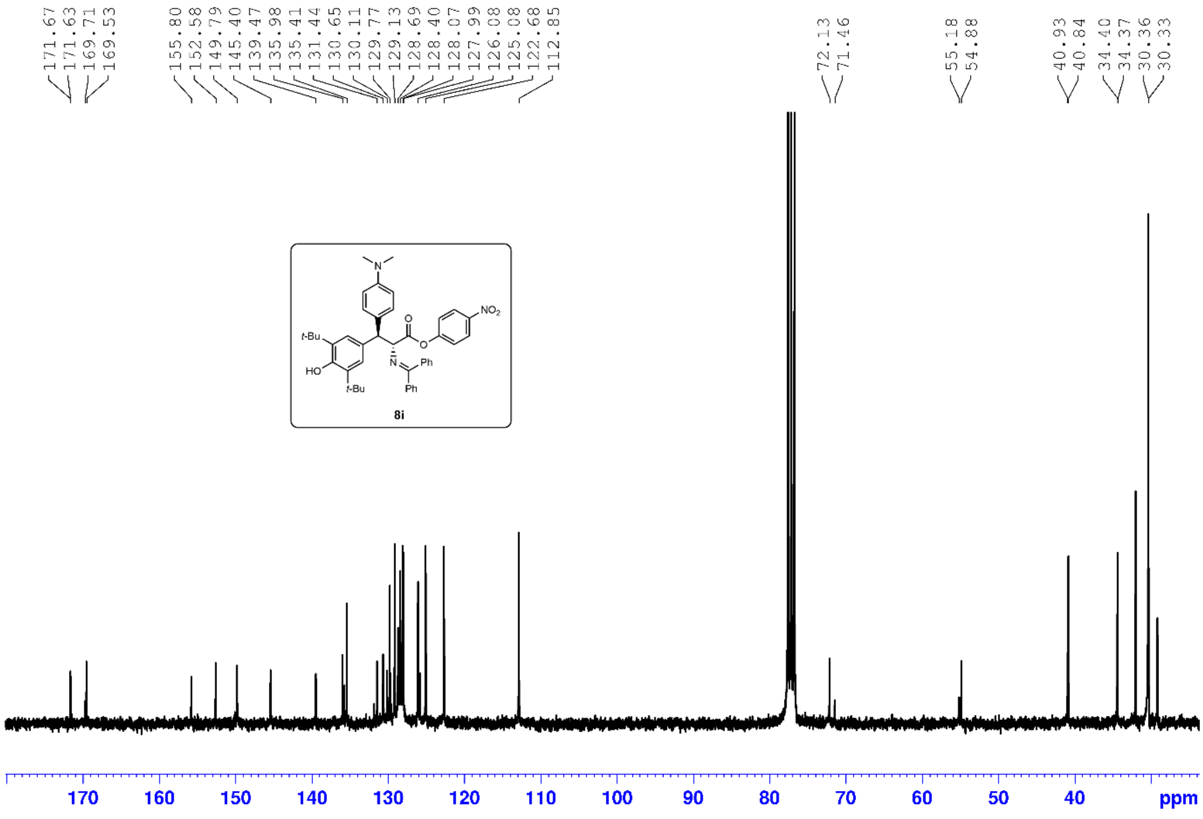

**Preparative HPLC** (Separation of the diastereomers of **8i**)

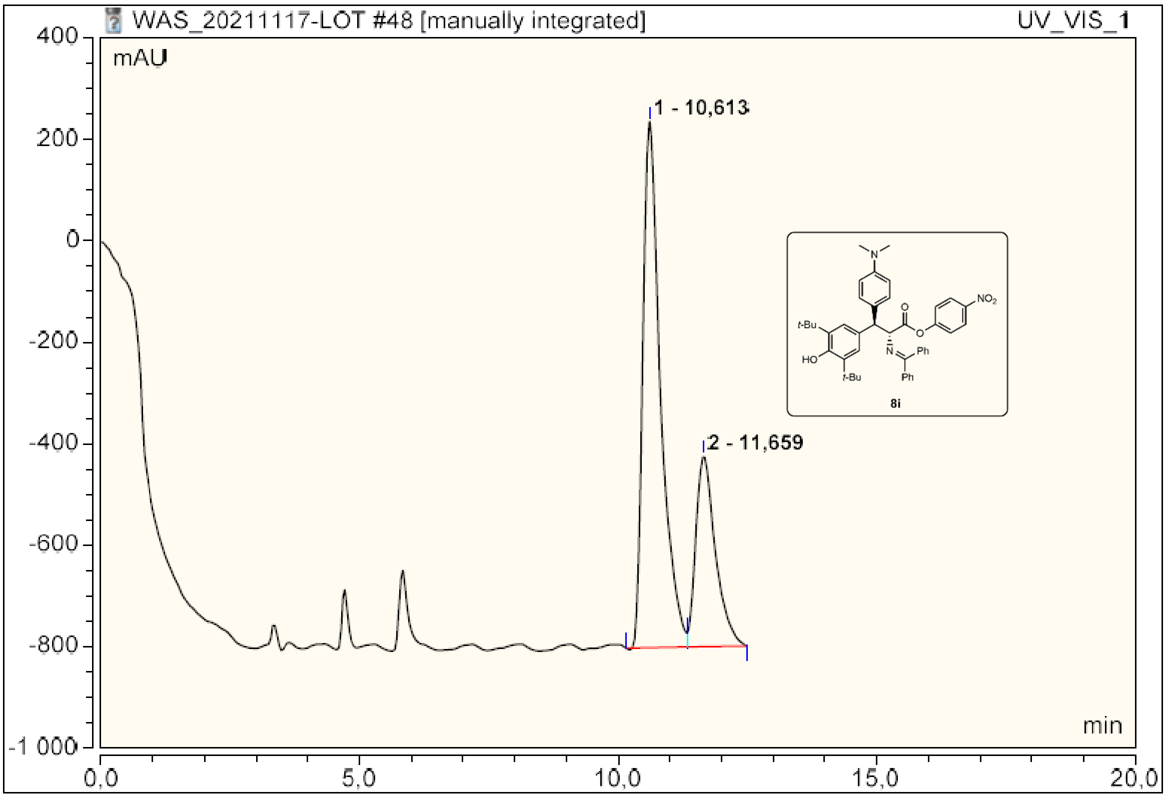


| No. | Ret.Time<br>min | Peak Name | Height<br>mAU | Area<br>mAU*min | Rel.Area<br>% | Amount<br>n.a. | Type |
|---|---|---|---|---|---|---|---|
| 1 | 10,61 | | 1036,479 | 408,462 | 71,36 | n.a. | BM * |
| 2 | 11,66 | | 376,518 | 163,909 | 28,64 | n.a. | MB* |
| Total: | | | 1412,997 | 572,372 | 100,00 | 0,000 | |

**Major diastereomer**

**Chromatogram**

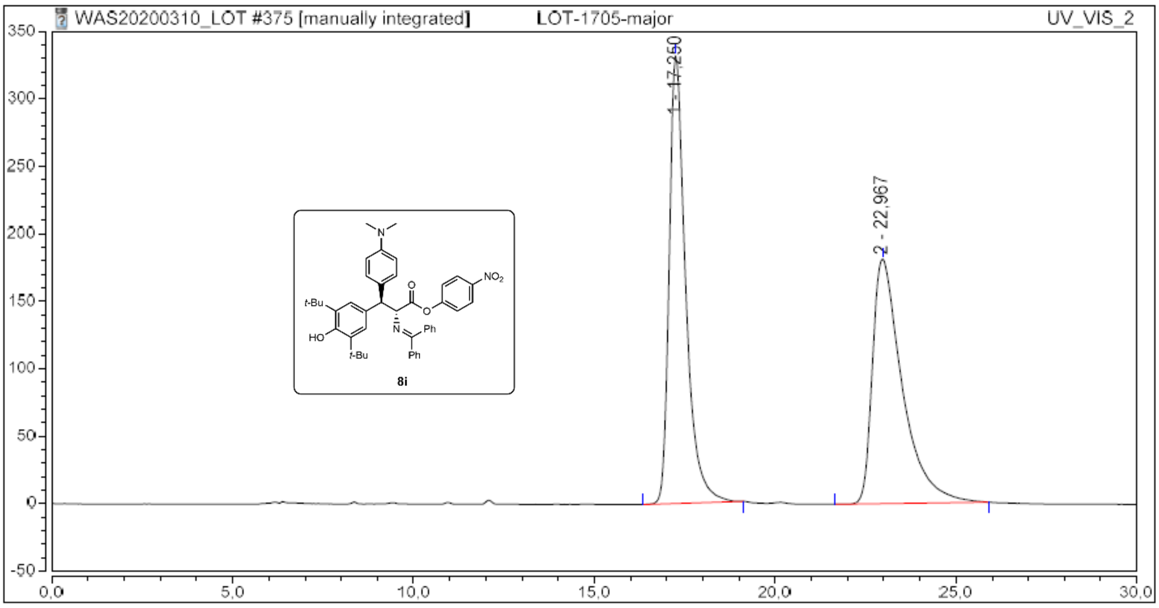


**Integration Results**

| No. | Peak Name | Retention Time min | Area mAU*min | Height mAU | Relative Area % | Relative Height % | Amount n.a. |
|---|---|---|---|---|---|---|---|
| 1 | | 17,250 | 174,989 | 332,065 | 50,26 | 64,67 | n.a. |
| 2 | | 22,967 | 173,210 | 181,442 | 49,74 | 35,33 | n.a. |
| | | | **348,198** | **513,507** | **100,00** | **100,00** | |

**Chromatogram**

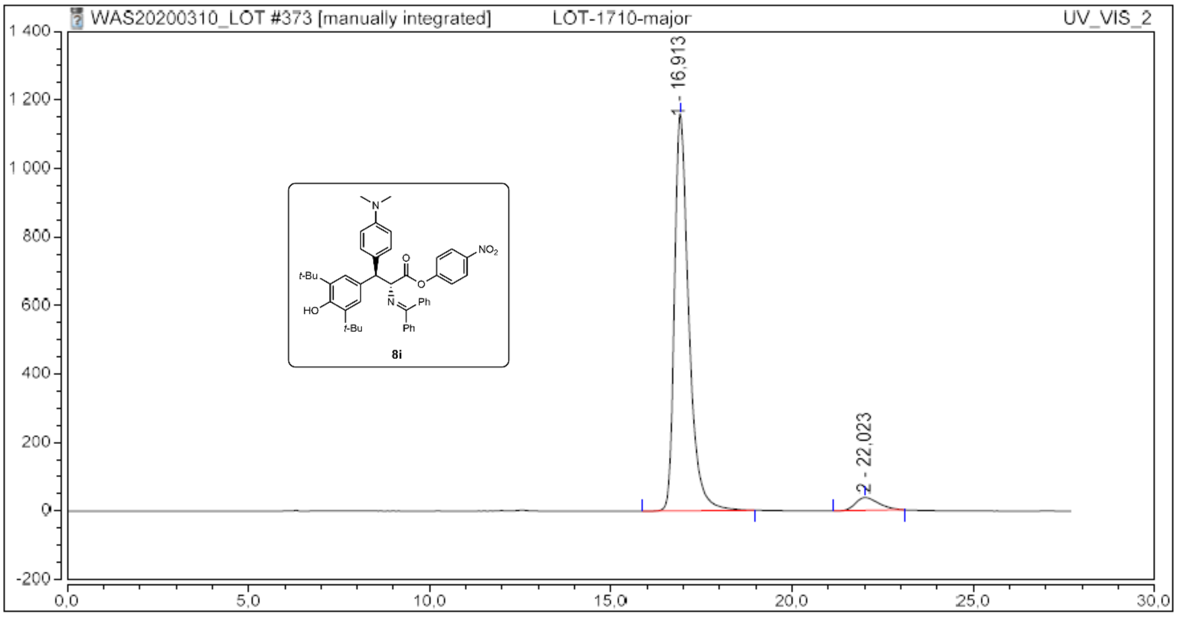


**Integration Results**

| No. | Peak Name | Retention Time min | Area mAU*min | Height mAU | Relative Area % | Relative Height % | Amount n.a. |
|---|---|---|---|---|---|---|---|
| 1 | | 16,913 | 529,373 | 1158,953 | 94,97 | 96,85 | n.a. |
| 2 | | 22,023 | 28,040 | 37,720 | 5,03 | 3,15 | n.a. |
| | | | **557,412** | **1196,673** | **100,00** | **100,00** | |

**Minor diastereomer**

**Chromatogram**

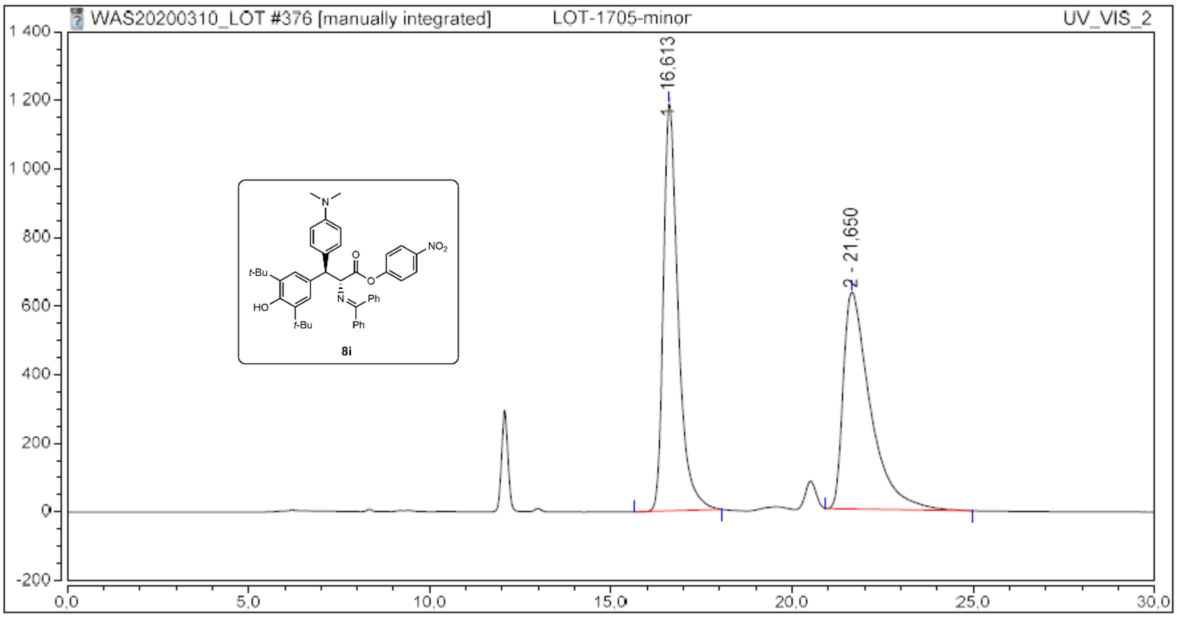


**Integration Results**

| No. | Peak Name | Retention Time min | Area mAU*min | Height mAU | Relative Area % | Relative Height % | Amount n.a. |
|---|---|---|---|---|---|---|---|
| 1 | | 16,613 | 561,922 | 1188,205 | 50,63 | 65,27 | n.a. |
| 2 | | 21,650 | 547,883 | 632,336 | 49,37 | 34,73 | n.a. |
| | | | **1109,805** | **1820,541** | **100,00** | **100,00** | |

**Chromatogram**

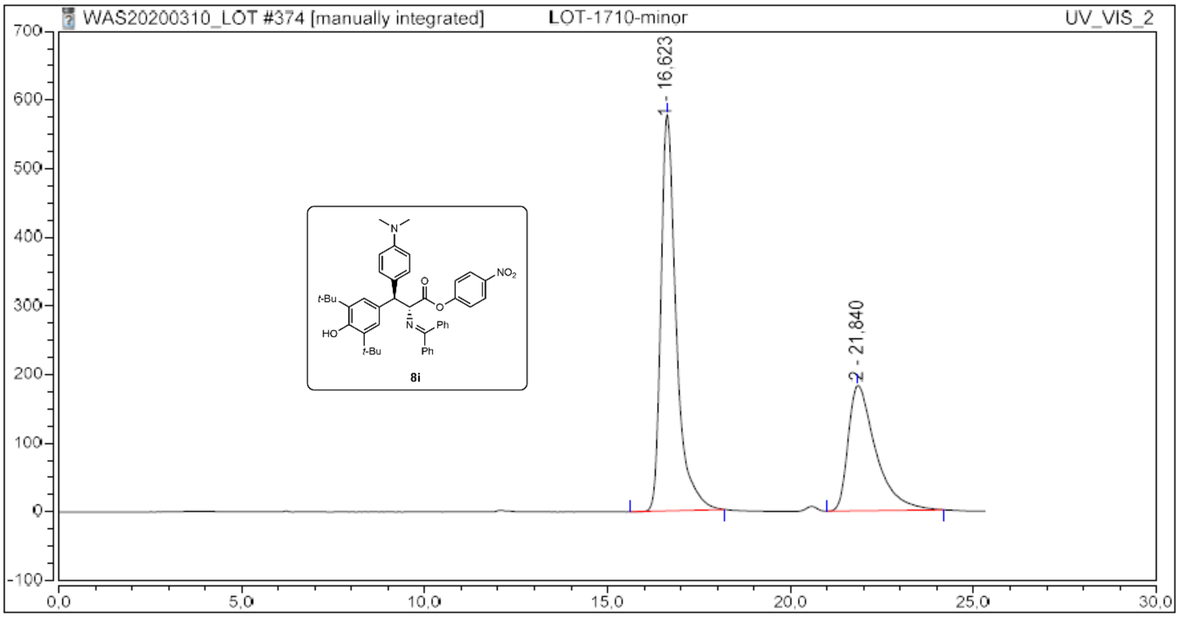


**Integration Results**

| No. | Peak Name | Retention Time min | Area mAU*min | Height mAU | Relative Area % | Relative Height % | Amount n.a. |
|---|---|---|---|---|---|---|---|
| 1 | | 16,623 | 280,271 | 577,610 | 63,72 | 75,97 | n.a. |
| 2 | | 21,840 | 159,600 | 182,677 | 36,28 | 24,03 | n.a. |
| | | | **439,872** | **760,287** | **100,00** | **100,00** | |

**4-nitrophenyl 3-(3,5-di-tert-butyl-4-hydroxyphenyl)-2-((diphenylmethylene)amino)-3-(4-(trifluoromethyl)phenyl)propanoate 8j** and the corresponding morpholine amide **9j**

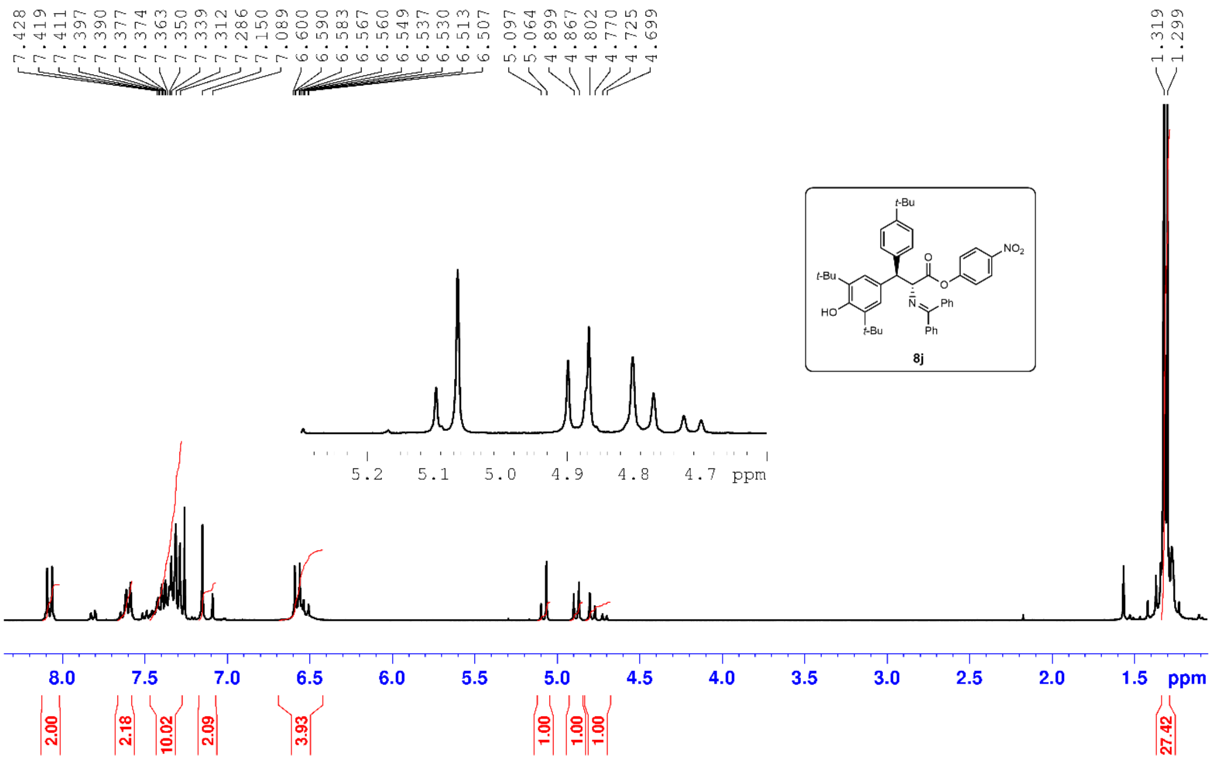


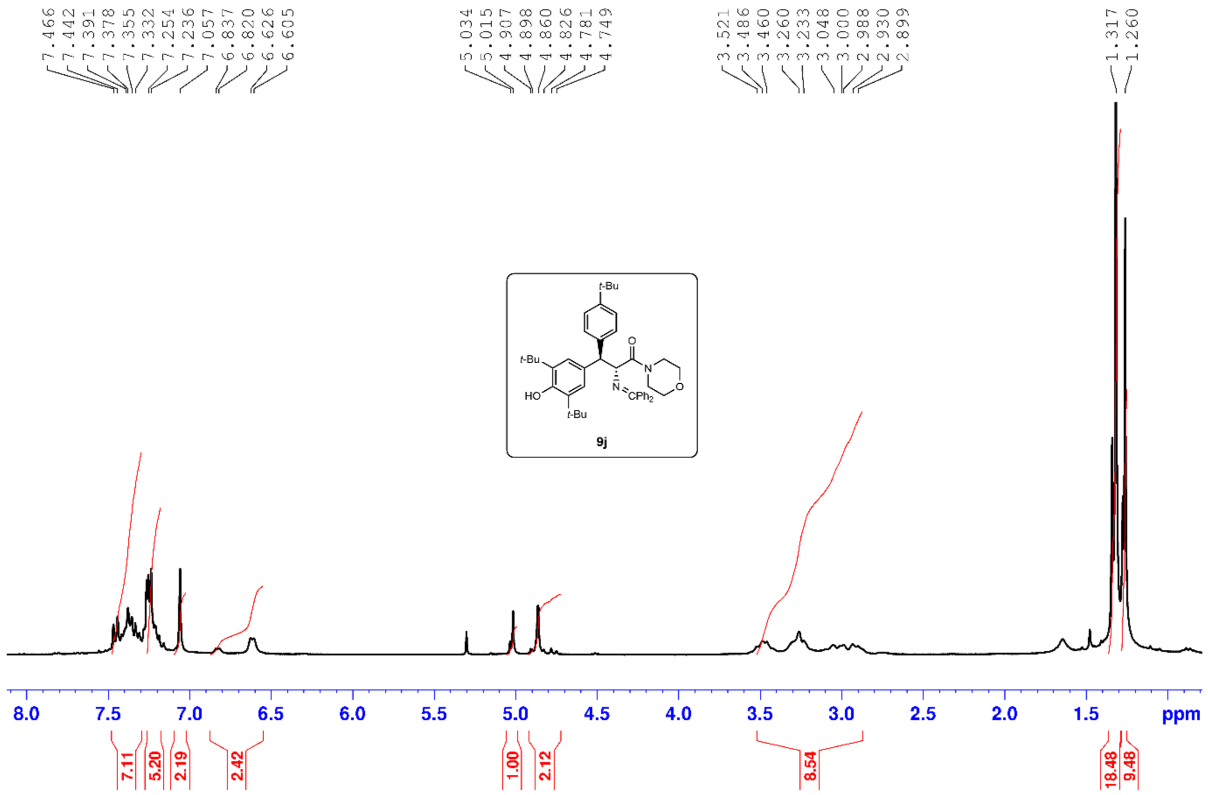

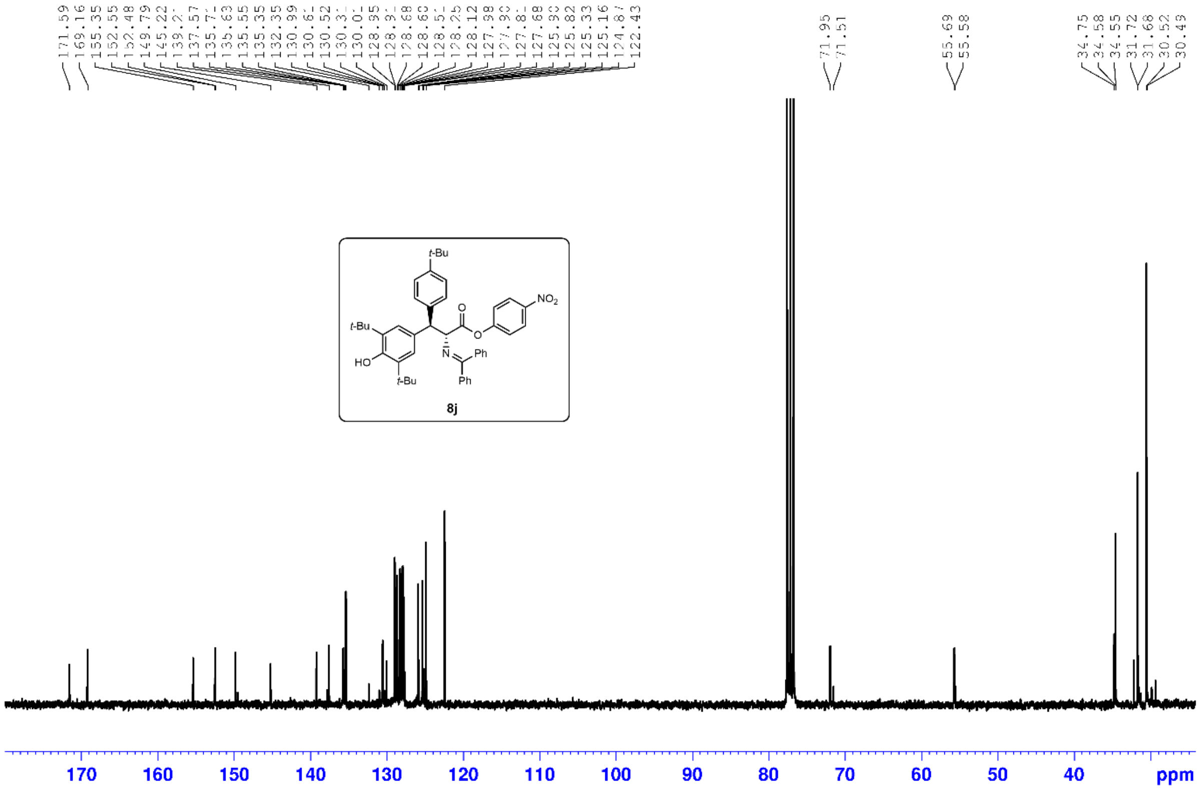
t-Bu
NO2
t-Bu
HO
N
Ph
Ph
t-Bu
8j
170 160 150 140 130 120 110 100 90 80 70 60 50 40 ppm

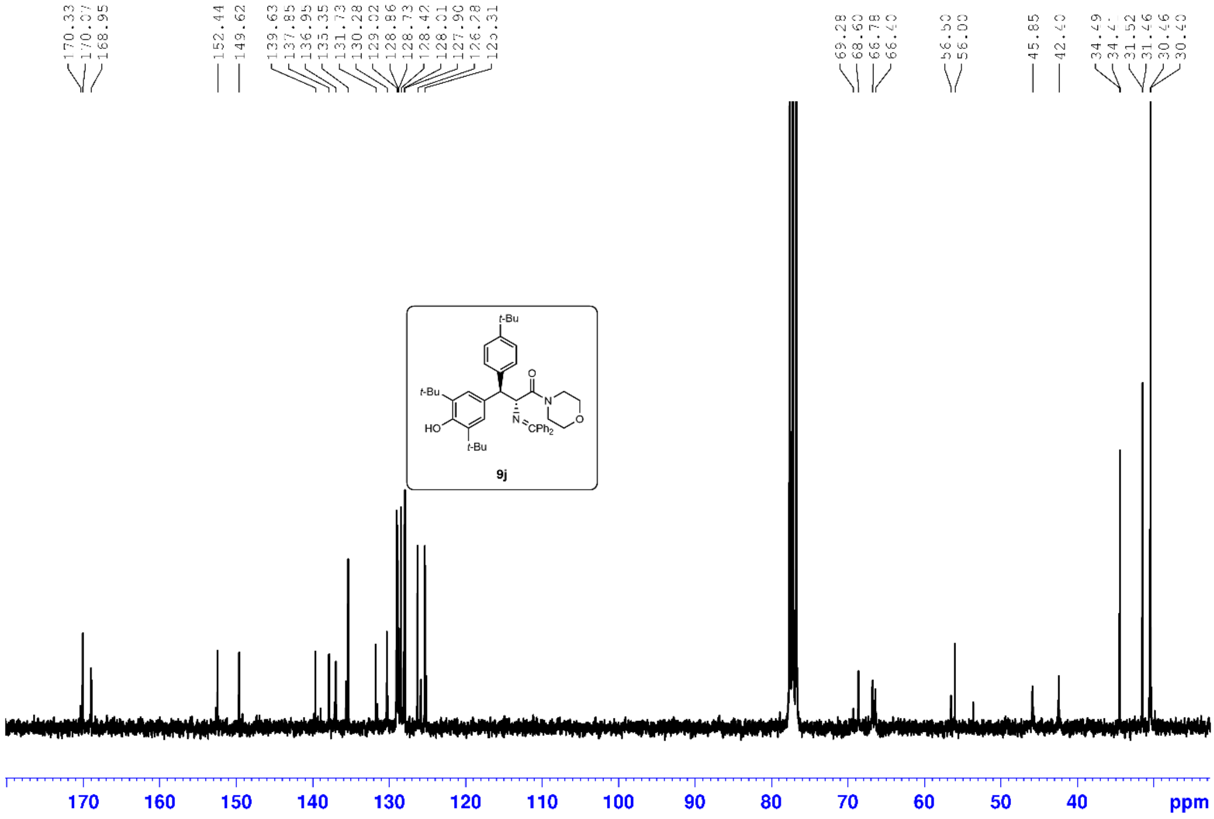
t-Bu
t-Bu
HO
N
CPh2
O
t-Bu
9j
170 160 150 140 130 120 110 100 90 80 70 60 50 40 ppm

## Chromatogram

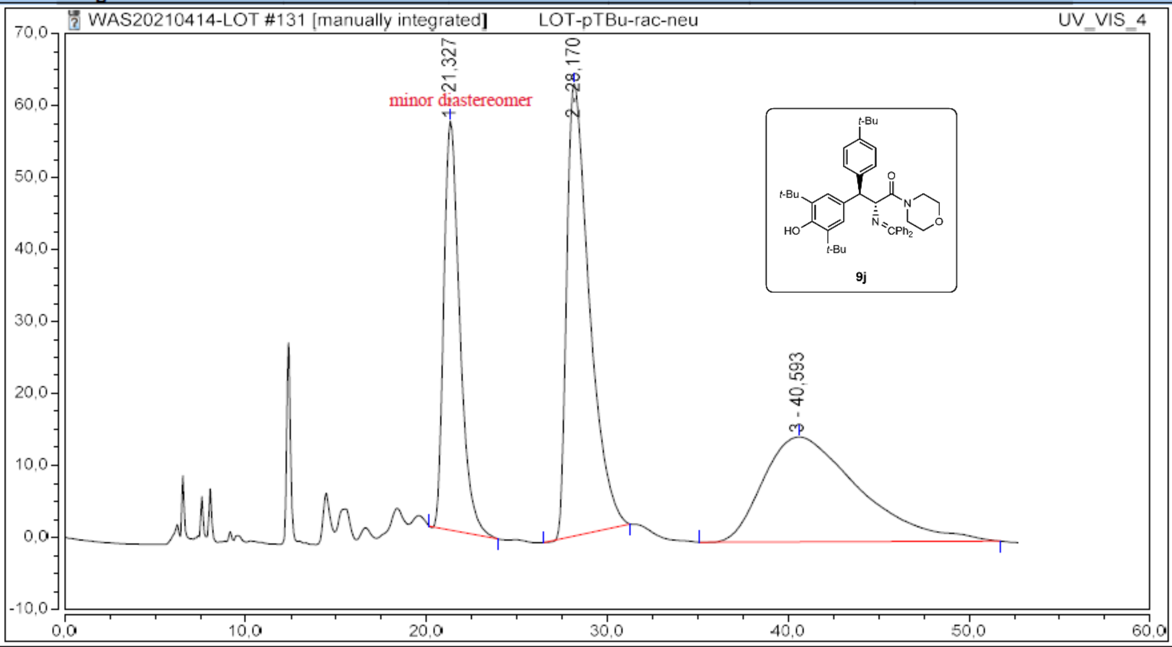


## Integration Results

| No. | Peak Name | Retention Time min | Area mAU*min | Height mAU | Relative Area % | Relative Height % | Amount n.a. |
|---|---|---|---|---|---|---|---|
| 1 | | 21,327 | 58,129 | 56,866 | 24,34 | 42,35 | n.a. |
| 2 | | 28,170 | 91,501 | 62,847 | 38,32 | 46,80 | n.a. |
| 3 | | 40,593 | 89,153 | 14,577 | 37,34 | 10,86 | n.a. |
| **Total:** | | | **238,783** | **134,290** | **100,00** | **100,00** | |

## Chromatogram

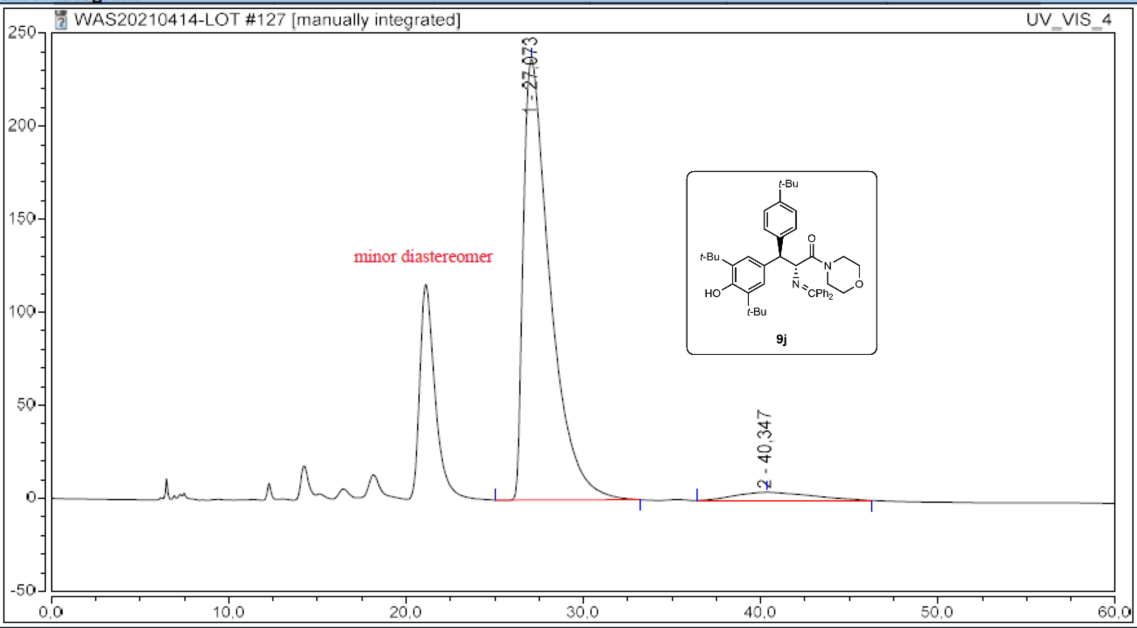


## Integration Results

| No. | Peak Name | Retention Time min | Area mAU*min | Height mAU | Relative Area % | Relative Height % | Amount n.a. |
|---|---|---|---|---|---|---|---|
| 1 | | 27,073 | 415,016 | 237,152 | 95,02 | 98,17 | n.a. |
| 2 | | 40,347 | 21,731 | 4,418 | 4,98 | 1,83 | n.a. |
| **Total:** | | | **436,747** | **241,569** | **100,00** | **100,00** | |

**4-nitrophenyl 3-(3,5-di-tert-butyl-4-hydroxyphenyl)-2-((diphenylmethylene)amino)-3-(4-(trifluoromethyl)phenyl)propanoate 8k** and the corresponding morpholine amide **9k**

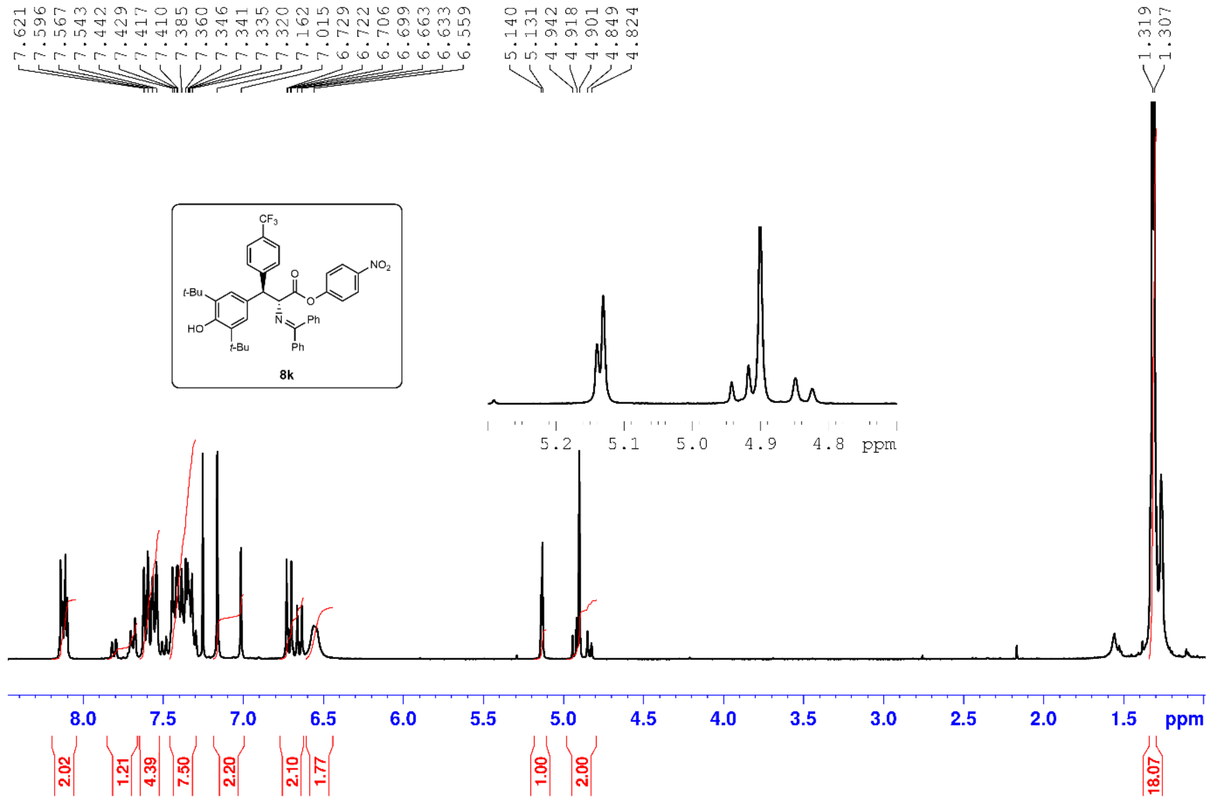


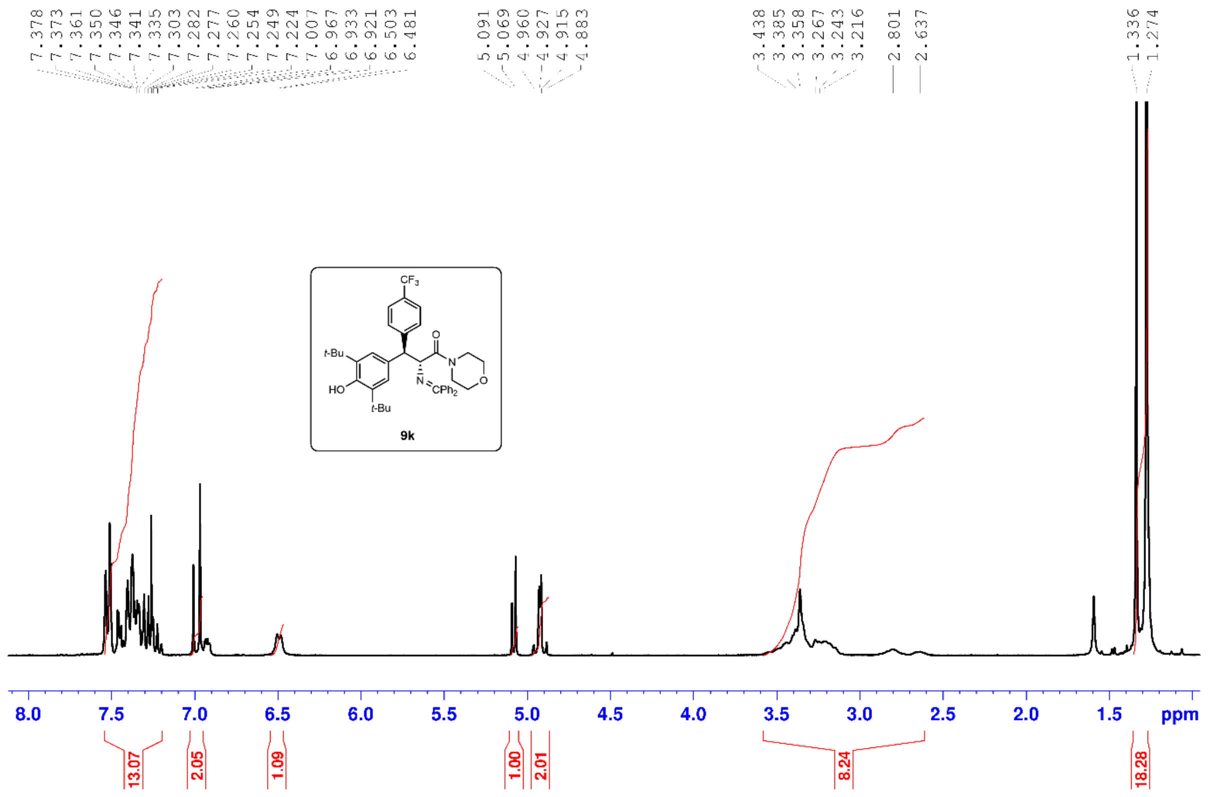

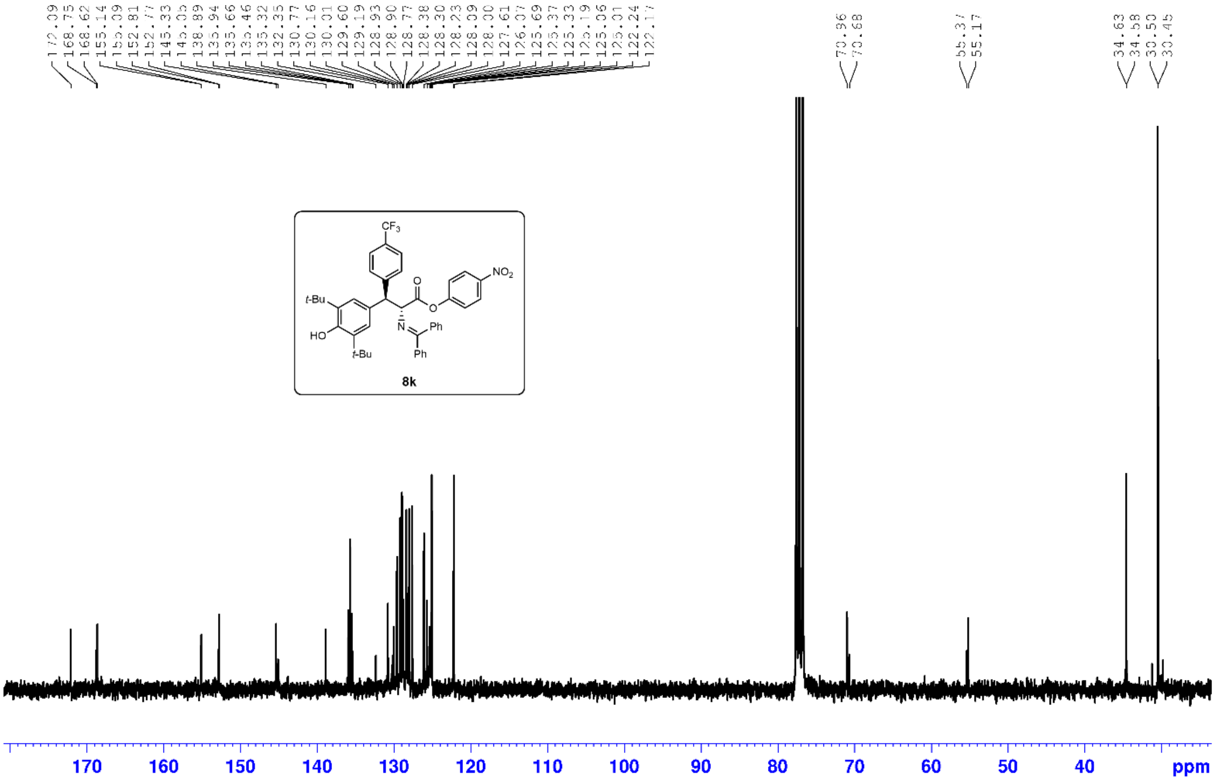
CF3
NO2
t-Bu
HO
t-Bu
Ph
Ph
8k
ppm

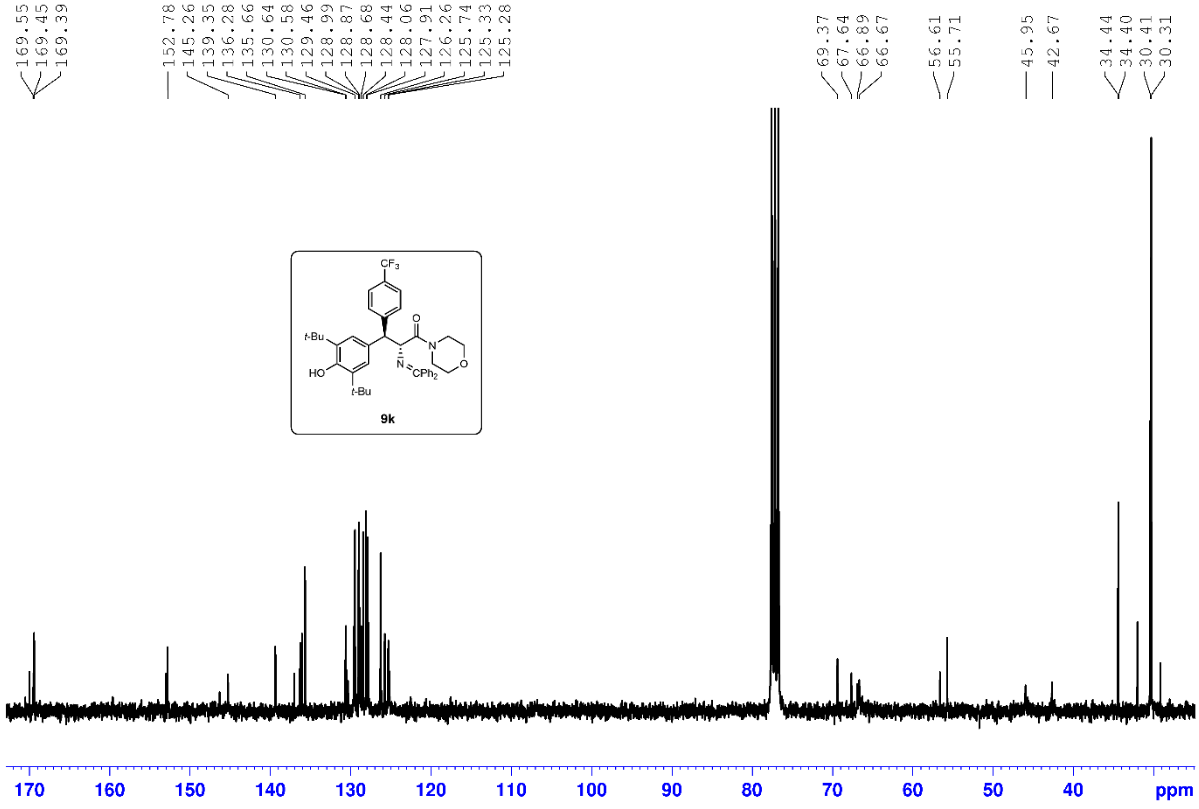
169.55
169.45
169.39
152.78
145.26
139.35
136.28
135.66
130.64
130.58
129.46
128.99
128.87
128.68
128.44
128.06
127.91
126.26
125.74
125.33
125.28
69.37
67.64
66.89
66.67
56.61
55.71
45.95
42.67
34.44
34.40
30.41
30.31
CF3
t-Bu
HO
t-Bu
CPh2
9k
ppm

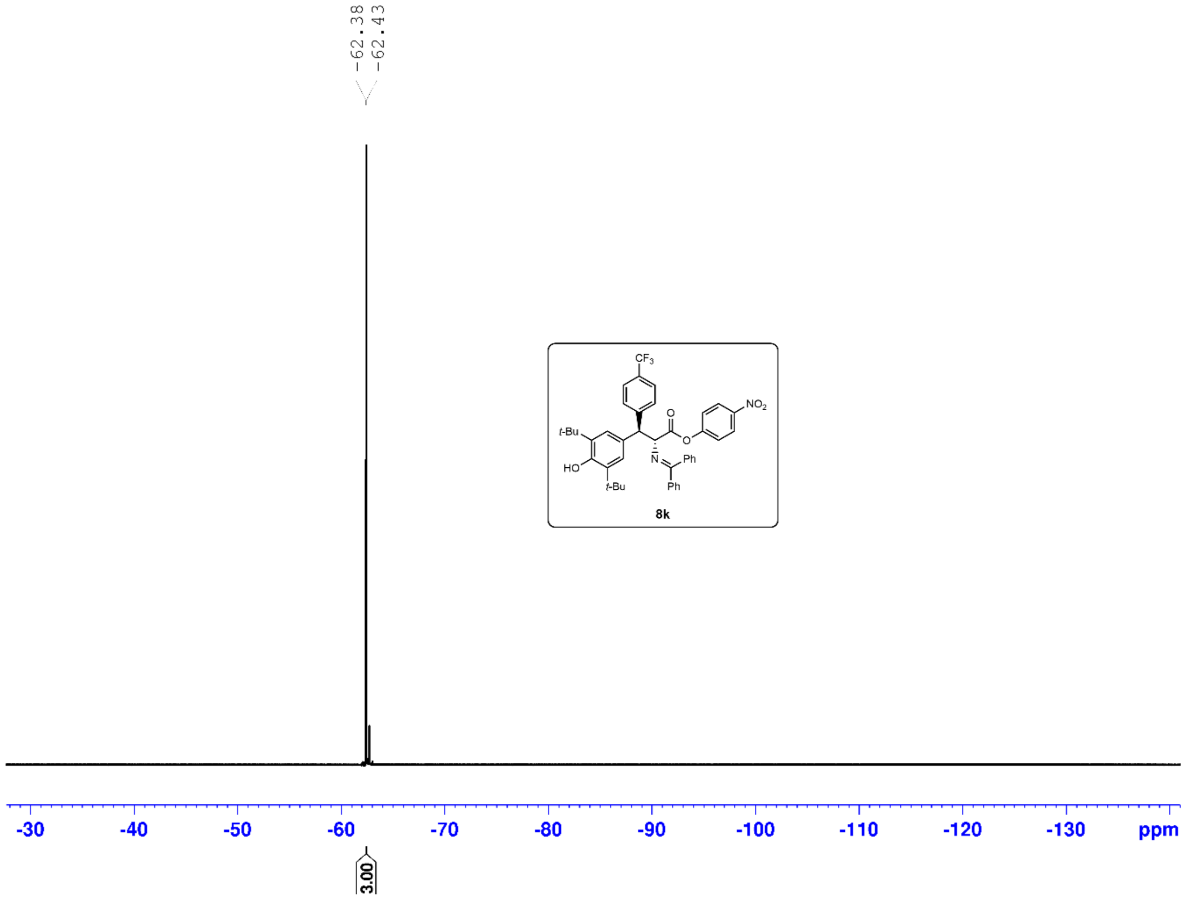
-62.38
-62.43
CF3
NO2
t-Bu
HO
t-Bu
O
O
N
Ph
Ph
8k
-30
-40
-50
-60
-70
-80
-90
-100
-110
-120
-130
ppm
3.00

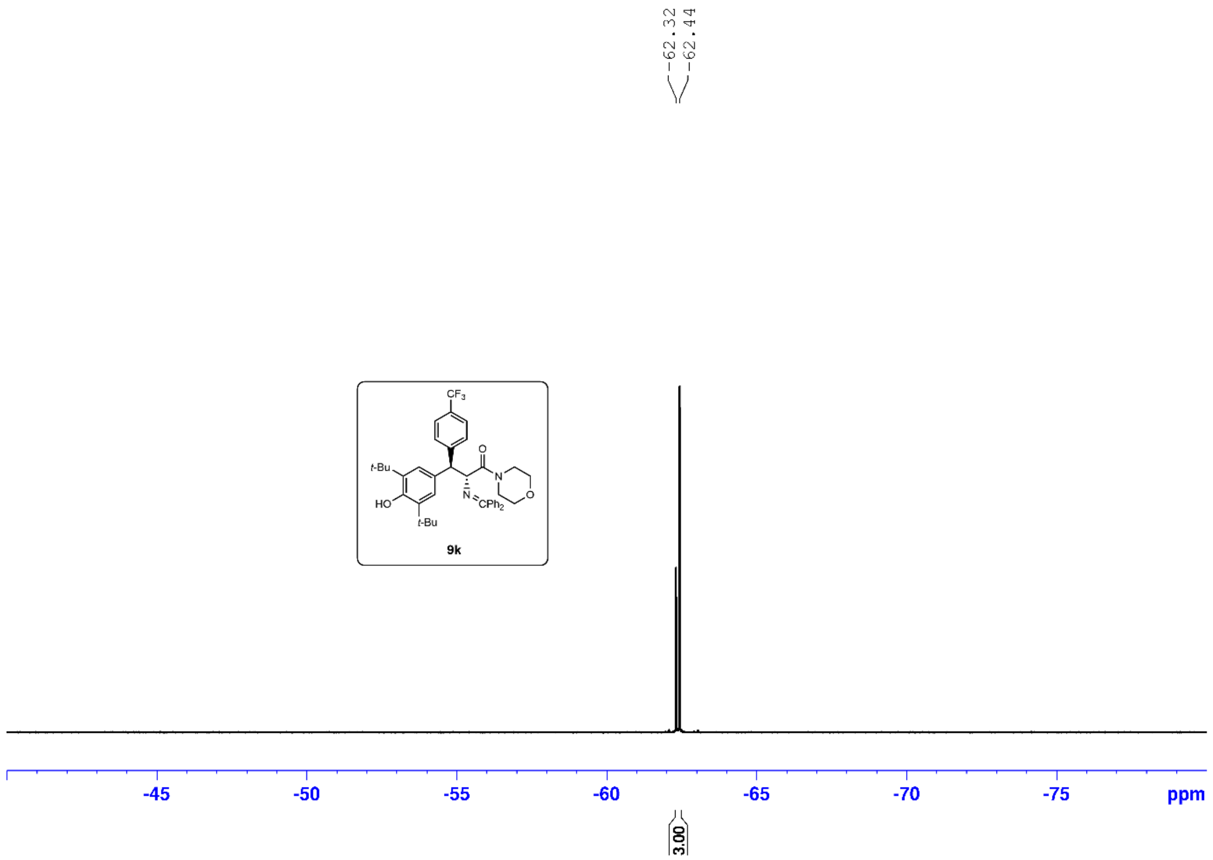
-62.32
-62.44
CF3
t-Bu
HO
t-Bu
O
N
O
N
CPh2
9k
-45
-50
-55
-60
-65
-70
-75
ppm
3.00

## Chromatogram

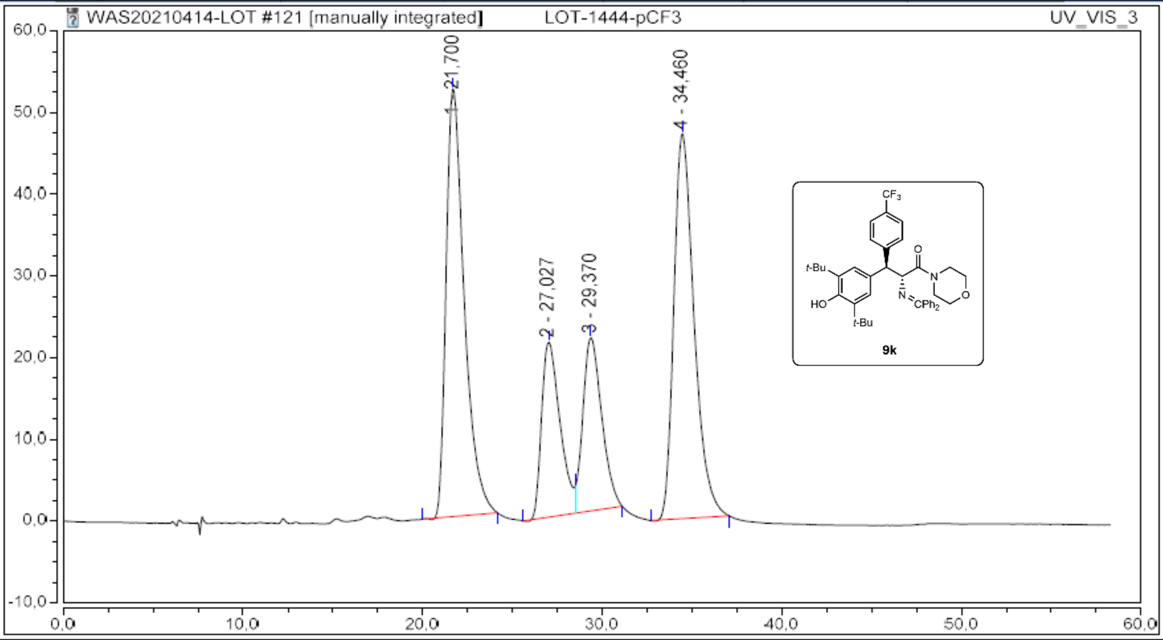


## Integration Results

| No. | Peak Name | Retention Time<br>min | Area<br>mAU*min | Height<br>mAU | Relative Area<br>% | Relative Height<br>% | Amount<br>n.a. |
|---|---|---|---|---|---|---|---|
| 1 | | 21,700 | 61,224 | 52,374 | 35,20 | 36,84 | n.a. |
| 2 | | 27,027 | 25,456 | 21,432 | 14,63 | 15,07 | n.a. |
| 3 | | 29,370 | 25,770 | 21,227 | 14,81 | 14,93 | n.a. |
| 4 | | 34,460 | 61,506 | 47,137 | 35,36 | 33,16 | n.a. |
| **Total:** | | | **173,956** | **142,169** | **100,00** | **100,00** | |

## Chromatogram

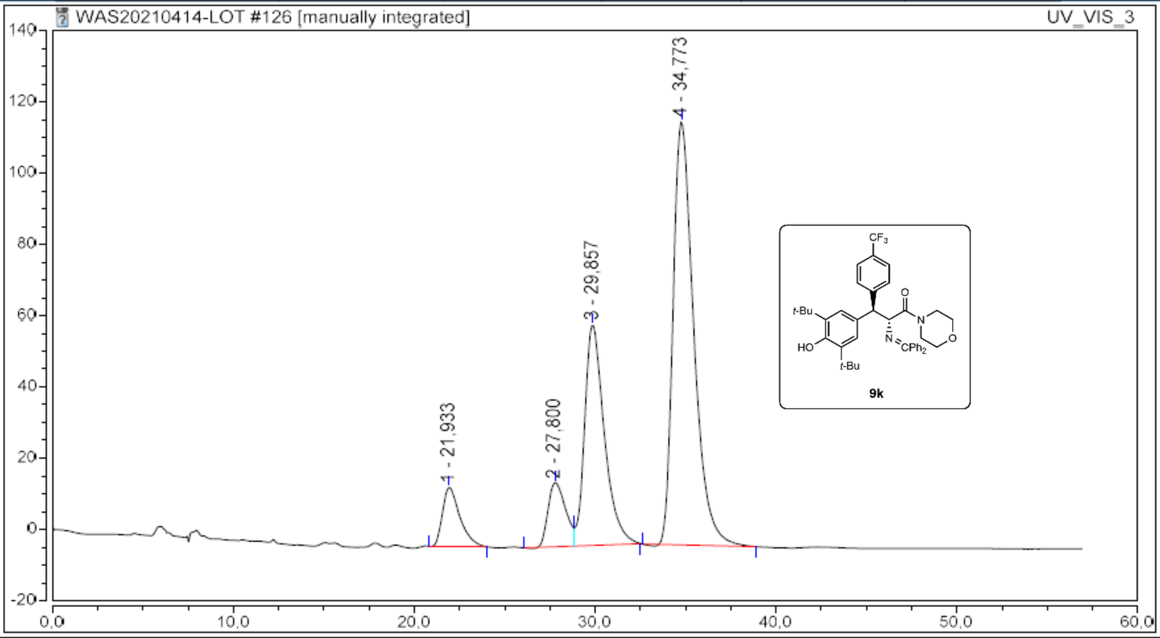


## Integration Results

| No. | Peak Name | Retention Time<br>min | Area<br>mAU*min | Height<br>mAU | Relative Area<br>% | Relative Height<br>% | Amount<br>n.a. |
|---|---|---|---|---|---|---|---|
| 1 | | 21,933 | 18,462 | 16,588 | 6,74 | 7,70 | n.a. |
| 2 | | 27,800 | 19,865 | 17,994 | 7,26 | 8,35 | n.a. |
| 3 | | 29,857 | 78,279 | 61,935 | 28,59 | 28,76 | n.a. |
| 4 | | 34,773 | 157,205 | 118,852 | 57,41 | 55,19 | n.a. |
| **Total:** | | | **273,812** | **215,369** | **100,00** | **100,00** | |

**4-nitrophenyl 3-(3,5-di-tert-butyl-4-hydroxyphenyl)-2-((diphenylmethylene)amino)-3-(4-fluorophenyl)propanoate 8l** and the corresponding morpholine amide **9l**

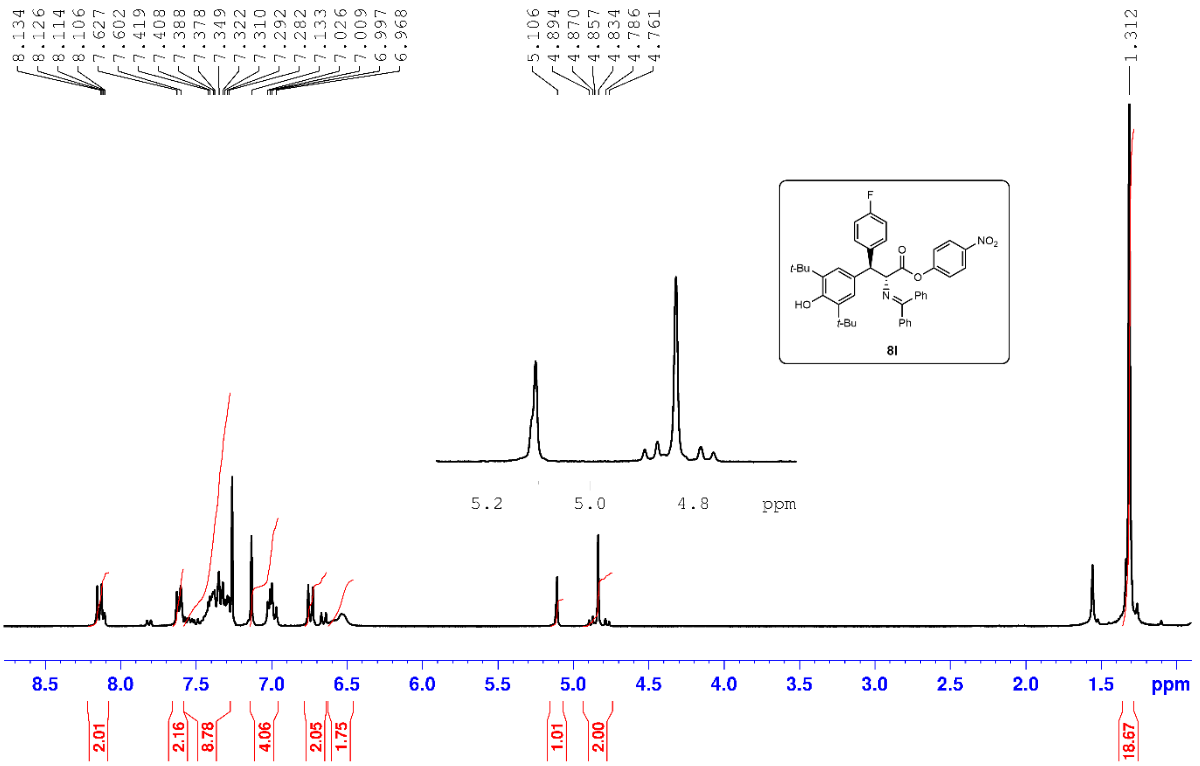

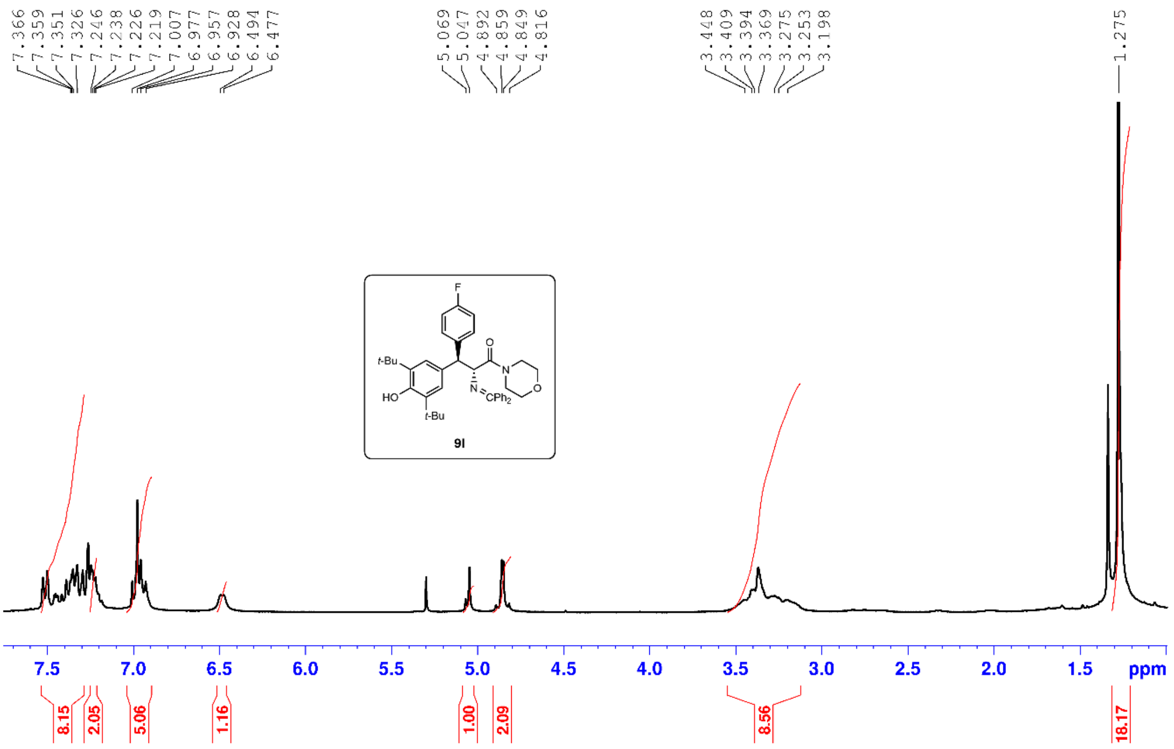

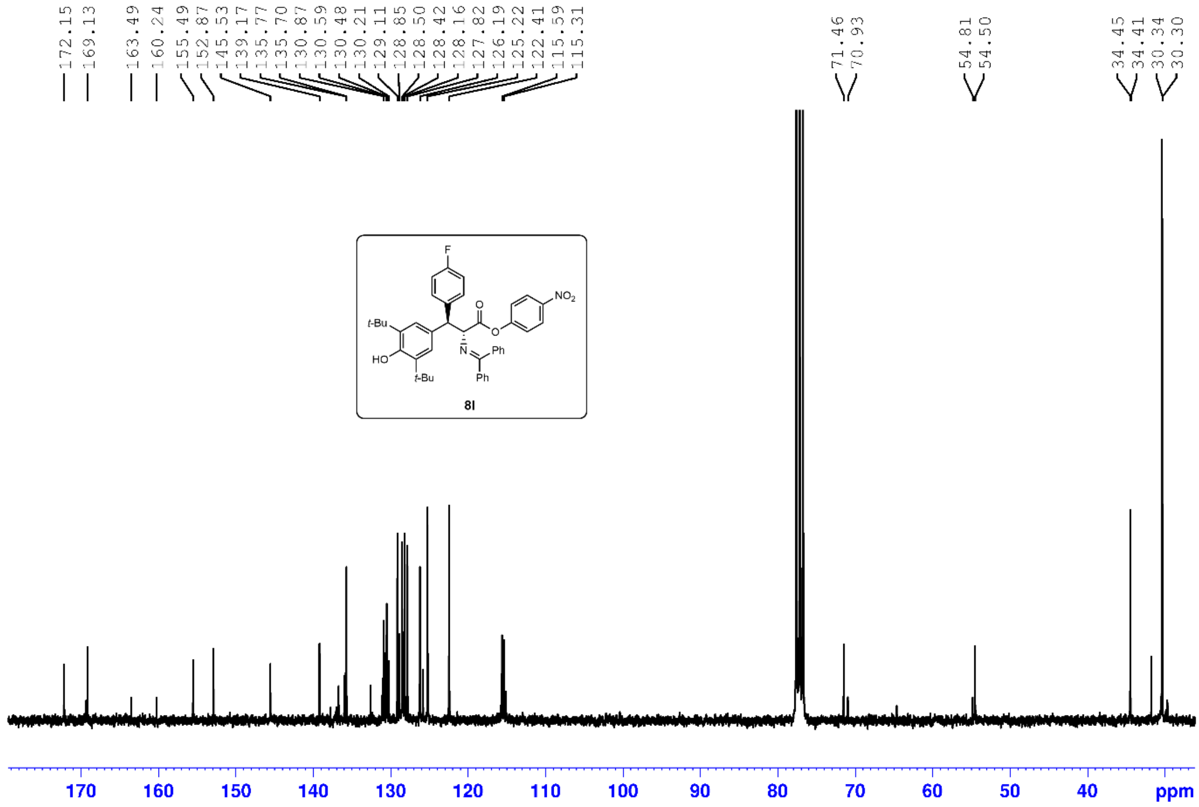
172.15
169.13
163.49
160.24
155.49
152.87
145.53
139.17
135.77
135.70
130.87
130.59
130.48
130.21
129.11
128.85
128.50
128.42
128.16
127.82
126.19
125.22
122.41
115.59
115.31
71.46
70.93
54.81
54.50
34.45
34.41
30.34
30.30
F
NO2
O
t-Bu
HO
t-Bu
N
Ph
Ph
8l
170
160
150
140
130
120
110
100
90
80
70
60
50
40
ppm

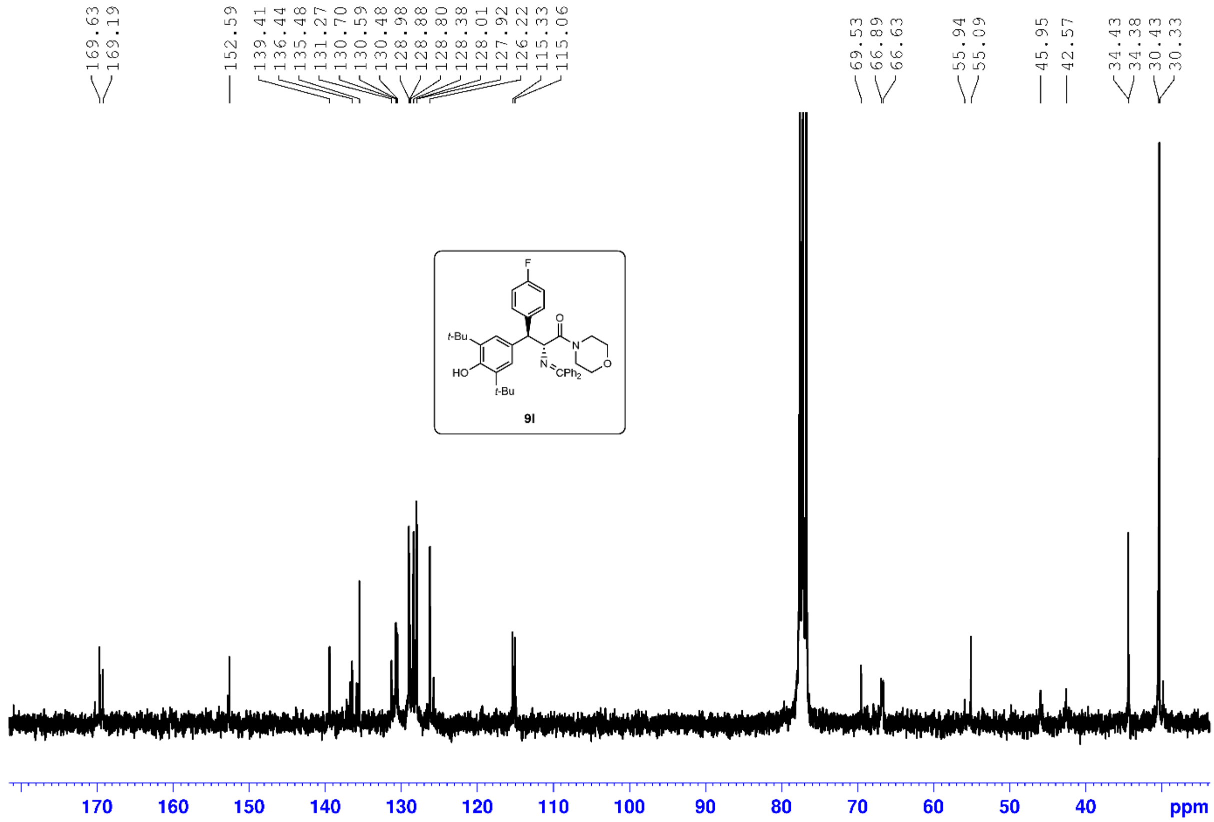
169.63
169.19
152.59
139.41
136.44
135.48
131.27
130.70
130.59
130.48
128.98
128.88
128.80
128.38
128.01
127.92
126.22
115.33
115.06
69.53
66.89
66.63
55.94
55.09
45.95
42.57
34.43
34.38
30.43
30.33
F
O
N
O
t-Bu
HO
t-Bu
N
CPh2
9l
170
160
150
140
130
120
110
100
90
80
70
60
50
40
ppm

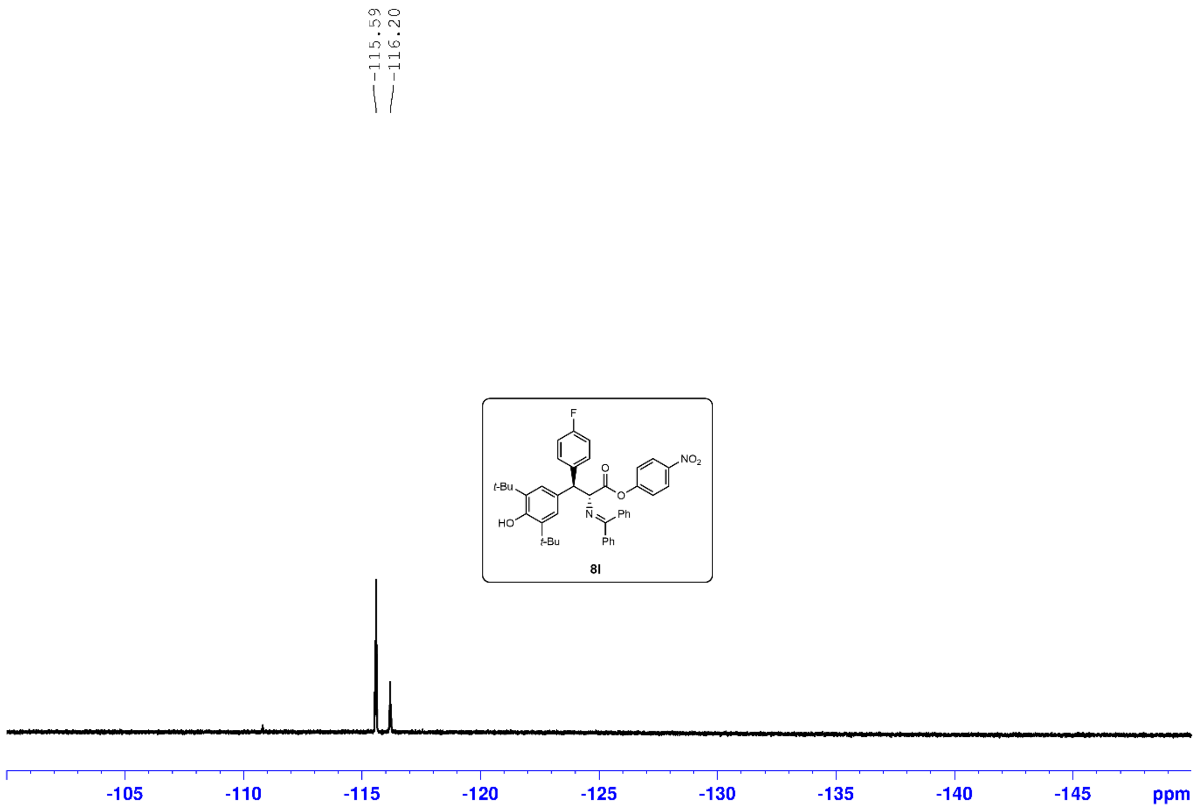
-115.59
-116.20
F
NO2
t-Bu
HO
t-Bu
O
O
N
Ph
Ph
8l
-105
-110
-115
-120
-125
-130
-135
-140
-145
ppm

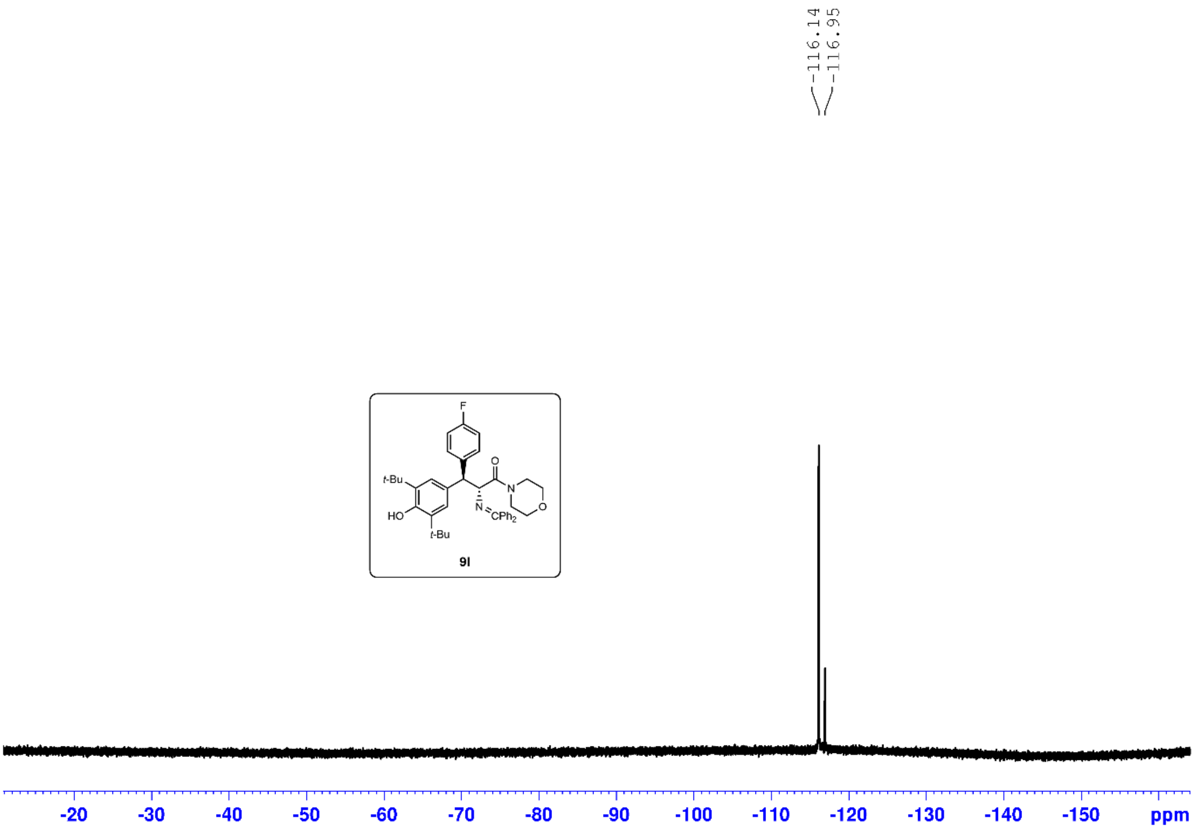
-116.14
-116.95
F
t-Bu
HO
t-Bu
O
N
O
N
CPh2
9l
-20
-30
-40
-50
-60
-70
-80
-90
-100
-110
-120
-130
-140
-150
ppm

## Chromatogram

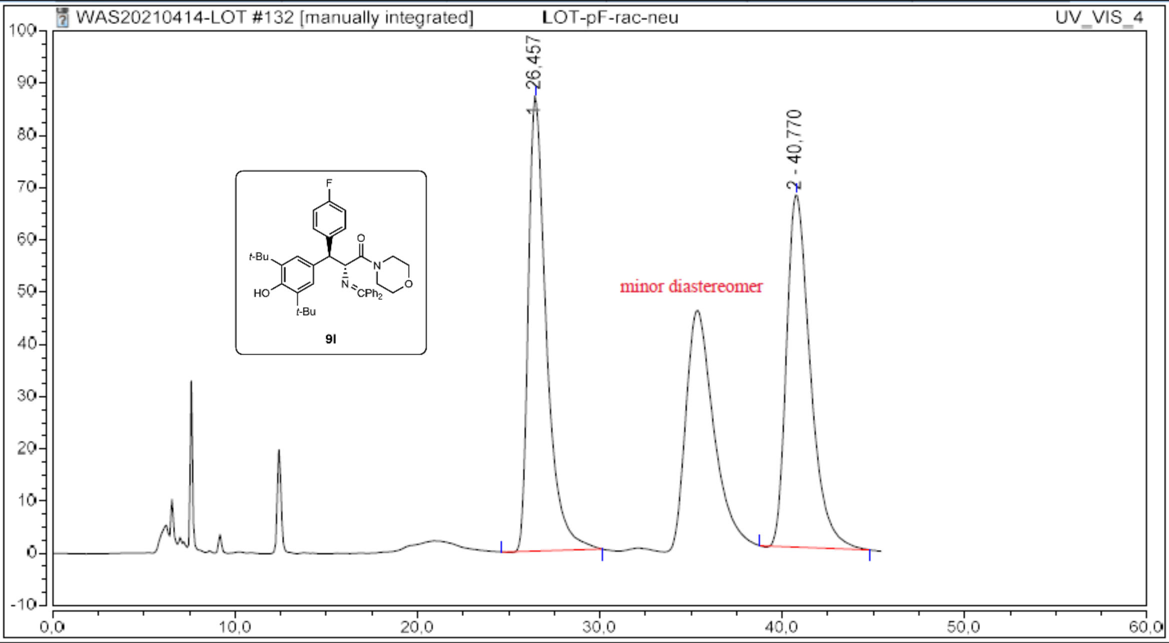


## Integration Results

| No. | Peak Name | Retention Time min | Area mAU*min | Height mAU | Relative Area % | Relative Height % | Amount n.a. |
|---|---|---|---|---|---|---|---|
| 1 | | 26,457 | 99,946 | 86,715 | 49,52 | 56,21 | n.a. |
| 2 | | 40,770 | 101,899 | 67,556 | 50,48 | 43,79 | n.a. |
| **Total:** | | | **201,845** | **154,271** | **100,00** | **100,00** | |

## Chromatogram

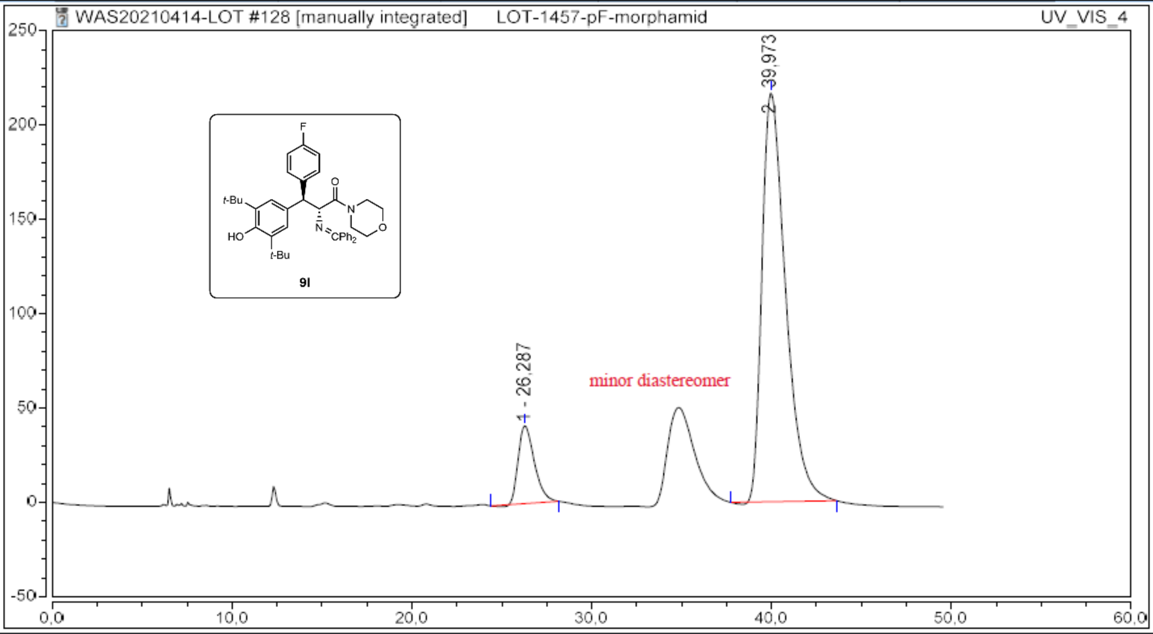


## Integration Results

| No. | Peak Name | Retention Time min | Area mAU*min | Height mAU | Relative Area % | Relative Height % | Amount n.a. |
|---|---|---|---|---|---|---|---|
| 1 | | 26,287 | 42,748 | 41,347 | 11,47 | 16,03 | n.a. |
| 2 | | 39,973 | 329,786 | 216,649 | 88,53 | 83,97 | n.a. |
| **Total:** | | | **372,534** | **257,996** | **100,00** | **100,00** | |

**4-nitrophenyl 3-(3,5-di-tert-butyl-4-hydroxyphenyl)-2-((diphenylmethylene)amino)-3-(4-chlorophenyl)propanoate 8m** and the corresponding morpholine amide **9m**

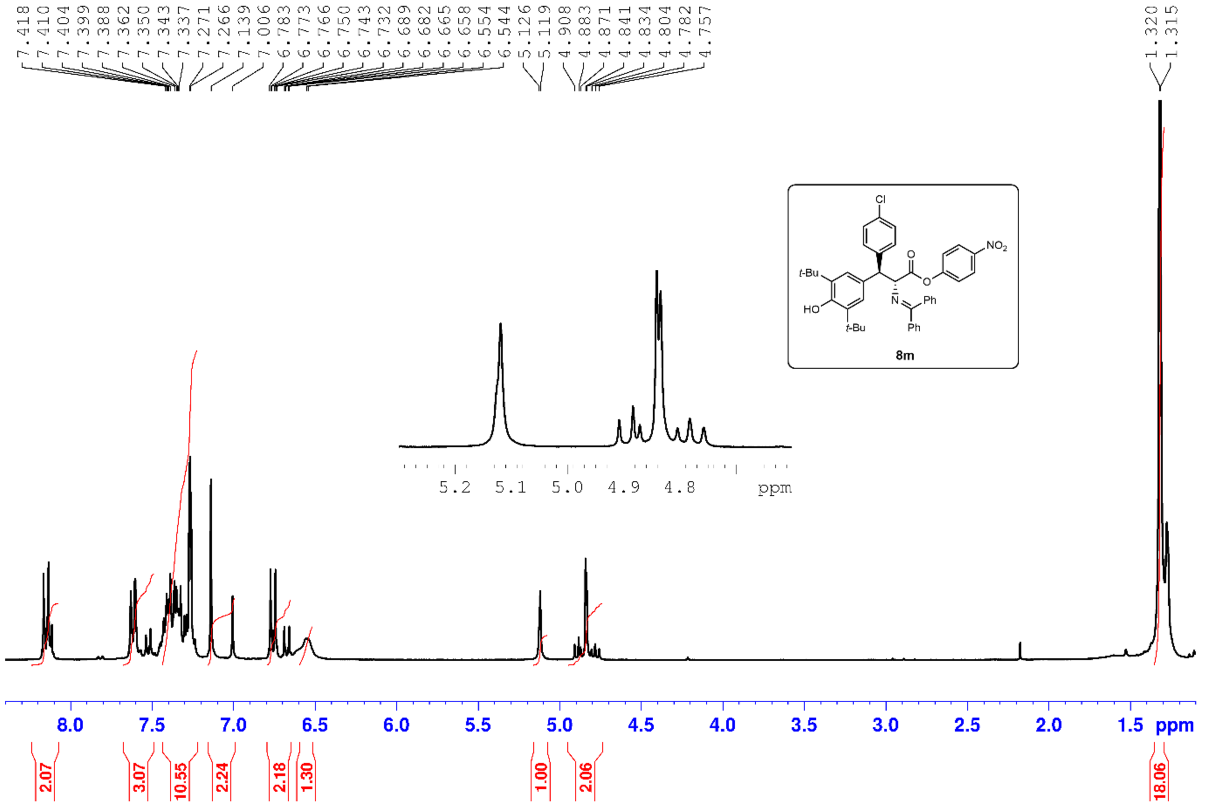


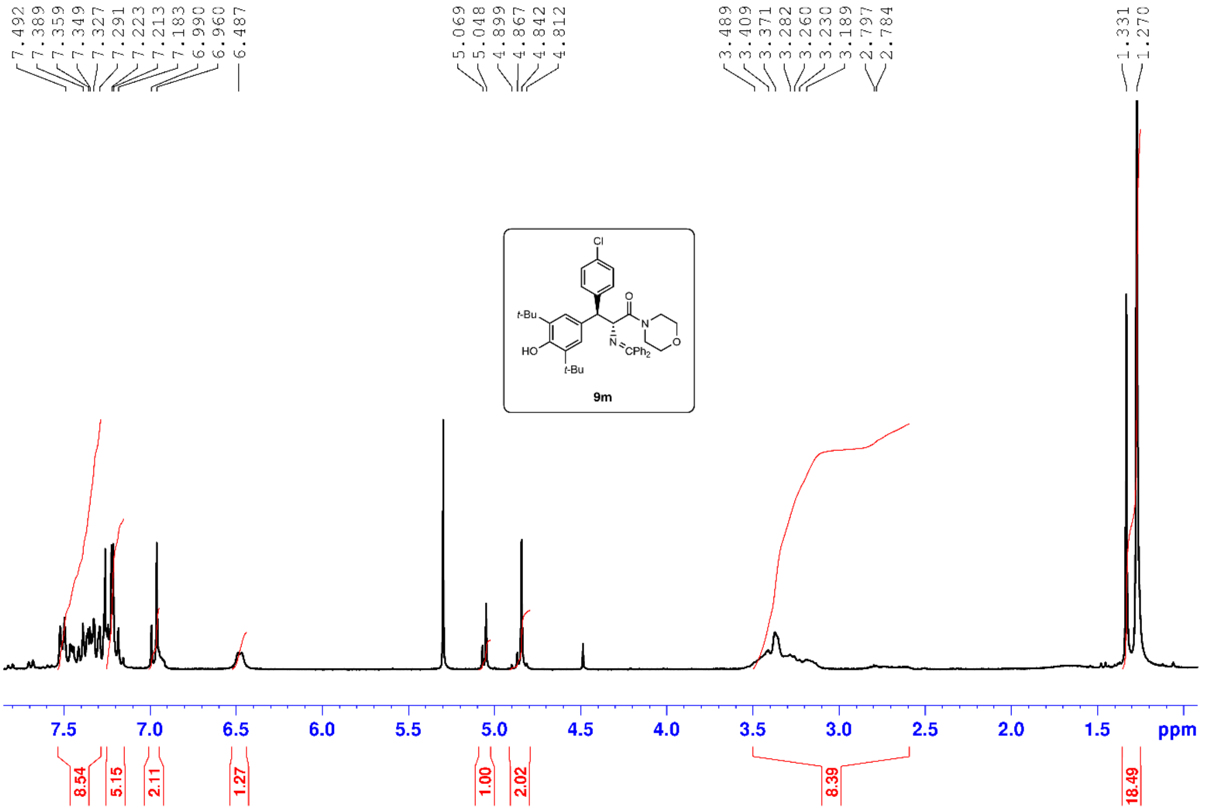

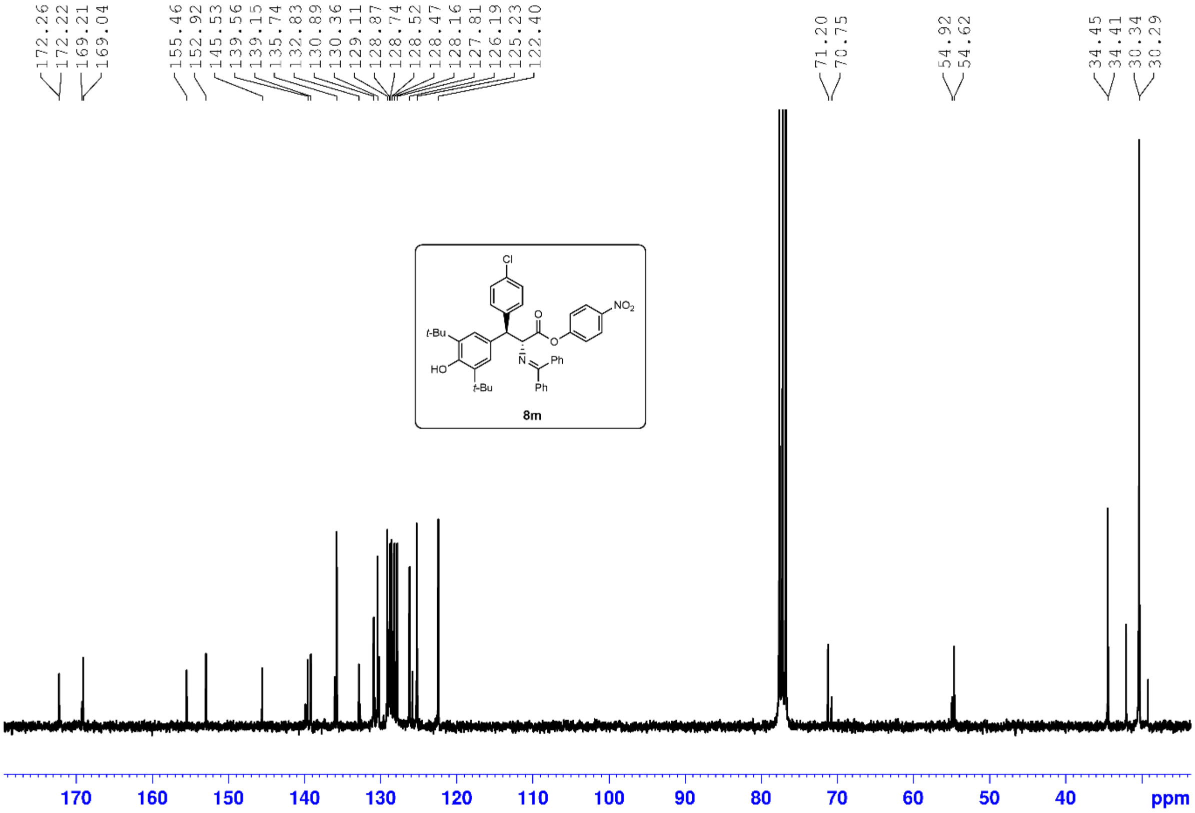

172.26
172.22
169.21
169.04
155.46
152.92
145.53
139.56
139.15
135.74
132.83
130.89
130.36
129.11
128.87
128.74
128.52
128.47
128.16
127.81
126.19
125.23
122.40
71.20
70.75
54.92
54.62
34.45
34.41
30.34
30.29
Cl
NO2
O
t-Bu
HO
t-Bu
O
N
Ph
Ph
8m
170 160 150 140 130 120 110 100 90 80 70 60 50 40 ppm


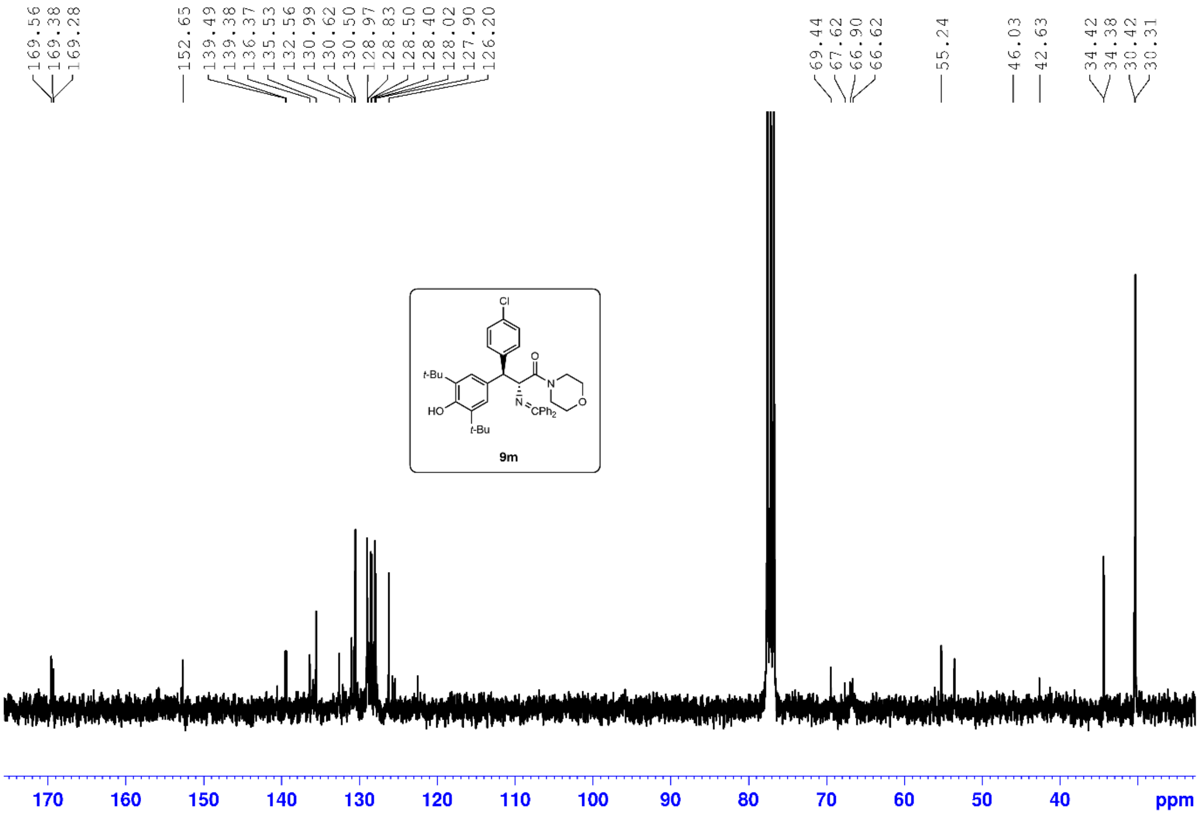

169.56
169.38
169.28
152.65
139.49
139.38
136.37
135.53
132.56
130.99
130.62
130.50
128.97
128.83
128.50
128.40
128.02
127.90
126.20
69.44
67.62
66.90
66.62
55.24
46.03
42.63
34.42
34.38
30.42
30.31
Cl
O
N
O
t-Bu
HO
t-Bu
N
CPh2
9m
170 160 150 140 130 120 110 100 90 80 70 60 50 40 ppm

**Chromatogram**

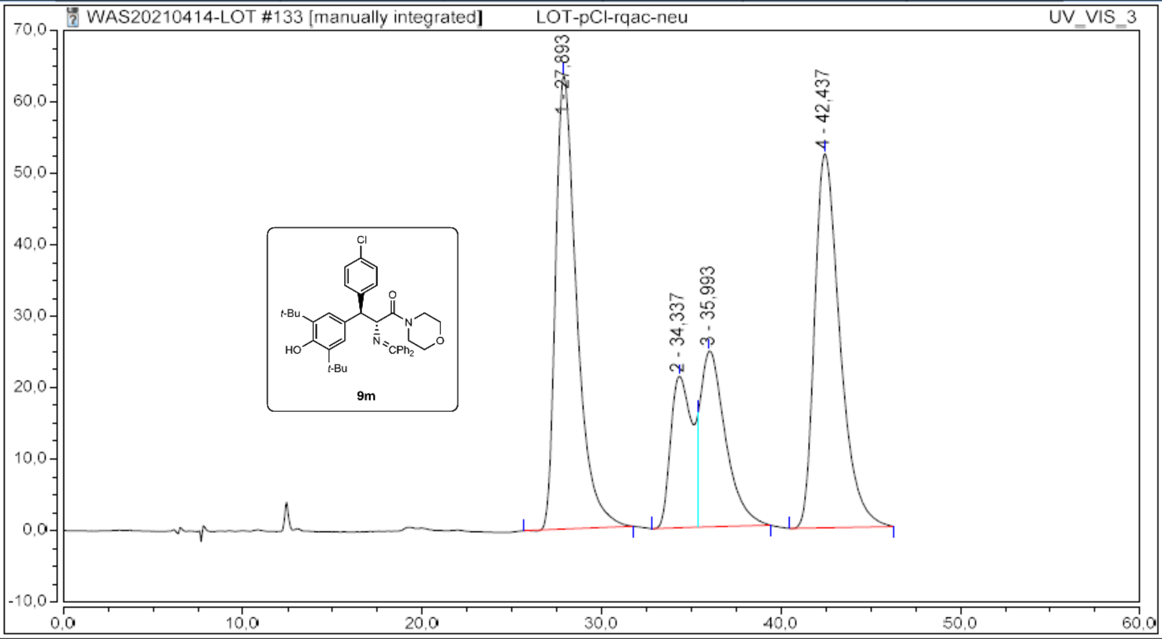


**Integration Results**

| No. | Peak Name | Retention Time min | Area mAU*min | Height mAU | Relative Area % | Relative Height % | Amount n.a. |
|---|---|---|---|---|---|---|---|
| 1 | | 27,893 | 86,764 | 63,561 | 35,91 | 39,25 | n.a. |
| 2 | | 34,337 | 30,074 | 21,223 | 12,45 | 13,11 | n.a. |
| 3 | | 35,993 | 39,058 | 24,664 | 16,17 | 15,23 | n.a. |
| 4 | | 42,437 | 85,703 | 52,475 | 35,47 | 32,41 | n.a. |
| **Total:** | | | **241,598** | **161,922** | **100,00** | **100,00** | |

**Chromatogram**

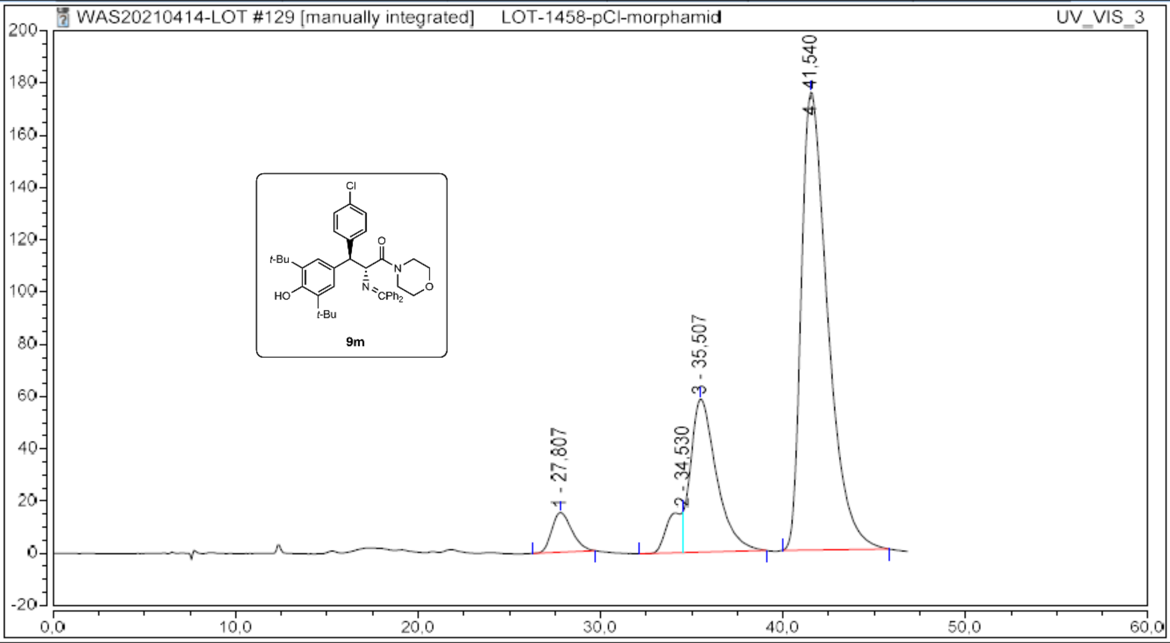


**Integration Results**

| No. | Peak Name | Retention Time min | Area mAU*min | Height mAU | Relative Area % | Relative Height % | Amount n.a. |
|---|---|---|---|---|---|---|---|
| 1 | | 27,807 | 19,489 | 15,249 | 4,57 | 5,75 | n.a. |
| 2 | | 34,530 | 15,626 | 15,782 | 3,67 | 5,95 | n.a. |
| 3 | | 35,507 | 96,573 | 58,680 | 22,66 | 22,14 | n.a. |
| 4 | | 41,540 | 294,587 | 175,373 | 69,11 | 66,16 | n.a. |
| **Total:** | | | **426,276** | **265,084** | **100,00** | **100,00** | |

**4-nitrophenyl 3-(3,5-di-tert-butyl-4-hydroxyphenyl)-2-((diphenylmethylene)amino)-3-(4-bromophenyl)propanoate 8n** and the corresponding morpholine amide **8n**

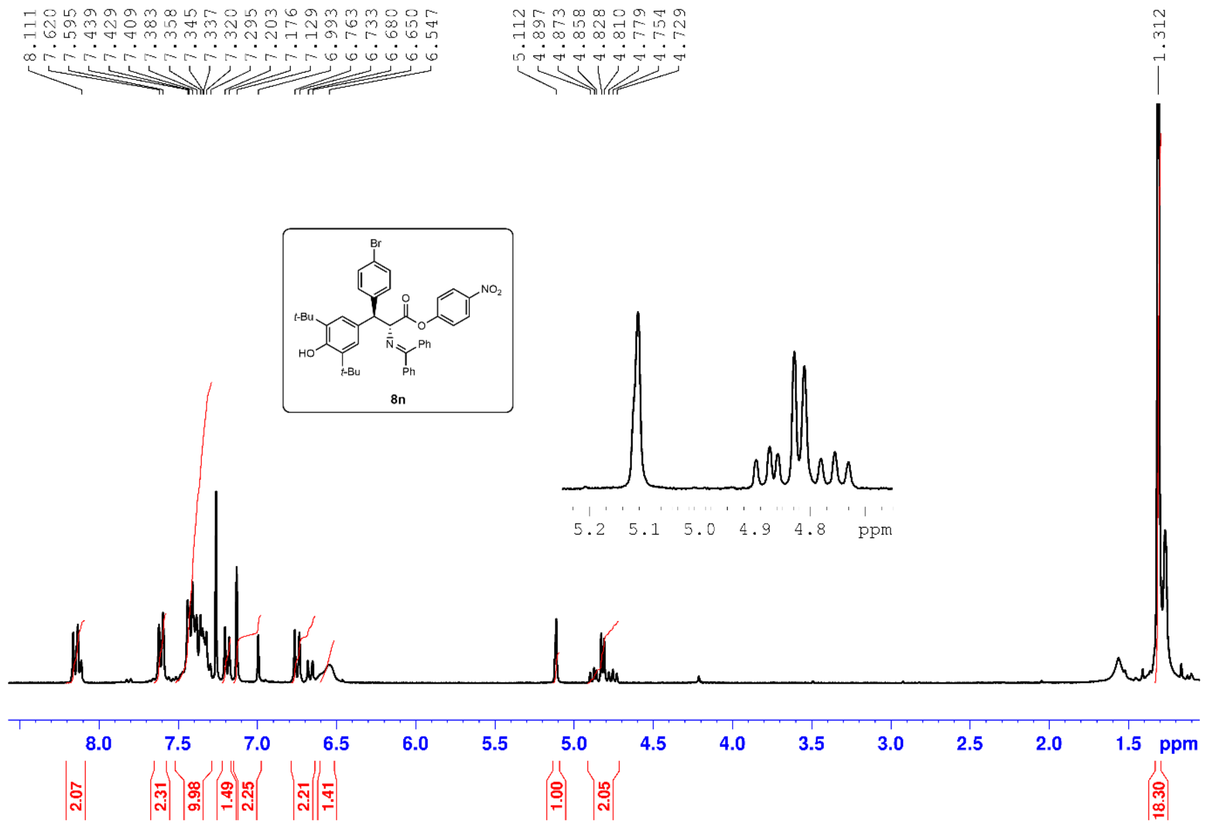


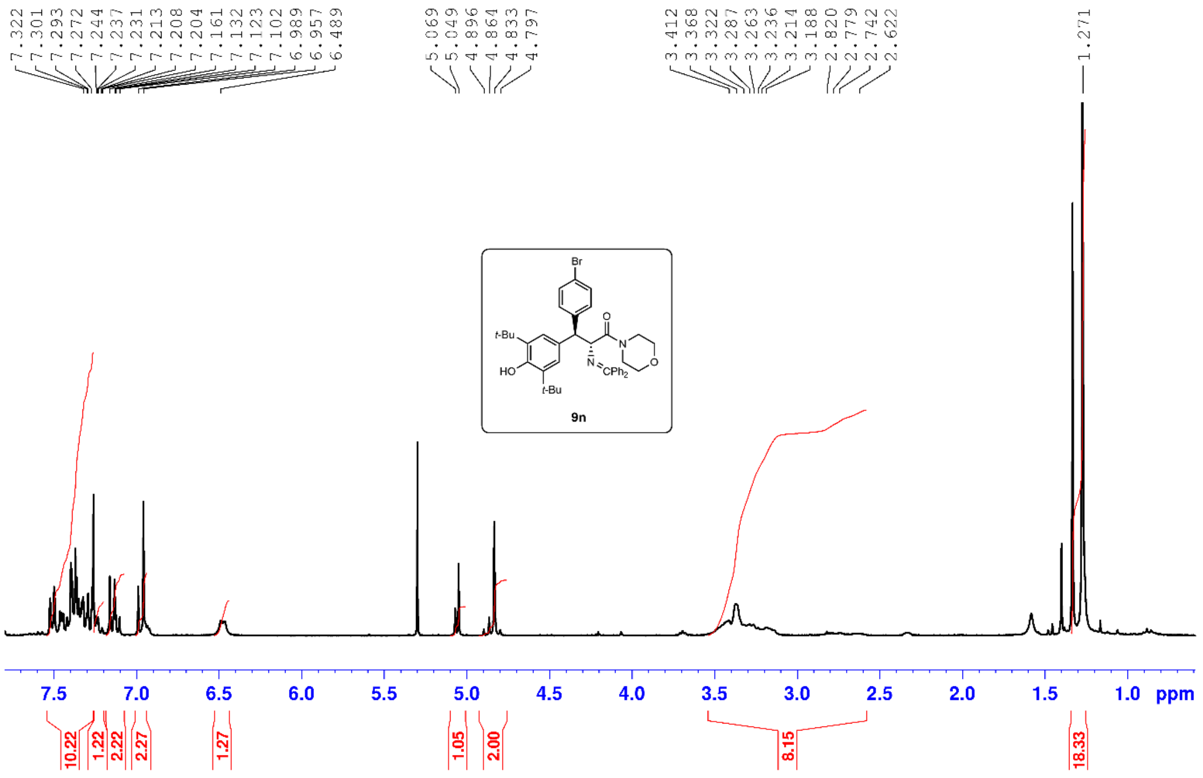

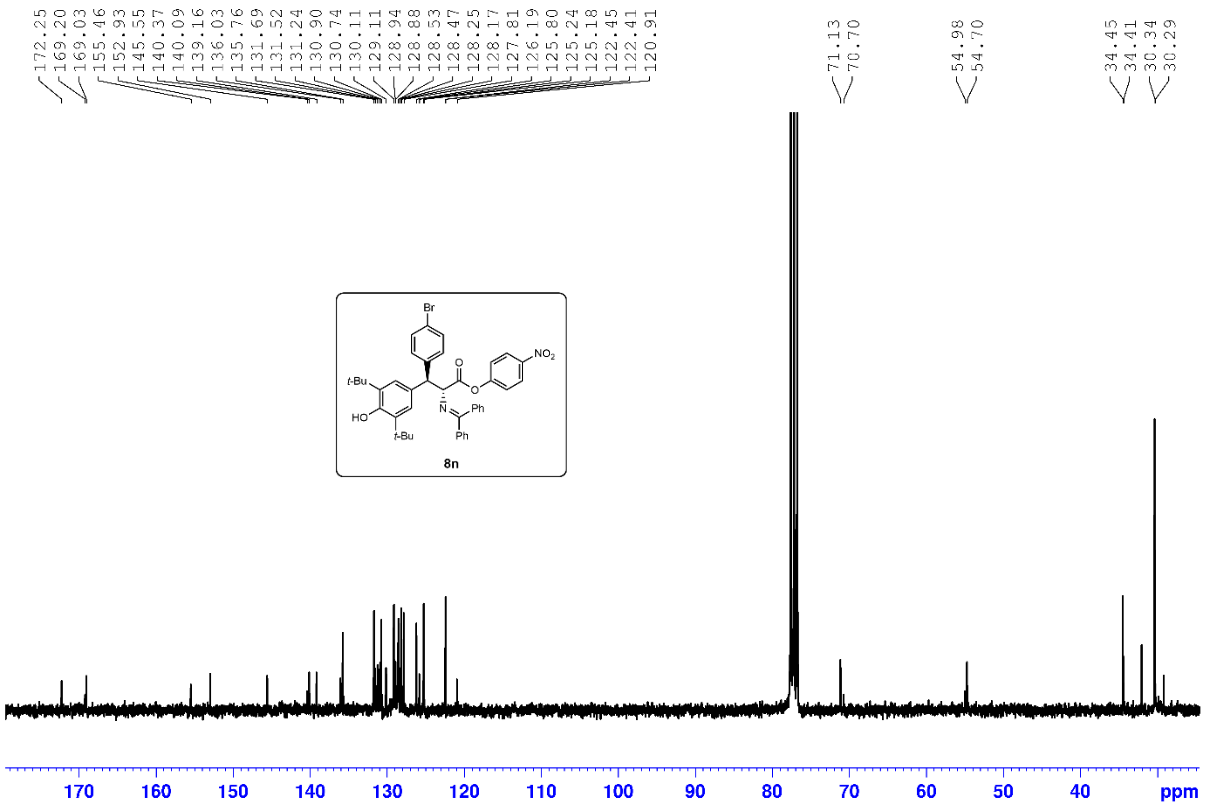

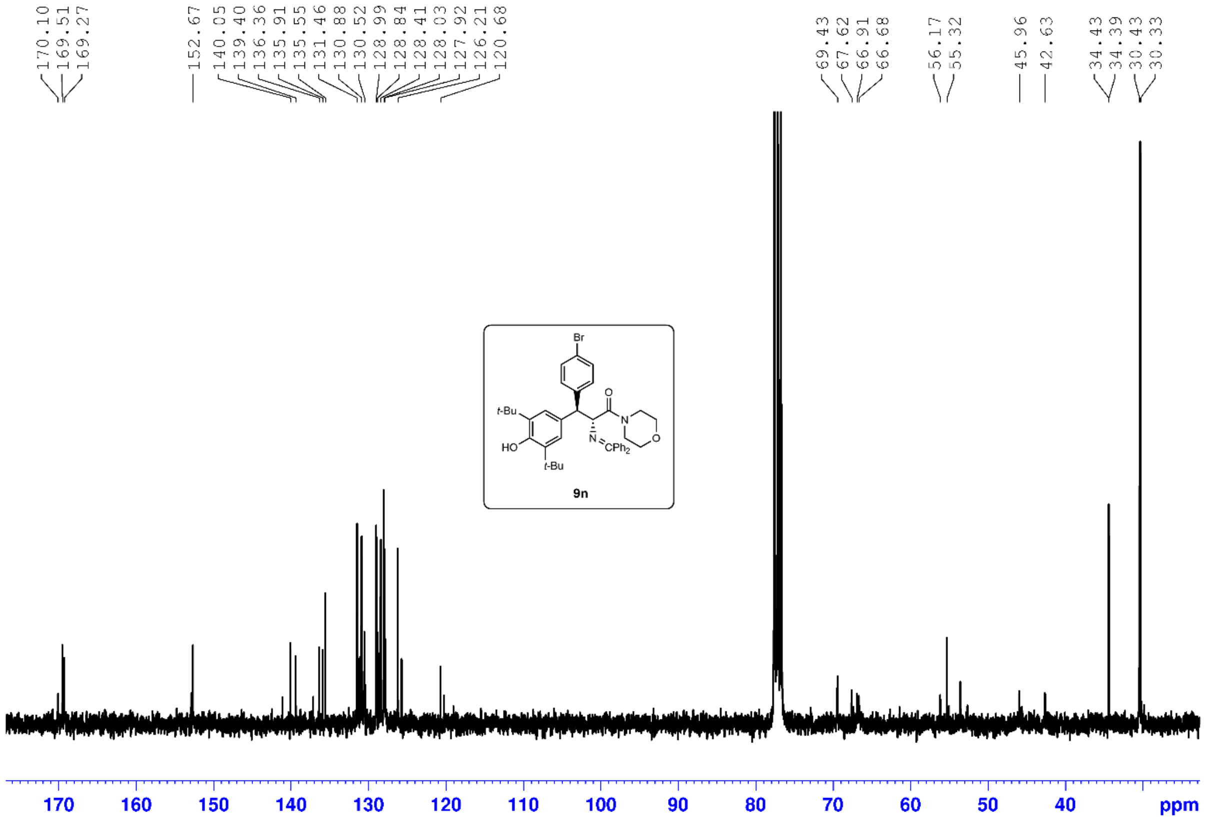

## Chromatogram

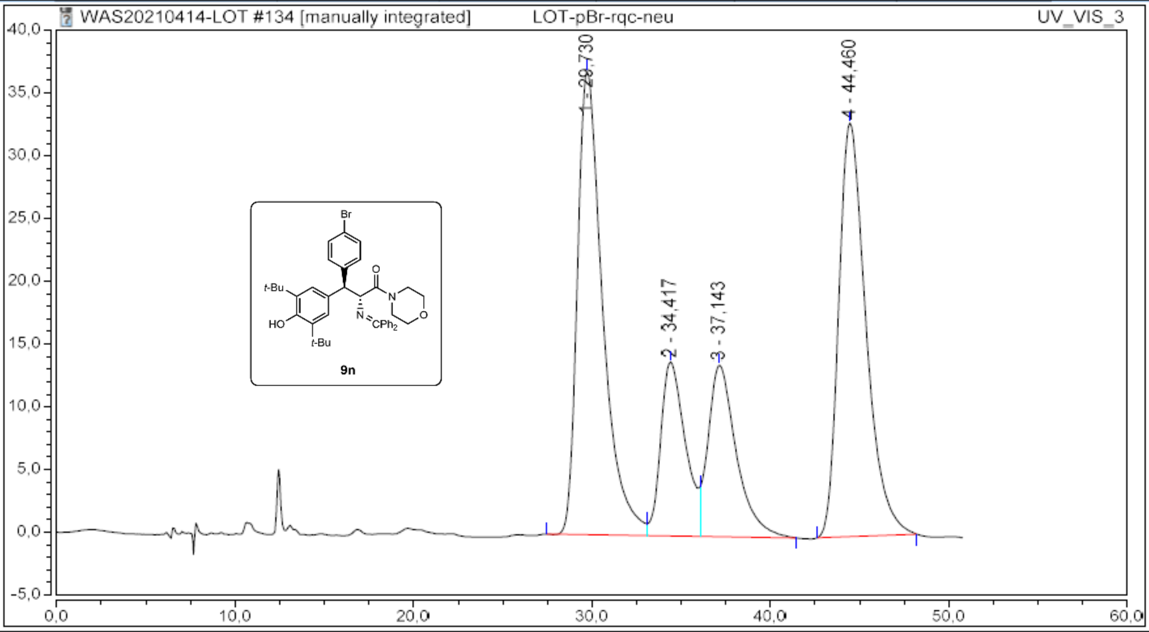


## Integration Results

| No. | Peak Name | Retention Time min | Area mAU*min | Height mAU | Relative Area % | Relative Height % | Amount n.a. |
|---|---|---|---|---|---|---|---|
| 1 | | 29,730 | 60,697 | 36,921 | 36,47 | 37,89 | n.a. |
| 2 | | 34,417 | 22,207 | 13,874 | 13,34 | 14,24 | n.a. |
| 3 | | 37,143 | 25,052 | 13,687 | 15,05 | 14,05 | n.a. |
| 4 | | 44,460 | 58,478 | 32,957 | 35,14 | 33,82 | n.a. |
| **Total:** | | | **166,433** | **97,440** | **100,00** | **100,00** | |

## Chromatogram

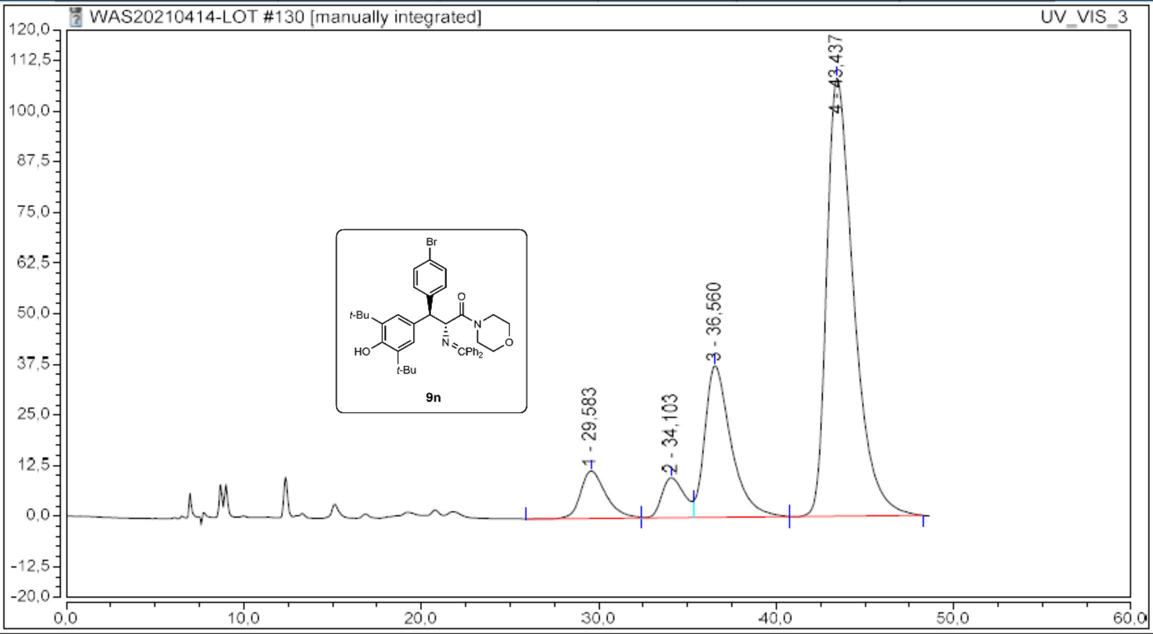


## Integration Results

| No. | Peak Name | Retention Time min | Area mAU*min | Height mAU | Relative Area % | Relative Height % | Amount n.a. |
|---|---|---|---|---|---|---|---|
| 1 | | 29,583 | 19,365 | 11,756 | 6,70 | 7,02 | n.a. |
| 2 | | 34,103 | 14,235 | 9,861 | 4,92 | 5,89 | n.a. |
| 3 | | 36,560 | 64,218 | 37,422 | 22,20 | 22,36 | n.a. |
| 4 | | 43,437 | 191,405 | 108,302 | 66,18 | 64,72 | n.a. |
| **Total:** | | | **289,223** | **167,341** | **100,00** | **100,00** | |

**4-nitrophenyl 3-(3,5-di-tert-butyl-4-hydroxyphenyl)-2-((diphenylmethylene)amino)-3-(naphthalen-1-yl)propanoate 8o**

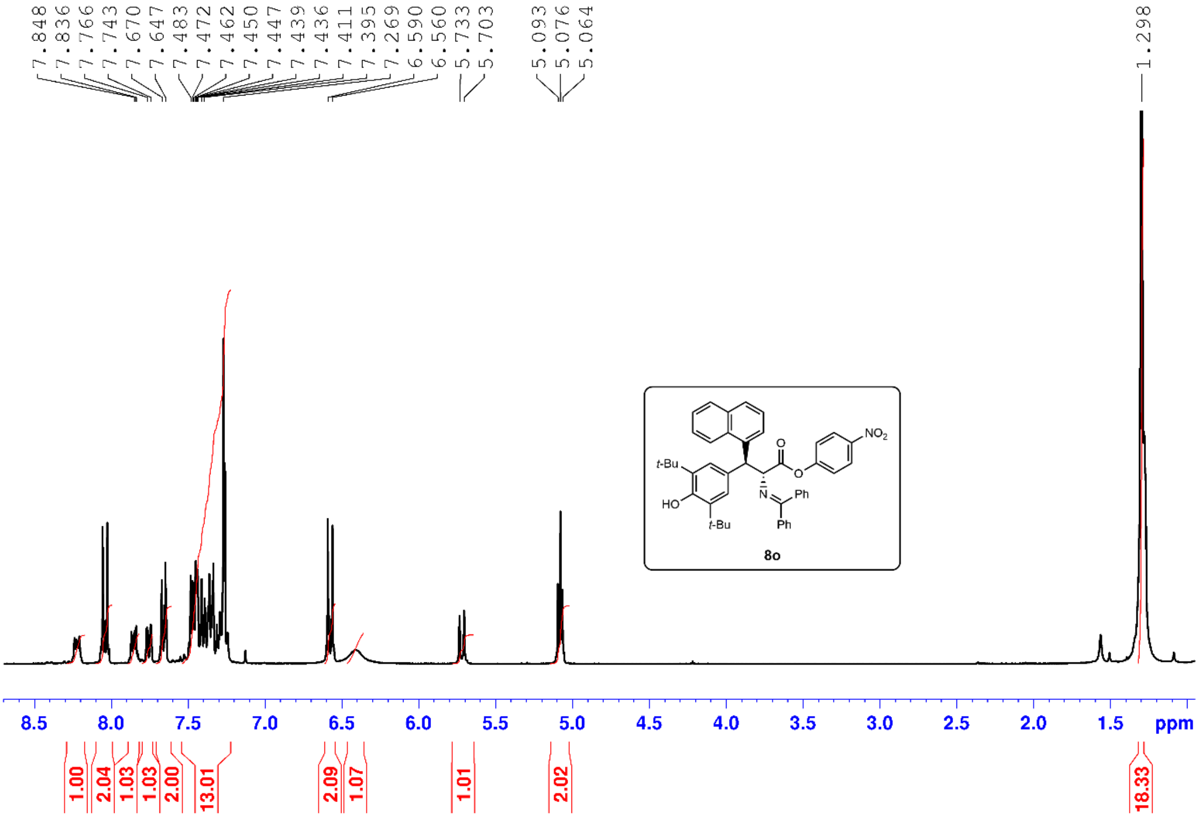


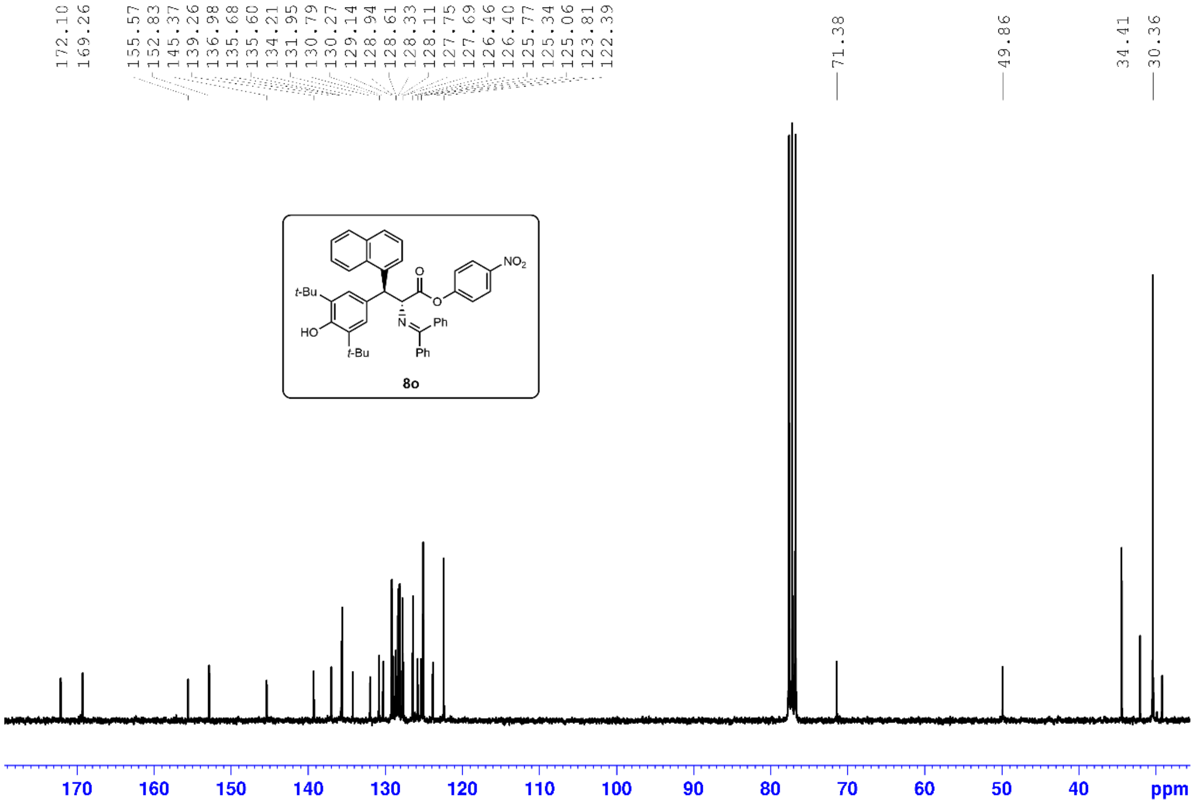

## Chromatogram

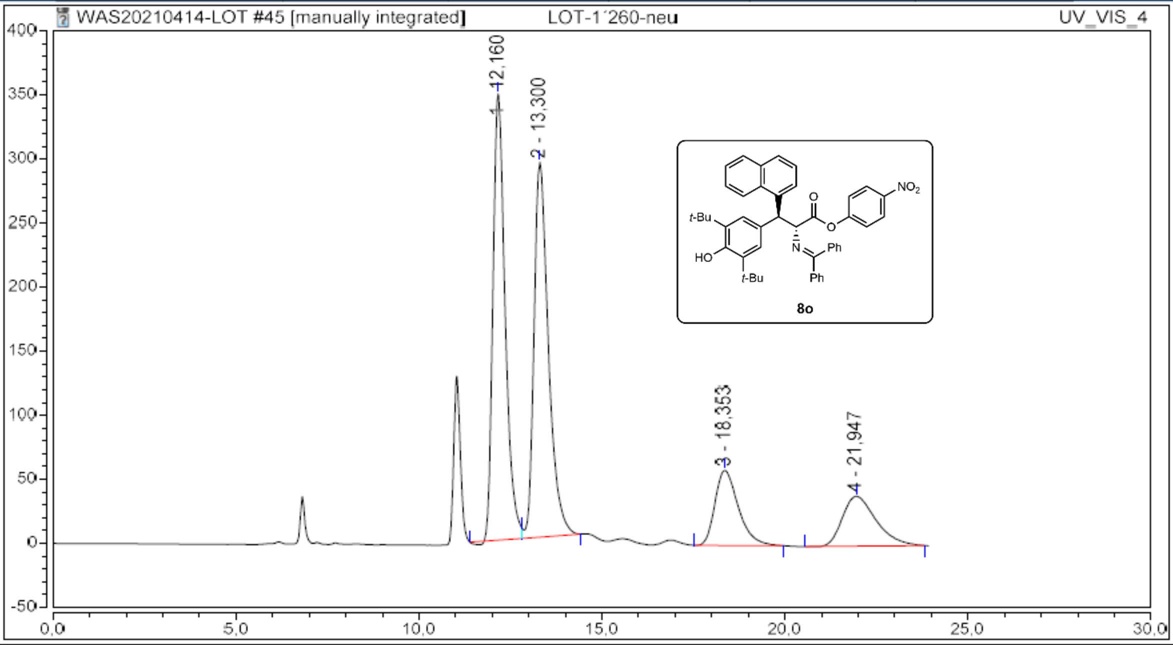


## Integration Results

| No. | Peak Name | Retention Time min | Area mAU*min | Height mAU | Relative Area % | Relative Height % | Amount n.a. |
|---|---|---|---|---|---|---|---|
| 1 | | 12,160 | 133,949 | 348,339 | 37,97 | 47,17 | n.a. |
| 2 | | 13,300 | 133,767 | 292,643 | 37,92 | 39,63 | n.a. |
| 3 | | 18,353 | 43,467 | 58,564 | 12,32 | 7,93 | n.a. |
| 4 | | 21,947 | 41,561 | 38,882 | 11,78 | 5,27 | n.a. |
| **Total:** | | | **352,745** | **738,428** | **100,00** | **100,00** | |

## Chromatogram

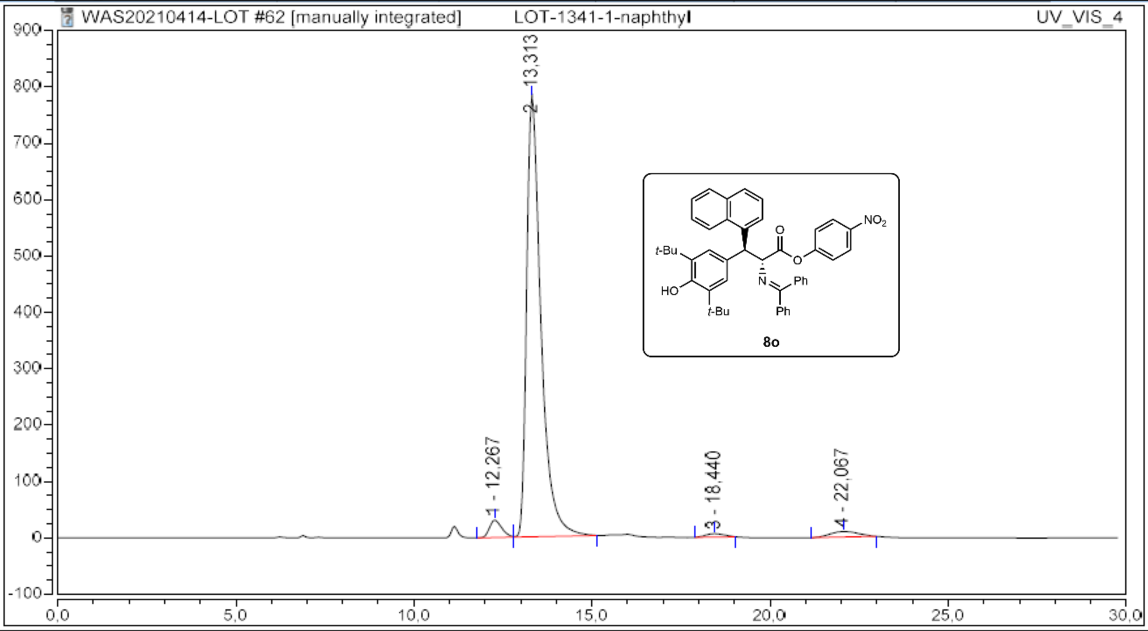


## Integration Results

| No. | Peak Name | Retention Time min | Area mAU*min | Height mAU | Relative Area % | Relative Height % | Amount n.a. |
|---|---|---|---|---|---|---|---|
| 1 | | 12,267 | 12,221 | 30,542 | 3,14 | 3,70 | n.a. |
| 2 | | 13,313 | 364,354 | 779,824 | 93,56 | 94,36 | n.a. |
| 3 | | 18,440 | 3,633 | 6,034 | 0,93 | 0,73 | n.a. |
| 4 | | 22,067 | 9,222 | 10,031 | 2,37 | 1,21 | n.a. |
| **Total:** | | | **389,430** | **826,431** | **100,00** | **100,00** | |

## Chromatogram

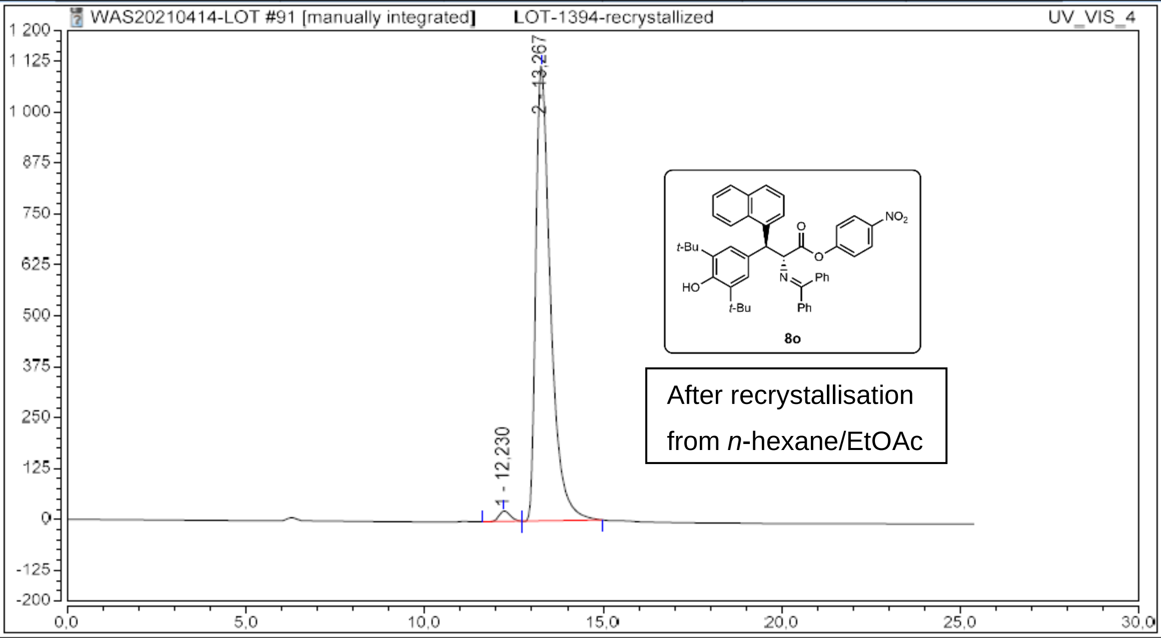


## Integration Results

| No. | Peak Name | Retention Time min | Area mAU*min | Height mAU | Relative Area % | Relative Height % | Amount n.a. |
|---|---|---|---|---|---|---|---|
| 1 | | 12,230 | 8,974 | 25,409 | 1,66 | 2,23 | n.a. |
| 2 | | 13,267 | 533,161 | 1116,305 | 98,34 | 97,77 | n.a. |
| **Total:** | | | **542,135** | **1141,715** | **100,00** | **100,00** | |

**4-nitrophenyl 3-(3,5-di-tert-butyl-4-hydroxyphenyl)-2-((diphenylmethylene)amino)-3-(naphthalen-2-yl)propanoate 8p**

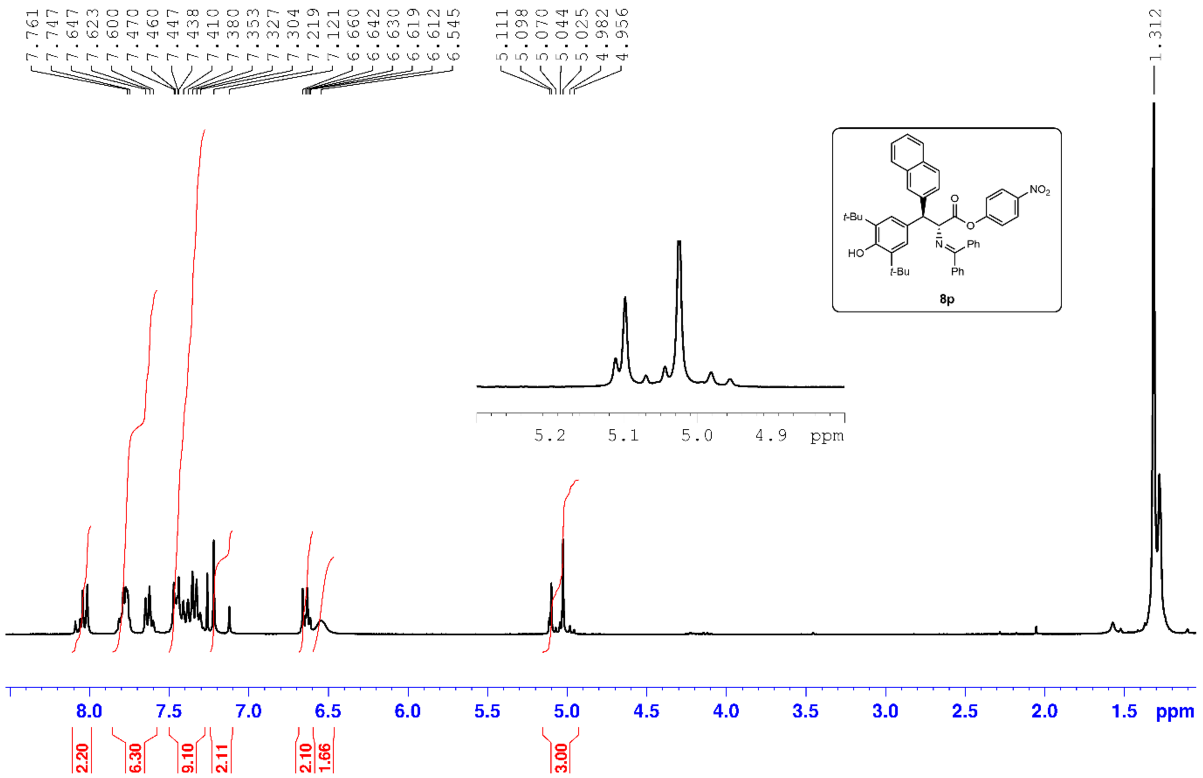


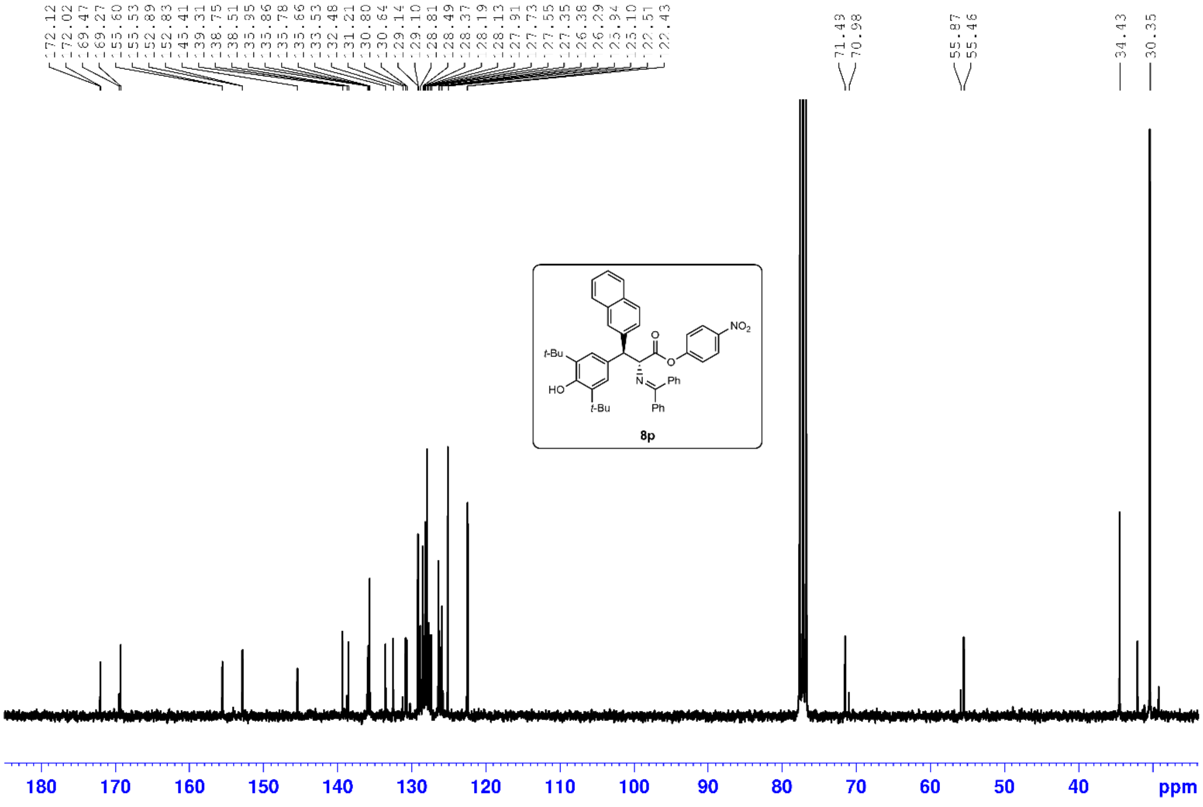

## Chromatogram

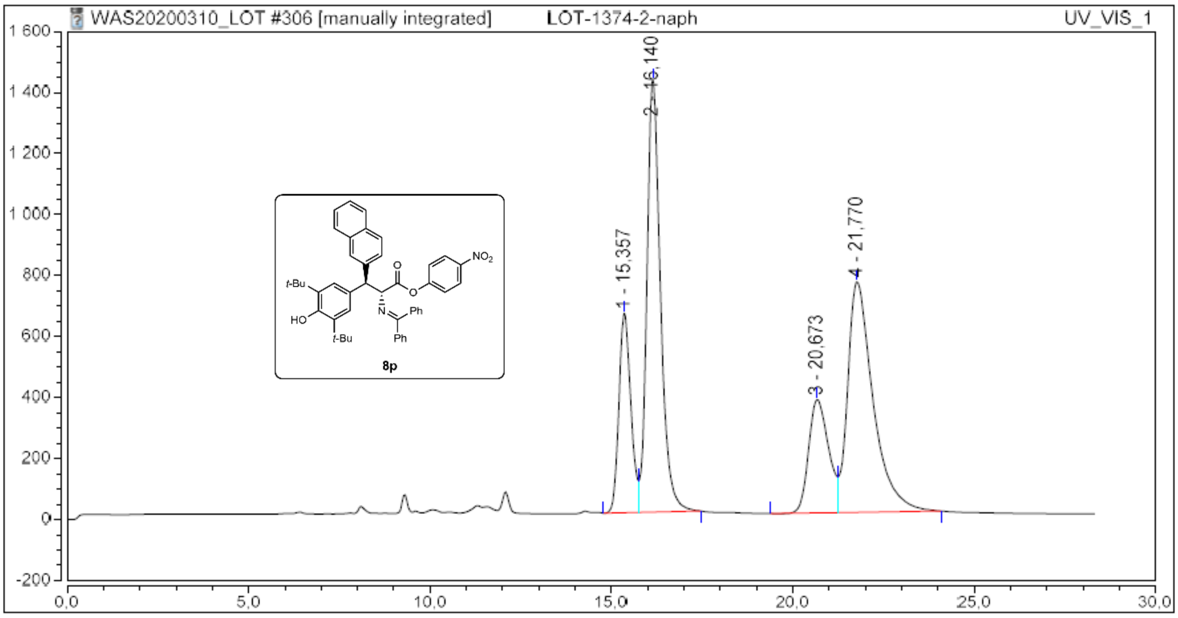


## Integration Results

| No. | Peak Name | Retention Time<br>min | Area<br>mAU*min | Height<br>mAU | Relative Area<br>% | Relative Height<br>% | Amount<br>n.a. |
|---|---|---|---|---|---|---|---|
| 1 | | 15,357 | 250,132 | 654,151 | 14,60 | 20,44 | n.a. |
| 2 | | 16,140 | 611,750 | 1418,449 | 35,71 | 44,31 | n.a. |
| 3 | | 20,673 | 238,019 | 371,620 | 13,90 | 11,61 | n.a. |
| 4 | | 21,770 | 613,023 | 756,685 | 35,79 | 23,64 | n.a. |
| | | | **1712,924** | **3200,904** | **100,00** | **100,00** | |

## Chromatogram

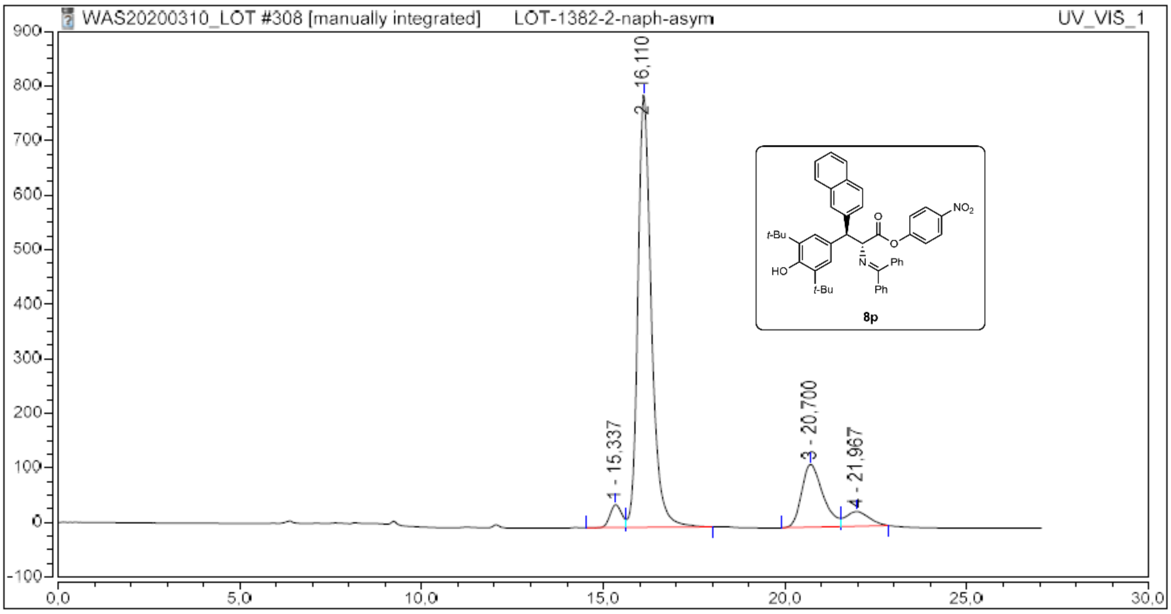


## Integration Results

| No. | Peak Name | Retention Time<br>min | Area<br>mAU*min | Height<br>mAU | Relative Area<br>% | Relative Height<br>% | Amount<br>n.a. |
|---|---|---|---|---|---|---|---|
| 1 | | 15,337 | 15,398 | 42,263 | 3,38 | 4,33 | n.a. |
| 2 | | 16,110 | 341,277 | 791,308 | 75,01 | 81,10 | n.a. |
| 3 | | 20,700 | 78,267 | 115,166 | 17,20 | 11,80 | n.a. |
| 4 | | 21,967 | 20,053 | 26,966 | 4,41 | 2,76 | n.a. |
| | | | **454,995** | **975,703** | **100,00** | **100,00** | |

**4-nitrophenyl 3-(3,5-di-tert-butyl-4-hydroxyphenyl)-2-((diphenylmethylene)amino)-3-(thiophen-2-yl)propanoate 8q**

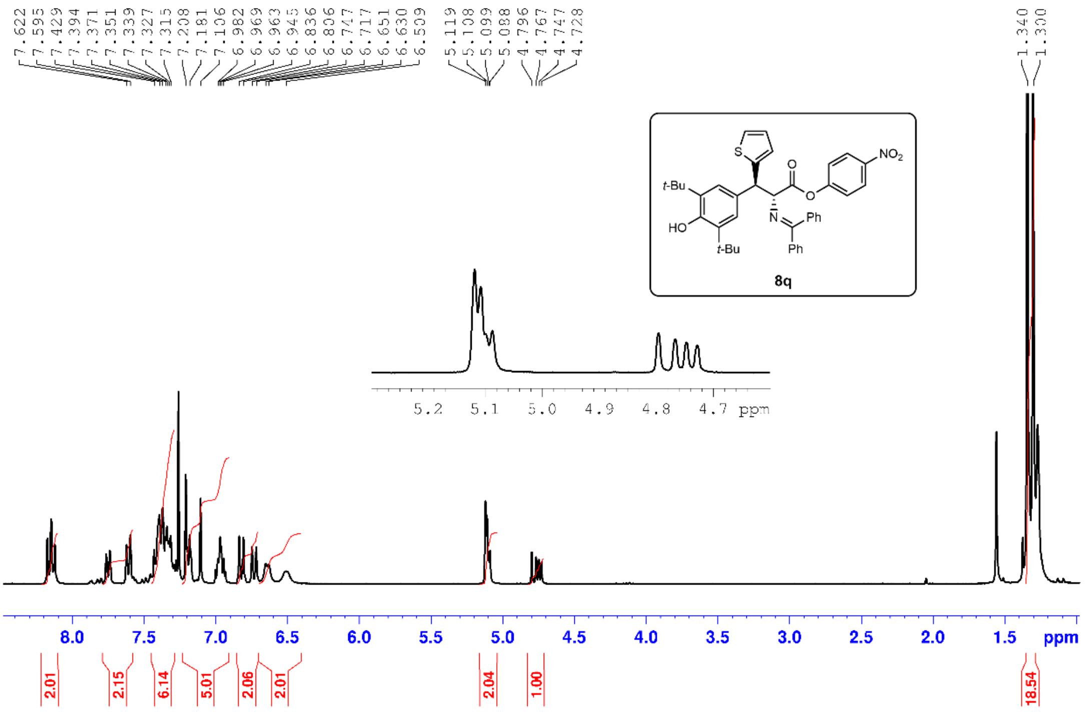

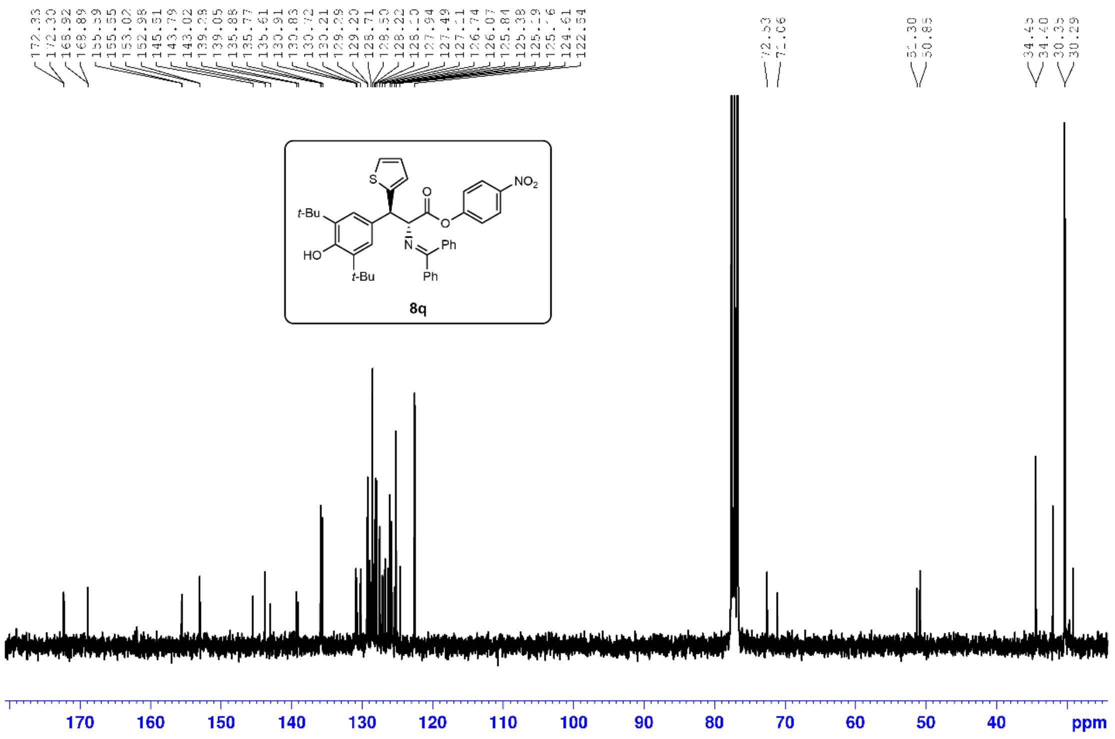

**Chromatogram**

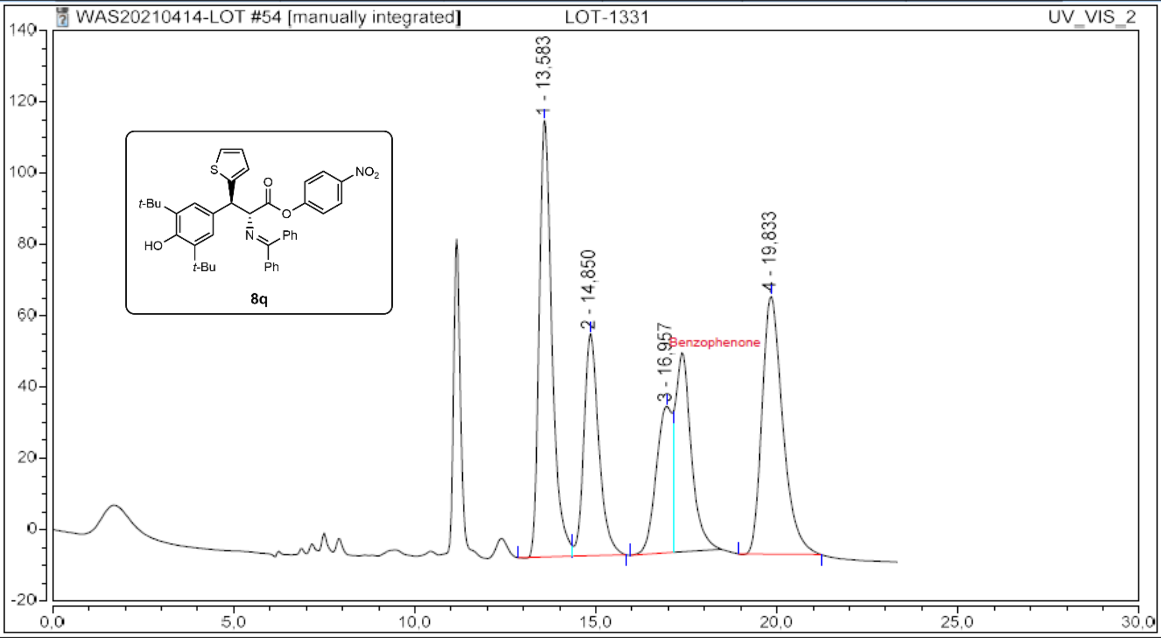


**Integration Results**

| No. | Peak Name | Retention Time<br>min | Area<br>mAU*min | Height<br>mAU | Relative Area<br>% | Relative Height<br>% | Amount<br>n.a. |
|---|---|---|---|---|---|---|---|
| 1 | | 13,583 | 50,032 | 122,528 | 33,58 | 41,07 | n.a. |
| 2 | | 14,850 | 29,738 | 62,329 | 19,96 | 20,89 | n.a. |
| 3 | | 16,957 | 21,217 | 41,132 | 14,24 | 13,79 | n.a. |
| 4 | | 19,833 | 48,023 | 72,385 | 32,23 | 24,26 | n.a. |
| **Total:** | | | **149,009** | **298,375** | **100,00** | **100,00** | |

**Chromatogram**

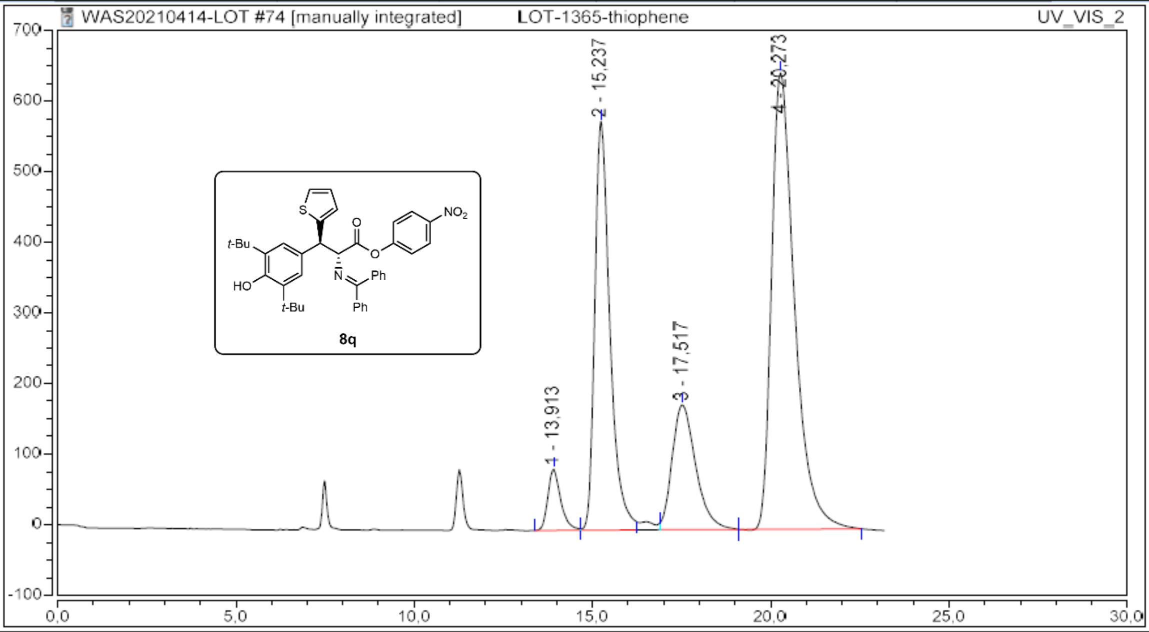


**Integration Results**

| No. | Peak Name | Retention Time<br>min | Area<br>mAU*min | Height<br>mAU | Relative Area<br>% | Relative Height<br>% | Amount<br>n.a. |
|---|---|---|---|---|---|---|---|
| 1 | | 13,913 | 36,132 | 86,883 | 3,85 | 5,83 | n.a. |
| 2 | | 15,237 | 285,641 | 578,879 | 30,40 | 38,87 | n.a. |
| 3 | | 17,517 | 135,920 | 176,844 | 14,47 | 11,87 | n.a. |
| 4 | | 20,273 | 481,870 | 646,803 | 51,29 | 43,43 | n.a. |
| **Total:** | | | **939,563** | **1489,409** | **100,00** | **100,00** | |

**4-nitrophenyl 3-(3,5-di-tert-butyl-4-hydroxyphenyl)-2-((diphenylmethylene)amino)-3-(pyridin-3-yl)propanoate 8r**

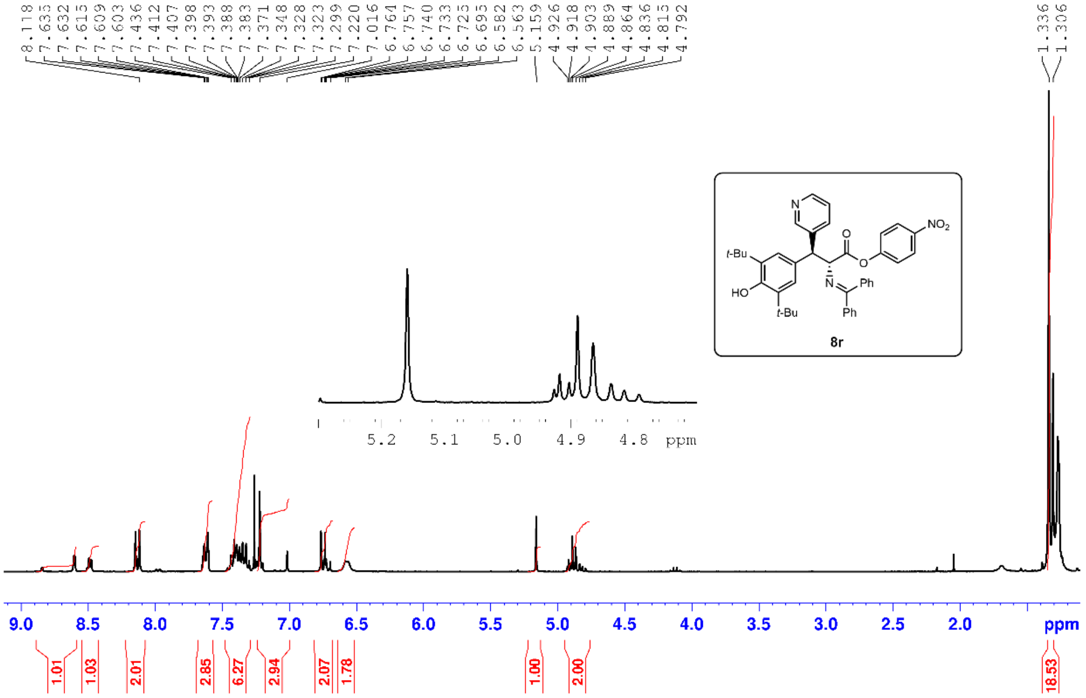

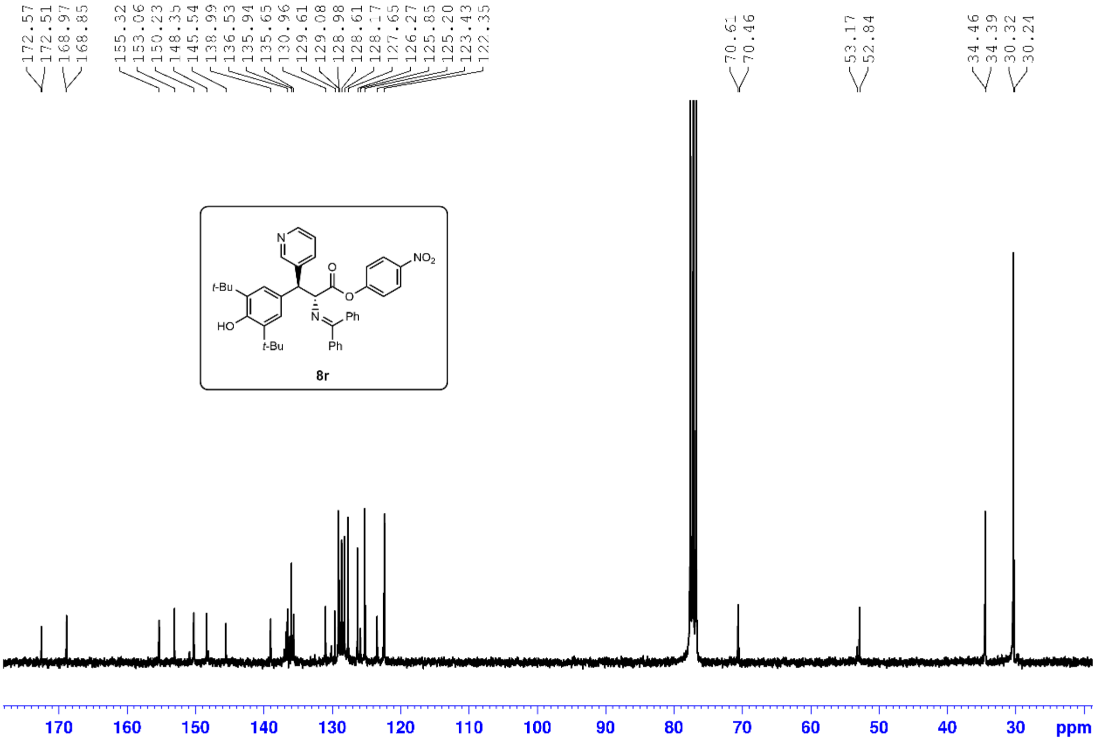

**Preparative HPLC** (Separation of the diastereomers of **8r**):

## 39 LOT-1476-py

| | | | |
|---|---|---|---|
| *Sample Name:* | LOT-1476-py | *Injection Volume:* | 290,0 |
| *Vial Number:* | BE4 | *Channel:* | UV_VIS_1 |
| *Sample Type:* | Unknown | *Wavelength:* | n.a. |
| *Control Program:* | 25%B_flow5_300µL_230nm_56min | *Bandwidth:* | n.a. |
| *Quantif. Method:* | Basic Quantitative | *Flow ml/min:* | 5,000 |
| *Recording Time:* | 21.4.2022 16:35 | *Sample Weight:* | 1,0000 |
| *Run Time (min):* | 16,62 | *Sample Amount:* | 1,0000 |

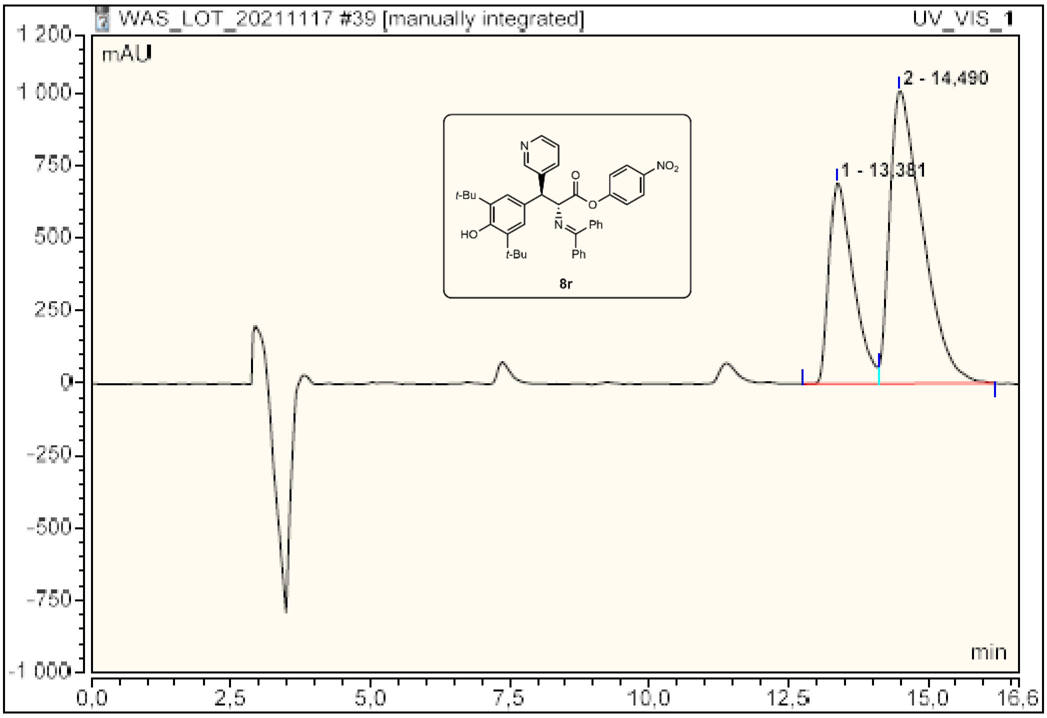


| No. | Ret.Time min | Peak Name | Height mAU | Area mAU*min | Rel.Area % | Amount n.a. | Type |
|---|---|---|---|---|---|---|---|
| 1 | 13,38 | | 693,399 | 346,757 | 33,43 | n.a. | BM * |
| 2 | 14,49 | | 1010,413 | 690,397 | 66,57 | n.a. | MB* |
| Total: | | | 1703,812 | 1037,153 | 100,00 | 0,000 | |

**Major diastereomer**

## Chromatogram

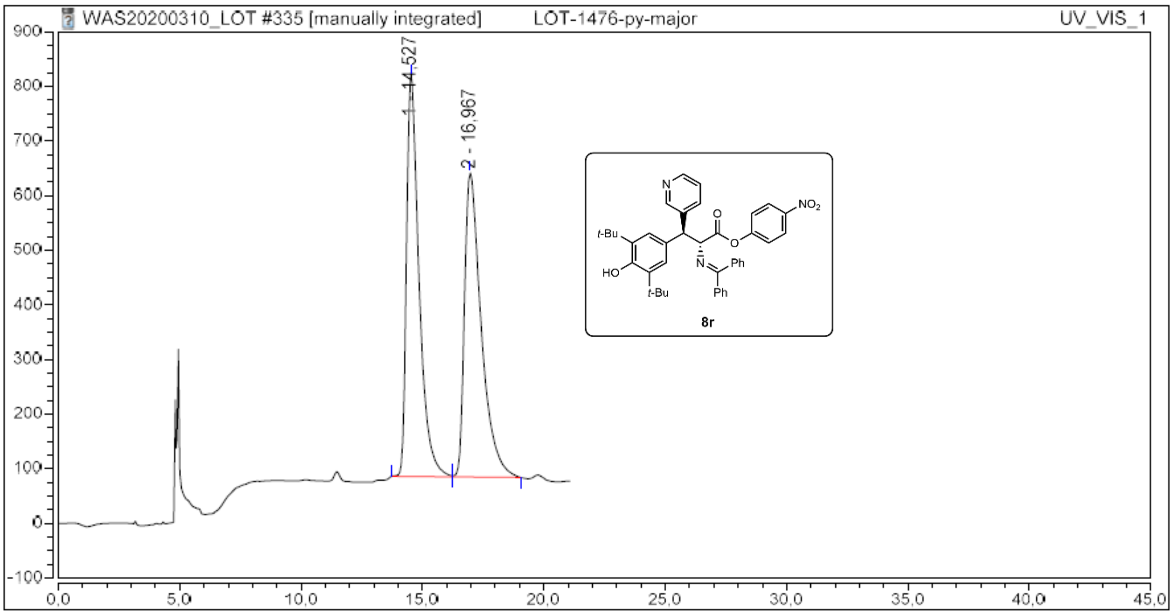


## Integration Results

| No. | Peak Name | Retention Time min | Area mAU*min | Height mAU | Relative Area % | Relative Height % | Amount n.a. |
|---|---|---|---|---|---|---|---|
| 1 | | 14,527 | 439,425 | 733,422 | 49,74 | 56,85 | n.a. |
| 2 | | 16,967 | 444,053 | 556,609 | 50,26 | 43,15 | n.a. |
| | | | **883,478** | **1290,031** | **100,00** | **100,00** | |

## Chromatogram

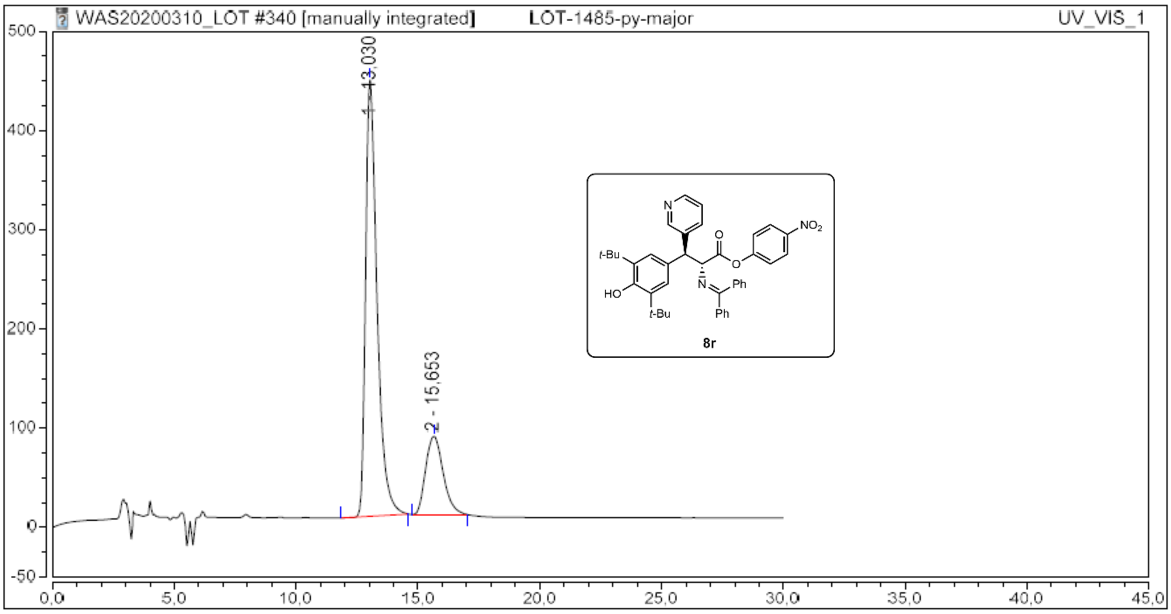


## Integration Results

| No. | Peak Name | Retention Time min | Area mAU*min | Height mAU | Relative Area % | Relative Height % | Amount n.a. |
|---|---|---|---|---|---|---|---|
| 1 | | 13,030 | 238,913 | 439,908 | 78,36 | 84,76 | n.a. |
| 2 | | 15,653 | 65,988 | 79,066 | 21,64 | 15,24 | n.a. |
| | | | **304,901** | **518,973** | **100,00** | **100,00** | |

## 4-nitrophenyl 3-(3,5-di-tert-butyl-4-hydroxyphenyl)-2-((diphenylmethylene)amino)butanoate 8s

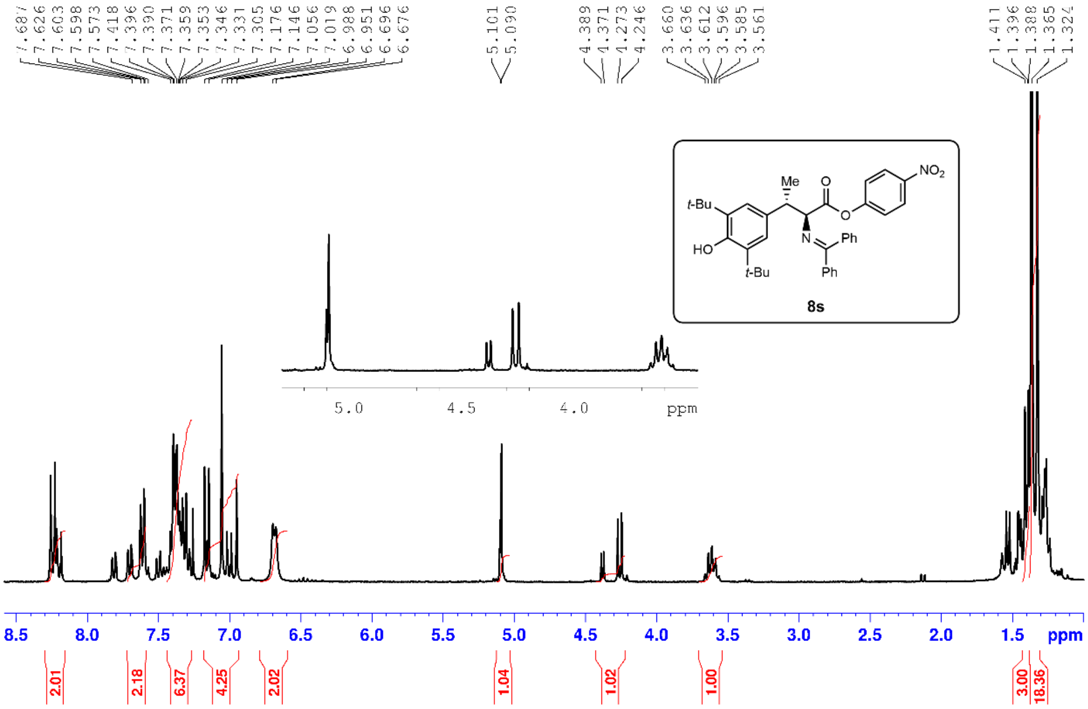


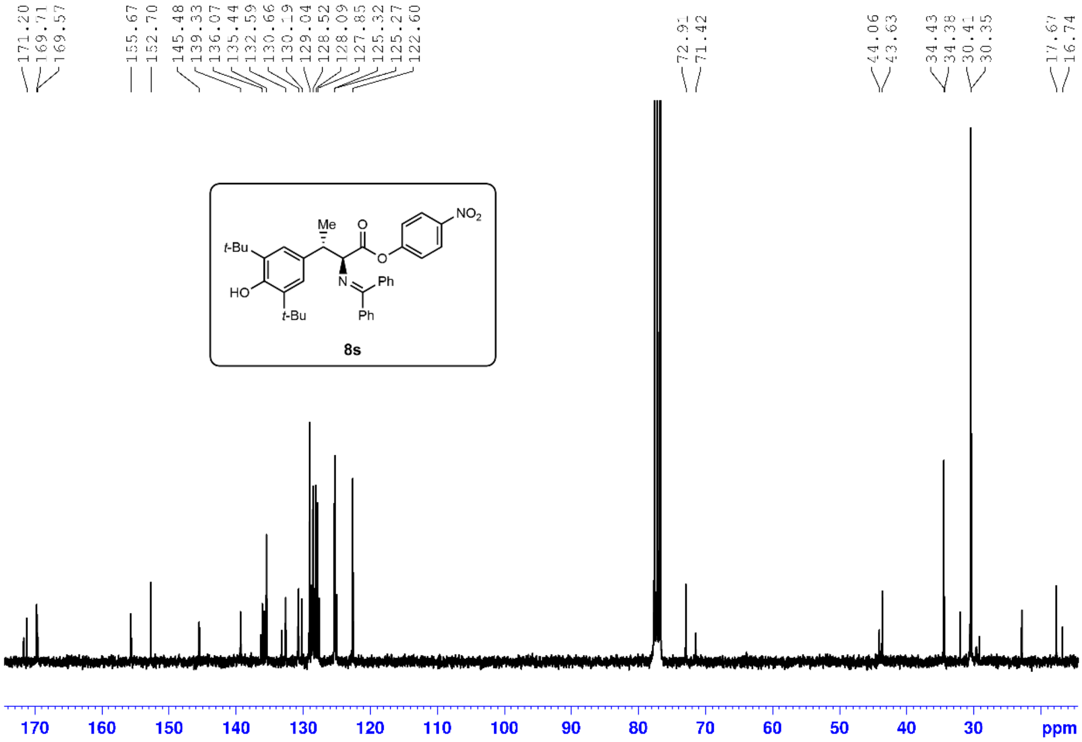

## Chromatogram

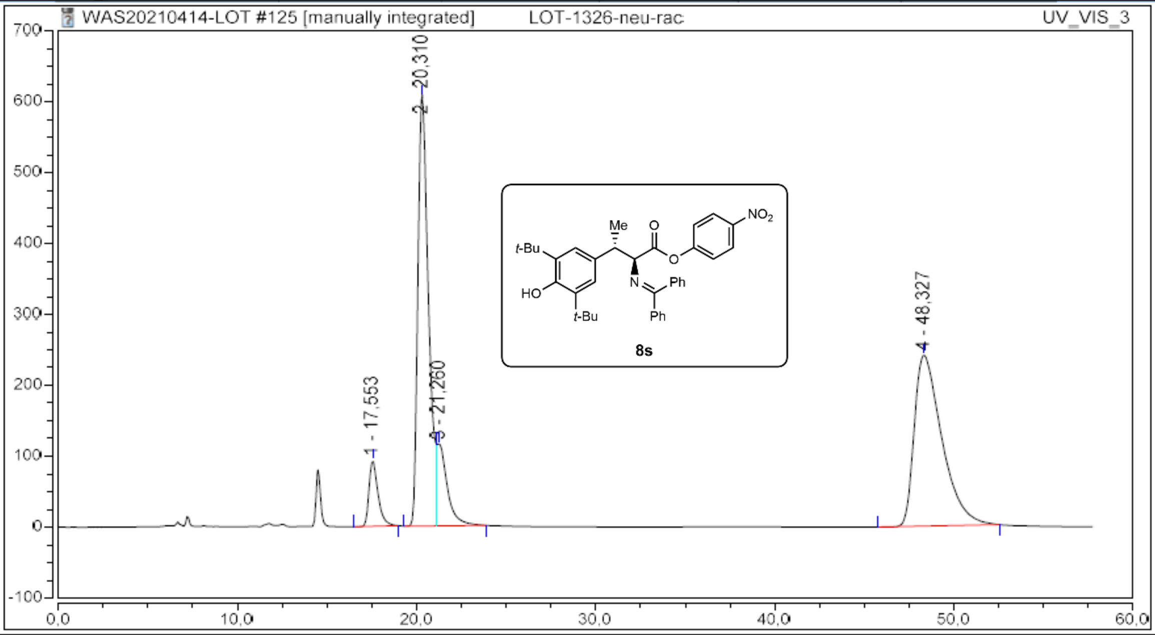


## Integration Results

| No. | Peak Name | Retention Time min | Area mAU*min | Height mAU | Relative Area % | Relative Height % | Amount n.a. |
|---|---|---|---|---|---|---|---|
| 1 | | 17,553 | 53,942 | 92,877 | 5,51 | 8,80 | n.a. |
| 2 | | 20,310 | 413,253 | 605,457 | 42,18 | 57,38 | n.a. |
| 3 | | 21,260 | 82,017 | 115,604 | 8,37 | 10,96 | n.a. |
| 4 | | 48,327 | 430,555 | 241,244 | 43,94 | 22,86 | n.a. |
| **Total:** | | | **979,767** | **1055,182** | **100,00** | **100,00** | |

## Chromatogram

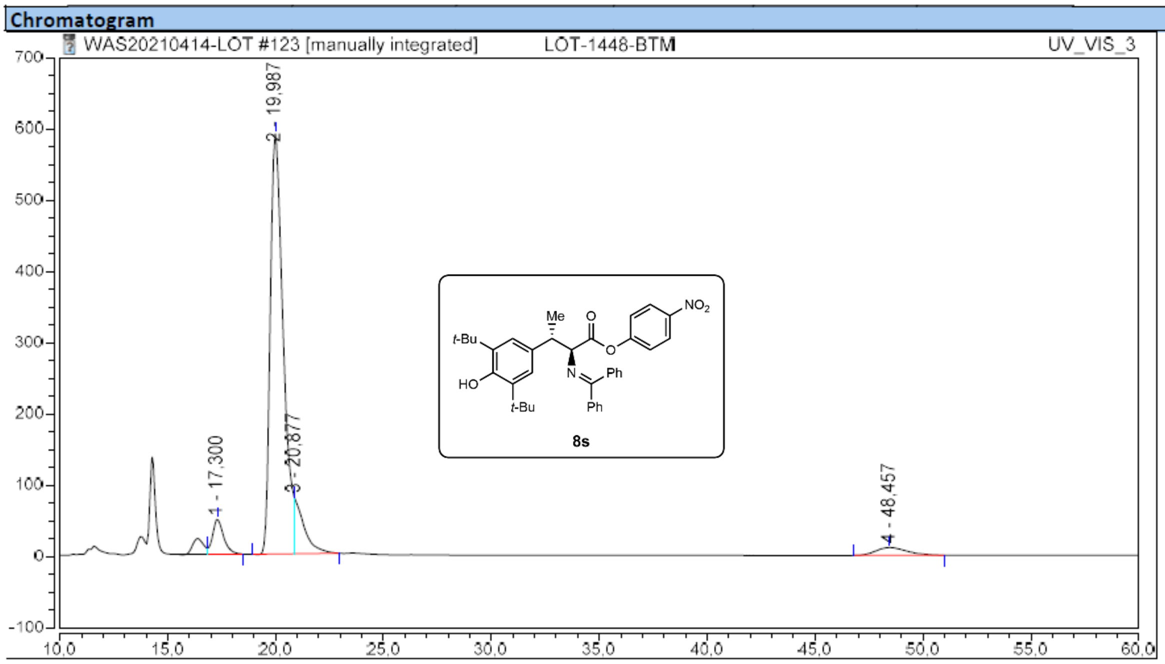


## Integration Results

| No. | Peak Name | Retention Time min | Area mAU*min | Height mAU | Relative Area % | Relative Height % | Amount n.a. |
|---|---|---|---|---|---|---|---|
| 1 | | 17,300 | 29,326 | 49,211 | 5,89 | 6,75 | n.a. |
| 2 | | 19,987 | 409,579 | 589,835 | 82,22 | 80,89 | n.a. |
| 3 | | 20,877 | 40,833 | 79,107 | 8,20 | 10,85 | n.a. |
| 4 | | 48,457 | 18,393 | 11,000 | 3,69 | 1,51 | n.a. |
| **Total:** | | | **498,131** | **729,153** | **100,00** | **100,00** | |

**4-nitrophenyl 3-(3,5-di-tert-butyl-4-hydroxyphenyl)-2-((diphenylmethylene)amino)-4,4,4-trifluorobutanoate 8t**

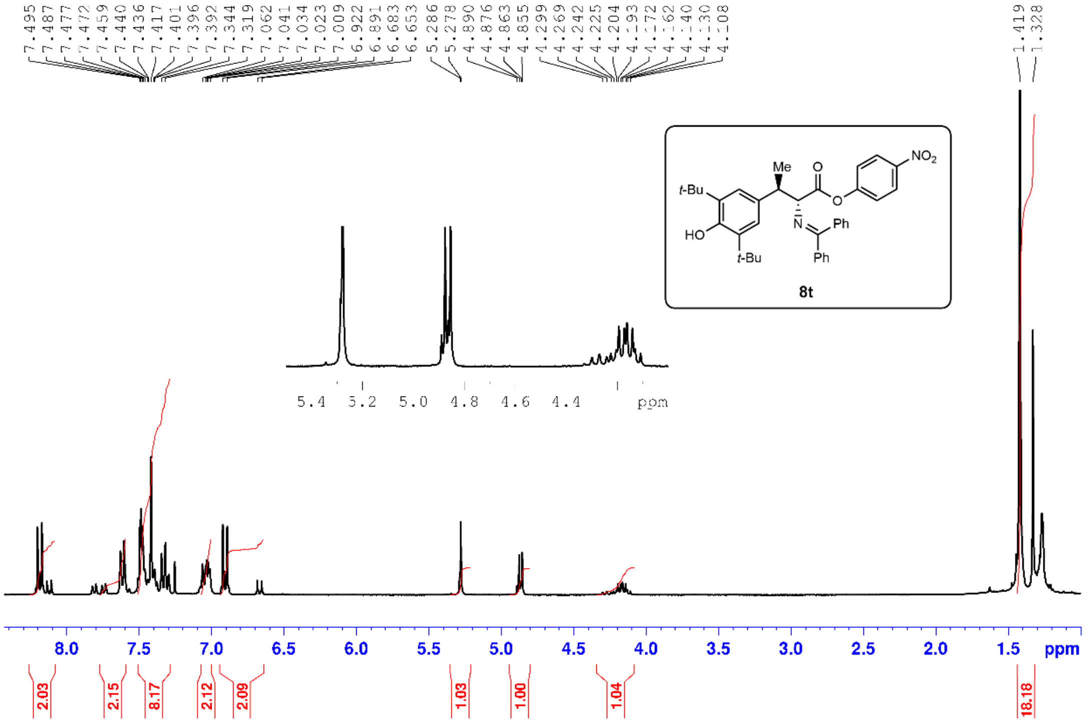


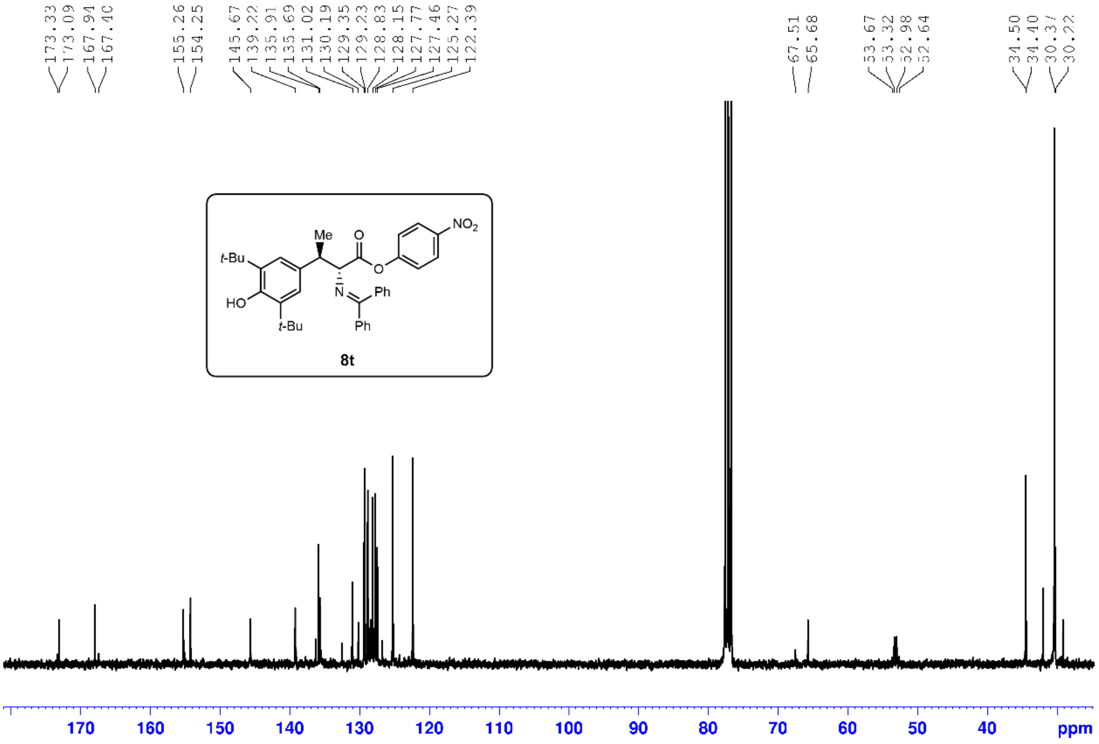

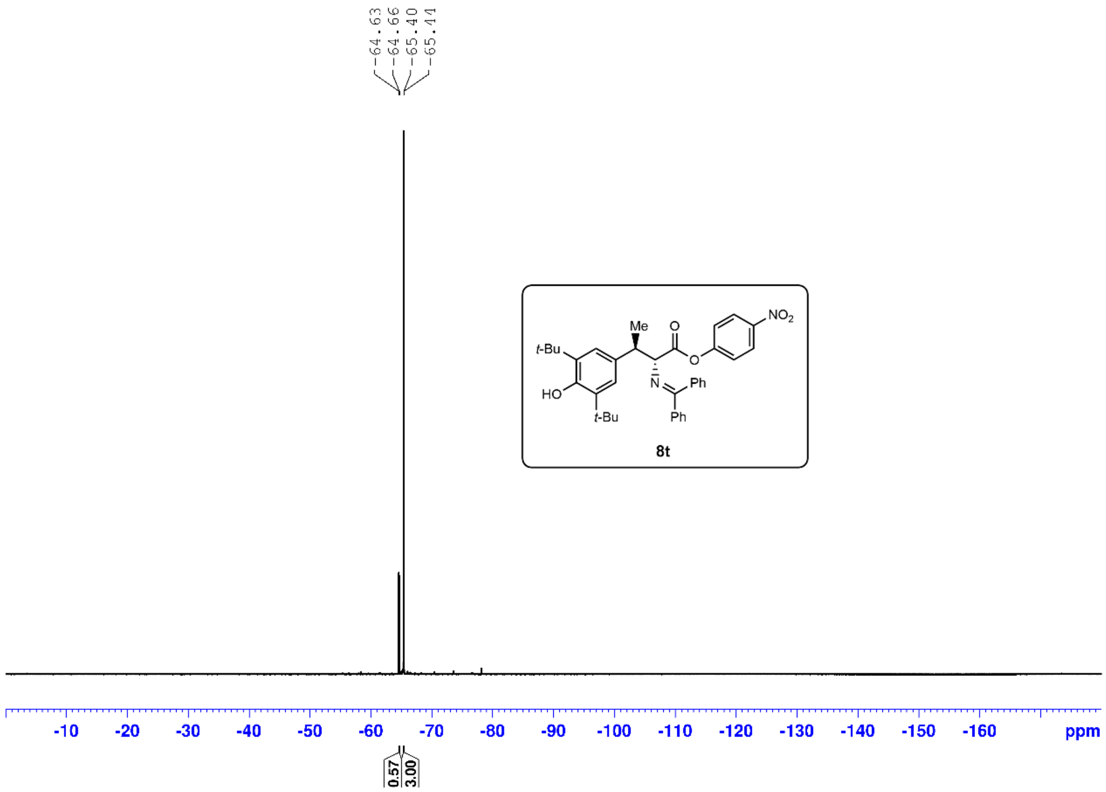
-64.63
-64.66
-65.40
-65.41
Me
O
NO2
t-Bu
HO
t-Bu
N
Ph
Ph
O
8t
-10
-20
-30
-40
-50
-60
-70
-80
-90
-100
-110
-120
-130
-140
-150
-160
ppm
0.57
3.00

**Chromatogram**

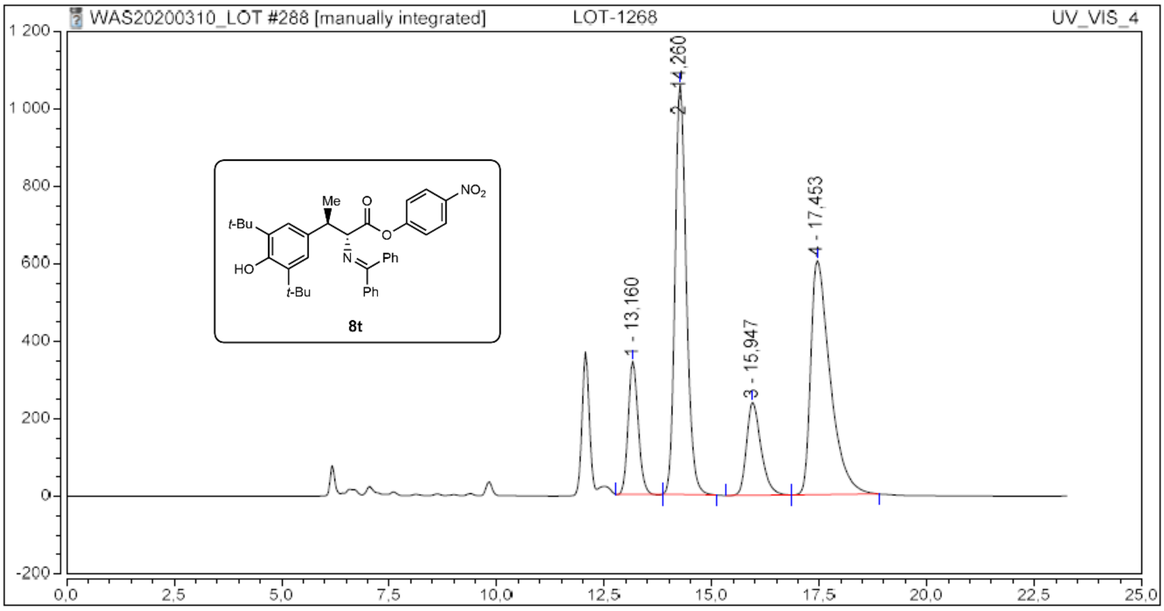


**Integration Results**

| No. | Peak Name | Retention Time min | Area mAU*min | Height mAU | Relative Area % | Relative Height % | Amount n.a. |
|---|---|---|---|---|---|---|---|
| 1 | | 13,160 | 92,488 | 343,361 | 11,20 | 15,29 | n.a. |
| 2 | | 14,260 | 322,063 | 1057,836 | 39,01 | 47,11 | n.a. |
| 3 | | 15,947 | 94,054 | 239,564 | 11,39 | 10,67 | n.a. |
| 4 | | 17,453 | 317,021 | 604,636 | 38,40 | 26,93 | n.a. |
| | | | **825,626** | **2245,396** | **100,00** | **100,00** | |

**Chromatogram**

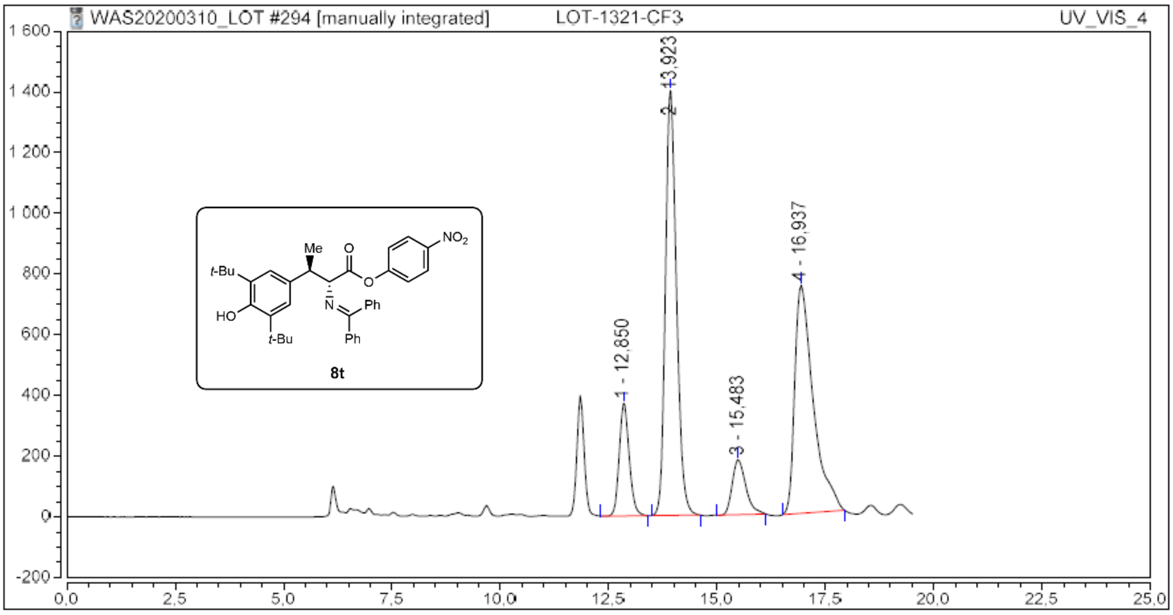


**Integration Results**

| No. | Peak Name | Retention Time min | Area mAU*min | Height mAU | Relative Area % | Relative Height % | Amount n.a. |
|---|---|---|---|---|---|---|---|
| 1 | | 12,850 | 98,731 | 371,829 | 10,41 | 13,74 | n.a. |
| 2 | | 13,923 | 411,387 | 1399,965 | 43,38 | 51,75 | n.a. |
| 3 | | 15,483 | 65,860 | 181,467 | 6,95 | 6,71 | n.a. |
| 4 | | 16,937 | 372,261 | 752,120 | 39,26 | 27,80 | n.a. |
| | | | **948,239** | **2705,381** | **100,00** | **100,00** | |

## Dipeptide 9a

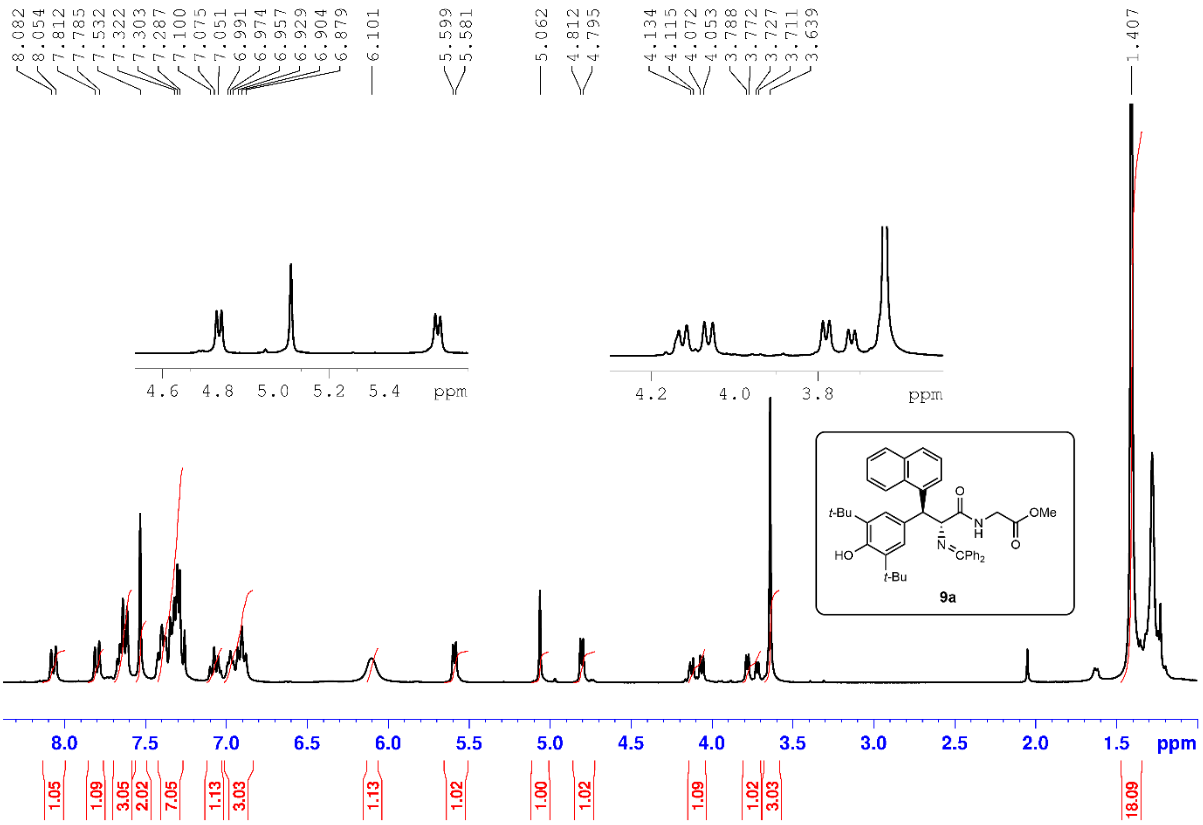


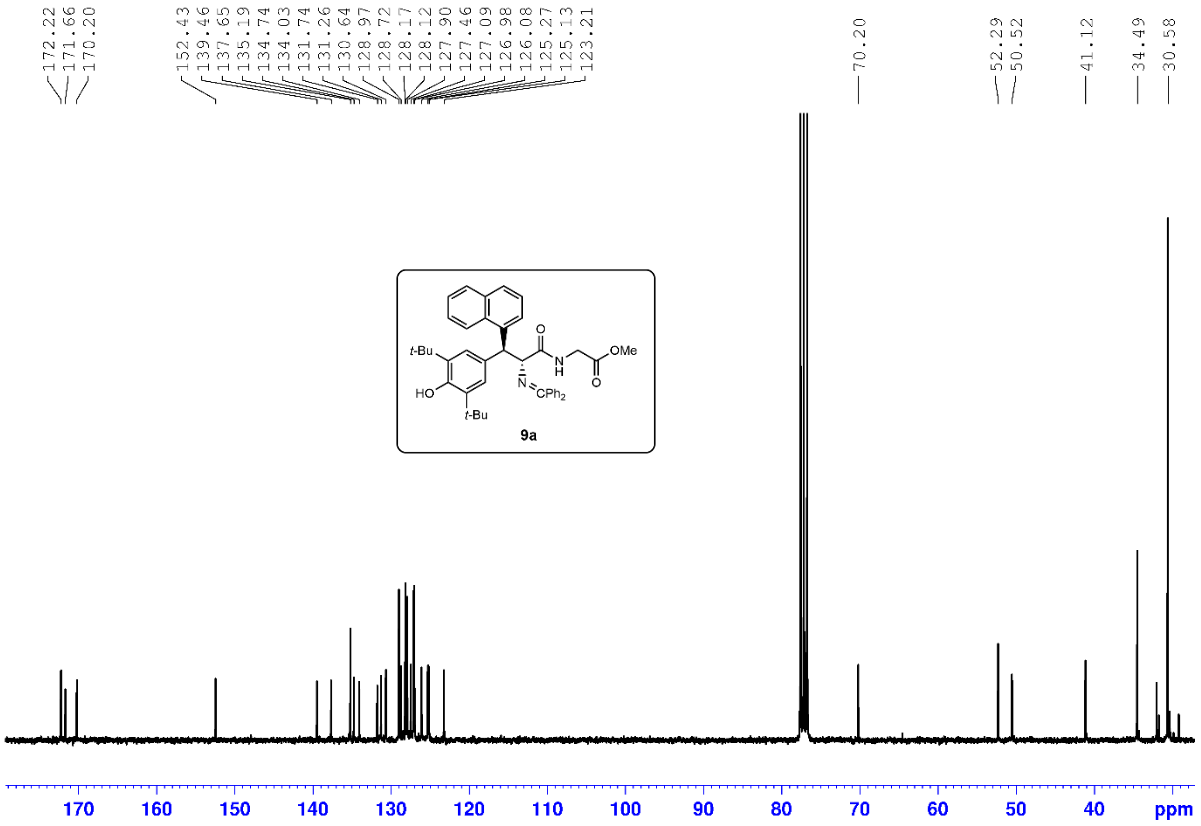

## Chromatogram

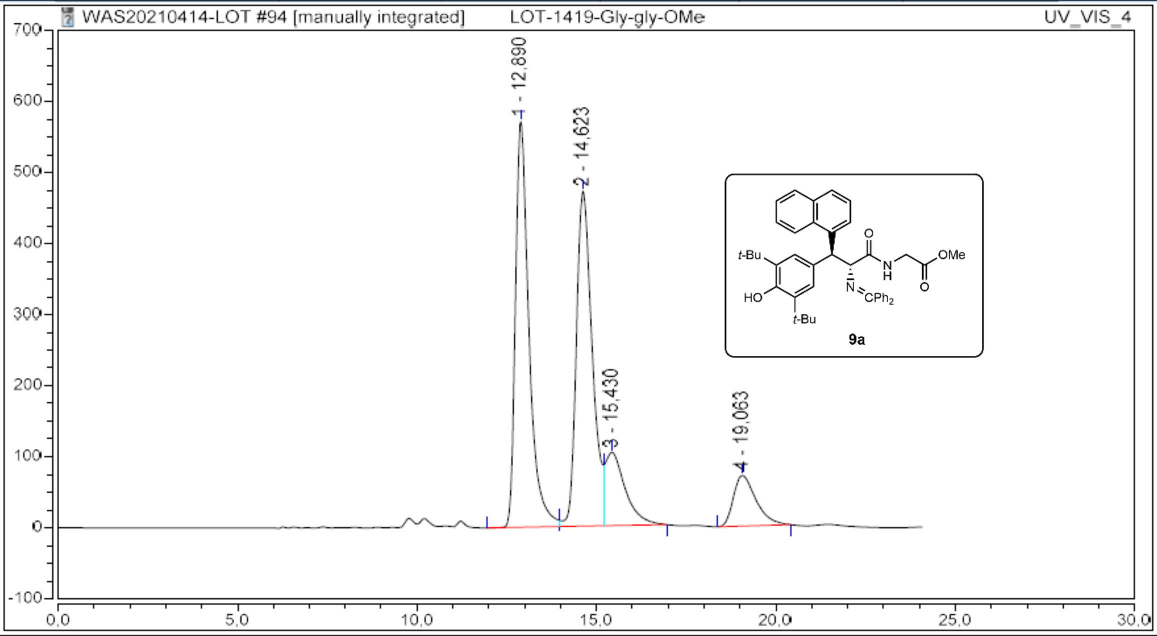


## Integration Results

| No. | Peak Name | Retention Time min | Area mAU*min | Height mAU | Relative Area % | Relative Height % | Amount n.a. |
|---|---|---|---|---|---|---|---|
| 1 | | 12,890 | 249,916 | 571,079 | 40,92 | 46,91 | n.a. |
| 2 | | 14,623 | 242,079 | 471,802 | 39,64 | 38,75 | n.a. |
| 3 | | 15,430 | 66,790 | 103,249 | 10,94 | 8,48 | n.a. |
| 4 | | 19,063 | 51,923 | 71,325 | 8,50 | 5,86 | n.a. |

## Chromatogram

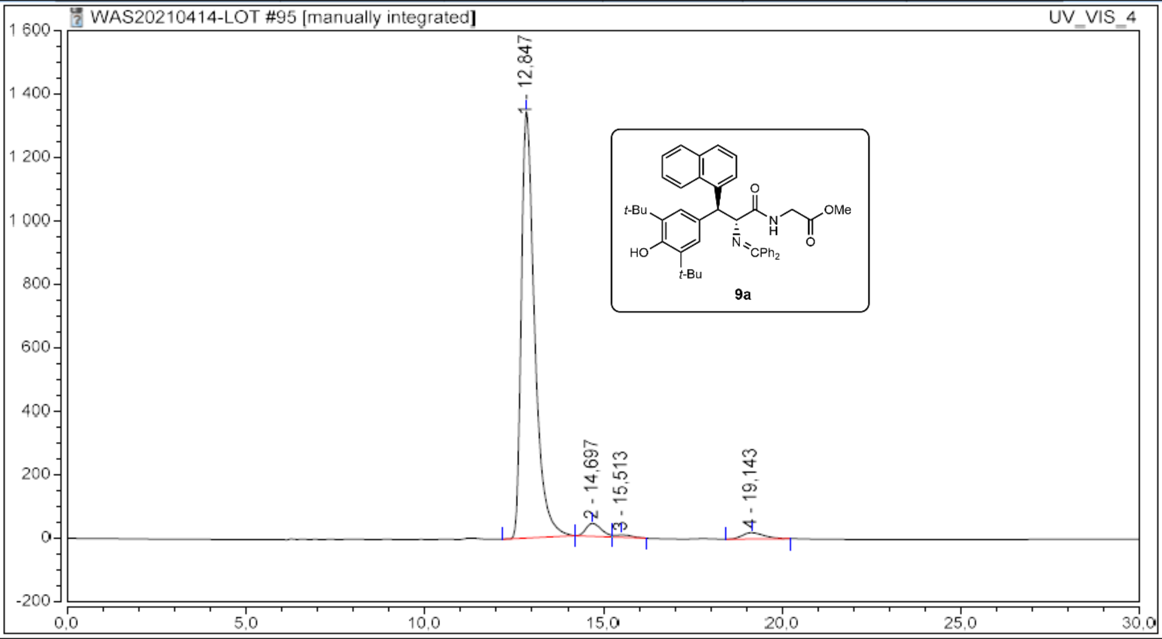


## Integration Results

| No. | Peak Name | Retention Time min | Area mAU*min | Height mAU | Relative Area % | Relative Height % | Amount n.a. |
|---|---|---|---|---|---|---|---|
| 1 | | 12,847 | 581,252 | 1345,222 | 93,75 | 95,16 | n.a. |
| 2 | | 14,697 | 20,378 | 40,863 | 3,29 | 2,89 | n.a. |
| 3 | | 15,513 | 4,379 | 7,741 | 0,71 | 0,55 | n.a. |
| 4 | | 19,143 | 14,011 | 19,804 | 2,26 | 1,40 | n.a. |

**Thiolester 11**

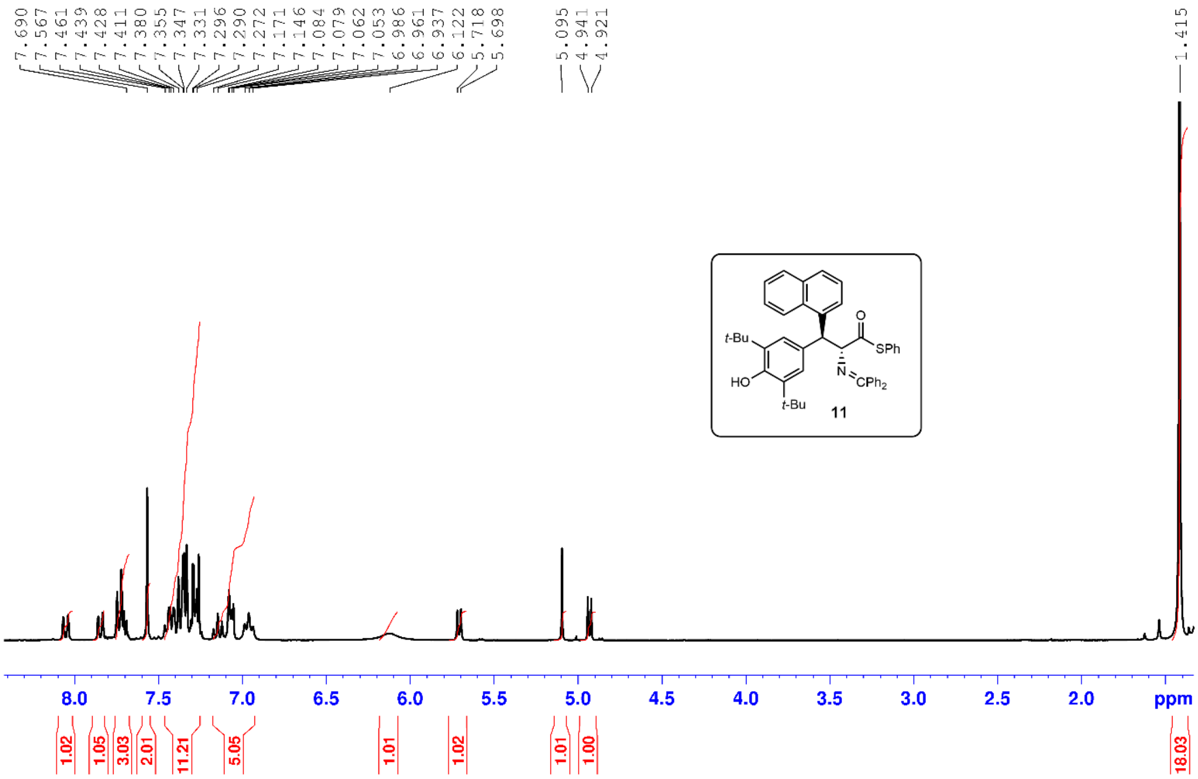


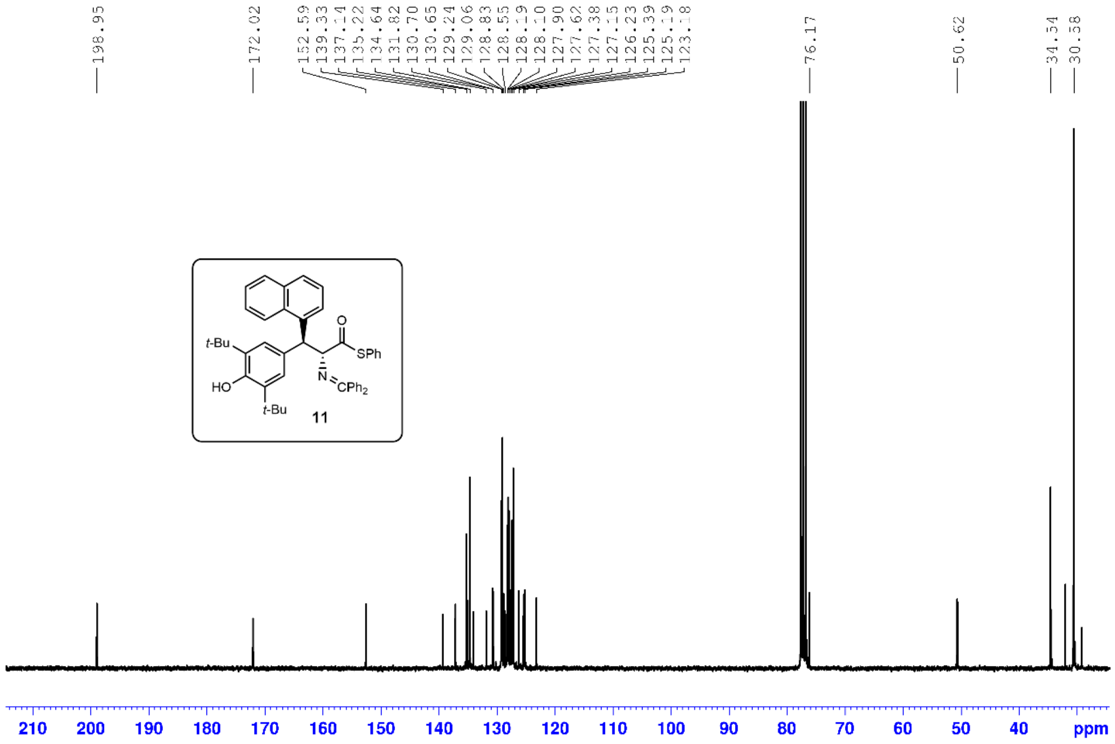

## Chromatogram

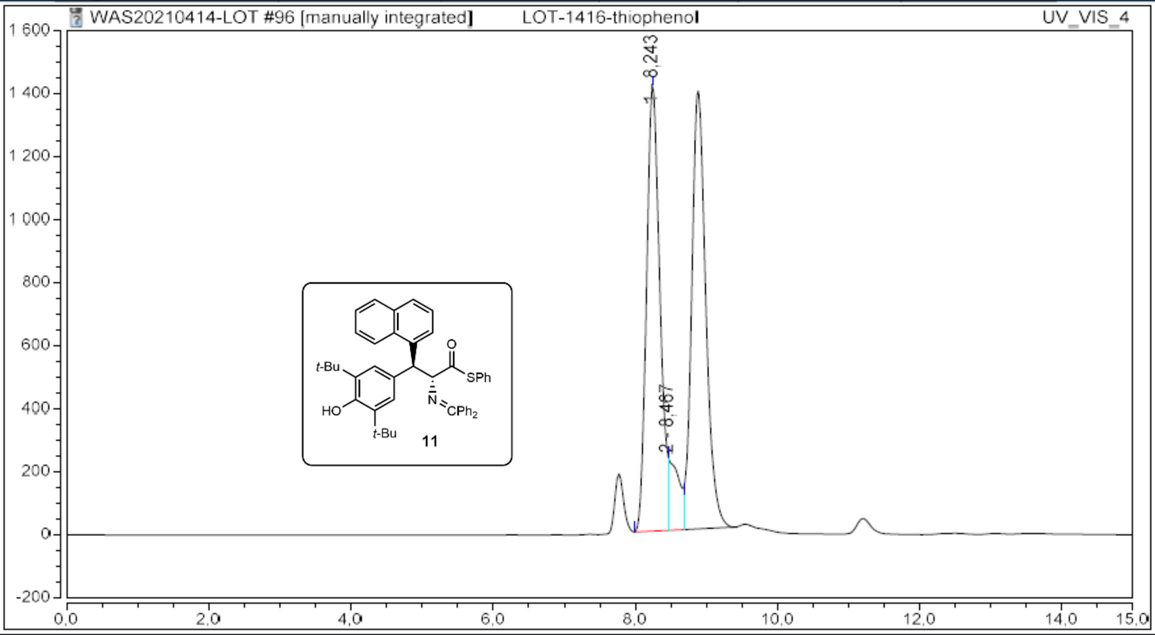


## Integration Results

| No. | Peak Name | Retention Time<br>min | Area<br>mAU*min | Height<br>mAU | Relative Area<br>% | Relative Height<br>% | Amount<br>n.a. |
|---|---|---|---|---|---|---|---|
| 1 | | 8,243 | 305,213 | 1410,250 | 88,19 | 85,95 | n.a. |
| 2 | | 8,467 | 40,885 | 230,550 | 11,81 | 14,05 | n.a. |
| **Total:** | | | **346,098** | **1640,800** | **100,00** | **100,00** | |

## Chromatogram

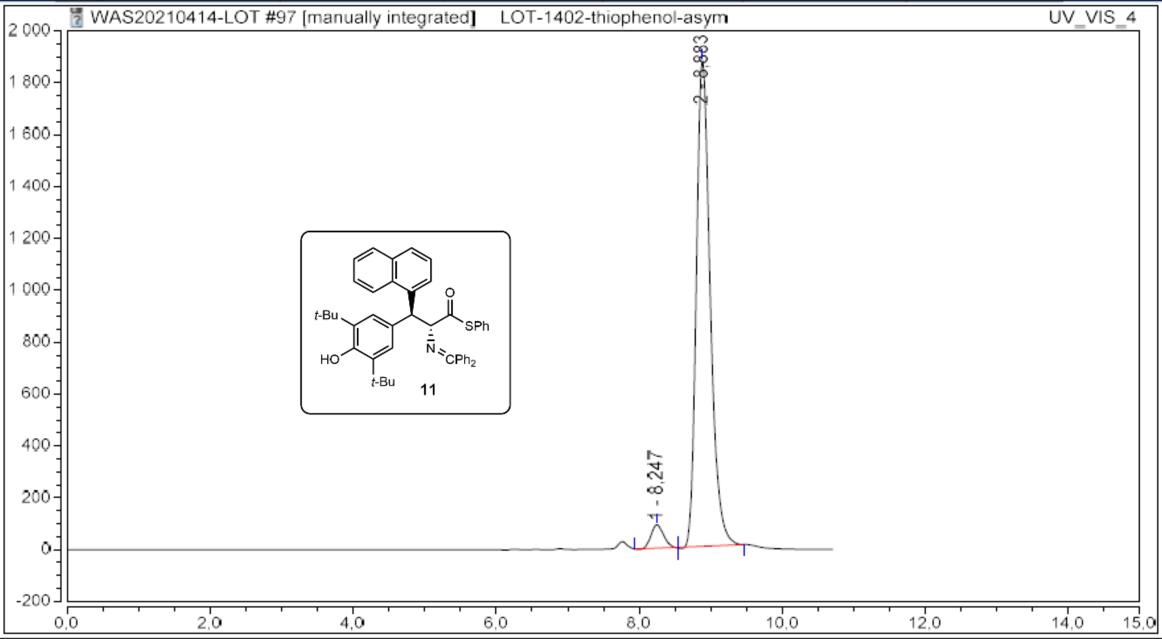


## Integration Results

| No. | Peak Name | Retention Time<br>min | Area<br>mAU*min | Height<br>mAU | Relative Area<br>% | Relative Height<br>% | Amount<br>n.a. |
|---|---|---|---|---|---|---|---|
| 1 | | 8,247 | 17,890 | 90,283 | 4,09 | 4,60 | n.a. |
| 2 | | 8,883 | 419,981 | 1872,985 | 95,91 | 95,40 | n.a. |
| **Total:** | | | **437,871** | **1963,268** | **100,00** | **100,00** | |

**Benzyl amide 9c**

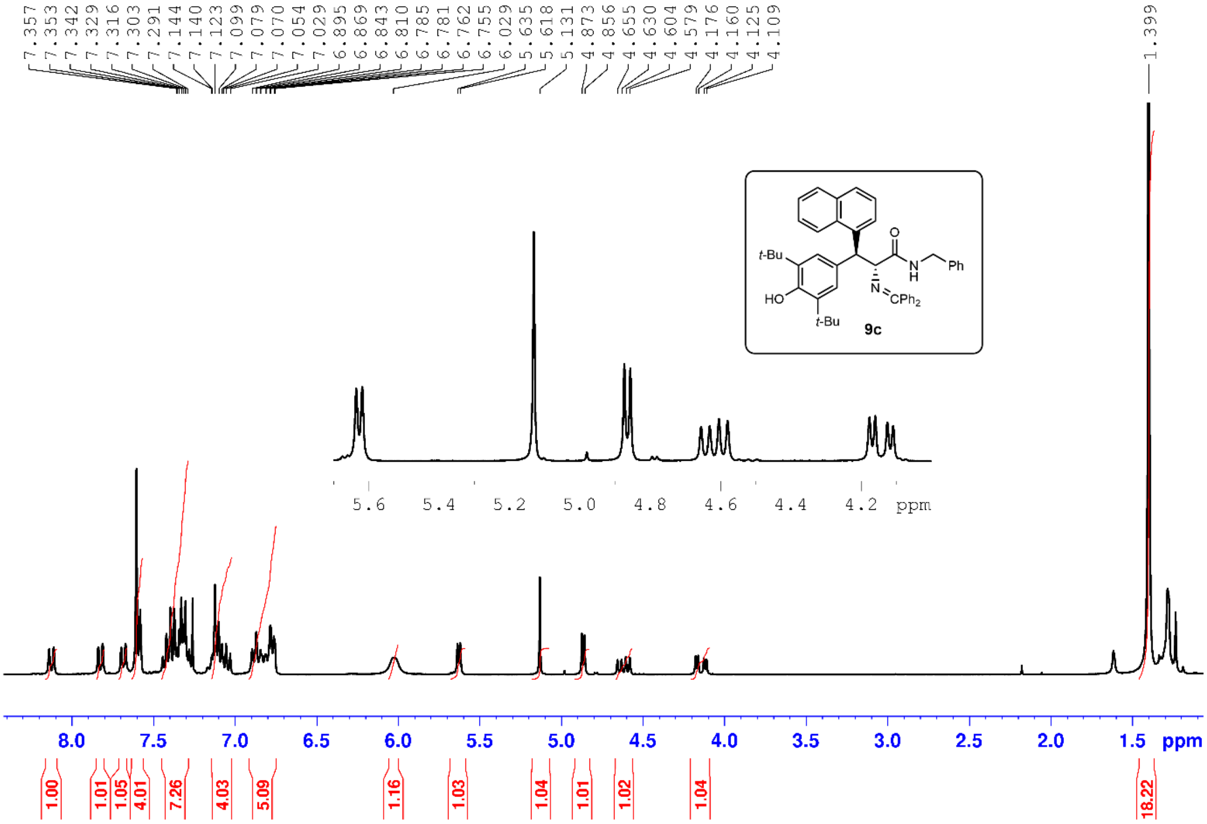


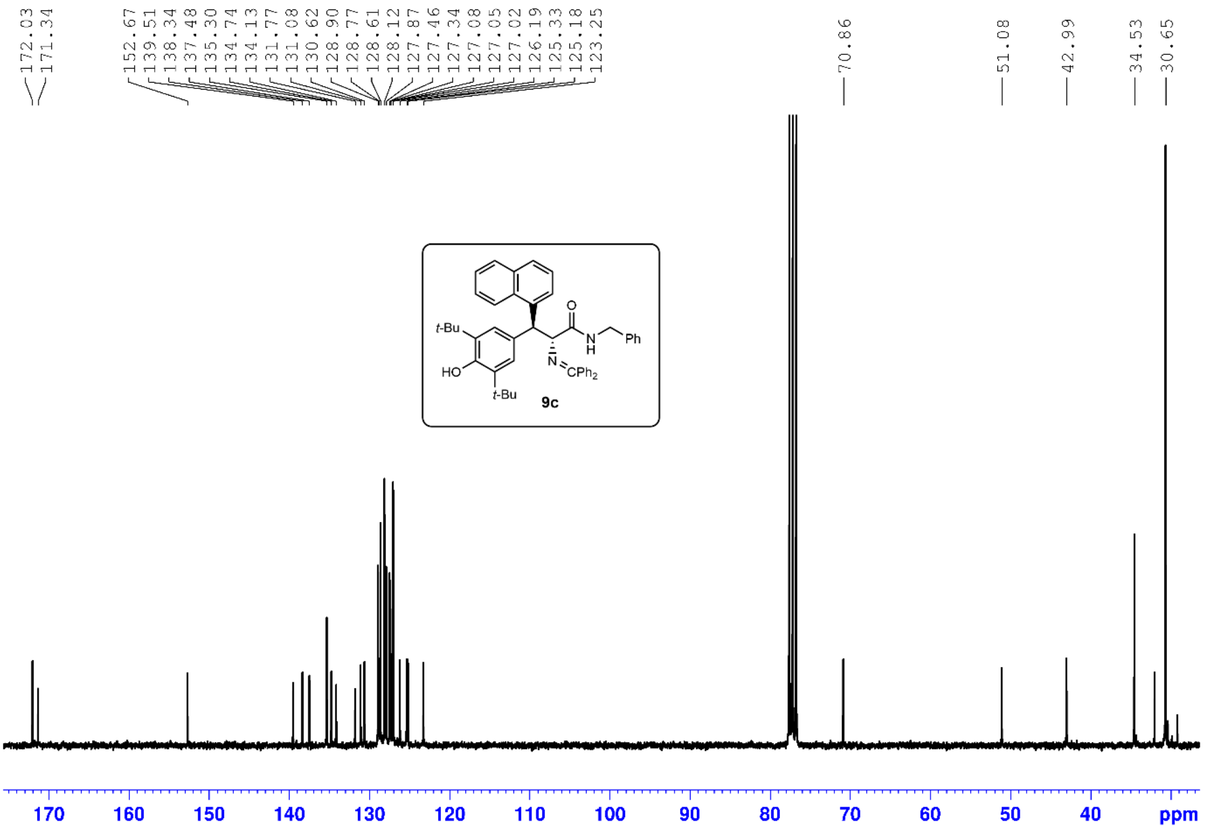

## Chromatogram

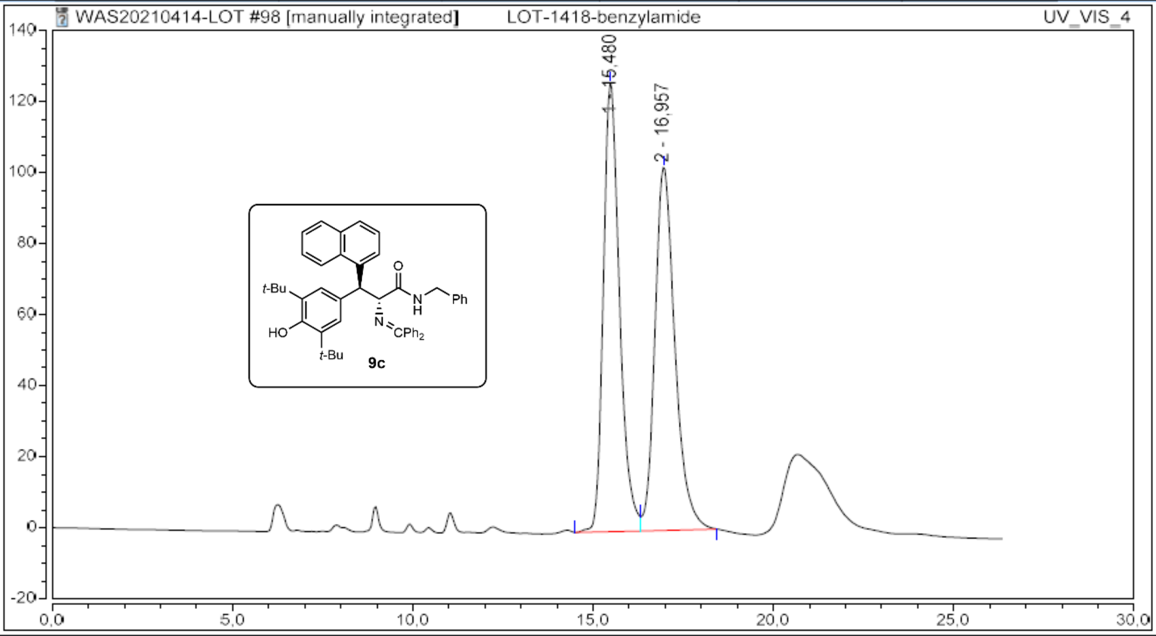


## Integration Results

| No. | Peak Name | Retention Time min | Area mAU*min | Height mAU | Relative Area % | Relative Height % | Amount n.a. |
|---|---|---|---|---|---|---|---|
| 1 | | 15,480 | 65,817 | 126,422 | 49,99 | 55,26 | n.a. |
| 2 | | 16,957 | 65,839 | 102,360 | 50,01 | 44,74 | n.a. |
| **Total:** | | | **131,656** | **228,782** | **100,00** | **100,00** | |

## Chromatogram

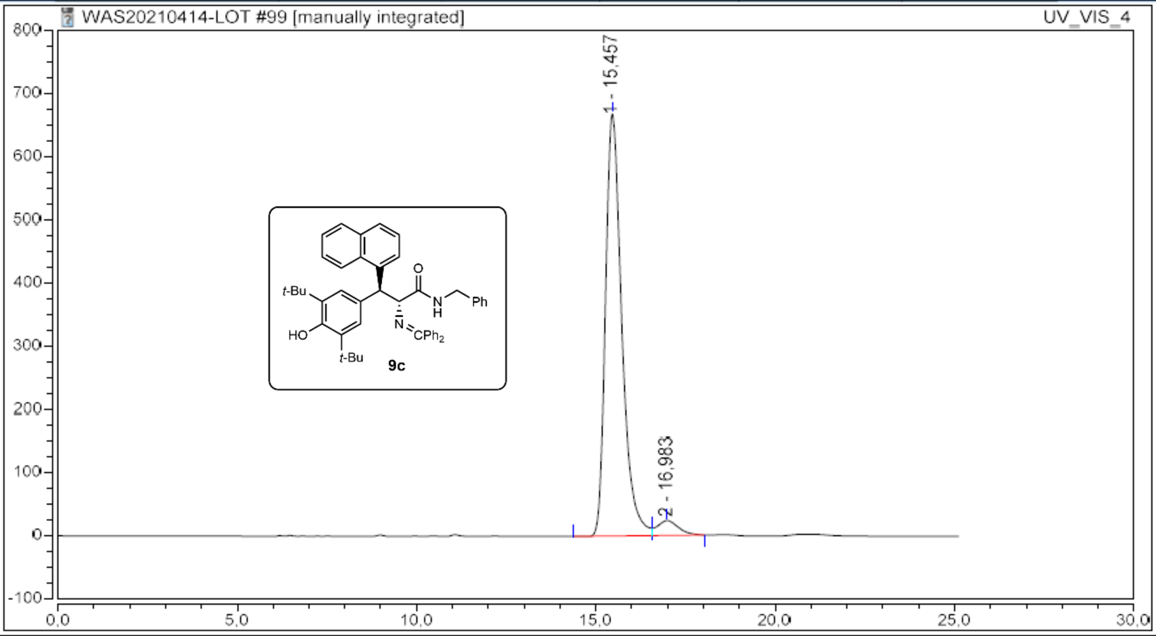


## Integration Results

| No. | Peak Name | Retention Time min | Area mAU*min | Height mAU | Relative Area % | Relative Height % | Amount n.a. |
|---|---|---|---|---|---|---|---|
| 1 | | 15,457 | 352,172 | 668,700 | 95,66 | 96,59 | n.a. |
| 2 | | 16,983 | 15,960 | 23,594 | 4,34 | 3,41 | n.a. |
| **Total:** | | | **368,132** | **692,294** | **100,00** | **100,00** | |

## *p*-Cl Benzylamide 9d

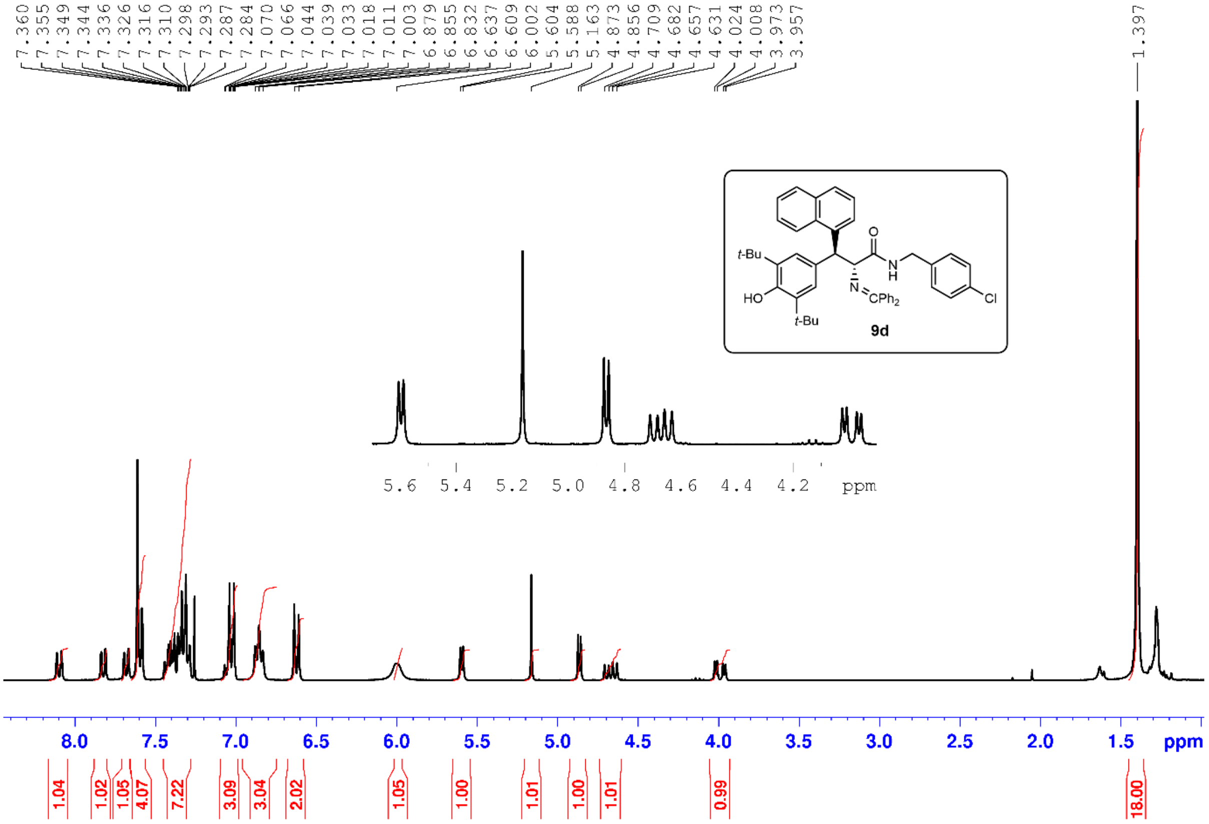


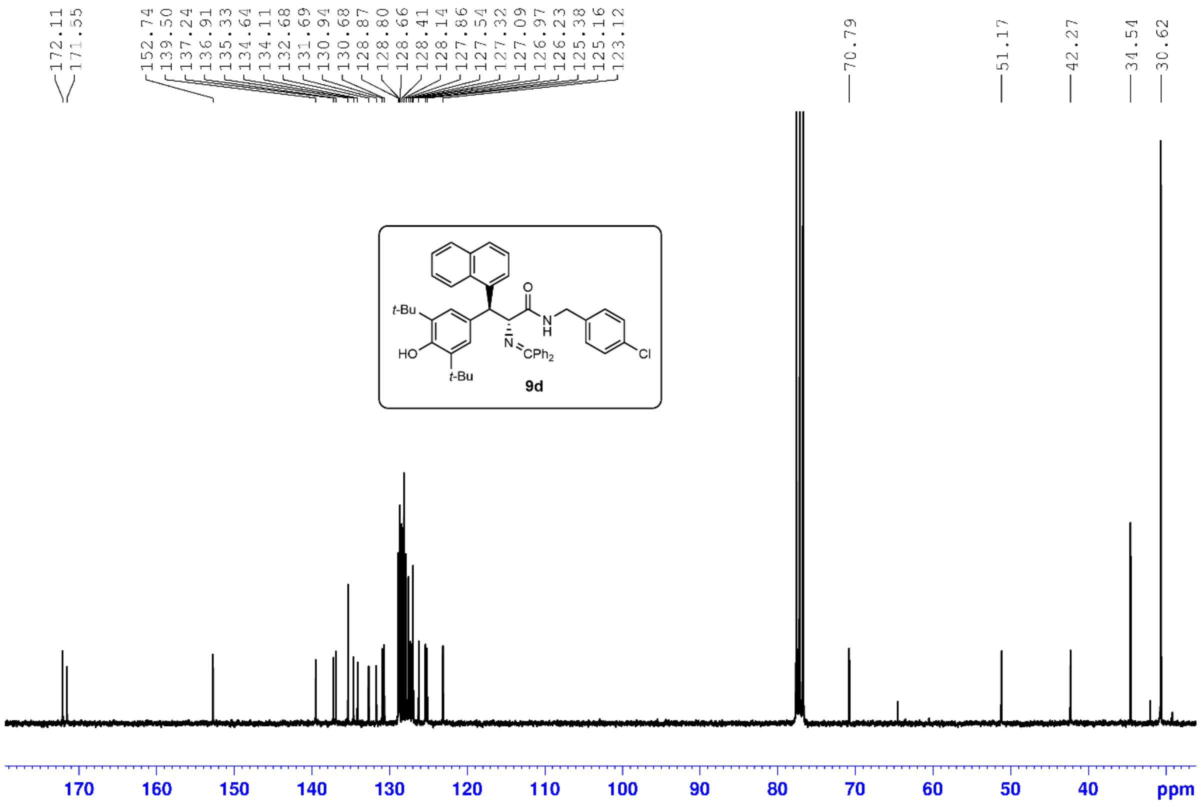

## Chromatogram

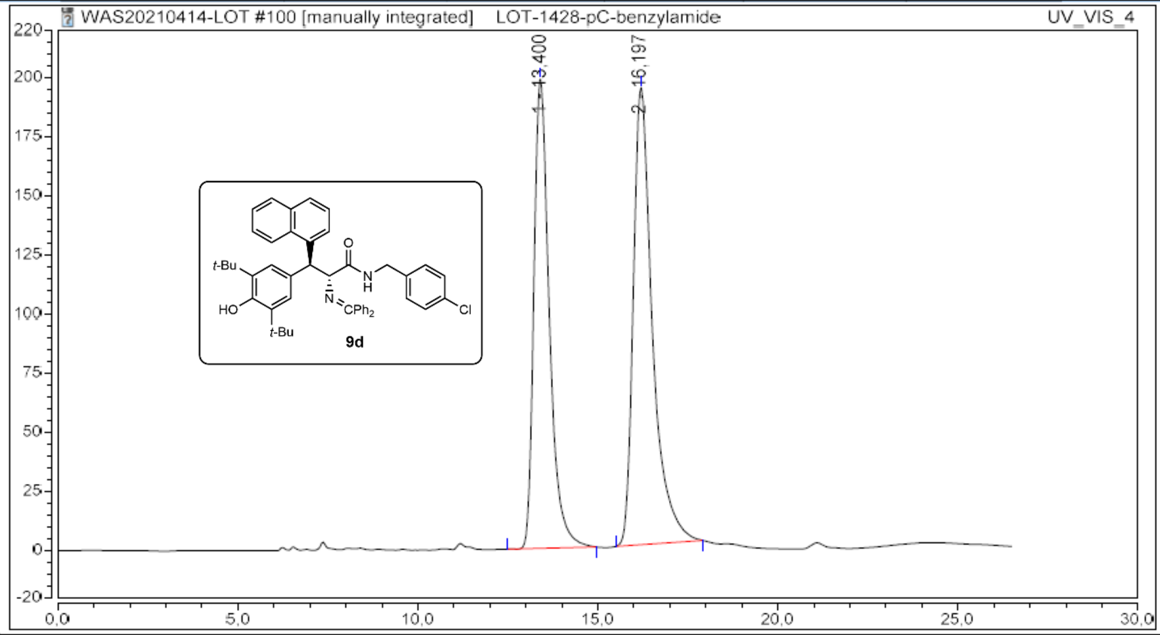


## Integration Results

| No. | Peak Name | Retention Time min | Area mAU*min | Height mAU | Relative Area % | Relative Height % | Amount n.a. |
|---|---|---|---|---|---|---|---|
| 1 | | 13,400 | 98,627 | 198,479 | 45,29 | 50,66 | n.a. |
| 2 | | 16,197 | 119,142 | 193,303 | 54,71 | 49,34 | n.a. |
| **Total:** | | | **217,769** | **391,782** | **100,00** | **100,00** | |

## Chromatogram

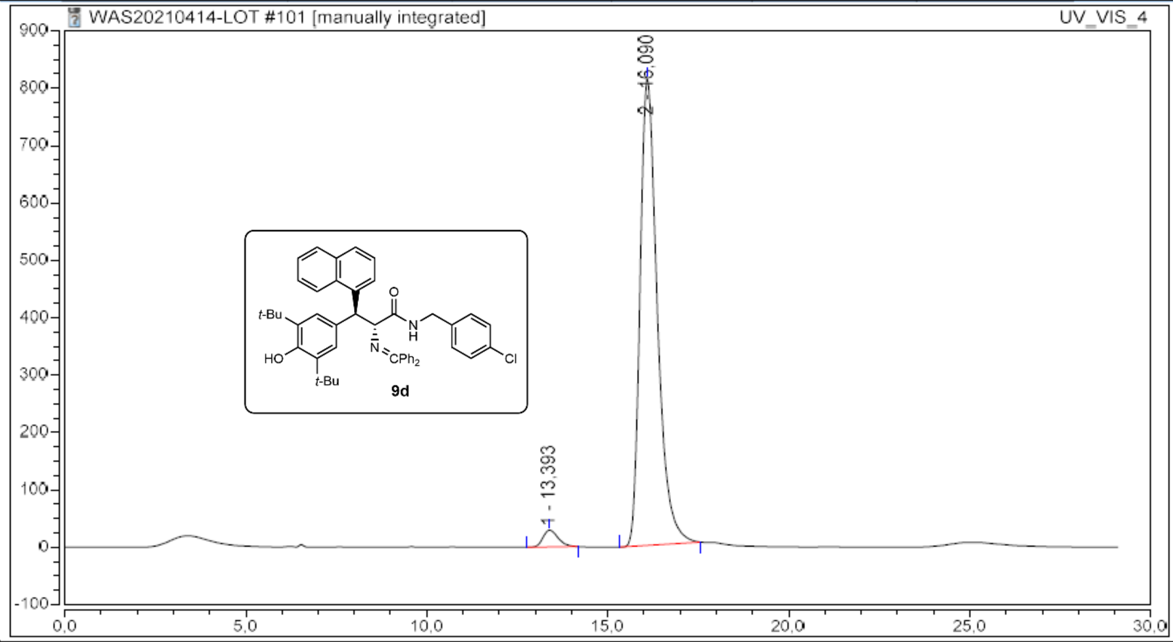


## Integration Results

| No. | Peak Name | Retention Time min | Area mAU*min | Height mAU | Relative Area % | Relative Height % | Amount n.a. |
|---|---|---|---|---|---|---|---|
| 1 | | 13,393 | 14,207 | 29,419 | 3,07 | 3,49 | n.a. |
| 2 | | 16,090 | 449,086 | 813,280 | 96,93 | 96,51 | n.a. |
| **Total:** | | | **463,293** | **842,699** | **100,00** | **100,00** | |

## Morpholine amide 9b

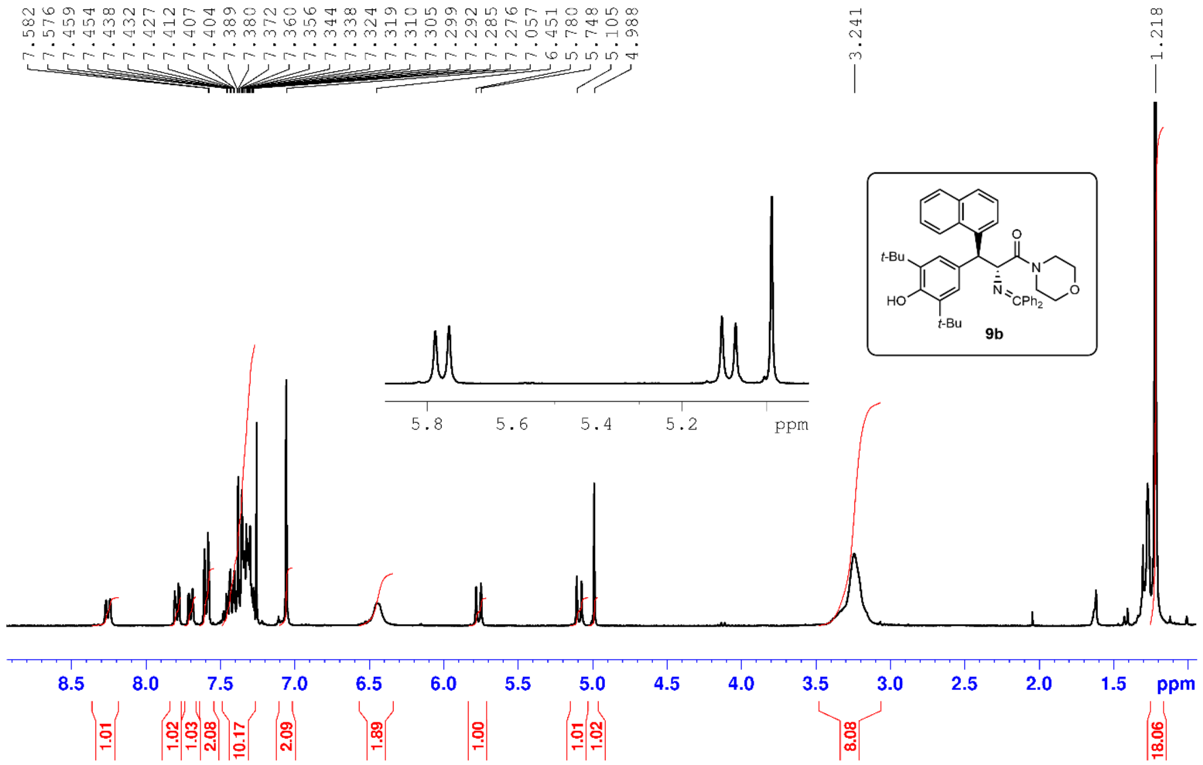


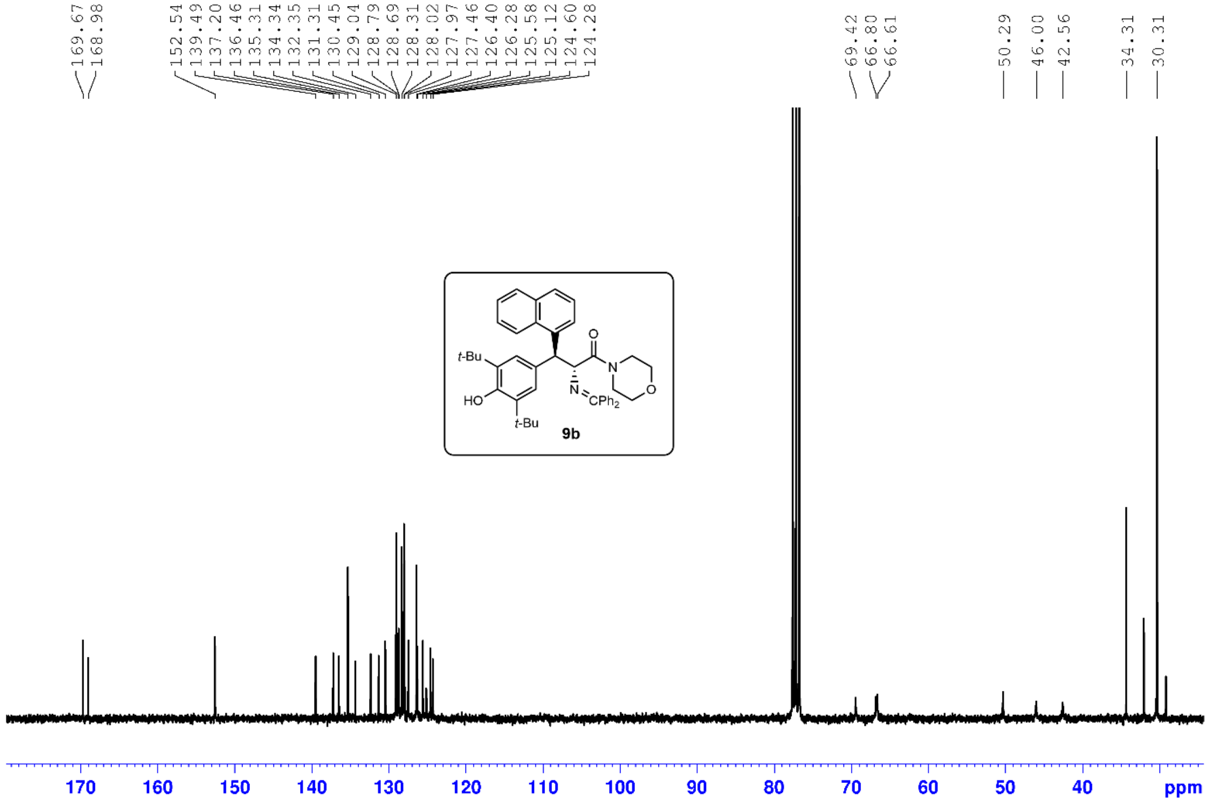

## Chromatogram

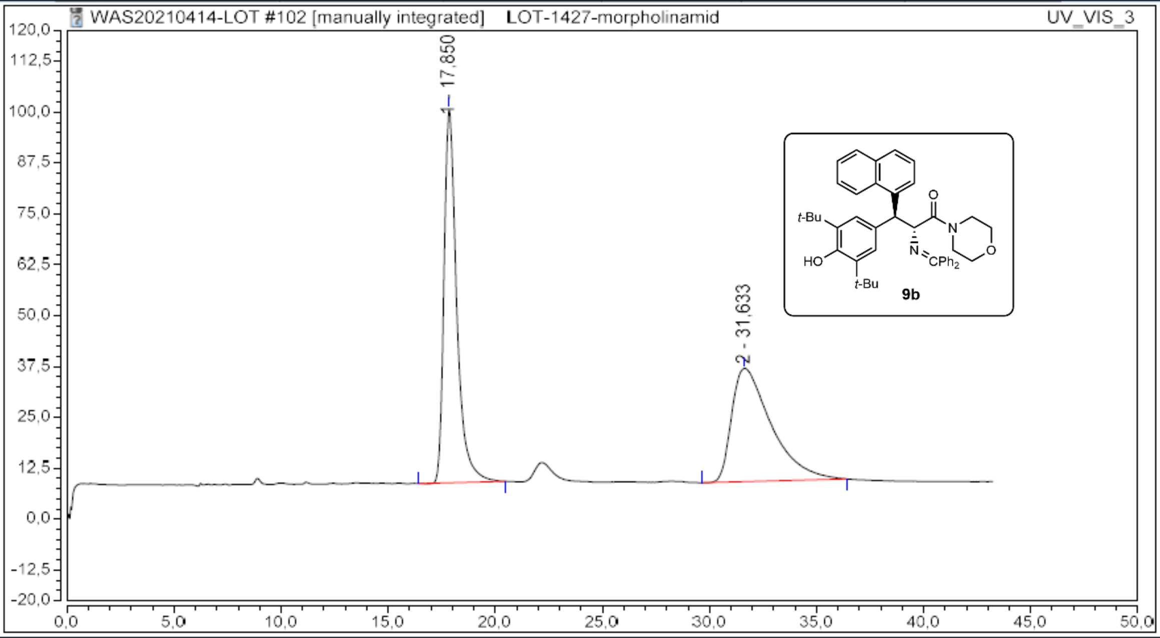


## Integration Results

| No. | Peak Name | Retention Time min | Area mAU*min | Height mAU | Relative Area % | Relative Height % | Amount n.a. |
|---|---|---|---|---|---|---|---|
| 1 | | 17,850 | 63,239 | 91,802 | 51,52 | 76,73 | n.a. |
| 2 | | 31,633 | 59,510 | 27,841 | 48,48 | 23,27 | n.a. |
| **Total:** | | | **122,748** | **119,642** | **100,00** | **100,00** | |

## Chromatogram

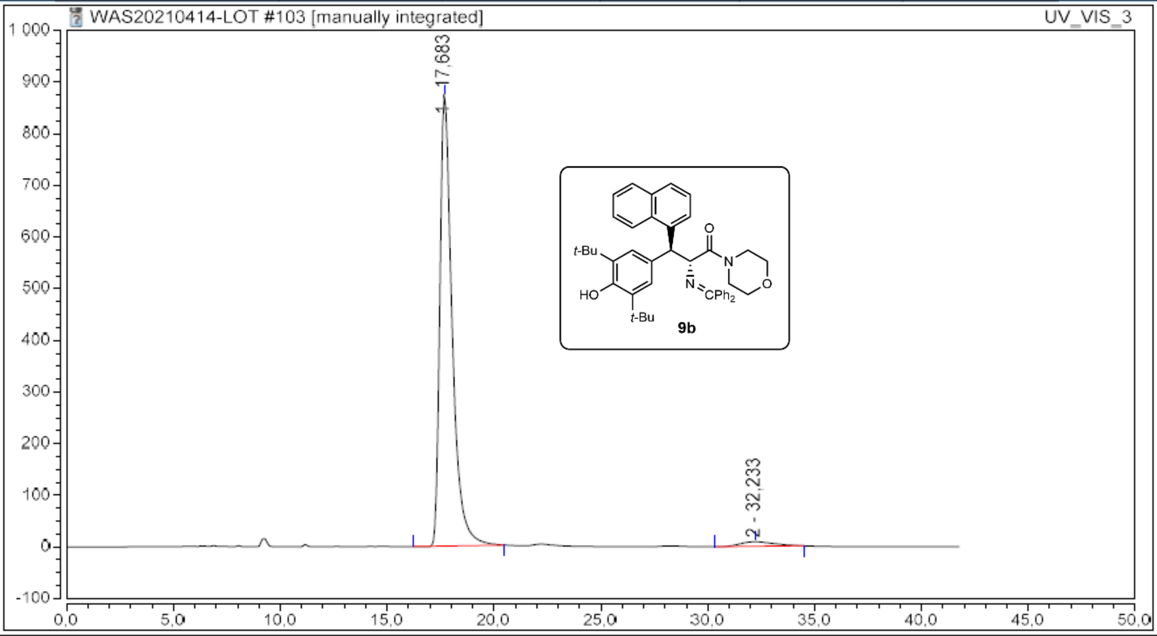


## Integration Results

| No. | Peak Name | Retention Time min | Area mAU*min | Height mAU | Relative Area % | Relative Height % | Amount n.a. |
|---|---|---|---|---|---|---|---|
| 1 | | 17,683 | 582,972 | 870,312 | 97,38 | 99,03 | n.a. |
| 2 | | 32,233 | 15,696 | 8,523 | 2,62 | 0,97 | n.a. |
| **Total:** | | | **598,668** | **878,836** | **100,00** | **100,00** | |

# Amine 10

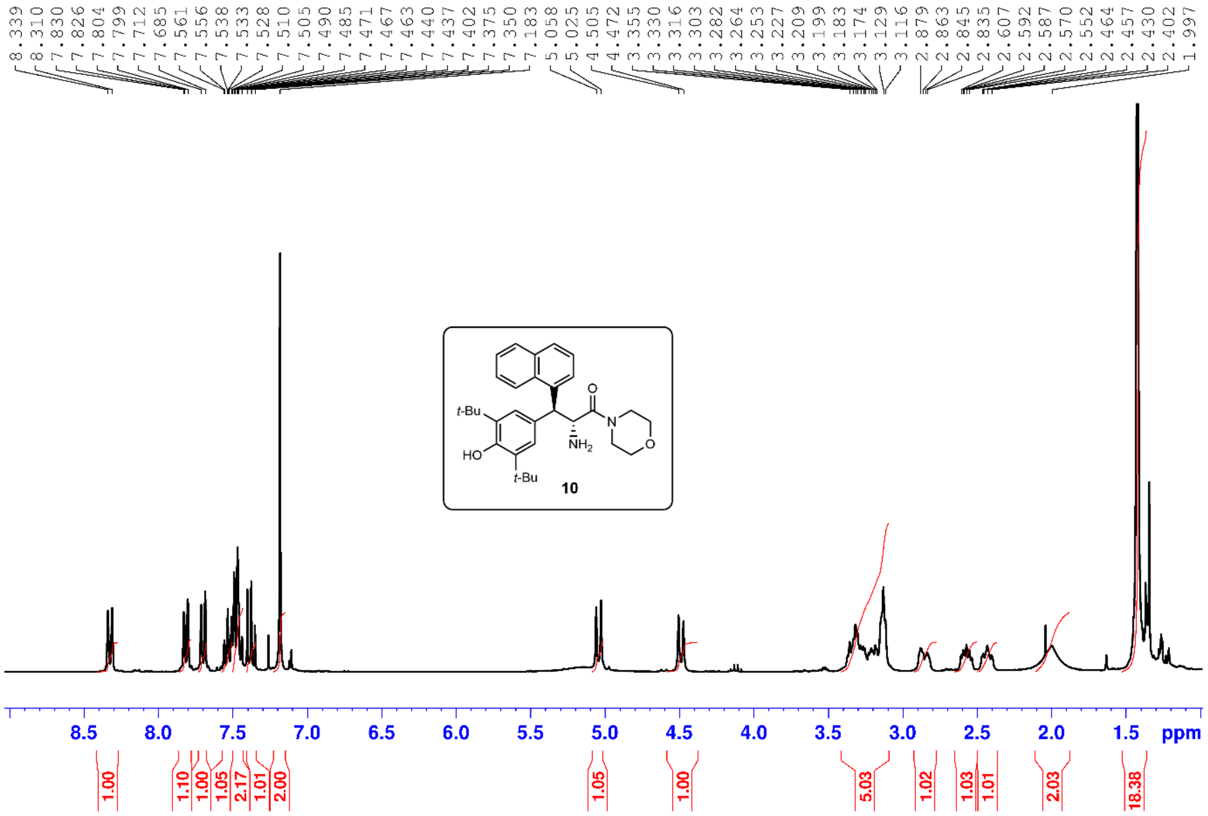
8.339
8.310
7.830
7.826
7.804
7.799
7.712
7.685
7.561
7.556
7.538
7.533
7.528
7.510
7.505
7.490
7.485
7.471
7.467
7.463
7.440
7.437
7.402
7.375
7.350
7.183
5.058
5.025
4.505
4.472
3.355
3.330
3.316
3.303
3.282
3.264
3.253
3.227
3.209
3.199
3.183
3.174
3.129
3.116
2.879
2.863
2.845
2.835
2.607
2.592
2.587
2.570
2.552
2.464
2.457
2.430
2.402
1.997
t-Bu
HO
t-Bu
O
N
O
NH2
10
8.5
8.0
7.5
7.0
6.5
6.0
5.5
5.0
4.5
4.0
3.5
3.0
2.5
2.0
1.5
ppm
1.00
1.10
1.00
1.05
2.17
1.01
2.00
1.05
1.00
5.03
1.02
1.03
1.01
2.03
18.38

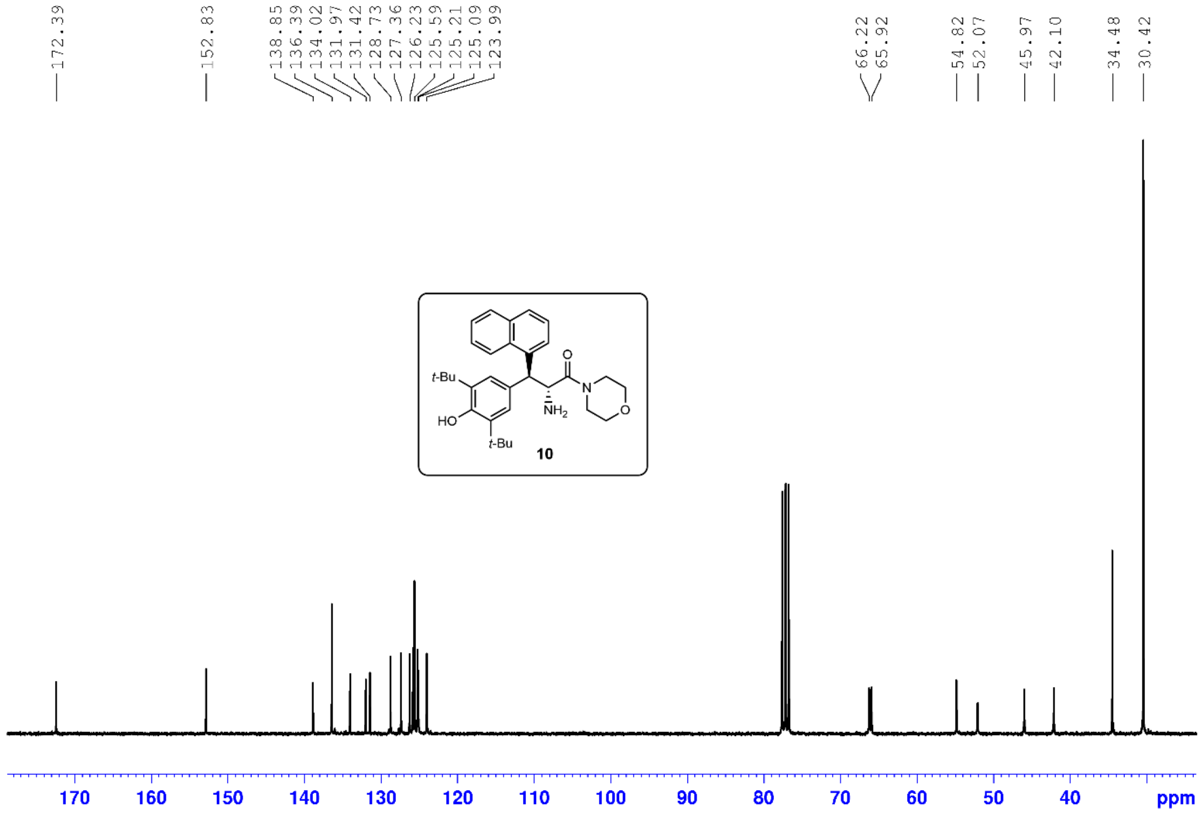
172.39
152.83
138.85
136.39
134.02
131.97
131.42
128.73
127.36
126.23
125.59
125.21
125.09
123.99
66.22
65.92
54.82
52.07
45.97
42.10
34.48
30.42
t-Bu
HO
t-Bu
O
N
O
NH2
10
170
160
150
140
130
120
110
100
90
80
70
60
50
40
ppm

## Chromatogram

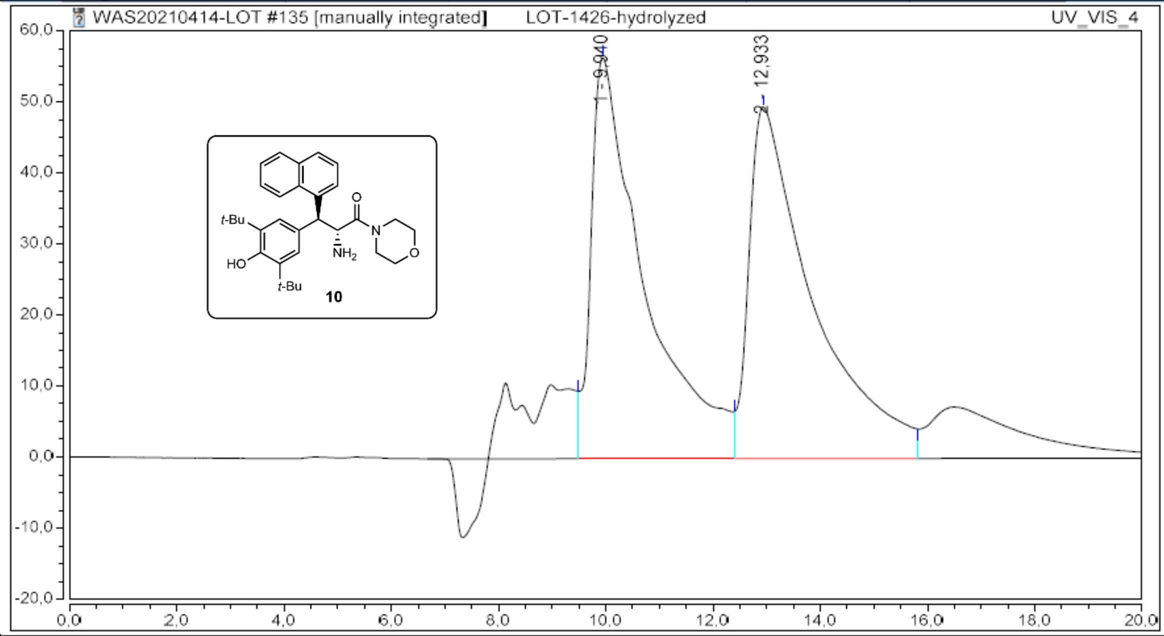


## Integration Results

| No. | Peak Name | Retention Time min | Area mAU*min | Height mAU | Relative Area % | Relative Height % | Amount n.a. |
|---|---|---|---|---|---|---|---|
| 1 | | 9,940 | 64,881 | 56,484 | 48,79 | 53,27 | n.a. |
| 2 | | 12,933 | 68,094 | 49,540 | 51,21 | 46,73 | n.a. |
| **Total:** | | | **132,975** | **106,024** | **100,00** | **100,00** | |

## Chromatogram

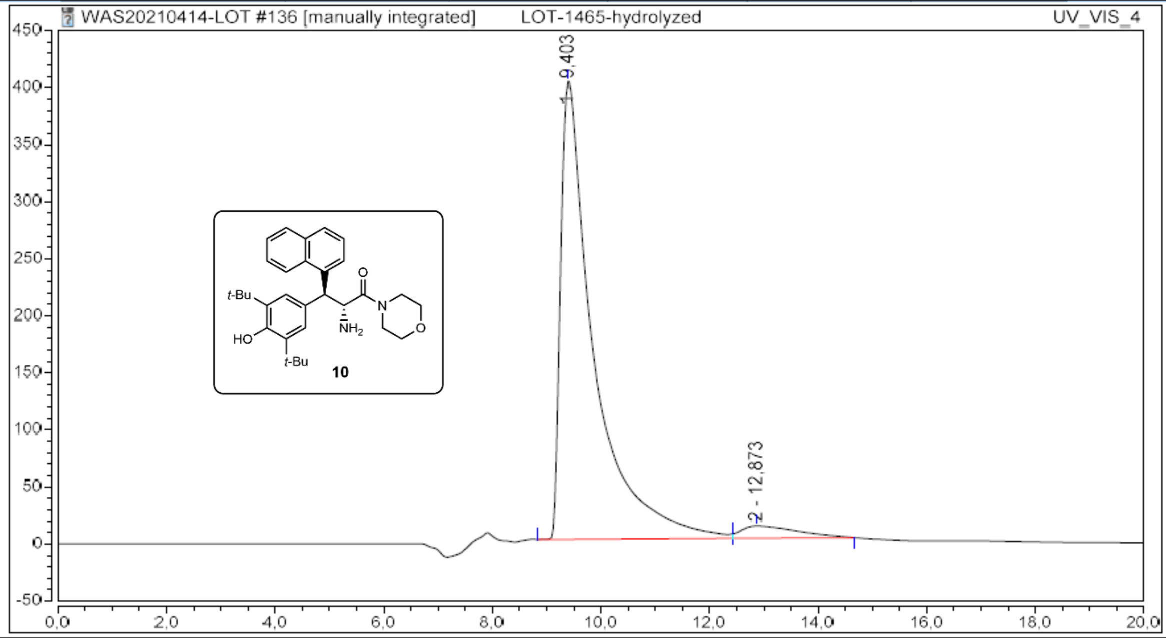


## Integration Results

| No. | Peak Name | Retention Time min | Area mAU*min | Height mAU | Relative Area % | Relative Height % | Amount n.a. |
|---|---|---|---|---|---|---|---|
| 1 | | 9,403 | 282,853 | 401,804 | 95,64 | 97,39 | n.a. |
| 2 | | 12,873 | 12,908 | 10,762 | 4,36 | 2,61 | n.a. |
| **Total:** | | | **295,762** | **412,566** | **100,00** | **100,00** | |

**Methyl ester 12**

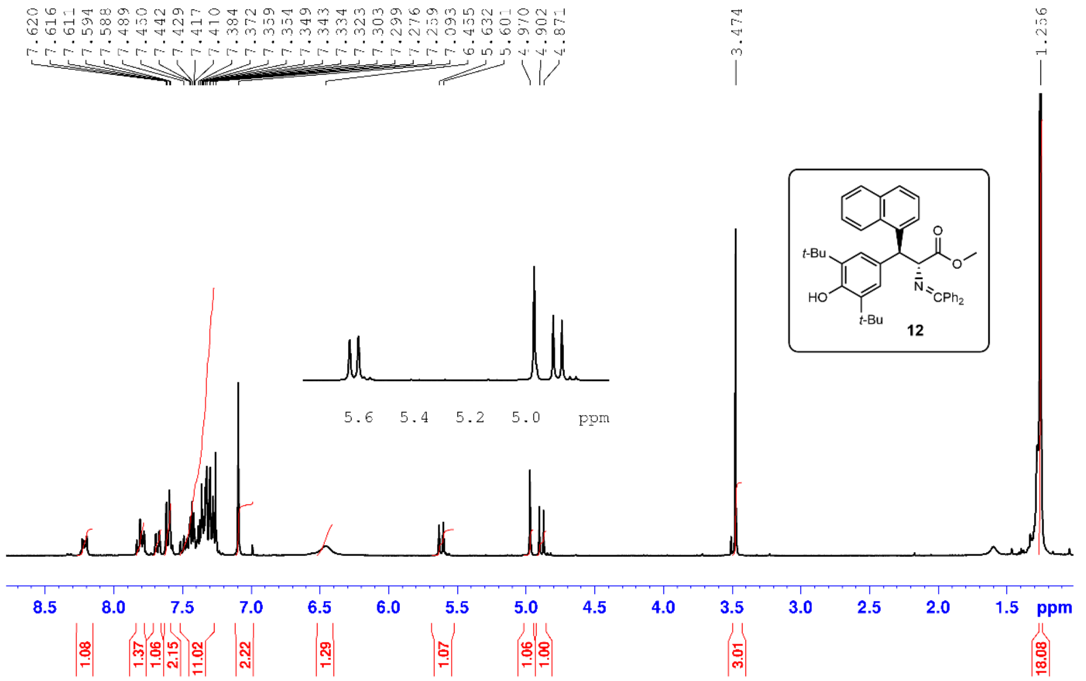


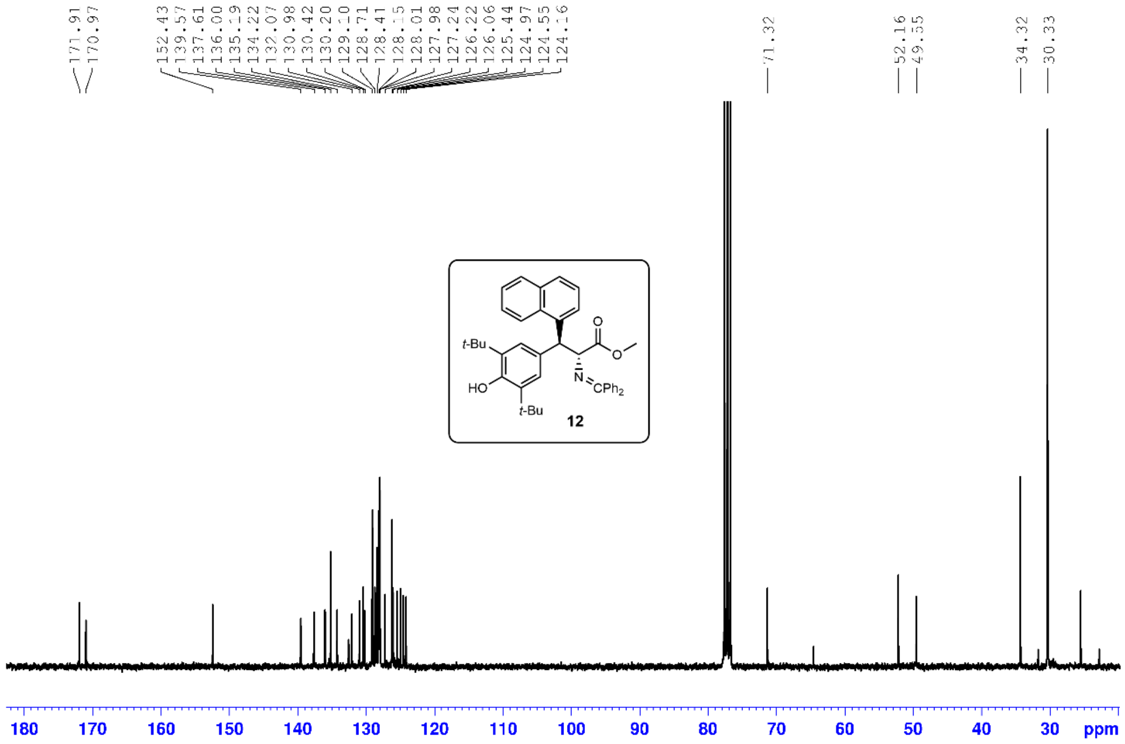

**Chromatogram**

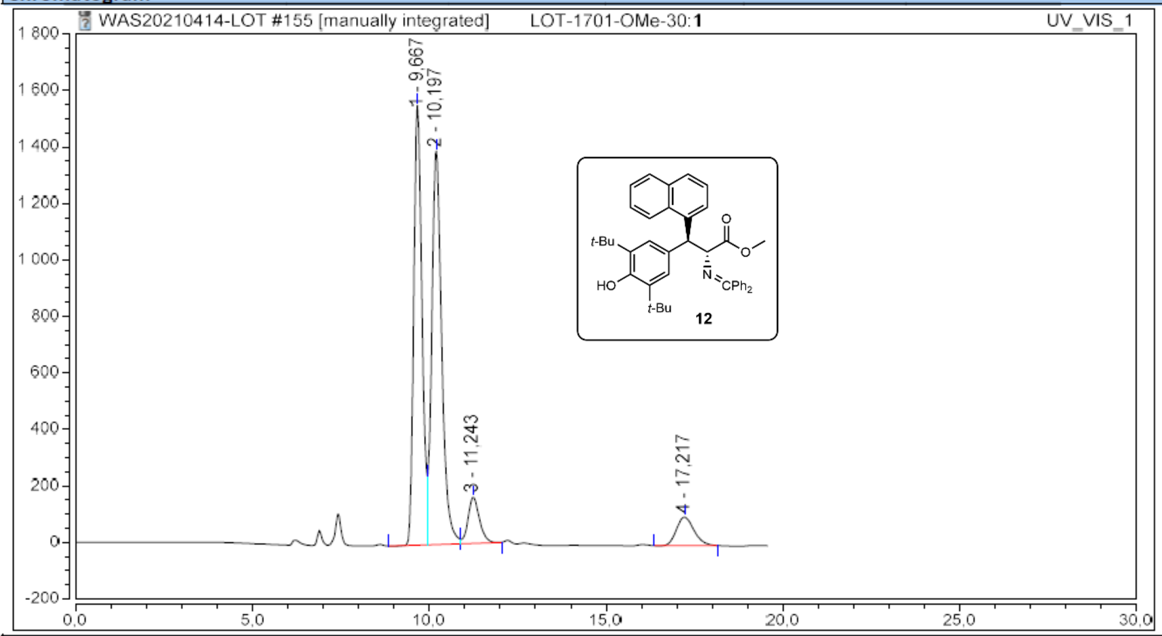


**Integration Results**

| No. | Peak Name | Retention Time<br>min | Area<br>mAU*min | Height<br>mAU | Relative Area<br>% | Relative Height<br>% | Amount<br>n.a. |
|---|---|---|---|---|---|---|---|
| 1 | | 9,667 | 417,567 | 1556,867 | 42,16 | 48,42 | n.a. |
| 2 | | 10,197 | 452,996 | 1392,988 | 45,74 | 43,33 | n.a. |
| 3 | | 11,243 | 61,691 | 164,189 | 6,23 | 5,11 | n.a. |
| 4 | | 17,217 | 58,220 | 101,088 | 5,88 | 3,14 | n.a. |
| **Total:** | | | **990,474** | **3215,131** | **100,00** | **100,00** | |

**Chromatogram**

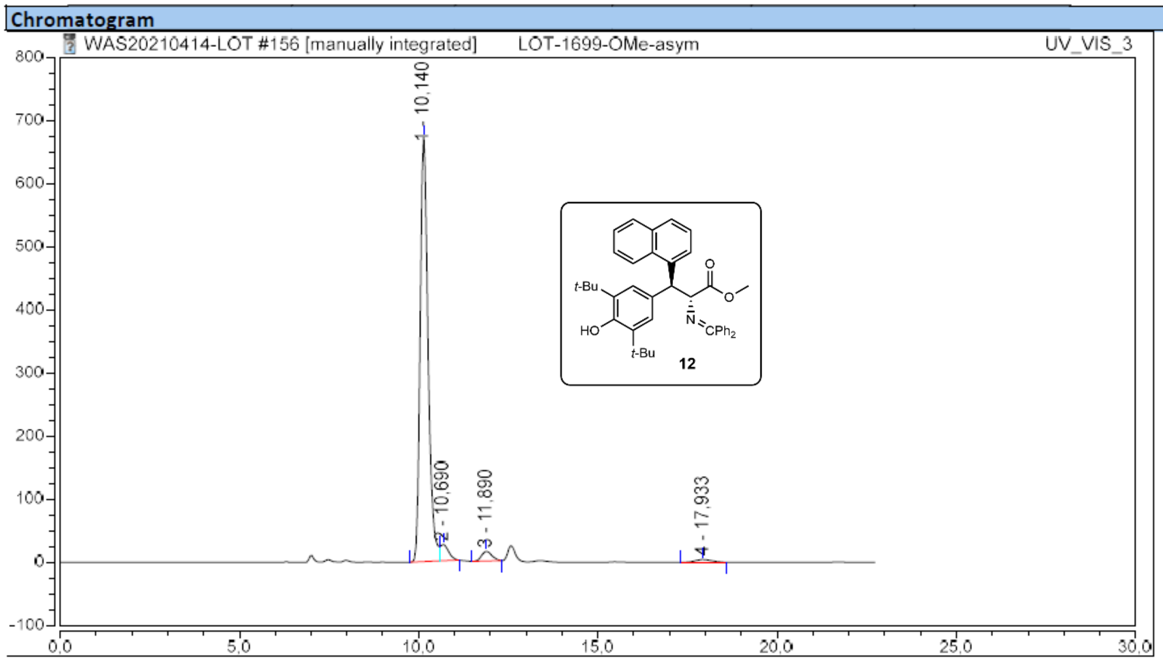


**Integration Results**

| No. | Peak Name | Retention Time<br>min | Area<br>mAU*min | Height<br>mAU | Relative Area<br>% | Relative Height<br>% | Amount<br>n.a. |
|---|---|---|---|---|---|---|---|
| 1 | | 10,140 | 172,280 | 672,801 | 92,11 | 93,70 | n.a. |
| 2 | | 10,690 | 7,313 | 25,691 | 3,91 | 3,58 | n.a. |
| 3 | | 11,890 | 5,089 | 15,195 | 2,72 | 2,12 | n.a. |
| 4 | | 17,933 | 2,352 | 4,356 | 1,26 | 0,61 | n.a. |
| **Total:** | | | **187,034** | **718,042** | **100,00** | **100,00** | |

**4-(3,5-di-tert-butyl-4-hydroxyphenyl)-3-((diphenylmethylene)amino)chroman-2-one 14a**

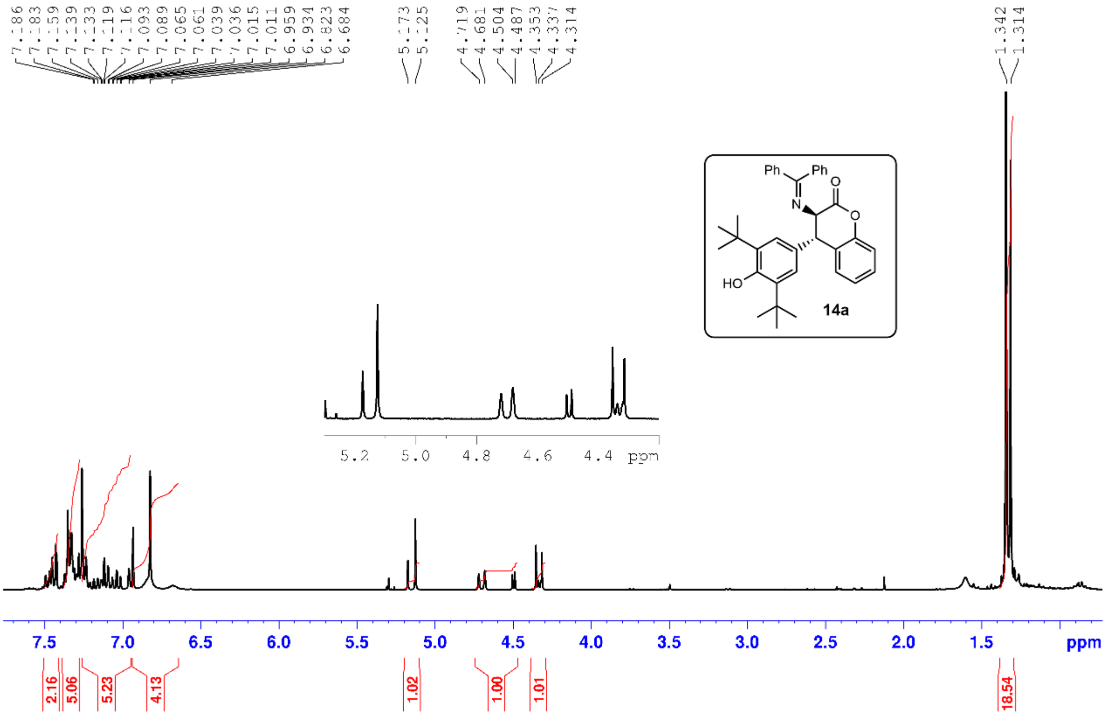


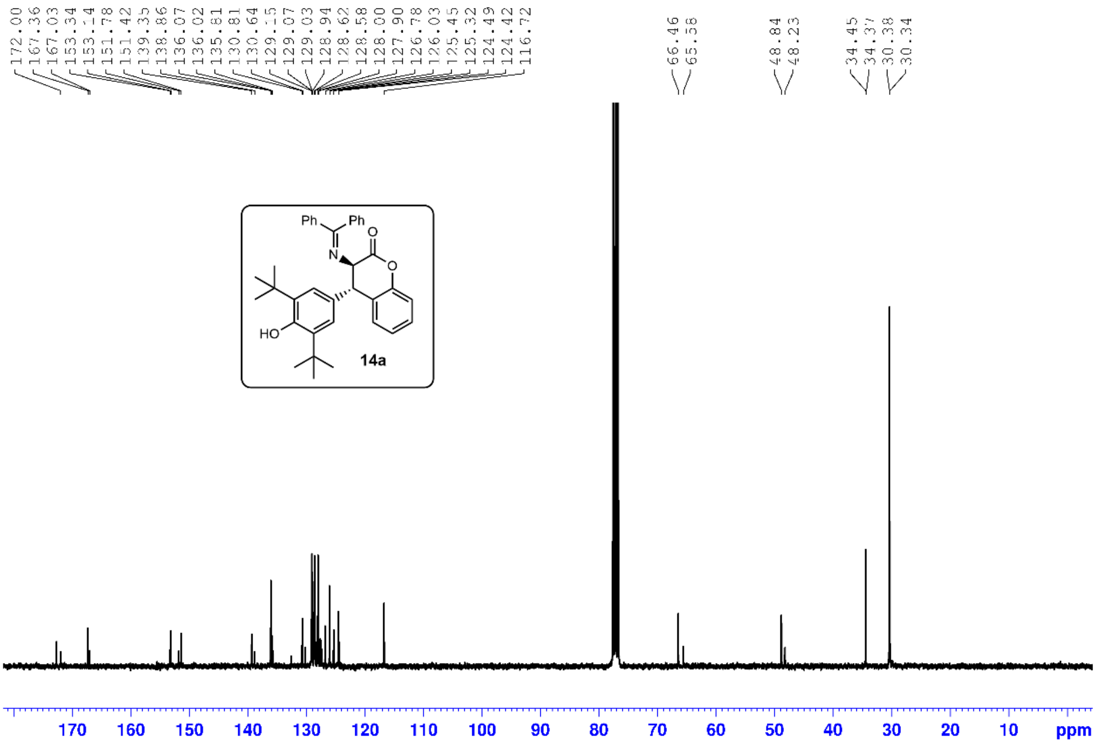

**Chromatogram**

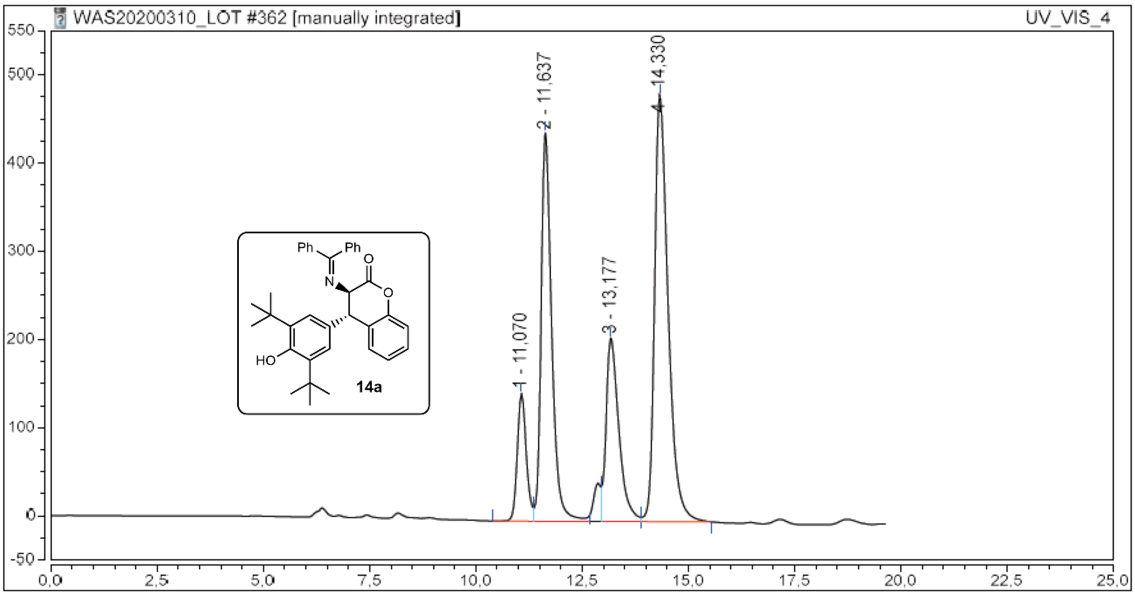


**Integration Results**

| No. | Peak Name | Retention Time min | Area mAU*min | Height mAU | Relative Area % | Relative Height % | Amount n.a. |
|---|---|---|---|---|---|---|---|
| 1 | | 11,070 | 35,487 | 145,235 | 8,57 | 11,37 | n.a. |
| 2 | | 11,637 | 127,137 | 440,503 | 30,72 | 34,47 | n.a. |
| 3 | | 13,177 | 72,481 | 208,118 | 17,51 | 16,29 | n.a. |
| 4 | | 14,330 | 178,796 | 483,906 | 43,20 | 37,87 | n.a. |
| | | | **413,900** | **1277,763** | **100,00** | **100,00** | |

**Chromatogram**

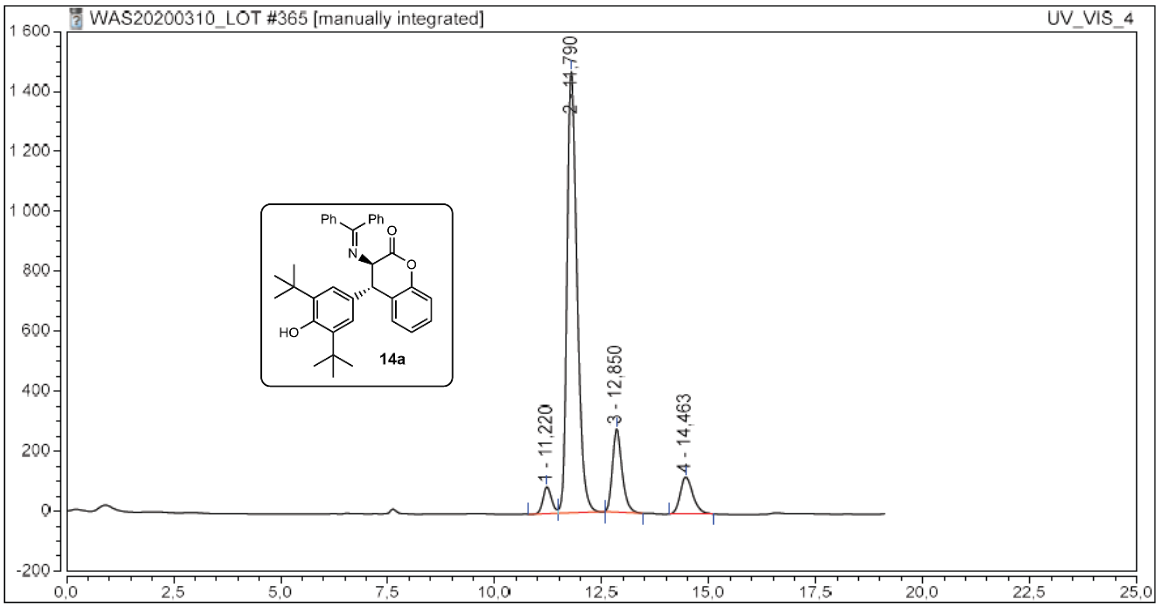


**Integration Results**

| No. | Peak Name | Retention Time min | Area mAU*min | Height mAU | Relative Area % | Relative Height % | Amount n.a. |
|---|---|---|---|---|---|---|---|
| 1 | | 11,220 | 20,703 | 89,575 | 3,87 | 4,56 | n.a. |
| 2 | | 11,790 | 398,718 | 1471,467 | 74,62 | 74,98 | n.a. |
| 3 | | 12,850 | 72,299 | 277,450 | 13,53 | 14,14 | n.a. |
| 4 | | 14,463 | 42,607 | 123,873 | 7,97 | 6,31 | n.a. |
| | | | **534,326** | **1962,366** | **100,00** | **100,00** | |

**1-(3,5-di-tert-butyl-4-hydroxyphenyl)-2-((diphenylmethylene)amino)-1,2-dihydro-3H-benzo[f]chromen-3-one 14b**

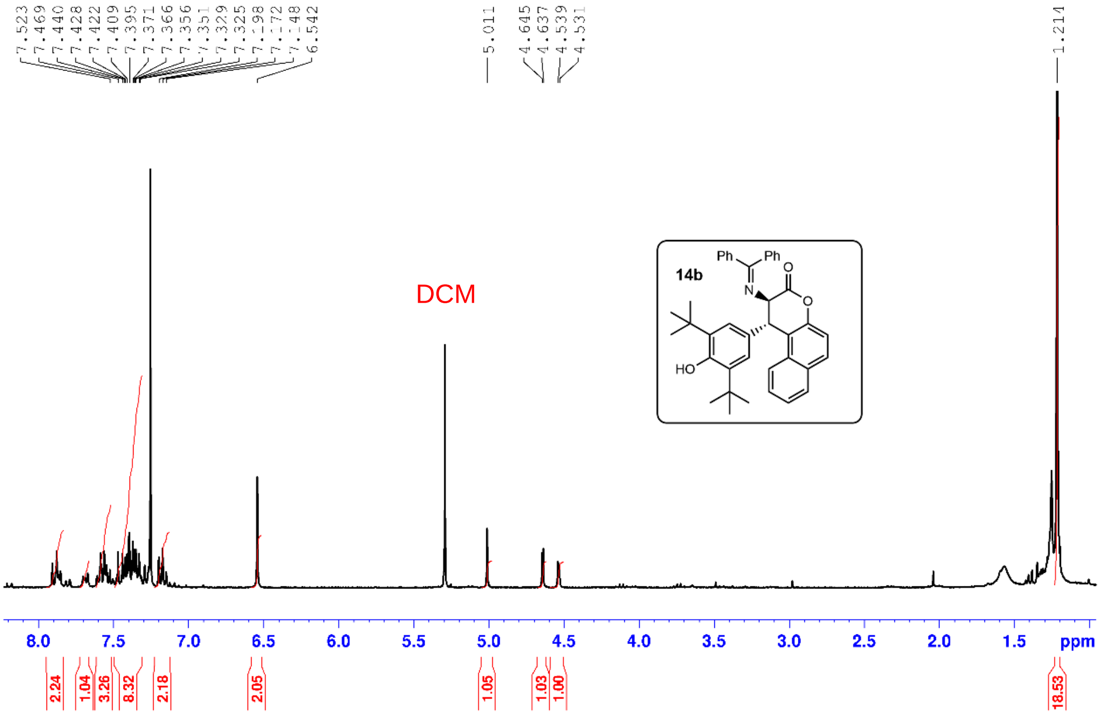


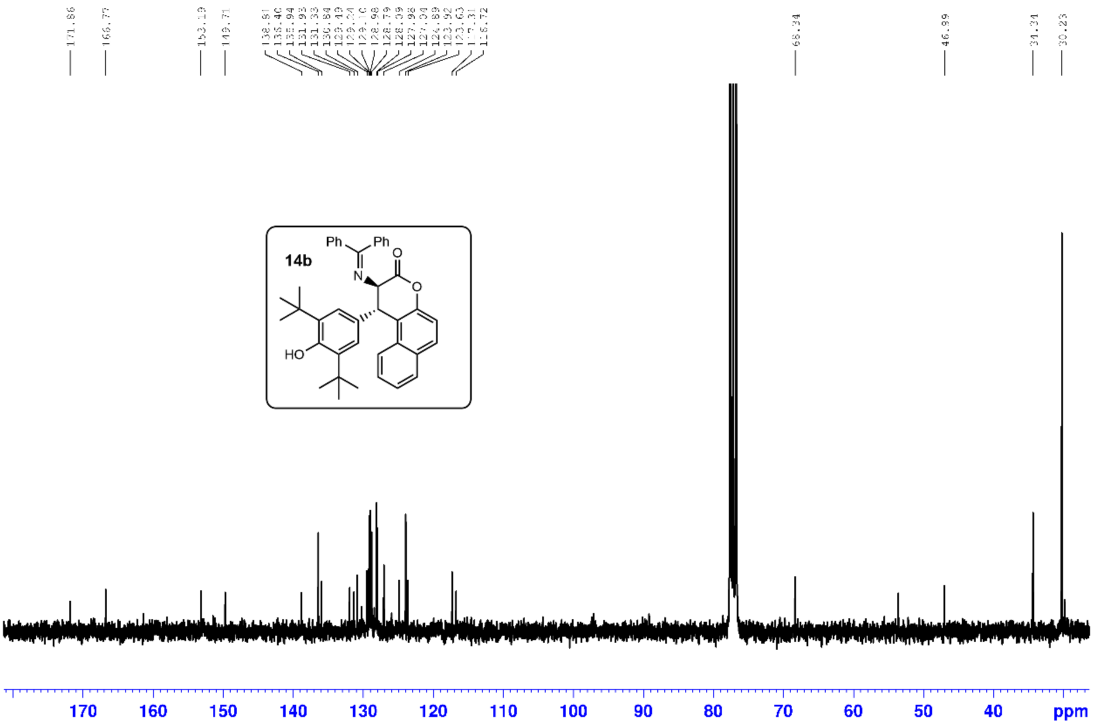

**Chromatogram**

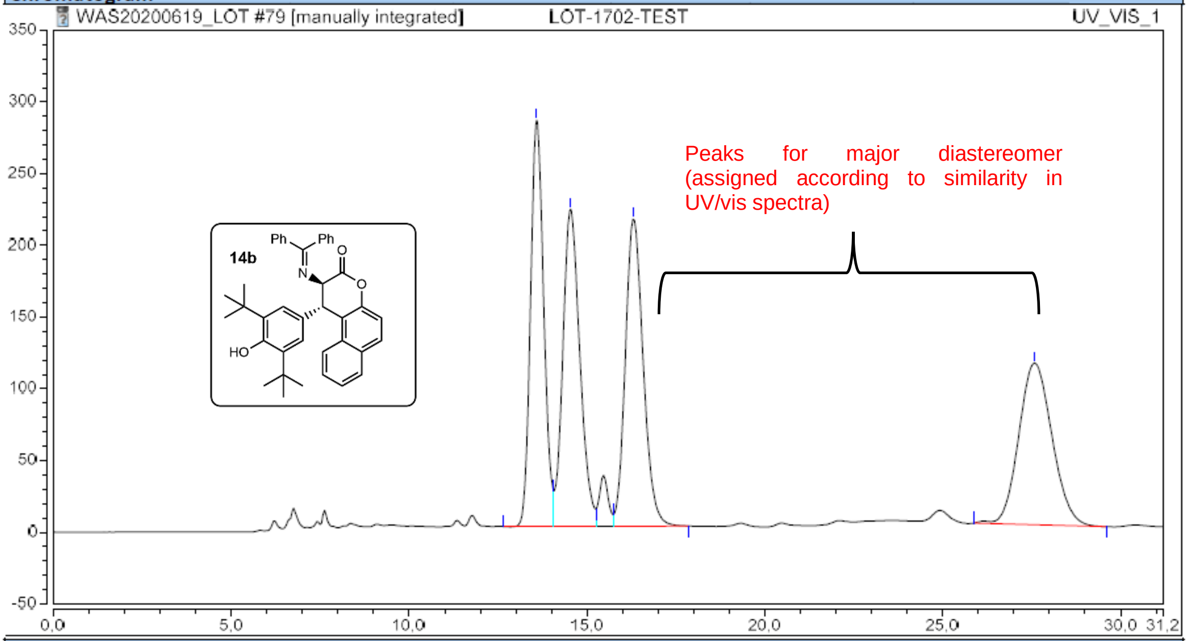


**Integration Results**

| No. | Peak Name | Retention Time min | Area mAU*min | Height mAU | Relative Area % | Relative Height % | Amount n.a. |
|---|---|---|---|---|---|---|---|
| 1 | | 13,590 | 121,936 | 283,243 | 24,79 | 34,06 | n.a. |
| 2 | | 14,540 | 122,881 | 221,099 | 24,98 | 26,59 | n.a. |
| 3 | | 16,310 | 125,073 | 214,584 | 25,42 | 25,80 | n.a. |
| 4 | | 27,580 | 122,039 | 112,740 | 24,81 | 13,56 | n.a. |
| **Total:** | | | **491,929** | **831,666** | **100,00** | **100,00** | |

**Chromatogram**

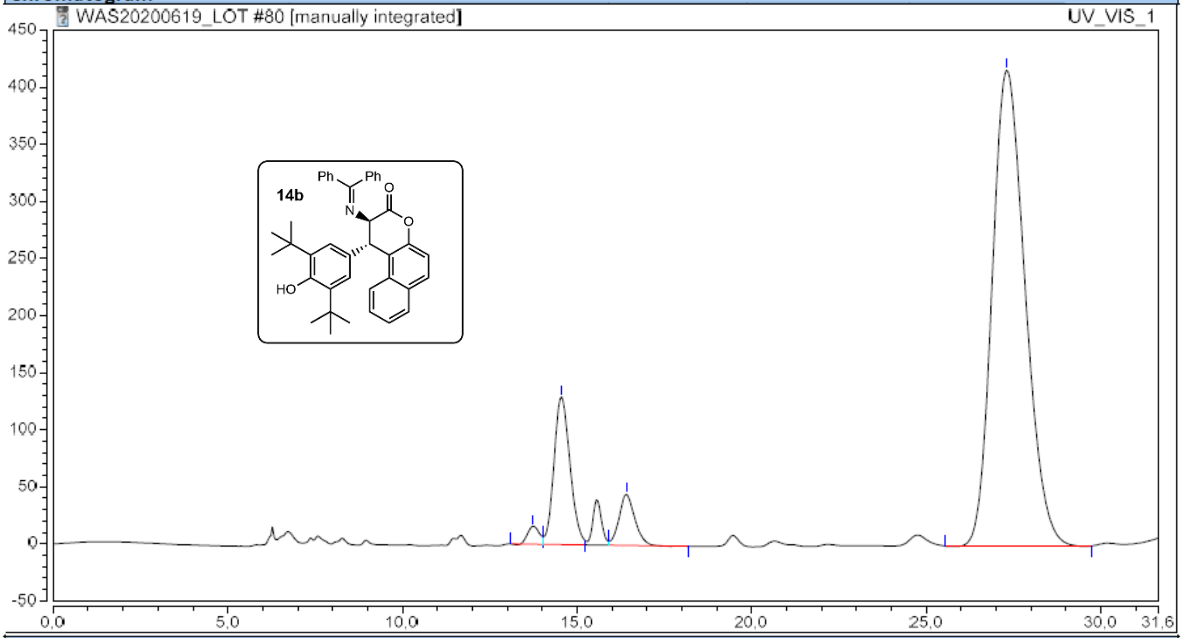


**Integration Results**

| No. | Peak Name | Retention Time min | Area mAU*min | Height mAU | Relative Area % | Relative Height % | Amount n.a. |
|---|---|---|---|---|---|---|---|
| 1 | | 13,747 | 5,910 | 15,770 | 1,07 | 2,60 | n.a. |
| 2 | | 14,550 | 68,894 | 129,342 | 12,47 | 21,32 | n.a. |
| 3 | | 16,410 | 23,397 | 44,786 | 4,24 | 7,38 | n.a. |
| 4 | | 27,307 | 454,132 | 416,660 | 82,22 | 68,69 | n.a. |
| **Total:** | | | **552,333** | **606,558** | **100,00** | **100,00** | |
